# Proteogenomic Analysis Greatly Expands the Identification of Proteins Related to Reproduction in the Apogamous Fern *Dryopteris affinis* ssp. *affinis*

**DOI:** 10.3389/fpls.2017.00336

**Published:** 2017-03-22

**Authors:** Jonas Grossmann, Helena Fernández, Pururawa M. Chaubey, Ana E. Valdés, Valeria Gagliardini, María J. Cañal, Giancarlo Russo, Ueli Grossniklaus

**Affiliations:** ^1^Functional Genomics Center ZurichZürich, Switzerland; ^2^Area of Plant Physiology, Department of Organisms and Systems Biology (BOS), Oviedo UniversityOviedo, Spain; ^3^Institute of Plant and Microbial Biology, Zurich-Basel Plant Science Center, University of ZurichZürich, Switzerland; ^4^Physiological Botany, Uppsala BioCenter, Uppsala UniversityUppsala, Sweden; ^5^Linnean Centre for Plant BiologyUppsala, Sweden

**Keywords:** apogamy, apomixis, *Dryopteris affinis* ssp. *affinis*, fern, gametophyte, proteogenomics

## Abstract

Performing proteomic studies on non-model organisms with little or no genomic information is still difficult. However, many specific processes and biochemical pathways occur only in species that are poorly characterized at the genomic level. For example, many plants can reproduce both sexually and asexually, the first one allowing the generation of new genotypes and the latter their fixation. Thus, both modes of reproduction are of great agronomic value. However, the molecular basis of asexual reproduction is not well understood in any plant. In ferns, it combines the production of unreduced spores (diplospory) and the formation of sporophytes from somatic cells (apogamy). To set the basis to study these processes, we performed transcriptomics by next-generation sequencing (NGS) and shotgun proteomics by tandem mass spectrometry in the apogamous fern *D. affinis* ssp. *affinis*. For protein identification we used the public viridiplantae database (VPDB) to identify orthologous proteins from other plant species and new transcriptomics data to generate a “species-specific transcriptome database” (SSTDB). In total 1,397 protein clusters with 5,865 unique peptide sequences were identified (13 decoy proteins out of 1,410, protFDR 0.93% on protein cluster level). We show that using the SSTDB for protein identification increases the number of identified peptides almost four times compared to using only the publically available VPDB. We identified homologs of proteins involved in reproduction of higher plants, including proteins with a potential role in apogamy. With the increasing availability of genomic data from non-model species, similar proteogenomics approaches will improve the sensitivity in protein identification for species only distantly related to models.

## Introduction

**Figure 1:**
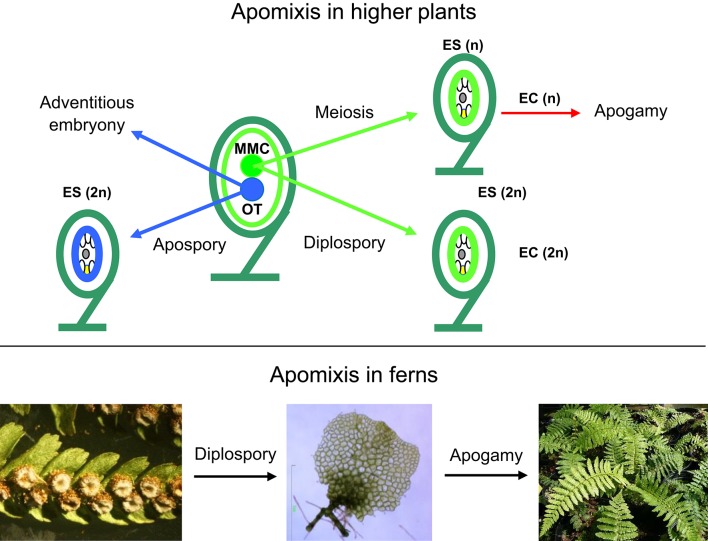
**Types of apomixis in higher plants (top)** and ferns **(botton)**. EC, egg cell; ES, embryo sac; MMC, megaspore mother cell; OT, other tissues such as nucellus or integuments; Bar 1 mm. (Photograph by Helena Fernández).

Most angiosperms reproduce sexually through seeds, but there are examples of asexual seed formation (apomixis), where seeds form without meiosis and fertilization (Figure 1). Apomictic plants produce clonal embryos by sporophytic or gametophytic apomixis (Nogler, 1984; Koltunow and Grossniklaus, 2003). In sporophytic apomixis, the embryo forms directly from the somatic diploid ovule tissue (nucellus or integument). In gametophytic apomixis, the multicellular embryo sac may originate from two different cellular lineages leading to a broad categorization of this developmental program into diplospory and apospory. In diplospory, the embryo sac originates from the megaspore mother cell, either directly by mitosis or after restitution during meiosis, while in apospory the embryo sac originates from nucellar cells. In both cases, the asexual embryo develops from the unreduced egg cell without fertilization (parthenogenesis). Because apomixis allows the fixation of complex genotypes, including that of highly productive F1 hybrids, many researchers have extolled the tremendous potential that apomixis holds for plant improvement (Spillane et al., 2004). In apogamy, somatic cells of the gametophyte are reprogrammed to start the sporophytic developmental program. Apogamy does not occur naturally in angiosperms but is frequent in ferns (Yang and Zhou, 1992; Okano et al., 2009). Apogamy may be obligate, when gametophytes produce non-functional gametes, facultative, or induced by exogenous factors (Fernández et al., 1996; Menéndez et al., 2006a; Cordle et al., 2007). In obligate apogamy, endomitosis prior to meiosis serves to maintain the sporophytic chromosome number throughout the life cycle (Manton, 1950; Sheffield et al., 1983).

Over the last decade, several studies focusing on apomixis in model species of angiosperms concluded that sexual and apomictic pathways share gene expression profiles and, thus, common molecular regulatory features, indicating that they are not distinct pathways (Grossniklaus et al., 2001; Tucker et al., 2003). However, how somatic cells, either of sporophytic or gametophytic (apogamy) origin, become embryogenic is unknown. Apogamy in ferns is easy to observe and the gametophyte of apogamous ferns can be useful for comparison with the gametophytic events in angiosperms (Cordle et al., 2010). Although ferns receive comparatively little attention and genome sequences of ferns are, so far, unavailable, it is accepted that we need to extend our analyses to more phylogenetic branches (Barker and Wolf, 2010). To date, only few fern species have been used to study developmental processes (Whittier, 1971; Wen et al., 1999; Salmi et al., 2005, 2010; Kaźmierczak, 2010; Lopez and Renzaglia, 2014; Valledor et al., 2014; de Vries et al., 2015).

*Dryopteris affinis* (Lowe) Fraser-Jenkins ssp. *affinis* (Western scaly male fern) is a diploid, apomictic fern, which originated from a cross between the sexual ancestor of the extant apomict *D. wallichiana* (Wallich's wood fern) and the sexual *D. oreades* (mountain male fern; Fraser-Jenkins, 1986). The gametophyte of this species forms male but no female reproductive organs and, when cultured *in vitro*, reproduces by apogamy. Once the gametophyte becomes heart-shaped, a brown organization center develops near to the apical indentation that directly forms an apogamous embryo sporophyte (Fernández et al., 1996; Menéndez et al., 2006a).

An alternative for examining gene expression in species without a genome sequence is to study its end products, the proteins (Miernyk et al., 2011). Moreover, RNA and protein profiling technologies have recently been applied in parallel to improve protein identification in proteomic studies (Desgagne-Penix et al., 2010; Lundberg et al., 2010). This has led to an emerging field of biological research at the intersection of proteomics and genomics referred to as proteogenomics, which can be used to either refine genome annotation in order to identify novel translated products or to assign and identify more spectra and, therefore, identify more proteins (Ansong et al., 2008). During the last years novel sequencing technologies, such as RNA-seq, besides high-throughput MS–based proteomics have sped-up proteogenomic research (Helmy et al., 2012). However, there is no available public web resource for mining the genomic and transcriptomic data of fern (Aya et al., 2015).

The goal of the present study is to create an extensive protein resource for the gametophyte of *D. affinis* spp. *affinis* that will be used to gain insights into the molecular basis of apogamy. Our proteogenomic approach, using a species lacking an annotated genome, increased four times the number of indentified peptides as compared to using only publicly available data bases and allowed us to identify a total of 1,397 protein clusters with 5,865 unique peptide sequences. All the raw RNA sequencing files in fastq format and the *de novo* transctipome assembly in fasta format have been deposited at the European Nucleotide Archive (ENA), accession number PRJEB18522, and all proteomics raw data and the relevant derived files have been deposited at ProteomeXchange Consortium via the PRIDE partner repository with the dataset identifier PXD005423.

## Materials and methods

### *In vitro* culture of spores and gametophytes

Spores of *D. affinis* ssp. *affinis* obtained from sporophytes growing in the forest of Turón (Asturias, Spain) were soaked in water for 2 h and then washed for 10 min with a solution of NaClO (0.5%) containing Tween 20 (0.1%). Then, they were rinsed three times with sterile distilled water. Spores were centrifuged at 1,300 g for 3 min between rinses, and then cultured in 500-ml Erlenmeyer flasks containing 100 mL of Murashige and Skoog (MS) medium (Murashige and Skoog, 1962), supplemented with 2% sucrose (w/v), pH 5.7.

**Figure 2:**
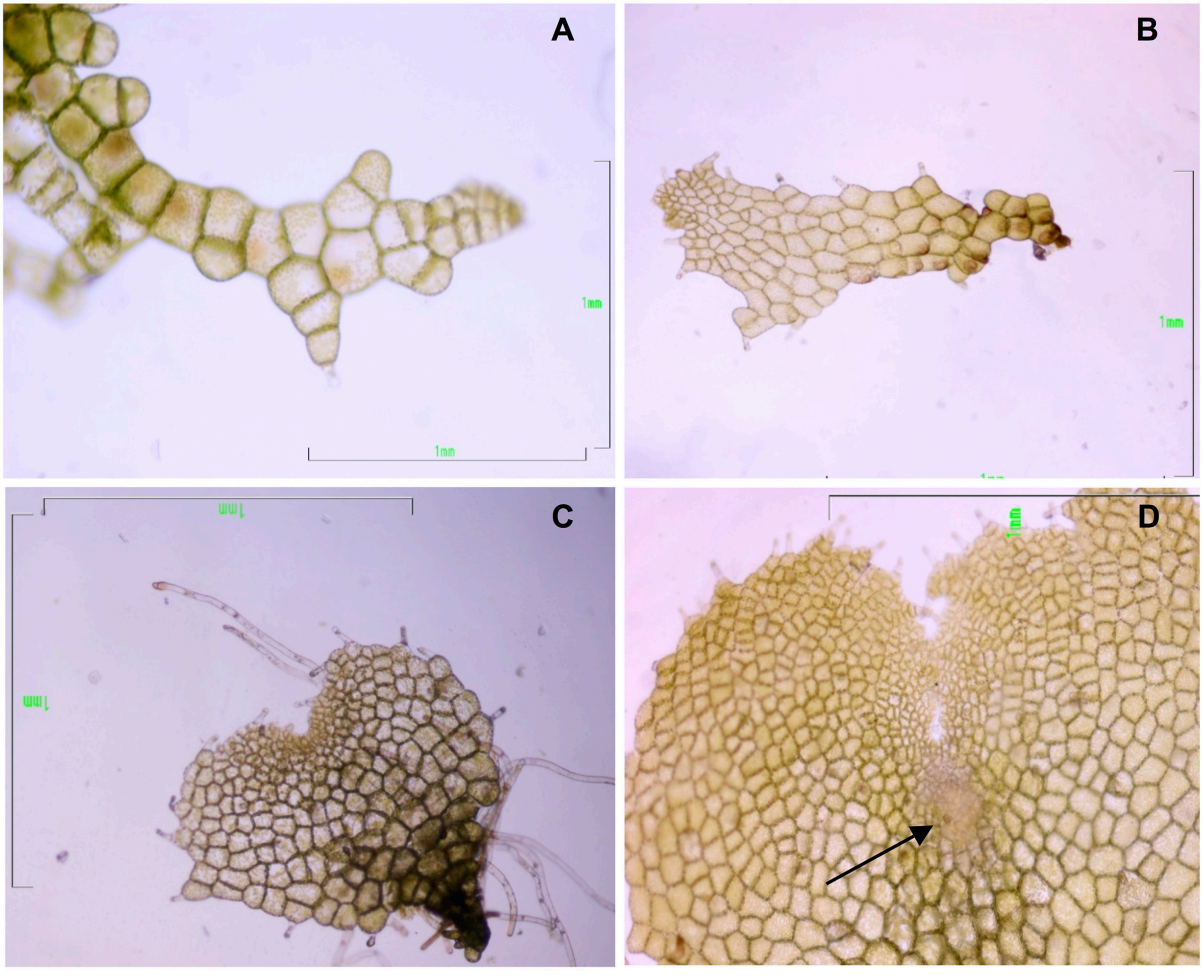
**Gametophytes of *Dryopteris affinis* ssp. *affinis* cultured on MS medium, at different developmental stages: (A)** filamentous, **(B)** spatula, and **(C)** heart-shaped. **(D)** Heart-shaped prothallium with an apogamic center (indicated by the arrow). Bar 1 mm (Photograph by Helena Fernández).

Gametophytes at three developmental stages—filamentous, spatula, and heart (in the last stage with visible signs of an evolving apogamic center)—were collected to carry out the molecular analyses (Figure 2). Cultures of filamentous gametophytes were obtained by maintaining the spores in liquid cultures placed on a gyratory shaker (75 rpm) for 50 days. Cultures of spatula and heart stage gametophytes were cultured in Petri dishes with 25 mL of MS medium containing 2% sucrose (w/v) and 0.7% agar, pH 5.7, for 65 days. All cultures were maintained at 25°C under cool-white fluorescent light (40 μmolm^−2^s^−1^) with a 16-h photoperiod. For RNA extraction, 100 mg of fresh plant material was weighed, immediately frozen in liquid nitrogen, and kept at −80°C until use. For proteomic analyses, gametophytes were lyophilized and kept at −20°C until use. Three biological replicates were used for RNA sequencing and two biological replicates were used for proteomics.

### RNA extraction

Plant material, 100 mg of gametophytes at specific stages, was homogenized by adding glass beads to an Eppendorf tube and shaking with a Silamat S5 shaker (Ivoclar Vivadent, Schaan, Liechtenstein) twice during 10 and 5 s, respectively. Total RNA was isolated using the Spectrum™ Plant Total RNA kit (Sigma-Aldrich, Buchs, Switzerland). DNA was removed using the TURBO DNA-free kit (Life Technologies, Carlsbad, CA), and checked to determine quality using the Bioanalyser Agilent RNA 6000 Pico Kit (Agilent Technologies, Waldbronn, Germany).

### Library preparation

The quality of the isolated RNA was determined with a Qubit® (1.0) Fluorometer (Life Technologies, California, USA) and a Bioanalyzer 2100 (Agilent Technologies, Waldbronn, Germany). Only those samples with a 260/280 nm ratio between 1.8–2.1 and a 28/18S ratio within 1.5–2.0 were further processed. The TruSeq RNA Sample Prep Kit v2 (Illumina, San Diego, CA) was used in successive steps. Briefly, total RNA samples (100–1,000 ng) were enriched for polyA RNA and then reverse-transcribed into double-stranded cDNA. The cDNA samples were fragmented, end-repaired, and polyadenylated before ligation of TruSeq adapters (Table S1) containing the index for multiplexing. Fragments containing TruSeq adapters on both ends were selectively enriched with PCR. The quality and quantity of the enriched libraries were validated using Qubit® (1.0) Fluorometer and the Caliper GX LabChip® GX (Caliper Life Sciences, Hopkinton, MA). The products resulted in a smear with an average fragment size of approximately 260 bp. The libraries were normalized to 10 nM in Tris-Cl 10 mM, pH8.5, with 0.1% Tween 20.

### Cluster generation, sequencing, *De novo* assembly, transcriptome coverage, and data quality

Each of the six samples (filamentous and heart tissues, three samples each) was sequenced on the Illumina HiSeq 2000 employing a 2 × 100 bp protocol. The number of raw reads generated was in the range 70–92 M. The fastq files were preprocessed using far (https://wiki.gacrc.uga.edu/wiki/FAR), the predecessor to flexbar (http://sourceforge.net/projects/flexbar/). The minimum length was set to 50 bp and adapters were trimmed as long as they would overlap 5 bases with the read. The reads passing these filters were then joined using fastqjoin (https://pods.iplantcollaborative.org/wiki/display/DEapps/Fastq-Join) so to maximize the length of the reads prior to the transcriptome assembly.

The joined reads were then passed onto Trinity (version 2013-02-25, http://trinityrnaseq.sourceforge.net) for the *de novo* transcriptome assembly with default settings.

The total number of putative transcripts generated by Trinity was 436,707.

Relative abundances of the transcripts originating from the different samples were estimated using RSEM (http://www.biomedcentral.com/1471-2105/12/323) by mapping to the newly generated transcriptome and differential expression, both at isoform and gene level, was measured with EBseq (http://www.biostat.wisc.edu/~kendzior/EBSEQ/).

The putative 436,707 transcripts as generated by Trinity were 6-frame translated using six pack (http://emboss.sourceforge.net/apps/release/6.6/emboss/apps/sixpack.html). 330,049 amino acid (AA) sequences longer than 60 AA were kept in the NGS database (DB). To add some minimal annotation to our NGS DB sequences, each was blasted (blastp) against the Swissprot DB, a well curated multi-species database where most of the proteins have an associated function. The description line of the corresponding SSTDB entry was extended if the best scoring BLAST hit was found with an *e*-value of 1E-4 or smaller. This cross species annotation of the closest BLAST hit should be seen dynamic (while the actual sequences are rather static): since databases get better curated overtime, there might be better homologs to annotate our sequences in the future.

### Protein extraction

From each of the four samples (filamentous and heart tissues, two samples each) an amount of 20 mg dry weight of plant gametophytes were homogenized using a Silamat S5 shaker (Ivoclar Vivadent, Schaan, Liechtenstein). Homogenized samples were solubilized in 800 μL of buffer A [0.5 M Tris-HCL pH 8.0, 5 mM EDTA, 0.1 M Hepes-KOH, 4 mM DTT, 15 mM EGTA, 1 mM PMSF, 0.5% PVP and 1 × protease inhibitor cocktail (Roche, Rotkreuz, Switzerland)] using a Potter homogenizer (Thermo Fisher Scientific, Bremen, Germany). Proteins were extracted in two steps: first, the homogenate was subjected to centrifugation at 16,200 g for 10 min at 4°C on a tabletop centrifuge and, second, the supernatant was subjected to ultracentrifugation at 117–124 kPa (~100,000 g) for 45 min at 4°C. Post-ultracentrifugation the supernatant contained the soluble protein fraction. The pellet from the first ultracentrifugation was re-dissolved in 200 μL of buffer B (40 mM Tris base, 40 mM DTT, 4% SDS, 1 × protease inhibitor cocktail (Roche, Rotkreuz, Switzerland) to extract membrane proteins using the ultracentrifuge as described before. The supernatant after the second ultracentrifugation step contained the membrane protein fraction. Ultracentrifugation was performed using an Airfuge (Beckman Coulter, Pasadena, CA). Protein concentrations were determined using a Qubit Fluorometer (Invitrogen, Carlsbad, CA).

### 1D gel electrophoresis

Approximately 1 mg protein per each soluble and membrane fraction was loaded separately onto a 0.75 mm tick, 12% SDS-PAGE mini-gel. Samples were treated with sample loading buffer and 2 M DTT, heated at 99°C for 5 min, followed by a short cooling period on ice, and loaded onto the gel. 1D gel electrophoresis was performed at 150 V and 250 mA for 1 h in 1X Running Buffer.

### Protein separation and in-gel digestion

After 1D SDS-PAGE each gel lane was cut into six 0.4 cm wide sections using a custom-made gel cutter, resulting in 48 slices. These slices were further fragmented into smaller pieces and subjected to 10 mM DTT (in 25 mM AmBic pH8) for 45 min at 56°C and 50 mM Iodoacetamide for 1 h at RT in the dark prior to trypsin digestion at 37°C overnight (Baerenfaller et al., 2008). The small pieces were washed twice with 100 μl of 100 mM NH_4_HCO_3_/50% acetonitrile, and washed once with 50 μl acetonitrile. All three supernatants were discarded and peptides digested with 20 μl trypsin (5 ng/μl in 10 mM Tris/2 mM CaCl_2_, pH 8.2) and 50 μl buffer (10 mM Tris/2 mM CaCl_2_, pH 8.2). After microwave-heating for 30 min at 60°C, the supernatant was removed and gel pieces extracted once with 150 μl 0.1% TFA/50% acetonitrile. All supernatants were combined and dried, and samples were then dissolved in 15 μl 0.1% formic acid/3% acetonitrile and transferred to auto-sampler vials for liquid chromatography (LC)-MS/MS where 5 μl were injected.

### Mass spectrometry and peptide identification (Orbitrap XL)

The samples were analyzed on a LTQ Orbitrap mass spectrometer (Thermo Fisher Scientific, Bremen, Germany) coupled to an Eksigent Nano HPLC system (Eksigent Technologies, Dublin, CA). Solvent composition of buffer A was 0.2% formic acid/1% acetonitrile, and of buffer B 0.2% formic acid/99.8% acetonitrile. Samples were dissolved in 3% acetonitrile/0.1% formic acid. Peptides were loaded onto a self-made tip column (75 μm × 80 mm) packed with reverse phase C18 material (AQ, particle size 3 μm, 200 Å) (Bischoff GmbH, Leonberg, Germany) and eluted at a flow rate of 200 nL per min. The following LC gradient was applied: 0 min: 5% buffer B, 56 min: 40% B, 60 min: 47% B, 64 min: 97% B, 71 min: 97% B. Mass spectra were acquired in the m/z range 300–2000 in the Orbitrap mass analyzer at a resolution of 60,000 at m/z 400. MS/MS spectra were acquired in a data-dependent manner from the five most intense signals in the ion trap, using 28% normalized collision energy and an activation time of 30 ms. The precursor ion isolation width was set to m/z 3.0. Charge state screening was enabled, and singly charged precursor ions and ions with undefined charge states were excluded. Precursor masses already selected for MS/MS acquisition were excluded from further selection for 120 s. MS/MS spectra were converted to the Mascot generic format (.mgf) using MascotDistiller 2.3.2 and the parameters recommended for Orbitrap instruments. These .mgf files were submitted to Mascot (Matrix Science, London UK; version 2.4.01) for searching. Trypsin was selected as the proteolytic enzyme, Mascot was set up to search against the in-house generated SSTDB (forward entries: 330,049) combined with the publicly available VPDB (forward entries: 1,031,407, downloaded from uniprot.org in March 2012), and a set of 260 known mass spectrometry contaminants in a target-decoy strategy (using reversed protein sequences). The concatenated DB is available online (http://fgcz-r-021.uzh.ch/fasta/p1222_combo_NGS_n_Viridi_20160205.fasta). Data was searched with a fragment ion mass tolerance of ± 0.6 Da and a precursor mass tolerance of ±10 ppm. A maximum of 2 missed cleavages were allowed. Carbamidomethylation of cysteine was specified as a fixed modification, and deamidation (N, Q), Gln->pyro-Glu (N-term Q), oxidation (M) were specified in Mascot as variable modifications.

### Protein identification, verification, and bioinformatic downstream analysis

Scaffold software (version Scaffold 4.2.1, Proteome Software Inc., Portland, OR) was used to validate MS/MS-based peptide and protein identifications. Mascot results were analyzed together using the MudPIT option. Peptide identifications were accepted if they scored better than 95.0% probability as specified by the PeptideProphet algorithm with delta mass correction, and protein identifications were accepted if the ProteinProphet probability was above 95%. Proteins that contained same peptides and could not be differentiated based on MS/MS alone were grouped to satisfy the principles of parsimony using scaffolds cluster analysis option. Only proteins that met the above criteria were considered positively identified for further analysis. The amount of random matches was evaluated by performing the Mascot searches against a database containing decoy entries and checking how many decoy entries (proteins or peptides) passed the applied quality filters. The peptide FDR and protein FDR was estimated at 0.21 and 0.93% respectively, indicating the stringency of the analysis. A total of 2,525 unique proteins were assembled into 1,397 protein clusters using Scaffold. The Spectrum Report from Scaffold satisfying the criteria mentioned above was exported and for each identified peptide-spectrum-match (PSM) and each peptide, the origin of the DB (being either from the VPDB, the SSTDB, or identified in both DBs) was evaluated. PSMs for which more than one hit was generated with exactly the same score but a different peptide sequence were considered as conflicts and omitted from subsequent analyses. These are cases where the AA composition of the two assignments are the same but the first two or three residues are permutated or represent Leu/Ile switches as these are isobaric AAs. All proteomics data have been deposited to the ProteomeXchange Consortium via the PRIDE partner repository with the dataset identifier PXD005423 (Vizcaíno et al., 2016).

## Results

### Using a *De Novo* generated SSTDB greatly improves peptide identification in the proteome of *D. affinis*

The gametophytic tissue of the fern *D. affinis* was used to generate its proteomic profile by using LC-MS/MS. The spectra were searched against a concatenated VPDB in addition to the new protein DB that was created based on transcriptomic datasets obtained in the present study (SSTDB) in order to identify PSMs from any of the two databases. This search database is large for a single organism and, therefore, probably redundant and biased to an inherent problem of the proteogenomic approach where some transcripts may not be completely assembled and, therefore, result in shorter sequences in general. To back-up this observation, we compared sequence lengths in SSTDB to VPDB and other organism-specific databases (Figure S1). It can be seen that on average the sequences in the SSTDB are clearly shorter. The large size of the SSTDB is also a result of the six-frame translation where usually only one of the six translations is correct.

Because of the lack of a completely species-specific annotated genome, we used the concatenated SSTDB and VPDB, which increases the chance for random matching due to the large search space generated. Thus, higher scores are required for individual PSMs compared to searching smaller databases. Here, we did not want to omit the full VPDB but accepted the loss of some peptide/protein identifications.

**Figure 3:**
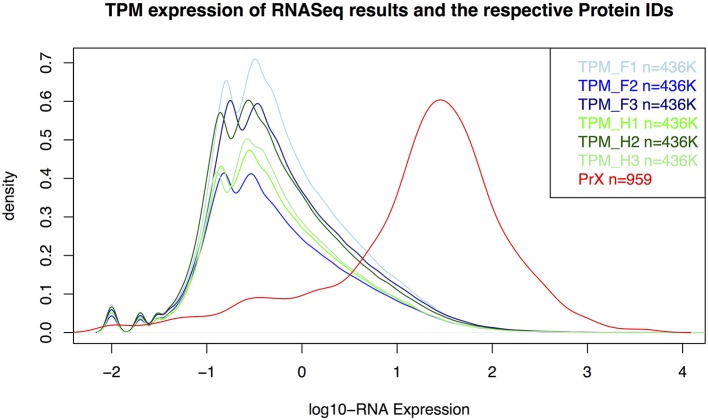
**Distribution for RNA-Seq counts (TPM) and RNA-expression values of the identified proteins in the gametophyte of *Dryopteris affinis* ssp. *affinis*.** F, filamentous; H, heart stage; PrX, proteomics; TPM, transcripts per million.

**Figure 4:**
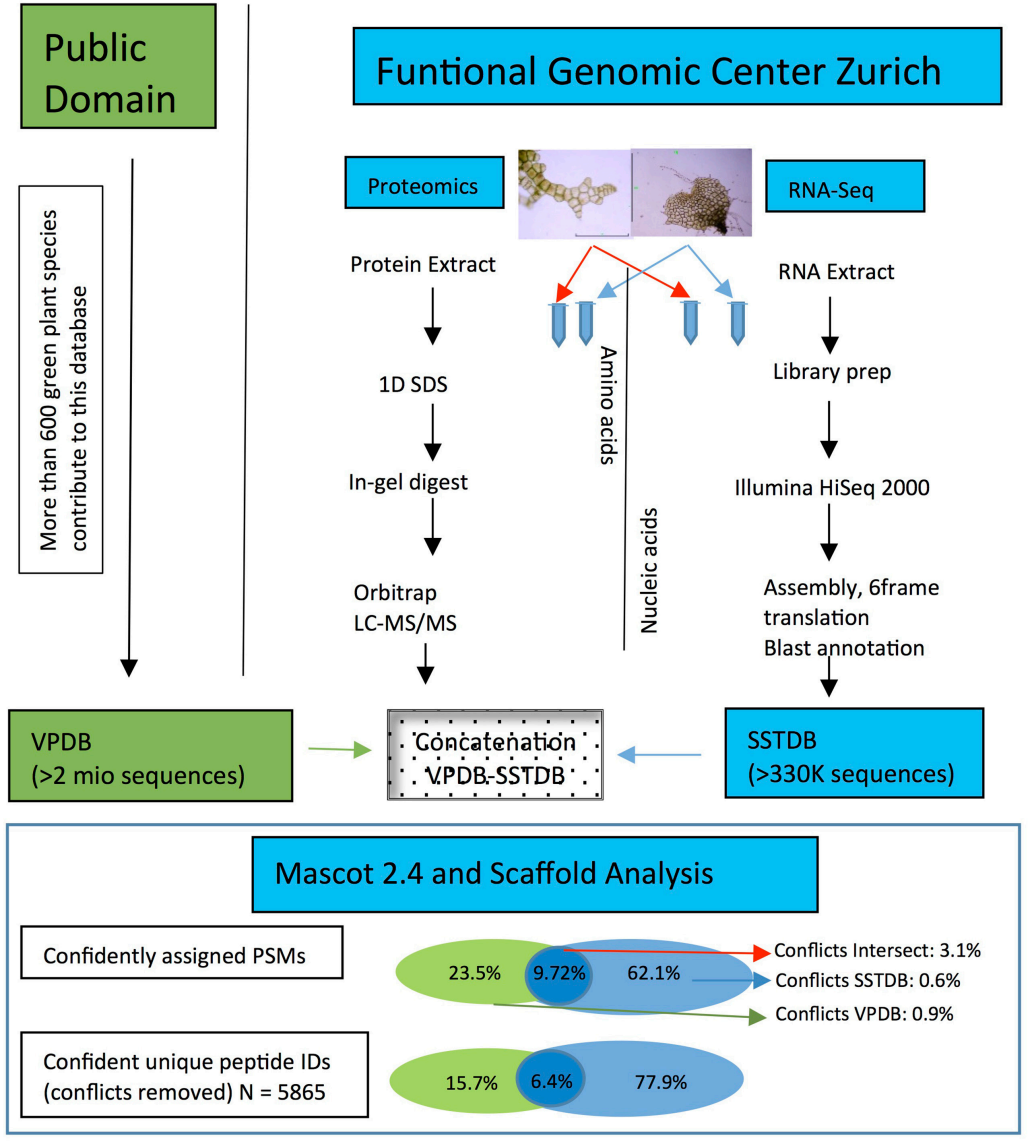
**Workflow showing the steps and the importance to make a transcriptome database for the non-sequenced species *Dryopteris affinis* ssp. *affinis*, in contrast to only using public resources**. The criteria for protein identification and definition of conflicts are laid out under “Experimental Procedures.”

As expected, proteins were more easily detected if they are more abundant (assuming correlation of transcript and protein abundance; Figure 3). The combination of both transcriptome and proteome methodologies yielded a total 1,397 true forward protein clusters with 5,865 unique peptide sequences identified (protFDR 0.93%; Table S2). The strategy of searching against an orthologue DB (VPDB) concatenated to a newly generated protein DB derived from species-specific transcriptome data (SSTDB) dramatically improved protein identification. Of all uniquely identified peptide sequences, more than 77.8% were exclusively matched in the SSTDB, while only about 15.7% were exclusively matched in the VPDB (Table 1). The intersection of peptides identified in both DBs was ca 6% after removing conflicting assignments. This is also obvious at the protein cluster level: more than 1,068 clusters (76.45%) were exclusively identified in the SSTDB, while only 329 clusters (23.55%) would have been identified if we had searched only against the VPDB. The intersection revealed 167 clusters (11.95%), which leaves only 162 clusters (11.6%) that are exclusively identified in the VPDB. This represents about 3.8 times more peptide sequences that could be identified using this proteogenomics approach as compared to using VPDB alone. The overview of the full experiment workflow is illustrated in Figure 4. A list of all proteins identified in this study is provided in Table S2 or the Scaffold file (.sf3), which can be downloaded from the PRIDE repository.

**Table 1:** **Occurrence of assigned peptide-spectrum-matches (PSM) and unique peptide sequences**.

	**Assigned PSM (Peptide-spectrum-matches)**	**Identified unique peptides *(Conflicts removed)***
Total assignments (%)	22,905 (100%)	5,865 (100%)
*Conflicting (%)*	*1,075 (4.69%)*	
Assigned with VPDB and SSTDB—no conflicts (%)	2,227 (9.72%)	376 (6.41%)
*Conflicting (%)*	*719 (3.14%)*	
Assigned ONLY with SSTDB—no conflicts (%)	14,222 (62.09%)	4,566 (77.85%)
*Conflicting (%)*	*140 (0.61%)*	
Assigned ONLY with VPDB -no conflicts (%)	5,381 (23.49%)	923 (15.74%)
*Conflicting (%)*	*216 (0.94%)*	

*The source of the identification i.e. the newly generated SSTDB or the publicly available VPDB, is depicted for each case. The conflicting PSM (different sequences which score exactly the same due to e.g. isobaric amino acid switches, Leu/Ile, or permutations in the first two or three residues) are indicated in italics*.

### Functional annotation reveals a high metabolic activity of *D. affinis* gametophytes

To gain information about possible functions of the proteins identified within VPDB, we assigned them to *gene ontology* (*GO*) functional categories (“biological process,” “molecular function,” “cellular component”). Our data reveal the usual behavior in a shotgun proteomics approach, in which proteins of high abundance are predominantly identified; however, some interesting categories that emphasize the nature of the tissue under investigation were also observed.

Under “biological process” the *GO* categories include “cellular processes” and “development and differentiation” as expected for developing gametophytes. The proteome of *D. affinis* gametophytes is dominated by processes that indicate a high metabolic activity. In addition, proteins involved in “regulation,” “defense,” “response to stimulus,” and “signaling,” reflect the intensive interactions of free living gametophytes with their environment.

Under “molecular functions” three *GO* categories dominate, namely “ion binding,” “enzyme activity,” and “nucleotide binding,” while under “cellular components” we mostly found proteins localized to “plastids” and “cytoplasm,” but also to the “nucleus” and “membrane” compartments. Proteins from virtually all cellular compartments as well as the extracellular cell wall were identified (data not shown).

Finally, we also identified proteins without a *GO* annotation, among others the Coiled-coil domain-containing protein 18, Elicitor-responsive protein 3, GEM-like protein 1, LEA1, UPF0763 protein NAMH 0545, and the B2 protein.

### *D. affinis* gametophytes contain proteins with similarity to plant, animal, and fungal proteins

**Figure 5:**
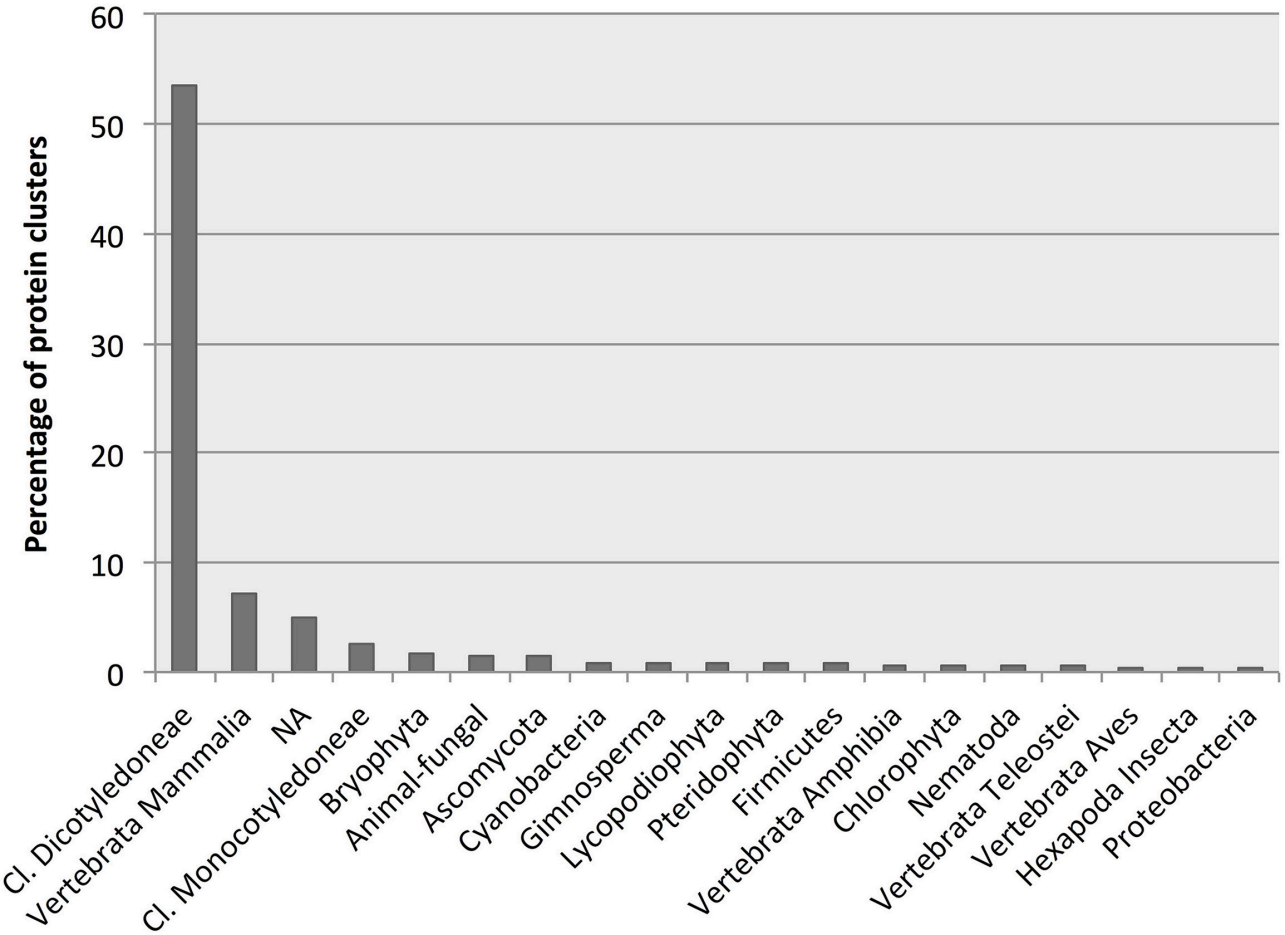
**Number of protein clusters obtained from gametophytes of *Dryopteris affinis* ssp. *affinis* with the best hits to species belonging to the phylogenetic groups indicated**.

More than half of all identified *D. affinis* proteins had BLAST hits to proteins from higher plants, followed by hits from animals, not mapped entries, and lower plants and algae as the most abundant (Figure 5). Table 2 shows the best species match for proteins identified within the VPDB or the species used to extend the description with useful annotation from SSTDB for up to a cumulative 70% of all identified proteins. For proteins identified within the SSTDB, we indicate the category and *e*-value for the BLAST annotation. Interestingly, for the identified proteins most of the BLAST hits were found with small *e*-values (*e* < 1E-20). In contrast to the complete database where most of the BLAST hits were found with an *e*-value above 1E-6 (Table S3).

**Table 2:** **Best matching species for all the proteins identified and the respective *e*-value category if the identification was identified with the SSTDB and having a blastp homolog**.

**Species/*E*-value category**	***E*-value<1E-20 (%)**		**1E-20<*E*-value<1E-10**		**1E-10<*E*-value<1E-6 (%)**		**1E-6<*E*-value (%)**		**No_useful blastp_hit**		**Viridi_entry (%)**		**Grand total (%)**		**Cumulative total (%)**
Arabidopsis thaliana	94.75	577	0.66	4	0.49	3	0.49	3			3.61	22	26.61	609	26.61
NA: Not mapped with blastp									76.67	69			3.93	90	30.54
Oryza sativa	71.76	61									28.24	24	3.71	85	34.25
Physcomitrella patens	1.64	1									96.72	59	2.66	61	36.91
Homo sapiens	81.08	30	5.41	2	8.11	3	5.41	2					1.62	37	38.53
Populus trichocarpa	2.7	1									97.3	36	1.62	37	40.15
Selaginella moellendorffii											97.14	34	1.53	35	41.68
Vitis vinifera	5.71	2									91.43	32	1.53	35	43.21
Bos taurus	93.33	28	3.33	1	3.33	1							1.31	30	44.52
Mus musculus	60	18	16.67	5	13.33	4	10	3					1.31	30	45.83
Glycine max	64.29	18									35.71	10	1.22	28	47.05
Pisum sativum	62.96	17	22.22	6	7.41	2					7.41	2	1.18	27	48.23
Solanum lycopersicum	69.23	18	7.69	2							23.08	6	1.14	26	49.37
Picea sitchensis											100	24	1.05	24	50.42
Zea mays	65.22	15									34.78	8	1	23	51.42
Dictyostelium discoideum	81.82	18	4.55	1	9.09	2	4.55	1					0.96	22	52.38
Nicotiana tabacum	80	16									20	4	0.87	20	53.25
Medicago truncatula											94.74	18	0.83	19	54.08
Solanum tuberosum	89.47	17									10.53	2	0.83	19	54.91
Hordeum vulgare											94.12	16	0.74	17	55.65
Spinacia oleracea	70.59	12									29.41	5	0.74	17	56.39
Ricinus communis	6.25	1									93.75	15	0.7	16	57.09
Schizosaccharomyces	66.67	10	13.33	2	13.33	2	6.67	1					0.66	15	57.75
Sorghum bicolor	6.67	1									86.67	13	0.66	15	58.41
Adiantum capillus-veneris	75	9									25	3	0.52	12	58.93
Acaryochloris marina	100	11											0.48	11	59.41
Arabidopsis lyrata			10	1							80	8	0.44	10	59.85
Bacillus subtilis	60	6	10	1	30	3							0.44	10	60.29
Rattus norvegicus	60	6			20	2	20	2					0.44	10	60.73
Daucus carota	44.44	4	22.22	2							33.33	3	0.39	9	61.12
Triticum aestivum	55.56	5									44.44	4	0.39	9	61.51
Brassica napus	75	6			12.5	1					12.5	1	0.35	8	61.86
Caenorhabditis elegans	87.5	7					12.5	1					0.35	8	62.21
Chlamydomonas reinhardtii	12.5	1									75	6	0.35	8	62.56
Chlorella variabilis											100	8	0.35	8	62.91
Hordeum vulgare	75	6									25	2	0.35	8	63.26
Micromonas sp											100	8	0.35	8	63.61
Xenopus laevis	75	6	25	2									0.35	8	63.96
Cicer arietinum	71.43	5									28.57	2	0.31	7	64.27
Danio rerio	57.14	4	42.86	3									0.31	7	64.58
Hordeum vulgare	71.43	5			14.29	1					14.29	1	0.31	7	64.89
Catharanthus roseus	83.33	5									16.67	1	0.26	6	65.15
Gallus gallus	100	6											0.26	6	65.41
Ostreococcus lucimarinus											83.33	5	0.26	6	65.67
Petunia hybrida	100	6											0.26	6	65.93
Picea abies	100	6											0.26	6	66.19
Volvox carteri											66.67	4	0.26	6	66.45
Cucumis sativus	80	4									20	1	0.22	5	66.67
Drosophila melanogaster	60	3	20	1	20	1							0.22	5	66.89
Escherichia coli	80	4	20	1									0.22	5	67.11
Gossypium hirsutum	20	1									80	4	0.22	5	67.33
Nicotiana plumbaginifolia	80	4									20	1	0.22	5	67.55
Saccharomyces cerevisiae	60	3			40	2							0.22	5	67.77
Anemia phyllitidis	75	3									25	1	0.17	4	67.94
Angiopteris evecta	25	1									75	3	0.17	4	68.11
Asparagus officinalis	50	2									50	2	0.17	4	68.28
Beta vulgaris	75	3									25	1	0.17	4	68.45
Nostoc sp.	100	4											0.17	4	68.62
Polystichum munitum	75	3									25	1	0.17	4	68.79
Pseudomonas putida	50	2					50	2					0.17	4	68.96
Pteris vittata											100	4	0.17	4	69.13
Ricinus communis	100	4											0.17	4	69.3
Synechocystis sp.	75	3			25	1							0.17	4	69.47
Thermosynechococcus	100	4											0.17	4	69.64
Xenopus tropicalis	75	3	25	1									0.17	4	69.81
Aedes aegypti	100	3											0.13	3	69.94

Most hits had similarity to proteins encoded by the best-annotated genomes of higher plants, namely *Arabidopsis thaliana* (mouse ear cress) and the monocot *Oryza sativa* (rice; Table 2). However, there might be a bias here because, as the best annotated plant species, those are the ones with most entries in the swissprot DB. Surprisingly, in an identification based on the SSTDB entry instead of the VPDB entry, they were not followed by other plants with well-annotated genomes, including *Solanum lycopersicum* (tomato) and *Vitis vinifera* (grape), but rather by *Homo sapiens* (human), *Bos taurus* (cattle), and *Mus musculus* (mouse). Apart from these animals and several additional plant species, hits were also identified to proteins from the protozoon *Dictyostelium discoideum* (slime mold) and the fungus *Schizosacharomyces pombe* (fission yeast; Table 2).

Figure S2 shows pairwise alignments for proteins discussed here, which were identified within the SSTDB and had an annotation from BLAST. Figure S3 provides annotated PSMs for proteins for which the basis of identification is a single confident peptide sequence.

Using the Scaffold software and the file provided in the PRIDE repository, GO categories can be visualized for each protein or also compared across samples, and blastp searches can directly be launched at the NCBI homepage.

## Discussion

Plant reproduction is key to understanding plant development but our knowledge on the molecular basis behind asexual reproduction or apomictic developmental programs is scarce. Ferns are frequent apogamous species and as such they can provide valuable information. Studies with an “omics” approach are scarce in ferns due to their complex, large genomes and low agronomic value (Bona et al., 2010; Der et al., 2011; Cordle et al., 2012; Shen et al., 2014; Aya et al., 2015; de Vries et al., 2015). This paper reports the first protein resource for a fern gametophyte, namely the apogamous gametophyte of *D. affinis* ssp. *affinis*. Although no genome sequence is yet available for this non-model species, it could prove useful for future research into the basic principles of apogamy, a process of great importance to agriculture (Spillane et al., 2004).

### Proteogenomics is a powerful approach to identify proteins in proteomic studies of non-model species

Identifying peptides and proteins from non-sequenced organisms has already been examined before. This is always possible based on completely identical peptide sequences between the species under investigation and the species in the search database. This only becomes problematic if the species under investigation is very distantly related to species where protein sequences are available in the search database. In these cases, peptide and protein identification can be performed by estimating the quality of a tandem mass spectrum, and if the quality is sufficient, *de-novo* sequencing followed by MS homology searching (Siddique et al., 2006; Grossmann et al., 2007; Vertommen et al., 2011). The major advantage of first generating a SSTDB is usually the increased sensitivity in the number of protein identifications as well as the number of peptides identified per protein.

In this study, we identified four-times more peptides with high confidence using a SSTDB concatenated with the VPDB than with the public VPDB alone. Although the concatenation of these databases results in a very large database with many homologous entries, our results demonstrate that the combination of proteomic and transcriptomic resources is essential to make adequate biological interpretations. In agreement with previous studies, we show that the sole use of the VPDB—or any other publicly available database for protein identification—is inefficient in non-model species, since they are under-represented in most databases, resulting in poor identification rates (Romero-Rodríguez et al., 2014).

As a result of searching SSTDB concatenated with the VPDB, we could identify about 1,400 protein clusters from gametophytic tissue of *D. affinis*. According to their assigned *GO* category under “biological process,” and according to the functions of mapped orthologous proteins, many proteins are associated with a high metabolic activity in agreement with the free-living nature of fern gametophytes that are photosynthetically active and thus autotroph (Der et al., 2011; Cordle et al., 2012). Similar to what was found in the transcriptomes of the MMC and female gametophyte of the flowering plant *A. thaliana* (Wuest et al., 2010), proteins involved in RNA metabolism and translation also feature prominently in the *D. affinis* proteome.

### *D. affinis* proteins are homologs to proteins involved in reproduction of higher plants

Among the identified proteins, those related to the biology of fern gametophytes are of special relevance to understand apogamy and the molecular basis of asexual reproduction (Table 3). As a reproductive structure, the gametophyte of ferns could be expected to be equivalent to the tissues giving rise to male (pollen) and female gametophytes (embryo sacs) in flowering plants. In line with this, *in silica* expression of the apogamy library *Arabidopsis* homologs, enriched in flower and seed structures, was reported for the apogamous gametophyte of *Ceratopteris richardii* (Cordle et al., 2012). Hence, despite the rapid evolution of reproductive proteins (Swanson and Vacquier, 2002), we found several homologs of proteins implicated in the reproduction of higher plants in the proteome of *D. affinis* gametophytes. In fact, many of the genes involved in development of the flower, for example, have homologs in non-flowering clades, illustrating the importance of examining the basic biology of taxa other than model organisms (Hasebe, 1999). Several proteins identified from the apogamous gametophyte of *D. affinis* have been implicated in embryo development of higher plants (Table 3). Among them are members of the LATE EMBRYOGENESIS ABUNDANT (LEA) type 1 family: embryonic protein DC-8, LEA1, the zygotic DNA replication licensing factor MCM6-A, some receptor-like kinases (RLKs), and the GEM-like protein 1 (Table 3). RLKs, such as those of the SOMATIC EMBRYOGENESIS RECEPTOR KINASE (SERK) subfamily, play a role in the acquisition of embryogenic competence (Hecht et al., 2001; Albertini et al., 2005). We also identified homologs of the leucine-rich repeat (LRR)-RLK GASSHO1, which exhibits uniform expression in the embryo from the globular to the mature stage (Tsuwamoto et al., 2008; Table 3). In addition, proteins involved in plant reproduction were identified, such as the pollen-expressed RLK ligand LAT52 (Tang et al., 2002) and the ubiquitin receptor DA1, controlling seed and organ size through the maternal sporophyte by restricting the period of cell proliferation (Li et al., 2008; Table 3). Furthermore, we identified homologs of animal proteins, such as Janus-B, which regulates somatic sex differentiation in *Drosophila melanogaster* (fruit fly; Yanicostas et al., 1989) and Radial Spoke Head 1 (RSPH1), required for sperm motility in humans (Table 3; Onoufriadis et al., 2014).

**Table 3:** **List of selected proteins from those identified in the gametophyte of *Dryopteris affinis* ssp. *affinis* by transcriptome (SSTDB) or proteome (VPDB) databases**.

**Category**	**# Proteins in cluster**	**Index in supp table**	**Protein accession**	**Origin of protein**	**Best matching swissprot accession**	**Gene name**	**Description**	**eValue (blastp)**	**Unique identified peptide sequences**	**%Coverage**
Cell wall modifications	2	578	313076-61_2_ORF2	SSTDB	Q9ZT66	E134_MAIZE	Endo-1,3;1,4-beta-D-glucanase	1.00E-42	3	15.10
Cell wall modifications	3	693	18361-579_1_ORF2(+5)	SSTDB	Q8VYZ3	PME53_ARATH	Probable Pectinesterase 53	3.00E-119	2	9.02
Embryo development	1	134	306456-65_2_ORF1 [2]	SSTDB	P20075	LEAD8_DAUCA	Embryonic protein DC-8	2.00E-20	11	18.50
Embryo development	1	794	378787-31_3_ORF2	SSTDB	Q9SE96	GEML1_ARATH	GEM-like protein 1	6.00E-82	2	7.59
Embryo development	4	188	363021-38_2_ORF1	SSTDB	C0LGQ5	GSO1_ARATH	LRR receptor-like serine/threonine-protein kinase GSO1	2.00E-99	10	15.70
Embryo development	1	722	245753-102_2_ORF1	SSTDB	Q498J7	MC6ZA_XENLA	Zygotic DNA replication licensing factor mcm6-A	0	3	4.44
Phytohormone signaling	1	840	390256-26_3_ORF2	SSTDB	Q9LJX0	AB19B_ARATH	ABC transporter B family member 19	0	1	2.63
Phytohormone signaling	2	692	24435-507_4_ORF2	SSTDB	P48417	CP74_LINUS	Allene oxide synthase, chloroplastic	1.00E-146	5	10.70
Phytohormone signaling	1	866	254191-97_6_ORF2 (+3)	SSTDB	Q9S9U6	1A111_ARATH	1-aminocyclopropane-1-carboxylate synthase 11	1.00E-87	2	6.58
Phytohormone signaling	1	1158	106730-241_3_ORF2	SSTDB	F4JSZ5	BIG1_ARATH	Brefeldin A-inhibited guanine nucleotide-exchange protein 1	0	1	0.73
Phytohormone signaling	1	762	13447-660_2_ORF1(+1)	SSTDB	Q93ZC5	AOC4_ARATH	Allene oxide cyclase 4, chloroplastic	3.00E-70	1	4.38
Phytohormone signaling	6	356	298264-69_1_ORF1[6]	SSTDB	Q9SK82	U85A1_ARATH	UDP-glycyltransferase 85A1	3.00E-73	5	10.00
Phytohormone signaling	1	902	30962-455_2_ORF1 (+39)	SSTDB	Q9ZTR1	SPD1_PEA	Spermidine synthase 1	0	1	4.36
Phytohormone signaling	2	1109	158601-182_4_ORF1 (+1)	SSTDB	Q8TL44	TRPB2_METAC	Tryptophan synthase beta chain 2	4.00E-170	1	2.37
Phytohormone signaling	1	671	311894-62_3_ORF2	SSTDB	Q0JCU7	ZEP_ORYSJ	Zeaxanthin epoxidase, chloroplastic	3.00E-32	3	9.26
Reproduction	1	1272	41629-395_1_ORF2 (+1)	SSTDB	P0C7Q8	DA1_ARATH	Protein DA1	0	1	4.00
Reproduction	1	1083	43959-384_1_ORF2	SSTDB	P54365	JANB_DROPS	Sex-regulated protein janus-B	7.00E-07	1	6.91
Reproduction	1	737	133239-212_4_ORF2 (+1)	SSTDB	P13447	LAT52_SOLLC	Anther-specific protein LAT52	9.00E-14	2	7.25
Reproduction	1	537	338575-49_3_ORF1	SSTDB	Q6VTH5	RSPH1_CYPCA	Radial spoke head 1 homolog	2.00E-09	2	3.40
Reproduction (Apogamy)	1	1202	429495-5_4_ORF2	SSTDB	Q9XGW1	AGO10_ARATH	Protein argonaute 10	3.00E-161	1	1.85
Reproduction (Apogamy)	1	967	284827-77_1_ORF2	SSTDB	Q9ZVD0	SRRT_ARATH	Serrate RNA effector molecule	0	1	2.89
Reproduction (Apomixis)	8	373	319623-58_1_ORF2	SSTDB	Q42798	C93AI_SOYBN	Cytochrome P450 93A1	5.00E-129	3	7.56
Reproduction (Apomixis)	7	751	101113-248_5_ORF2 (+4)	SSTDB	P42825	DNAJ2_ARATH	Chaperone protein dnaJ 2	0.00E+00	1	2.08
Reproduction (Apomixis)	2	1230	95505-256_3_ORF2	SSTDB	Q1PFK0	FBL32_ARATH	F-box/LRR-repeat protein At1g55660	8.00E-06	2	6.67
Reproduction (Apomixis)	1	1359	200671-137_4_ORF2 (+1)	SSTDB	P92963	RAB1C_ARATH	Ras-related protein RABB1c	3.00E-133	1	6.98
Reproduction (Apomixis)	1	1301	162652-176_2_ORF2	SSTDB	Q9LVI6	RLK90_ARATH	Probable inactive receptor kinase RLK902	0	1	2.22
Stress response	2	490	34681-431_3_ORF2	SSTDB	Q06850	CDPK1_ARATH	Calcium-dependent protein kinase 1	0.00E+00	1	3.16
Stress response	1	241	82340-277_1_ORF2 [3]	SSTDB	Q9AR14	PIP15_MAIZE	Aquaporin PIP1-5	1.00E-173	4	21.50
Stress response	1	1373	319597-581_1_ORF2 (+1)	SSTDB	P4659	LEA14_SOYBN	desiccation protectant protein LEA14 homolog	9.00E-24	1	3.37
Stress response	1	1010	163213-176_3_ORF1 (+1)	SSTDB	P0CW97	PCR3_ARATH	Protein PLANT CADMIUM RESISTANCE 3	1.00E-36	3	17.70
Stress response	1	837	tr|Q5IDA4|Q5IDA4_PINTA	VPDB	NA	NA	Cluster of Cinnamate 4-hydroxylase (Fragment)	NA	1	5.76
Stress response	2	449	272418-85_1_ORF2	SSTDB	P22242	DRPE_CRAPL	Desiccation-related protein PCC13-62	7.00E-96	2	13.30
Stress response	10	1087	213971-126_6_ORF1	SSTDB	P13240	DR206_PEA	Disease resistance response protein 206	3.00E-23	2	9.60
Stress response	2	703	3863-1099_6_ORF1 (+1)	SSTDB	P42761	GSTFA_ARATH	Glutathione S-transferase F10	3.00E-70	1	8.89
Stress response	7	267	sp|P31082|HSP7S_CUCMA	VPDB	NA	NA	Stromal 70 kDa heat shock-related protein, chloroplastic (Fragment)	NA	4	97.10
Stress response	1	673	2203-1350_3_ORF1	SSTDB	Q9SPV5	NEC1_NICPL	Nectarin-1	3.00E-30	3	19.70
Stress response	1	1091	149244-199_3_ORF2	SSTDB	Q94AL8	CRIM1_ARATH	Cold-regulated 413 inner membrane protein 1, Chloroplastic	4.00E-46	1	4.87
Stress response	1	941	307117-64_1_ORF1 (+2)	SSTDB	A4FF33	VGB_SACEN	Virginiamycin B lyase	2.00E-11	2	3.33
Stress response/Embryo development	2	320	249225-100_3_ORF2 [4]	SSTDB	O49816	LEA1_CICAR	Late embryogenesis abundant protein 1	5.00E-09	8	66.00

“*Category” as described in the Discussion Section*.

### Identification of proteins with a potential role in apogamy

In both apomixis and apogamy, unreduced cells form an embryo without fertilization and, thus, they share some common features. Moreover, the mechanism of asexual reproduction in lower and higher plants appears to be controlled by overlapping sets of genes (Cordle et al., 2012). ARGONAUTE (AGO) proteins play important roles in RNA-mediated silencing during plant development, including reproduction (Olmedo-Monfil et al., 2014). In this study, we identified a fern protein homologous to ARGONAUTE10/PINHEAD/ZWILLE (AGO10; Table 3), which represses cell entry into sexual reproduction and contributes to the maintenance of the shoot apical meristem and the establishment of leaf polarity by repressing miR165/166 in *A. thaliana* (Liu et al., 2009). Similarly to AGO proteins, the *A. thaliana* SERRATE (SE) RNA effector protein, a homolog of which was also identified here (Table 3), acts as a regulator of meristem activity and leaf polarity via the miRNA pathway (Prigge and Wagner, 2001). These proteins could potentially play a role in the meristematic activity of the incipient apogamic embryo or unknown roles in the switch between sexual and asexual reproduction. We speculate that SE and some AGO family proteins may be involved in the regulation of apogamy in ferns.

Previous reports in grasses described proteins associated with apomixis and the regulation of ploidy regulation suggesting that these are inter-related phenomena (Albertini et al., 2004). In this study, we found fern homologs of some of the described proteins, such as the Ras-related proteins, 4 DNAJ domain-containing proteins, cytochrome P450, several LRR-proteins, and proteins involved in gene silencing.

Finally, an important group of proteins identified in this study play a role in cell wall modifications, including glucanases, glycosyltransferases, and pectinases, (Table 3). Consistent with the presence of proteins associated with pectin catabolism, it has previously been reported that pectins are present in lower concentrations in ferns than in higher plants (Silva et al., 2011). Recently, gene expression in enlarging aposporous initial cells and early aposporous embryo sacs was compared to that in surrounding cells during apomictic initiation in *Hiercium praealtum* (tall hawkweed) and, interestingly, pectinecterases and other cell wall-modifying enzymes were identified, consistent with a role of cell wall modifications in apomixis and apogamy (Li et al., 2011).

### Identification of proteins involved in phytohormone signaling

The sessile lifestyle of plants requires a continuous crosstalk between the plant and its immediate environment. Phytohormones are of prime importance in this dynamic interaction to regulate and integrate overall plant growth and development. In *D. affinis*, auxins and gibberellins play a stimulatory role during the induction and differentiation of apogamous embryos, but phytohormones are also important for the vegetative development of the gametophyte (Menéndez et al., 2006b, 2009). In this study, we found several proteins related to the action of the classical phytohormones auxin, cytokinin, ethylene, and abscisic acid, as well as brassinosteroids, jasmonic acid, and polyamines. The above mentioned proteins may potentially participate in key aspects of vegetative and reproductive gametophyte development in ferns.

### Identification of proteins involved in stress responses

A particularity of the gametophyte of ferns is its vulnerability to stress. Hence, many proteins identified in this study are related to responses to biotic or abiotic stimuli. Regarding abiotic stress, we identified several heat-shock proteins (70, 90, 105, and hsc70), the glutathione-S-transferase protein F10/EARLY RESPONSE TO DEHYDRATION13, homologs of the desiccation-related protein PCC13-62 from *Craterostigma plantagineum* (resurrection plant; Piatkowski et al., 1990), and many other proteins known to participate in ABA-mediated stress responses. We also found proteins involved in cellular responses to toxic substances, including PLANT CADMIUM RESISTANCE3 (Song et al., 2004). Regarding proteins involved in biotic stress responses, we identified homologs of Virginiamycin B lyase, which is involved in antibiotic resistance, Nectarin 1, which may interact with bacterial adhesins and may protect from microbial attack (Carter et al., 1999), and proteins related to the cytochrome P450 family that, in the fern species *Polypodium vulgare* (common polypody), are associated with ecdysteroids, which are also present in plants (phytoecdysteroids) and suggested to participate in the defense against non-adapted phytophagous invertebrates (Canals et al., 2005). It has been suggested that an increase in metabolic activity and stress responses together induce the developmental switch to apogamy (Cordlel et al., 2012). Accordingly, the *in vitro* conditions that induce apogamous sporophytes in angiosperms from pollen or embryo sacs, universally include a stress treatment (Shariatpanahi et al., 2006).

The combination of different “omics” approaches is a promising way to obtain a comprehensive picture of regulatory processes. By integrating reference transcriptome and proteome analyses, we greatly improved protein identification in a non-model species, providing an important basis to gain further insights into apogamy in *D. affinis* ssp. *affinis*. Studying the molecular mechanisms of asexual reproduction, i.e., the generation of clonal offspring, is an important topic aiming at the introduction of self-sustainable hybrids in agriculture. Hence, the introduction of apomixis has a tremendous potential for crop improvement, and extending our analyses to phylogenetic branches other than those of model species may help to unravel underlying processes common to a broad range of organisms.

## Author contributions

HF, MC, and UG. conceived the project; HF, JG, PC, VG, and GR. performed experiments and/or analyzed data; JG, HF, and AV wrote the manuscript and contribute figures and tables; UG revised the manuscript.

### Conflict of interest statement

The authors declare that the research was conducted in the absence of any commercial or financial relationships that could be construed as a potential conflict of interest.

## Supplementary material

Figure S1 **Boxplot comparison of sequence lengths for the different databases used in this study in addition to other species-specific standard databases used in proteomics**.

Figure S2 **Pairwise alignments of the proteins discussed in our study with their best swissprot blastp match**.

Figure S3 **Peptide-spectrum-match assignments for proteins identified with only one single peptide sequence**.

Table S1 **Oligonucleotide sequences for truSeq™RNA**.

Table S2 **List of proteins identified in the gametophyte of *Dryopteris affinis* ssp. *affinis* by transcriptome (SSTDB) and proteome (VPDB) databases including additional information such as total assigned spectra, ProteinProphet probability and sequence coverage**.

Table S3 **Heatmap overview for all entries in the complete SSTDB, showing which species provides the best homolog with which *e*-value category**.

## Abbreviations

AA: amino acid
1D: one dimensional
DB: data base
FDR: false discovery rate
Gln: glutamine
Glu: glutamic acid
ID: identity
Ile: isoleucine
LC: liquid chromatography
Leu: leucine
MS: Murashige and Skoog (culture medium)
MS/MS: tandem mass spectrometry
NGS: next generation sequencing
PSM: peptide spectrum match
RT: room temperature
SSTDB: species-specific transcriptome database
TPM: transcripts per million
VPDB: viridiplantae database.
