# Supplementary figures and images for: Proteogenomic Analysis Greatly Expands the Identification of Proteins Related to Reproduction in the Apogamous Fern Dryopteris affinis ssp. affinis
Source: Front Plant Sci. 2017 Mar 22;8:336. doi: 10.3389/fpls.2017.00336 (PMC5360702; doi:10.3389/fpls.2017.00336)

# Figure S1

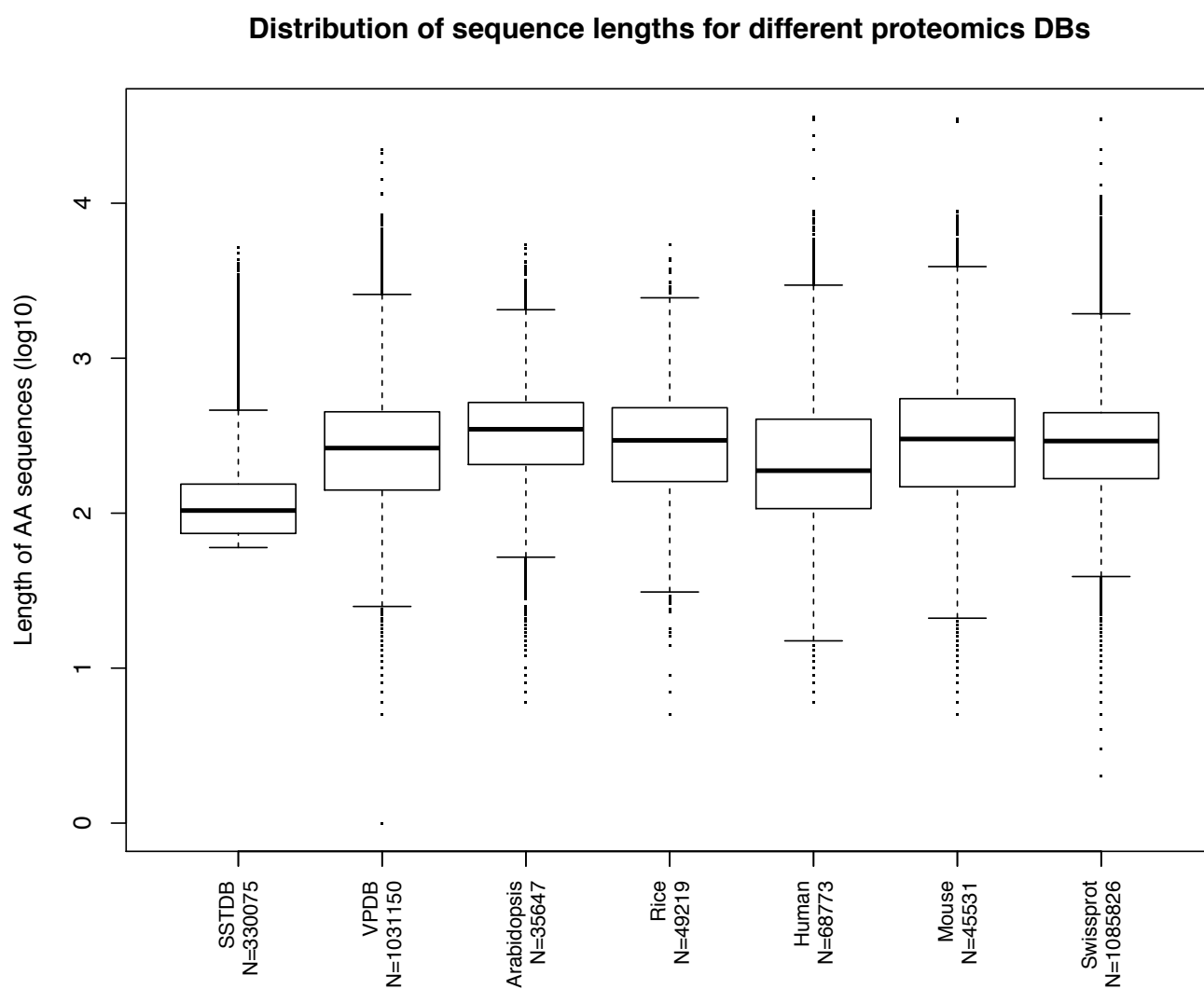

Supplement: Figure S1 — Boxplot comparison of sequence lengths for the different databases used in this study in addition to other species-specific standard databases used in proteomics. [file Image1.PDF]
