# Supplementary material for: Proteogenomic Analysis Greatly Expands the Identification of Proteins Related to Reproduction in the Apogamous Fern Dryopteris affinis ssp. affinis
Source: Front Plant Sci. 2017 Mar 22;8:336. doi: 10.3389/fpls.2017.00336 (PMC5360702; doi:10.3389/fpls.2017.00336)
Supplement: Figure S2 — Pairwise alignments of the proteins discussed in our study with their best swissprot blastp match. [file Image2.PDF]

//
//////

PAIRWISE ALIGNMENTS FOR PROTEINS DISCUSSED IN THE MANUSCRIPT

//
//////

blast version: BLASTP 2.2.29+

Query= sp|A1TLH9|
DNAK_ACIAC_Chaperone_protein_DnaK_OS=Acidovorax_citrulli_(
strain_AAC00-1)_GN=dnaK_PE=3_SV=1

Length=654

Subject= 73106-295_5_ORF2
>sp|A1TLH9|DNAK_ACIAC_Chaperone_protein_DnaK_OS=Acidovorax_citrulli_
(strain_AAC00-1)_GN=dnaK_PE=3_SV=1|||0

Length=697

Score = 804 bits (2077), Expect = 0.0, Method: Compositional
matrix adjust.
Identities = 390/600 (65%), Positives = 486/600 (81%), Gaps = 7/600
(1%)

```
Query  4
IIGIDLGTTNSCVAIMEGNTTRVIENSEGARTTPSIIAYQEDGEILVGASAKRQAVTNPK  63
            IIGIDLGTTNSCVA+MEG + RVIENSEGARTTPS++A+   GE L G
AKRQAVTNP
Sbjct  79
IIGIDLGTTNSCVAVMEGKSARVIENSEGARTTPSVVAFTSKGERLAGTPAKRQAVTNPL  138

Query  64
NTIYAAKRLIGRKFEEKEVQKDIDLMPYTIAKADNGDAWVEVRGNKLAPPQISAEVLRKM  123
            NT+Y  KRLIGR F++ +VQK++ ++ Y I +A NGDAWVE  G + +P QI A V
+ KM
Sbjct  139
NTVYGTKRLIGRNFDDAQVQKEMKMVSYKIVRAPNGDAWVEAGGQRYSPSQIGAFVVTKM  198

Query  124
KKTAEDYLGEPVTEAVITVPAYFNDAQRQATKDAGRIAGLDVKRIINEPTAAALAFGLDK  183
            K+TAE YLG PV++AVITVPAYFNDAQRQATKDAGRIAGL+V RIINEPTAA+L+
+G DK
Sbjct  199
```

```
KETAEAYLGRPVSKAVITVPAYFNDAQRQATKDAGRIAGLEVSRIINEPTAASLSYGADK  258

Query  184
QEKGDRKIAVYDLGGGTFDVSIIEIADVDGEKQFEVLSTNGDTFLGGEDFDQRIIDYIIA  243
            +E     +AV+DLGGGTFD+SI+EI+       FEV +TNGDTFLGGEDFD  ++
Y++
Sbjct  259  KEG---IVAVFDLGGGTFDISILEISS----
GVFEVKATNGDTFLGGEDFDNALLQYLVQ  311

Query  244
EFKKEQGVDLSKDVLALQRLKEAAEKAKIELSSSAATDINLPYITADASGPKHLNIKLTR  303
            EFKK+QG+DLS D +A+QRL+EAAEKAK+ELSSS  TDINLP+ITADASG KHLNI
LTR
Sbjct  312
EFKKDQGLDLSSDRMAIQRLREAAEKAKVELSSSTQTDINLPFITADASGAKHLNITLTR  371

Query  304
AKLESLVEELIERTIAPCRTAIKDAGISVSDIHDVILVGGMTRMPKVQEKVKEFFGKEPR  363
            +K ESLV +LIERT  PC+  +KDAGIS  D+H+V+LVGGMTRMPKVQE V +
FGK+P
Sbjct  372
SKFESLVHDLIERTKQPCKDCLKDAGISAKDVHEVLLVGGMTRMPKVQEIVSQIFGKDPS  431

Query  364
KDVNPDEAVAVGAAIQGQVLSGDRKDVLLLDVTPLSLGIETLGGVMTKMITKNTTIPTKF  423
            K VNPDE VA+GAAIQG VL GD KD+LLLDVTPLSLGIETLGGV T++I+
+NTTIPTK
Sbjct  432
KGVNPDECVAMGAAIQGGVLRGDVKDLLLLDVTPLSLGIETLGGVFTRLISRNTTIPTKK  491

Query  424
AQTFSTADDNQPAVTIKVFQGEREIASANKLLGEFNLEGIPPAGRGVPQIEVTFDIDANG  483
            +Q FSTA D+Q  V IKV QGERE+AS NKLLG+F L GIPPA
RGVPQIEVTFDIDANG
Sbjct  492
SQVFSTAADSQTQVGIKVLQGEREMASDNKLLGQFELVGIPPAPRGVPQIEVTFDIDANG  551

Query  484
ILHVSAKDKGTGKENKITIKANSGLSEDEIQKMVKDAELNAADDKKKLELVQARNQGEAA  543
            I++VSAKDK T KE +ITI+++ GLSE +I++MVKDAEL +  DK++  ++ A+N
+G++
Sbjct  552
IVNVSAKDKATAKEQQITIQSSGGLSETDIERMVKDAELYSQKDKERKGVIDAKNEGDSV  611

Query  544
VHSVTKSLSEHGDKLEAGEKETIEAAVKDLEAALKGEDKAAIEEKTNALMAASQKLGEKM  603
            ++S  KSL+E+ DKL +   E+I++A+KDL + L+ E+   I+EK  A+  A+ K
+GE +
Sbjct  612
IYSTEKSLNEYKDKLSSDVVESIQSALKDLRSVLESENAELIKEKITAVQTAAMKIGEAL  671
```

Score = 19.2 bits (38), Expect = 0.66, Method: Compositional matrix adjust.

```
 Identities = 14/36 (39%), Positives = 21/36 (58%), Gaps = 5/36
(14%)

Query  583  AAIEEKTNALMAASQ---KLGEKMYADAQAAAAAAG  615
            AA+EE+T +  AA Q    L  +  A A+A ++ AG
Sbjct  42   AAVEEQTTS--AARQFGASLSRRWLASARAFSSKAG  75



Lambda      K        H        a         alpha
   0.311    0.130    0.348    0.792     4.96

Gapped
Lambda      K        H        a         alpha    sigma
   0.267   0.0410    0.140     1.90     42.6     43.6

Effective search space used: 404670




Matrix: BLOSUM62
Gap Penalties: Existence: 11, Extension: 1
Neighboring words threshold: 11
Window for multiple hits: 40





Query= sp|A2XG55|
LEA1_ORYSI_Late_embryogenesis_abundant_protein_1_OS=Oryza_
sativa_subsp._indica_GN=LEA1_PE=1_SV=2

Length=333

Subject= 253046-98_1_ORF1
>sp|A2XG55|LEA1_ORYSI_Late_embryogenesis_abundant_protein_1_OS=Oryza
_sativa_subsp._indica_GN=LEA1_PE=1_SV=2|||1e-11

Length=134


 Score = 40.0 bits (92),  Expect = 4e-09, Method: Compositional
matrix adjust.
 Identities = 45/115 (39%), Positives = 51/115 (44%), Gaps = 11/115
(10%)

Query  116  KDYTADKARETNDSVARKTNETADATRDKLGEY-------
KDYTADKTQETKDAVAQKAS  168
            KD TA KA ET      KT E  DAT  K GE        KD TA K +ETK
QKA
Sbjct  16
KDTTAHKAEETKQYGQEKTGEAKDATAHKAGEAQHQAQQAKDTTAHKAEETKQYGQQKAG  75
```

```
Query  169  DASEATKNKLGEYKDALARKTRDAKDTTAQKATEFKDGVKATAQETRDATKDTTQ
223
            +A    +        A   K   A   T +KAT+ KDGV    Q+  DA K   Q
Sbjct  76   EAQHQAQG----ITGAAKEKAEGAAHATQEKATQAKDGVGHAFQQAGDAIKGAAQ
126


 Score = 34.7 bits (78),  Expect = 3e-07, Method: Compositional
matrix adjust.
 Identities = 26/59 (44%), Positives = 30/59 (51%), Gaps = 7/59
(12%)

Query  153  ADKTQETKDAVAQKASDASEATKNKLGEYKDALARK-------
TRDAKDTTAQKATEFK  204
            AD  Q  KD  A KA +  +  + K GE KDA A K        + AKDTTA KA
E K
Sbjct  9
ADHAQHAKDTTAHKAEETKQYGQEKTGEAKDATAHKAGEAQHQAQQAKDTTAHKAEETK  67



Lambda      K        H        a         alpha
   0.307    0.120    0.319    0.792     4.96

Gapped
Lambda      K        H        a         alpha    sigma
   0.267   0.0410    0.140     1.90     42.6     43.6

Effective search space used: 35256




Matrix: BLOSUM62
Gap Penalties: Existence: 11, Extension: 1
Neighboring words threshold: 11
Window for multiple hits: 40






Query= sp|A4FF33|
VGB_SACEN_Virginiamycin_B_lyase_OS=Saccharopolyspora_eryth
raea_(strain_NRRL_23338)_GN=vgb_PE=3_SV=1

Length=300

Subject= 307117-64_1_ORF1
>sp|A4FF33|VGB_SACEN_Virginiamycin_B_lyase_OS=Saccharopolyspora_eryt
hraea_(strain_NRRL_23338)_GN=vgb_PE=3_SV=1|||2e-11

Length=480
```

```
 Score = 51.2 bits (121),  Expect = 1e-11, Method: Compositional
matrix adjust.
 Identities = 63/253 (25%), Positives = 94/253 (37%), Gaps = 27/253
(11%)

Query  13   EVPDPAGGPYGITAGPDGALWFTLVHSGLIARLA---
PGGEATTHRLDADSGPAII-TAG  68
             +P P  GP+ I       LW +   S  + R+    P            S P  I
T
Sbjct  175
HLPQPMFGPHCIYEVGQTDLWTSCKDSRHVVRVNHTDPDAVDGISFYPCSSRPIFIATHP  234

Query  69   ADGALWFTEHRAHRIGRLTTEDGLTEF--
APPTPQAGPYGLATGADGALWFT-----EAS  121
              G ++ +     +I RL    GLT     PP     P GL  G DG +WFT
Sbjct  235
KSGDVYASLDLGSKIWRLEQSTGLTSELDIPPHRGTTPVGLIAGPDGNIWFTLLGGPSGG  294

Query  122  AGRIGRITAEGEIAEFGLPVP-
GAFPSMIAAGPDDAMWFTANQANAIGRMSFDGTAVLHE  180
             G   RI+A GE   F L  P GA   +I         F      +  R+    ++
++ E
Sbjct  295
TGTFARISATGEFTWFSLTTPLGANAGLIHLAFYHHSLFRDRGRGSGLRLWLLSSSMVAE  354

Query  181
LPTEAAAPVGLALGPDGALWFTEIGAGQIGRVTADGAISEFPLPDRTSRPHAIVARGDEL  240
            + T   A V   +  D            +GR+  +  I+   LP +  R H ++
G  L
Sbjct  355  MGTSIDAIVTAVIDDD------------VGRIITEHTIA---
LPSQPCRAHRVLPHGTGL  399

Query  241  WFTEWGANRVGRI  253
            + TE G + +  +
Sbjct  400  FVTELGISSLAHV  412


 Score = 23.5 bits (49),  Expect = 0.008, Method: Compositional
matrix adjust.
 Identities = 18/53 (34%), Positives = 26/53 (49%), Gaps = 4/53 (8%)

Query  239  ELWFTEWGANRVGRID--LDGRIDVHELPTPNSEPHGIA-VGQDGALWVALEN
288
             +W T    + + RID  L+  + V  LP P   PH I  VGQ   LW + ++
Sbjct  149  HVWATLQFRSLLIRIDVALNEIVQVIHLPQPMFGPHCIYEVGQTD-LWTSCKD
200


 Score = 22.7 bits (47),  Expect = 0.014, Method: Compositional
matrix adjust.
 Identities = 14/59 (24%), Positives = 23/59 (39%), Gaps = 8/59
(14%)
```

```
Query  231
HAIVARGDELWFTEWGANRVGRIDLDGRIDVHELPTPNSEPHGIAVGQDGALWVALENG  289
            +A +  G ++W  E        +D+         P   + P G+  G DG +W  L
G
Sbjct  240  YASLDLGSKIWRLEQSTGLTSELDIP--------
PHRGTTPVGLIAGPDGNIWFTLLGG  290


 Score = 16.9 bits (32),  Expect = 1.0, Method: Compositional matrix
adjust.
 Identities = 5/7 (71%), Positives = 6/7 (86%), Gaps = 0/7 (0%)

Query  94  EFAPPTP  100
           +F PPTP
Sbjct  62  QFLPPTP  68


 Score = 13.9 bits (24),  Expect = 7.9, Method: Compositional matrix
adjust.
 Identities = 5/9 (56%), Positives = 6/9 (67%), Gaps = 0/9 (0%)

Query  138  GLPVPGAFP  146
            GLP+  A P
Sbjct  39   GLPLISALP  47



Lambda      K        H        a         alpha
   0.317    0.137    0.431    0.792     4.96

Gapped
Lambda      K        H        a         alpha    sigma
   0.267   0.0410    0.140     1.90     42.6     43.6

Effective search space used: 121500




Query= sp|A4FF33|
VGB_SACEN_Virginiamycin_B_lyase_OS=Saccharopolyspora_eryth
raea_(strain_NRRL_23338)_GN=vgb_PE=3_SV=1

Length=300

Subject= 73514-294_4_ORF1
>sp|A4FF33|VGB_SACEN_Virginiamycin_B_lyase_OS=Saccharopolyspora_eryt
hraea_(strain_NRRL_23338)_GN=vgb_PE=3_SV=1|||2e-11

Length=474


 Score = 51.6 bits (122),  Expect = 1e-11, Method: Compositional
```

```
matrix adjust.
 Identities = 63/253 (25%), Positives = 94/253 (37%), Gaps = 27/253
(11%)

Query  13   EVPDPAGGPYGITAGPDGALWFTLVHSGLIARLA---
PGGEATTHRLDADSGPAII-TAG  68
             +P P  GP+ I       LW +   S  + R+    P            S P  I
T
Sbjct  169
HLPQPMFGPHCIYEVGQTDLWTSCKDSRHVVRVNHTDPDAVDGISFYPCSSRPIFIATHP  228

Query  69   ADGALWFTEHRAHRIGRLTTEDGLTEF--
APPTPQAGPYGLATGADGALWFT-----EAS  121
              G ++ +     +I RL    GLT     PP     P GL  G DG +WFT
Sbjct  229
KSGDVYASLDLGSKIWRLEQSTGLTSELDIPPHRGTTPVGLIAGPDGNIWFTLLGGPSGG  288

Query  122  AGRIGRITAEGEIAEFGLPVP-
GAFPSMIAAGPDDAMWFTANQANAIGRMSFDGTAVLHE  180
             G   RI+A GE   F L  P GA   +I         F      +  R+    ++
++ E
Sbjct  289
TGTFARISATGEFTWFSLTTPLGANAGLIHLAFYHHSLFRDRGRGSGLRLWLLSSSMVAE  348

Query  181
LPTEAAAPVGLALGPDGALWFTEIGAGQIGRVTADGAISEFPLPDRTSRPHAIVARGDEL  240
            + T   A V   +  D            +GR+  +  I+   LP +  R H ++
G  L
Sbjct  349  MGTSIDAIVTAVIDDD------------VGRIITEHTIA---
LPSQPCRAHRVLPHGTGL  393

Query  241  WFTEWGANRVGRI  253
            + TE G + +  +
Sbjct  394  FVTELGISSLAHV  406


 Score = 23.5 bits (49),  Expect = 0.009, Method: Compositional
matrix adjust.
 Identities = 18/53 (34%), Positives = 26/53 (49%), Gaps = 4/53 (8%)

Query  239  ELWFTEWGANRVGRID--LDGRIDVHELPTPNSEPHGIA-VGQDGALWVALEN
288
             +W T    + + RID  L+  + V  LP P   PH I  VGQ   LW + ++
Sbjct  143  HVWATLQFRSLLIRIDVALNEIVQVIHLPQPMFGPHCIYEVGQTD-LWTSCKD
194


 Score = 22.7 bits (47),  Expect = 0.016, Method: Compositional
matrix adjust.
 Identities = 14/59 (24%), Positives = 23/59 (39%), Gaps = 8/59
(14%)

Query  231
HAIVARGDELWFTEWGANRVGRIDLDGRIDVHELPTPNSEPHGIAVGQDGALWVALENG  289
```

```
            +A +  G ++W  E        +D+         P   + P G+  G DG +W  L
G
Sbjct  234  YASLDLGSKIWRLEQSTGLTSELDIP--------
PHRGTTPVGLIAGPDGNIWFTLLGG  284


 Score = 15.8 bits (29),  Expect = 2.4, Method: Compositional matrix
adjust.
 Identities = 6/19 (32%), Positives = 9/19 (47%), Gaps = 0/19 (0%)

Query  215  DGAISEFPLPDRTSRPHAI  233
            DGA+   P P +   P  +
Sbjct  53   DGALPIRPTPPKVEVPKVV  71


 Score = 14.6 bits (26),  Expect = 4.8, Method: Compositional matrix
adjust.
 Identities = 8/20 (40%), Positives = 10/20 (50%), Gaps = 0/20 (0%)

Query  138  GLPVPGAFPSMIAAGPDDAM  157
            GLP+    PS   A  D A+
Sbjct  37   GLPLISTLPSPPPAQYDGAL  56



Lambda      K        H        a         alpha
   0.317    0.137    0.431    0.792     4.96

Gapped
Lambda      K        H        a         alpha    sigma
   0.267   0.0410    0.140     1.90     42.6     43.6

Effective search space used: 121500




Query= sp|A4FF33|
VGB_SACEN_Virginiamycin_B_lyase_OS=Saccharopolyspora_eryth
raea_(strain_NRRL_23338)_GN=vgb_PE=3_SV=1

Length=300

Subject= 73817-294_4_ORF1
>sp|A4FF33|VGB_SACEN_Virginiamycin_B_lyase_OS=Saccharopolyspora_eryt
hraea_(strain_NRRL_23338)_GN=vgb_PE=3_SV=1|||2e-11

Length=481


 Score = 51.2 bits (121),  Expect = 1e-11, Method: Compositional
matrix adjust.
 Identities = 63/253 (25%), Positives = 94/253 (37%), Gaps = 27/253
(11%)
```

```
Query  13   EVPDPAGGPYGITAGPDGALWFTLVHSGLIARLA---
PGGEATTHRLDADSGPAII-TAG  68
             +P P  GP+ I       LW +   S  + R+    P            S P  I
T
Sbjct  176
HLPQPMFGPHCIYEVGQTDLWTSCKDSRHVVRVNHTDPDAVDGISFYPCSSRPIFIATHP  235

Query  69   ADGALWFTEHRAHRIGRLTTEDGLTEF--
APPTPQAGPYGLATGADGALWFT-----EAS  121
              G ++ +     +I RL    GLT     PP     P GL  G DG +WFT
Sbjct  236
KSGDVYASLDLGSKIWRLEQSTGLTSELDIPPHRGTTPVGLIAGPDGNIWFTLLGGPSGG  295

Query  122  AGRIGRITAEGEIAEFGLPVP-
GAFPSMIAAGPDDAMWFTANQANAIGRMSFDGTAVLHE  180
             G   RI+A GE   F L  P GA   +I         F      +  R+    ++
++ E
Sbjct  296
TGTFARISATGEFTWFSLTTPLGANAGLIHLAFYHHSLFRDRGRGSGLRLWLLSSSMVAE  355

Query  181
LPTEAAAPVGLALGPDGALWFTEIGAGQIGRVTADGAISEFPLPDRTSRPHAIVARGDEL  240
            + T   A V   +  D            +GR+  +  I+   LP +  R H ++
G  L
Sbjct  356  MGTSIDAIVTAVIDDD------------VGRIITEHTIA---
LPSQPCRAHRVLPHGTGL  400

Query  241  WFTEWGANRVGRI  253
            + TE G + +  +
Sbjct  401  FVTELGISSLAHV  413


 Score = 23.5 bits (49),  Expect = 0.008, Method: Compositional
matrix adjust.
 Identities = 18/53 (34%), Positives = 26/53 (49%), Gaps = 4/53 (8%)

Query  239  ELWFTEWGANRVGRID--LDGRIDVHELPTPNSEPHGIA-VGQDGALWVALEN
288
             +W T    + + RID  L+  + V  LP P   PH I  VGQ   LW + ++
Sbjct  150  HVWATLQFRSLLIRIDVALNEIVQVIHLPQPMFGPHCIYEVGQTD-LWTSCKD
201


 Score = 22.7 bits (47),  Expect = 0.016, Method: Compositional
matrix adjust.
 Identities = 14/59 (24%), Positives = 23/59 (39%), Gaps = 8/59
(14%)

Query  231
HAIVARGDELWFTEWGANRVGRIDLDGRIDVHELPTPNSEPHGIAVGQDGALWVALENG  289
            +A +  G ++W  E        +D+         P   + P G+  G DG +W  L
G
Sbjct  241  YASLDLGSKIWRLEQSTGLTSELDIP--------
```

```
PHRGTTPVGLIAGPDGNIWFTLLGG  291


 Score = 16.9 bits (32),  Expect = 0.94, Method: Compositional
matrix adjust.
 Identities = 5/7 (71%), Positives = 6/7 (86%), Gaps = 0/7 (0%)

Query  94  EFAPPTP  100
           +F PPTP
Sbjct  63  QFLPPTP  69


 Score = 14.2 bits (25),  Expect = 7.1, Method: Compositional matrix
adjust.
 Identities = 5/9 (56%), Positives = 6/9 (67%), Gaps = 0/9 (0%)

Query  138  GLPVPGAFP  146
            GLP+  A P
Sbjct  40   GLPLISALP  48



Lambda      K        H        a         alpha
   0.317    0.137    0.431    0.792     4.96

Gapped
Lambda      K        H        a         alpha    sigma
   0.267   0.0410    0.140     1.90     42.6     43.6

Effective search space used: 121500



Matrix: BLOSUM62
Gap Penalties: Existence: 11, Extension: 1
Neighboring words threshold: 11
Window for multiple hits: 40



Query= sp|B4KJW1|
IHOG_DROMO_Interference_hedgehog_OS=Drosophila_mojavensis_
GN=iHog_PE=3_SV=1

Length=897

Subject= 343536-46_3_ORF2
>sp|B4KJW1|IHOG_DROMO_Interference_hedgehog_OS=Drosophila_mojavensis
_GN=iHog_PE=3_SV=1|||4e-06

Length=133
```

```
 Score = 33.5 bits (75),  Expect = 2e-06, Method: Compositional
matrix adjust.
 Identities = 22/81 (27%), Positives = 41/81 (51%), Gaps = 9/81
(11%)

Query  330
LEHYIQLVVQQAPRIVRPPSANLTNEGEFMVLECAATGTPTPKIYWLLNGENSVYDTESE  389
            L+ Y+Q+VVQ   R+  P        GE+  +  AA     P  + +LN  +++
++++
Sbjct  43   LDFYMQIVVQNLTRLSSP--------GEYSAVRTAAPAVQQPFTFGVLNTFDNLL-
SKTQ  93

Query  390  LPANGSLILRRVQKRHAGCVQ  410
            L  N + ++ RVQ  +  C Q
Sbjct  94   LFNNSADLIGRVQGWYGDCGQ  114


 Score = 15.4 bits (28),  Expect = 2.1, Method: Compositional matrix
adjust.
 Identities = 13/48 (27%), Positives = 19/48 (40%), Gaps = 8/48
(17%)

Query  139  ARLELATISGDKIAQRSNWRVAAGNTVLWQCGQVVSNPAPTWSFYYND  186
            ++ +L   S D I +   W    G+     CGQ       T +F Y D
Sbjct  90   SKTQLFNNSADLIGRVQGWY---GD-----CGQDQLTLCLTQTFTYAD  129


 Score = 14.2 bits (25),  Expect = 5.0, Method: Compositional matrix
adjust.
 Identities = 5/6 (83%), Positives = 6/6 (100%), Gaps = 0/6 (0%)

Query  467  NVTRLS  472
            N+TRLS
Sbjct  53   NLTRLS  58



Lambda      K        H        a         alpha
   0.315    0.129    0.380    0.792     4.96

Gapped
Lambda      K        H        a         alpha    sigma
   0.267   0.0410    0.140     1.90     42.6     43.6

Effective search space used: 91245



Matrix: BLOSUM62
Gap Penalties: Existence: 11, Extension: 1
Neighboring words threshold: 11
```

```
Window for multiple hits: 40




Query= sp|B9L8K3|
Y545_NAUPA_UPF0763_protein_NAMH_0545_OS=Nautilia_profundic
ola_(strain_ATCC_BAA-1463_/_DSM_18972_/_AmH)_GN=NAMH_0545_PE=3_SV=1

Length=125

Subject= 138449-208_6_ORF2
>sp|B9L8K3|Y545_NAUPA_UPF0763_protein_NAMH_0545_OS=Nautilia_profundi
cola_(strain_ATCC_BAA-
1463_/_DSM_18972_/_AmH)_GN=NAMH_0545_PE=3_SV=1|||9e-07

Length=1116


 Score = 34.3 bits (77),  Expect = 1e-06, Method: Composition-based
stats.
 Identities = 20/66 (30%), Positives = 35/66 (53%), Gaps = 8/66
(12%)

Query  66   FELKLEKAILSEFPVDYDDVKAVVLEEMKRDENASISEILNKVKLEHP-------
NLFYN  118
            + L+L   ++S    +Y  +KA     M++DEN+ ++E + K  LE+P
NLF +
Sbjct  537  YPLQLPMDVVSNMSYEYTKIKAAA-
ALMEKDENSGVAEQIKKSLLEYPSIEKNVLNLFED  595

Query  119  LDLEKI  124
            L L  +
Sbjct  596  LSLRSL  601


 Score = 15.0 bits (27),  Expect = 3.0, Method: Composition-based
stats.
 Identities = 5/25 (20%), Positives = 14/25 (56%), Gaps = 0/25 (0%)

Query  34   WIMHINNEFKIVLDVEEYMKLMETM  58
            W   ++ ++   + ++EY  LM+ +
Sbjct  947  WRGGLHKQYDWEMILKEYYGLMQDL  971


 Score = 14.2 bits (25),  Expect = 5.8, Method: Composition-based
stats.
 Identities = 4/11 (36%), Positives = 7/11 (64%), Gaps = 0/11 (0%)

Query  32  SLWIMHINNEF  42
           ++ + H NN F
Sbjct  24  TMLLPHSNNRF  34
```

```
Lambda      K        H        a         alpha
   0.316    0.134    0.363    0.792     4.96

Gapped
Lambda      K        H        a         alpha    sigma
   0.267   0.0410    0.140     1.90     42.6     43.6

Effective search space used: 104352



Query= sp|B9L8K3|
Y545_NAUPA_UPF0763_protein_NAMH_0545_OS=Nautilia_profundic
ola_(strain_ATCC_BAA-1463_/_DSM_18972_/_AmH)_GN=NAMH_0545_PE=3_SV=1

Length=125

Subject= 28271-475_5_ORF2
>sp|B9L8K3|Y545_NAUPA_UPF0763_protein_NAMH_0545_OS=Nautilia_profundi
cola_(strain_ATCC_BAA-
1463_/_DSM_18972_/_AmH)_GN=NAMH_0545_PE=3_SV=1|||9e-07

Length=1116


 Score = 34.3 bits (77),  Expect = 1e-06, Method: Composition-based
stats.
 Identities = 20/66 (30%), Positives = 35/66 (53%), Gaps = 8/66
(12%)

Query  66   FELKLEKAILSEFPVDYDDVKAVVLEEMKRDENASISEILNKVKLEHP-------
NLFYN  118
            + L+L   ++S    +Y  +KA     M++DEN+ ++E + K  LE+P
NLF +
Sbjct  537  YPLQLPMDVVSNMSYEYTKIKAAA-
ALMEKDENSGVAEQIKKSLLEYPSIEKNVLNLFED  595

Query  119  LDLEKI  124
            L L  +
Sbjct  596  LSLRSL  601


 Score = 15.0 bits (27),  Expect = 3.1, Method: Composition-based
stats.
 Identities = 5/25 (20%), Positives = 14/25 (56%), Gaps = 0/25 (0%)

Query  34   WIMHINNEFKIVLDVEEYMKLMETM  58
            W   ++ ++   + ++EY  LM+ +
Sbjct  947  WRGGLHKQYDWEMILKEYYGLMQDL  971
```

```
 Score = 13.9 bits (24),  Expect = 7.5, Method: Composition-based
stats.
 Identities = 4/6 (67%), Positives = 4/6 (67%), Gaps = 0/6 (0%)

Query  37  HINNEF  42
           H NN F
Sbjct  29  HSNNHF  34



Lambda      K        H        a         alpha
   0.316    0.134    0.363    0.792     4.96

Gapped
Lambda      K        H        a         alpha    sigma
   0.267   0.0410    0.140     1.90     42.6     43.6

Effective search space used: 104352




Matrix: BLOSUM62
Gap Penalties: Existence: 11, Extension: 1
Neighboring words threshold: 11
Window for multiple hits: 40





Query= sp|C0LGG3|Y5182_ARATH_Probable_LRR_receptor-like_serine/
threonine-
protein_kinase_At1g51820_OS=Arabidopsis_thaliana_GN=At1g51820_PE=2_S
V=1

Length=885

Subject= 260337-93_5_ORF1
>sp|C0LGG3|Y5182_ARATH_Probable_LRR_receptor-like_serine/threonine-
protein_kinase_At1g51820_OS=Arabidopsis_thaliana_GN=At1g51820_PE=2_S
V=1|||4e-124

Length=718


 Score =   376 bits (965),  Expect = 4e-121, Method: Compositional
matrix adjust.
 Identities = 244/693 (35%), Positives = 375/693 (54%), Gaps =
67/693 (10%)

Query  235  LPQSVMAKAATPIKANDTLNITWTVEPPTTQFYSYVHIAEIQ-
ALRANETREFNVTLNGE  293
            LP  ++  A T    N  + + ++   P  ++ + ++ AE   A+ A+  R FN+
```

```
+NGE
Sbjct  22   LPARILQTAYT----
NTFITVNFSGLNPGLKYVANLYFAEFDSAVNASGQRAFNILVNGE  77

Query  294  Y--TFGP---
FSPIPLKTASIVDLSPGQCDGGRCILQVVKTLKSTLPPLLNAIEAFTVID  348
                T GP   + +    A    +      G     +  T  S   P + A E F
+
Sbjct  78
LKTTSGPVDVYDAVGANAAYSYGVVATPNTAGIMTFNLTPTSTSIFQPYVAAAEFFSTQF  137

Query  349
FPQMETNENDVAGIKNVQGTYGLSRISWQGDPCVPKQLLWDGLNCKNS------------  396
            F  + T+ N V+ + +++ +  L+  S++GDPC P    ++ LNC
Sbjct  138  FTPL-TDANTVSAVDDIKTSLNLN--
SYKGDPCFPIGFGYEWLNCSQDPNITAISLSNYA  194

Query  397  -------
DISTPPIITSLDLSSSGLTGIITQAIKNLTHLQILDLSDNNLTGEVPEFLADI  449
                  ++T  ++T + L  +GL G+I   +  LT+LQ L L++N L+G +P
+LA +
Sbjct  195  TGGEIPEALNTLTLLTQIHLDGNGLQGVIPD-
LSALTNLQALVLNNNQLSGPIPNYLATL  253

Query  450  KSLLVINLSGNNLSGSVPPSLLQKKG---
MKLNVEGNPHILCTTGSCVK-----------  495
            K+L V++L  N LSG +P +LLQ+K    +     GNP  LC + S V
Sbjct  254  KNLKVLDLQNNKLSGEIPAALLQRKQASTLDFEFSGNP--
LCDSNSGVACTPSTSPAPPN  311

Query  496  --KKEDGHKKKSVIVPVVASIASIAVLIG-
ALVLFLILRKKRSPKVEGPPPSYMQASDGR  552
                  K  S ++  VA    + V IG  + ++ I +KK    +E P   +  +
+
Sbjct  312
APSPTKSKKSSSGVIVGVAVAVVLLVGIGIGIAVYCICKKKTPSSLEQPINKHQASTQQE  371

Query  553  LPR--SSEPAIVTKN-------
RRFSYSQVVIMTNNFQRILGKGGFGMVYHGFVNGTEQV  603
            L +   S   +++K+       + FS+ ++ + TNNF   LG GGFG VY G +
V
Sbjct  372
LSQVYQSPGPVISKDVIPKLSVQEFSFQEIKVATNNFSTQLGIGGFGPVYKGRLQDGRFV  431

Query  604
AVKILSHSSSQGYKQFKAEVELLLRVHHKNLVGLVGYCDEGDNLALIYEYMANGDLKEHM  663
            A+K+ S+SS QG K+F  EV+LL RVHHKNLVGL+GYC+E + L L+YE+M+NG L
+ +
Sbjct  432  AIKVASNSSHQGSKEFLNEVDLLSRVHHKNLVGLLGYCNE-
EKLVLVYEFMSNGSLFDCL  490

Query  664  SGTRNRFI-
LNWGTRLKIVIESAQGLEYLHNGCKPPMVHRDVKTTNILLNEHFEAKLADF  722
             G   +   L+WGTRL I+++SAQG +YLH GC P ++HRDVK++NILL++  EAK
```

```
++DF
Sbjct  491
HGPYAKASPLSWGTRLGILVDSAQGFDYLHYGCSPRIIHRDVKSSNILLDDKLEAKISDF  550

Query  723
GLSRSFLIEGETHVSTVVAGTPGYLDPEYHRTNWLTEKSDVYSFGILLLEIITNRHVI--  780
            G+SR+ LI       T + G+ GY+DPEY  T  LTEK DVYSFG+LL E++  R
+
Sbjct  551
GISRNSLINETGAPPTALMGSMGYMDPEYLSTMKLTEKVDVYSFGVLLFEVVCGRTAVFQ  610

Query  781
DQSREKPHIGEWVGVMLTKGDIQSIMDPSLNEDYDSGSVWKAVELAMSCLNHSSARRPTM  840
            D S+++ HI EW    + +G I  I+D SL   YD  S+WK +E+A++C+N SSA
RP M
Sbjct  611
DSSQQQTHIAEWAKASIDRGVIDDIVDESLQRQYDISSIWKVLEIALACVNFSSAHRPKM  670

Query  841  SQVVIELNECLASENARGGASRDMESKSSIEVS  873
            S V +EL E  A         R++  K S+E S
Sbjct  671  SAVFLELKE--AERMELESDQRNVFDKPSVEFS  701


 Score = 20.0 bits (40),  Expect = 0.47, Method: Compositional
matrix adjust.
 Identities = 10/23 (43%), Positives = 13/23 (57%), Gaps = 2/23 (9%)

Query  105  TFVYGNYDGLNVGPNF--NLYLG  125
            TF+  N+ GLN G  +  NLY
Sbjct  34   TFITVNFSGLNPGLKYVANLYFA  56



Lambda      K        H        a         alpha
   0.317    0.135    0.396    0.792     4.96

Gapped
Lambda      K        H        a         alpha    sigma
   0.267   0.0410    0.140     1.90     42.6     43.6

Effective search space used: 571388




Query= sp|C0LGG3|Y5182_ARATH_Probable_LRR_receptor-like_serine/
threonine-
protein_kinase_At1g51820_OS=Arabidopsis_thaliana_GN=At1g51820_PE=2_S
V=1

Length=885

Subject= 260587-93_5_ORF1
>sp|C0LGG3|Y5182_ARATH_Probable_LRR_receptor-like_serine/threonine-
```

```
protein_kinase_At1g51820_OS=Arabidopsis_thaliana_GN=At1g51820_PE=2_S
V=1|||2e-112

Length=489


 Score =   335 bits (858),  Expect = 1e-108, Method: Compositional
matrix adjust.
 Identities = 196/477 (41%), Positives = 285/477 (60%), Gaps =
34/477 (7%)

Query  426  THLQILDLSDNNLTGEVPEFLADIKSLLVINLSGNNLSGSVPPSLLQKKG---
MKLNVEG  482
            T+LQ+L LS+N L+G +P +LA +K+L V++L  N LSG +P +LLQ+K    +
G
Sbjct  1
TNLQVLVLSNNELSGPIPNYLATLKNLKVLDLQNNKLSGEIPAALLQRKQASTLDFEFSG  60

Query  483  NPHILCTTGSCVK-------------KKEDGHKKKSVIVPVVASIASIAVLIG-
ALVLFL  528
            NP  LC + S V                    K  S ++  VA    + V IG  +
++
Sbjct  61   NP--
LCDSNSGVACTPSTSPAPPNAPSPTKSKKSSSGVIVGVAVAVVLLVGIGIGIAVYC  118

Query  529  ILRKKRSPKVEGPPPSYMQASDGRLPR--SSEPAIVTKN-------
RRFSYSQVVIMTNN  579
            I +KK    +E P   +  ++   L +   S   +++K+       + FS+ ++ +
TNN
Sbjct  119
ICKKKTPSSLEQPINKHQASTQQELSQVYQSPGPVISKDVIPKLSVQEFSFQEIKVATNN  178

Query  580
FQRILGKGGFGMVYHGFVNGTEQVAVKILSHSSSQGYKQFKAEVELLLRVHHKNLVGLVG  639
            F   LG GGFG VY G +     VA+K+ S+SS QG K+F  EV+LL
RVHHKNLVGL+G
Sbjct  179
FSTQLGIGGFGPVYKGRLQDGRFVAIKVASNSSHQGSKEFLNEVDLLSRVHHKNLVGLLG  238

Query  640  YCDEGDNLALIYEYMANGDLKEHMSGTRNRFI-
LNWGTRLKIVIESAQGLEYLHNGCKPP  698
            YC+E + L L+YE+M+NG L + + G   +   L+WGTRL I+++SAQG +YLH
GC P
Sbjct  239  YCNE-
EKLVLVYEFMSNGSLFDCLHGPYAKASPLSWGTRLGILVDSAQGFDYLHYGCSPR  297

Query  699
MVHRDVKTTNILLNEHFEAKLADFGLSRSFLIEGETHVSTVVAGTPGYLDPEYHRTNWLT  758
            ++HRDVK++NILL++  EAK++DFG+SR+ LI       T + G+ GY+DPEY  T
LT
Sbjct  298
IIHRDVKSSNILLDDKLEAKISDFGISRNSLINETGAPPTALMGSMGYMDPEYLSTMKLT  357

Query  759  EKSDVYSFGILLLEIITNRHVI--
```

DQSREKPHIGEWVGVMLTKGDIQSIMDPSLNEDYDS 816
EK DVYSFG+LL E++ R + D S+++ HI EW + +G I I+D SL
YD
Sbjct 358
EKVDVYSFGVLLFEVVCGRTAVFQDSSQQQTHIAEWAKASIDRGVIDDIVDESLQRQYDI 417

Query 817
GSVWKAVELAMSCLNHSSARRPTMSQVVIELNECLASENARGGASRDMESKSSIEVS 873
S+WK +E+A++C+N SSA RP MS V +EL E A R++ K S+E
S
Sbjct 418 SSIWKVLEIALACVNFSSAHRPKMSAVFLELKE--
AERMELESDQRNVFDKPSVEFS 472

Lambda K H a alpha
0.317 0.135 0.396 0.792 4.96

Gapped
Lambda K H a alpha sigma
0.267 0.0410 0.140 1.90 42.6 43.6

Effective search space used: 571388

Matrix: BLOSUM62
Gap Penalties: Existence: 11, Extension: 1
Neighboring words threshold: 11
Window for multiple hits: 40

Query= sp|C0LGI2|Y1677_ARATH_Probable_LRR_receptor-like_serine/
threonine-
protein_kinase_At1g67720_OS=Arabidopsis_thaliana_GN=At1g67720_PE=2_S
V=1

Length=929

Subject= 259284-93_5_ORF1
>sp|C0LGI2|Y1677_ARATH_Probable_LRR_receptor-like_serine/threonine-
protein_kinase_At1g67720_OS=Arabidopsis_thaliana_GN=At1g67720_PE=2_S
V=1|||2e-149

Length=960

Score = 206 bits (523), Expect = 5e-59, Method: Compositional
matrix adjust.
Identities = 177/545 (32%), Positives = 263/545 (48%), Gaps =

```
50/545 (9%)

Query  1    MGLCLAQLAVTCLFLVPFVLSQVTEFVSIDCG--
CSSNYTDPRTGLGWVSDSEIIKQGKP  58
            MG  L  L+ TC+       SQV  F+SIDCG   ++ YTD   G+ WV DS +I
+G P
Sbjct  12   MGRWLLFLS-TCMLFWSLGRSQVPGFLSIDCGSDATTTYTD-
DIGIVWVGDSNLITEGTP  69

Query  59   VTLANTNWNSMQYRRRRDFPTDNKKYCYRLSTK---ERRRYIVRTTFLYG-----
GLGSE  110
              ++     S      R F     KYCY L++        ++VR +F  G
+
Sbjct  70   TAISGGT--
SRVLSTMRLFDGKQSKYCYSLTSSAIISGAFFMVRGSFWPGITPPYKPQNP  127

Query  111  EAYPKFQLYLDATKWATVTIQEVSRVYVE-ELIVRATSSYVDVCVCCAITG--
SPFMSTL  167
            +   +F+L +DA  W+ V I   +  +   ++ +RA  S +DVC   +     +PF
+S L
Sbjct  128
DGNFRFKLIVDADVWSDVEIPYGTTTWWSFDIYIRAKRSSIDVCFARSTPSGDAPFVSAL  187

Query  168  ELRPLNLSMYATDYEDN----
FFLKVAARVNFGAPNMDALRYPDDPYDRIWESDINKRPN  223
            ELRPL  ++ AT    N    F   V A     + +  + RYP D  DR+W S
PN
Sbjct  188
ELRPLPSTLTATILMLNSSRIFVPLVHADYGVLSTSASSTRYPLDALDRLWLSYTASPPN  247

Query  224  YLVGVAPGTTRINTSKTINT-
LTREYPPMKVMQTAVVGTQGLISYRLNLEDFPANARAYA  282
                        +T  +I+T L  +  P +++QTA   T   I+   +  +
A
Sbjct  248  ----------MTSTDSSISTALQEDMLPARILQTAYTNT--
FITVNFSGLNPGLKYVANL  295

Query  283  YFAEIEE-LGANETRKFK-LVQPYFPDYSNAV-
VNIAENANGSYTLYEPSYMNVTLDFVL  339
            YFAE +  + A+  R F  LV       S  V V  A  AN +Y+    +  N
++
Sbjct  296
YFAEFDSAVNASGQRAFNILVNGELKTTSGPVDVYDAVGANAAYSYGVVATPNTA--GIM  353

Query  340
TFSFGKTKDSTQGPLLNAIEISKYLPISVKTDRSDVSVLDAIRSMSPDSDWASEGGDPCI  399
            TF+   T  S   P + A E       +  TD + VS +D I++     +  S
GDPC
Sbjct  354  TFNLTPTSTSIFQPYVAAAEFFSTQFFTPLTDANTVSAVDDIKT---
SLNLNSYKGDPCF  410

Query  400  PVL--
WSWVNCSSTSPPRVTKIALSRKNLRGEIPPGINYMEALTELWLDDNELTGTLPDM  457
            P+   + W+NCS    P +T I+LS     GEIP  +N +  LT++ LD N L G
```

```
+PD+
Sbjct  411  PIGFGYEWLNCSQD--
PNITAISLSNYATGGEIPEALNTLTLLTQIHLDGNGLQGVIPDL  468

Query  458  SKLVNLKIMHLENNQLSGSLPPYLAHLPNLQELSIENNSFKGKIPSALLKGK----
VLFK  513
            S L NL+ + L NNQLSG +P YLA L NL+ L ++NN   G+IP+ALL+ K
+ F+
Sbjct  469
SALTNLQALVLNNNQLSGPIPNYLATLKNLKVLDLQNNKLSGEIPAALLQRKQASTLDFE  528

Query  514  YNNNP  518
            ++ NP
Sbjct  529  FSGNP  533



Lambda      K        H        a         alpha
   0.318    0.134    0.395    0.792     4.96

Gapped
Lambda      K        H        a         alpha    sigma
   0.267   0.0410    0.140     1.90     42.6     43.6

Effective search space used: 810660




Query= sp|C0LGI2|Y1677_ARATH_Probable_LRR_receptor-like_serine/
threonine-
protein_kinase_At1g67720_OS=Arabidopsis_thaliana_GN=At1g67720_PE=2_S
V=1

Length=929

Subject= 75423-291_3_ORF1
>sp|C0LGI2|Y1677_ARATH_Probable_LRR_receptor-like_serine/threonine-
protein_kinase_At1g67720_OS=Arabidopsis_thaliana_GN=At1g67720_PE=2_S
V=1|||7e-149

Length=984


 Score =   205 bits (522),  Expect = 8e-59, Method: Compositional
matrix adjust.
 Identities = 177/545 (32%), Positives = 263/545 (48%), Gaps =
50/545 (9%)

Query  1    MGLCLAQLAVTCLFLVPFVLSQVTEFVSIDCG--
CSSNYTDPRTGLGWVSDSEIIKQGKP  58
            MG  L  L+ TC+       SQV  F+SIDCG   ++ YTD   G+ WV DS +I
+G P
Sbjct  36   MGRWLLFLS-TCMLFWSLGRSQVPGFLSIDCGSDATTTYTD-
```

```
DIGIVWVGDSNLITEGTP  93

Query  59   VTLANTNWNSMQYRRRRDFPTDNKKYCYRLSTK---ERRRYIVRTTFLYG-----
GLGSE  110
              ++     S      R F     KYCY L++        ++VR +F  G
+
Sbjct  94   TAISGGT--
SRVLSTMRLFDGKQSKYCYSLTSSAIISGAFFMVRGSFWPGITPPYKPQNP  151

Query  111  EAYPKFQLYLDATKWATVTIQEVSRVYVE-ELIVRATSSYVDVCVCCAITG--
SPFMSTL  167
            +   +F+L +DA  W+ V I   +  +   ++ +RA  S +DVC   +     +PF
+S L
Sbjct  152  
DGNFRFKLIVDADVWSDVEIPYGTTTWWSFDIYIRAKRSSIDVCFARSTPSGDAPFVSAL  211

Query  168  ELRPLNLSMYATDYEDN----
FFLKVAARVNFGAPNMDALRYPDDPYDRIWESDINKRPN  223
            ELRPL  ++ AT    N    F   V A     + +  + RYP D  DR+W S
PN
Sbjct  212  
ELRPLPSTLTATILMLNSSRIFVPLVHADYGVLSTSASSTRYPLDALDRLWLSYTASPPN  271

Query  224  YLVGVAPGTTRINTSKTINT-
LTREYPPMKVMQTAVVGTQGLISYRLNLEDFPANARAYA  282
                        +T  +I+T L  +  P +++QTA   T   I+   +  +
A
Sbjct  272  ----------MTSTDSSISTALQEDMLPARILQTAYTNT--
FITVNFSGLNPGLKYVANL  319

Query  283  YFAEIEE-LGANETRKFK-LVQPYFPDYSNAV-
VNIAENANGSYTLYEPSYMNVTLDFVL  339
            YFAE +  + A+  R F  LV       S  V V  A  AN +Y+    +  N
++
Sbjct  320  
YFAEFDSAVNASGQRAFNILVNGELKTTSGPVDVYDAVGANAAYSYGVVATPNTA--GIM  377

Query  340  
TFSFGKTKDSTQGPLLNAIEISKYLPISVKTDRSDVSVLDAIRSMSPDSDWASEGGDPCI  399
            TF+   T  S   P + A E       +  TD + VS +D I++     +  S
GDPC
Sbjct  378  TFNLTPTSTSIFQPYVAAAEFFSTQFFTPLTDANTVSAVDDIKT---
SLNLNSYKGDPCF  434

Query  400  PVL--
WSWVNCSSTSPPRVTKIALSRKNLRGEIPPGINYMEALTELWLDDNELTGTLPDM  457
            P+   + W+NCS    P +T I+LS     GEIP  +N +  LT++ LD N L G
+PD+
Sbjct  435  PIGFGYEWLNCSQD--
PNITAISLSNYATGGEIPEALNTLTLLTQIHLDGNGLQGVIPDL  492

Query  458  SKLVNLKIMHLENNQLSGSLPPYLAHLPNLQELSIENNSFKGKIPSALLKGK----
VLFK  513
            S L NL+ + L NNQLSG +P YLA L NL+ L ++NN   G+IP+ALL+ K
```

```
+ F+
Sbjct  493
SALTNLQALVLNNNQLSGPIPNYLATLKNLKVLDLQNNKLSGEIPAALLQRKQASTLDFE  552

Query  514  YNNNP  518
            ++ NP
Sbjct  553  FSGNP  557



Lambda      K        H        a         alpha
   0.318    0.134    0.395    0.792     4.96

Gapped
Lambda      K        H        a         alpha    sigma
   0.267   0.0410    0.140     1.90     42.6     43.6

Effective search space used: 810660




Matrix: BLOSUM62
Gap Penalties: Existence: 11, Extension: 1
Neighboring words threshold: 11
Window for multiple hits: 40





Query= sp|C0LGQ5|GSO1_ARATH_LRR_receptor-like_serine/threonine-
protein_kinase_GSO1_OS=Arabidopsis_thaliana_GN=GSO1_PE=2_SV=1

Length=1249

Subject= 363021-38_2_ORF1
>sp|C0LGQ5|GSO1_ARATH_LRR_receptor-like_serine/threonine-
protein_kinase_GSO1_OS=Arabidopsis_thaliana_GN=GSO1_PE=2_SV=1|||2e-
99

Length=909


 Score =   334 bits (856),  Expect = 2e-101, Method: Compositional
matrix adjust.
 Identities = 294/919 (32%), Positives = 448/919 (49%), Gaps =
88/919 (10%)

Query  4
LVLLLLFILCFSGLGQPGIINNDLQTLLEVKKSLVTNPQEDDPLRQWNSDNINYCSWTGV  63
             VL+L  ++C  G   P  + +D   LL  K  +    +    L+ W++   +
CSW GV
Sbjct  9    FVLMLSVVICVVG-DVPECVPSDRDALLAFKAGVSDTKKA---LQTWSTSK-
```

```
SCCSWHGV  63

Query  64   TCDNTGLFRVIALNLTGLGLTGSISPWFGR-FDNLIHLD-------LSSNNLVG-
PIPTA  114
            +C+  G  RVI L++T  G+        G    NL  L+       L+SN
+PT
Sbjct  64   SCNALG--
RVIRLSVTAYGIVVQSPNIIGSTLSNLTELEVLVLQNLLNSNGFPTLELPTE  121

Query  115  LSNLTSLESLFLFSNQLTGEIPSQLGSLVNIRSLRIGDN-
ELVGDIPETLGNLVNLQMLA  173
            L +L  L +L L  N+  G IP +   +  +++L +  N E+   IP +L  L
+LQ
Sbjct  122
LKSLRKLVTLSLVDNRFGGPIPMEWSEITTLQNLNLSLNWEINSTIPASLCKLRSLQTFG  181

Query  174  LASCR-----
LTGPIPSQLGRLVRVQSLILQDNYLEGPIPAELGNCSDLTVFTAAENMLN  228
            L + +     LTG IPS  G L+++  + L +N L G IPAE GN  +L     +
NM++
Sbjct  182
LVNSKSIPGSLTGSIPSCFGSLIQLTDIELSNNQLTGSIPAEFGNLINLQALRLSNNMMS  241

Query  229
GTIPAELGRLENLEILNLANNSLTGEIPSQLGEMSQLQ---------------------  266
            GTIP+ LG  + L+ L+L  N+ TGE+P+ LG +  L+
Sbjct  242
GTIPSSLGNPKQLQFLHLQGNTFTGEVPASLGNLINLKEALLGASEDDSGNLVGRNKGLS  301

Query  267  -------------YLSLMANQLQGLIPKSLADLGN-
LQTLDLSANNLTGEIPEEFWNMSQ  312
                           L +    + G +P SL    N L+ + L  N  TG IP
+ +
Sbjct  302
GSFPVGIGGKAIRILRIRGTSITGPLPASLGLTSNKLRDITLDNNAFTGSIPVSLGALKK  361

Query  313
LLDLVLANNHLSGSLPKSICSNNTNLEQLVLSGTQLSGEIPVELSKCQSLKQLDLSNNSL  372
            L+ L LA N L G +P S+    + L  + LS  +LS  IP   S+  SL  LDLS
+N L
Sbjct  362  LITLNLAGNQLEGPIPSSLARPKSKLNSINLSQNRLSDSIPSSFSRL-
SLSTLDLSHNQL  420

Query  373  AGSIPEALFELVELTDLYLHNNTLEGTLSPSISNLTNLQWLVLYHNNL-
EGKLPKEISAL  431
             GSIP  L   +    L L NN L G    S+ NLT +     + N+L  G LP +
+++
Sbjct  421
TGSIPSDLGNALLFGTLNLDNNELTGAFPTSLWNLTKITLSNNHLNSLPSGHLPSDVNS-  479

Query  432
RKLEVLFLYENRFSGEIPQEIGNCTSLKMIDMFGNHFEGEIPPSIGRLKELNLLHLRQNE  491
              L+ LFL  N FSG IP  + +   +  +++  N F   IP        ++  +L
N
```

```
Sbjct  480  -
HLQQLFLDSNEFSGLIPPWVIDLDVIFFLNLANNKFVSPIPTINTDSLTVSYFNLSHNL  538

Query  492  LVGGLPASLGNCHQLNILDLADNQLSGSIPSSFGFLKG-
LEQLMLYNNSLQGNLPDSLIS  550
                G +P + GN   LN LDL+ NQ SG IP++F    G    L+L +N L G LPD
+ +
Sbjct  539
FTGSIPEAFGNIRGLNTLDLSYNQFSGRIPANFTSGSGFFNSLVLSHNLLSGPLPDQIFA  598

Query  551  LRNLTRINLSHNRLNGTIHPLCGSSSYL--SFDVTNNGFEDEIPLELGN-
SQNLDRLRLG  607
                     ++LS+N L+G I  +    +    S D++ N     IP +L   S  L++
++LG
Sbjct  599
SGFFNSLDLSYNNLSGPIPNIFPDVTPFLSSIDLSFNQLSGAIPPKLFTYSSYLEKIKLG  658

Query  608  KNQLTGKIPWTLGKIRE-----LSLLDMSSNALTGTIPLQLVL---
CKKLTHIDLNNNFL  659
             NQ TG IP  L +  +     L+ + ++ N  TGT+     +    +K T  D++
N +
Sbjct  659  HNQFTGAIP-
NLSQACQPAATGLNFVALNDNKFTGTLSNASWIQDCAEKFTVFDISGNAV  717

Query  660  SGPIPPWLGKLSQLGELKLSSNQFVESLPTELFNC-
TKLLVLSLDGNSLNGSIPQEIGNL  718
            +GPIP  L   +QL  L L+ N+F   LPT LF     L VL L  N L+GS+P +
NL
Sbjct  718  TGPIPN-
LSSWTQLRVLNLARNKFTGPLPTFLFTSLPSLQVLDLSRNILSGSLPFK--NL  774

Query  719  GAL-
NVLNLDKNQFSGSLPQAMGKLSKLYELRLSRNSLTGEIPVEIGQLQDLQSALDLSY  777
            G L +++N      +G+  + M K    YE  L        +P  +       +A
+DLS
Sbjct  775  GGLKSIINA-----TGAELKPM-KGGPYYEDGLVFGGADFVVPFTL--
TLSTTTAIDLSN  826

Query  778
NNFTGDIPSTIGTLSKLETLDLSHNQLTGEVPGSVGDMKSLGYLNVSFNNLGGKLKKQFS  837
            N  +G + + +G+L+ L   D+++N+L+G +P S+GD+K L  L+VS N   G++
Sbjct  827
NALSGPLSANVGSLTGLHVFDVANNKLSGSLPSSLGDIKGLEILDVSNNAFSGQIPASLQ  886

Query  838  RWPADSFLGNTGLCGSPLS  856
             +PA  F GN  LCG PLS
Sbjct  887  SFPASDFAGNAKLCGRPLS  905



Lambda      K        H        a         alpha
   0.316    0.135    0.392    0.792     4.96

Gapped
```

```
Lambda      K        H        a         alpha    sigma
   0.267   0.0410    0.140     1.90     42.6     43.6

Effective search space used: 1040256




Matrix: BLOSUM62
Gap Penalties: Existence: 11, Extension: 1
Neighboring words threshold: 11
Window for multiple hits: 40






Query= sp|F4JSZ5|BIG1_ARATH_Brefeldin_A-
inhibited_guanine_nucleotide-
exchange_protein_1_OS=Arabidopsis_thaliana_GN=BIG1_PE=2_SV=1

Length=1687

Subject= 106730-241_3_ORF2
>sp|F4JSZ5|BIG1_ARATH_Brefeldin_A-inhibited_guanine_nucleotide-
exchange_protein_1_OS=Arabidopsis_thaliana_GN=BIG1_PE=2_SV=1|||0

Length=1783


 Score =  1670 bits (4324),  Expect = 0.0, Method: Compositional
matrix adjust.
 Identities = 876/1767 (50%), Positives = 1195/1767 (68%), Gaps =
99/1767 (6%)

Query  11    TRCGRVIGPSLDKIIKNAAWRKHTFLVSACKSVLDKL---
EALSDSPDPS---------S  58
             +R GRV+ P+L+K+IKNAAWRKHT LV  CK+V+DKL   E+ S+  D S
S
Sbjct  19
SRLGRVLNPALEKVIKNAAWRKHTKLVQDCKAVIDKLSVPESASEGSDGSERPPMKIVES  78

Query  59    PLFG----
LTTSDADAVLQPLLLSLDTGYAKVIEPALDCSFKLFSLSLLRGEVCSSSP--  112
             PLF      +  DA  +LQPL+ + +TG  KV+EPALD   KL S   LRGEV +
++
Sbjct  79
PLFDGSRYCSADDAIFILQPLVSACETGSLKVVEPALDAVQKLISHGYLRGEVDAANAVD  138

Query  113
DSLLYKLIHAICKVCGIGEESIELAVLRVLLAAVRSPRILIRGDCLLHLVRTCYNVYLGG  172
             + +L +L+ A+CK   + +++IEL VL+ LL AV S  + I G+ LL
VRTCYNVYLG
Sbjct  139
```

```
NGILVQLMEAVCKCHDLADDAIELLVLKTLLTAVTSVPLRIHGETLLKAVRTCYNVYLGS  198

Query  173
FNGTNQICAKSVLAQIMLIVFTRSEANSMDASLKTVNVNDLLAITDKNVNEGNSVHICQG  232
                 NQ  AK+ L Q+++IVF R EA+S    ++ + V DL+  ++++V++ N
Q
Sbjct  199
KVIVNQTTAKASLTQMLVIVFRRMEADSSTVPVQPIVVADLMEPSERSVSDTNITQFVQS  258

Query  233   FINDVITAGEAA--PPP-------
DFALVQPPEEGASSTE--------------------  263
             FI+ V+   E    P P       D A      EGA  T
Sbjct  259
FISKVVQDIEVVLNPSPSLKSMGHDGAFDTVATEGADPTNYLESTDKDMLDAKYWEISMY  318

Query  264   ----DEGTG------------------
SKIREDGFLLFKNLCKLSMKFSSQENTDDQILV  301
                 +E  G                 +K+R D FL+F+ LCKLSMK S+QE TD
+ +
Sbjct  319
KSALEERKGELSEVDLDREGDMELQITNKLRRDAFLVFRALCKLSMKSSAQEGTD-PLAL  377

Query  302
RGKTLSLELLKVIIDNGGPIWLSDERFLNAIKQLLCLSLLKNSALSVMSIFQLQCAIFTT  361
             RGK ++LELLK++++N G I+   +RFL AIKQ LCLSLLKNSA + M+IFQL C
+IF +
Sbjct  378
RGKIIALELLKILLENAGEIFRKSDRFLGAIKQYLCLSLLKNSASATMNIFQLSCSIFMS  437

Query  362
LLRKYRSGMKSEVGIFFPMLVLRVLENVLQPSFVQKMTVLSLLENICHDPNLIIDIFVNF  421
             L+ ++R+G+K+E+G+FFPM+VLRVLENV Q +F QKM VL  LE +C D  +++D
+FVN+
Sbjct  438
LVSRFRAGLKAEIGVFFPMIVLRVLENVAQANFQQKMVVLRFLEKLCVDSQILVDVFVNY  497

Query  422
DCDVESPNIFERIVNGLLKTALGPPPGSSTILSPVQDITFRHESVKCLVSIIKAMGTWMD  481
             DCDV S NIFER+VNGLLKTA G PPG+ T LSP+QD T +  ++KCLV +++++
WM+
Sbjct  498
DCDVNSSNIFERMVNGLLKTAQGIPPGAVTSLSPLQDATLKLAAMKCLVGVLRSIKNWMN  557

Query  482   QQLSVGDSLLPKSLENEAPANNHSNSN-------
EEDGTTIDHDFHPDLNPESSDAATLE  534
             +QL + D+   K  E+    +   N+         E+ +    +  P+ N E++
AT E
Sbjct  558
KQLRITDAHTQKIFESSEETHETGNTGLAAVQEGVEETSAEGSETRPESNGETTAVATFE  617

Query  535
QRRAYKIERQKGVTLFNRKPSKGIEFLISSKKVGNSPDEVVSFLRNTTGLNATMIGDYLG  594
             QRRAYK+E Q+G++LFN+KP  GI FL+ + KVG SP+E+ +FL+NT+G +
MIGDYLG
```

```
Sbjct  618
QRRAYKLELQEGISLFNKKPRSGIAFLVKANKVGESPEEICAFLKNTSGFDKGMIGDYLG  677

Query  595
EREDFPMKVMHAYVDSFDFKEMNFGEAIRFFLRGFRLPGEAQKIDRIMEKFAERFCKCNP  654
             E E+  ++VMHAYVDSF+F+ M F EAIR  L GFRLPGEAQKIDRIMEKFAER
+CKCNP
Sbjct  678
ENEELSLRVMHAYVDSFNFQGMEFDEAIRTLLLGFRLPGEAQKIDRIMEKFAERYCKCNP  737

Query  655
NSFSSADTAYVLAYSVIMLNTDAHNIMVKEKMTKADFIRNNRGIDDGKDLPEEYLGALYD  714
              +FSSADTAYVLAYSVI+LNTDAHN  VK KM+KA+F RNNRGI+DGKD+PE++
+GALYD
Sbjct  738
EAFSSADTAYVLAYSVILLNTDAHNPQVKNKMSKAEFFRNNRGINDGKDIPEDFMGALYD  797

Query  715
QVVINEIKMSSDSSAPESRQSNGLNKLLGLDGILNLVYWTQTEEKAVGANGLLIKDIQEK  774
             ++V NEIK+  D    +++Q +  +K+L LD ILN+V   + E+KA+  +  +I+
+QE+
Sbjct  798
RIVKNEIKLKEDPMVTQNKQPSNASKILSLDAILNIVIRKRGEDKALETSDDVIRHMQEQ  857

Query  775
FRSKSGKSESAYHVVTDVAILRFMVEVSWGPMLAAFSVTLDQSDDRLAAVECLRGFRYAV  834
             F++K+ KSE+ Y+  +DV ILR MVEVSW PMLAAFSV LDQS+D +   +CL
GFRYAV
Sbjct  858
FKAKASKSETVYYAASDVEILRPMVEVSWAPMLAAFSVPLDQSEDEVVTFQCLEGFRYAV  917

Query  835
HVTAVMGMQTQRDAFVTSMAKFTNLHCAGDMKQKNVDAVKAIISIAIEDGNHLQDAWEHI  894
             H+TAVM M+TQRDAF+TS+AKFT+LH A D+KQKN+DA+KAII+IA EDGN
+LQDAWEHI
Sbjct  918
HITAVMCMKTQRDAFLTSLAKFTSLHSAADIKQKNIDAIKAIIAIAEEDGNYLQDAWEHI  977

Query  895   LTCLSRIEHLQLLGEGAPSDASYFASTETEEKKALG-------
FPNLKKKGALQNPVMMA  947
             LTC+SR EHL LLGEGAP DA++FA+ + E +K+          P L++KG  +
A
Sbjct  978
LTCVSRFEHLHLLGEGAPPDATFFAAPQNESEKSRQAALKTPVLPVLRRKGPGRLQYAAA  1037

Query  948
VVRGGSYDSSTIGPNMPGLVKQDQINNFIANLNLLDQIGSFQLNNVYAHSQRLKTEAIVA  1007
               R GSYDS+ +G +  G+V  +Q+NN + NLN+L+QIGSF++N ++  SQ L
+EAIV
Sbjct  1038
AARRGSYDSAGVGGHASGVVTTEQMNNLVTNLNMLEQIGSFEMNRIFTRSQNLNSEAIVD  1097

Query  1008
FVKALCKVSMSELQSPTDPRVFSLTKLVEIAHYNMNRIRLVWSRIWSILSDFFVSVGLSE  1067
```

FVKALCKVSM EL+SPTDPRVFSLTK+VEIAHYNMNRIRLVWS IW++LSDFF
+SVG SE
Sbjct 1098
FVKALCKVSMEELRSPTDPRVFSLTKIVEIAHYNMNRIRLVWSLIWNVLSDFFISVGCSE 1157

Query 1068
NLSVAIFVMDSLRQLSMKFLEREELANYNFQNEFLRPFVIVMQKSSSAEIRELIVRCISQ 1127
NLS+AIF MDSLRQL+MKFLEREELANYNFQNEF++PFVIVM+KSS+ EIRELI
+RC+SQ
Sbjct 1158
NLSIAIFAMDSLRQLAMKFLEREELANYNFQNEFMKPFVIVMRKSSAVEIRELIIRCVSQ 1217

Query 1128
MVLSRVSNVKSGWKSVFKVFTTAAADERKNIVLLAFETMEKIVREYFSYITETEATTFTD 1187
MVLSRV+NVKSGWK +F VFTTAA D+ K+IVLLAFET+EK+VREYF YITETE
TTFTD
Sbjct 1218
MVLSRVNNVKSGWKIMFMVFTTAATDDHKSIVLLAFETIEKVVREYFPYITETETTTFTD 1277

Query 1188
CVRCLITFTNSTFTSDVSLNAIAFLRFCALKLADGGLVWNEKGRSSSPSTPVTDDHSPS- 1246
CV CLI FTNS F DVSLNAIAFLRFCALKLA+G E G S D
S S
Sbjct 1278 CVNCLIAFTNSRFNQDVSLNAIAFLRFCALKLAEG-----
ELGASGRNKEKDKDKASKSD 1332

Query 1247
TQNFMDADENISYWVPLLTGLSKLTSDSRSAIRKSSLEVLFNILKDHGHIFSRTFWIGVF 1306
T F D D+++ +W PLL GLS+L+ D R IRKS+L++LF+ L+ HGH+FS
W VF
Sbjct 1333
TPTFTDKDDHLYFWFPLLVGLSELSFDPRPDIRKSALDILFDTLRFHGHLFSLGLWERVF 1392

Query 1307
SSVIYPIFNSVWGENDLLSKDEHSSFPSTFSSHPSEVSWDAETSAMAAQYLVDLFVSFFT 1366
SV++PIF+SV D + + + +W ET +A Q
+VDLFV F+
Sbjct 1393
DSVLFPIFDSVRRAMDPPLEKREAEGVDVEQTEVEVDAWLYETCTLALQLVVDLFVKFYG 1452

Query 1367
VIRSQLSSVVSLLAGLIRSPAQGPTVAGVGALLRLADELGDRFSENEWKEIFLAVNEAAS 1426
V+ + ++ LL G I+ Q GV A +RL G FS+ +W E+ A+
EAA
Sbjct 1453
VVNPLVGKILHLLTGFIKRSHQSLAAIGVAAFVRLISNAGSLFSDQKWLEVLSALKEAAL 1512

Query 1427 LTLSSFMKTLRTMDDIPDEDTLSDQDFSN--EDDIDE-DSLQT-
MSYVVARTKSHITVQL 1482
TL +K + ++ T+ + ID DSL+ + Y V+ KS
VQL
Sbjct 1513 ETLPDVIKVVECA-
ELQVARTMQQHGLRTGYTERIDSMDSLRNRLHYAVSDMKSRTAVQL 1571

```
Query  1483  
QVVQVVTDLYRIHQQSLLASHVTVILEILSSISSHAHQLNSDLILQKKVRRACSILELSE  1542
              +VQ V ++Y +H   L A H  ++L++L  I+ H+H++N D  L+ K+++  ++
+ ++
Sbjct  1572  
LLVQAVNEIYNMHGGKLTAGHTMLLLDMLHEIAVHSHKVNGDFQLRSKLQKLQAVTQFTD  1631

Query  1543  
PPMLHFENDTFQNYLDILQAIVTNNPGVSLELNVESQLMTVCMQILKMYLKCTLFQGDEL  1602
             PP+L  E++++Q YL +LQ +  + P ++ ++ VES+L+ +C ++L++YL
Sbjct  1632  
PPLLRLESESYQVYLTLLQRLPLDKPDLAKDVEVESRLVELCEEVLQVYLNTATSAPHPT  1691

Query  1603  EETR--QP--
KNWILPMGAASKEEAAARSPLVVAVLKALRELKRDSFKRYAPNFFPLLVE  1658
              + +   P    W++P+G++ + E A+R PLVV+ L+A+  LK  SF+++
FFPLL
Sbjct  1692  
PQIQVEHPLSNPWVIPLGSSRRRELASRGPLVVSTLQAISGLKDSSFEKHLKQFFPLLAS  1751

Query  1659  LVRSEHSSSQVPQVLSTVFHTCMGAMM  1685
             L+  EH S +V   LS +F + +G ++
Sbjct  1752  LISCEHGSGEVLVALSDMFSSWIGPIL  1778


 Score = 23.5 bits (49),  Expect = 0.24, Method: Compositional 
matrix adjust.
 Identities = 8/27 (30%), Positives = 15/27 (56%), Gaps = 0/27 (0%)

Query  932  PNLKKKGALQNPVMMAVVRGGSYDSST  958
            PNL + G + NP +  V++  ++   T
Sbjct  16   PNLSRLGRVLNPALEKVIKNAAWRKHT  42



Lambda      K        H        a         alpha
   0.319    0.133    0.383    0.792     4.96

Gapped
Lambda      K        H        a         alpha    sigma
   0.267   0.0410    0.140     1.90     42.6     43.6

Effective search space used: 2830185




Matrix: BLOSUM62
Gap Penalties: Existence: 11, Extension: 1
Neighboring words threshold: 11
Window for multiple hits: 40
```

```
Query= sp|O22765|
TRPA1_ARATH_Tryptophan_synthase_alpha_chain_OS=Arabidopsis
_thaliana_GN=TRPA1_PE=1_SV=2

Length=275

Subject= 194247-143_2_ORF2
>sp|O22765|TRPA1_ARATH_Tryptophan_synthase_alpha_chain_OS=Arabidopsi
s_thaliana_GN=TRPA1_PE=1_SV=2|||4e-129

Length=320


 Score =   361 bits (927),  Expect = 4e-129, Method: Compositional
matrix adjust.
 Identities = 169/261 (65%), Positives = 216/261 (83%), Gaps = 0/261
(0%)

Query  13
LSETFARLKSQGKVALIPYITAGDPDLSTTAKALKVLDSCGSDIIELGVPYSDPLADGPA  72
            ++ TF+ LK+ GKVALIPYITAGDP LSTTA+AL VLD+ G
+DIIELGVPYSDPLADGP
Sbjct  59
VANTFSDLKTAGKVALIPYITAGDPSLSTTAEALVVLDNSGADIIELGVPYSDPLADGPV  118

Query  73
IQAAARRSLLKGTNFNSIISMLKEVIPQLSCPIALFTYYNPILRRGVENYMTVIKNAGVH  132
            IQAAA R+L  GTN +++++ML+EV+P+L+ P+ LFTYYNPIL+RG+E ++  IK
GV
Sbjct  119
IQAAATRALQNGTNMDAVLNMLREVVPRLTAPLILFTYYNPILKRGIEVFLRAIKEVGVT  178

Query  133
GLLVPDVPLEETETLRNEARKHQIELVLLTTPTTPKERMNAIVEASEGFIYLVSSVGVTG  192
            GL++PD+PLEET+TLR+    + +ELVLLTTPTTPKERM+ I +A++GF+YLVS
GVTG
Sbjct  179
GLVIPDLPLEETDTLRSLTASNGLELVLLTTPTTPKERMDEISQATQGFVYLVSLTGVTG  238

Query  193
TRESVNEKVQSLLQQIKEATSKPVAVGFGISKPEHVKQVAEWGADGVIVGSAMVKILGES  252
             R  V  +V+SLL+++K+ T KP+AVGFGISKPEH  QVAEWGADGVIVGSAMVK
+LGE+
Sbjct  239
ARAKVETRVESLLKELKKVTDKPIAVGFGISKPEHAVQVAEWGADGVIVGSAMVKLLGEA  298

Query  253  ESPEQGLKELEFFTKSLKSAL  273
             SPE+G+  L   T+ L+ AL
Sbjct  299  SSPEEGICALRNLTQDLRKAL  319
```

```
Lambda      K        H        a         alpha
   0.314    0.132    0.361    0.792     4.96

Gapped
Lambda      K        H        a         alpha    sigma
   0.267   0.0410    0.140     1.90     42.6     43.6

Effective search space used: 73206



Matrix: BLOSUM62
Gap Penalties: Existence: 11, Extension: 1
Neighboring words threshold: 11
Window for multiple hits: 40



Query= sp|O48786|
C734A_ARATH_Cytochrome_P450_734A1_OS=Arabidopsis_thaliana_
GN=CYP734A1_PE=2_SV=1

Length=520

Subject= 314455-60_3_ORF2
>sp|O48786|C734A_ARATH_Cytochrome_P450_734A1_OS=Arabidopsis_thaliana
_GN=CYP734A1_PE=2_SV=1|||2e-146

Length=530


 Score =   399 bits (1024),  Expect = 9e-137, Method: Compositional
matrix adjust.
 Identities = 204/477 (43%), Positives = 307/477 (64%), Gaps =
19/477 (4%)

Query  31   LWWRPRKIEEHFSKQGIRGPPYHFFIGNVKELVGMMLKASSHPMP-
FSHNILPRVLSFYH  89
            ++W P +I+  F  QGI+  P+HFF GN+ EL  +   A  HPMP  SH+I PR+L
+ Y+
Sbjct  59
MYWTPLRIKRAFEAQGIKTLPFHFFHGNLPELNAIDKAARCHPMPQVSHDIAPRILAHYY  118

Query  90   HWRKIYGATFLVWFGPTFRLTVADPDLIREI-
FSKSEFYEKNEAHPLVKQLEGDGLLSLK  148
            HW K YG T++ WFG   R T+ +P+  ++I F+K   + K    P  K L G+G+
+ L+
Sbjct  119
HWAKKYGVTYVYWFGSQARYTLINPEDAKDILFTKFGHFLKPYRRPDAKALLGNGIVFLE  178

Query  149
```

```
GEKWAHHRKIISPTFHMENLKLLVPVVLKSVTDMVDKWSDKLSENGEVEVDVYEWFQILT  208
            GEKWA HR+I++P F ++ LK ++P +      M++ W   L E G+  +D+ +
+ LT
Sbjct  179  GEKWAQHRRILNPAFFLDKLKAMIPSMEACTIAMMENWGS-LVETGQA-
IDMQKNLKDLT  236

Query  209
EDVISRTAFGSSYEDGRAVFRLQAQQMLLCAEAFQKVFIPGYRFFPTRGNLKSWKLDKEI  268
             D+I+ TAFGSSY +G+ VF LQ +Q +L  +     +IPG RFFPT  N  SWK
+D++I
Sbjct  237
SDIIAHTAFGSSYAEGKQVFELQCEQKVLMDKLLSAAYIPGARFFPTAINRYSWKIDRDI  296

Query  269  RKSLLKLIERRRQNAIDGEGEECKEPAAKDLLGLMIQA---------KNVT--
VQDIVEE  317
            ++ L ++I  R ++   G G+        DLLGLM+ A         KN+T  +
DI++E
Sbjct  297  KRCLRQVINNREESIKVGRGDS----
YGNDLLGLMMAANKHMLQGNQKNLTMNLNDIIDE  352

Query  318
CKSFFFAGKQTTSNLLTWTTILLSMHPEWQAKARDEVLRVCGSRDVPTKDHVVKLKTLSM  377
            CK+FFFAG +TT+ LLTWT +LL+++PEWQ + R+EV+ VCG+ D PT D +  LK
+ M
Sbjct  353
CKTFFFAGHETTATLLTWTFLLLAINPEWQTRTREEVVAVCGTTDTPTADSISHLKIMGM  412

Query  378
ILNESLRLYPPIVATIRRAKSDVKLGGYKIPCGTELLIPIIAVHHDQAIWGNDVNEFNPA  437
            +LNE LRLYPP++  +R A  D++LG   IP GT + +P++  HHD+  WG D
NEF P
Sbjct  413
VLNEVLRLYPPVLRIMRTAGRDMQLGKVVIPKGTVVTVPLVLWHHDERYWGVDANEFRPE  472

Query  438
RFADGVPRAAKHPVGFIPFGLGVRTCIGQNLAILQAKLTLAVMIQRFTFHLAPTYQH  494
            RF++G  RA+     F+PF +G R CIGQ+  +L+AK+ L  +++++ F L+PTY
+H
Sbjct  473
RFSEGAARASIVAGAFLPFSMGPRVCIGQSFTLLEAKVVLCTILRQYRFCLSPTYKH  529



Lambda      K        H        a         alpha
   0.323    0.139    0.428    0.792     4.96

Gapped
Lambda      K        H        a         alpha    sigma
   0.267   0.0410    0.140     1.90     42.6     43.6

Effective search space used: 240075
```

Query= sp|O48786|
C734A_ARATH_Cytochrome_P450_734A1_OS=Arabidopsis_thaliana_
GN=CYP734A1_PE=2_SV=1

Length=520

Subject= 314966-60_3_ORF2
>sp|O48786|C734A_ARATH_Cytochrome_P450_734A1_OS=Arabidopsis_thaliana
_GN=CYP734A1_PE=2_SV=1|||8e-155

Length=550

Score =   421 bits (1083), Expect = 2e-145, Method: Compositional
matrix adjust.
Identities = 215/498 (43%), Positives = 320/498 (64%), Gaps =
19/498 (4%)

```
Query  31   LWWRPRKIEEHFSKQGIRGPPYHFFIGNVKELVGMMLKASSHPMP-
FSHNILPRVLSFYH  89
            ++W P +I+  F  QGI+  P+HFF GN+ EL  +   A  HPMP  SH+I PR+L
+ Y+
Sbjct  59
MYWTPLRIKRAFEAQGIKTLPFHFFHGNLPELNAIDKAARCHPMPQVSHDIAPRILAHYY  118

Query  90   HWRKIYGATFLVWFGPTFRLTVADPDLIREI-
FSKSEFYEKNEAHPLVKQLEGDGLLSLK  148
            HW K YG T++ WFG   R T+ +P+  ++I F+K   + K    P  K L G+G+
+ L+
Sbjct  119
HWAKKYGVTYVYWFGSQARYTLINPEDAKDILFTKFGHFLKPYRRPDAKALLGNGIVFLE  178

Query  149
GEKWAHHRKIISPTFHMENLKLLVPVVLKSVTDMVDKWSDKLSENGEVEVDVYEWFQILT  208
            GEKWA HR+I++P F ++ LK ++P +      M++ W   L E G+  +D+ +
+ LT
Sbjct  179  GEKWAQHRRILNPAFFLDKLKAMIPSMEACTIAMMENWGS-LVETGQA-
IDMQKNLKDLT  236

Query  209
EDVISRTAFGSSYEDGRAVFRLQAQQMLLCAEAFQKVFIPGYRFFPTRGNLKSWKLDKEI  268
             D+I+ TAFGSSY +G+ VF LQ +Q +L  +     +IPG RFFPT  N  SWK
+D++I
Sbjct  237
SDIIAHTAFGSSYAEGKQVFELQCEQKVLMDKLLSAAYIPGARFFPTAINRYSWKIDRDI  296

Query  269  RKSLLKLIERRRQNAIDGEGEECKEPAAKDLLGLMIQA---------KNVT--
VQDIVEE  317
            ++ L ++I  R ++   G G+        DLLGLM+ A         KN+T  +
DI++E
Sbjct  297  KRCLRQVINNREESIKVGRGDS----
YGNDLLGLMMAANKHMLQGNQKNLTMNLNDIIDE  352
```

```
Query  318
CKSFFFAGKQTTSNLLTWTTILLSMHPEWQAKARDEVLRVCGSRDVPTKDHVVKLKTLSM  377
            CK+FFFAG +TT+ LLTWT +LL+++PEWQ + R+EV+ VCG+ D PT D +  LK
+ M
Sbjct  353
CKTFFFAGHETTATLLTWTFLLLAINPEWQTRTREEVVAVCGTTDTPTADSISHLKIMGM  412

Query  378
ILNESLRLYPPIVATIRRAKSDVKLGGYKIPCGTELLIPIIAVHHDQAIWGNDVNEFNPA  437
            +LNE LRLYPP++  +R A  D++LG   IP GT + +P++  HHD+  WG D
NEF P
Sbjct  413
VLNEVLRLYPPVLRIMRTAGRDMQLGKVVIPKGTVVTVPLVLWHHDERYWGVDANEFRPE  472

Query  438
RFADGVPRAAKHPVGFIPFGLGVRTCIGQNLAILQAKLTLAVMIQRFTFHLAPTYQHAPT  497
            RF++G  RA+     F+PF +G R CIGQ+  +L+AK+ L  +++++ F L+PTY
HAPT
Sbjct  473
RFSEGAARASIVAGAFLPFSMGPRVCIGQSFTLLEAKVVLCTILRQYRFCLSPTYTHAPT  532

Query  498  VLMLLYPQHGAPITFRRL  515
             ++ L PQ G PI F +L
Sbjct  533  TVLTLQPQFGVPILFEKL  550



Lambda      K        H        a         alpha
   0.323    0.139    0.428    0.792     4.96

Gapped
Lambda      K        H        a         alpha    sigma
   0.267   0.0410    0.140     1.90     42.6     43.6

Effective search space used: 240075




Query= sp|O48786|
C734A_ARATH_Cytochrome_P450_734A1_OS=Arabidopsis_thaliana_
GN=CYP734A1_PE=2_SV=1

Length=520

Subject= 390160-26_3_ORF2
>sp|O48786|C734A_ARATH_Cytochrome_P450_734A1_OS=Arabidopsis_thaliana
_GN=CYP734A1_PE=2_SV=1|||1e-154

Length=562


 Score =   422 bits (1085),  Expect = 2e-145, Method: Compositional
matrix adjust.
```

```
 Identities = 216/498 (43%), Positives = 320/498 (64%), Gaps =
19/498 (4%)

Query  31   LWWRPRKIEEHFSKQGIRGPPYHFFIGNVKELVGMMLKASSHPMP-
FSHNILPRVLSFYH  89
            + WRP +I+  F  QGI+  P+HFF GN+ EL  +   A  HPMP  SH+I PR+L
+ Y+
Sbjct  71
MCWRPLRIKRAFEAQGIKTLPFHFFHGNLPELNAIDKAARCHPMPQVSHDIAPRILAHYY  130

Query  90   HWRKIYGATFLVWFGPTFRLTVADPDLIREI-
FSKSEFYEKNEAHPLVKQLEGDGLLSLK  148
            HW K YG T++ WFG   R T+ +P+  ++I F+K   + K    P  K L G+G+
+ L+
Sbjct  131
HWAKKYGVTYVYWFGSQARYTLINPEDAKDILFTKFGHFLKPYRRPDAKALLGNGIVFLE  190

Query  149
GEKWAHHRKIISPTFHMENLKLLVPVVLKSVTDMVDKWSDKLSENGEVEVDVYEWFQILT  208
            GEKWA HR+I++P F ++ LK ++P +      M++ W   L E G+  +D+ +
+ LT
Sbjct  191  GEKWAQHRRILNPAFFLDKLKAMIPSMEACTIAMMENWGS-LVETGQA-
IDMQKNLKDLT  248

Query  209
EDVISRTAFGSSYEDGRAVFRLQAQQMLLCAEAFQKVFIPGYRFFPTRGNLKSWKLDKEI  268
             D+I+ TAFGSSY +G+ VF LQ +Q +L  +     +IPG RFFPT  N  SWK
+D++I
Sbjct  249
SDIIAHTAFGSSYAEGKQVFELQCEQKVLMDKLLSAAYIPGARFFPTAINRYSWKIDRDI  308

Query  269  RKSLLKLIERRRQNAIDGEGEECKEPAAKDLLGLMIQA---------KNVT--
VQDIVEE  317
            ++ L ++I  R ++   G G+        DLLGLM+ A         KN+T  +
DI++E
Sbjct  309  KRCLRQVINNREESIKVGRGDS----
YGNDLLGLMMAANKHMLQGNQKNLTMNLNDIIDE  364

Query  318
CKSFFFAGKQTTSNLLTWTTILLSMHPEWQAKARDEVLRVCGSRDVPTKDHVVKLKTLSM  377
            CK+FFFAG +TT+ LLTWT +LL+++PEWQ + R+EV+ VCG+ D PT D +  LK
+ M
Sbjct  365
CKTFFFAGHETTATLLTWTFLLLAINPEWQTRTREEVVAVCGTTDTPTADSISHLKIMGM  424

Query  378
ILNESLRLYPPIVATIRRAKSDVKLGGYKIPCGTELLIPIIAVHHDQAIWGNDVNEFNPA  437
            +LNE LRLYPP++  +R A  D++LG   IP GT + +P++  HHD+  WG D
NEF P
Sbjct  425
VLNEVLRLYPPVLRIMRTAGRDMQLGKVVIPKGTVVTVPLVLWHHDERYWGVDANEFRPE  484

Query  438
RFADGVPRAAKHPVGFIPFGLGVRTCIGQNLAILQAKLTLAVMIQRFTFHLAPTYQHAPT  497
```

```
            RF++G  RA+     F+PF +G R CIGQ+  +L+AK+ L  +++++ F L+PTY
HAPT
Sbjct  485
RFSEGAARASIVAGAFLPFSMGPRVCIGQSFTLLEAKVVLCTILRQYRFCLSPTYTHAPT  544

Query  498  VLMLLYPQHGAPITFRRL  515
             ++ L PQ G PI F +L
Sbjct  545  TVLTLQPQFGVPILFEKL  562


 Score = 15.4 bits (28),  Expect = 6.9, Method: Compositional matrix
adjust.
 Identities = 5/9 (56%), Positives = 6/9 (67%), Gaps = 0/9 (0%)

Query  380  NESLRLYPP  388
            N  LR+ PP
Sbjct  6    NVELRIIPP  14



Lambda      K        H        a         alpha
   0.323    0.139    0.428    0.792     4.96

Gapped
Lambda      K        H        a         alpha    sigma
   0.267   0.0410    0.140     1.90     42.6     43.6

Effective search space used: 240075




Query= sp|O48786|
C734A_ARATH_Cytochrome_P450_734A1_OS=Arabidopsis_thaliana_
GN=CYP734A1_PE=2_SV=1

Length=520

Subject= 390778-26_3_ORF2
>sp|O48786|C734A_ARATH_Cytochrome_P450_734A1_OS=Arabidopsis_thaliana
_GN=CYP734A1_PE=2_SV=1|||3e-146

Length=542


 Score =   400 bits (1028),  Expect = 4e-137, Method: Compositional
matrix adjust.
 Identities = 205/477 (43%), Positives = 307/477 (64%), Gaps =
19/477 (4%)

Query  31   LWWRPRKIEEHFSKQGIRGPPYHFFIGNVKELVGMMLKASSHPMP-
FSHNILPRVLSFYH  89
            + WRP +I+  F  QGI+  P+HFF GN+ EL  +   A  HPMP  SH+I PR+L
+ Y+
```

```
Sbjct  71
MCWRPLRIKRAFEAQGIKTLPFHFFHGNLPELNAIDKAARCHPMPQVSHDIAPRILAHYY  130

Query  90   HWRKIYGATFLVWFGPTFRLTVADPDLIREI-
FSKSEFYEKNEAHPLVKQLEGDGLLSLK  148
            HW K YG T++ WFG   R T+ +P+  ++I F+K   + K    P  K L G+G+
+ L+
Sbjct  131
HWAKKYGVTYVYWFGSQARYTLINPEDAKDILFTKFGHFLKPYRRPDAKALLGNGIVFLE  190

Query  149
GEKWAHHRKIISPTFHMENLKLLVPVVLKSVTDMVDKWSDKLSENGEVEVDVYEWFQILT  208
            GEKWA HR+I++P F ++ LK ++P +      M++ W   L E G+  +D+ +
+ LT
Sbjct  191  GEKWAQHRRILNPAFFLDKLKAMIPSMEACTIAMMENWGS-LVETGQA-
IDMQKNLKDLT  248

Query  209
EDVISRTAFGSSYEDGRAVFRLQAQQMLLCAEAFQKVFIPGYRFFPTRGNLKSWKLDKEI  268
             D+I+ TAFGSSY +G+ VF LQ +Q +L  +     +IPG RFFPT  N  SWK
+D++I
Sbjct  249
SDIIAHTAFGSSYAEGKQVFELQCEQKVLMDKLLSAAYIPGARFFPTAINRYSWKIDRDI  308

Query  269  RKSLLKLIERRRQNAIDGEGEECKEPAAKDLLGLMIQA---------KNVT--
VQDIVEE  317
            ++ L ++I  R ++   G G+        DLLGLM+ A         KN+T  +
DI++E
Sbjct  309  KRCLRQVINNREESIKVGRGDS----
YGNDLLGLMMAANKHMLQGNQKNLTMNLNDIIDE  364

Query  318
CKSFFFAGKQTTSNLLTWTTILLSMHPEWQAKARDEVLRVCGSRDVPTKDHVVKLKTLSM  377
            CK+FFFAG +TT+ LLTWT +LL+++PEWQ + R+EV+ VCG+ D PT D +  LK
+ M
Sbjct  365
CKTFFFAGHETTATLLTWTFLLLAINPEWQTRTREEVVAVCGTTDTPTADSISHLKIMGM  424

Query  378
ILNESLRLYPPIVATIRRAKSDVKLGGYKIPCGTELLIPIIAVHHDQAIWGNDVNEFNPA  437
            +LNE LRLYPP++  +R A  D++LG   IP GT + +P++  HHD+  WG D
NEF P
Sbjct  425
VLNEVLRLYPPVLRIMRTAGRDMQLGKVVIPKGTVVTVPLVLWHHDERYWGVDANEFRPE  484

Query  438
RFADGVPRAAKHPVGFIPFGLGVRTCIGQNLAILQAKLTLAVMIQRFTFHLAPTYQH  494
            RF++G  RA+     F+PF +G R CIGQ+  +L+AK+ L  +++++ F L+PTY
+H
Sbjct  485
RFSEGAARASIVAGAFLPFSMGPRVCIGQSFTLLEAKVVLCTILRQYRFCLSPTYKH  541


 Score = 15.4 bits (28),  Expect = 6.6, Method: Compositional matrix
```

```
adjust.
 Identities = 5/9 (56%), Positives = 6/9 (67%), Gaps = 0/9 (0%)

Query  380  NESLRLYPP  388
            N  LR+ PP
Sbjct  6    NVELRIIPP  14



Lambda      K        H        a         alpha
   0.323    0.139    0.428    0.792     4.96

Gapped
Lambda      K        H        a         alpha    sigma
   0.267   0.0410    0.140     1.90     42.6     43.6

Effective search space used: 240075




Matrix: BLOSUM62
Gap Penalties: Existence: 11, Extension: 1
Neighboring words threshold: 11
Window for multiple hits: 40





Query= sp|O48928|
C77A3_SOYBN_Cytochrome_P450_77A3_OS=Glycine_max_GN=CYP77A3
_PE=2_SV=1

Length=513

Subject= 273313-84_6_ORF2
>sp|O48928|C77A3_SOYBN_Cytochrome_P450_77A3_OS=Glycine_max_GN=CYP77A
3_PE=2_SV=1|||1e-84

Length=423


 Score =   225 bits (574),  Expect = 1e-71, Method: Compositional
matrix adjust.
 Identities = 133/373 (36%), Positives = 215/373 (58%), Gaps =
20/373 (5%)

Query  48
GNLFQVARSGKPFFEYVNDVRLKYGSIFTLKMGTRTMIILTDAKLVHEAMIQKGATYATR  107
            GNLF + +        ++ +R K G IFTL  G   +I +T + L HEA+++KG
+A R
Sbjct  67   GNLFHLPKLQAS----
LHGIRAKLGPIFTLYAGRTPLIFITSSALAHEALVEKGRIFAAR  122
```

```
Query  108
PPENPTRTIFSENKFTVNAATYGPVWKSLRRNMVQNMLSSTRLKEFRSVRDNAMDKLINR  167
            P   P+R +F+ N  ++N+A+YGP W+S+RRN+V +ML+   +  F+ VR + +D
LI+
Sbjct  123  PVL-
PSRIVFTNNYRSINSASYGPYWRSIRRNLVHDMLTMPNILSFKPVRLSTIDHLISH  181

Query  168
LKDEAEKNNGVVWVLKDARFAVFCILVAMCFGLEMDEETVERIDQVMKSVLITLDPRIDD  227
            ++ +A+ ++G + V    R A+F +++ MCFG  M E+   ++   M  +L
+
Sbjct  182
IRTKAQLSDGRILVYPIIRTAMFELILFMCFGFHMSEDDALQMCNHMDELLHYSAGTLQY  241

Query  228  YLPILSPFFSKQRKKALEVRREQVEFLVPIIEQRRRAIQ----
NPGSDHTATTFSYLDTL  283
            +   L     K R++ L ++ +QV+      E+     +     PGS        Y
++TL
Sbjct  242  FYDFLGFPTWKNRQRGLALQAKQVQLFSSHFEKHHELKKLGQIAPGS--------
YVETL  293

Query  284
FDLKVEGKKSAPSDAELVSLCSEFLNGGTDTTATAVEWGIAQLIANPNVQTKLYEEIKRT  343
              L ++G  S  S  ++  LC+EFL  GTDTTAT +EW +A+++ + ++Q+KLY E
+
Sbjct  294  --LHMDGSISL-
STVDMAILCTEFLAAGTDTTATTLEWAMARIVDDSSIQSKLYNEMYDV  350

Query  344
VGEKKVDEKDVEKMPYLHAVVKELLRKHPPTHFVLTHAVTEPTTLGGYDIPIDANVEVYT  403
            VG + V+EKD+  + YL AV+KE LR HPP  F+L H V+E   +GGYDIP +A V
Sbjct  351
VGNENVEEKDLPNLTYLQAVIKETLRLHPPGTFLLPHCVSEDCKIGGYDIPTNAVVMFNI  410

Query  404  PAIAEDPKNWLNP  416
              IA+DP+ W  P
Sbjct  411  TFIAKDPEIWEEP  423



Lambda      K        H        a         alpha
   0.320    0.136    0.404    0.792     4.96

Gapped
Lambda      K        H        a         alpha    sigma
   0.267   0.0410    0.140     1.90     42.6     43.6

Effective search space used: 187200



Matrix: BLOSUM62
```

```
Gap Penalties: Existence: 11, Extension: 1
Neighboring words threshold: 11
Window for multiple hits: 40





Query= sp|O48956|
C98A1_SORBI_Cytochrome_P450_98A1_OS=Sorghum_bicolor_GN=CYP
98A1_PE=2_SV=1

Length=512

Subject= 246414-102_1_ORF2
>sp|O48956|C98A1_SORBI_Cytochrome_P450_98A1_OS=Sorghum_bicolor_GN=CY
P98A1_PE=2_SV=1|||0

Length=553


 Score =   645 bits (1664),  Expect = 0.0, Method: Compositional
matrix adjust.
 Identities = 301/468 (64%), Positives = 371/468 (79%), Gaps = 2/468
(0%)

Query  41
LGNLRQIKPIRCRCFQEWAERYGPVISVWFGSGLTVVVSTSELAKEVLKENDQQLADRPR  100
            +GNL  I P+R RCF EW++ YGP++SVWFGS L VVVS+++LAKEVLK+ DQQLA
R R
Sbjct  87
VGNLHDIAPVRFRCFWEWSKIYGPIMSVWFGSTLNVVVSSADLAKEVLKDKDQQLAARNR  146

Query  101
NRSTQRFSRNGQDLIWADYGPHYIKVRKLCNLELFTPKRLEALRPIREDEVTAMVESVYR  160
             R+   FSRNGQDLIWADYGPHY+KVRK+C LELFTPKRLEALRP+REDEV
MVES+++
Sbjct  147
TRAANLFSRNGQDLIWADYGPHYVKVRKVCTLELFTPKRLEALRPLREDEVATMVESIFK  206

Query  161
AATAPGNEGKPMVVRNHLSMVAFNNITRLAFGKRFMNANGDIDEQGREFKTIVNNGIKIG  220
                 G     + ++++LS VAFNNITR+AFGKRF++ +G ID QG EFK +++ G
+K+G
Sbjct  207
DCGGKGGNTSAITLKSYLSAVAFNNITRIAFGKRFVDGDGKIDPQGIEFKEVISQGMKLG  266

Query  221  ASLSVAEFIWYLRWLCPLNEELYKTHNERRDRLTMKIIEEHAKSLKESG-
AKQHFVDALF  279
            AS+   EFI ++RW+ PL +E +  H  RRD LT  I++EH  + + SG
AKQHFVDAL
Sbjct  267
ASIKTPEFIPWIRWMFPLQKEEFLKHGARRDNLTKLIMQEHTIARETSGEAKQHFVDALL  326
```

```
Query  280
TLKQQYDLSEDTVIGLLWDMITAGMDTTVISVEWAMAELVRNPRVQKKLQEELDRVVGRD  339
            TL++Q+ L++  +IGLLWDMITAGMDTT ISVEWAMAELVRNPRVQ+K QEELDRV
+G +
Sbjct  327
TLQKQHGLTDTHIIGLLWDMITAGMDTTAISVEWAMAELVRNPRVQQKAQEELDRVIGME  386

Query  340
RVMLETDFQNLPYLQAVVKESLRLHPPTPLMLPHKASTNVKIGGYDIPKGANVMVNVWAV  399
             V  E DF  L YLQ VVKESLRLHPPTPLMLPHKA+ +VKIGGYD+PKG  V
VNVWA+
Sbjct  387
DVAKEVDFSKLHYLQCVVKESLRLHPPTPLMLPHKATEDVKIGGYDVPKGTVVHVNVWAI  446

Query  400
ARDPKVWSNPLEYRPERFLEENIDIKGSDFRVLPFGAGRRVCPGAQLGINLVASMIGHLL  459
            ARDP +W +PL +RPERFLEE++DIKG D+R+LPFGAGRRVCPGAQLG+NLV  M+
LL
Sbjct  447
ARDPAIWKDPLAFRPERFLEEDVDIKGHDYRLLPFGAGRRVCPGAQLGLNLVELMLARLL  506

Query  460  HHFEWSLPEGTRPEDVNMMESPGLVTFMGTPLQAVAKPRLEKEELYNR  507
            H F WS PEG + +D++M E PG+VTFM  PL+ VA+PRL    LY R
Sbjct  507  HQFTWSPPEGMQADDIDMTERPGVVTFMAKPLEVVAQPRLPA-HLYTR  553



Lambda      K        H        a         alpha
   0.321    0.137    0.416    0.792     4.96

Gapped
Lambda      K        H        a         alpha    sigma
   0.267   0.0410    0.140     1.90     42.6     43.6

Effective search space used: 247086




Matrix: BLOSUM62
Gap Penalties: Existence: 11, Extension: 1
Neighboring words threshold: 11
Window for multiple hits: 40





Query= sp|O49816|
LEA1_CICAR_Late_embryogenesis_abundant_protein_1_OS=Cicer_
arietinum_PE=2_SV=1

Length=177
```

```
Subject= 194085-143_1_ORF2
>sp|O49816|LEA1_CICAR_Late_embryogenesis_abundant_protein_1_OS=Cicer
_arietinum_PE=2_SV=1|||5e-09

Length=131


 Score = 41.2 bits (95),  Expect = 4e-10, Method: Compositional
matrix adjust.
 Identities = 43/110 (39%), Positives = 59/110 (54%), Gaps = 4/110
(4%)

Query  37
EKAQQAAQTAKDKTSQTAQAAKEKTQQTAQAAKEKTQQTAQAAKDETQQTAQAAKDKTQQ  96
            +KAQ  A+ AKD T+  AQ AK  T+Q A  A    Q+TAQ A +  +  AQ A +
Q
Sbjct  13   DKAQYKAEQAKDATAGKAQEAKGVTEQKAGEA----
QKTAQGAAETAKNKAQGATESGQG  68

Query  97   TTEATKEKAQDTTGRAREKGSEMGQSTKETAQSGKDNSAGFLQQTGEKVK  146
              E TK KA D T  A+EK +      +++A + KD + G L Q  + VK
Sbjct  69   VWEQTKTKAADATAVAQEKATGAAGYVRDSAIAAKDQAVGALGQAQQTVK  118


 Score = 16.9 bits (32),  Expect = 0.10, Method: Compositional
matrix adjust.
 Identities = 16/51 (31%), Positives = 28/51 (55%), Gaps = 0/51 (0%)

Query  86   TAQAAKDKTQQTTEATKEKAQDTTGRAREKGSEMGQSTKETAQSGKDNSAG  136
            TA  A+ K +Q  +AT  KAQ+  G   +K  E  ++ +  A++ K+ + G
Sbjct  11   TADKAQYKAEQAKDATAGKAQEAKGVTEQKAGEAQKTAQGAAETAKNKAQG  61



Lambda      K        H        a         alpha
   0.297    0.109    0.274    0.792     4.96

Gapped
Lambda      K        H        a         alpha    sigma
   0.267   0.0410    0.140     1.90     42.6     43.6

Effective search space used: 18240




Query= sp|O49816|
LEA1_CICAR_Late_embryogenesis_abundant_protein_1_OS=Cicer_
arietinum_PE=2_SV=1

Length=177

Subject= 249225-100_3_ORF2
>sp|O49816|LEA1_CICAR_Late_embryogenesis_abundant_protein_1_OS=Cicer
```

```
_arietinum_PE=2_SV=1|||5e-09

Length=102


 Score = 28.1 bits (61),  Expect = 1e-05, Method: Compositional
matrix adjust.
 Identities = 30/78 (38%), Positives = 38/78 (49%), Gaps = 0/78 (0%)

Query  76
AQAAKDETQQTAQAAKDKTQQTTEATKEKAQDTTGRAREKGSEMGQSTKETAQSGKDNSA  135
            AQ AKD T   A+  K   QQ     + +AQ  TG A+EK      +T+E A
KD
Sbjct  18
AQHAKDTTAHKAEETKQYGQQKAGEAQHQAQGITGAAKEKAEGAAHATQEKATQAKDGVG  77

Query  136  GFLQQTGEKVKGMAQGAT  153
               QQ G+ +KG AQ  T
Sbjct  78   HAFQQAGDAIKGAAQKVT  95


 Score = 13.9 bits (24),  Expect = 1.0, Method: Compositional matrix
adjust.
 Identities = 24/83 (29%), Positives = 34/83 (41%), Gaps = 11/83
(13%)

Query  43   AQTAKDKTSQTAQAAKEKTQQTAQAAKEKT-----------
QQTAQAAKDETQQTAQAAK  91
            AQ AKD T+  A+  K+  QQ A  A+ +            +  A A +++  Q
Sbjct  18
AQHAKDTTAHKAEETKQYGQQKAGEAQHQAQGITGAAKEKAEGAAHATQEKATQAKDGVG  77

Query  92   DKTQQTTEATKEKAQDTTGRARE  114
               QQ  +A K  AQ  TG   E
Sbjct  78   HAFQQAGDAIKGAAQKVTGGGGE  100


 Score = 11.9 bits (19),  Expect = 4.1, Method: Compositional matrix
adjust.
 Identities = 6/10 (60%), Positives = 7/10 (70%), Gaps = 0/10 (0%)

Query  33  QAAKEKAQQA  42
            A +EKA QA
Sbjct  63  HATQEKATQA  72



Lambda      K        H        a         alpha
   0.297    0.109    0.274    0.792     4.96

Gapped
Lambda      K        H        a         alpha    sigma
   0.267   0.0410    0.140     1.90     42.6     43.6
```

Effective search space used: 18240

Matrix: BLOSUM62
Gap Penalties: Existence: 11, Extension: 1
Neighboring words threshold: 11
Window for multiple hits: 40

Query= sp|P09189|
HSP7C_PETHY_Heat_shock_cognate_70_kDa_protein_OS=Petunia_h
ybrida_GN=HSP70_PE=2_SV=1

Length=651

Subject= 289383-74_4_ORF2
>sp|P09189|HSP7C_PETHY_Heat_shock_cognate_70_kDa_protein_OS=Petunia_
hybrida_GN=HSP70_PE=2_SV=1|||3e-157

Length=241

Score =   444 bits (1142), Expect = 3e-157, Method: Compositional
matrix adjust.
Identities = 213/225 (95%), Positives = 219/225 (97%), Gaps = 0/225
(0%)

Query 6
EGPAIGIDLGTTYSCVGVWQHDRVEIIANDQGNRTTPSYVGFTDTERLIGDAAKNQVAMN 65
EGPAIGIDLGTTYSCVGVWQHDRVEIIANDQGNRTTPSYV
FTDTERLIGDAAKNQVAMN
Sbjct 17
EGPAIGIDLGTTYSCVGVWQHDRVEIIANDQGNRTTPSYVAFTDTERLIGDAAKNQVAMN 76

Query 66
PINTVFDAKRLIGRRFSDPSVQSDIKLWPFKVIPGPGDKPMIVVTYKGEEKQFAAEEISS 125
PINTVFDAKRLIGRRFSDP+VQSD KLWPFKVIPGPGDKPMIVV YKGEE
+QFAAEEISS
Sbjct 77
PINTVFDAKRLIGRRFSDPTVQSDSKLWPFKVIPGPGDKPMIVVQYKGEERQFAAEEISS 136

Query 126
MVLTKMKEIAEAYLGTTIKNAVVTVPAYFNDSQRQATKDAGVIAGLNVMRIINEPTAAAI 185
MVL KMKEIAEA+LG+TIKNAVVTVPAYFNDSQRQATKDAGVI
+GLNVMRIINEPTAAAI
Sbjct 137
MVLVKMKEIAEAFLGSTIKNAVVTVPAYFNDSQRQATKDAGVISGLNVMRIINEPTAAAI 196

Query 186 AYGLDKKASSAGEKNVLIFDLGGGTFDVSLLTIEEGIFEVKATAG 230

```
            AYGLDKK +S GEKNVLIFDLGGGTFDVSLLTIEEGIFEVKATAG
Sbjct  197  AYGLDKKTTSQGEKNVLIFDLGGGTFDVSLLTIEEGIFEVKATAG  241
```

 Score = 15.0 bits (27), Expect = 3.7, Method: Compositional matrix adjust.
 Identities = 8/28 (29%), Positives = 15/28 (54%), Gaps = 6/28 (21%)

```
Query  509  ITNDKGR------LSKEEIERMVQEAEK  530
            I ND+G       ++  + ER++ +A K
Sbjct  43   IANDQGNRTTPSYVAFTDTERLIGDAAK  70
```

 Score = 14.2 bits (25), Expect = 7.4, Method: Compositional matrix adjust.
 Identities = 4/9 (44%), Positives = 6/9 (67%), Gaps = 0/9 (0%)

```
Query  611  PIIAKMYQG  619
            P+I   Y+G
Sbjct  116  PMIVVQYKG  124
```

 Score = 14.2 bits (25), Expect = 7.6, Method: Compositional matrix adjust.
 Identities = 5/13 (38%), Positives = 6/13 (46%), Gaps = 0/13 (0%)

```
Query  472  PAPRGVPQITVCF  484
            P P   P I V +
Sbjct  110  PGPGDKPMIVVQY  122
```

 Score = 13.9 bits (24), Expect = 8.5, Method: Compositional matrix adjust.
 Identities = 4/5 (80%), Positives = 5/5 (100%), Gaps = 0/5 (0%)

```
Query  374  AVAYG  378
            A+AYG
Sbjct  195  AIAYG  199
```

```
Lambda      K        H        a         alpha
   0.313    0.132    0.371    0.792     4.96

Gapped
Lambda      K        H        a         alpha    sigma
   0.267   0.0410    0.140     1.90     42.6     43.6
```

Effective search space used: 130200

Query= sp|P09189|

```
HSP7C_PETHY_Heat_shock_cognate_70_kDa_protein_OS=Petunia_h
ybrida_GN=HSP70_PE=2_SV=1

Length=651

Subject= 290688-74_4_ORF1
>sp|P09189|HSP7C_PETHY_Heat_shock_cognate_70_kDa_protein_OS=Petunia_
hybrida_GN=HSP70_PE=2_SV=1|||2e-156

Length=263


 Score =   443 bits (1140),  Expect = 2e-156, Method: Compositional
matrix adjust.
 Identities = 214/230 (93%), Positives = 221/230 (96%), Gaps = 0/230
(0%)

Query  1
MAGKGEGPAIGIDLGTTYSCVGVWQHDRVEIIANDQGNRTTPSYVGFTDTERLIGDAAKN  60
            +A   EGPAIGIDLGTTYSCVGVWQHDRVEIIANDQGNRTTPSYV
FTDTERLIGDAAKN
Sbjct  34
LAMAKEGPAIGIDLGTTYSCVGVWQHDRVEIIANDQGNRTTPSYVAFTDTERLIGDAAKN  93

Query  61
QVAMNPINTVFDAKRLIGRRFSDPSVQSDIKLWPFKVIPGPGDKPMIVVTYKGEEKQFAA  120
            QVAMNPINTVFDAKRLIGRRFSDP+VQSD KLWPFKVIPGPGDKPMIVV YKGEE
+QFAA
Sbjct  94
QVAMNPINTVFDAKRLIGRRFSDPTVQSDSKLWPFKVIPGPGDKPMIVVQYKGEERQFAA  153

Query  121
EEISSMVLTKMKEIAEAYLGTTIKNAVVTVPAYFNDSQRQATKDAGVIAGLNVMRIINEP  180
            EEISSMVL KMKEIAEA+LG+TIKNAVVTVPAYFNDSQRQATKDAGVI
+GLNVMRIINEP
Sbjct  154
EEISSMVLVKMKEIAEAFLGSTIKNAVVTVPAYFNDSQRQATKDAGVISGLNVMRIINEP  213

Query  181  TAAAIAYGLDKKASSAGEKNVLIFDLGGGTFDVSLLTIEEGIFEVKATAG  230
            TAAAIAYGLDKK +S GEKNVLIFDLGGGTFDVSLLTIEEGIFEVKATAG
Sbjct  214  TAAAIAYGLDKKTTSQGEKNVLIFDLGGGTFDVSLLTIEEGIFEVKATAG  263


 Score = 15.8 bits (29),  Expect = 2.4, Method: Compositional matrix
adjust.
 Identities = 10/46 (22%), Positives = 21/46 (46%), Gaps = 6/46
(13%)

Query  509  ITNDKGR------LSKEEIERMVQEAEKYKSEDEELKKKVEAKNAL  548
            I ND+G       ++  + ER++ +A K +     +    +AK  +
Sbjct  65   IANDQGNRTTPSYVAFTDTERLIGDAAKNQVAMNPINTVFDAKRLI  110


 Score = 14.2 bits (25),  Expect = 8.6, Method: Compositional matrix
```

```
adjust.
 Identities = 9/24 (38%), Positives = 12/24 (50%), Gaps = 1/24 (4%)

Query  374  AVAYGAAVQAAILSGEGNEKVQDL  397
            A+AYG   +     GE N  + DL
Sbjct  217  AIAYGLD-KKTTSQGEKNVLIFDL  239



Lambda      K        H        a         alpha
   0.313    0.132    0.371    0.792     4.96

Gapped
Lambda      K        H        a         alpha    sigma
   0.267   0.0410    0.140     1.90     42.6     43.6

Effective search space used: 130200




Matrix: BLOSUM62
Gap Penalties: Existence: 11, Extension: 1
Neighboring words threshold: 11
Window for multiple hits: 40





Query= sp|P09444|LEA34_GOSHI_Late_embryogenesis_abundant_protein_D-
34_OS=Gossypium_hirsutum_PE=4_SV=1

Length=264

Subject= 43963-384_3_ORF2
>sp|P09444|LEA34_GOSHI_Late_embryogenesis_abundant_protein_D-
34_OS=Gossypium_hirsutum_PE=4_SV=1|||2e-37

Length=297


 Score =   137 bits (345),  Expect = 1e-42, Method: Compositional
matrix adjust.
 Identities = 96/235 (41%), Positives = 131/235 (56%), Gaps = 27/235
(11%)

Query  1    MSQGQPRRPQQPAGQGENQEPIKYGDVFN--
VSGELANKPIAPQDAAMMQTAETQVLGQT  58
            MSQ QP RP            + Y DVF   V GE A++P+  +DAA+MQ+AET
LG+T
Sbjct  16   MSQEQPARP------------
VTYKDVFGDAVQGEAADQPVTREDAALMQSAETIGLGKT  63
```

```
Query  59   QKGGTAAVMQAAATRNEQ---
VGVVGHNDITDIAGEQGVTLAETDVAGRRIITEAVAGQV  115
            +KGG  A+MQ+AA +N +   +  V H+D    A E G  L ET + G+ +  E +
Q
Sbjct  64   RKGGAGALMQSAADKNVRDHIIDPVSHSD----
AAESGFGLRETIIDGKVVQQEYIGQQR  119

Query  116
VGQYVQATPVMTSQVGVVLQNAITIGEALEATAKTAGDKPVDQSDAAAVQAAEVRATGSN  175
            V +     P +     +     ITIGEALE  A    DK +D++ A A+Q+AE
RATG
Sbjct  120  VSEATSFVPTLPE---
IAATQGITIGEALEEVAVNEPDKIMDEATARAIQSAEARATGIP  176

Query  176  VIIPGGLAATAQSAAAHNATLDRDEEKIKLNQVLTGATAKLPADKAVTRQDAEGV
230
            +   GGL ATAQSAA  NA    + EKI +  VL  AT ++  DK VT++D E +
Sbjct  177  ISFKGGLGATAQSAAQKNA---HNGEKITILDVLDDATERMLVDKVVTKEDVEKL
228



Lambda      K        H        a         alpha
   0.308    0.123    0.327    0.792     4.96

Gapped
Lambda      K        H        a         alpha    sigma
   0.267   0.0410    0.140     1.90     42.6     43.6

Effective search space used: 64498



Matrix: BLOSUM62
Gap Penalties: Existence: 11, Extension: 1
Neighboring words threshold: 11
Window for multiple hits: 40



Query= sp|P0C7Q8|
DA1_ARATH_Protein_DA1_OS=Arabidopsis_thaliana_GN=DA1_PE=1_
SV=1

Length=532

Subject= 41629-395_1_ORF2
>sp|P0C7Q8|DA1_ARATH_Protein_DA1_OS=Arabidopsis_thaliana_GN=DA1_PE=1
_SV=1|||0

Length=475
```

```
 Score =   555 bits (1430),  Expect = 0.0, Method: Compositional
matrix adjust.
 Identities = 291/538 (54%), Positives = 373/538 (69%), Gaps =
74/538 (14%)

Query  1
MGWFNKIFKGSNQRLRVGNNKHNHNVYYDNYPTASHDDEPSAADTDADNDEPHHTQEPST  60
            M W ++IFKGS+   RVG     +   YDN          S A+T+ D D     Q
Sbjct  6    MKWLDRIFKGSSHH-RVGE----YESRYDN----------
SDANTNIDRDYKSREQ----  46

Query  61
SEDNTSNDQENEDIDRAIALSLLEENQEQTSISGKYSMPVDEDEQLARALQESMVVGNSP  120
                     E+E++D AIALSL E +  +T+  G +S  ++ DE LARALQ S+
Sbjct  47   --------VEDEELDHAIALSLSEADYRRTN--
GIHSPQLEADEDLARALQASL------  90

Query  121  RHKSGSTYDNGNAYGAGDLYGNGHMYGGGNVYANGDIYYPRPITFQMDF----
RICAGCN  176
                             DL               D   PRP+  ++      R+C
GC
Sbjct  91   -----------------DL----------------
DEPSPRPVAARVQVPTGRRVCEGCK  117

Query  177
MEIGHGRFLNCLNSLWHPECFRCYGCSQPISEYEFSTSGNYPFHKACYRERYHPKCDVCS  236
             EIG+GRFL+C+ S+WHP+CFRCY C  PIS++EFS SGN P+HK+CY+E +HP
+CDVC
Sbjct  118
NEIGYGRFLSCMGSVWHPDCFRCYACHSPISDHEFSMSGNNPYHKSCYKELFHPRCDVCK  177

Query  237
HFIPTNHAGLIEYRAHPFWVQKYCPSHEHDATPRCCSCERMEPRNTRYVELNDGRKLCLE  296
             FIPTN  GLIEYRAHPFW Q+YCPSHE D+TPRCCSCER+E ++  Y+ L
DGRKLCLE
Sbjct  178
EFIPTNSTGLIEYRAHPFWGQRYCPSHEIDSTPRCCSCERVESKDAIYISLEDGRKLCLE  237

Query  297
CLDSAVMDTMQCQPLYLQIQNFYEGLNMKVEQEVPLLLVERQALNEAREGEKNGHYHMPE  356
            CLDSA+MDT +CQPLY +I++FYEG+NMK+EQ++P+LLV+RQALNEA EGEK GH
+HMPE
Sbjct  238
CLDSAIMDTGECQPLYHEIRDFYEGMNMKIEQQIPMLLVQRQALNEAMEGEKEGHHHMPE  297

Query  357  TRGLCLSEEQTVSTVRKRSKHGTGKWAGNI-
TEPYKLTRQCEVTAILILFGLPRLLTGSI  415
            TRGLCLSEEQTV+ + ++ + G G    ++ TE  KLTR CEVTAIL+L
+GLPRLLTGSI
Sbjct  298
TRGLCLSEEQTVNIIFRKPRIGAGNSIIDMRTESQKLTRHCEVTAILVLYGLPRLLTGSI  357
```

Query 416
LAHEMMHAWMRLKGFRTLSQDVEEGICQVMAHKWLDAELAAGSTNSNAASSSSSSQGLK- 474
LAHE+MHAW+RL GFR L DVEEGICQV+AH WL++E+ AGST+++++SSSSSS
Sbjct 358
LAHELMHAWLRLNGFRNLQPDVEEGICQVLAHMWLESEVMAGSTSASSSSSSSSSASKNP 417

Query 475
KGPRSQYERKLGEFFKHQIESDASPVYGDGFRAGRLAVHKYGLRKTLEHIQMTGRFPV 532
KGP+S E+KLGEFF HQI D+SP+YG+GFRAG ++ ++GLR+TLEHI+MTG
FP+
Sbjct 418
KGPKSDVEKKLGEFFLHQIAMDSSPIYGEGFRAGHASMVQFGLRRTLEHIKMTGGFPI 475

Lambda	K	H	a	alpha
0.317	0.134	0.418	0.792	4.96

Gapped

Lambda	K	H	a	alpha	sigma
0.267	0.0410	0.140	1.90	42.6	43.6

Effective search space used: 219618

Query= sp|P0C7Q8|
DA1_ARATH_Protein_DA1_OS=Arabidopsis_thaliana_GN=DA1_PE=1_
SV=1

Length=532

Subject= 69992-302_5_ORF2
>sp|P0C7Q8|DA1_ARATH_Protein_DA1_OS=Arabidopsis_thaliana_GN=DA1_PE=1
_SV=1|||0

Length=475

Score = 554 bits (1428), Expect = 0.0, Method: Compositional
matrix adjust.
Identities = 289/538 (54%), Positives = 373/538 (69%), Gaps =
74/538 (14%)

Query 1
MGWFNKIFKGSNQRLRVGNNKHNHNVYYDNYPTASHDDEPSAADTDADNDEPHHTQEPST 60
M W ++IFKGS+ RVG + YDN S A+T+ D D Q
Sbjct 6 MKWLDRIFKGSSHH-RVGE----YESRYDN----------
SDANTNIDRDYKSREQ---- 46

Query 61
SEDNTSNDQENEDIDRAIALSLLEENQEQTSISGKYSMPVDEDEQLARALQESMVVGNSP 120
E+E++D AIALSL E + +T+ G ++ ++ DE LARALQ S+

```
Sbjct  47   --------VEDEELDHAIALSLSEADYRRTN--
GLHTPQLEADEDLARALQASL------  90

Query  121  RHKSGSTYDNGNAYGAGDLYGNGHMYGGGNVYANGDIYYPRPITFQMDF----
RICAGCN  176
                              DL                D   PRP+  ++      R+C
GC
Sbjct  91   -----------------DL---------------
DEPSPRPVAARVQVPTGRRVCEGCK  117

Query  177
MEIGHGRFLNCLNSLWHPECFRCYGCSQPISEYEFSTSGNYPFHKACYRERYHPKCDVCS  236
             EIG+GRFL+C+ S+WHP+CFRCY C  PIS++EFS SGN P+HK+CY+E +HP
+CDVC
Sbjct  118
NEIGYGRFLSCMGSVWHPDCFRCYACHSPISDHEFSMSGNNPYHKSCYKELFHPRCDVCK  177

Query  237
HFIPTNHAGLIEYRAHPFWVQKYCPSHEHDATPRCCSCERMEPRNTRYVELNDGRKLCLE  296
             FIPTN  GLIEYRAHPFW Q+YCPSHE D+TPRCCSCER+E ++  Y+ L
DGRKLCLE
Sbjct  178
EFIPTNSTGLIEYRAHPFWGQRYCPSHEIDSTPRCCSCERVESKDAIYISLEDGRKLCLE  237

Query  297
CLDSAVMDTMQCQPLYLQIQNFYEGLNMKVEQEVPLLLVERQALNEAREGEKNGHYHMPE  356
            CLDSA+MDT +CQPLY +I++FYEG+NMK+EQ++P+LLV+RQALNEA EGEK GH
+HMPE
Sbjct  238
CLDSAIMDTGECQPLYHEIRDFYEGMNMKIEQQIPMLLVQRQALNEAMEGEKEGHHHMPE  297

Query  357  TRGLCLSEEQTVSTVRKRSKHGTGKWAGNI-
TEPYKLTRQCEVTAILILFGLPRLLTGSI  415
            TRGLCLSEEQTV+ + ++ + G G    ++ TE  KLTR CEVTAIL+L
+GLPRLLTGSI
Sbjct  298
TRGLCLSEEQTVNIIFRKPRIGAGNSIIDMRTESQKLTRHCEVTAILVLYGLPRLLTGSI  357

Query  416
LAHEMMHAWMRLKGFRTLSQDVEEGICQVMAHKWLDAELAAGSTNSNAASSSSSSQGLK-  474
            LAHE+MHAW+RL GFR L  DVEEGICQV+AH WL++E+ AGS++++++SSSSSS
Sbjct  358
LAHELMHAWLRLNGFRNLQPDVEEGICQVLAHMWLESEVMAGSSSASSSSSSSSSASKNP  417

Query  475
KGPRSQYERKLGEFFKHQIESDASPVYGDGFRAGRLAVHKYGLRKTLEHIQMTGRFPV  532
            KGP+S  E+KLGEFF HQI  D+SP+YG+GFRAG  ++ ++GLR+TLEHI+MTG
FP+
Sbjct  418
KGPKSDVEKKLGEFFLHQIAMDSSPIYGEGFRAGHASMVQFGLRRTLEHIKMTGGFPI  475



Lambda      K        H        a         alpha
```

```
   0.317    0.134    0.418    0.792     4.96

Gapped
Lambda      K        H        a         alpha    sigma
   0.267   0.0410    0.140     1.90     42.6     43.6

Effective search space used: 219618
```

```
Matrix: BLOSUM62
Gap Penalties: Existence: 11, Extension: 1
Neighboring words threshold: 11
Window for multiple hits: 40
```

```
Query= sp|P0CW97|
PCR3_ARATH_Protein_PLANT_CADMIUM_RESISTANCE_3_OS=Arabidops
is_thaliana_GN=PCR3_PE=3_SV=1

Length=152

Subject= 163213-176_3_ORF1
>sp|P0CW97|PCR3_ARATH_Protein_PLANT_CADMIUM_RESISTANCE_3_OS=Arabidop
sis_thaliana_GN=PCR3_PE=3_SV=1|||1e-36

Length=147


 Score = 98.6 bits (244),  Expect = 5e-31, Method: Compositional
matrix adjust.
 Identities = 53/119 (45%), Positives = 74/119 (62%), Gaps = 0/119
(0%)

Query  14
GEWSTGFCDCFSDCQNCCITWLCPCITFGQVADIVDRGNTSCGTAGALYVLLAAITGCGC  73
            G+WSTG C C  D  +CC    CPC++ G++ +++D G TSC   G ++ LL
TG GC
Sbjct  19
GQWSTGLCGCCEDGSSCCCACCCPCVSVGRIVNVLDNGQTSCFEGGLIFYLLHQFTGLGC  78

Query  74
LYSCIYRGKIRAQYNIRGDGCTDCLKHFCCELCALTQEYRELKHRGFDMSLGWAGNVEK  132
            LY+C YR K+R +YN+  D C D L   CC  C+++Q YRELK+R  D S G+
E+
Sbjct  79
LYTCGYRKKLRIRYNLPEDPCNDVLTDCCCLCCSISQTYRELKNRNIDPSQGYVNVQER  137
```

```
Lambda      K        H        a         alpha
   0.326    0.139    0.488    0.792     4.96

Gapped
Lambda      K        H        a         alpha    sigma
   0.267   0.0410    0.140     1.90     42.6     43.6

Effective search space used: 17816



Query= sp|P0CW97|
PCR3_ARATH_Protein_PLANT_CADMIUM_RESISTANCE_3_OS=Arabidops
is_thaliana_GN=PCR3_PE=3_SV=1

Length=152

Subject= 399894-23_5_ORF1
>sp|P0CW97|PCR3_ARATH_Protein_PLANT_CADMIUM_RESISTANCE_3_OS=Arabidop
sis_thaliana_GN=PCR3_PE=3_SV=1|||1e-36

Length=147


 Score = 98.6 bits (244),  Expect = 5e-31, Method: Compositional
matrix adjust.
 Identities = 53/119 (45%), Positives = 74/119 (62%), Gaps = 0/119
(0%)

Query  14
GEWSTGFCDCFSDCQNCCITWLCPCITFGQVADIVDRGNTSCGTAGALYVLLAAITGCGC  73
            G+WSTG C C  D  +CC    CPC++ G++ +++D G TSC   G ++ LL
TG GC
Sbjct  19
GQWSTGLCGCCEDGSSCCCACCCPCVSVGRIVNVLDNGQTSCFEGGLIFYLLHQFTGLGC  78

Query  74
LYSCIYRGKIRAQYNIRGDGCTDCLKHFCCELCALTQEYRELKHRGFDMSLGWAGNVEK  132
            LY+C YR K+R +YN+  D C D L   CC  C+++Q YRELK+R  D S G+
E+
Sbjct  79
LYTCGYRKKLRIRYNLPEDPCNDVLTDCCCLCCSISQTYRELKNRNIDPSQGYVNVQER  137



Lambda      K        H        a         alpha
   0.326    0.139    0.488    0.792     4.96

Gapped
Lambda      K        H        a         alpha    sigma
   0.267   0.0410    0.140     1.90     42.6     43.6

Effective search space used: 17816
```

Matrix: BLOSUM62
Gap Penalties: Existence: 11, Extension: 1
Neighboring words threshold: 11
Window for multiple hits: 40

Query= sp|P11143|
HSP70_MAIZE_Heat_shock_70_kDa_protein_OS=Zea_mays_GN=HSP70
_PE=3_SV=2

Length=645

Subject= 7820-836_2_ORF1
>sp|P11143|HSP70_MAIZE_Heat_shock_70_kDa_protein_OS=Zea_mays_GN=HSP7
0_PE=3_SV=2|||0

Length=279

Score = 507 bits (1305), Expect = 0.0, Method: Compositional
matrix adjust.
Identities = 251/278 (90%), Positives = 263/278 (95%), Gaps = 2/278
(1%)

Query 324 CLRDAKMDKSSVHDVVLVGGSTRIPKVQQL-
QDFFNGKELCKSINPDEAVAYGAAVQAAI 382
CLRDAKMDK+S+HDVVLVGGSTRIPKVQQL
QDFFNGKELCKSINPDEAVAYGAAVQAAI
Sbjct 1
CLRDAKMDKNSIHDVVLVGGSTRIPKVQQLLQDFFNGKELCKSINPDEAVAYGAAVQAAI 60

Query 383 LSGEGNER-
SDLLLLDVTPLSLGLETAGGVMTVLIPRNTTIPTKKEQVFSTYSDNQPGVL 441
LSGEGNE+
DLLLLDVTPLSLGLETAGGVMTVLIPRNTTIPTKKEQVFSTYSDNQPGVL
Sbjct 61
LSGEGNEKVQDLLLLDVTPLSLGLETAGGVMTVLIPRNTTIPTKKEQVFSTYSDNQPGVL 120

Query 442
IQVYEGERARTKDNNLLGKFELSGIPPAPRGVPQITVTFDIDVNNILNVSAEDKTTGQKN 501
IQVYEGER RTKDNNLLGKFELSGIPPAPRGVPQITV FDID N
ILNVSAEDKTTGQKN
Sbjct 121
IQVYEGERTRTKDNNLLGKFELSGIPPAPRGVPQITVCFDIDANGILNVSAEDKTTGQKN 180

Query 502
KITITNDKGRLSKEEIEKMVQEAEKYKAEDEEVKKKVDAKNALENYAYNMRNTIKDDKIA 561

```
           KITITNDKGRLSKEEIEKMVQEAEKYK+EDEE KKKV++KNALENYAYNMRNTIKD
+KIA
Sbjct  181
KITITNDKGRLSKEEIEKMVQEAEKYKSEDEEHKKKVESKNALENYAYNMRNTIKDEKIA  240

Query  562  SKLPAEDKKKIEDAVDGAISWLDSNQLAEVEEFEDKMK  599
             KL A DKKK+E+AV+ AI WL+ NQLAE EEF+DKMK
Sbjct  241  GKLDAADKKKVEEAVEQAIQWLEHNQLAEAEEFDDKMK  278


 Score = 17.3 bits (33),  Expect = 0.80, Method: Compositional
matrix adjust.
 Identities = 14/36 (39%), Positives = 20/36 (56%), Gaps = 3/36 (8%)

Query  182  AIAYGLD-KKATSSGEKNVLIFDLGGGTFDVSLLTI  216
            A+AYG   + A  SGE N  + DL     DV+ L++
Sbjct  49   AVAYGAAVQAAILSGEGNEKVQDL--LLLDVTPLSL  82


 Score = 15.8 bits (29),  Expect = 2.9, Method: Compositional matrix
adjust.
 Identities = 5/12 (42%), Positives = 9/12 (75%), Gaps = 0/12 (0%)

Query  633  GGSGAGPKIEEV  644
            GGS   PK++++
Sbjct  19   GGSTRIPKVQQL  30



Lambda      K        H        a         alpha
   0.313    0.132    0.368    0.792     4.96

Gapped
Lambda      K        H        a         alpha    sigma
   0.267   0.0410    0.140     1.90     42.6     43.6

Effective search space used: 151411




Matrix: BLOSUM62
Gap Penalties: Existence: 11, Extension: 1
Neighboring words threshold: 11
Window for multiple hits: 40




Query= sp|P13240|
DR206_PEA_Disease_resistance_response_protein_206_OS=Pisum
_sativum_GN=PI206_PE=2_SV=2
```

```
Length=184

Subject= 130267-215_1_ORF2
>sp|P13240|DR206_PEA_Disease_resistance_response_protein_206_OS=Pisu
m_sativum_GN=PI206_PE=2_SV=2|||2e-13

Length=187


 Score = 46.6 bits (109),  Expect = 2e-11, Method: Compositional
matrix adjust.
 Identities = 30/90 (33%), Positives = 45/90 (50%), Gaps = 2/90 (2%)

Query  73   FGNIIVFDDPI--
TLSHSLSSKQVGRAQGFYIYDTKNTYTSWLSFTFVLNSTHHQGTITF  130
            FG +  FD+ +  TLS    S  +GR QG+Y    ++     L+ TF  N
GT +
Sbjct  73
FGVLNTFDNLLSKTLSVDNPSDLLGRVQGWYGDCGQDELVLCLAQTFTYNDGTFNGTFSL  132

Query  131  AGADPIVAKTRDISVTGGTGDFFMHRGIAT  160
             G     A +++ ++ GGTG F   RG+AT
Sbjct  133  IGVSVATAPSKEAAIVGGTGGFAFCRGVAT  162



Lambda      K        H        a         alpha
   0.324    0.138    0.422    0.792     4.96

Gapped
Lambda      K        H        a         alpha    sigma
   0.267   0.0410    0.140     1.90     42.6     43.6

Effective search space used: 27720




Query= sp|P13240|
DR206_PEA_Disease_resistance_response_protein_206_OS=Pisum
_sativum_GN=PI206_PE=2_SV=2

Length=184

Subject= 14211-641_4_ORF1
>sp|P13240|DR206_PEA_Disease_resistance_response_protein_206_OS=Pisu
m_sativum_GN=PI206_PE=2_SV=2|||3e-22

Length=196


 Score = 77.0 bits (188),  Expect = 3e-22, Method: Compositional
matrix adjust.
 Identities = 48/141 (34%), Positives = 75/141 (53%), Gaps = 6/141
```

(4%)

Query 24   KRKPYKPC--
KNLVFYFHDILYNGKNAANATSAIVAAPEGVSLTKLAPQSHFGNIIVFDD 81
            K++ KPC   ++ FY H+ YN   AA   +   +   +   P+ FG
F+D
Sbjct 37   KKRASKPCGPSSVSFYLHNTEYN---AAVNNTGYFNSVYSFTPPPFIPK-
FFGATAAFED 92

Query 82
PITLSHSLSSKQVGRAQGFYIYDTKNTYTSWLSFTFVLNSTHHQGTITFAGADPIVAKTR 141
            P+T+S + +SKQ+G AQG Y+ D+   +T + FT ++   HQGT++ G + +
Sbjct 93
PLTVSRANNSKQIGVAQGLYLADSIVEFTLFYVFTANISDGQHQGTLSIMGQERLTQPVS 152

Query 142 DISVTGGTGDFFMHRGIATIT 162
             ++V GGTGDF   GIAT T
Sbjct 153 YLTVLGGTGDFAGAHGIATST 173

Lambda   K   H   a   alpha
   0.324   0.138   0.422   0.792   4.96

Gapped
Lambda   K   H   a   alpha   sigma
   0.267   0.0410   0.140   1.90   42.6   43.6

Effective search space used: 27720

Query= sp|P13240|
DR206_PEA_Disease_resistance_response_protein_206_OS=Pisum
_sativum_GN=PI206_PE=2_SV=2

Length=184

Subject= 14216-641_4_ORF1
>sp|P13240|DR206_PEA_Disease_resistance_response_protein_206_OS=Pisu
m_sativum_GN=PI206_PE=2_SV=2|||3e-22

Length=195

 Score = 77.0 bits (188), Expect = 3e-22, Method: Compositional
matrix adjust.
 Identities = 48/141 (34%), Positives = 75/141 (53%), Gaps = 6/141
(4%)

Query 24   KRKPYKPC--
KNLVFYFHDILYNGKNAANATSAIVAAPEGVSLTKLAPQSHFGNIIVFDD 81
            K++ KPC   ++ FY H+ YN   AA   +   +   +   P+ FG

```
F+D
Sbjct  37   KKRASKPCGPSSVSFYLHNTEYN---AAVNNTGYFNSVYSFTPPPFIPK-
FFGATAAFED  92

Query  82
PITLSHSLSSKQVGRAQGFYIYDTKNTYTSWLSFTFVLNSTHHQGTITFAGADPIVAKTR  141
            P+T+S + +SKQ+G AQG Y+ D+   +T +  FT  ++   HQGT++  G + +
Sbjct  93
PLTVSRANNSKQIGVAQGLYLADSIVEFTLFYVFTANISDGQHQGTLSIMGQERLTQPVS  152

Query  142  DISVTGGTGDFFMHRGIATIT  162
             ++V GGTGDF    GIAT T
Sbjct  153  YLTVLGGTGDFAGAHGIATST  173



Lambda      K        H        a         alpha
   0.324    0.138    0.422    0.792     4.96

Gapped
Lambda      K        H        a         alpha    sigma
   0.267   0.0410    0.140     1.90     42.6     43.6

Effective search space used: 27720




Query= sp|P13240|
DR206_PEA_Disease_resistance_response_protein_206_OS=Pisum
_sativum_GN=PI206_PE=2_SV=2

Length=184

Subject= 201664-136_1_ORF1
>sp|P13240|DR206_PEA_Disease_resistance_response_protein_206_OS=Pisu
m_sativum_GN=PI206_PE=2_SV=2|||2e-17

Length=232


 Score = 63.9 bits (154),  Expect = 2e-17, Method: Compositional
matrix adjust.
 Identities = 54/183 (30%), Positives = 81/183 (44%), Gaps = 16/183
(9%)

Query  3
SKLLVLFVFVMLFALSSAIPNKRKPYKPCKNLVFYFHDILYNGKNAANATSAIVAAPEGV  62
            S L +  +F  L AL+S  P         K+L FY +  + N  N  N      A
Sbjct  61   SALALSLLFAPLPALASKGP---------
KHLEFYMYIAVQNNSNLHNPNVTFTAVQSAQ  111

Query  63
SLTKLAPQSHFGNIIVFDDPITLSHSLSSKQVGRAQGFYIYDTKNTYTSWLSFTFVLNST  122
```

```
             L+  A  + FG I  FD+P+T    L+S  +GR QG+Y    ++  T +L  TF
+
Sbjct  112  PLS--
AQPNSFGIIHTFDNPLTSGADLNSTHLGRVQGWYGDVGQDLLTLFLVQTFTWDDG  169

Query  123  HHQGTITFAGADPIVAKTRDISVTGGTGDFFMHRGIA-----
TITTDAFEGEAYFRLGVY  177
             + GT +  G D      +   + GGTGDF   RG+A     +  +  FE  ++F
V
Sbjct  170
KYNGTFSLLGVDIATDPQKFAPIVGGTGDFAYARGVAQQSLLSTASINFETVSWFHYIVD  229

Query  178  IKF  180
             K+
Sbjct  230  FKY  232



Lambda      K        H        a         alpha
   0.324    0.138    0.422    0.792     4.96

Gapped
Lambda      K        H        a         alpha    sigma
   0.267   0.0410    0.140     1.90     42.6     43.6

Effective search space used: 27720




Query= sp|P13240|
DR206_PEA_Disease_resistance_response_protein_206_OS=Pisum
_sativum_GN=PI206_PE=2_SV=2

Length=184

Subject= 201781-136_2_ORF2
>sp|P13240|DR206_PEA_Disease_resistance_response_protein_206_OS=Pisu
m_sativum_GN=PI206_PE=2_SV=2|||9e-18

Length=185


 Score = 62.4 bits (150),  Expect = 3e-17, Method: Compositional
matrix adjust.
 Identities = 42/131 (32%), Positives = 61/131 (47%), Gaps = 2/131
(2%)

Query  29
KPCKNLVFYFHDILYNGKNAANATSAIVAAPEGVSLTKLAPQSHFGNIIVFDDPITLSHS  88
            K  K+L FY +  + N  N  N      A      L+  A  + FG I  FD+P+T
Sbjct  31   KGPKHLEFYMYIAVQNNSNLHNPNVTFTAVQSAQPLS--
AQPNSFGIIHTFDNPLTSGAD  88
```

```
Query  89
LSSKQVGRAQGFYIYDTKNTYTSWLSFTFVLNSTHHQGTITFAGADPIVAKTRDISVTGG  148
            L+S  +GR QG+Y    ++  T +L  TF  +   + GT +  G D      +
+ GG
Sbjct  89
LNSTHLGRVQGWYGDVGQDLLTLFLVQTFTWDDGKYNGTFSLLGVDIATDPQKFAPIVGG  148

Query  149  TGDFFMHRGIA  159
            TGDF   RG+A
Sbjct  149  TGDFAYARGVA  159



Lambda      K        H        a         alpha
   0.324    0.138    0.422    0.792     4.96

Gapped
Lambda      K        H        a         alpha    sigma
   0.267   0.0410    0.140     1.90     42.6     43.6

Effective search space used: 27720




Query= sp|P13240|
DR206_PEA_Disease_resistance_response_protein_206_OS=Pisum
_sativum_GN=PI206_PE=2_SV=2

Length=184

Subject= 213971-126_6_ORF1
>sp|P13240|DR206_PEA_Disease_resistance_response_protein_206_OS=Pisu
m_sativum_GN=PI206_PE=2_SV=2|||3e-23

Length=198


 Score = 74.3 bits (181),  Expect = 3e-21, Method: Compositional
matrix adjust.
 Identities = 50/165 (30%), Positives = 83/165 (50%), Gaps = 10/165
(6%)

Query  3    SKLLVLFVFVMLFALSSAIPNKRKPYKPC----KNLVFYFHDILYNGK-
NAANATSAIVA  57
             K LV+ + V    +++         K C     ++ FY H++ YN + N     +
++
Sbjct  16
EKQLVVLLVVWCIMIAAGPYAGLADCKSCACGPNSVSFYLHNLEYNAQVNNTGYFNSVYG  75

Query  58
APEGVSLTKLAPQSHFGNIIVFDDPITLSHSLSSKQVGRAQGFYIYDTKNTYTSWLSFTF  117
             P     +       FG + VF+DP+T S + +SKQ+G AQG Y+ D K   + +
FT
```

```
Sbjct  76   PPPPPLPSGF-----
FGAMAVFEDPLTASRANNSKQIGVAQGLYVNDGKTDVSFFYVFTA  130

Query  118  VLNSTHHQGTITFAGADPIVAKTRDISVTGGTGDFFMHRGIATIT  162
             +++  HQGT++  G   ++     ++VTGGTGDF    G+AT T
Sbjct  131  NISTGEHQGTLSIMGQARLLHPVSYLTVTGGTGDFARAHGVATST  175



Lambda      K        H        a         alpha
   0.324    0.138    0.422    0.792     4.96

Gapped
Lambda      K        H        a         alpha    sigma
   0.267   0.0410    0.140     1.90     42.6     43.6

Effective search space used: 27720




Query= sp|P13240|
DR206_PEA_Disease_resistance_response_protein_206_OS=Pisum
_sativum_GN=PI206_PE=2_SV=2

Length=184

Subject= 269834-87_4_ORF2
>sp|P13240|DR206_PEA_Disease_resistance_response_protein_206_OS=Pisu
m_sativum_GN=PI206_PE=2_SV=2|||7e-18

Length=199


 Score = 60.8 bits (146),  Expect = 1e-16, Method: Compositional
matrix adjust.
 Identities = 47/137 (34%), Positives = 65/137 (47%), Gaps = 6/137
(4%)

Query  26   KPYKPCKNLVFYFHDILYNGKNAANATSAIVAAPEGVSLTKLAPQS-
HFGNIIVFDDPIT  84
            K  K  K L FY   +  N     N      A     ++T   PQ+  FG I  FD
+PI+
Sbjct  40   KTSKKPKKLDFYMFIVPQNDTFLDNPKGQYTAVRSAAAVT---
PQAFAFGTIHTFDNPIS  96

Query  85   LSHSLS--
SKQVGRAQGFYIYDTKNTYTSWLSFTFVLNSTHHQGTITFAGADPIVAKTRD  142
             + SL+  S  +GR QG+Y    ++  T  L+ TF  +  H +GT +  GAD
+
Sbjct  97
KAMSLANPSDVLGRVQGWYGNCGQHELTLCLAQTFTYDDGHFKGTFSLMGADIANDPLKY  156

Query  143  ISVTGGTGDFFMHRGIA  159
```

```
            + GGTGDF   RGIA
Sbjct  157  APIVGGTGDFAFCRGIA  173



Lambda      K        H        a         alpha
   0.324    0.138    0.422    0.792     4.96

Gapped
Lambda      K        H        a         alpha    sigma
   0.267   0.0410    0.140     1.90     42.6     43.6

Effective search space used: 27720




Query= sp|P13240|
DR206_PEA_Disease_resistance_response_protein_206_OS=Pisum
_sativum_GN=PI206_PE=2_SV=2

Length=184

Subject= 294639-71_1_ORF1
>sp|P13240|DR206_PEA_Disease_resistance_response_protein_206_OS=Pisu
m_sativum_GN=PI206_PE=2_SV=2|||3e-21

Length=212


 Score = 74.7 bits (182),  Expect = 3e-21, Method: Compositional
matrix adjust.
 Identities = 54/177 (31%), Positives = 83/177 (47%), Gaps = 17/177
(10%)

Query  1    MGSKLLVLFVFVMLFALSSAIPNKRKPYKP------C--KNLVFYFHDILYNG---
KNAA  49
            M   L VLFV     A+ +    K +  +       C    L FY H+ +Y+
+ A
Sbjct  24
MKQVLAVLFVVACCMAVGALAGGKEEECRERARKLSCGPSTLTFYLHNTVYDASVNNSVA  83

Query  50
NATSAIVAAPEGVSLTKLAPQSHFGNIIVFDDPITLSHSLSSKQVGRAQGFYIYDTKNTY  109
             + +++  AP  VS         FG +  F+DP+T   S  S+ +G AQGF+++D
+
Sbjct  84   GSFNSVTGAPPPVS------
PFFFGIMATFEDPLTTGASNDSEALGVAQGFFLFDALQEH  137

Query  110
TSWLSFTFVLNSTHHQGTITFAGADPIVAKTRDISVTGGTGDFFMHRGIATITTDAF  166
            T +  FT  ++   H GT+   G     +  R +SV GGTG F   RG+AT T
+F
Sbjct  138
```

```
TLFHVFTVNISQGSHTGTLAIMGQVREQSPVRSLSVVGGTGAFLGARGLATNTLISF  194



Lambda      K        H        a         alpha
   0.324    0.138    0.422    0.792     4.96

Gapped
Lambda      K        H        a         alpha    sigma
   0.267   0.0410    0.140     1.90     42.6     43.6

Effective search space used: 27720




Query= sp|P13240|
DR206_PEA_Disease_resistance_response_protein_206_OS=Pisum
_sativum_GN=PI206_PE=2_SV=2

Length=184

Subject= 326656-54_1_ORF1
>sp|P13240|DR206_PEA_Disease_resistance_response_protein_206_OS=Pisu
m_sativum_GN=PI206_PE=2_SV=2|||9e-17

Length=205


 Score = 53.9 bits (128),  Expect = 5e-14, Method: Compositional
matrix adjust.
 Identities = 41/128 (32%), Positives = 59/128 (46%), Gaps = 4/128
(3%)

Query  34
LVFYFHDILYNGKNAANATSAIVAAPEGVSLTKLAPQSHFGNIIVFDDPITLSHSLS--S  91
            L FY   I  N     N  +   A     ++T  A    FG I  FD+PI+ + SL
+  S
Sbjct  54   LEFYMFIIPQNDSFLDNPKAQYTAVRSAAAVT--
AQPFAFGTIHTFDNPISKTMSLAKPS  111

Query  92
KQVGRAQGFYIYDTKNTYTSWLSFTFVLNSTHHQGTITFAGADPIVAKTRDISVTGGTGD  151
              +GR QG+Y    +   T  L+ TF  +  H +GT +  GAD      +   +
GGTG
Sbjct  112
DVLGRVQGWYGNCGQKELTLCLAQTFTYDDGHFKGTFSLMGADIANDPLKYAPIVGGTGH  171

Query  152  FFMHRGIA  159
            F   RG++
Sbjct  172  FAFCRGVS  179
```

```
Lambda      K        H        a         alpha
   0.324    0.138    0.422    0.792     4.96

Gapped
Lambda      K        H        a         alpha    sigma
   0.267   0.0410    0.140     1.90     42.6     43.6

Effective search space used: 27720




Query= sp|P13240|
DR206_PEA_Disease_resistance_response_protein_206_OS=Pisum
_sativum_GN=PI206_PE=2_SV=2

Length=184

Subject= 327641-54_1_ORF1
>sp|P13240|DR206_PEA_Disease_resistance_response_protein_206_OS=Pisu
m_sativum_GN=PI206_PE=2_SV=2|||1e-18

Length=181


 Score = 58.5 bits (140),  Expect = 6e-16, Method: Compositional
matrix adjust.
 Identities = 44/128 (34%), Positives = 60/128 (47%), Gaps = 4/128
(3%)

Query  34
LVFYFHDILYNGKNAANATSAIVAAPEGVSLTKLAPQSHFGNIIVFDDPITLSHSLS--S  91
            L FY   I  N     N  +   A     ++T  A    FG I  FD+PI+ + SL
+  S
Sbjct  54   LEFYMFIIPQNDSFLDNPKAQYTAVRSAAAVT--
AQPFAFGTIHTFDNPISKTMSLAKPS  111

Query  92
KQVGRAQGFYIYDTKNTYTSWLSFTFVLNSTHHQGTITFAGADPIVAKTRDISVTGGTGD  151
              +GR QG+Y    +   T  L+ TF  +  H +GT +  GAD      +   +
GGTGD
Sbjct  112
DVLGRVQGWYGNCGQKELTLCLAQTFTYDDGHFKGTFSLMGADIANDPLKYAPIVGGTGD  171

Query  152  FFMHRGIA  159
            F   RGIA
Sbjct  172  FAFCRGIA  179



Lambda      K        H        a         alpha
   0.324    0.138    0.422    0.792     4.96

Gapped
```

```
Lambda      K        H        a         alpha    sigma
   0.267   0.0410    0.140     1.90     42.6     43.6

Effective search space used: 27720



Query= sp|P13240|
DR206_PEA_Disease_resistance_response_protein_206_OS=Pisum
_sativum_GN=PI206_PE=2_SV=2

Length=184

Subject= 337667-49_1_ORF2
>sp|P13240|DR206_PEA_Disease_resistance_response_protein_206_OS=Pisu
m_sativum_GN=PI206_PE=2_SV=2|||2e-17

Length=157


 Score = 62.4 bits (150),  Expect = 2e-17, Method: Compositional
matrix adjust.
 Identities = 47/157 (30%), Positives = 71/157 (45%), Gaps = 7/157
(4%)

Query  29
KPCKNLVFYFHDILYNGKNAANATSAIVAAPEGVSLTKLAPQSHFGNIIVFDDPITLSHS  88
            K  K+L FY +  + N  N  N      A      L+  A  + FG I  FD+P+T
Sbjct  3    KGPKHLEFYMYIAVQNNSNLHNPNQTFTAVQSAQPLS--
AQPNSFGIIHTFDNPLTSGAD  60

Query  89
LSSKQVGRAQGFYIYDTKNTYTSWLSFTFVLNSTHHQGTITFAGADPIVAKTRDISVTGG  148
            L+S  +GR QG+Y    ++  T +L  TF  +   + GT +  G D      +
+ GG
Sbjct  61
LNSTHLGRVQGWYGDVGQDLLTLFLVQTFTWDDGKYNGTFSLLGLDVATDPQKFAPIVGG  120

Query  149  TGDFFMHRGIA-----TITTDAFEGEAYFRLGVYIKF  180
            TGDF   RG+A     +  +  FE  ++F   V  K+
Sbjct  121  TGDFAYARGVAQQSLFSTASINFETVSWFHFIVDFKY  157



Lambda      K        H        a         alpha
   0.324    0.138    0.422    0.792     4.96

Gapped
Lambda      K        H        a         alpha    sigma
   0.267   0.0410    0.140     1.90     42.6     43.6

Effective search space used: 27720
```

```
Query= sp|P13240|
DR206_PEA_Disease_resistance_response_protein_206_OS=Pisum
_sativum_GN=PI206_PE=2_SV=2

Length=184

Subject= 339359-48_3_ORF1
>sp|P13240|DR206_PEA_Disease_resistance_response_protein_206_OS=Pisu
m_sativum_GN=PI206_PE=2_SV=2|||8e-12

Length=191


 Score = 47.8 bits (112),  Expect = 8e-12, Method: Compositional
matrix adjust.
 Identities = 49/176 (28%), Positives = 78/176 (44%), Gaps = 17/176
(10%)

Query  5
LLVLFVFVMLFALSSAIPNKRKPYKPCKNLVFYFHDILYNGKNAANATSAIVAAPEGVSL  64
            LLV+   V+L A   A   K K +K     VF +  +   G N+    +A+ +A
+
Sbjct  15   LLVMVAIVLLCASPQADARKLKHHKIPAFEVFNY--
LATQGPNSTQGYNAVRSAHPATTQ  72

Query  65
TKLAPQSHFGNIIVFDDPITLSHSLSSKQVGRAQGFYIYDTKNTYTSWLSFTFVLNSTH-  123
                  + FG+I  FD P+     L S+ +GR QG +          +++ TF+LN
T
Sbjct  73   A-----
NQFGDIYSFDMPLLKKKDLGSELLGRVQGTFTISQLTGGRVFVTETFILNGTSL  127

Query  124  -HQGTITFAGADPIVAKTRDISVTGGTGDFFMHRG------IATITTDAFEGEAYF
172
              +G+ +  G + I  K     +TGGT D+ +  G      +  I  D F G A+F
Sbjct  128  AFKGSFSALGIENI-GKMSSKPITGGTEDYELVSGNAFTTPLGGINFDKF-GNAFF
181



Lambda      K        H        a         alpha
   0.324    0.138    0.422    0.792     4.96

Gapped
Lambda      K        H        a         alpha    sigma
   0.267   0.0410    0.140     1.90     42.6     43.6

Effective search space used: 27720
```

```
Query= sp|P13240|
DR206_PEA_Disease_resistance_response_protein_206_OS=Pisum
_sativum_GN=PI206_PE=2_SV=2

Length=184

Subject= 341112-47_2_ORF1
>sp|P13240|DR206_PEA_Disease_resistance_response_protein_206_OS=Pisu
m_sativum_GN=PI206_PE=2_SV=2|||2e-10

Length=210


 Score = 39.7 bits (91),  Expect = 6e-09, Method: Compositional
matrix adjust.
 Identities = 27/90 (30%), Positives = 44/90 (49%), Gaps = 2/90 (2%)

Query  73   FGNIIVFDDPITLSH--
SLSSKQVGRAQGFYIYDTKNTYTSWLSFTFVLNSTHHQGTITF  130
            FG +  FD+ ++ +   + S+  +GR QG+Y    ++  T  L+ TF
GT +
Sbjct  96
FGVLNTFDNLLSKTQLFNNSADLIGRVQGWYGDCGQDQLTLCLTQTFTYADGTFNGTFSL  155

Query  131  AGADPIVAKTRDISVTGGTGDFFMHRGIAT  160
             G       +  +V GGTGDF   RG+A+
Sbjct  156  IGVSIATEPLKFAAVVGGTGDFAFCRGVAS  185



Lambda      K        H        a         alpha
   0.324    0.138    0.422    0.792     4.96

Gapped
Lambda      K        H        a         alpha    sigma
   0.267   0.0410    0.140     1.90     42.6     43.6

Effective search space used: 27720




Query= sp|P13240|
DR206_PEA_Disease_resistance_response_protein_206_OS=Pisum
_sativum_GN=PI206_PE=2_SV=2

Length=184

Subject= 342575-47_1_ORF2
>sp|P13240|DR206_PEA_Disease_resistance_response_protein_206_OS=Pisu
m_sativum_GN=PI206_PE=2_SV=2|||1e-10

Length=192
```

```
 Score = 40.0 bits (92),  Expect = 5e-09, Method: Compositional
matrix adjust.
 Identities = 27/90 (30%), Positives = 44/90 (49%), Gaps = 2/90 (2%)

Query  73   FGNIIVFDDPITLSH--
SLSSKQVGRAQGFYIYDTKNTYTSWLSFTFVLNSTHHQGTITF  130
            FG +  FD+ ++ +   + S+  +GR QG+Y    ++  T  L+ TF
GT +
Sbjct  78
FGVLNTFDNLLSKTQLFNNSADLIGRVQGWYGDCGQDQLTLCLTQTFTYADGTFNGTFSL  137

Query  131  AGADPIVAKTRDISVTGGTGDFFMHRGIAT  160
             G        +  +V GGTGDF   RG+A+
Sbjct  138  IGVSIATEPLKFAAVVGGTGDFAFCRGVAS  167



Lambda      K        H        a         alpha
   0.324    0.138    0.422    0.792     4.96

Gapped
Lambda      K        H        a         alpha    sigma
   0.267   0.0410    0.140     1.90     42.6     43.6

Effective search space used: 27720




Query= sp|P13240|
DR206_PEA_Disease_resistance_response_protein_206_OS=Pisum
_sativum_GN=PI206_PE=2_SV=2

Length=184

Subject= 344206-46_3_ORF2
>sp|P13240|DR206_PEA_Disease_resistance_response_protein_206_OS=Pisu
m_sativum_GN=PI206_PE=2_SV=2|||5e-10

Length=193


 Score = 40.0 bits (92),  Expect = 4e-09, Method: Compositional
matrix adjust.
 Identities = 27/90 (30%), Positives = 43/90 (48%), Gaps = 2/90 (2%)

Query  73   FGNIIVFDDPITLSHSL--
SSKQVGRAQGFYIYDTKNTYTSWLSFTFVLNSTHHQGTITF  130
            FG +  FD+ ++ +     S+  +GR QG+Y    ++  T  L+ TF
GT +
Sbjct  79
FGVLNTFDNLLSKTQLFNNSADLIGRVQGWYGDCGQDQLTLCLTQTFTYADGTFNGTFSL  138
```

```
Query  131  AGADPIVAKTRDISVTGGTGDFFMHRGIAT  160
             G        +  +V GGTGDF   RG+A+
Sbjct  139  IGVSIATEPLKFAAVVGGTGDFAFCRGVAS  168



Lambda      K        H        a         alpha
   0.324    0.138    0.422    0.792     4.96

Gapped
Lambda      K        H        a         alpha    sigma
   0.267   0.0410    0.140     1.90     42.6     43.6

Effective search space used: 27720




Query= sp|P13240|
DR206_PEA_Disease_resistance_response_protein_206_OS=Pisum
_sativum_GN=PI206_PE=2_SV=2

Length=184

Subject= 392060-26_4_ORF1
>sp|P13240|DR206_PEA_Disease_resistance_response_protein_206_OS=Pisu
m_sativum_GN=PI206_PE=2_SV=2|||4e-22

Length=196


 Score = 76.6 bits (187),  Expect = 4e-22, Method: Compositional
matrix adjust.
 Identities = 48/141 (34%), Positives = 75/141 (53%), Gaps = 6/141
(4%)

Query  24   KRKPYKPC--
KNLVFYFHDILYNGKNAANATSAIVAAPEGVSLTKLAPQSHFGNIIVFDD  81
            K++  KPC   ++ FY H+  YN   AA   +    +    +     P+  FG
F+D
Sbjct  37   KKRASKPCGPSSVSFYLHNTEYN---AAVNNTGYFNSVYSFTPPPFIPK-
FFGATAAFED  92

Query  82
PITLSHSLSSKQVGRAQGFYIYDTKNTYTSWLSFTFVLNSTHHQGTITFAGADPIVAKTR  141
            P+T+S + +SKQ+G AQG Y+ D+   +T +  FT  ++   HQGT++  G + +
Sbjct  93
PLTVSRANNSKQIGVAQGLYLADSIVEFTLFYVFTANISDGQHQGTLSIMGQERLTQPVS  152

Query  142  DISVTGGTGDFFMHRGIATIT  162
             ++V GGTGDF    GIAT T
Sbjct  153  YLTVLGGTGDFAGAHGIATST  173
```

```
Lambda      K        H        a         alpha
   0.324    0.138    0.422    0.792     4.96

Gapped
Lambda      K        H        a         alpha    sigma
   0.267   0.0410    0.140     1.90     42.6     43.6

Effective search space used: 27720



Query= sp|P13240|
DR206_PEA_Disease_resistance_response_protein_206_OS=Pisum
_sativum_GN=PI206_PE=2_SV=2

Length=184

Subject= 78986-284_3_ORF2
>sp|P13240|DR206_PEA_Disease_resistance_response_protein_206_OS=Pisu
m_sativum_GN=PI206_PE=2_SV=2|||3e-13

Length=232


 Score = 52.0 bits (123),  Expect = 4e-13, Method: Compositional
matrix adjust.
 Identities = 41/117 (35%), Positives = 58/117 (50%), Gaps = 11/117
(9%)

Query  73   FGNIIVFDDPITLSHSL-
SSKQVGRAQGFYIYDTKNTYTSWLSFTFVLNSTHHQGTITFA  131
            FG I  FD+P+    S  +S  +GRAQG+Y    ++  T + + T       + GT
+
Sbjct  118
FGIIHTFDNPLFSEPSRENSTHLGRAQGWYGDVGQDALTLFFALTVTYGDAKYNGTFSML  177

Query  132  G---ADPIVAKTRDISVTGGTGDFFMHRGIATIT-----
TDAFEGEAYFRLGVYIKF  180
            G   ADP  AK   + V GGTGDF   RGIA I+     T   E  ++F+  V  K
+
Sbjct  178  GVDVADPTAAKF--
VPVVGGTGDFAYARGIAQISLISDVTIDHETVSWFKYAVDFKY  232


 Score = 12.7 bits (21),  Expect = 5.1, Method: Compositional matrix
adjust.
 Identities = 4/11 (36%), Positives = 8/11 (73%), Gaps = 0/11 (0%)

Query  106  KNTYTSWLSFT  116
            + T+T  ++FT
Sbjct  37   QQTHTHMVTFT  47
```

```
Lambda      K        H        a         alpha
   0.324    0.138    0.422    0.792     4.96

Gapped
Lambda      K        H        a         alpha    sigma
   0.267   0.0410    0.140     1.90     42.6     43.6

Effective search space used: 27720




Query= sp|P13240|
DR206_PEA_Disease_resistance_response_protein_206_OS=Pisum
_sativum_GN=PI206_PE=2_SV=2

Length=184

Subject= 99810-250_6_ORF2
>sp|P13240|DR206_PEA_Disease_resistance_response_protein_206_OS=Pisu
m_sativum_GN=PI206_PE=2_SV=2|||1e-16

Length=199


 Score = 57.4 bits (137),  Expect = 2e-15, Method: Compositional
matrix adjust.
 Identities = 45/137 (33%), Positives = 64/137 (47%), Gaps = 6/137
(4%)

Query  26   KPYKPCKNLVFYFHDILYNGKNAANATSAIVAAPEGVSLTKLAPQS-
HFGNIIVFDDPIT  84
            K  K  K L FY   +  N     N      A     ++T   PQ+  FG I  FD
+PI+
Sbjct  40   KTSKKPKKLDFYMFIVPQNDTFLDNPKGQYTAVRSAAAVT---
PQAFAFGTIHTFDNPIS  96

Query  85   LSHSLS--
SKQVGRAQGFYIYDTKNTYTSWLSFTFVLNSTHHQGTITFAGADPIVAKTRD  142
             + SL+  S  +GR QG+Y    ++  T  L+ TF  +  H +GT +  GAD
+
Sbjct  97
KAMSLANPSDVLGRVQGWYGNCGQHELTLCLAQTFTYDDGHFKGTFSLMGADIANDPLKY  156

Query  143  ISVTGGTGDFFMHRGIA  159
              + GGTG F   RG+A
Sbjct  157  APIVGGTGHFAFCRGVA  173



Lambda      K        H        a         alpha
```

```
   0.324    0.138    0.422    0.792     4.96

Gapped
Lambda      K        H        a         alpha    sigma
   0.267   0.0410    0.140     1.90     42.6     43.6

Effective search space used: 27720




Matrix: BLOSUM62
Gap Penalties: Existence: 11, Extension: 1
Neighboring words threshold: 11
Window for multiple hits: 40





Query= sp|P13447|LAT52_SOLLC_Anther-
specific_protein_LAT52_OS=Solanum_lycopersicum_GN=LAT52_PE=2_SV=1

Length=161

Subject= 133239-212_4_ORF2
>sp|P13447|LAT52_SOLLC_Anther-
specific_protein_LAT52_OS=Solanum_lycopersicum_GN=LAT52_PE=2_SV=1|||
9e-14

Length=331


 Score = 52.4 bits (124),  Expect = 3e-13, Method: Compositional
matrix adjust.
 Identities = 40/141 (28%), Positives = 64/141 (45%), Gaps = 11/141
(8%)

Query  28
DVEGKVYCDTCRVQFETKLSENLEGATVKLQCRNISTEAETFSVEGVTDKDGKYKLTVNG  87
             V G V+CD C     +     L GA V ++C++ ST   T S EG ++  G Y +
+++G
Sbjct  69   SVVGSVFCDQCLNGHRSMWGLPLNGAKVLVECKD-
STGKVTLSKEGSSNMIGAYTISISG  127

Query  88   DHENDICEVTVV-
KSPREDCKESVSGYEKARIECSDNVGIHNAVRFANPLFFMKAESVQG  146
              + D C   ++  SP+  C  +V+G     +E S  +    A+  A  LFF   +
+
Sbjct  128  SPKMDGCSTRLLGTSPQSSC--AVAGVSTRPVELSWKL-
FGMAMYTAEGLFFKPPKPMSF  184

Query  147  CKEALDE------LGLFPLEF  161
             C  A        + L PL F
```

```
Sbjct  185  CPNAATNSSAPAGVSLLPLPF  205



Lambda      K        H        a         alpha
   0.319    0.135    0.397    0.792     4.96

Gapped
Lambda      K        H        a         alpha    sigma
   0.267   0.0410    0.140     1.90     42.6     43.6

Effective search space used: 42504




Query= sp|P13447|LAT52_SOLLC_Anther-
specific_protein_LAT52_OS=Solanum_lycopersicum_GN=LAT52_PE=2_SV=1

Length=161

Subject= 133903-212_4_ORF2
>sp|P13447|LAT52_SOLLC_Anther-
specific_protein_LAT52_OS=Solanum_lycopersicum_GN=LAT52_PE=2_SV=1|||
9e-14

Length=335


 Score = 52.4 bits (124),  Expect = 4e-13, Method: Compositional
matrix adjust.
 Identities = 40/141 (28%), Positives = 64/141 (45%), Gaps = 11/141
(8%)

Query  28
DVEGKVYCDTCRVQFETKLSENLEGATVKLQCRNISTEAETFSVEGVTDKDGKYKLTVNG  87
             V G V+CD C     +     L GA V ++C++ ST   T S EG ++  G Y +
+++G
Sbjct  73   SVVGSVFCDQCLNGHRSMWGLPLNGAKVLVECKD-
STGKVTLSKEGSSNMIGAYTISISG  131

Query  88   DHENDICEVTVV-
KSPREDCKESVSGYEKARIECSDNVGIHNAVRFANPLFFMKAESVQG  146
              + D C   ++  SP+  C  +V+G     +E S  +    A+  A  LFF   +
+
Sbjct  132  SPKMDGCSTRLLGTSPQSSC--AVAGVSTRPVELSWKL-
FGMAMYTAEGLFFKPPKPMSF  188

Query  147  CKEALDE------LGLFPLEF  161
            C  A         + L PL F
Sbjct  189  CPNAATNSSAPAGVSLLPLPF  209
```

```
Lambda      K        H        a         alpha
   0.319    0.135    0.397    0.792     4.96

Gapped
Lambda      K        H        a         alpha    sigma
   0.267   0.0410    0.140     1.90     42.6     43.6

Effective search space used: 42504
```

```
Matrix: BLOSUM62
Gap Penalties: Existence: 11, Extension: 1
Neighboring words threshold: 11
Window for multiple hits: 40
```

```
Query= sp|P13853|
HS17C_ARATH_17.6_kDa_class_I_heat_shock_protein_3_OS=Arabi
dopsis_thaliana_GN=HSP17.6C_PE=2_SV=2

Length=157

Subject= 115180-231_5_ORF2
>sp|P13853|HS17C_ARATH_17.6_kDa_class_I_heat_shock_protein_3_OS=Arab
idopsis_thaliana_GN=HSP17.6C_PE=2_SV=2|||3e-60

Length=163


 Score =   175 bits (443),  Expect = 3e-60, Method: Compositional
matrix adjust.
 Identities = 91/159 (57%), Positives = 119/159 (75%), Gaps = 8/159
(5%)

Query  1    MSLIPSIFGGRRTNVFDPFSLDVFDPFEGFLTPSGLAN--
APAMDVAAFTNAKVDWRETP  58
            M+L P I GG  +++FDPF+ D F  F G  +  G  +  + A DV A  N +VDW
+ETP
Sbjct  7    MALAPFILGG--SDIFDPFNFDSF-
LFGGRSSGGGKDDRSSYARDVVAVANTQVDWKETP  63

Query  59   EAHVFKADLPGLRKEEVKVEVEDGNILQISGERSN-
ENEEKNDKWHRVERSSGKFTRRFR  117
            EAH+FKA+LPGL KE+VKV+VEDG++LQI GER   E + + DKWHRVER+ G F
RRFR
Sbjct  64
EAHIFKANLPGLSKEDVKVQVEDGHVLQICGERKKEETKSEGDKWHRVERAQGSFLRRFR  123

Query  118  LPENAKMEEIKASMENGVLSVTVPKVPEKKPEVKSIDIS  156
```

```
            LP+NAK+EE+KA+MENGVL+V VPK  E+KP  ++IDI+
Sbjct  124  LPDNAKVEEVKATMENGVLTVAVPK--EQKPAPRTIDIA  160



Lambda      K        H        a         alpha
   0.313    0.133    0.381    0.792     4.96

Gapped
Lambda      K        H        a         alpha    sigma
   0.267   0.0410    0.140     1.90     42.6     43.6

Effective search space used: 20440




Query= sp|P13853|
HS17C_ARATH_17.6_kDa_class_I_heat_shock_protein_3_OS=Arabi
dopsis_thaliana_GN=HSP17.6C_PE=2_SV=2

Length=157

Subject= 178788-157_5_ORF2
>sp|P13853|HS17C_ARATH_17.6_kDa_class_I_heat_shock_protein_3_OS=Arab
idopsis_thaliana_GN=HSP17.6C_PE=2_SV=2|||3e-58

Length=155


 Score =   169 bits (428),  Expect = 3e-58, Method: Compositional
matrix adjust.
 Identities = 85/151 (56%), Positives = 111/151 (74%), Gaps = 7/151
(5%)

Query  14   NVFDPFSLDVFDPFEGFLTPSGLANAP-----
AMDVAAFTNAKVDWRETPEAHVFKADLP  68
            ++F+  S D F+ F+    PS L   P     A DVAA  N +VDW+ET ++H
+FKA+LP
Sbjct  5
SLFNRRSDDPFELFDSLAFPSSLMRFPEHPSFARDVAAVANTQVDWKETSDSHIFKANLP  64

Query  69
GLRKEEVKVEVEDGNILQISGERSNENEEKNDKWHRVERSSGKFTRRFRLPENAKMEEIK  128
            GL KE+VKV+VEDG++LQISGER  E    +D+WHRVER+ G F RRFRLP+NAK
+EE+K
Sbjct  65
GLAKEDVKVQVEDGHVLQISGERKKEETTSSDRWHRVERAQGSFLRRFRLPDNAKVEEVK  124

Query  129  ASMENGVLSVTVPKV--PEKKPEVKSIDISG  157
            A+MENGVL++TVPK   PE K   +SID+SG
Sbjct  125  AAMENGVLTITVPKASPPEPKAAKRSIDVSG  155
```

```
Lambda      K        H        a         alpha
   0.313    0.133    0.381    0.792     4.96

Gapped
Lambda      K        H        a         alpha    sigma
   0.267   0.0410    0.140     1.90     42.6     43.6

Effective search space used: 20440



Query= sp|P13853|
HS17C_ARATH_17.6_kDa_class_I_heat_shock_protein_3_OS=Arabi
dopsis_thaliana_GN=HSP17.6C_PE=2_SV=2

Length=157

Subject= 22356-529_5_ORF1
>sp|P13853|HS17C_ARATH_17.6_kDa_class_I_heat_shock_protein_3_OS=Arab
idopsis_thaliana_GN=HSP17.6C_PE=2_SV=2|||7e-39

Length=85


 Score =   116 bits (291),  Expect = 7e-39, Method: Compositional
matrix adjust.
 Identities = 56/85 (66%), Positives = 69/85 (81%), Gaps = 2/85 (2%)

Query  75
VKVEVEDGNILQISGERSNENEEKNDKWHRVERSSGKFTRRFRLPENAKMEEIKASMENG  134
            VKV+VEDG +LQISGER  E    +D+WHRVER+ G F RRFRLP+NAK++E+KA
+MENG
Sbjct  1
VKVQVEDGRVLQISGERKKEGTSSSDRWHRVERAQGSFLRRFRLPDNAKVDEVKAAMENG  60

Query  135  VLSVTVPKV--PEKKPEVKSIDISG  157
            VL+VTVPK   PE K  V++IDISG
Sbjct  61   VLTVTVPKTAPPEPKAVVRTIDISG  85



Lambda      K        H        a         alpha
   0.313    0.133    0.381    0.792     4.96

Gapped
Lambda      K        H        a         alpha    sigma
   0.267   0.0410    0.140     1.90     42.6     43.6

Effective search space used: 20440
```

```
Matrix: BLOSUM62
Gap Penalties: Existence: 11, Extension: 1
Neighboring words threshold: 11
Window for multiple hits: 40





Query= sp|P13934|
LEA76_BRANA_Late_embryogenesis_abundant_protein_76_OS=Bras
sica_napus_PE=2_SV=2

Length=280

Subject= 249759-100_3_ORF2
>sp|P13934|LEA76_BRANA_Late_embryogenesis_abundant_protein_76_OS=Bra
ssica_napus_PE=2_SV=2|||4e-09

Length=100


 Score = 30.4 bits (67),  Expect = 3e-06, Method: Compositional
matrix adjust.
 Identities = 31/81 (38%), Positives = 42/81 (52%), Gaps = 4/81 (5%)

Query  81
DKTSQAAQTTQQKAQETAQAAKDKTSQAAQTTQQKAHETTQSSKEKTSQAAQTAQEKARE  140
            D+   A  TT  KA+ET Q  + K  +A    Q +A   T ++KEK   AA
QEKA +
Sbjct  16   DQAQHAKDTTAHKAEETKQYGQQKAGEA----
QHQAQGITGAAKEKAEGAAHATQEKATQ  71

Query  141  TKDKTGSYLSETGEAVKQKAQ  161
             KD  G    + G+A+K  AQ
Sbjct  72   AKDGVGHAFQQAGDAIKGAAQ  92


 Score = 18.5 bits (36),  Expect = 0.036, Method: Compositional
matrix adjust.
 Identities = 10/27 (37%), Positives = 17/27 (63%), Gaps = 0/27 (0%)

Query  28  MRDKAEEGKDKTSQTAQKAQQKAQETA  54
           M D+A+  KD T+  A++ +Q  Q+ A
Sbjct  14  MADQAQHAKDTTAHKAEETKQYGQQKA  40


 Score = 17.3 bits (33),  Expect = 0.11, Method: Compositional
matrix adjust.
 Identities = 26/58 (45%), Positives = 31/58 (53%), Gaps = 4/58 (7%)

Query  31
KAEEGKDKTSQTAQKAQQKAQETAQAAKDKTSQAAQTTQQKAQETAQAAKDKTSQAAQ  88
```

```
           KAEE K    Q A +AQ +AQ    AAK+K   AA  TQ+KA +    AKD    A
Q
Sbjct  28  KAEETKQYGQQKAGEAQHQAQGITGAAKEKAEGAAHATQEKATQ----
AKDGVGHAFQ  81


 Score = 15.0 bits (27),  Expect = 0.56, Method: Compositional
matrix adjust.
 Identities = 8/18 (44%), Positives = 13/18 (72%), Gaps = 0/18 (0%)

Query  7   SYKAGETRGKTQEKTGQA  24
           ++KA ET+   Q+K G+A
Sbjct  26  AHKAEETKQYGQQKAGEA  43


 Score = 14.6 bits (26),  Expect = 0.85, Method: Compositional
matrix adjust.
 Identities = 11/28 (39%), Positives = 14/28 (50%), Gaps = 0/28 (0%)

Query  174  AAQYTKETAEAGKDKTGGFLSQTGEHVK  201
            AA  T+E A   KD  G    Q G+ +K
Sbjct  61   AAHATQEKATQAKDGVGHAFQQAGDAIK  88


 Score = 12.3 bits (20),  Expect = 4.3, Method: Compositional matrix
adjust.
 Identities = 10/29 (34%), Positives = 13/29 (45%), Gaps = 0/29 (0%)

Query  17  TQEKTGQAMGAMRDKAEEGKDKTSQTAQK  45
           TQEK  QA   +    ++  D     AQK
Sbjct  65  TQEKATQAKDGVGHAFQQAGDAIKGAAQK  93


 Score = 11.9 bits (19),  Expect = 5.5, Method: Compositional matrix
adjust.
 Identities = 4/7 (57%), Positives = 6/7 (86%), Gaps = 0/7 (0%)

Query  175  AQYTKET  181
            AQ+ K+T
Sbjct  18   AQHAKDT  24



Lambda      K        H        a         alpha
   0.302    0.111    0.290    0.792     4.96

Gapped
Lambda      K        H        a         alpha    sigma
   0.267   0.0410    0.140     1.90     42.6     43.6

Effective search space used: 21484
```

Matrix: BLOSUM62
Gap Penalties: Existence: 11, Extension: 1
Neighboring words threshold: 11
Window for multiple hits: 40

Query= sp|P20075|LEAD8_DAUCA_Embryonic_protein_DC-
8_OS=Daucus_carota_PE=3_SV=1

Length=555

Subject= 253001-98_1_ORF1
>sp|P20075|LEAD8_DAUCA_Embryonic_protein_DC-
8_OS=Daucus_carota_PE=3_SV=1|||1e-12

Length=136

Score = 47.0 bits (110), Expect = 4e-11, Method: Compositional
matrix adjust.
Identities = 49/123 (40%), Positives = 63/123 (51%), Gaps = 11/123
(9%)

```
Query  304
KDYSAQKAAETKDATMEKTKEYKDYTAQKAAETKDATMEKAKEAKDTTVQKTGEYKDYAA  363
            KD +A KA ETK    EKT E KD TA KA E +     +A++AKDTT  K  E K
Y
Sbjct  16   KDTTAHKAEETKQYGQEKTGEAKDATAHKAGEAQ----
HQAQQAKDTTAHKAEETKQYGQ  71

Query  364  EKAKEGKD----VT---
VEKAKEGKDTTVGKMTELKDSAADAARKAMDMFLGKKEEVKGK  416
            +KA E +     +T    EKA+     T  K T+ KD    A ++A D   G  +
+V G
Sbjct  72
QKAGEAQHQAQGITGAAKEKAEGAAHATQEKATQAKDGVGHAFQQAGDAIKGAAQKVTGG  131

Query  417  AGE  419
             GE
Sbjct  132  GGE  134
```

Score = 44.3 bits (103), Expect = 3e-10, Method: Compositional
matrix adjust.
Identities = 42/91 (46%), Positives = 50/91 (55%), Gaps = 18/91
(20%)

```
Query  130
AQKAEEAKEKAAQKAEETKEKAGEYKNYTAQKAGEAKDTTLGKAGEYKDYAAQKAAEAKD  189
            A  A+ AK+  A KAEETK+       Y  +K GEAKD T  KAGE    A  +A
```

```
+AKD
Sbjct  9    ADHAQHAKDTTAHKAEETKQ-------YGQEKTGEAKDATAHKAGE----
AQHQAQQAKD  57

Query  190  TTAQKAAEAKEKTGEYKDYAAQKAAEAKVLA  220
            TTA KA E K+       Y  QKA EA+  A
Sbjct  58   TTAHKAEETKQ-------YGQQKAGEAQHQA  81


 Score = 42.0 bits (97),  Expect = 2e-09, Method: Compositional
matrix adjust.
 Identities = 45/108 (42%), Positives = 51/108 (47%), Gaps = 8/108
(7%)

Query  242
AQKAAEAKDATMQKTGEYKDYAAQKTAETKDATMEKAKEYKEYAAQKAAEAKDATMQKTG  301
            A  A  AKD T  K  E K Y  +KT E KDAT  KA E    A  +A +AKD T
K
Sbjct  9    ADHAQHAKDTTAHKAEETKQYGQEKTGEAKDATAHKAGE----
AQHQAQQAKDTTAHKAE  64

Query  302  EYKDYSAQKAAETKDATMEKTKEYKDYTAQKAAETKDATMEKAKEAKD  349
            E K Y  QKA E +      T        +KA     AT EKA +AKD
Sbjct  65   ETKQYGQQKAGEAQHQAQGITGA----AKEKAEGAAHATQEKATQAKD  108


 Score = 40.8 bits (94),  Expect = 4e-09, Method: Compositional
matrix adjust.
 Identities = 40/74 (54%), Positives = 42/74 (57%), Gaps = 9/74
(12%)

Query  181  AQKAAEAKDTTAQKAAEAK----
EKTGEYKDYAAQKAAEAKVLAAQKAAEAKDTTG-KDG  235
            A  A  AKDTTA KA E K    EKTGE KD  A KA EA+  A Q    AKDTT
K
Sbjct  9    ADHAQHAKDTTAHKAEETKQYGQEKTGEAKDATAHKAGEAQHQAQQ----
AKDTTAHKAE  64

Query  236  EYKDYAAQKAAEAK  249
            E K Y  QKA EA+
Sbjct  65   ETKQYGQQKAGEAQ  78


 Score = 38.5 bits (88),  Expect = 3e-08, Method: Compositional
matrix adjust.
 Identities = 38/92 (41%), Positives = 46/92 (50%), Gaps = 11/92
(12%)

Query  80
EVSRENTDYAYDKGREGGDVAAQKAEEAKEKAKMAKDTTMGKAGEYKDYTAQKAEEAKEK  139
            E  RE  D+A    +   D  A KAEE K+  +        K GE KD TA KA
EA+ +
Sbjct  3    EREREMADHA----QHAKDTTAHKAEETKQYGQE-------
KTGEAKDATAHKAGEAQHQ  51
```

```
Query  140  AAQKAEETKEKAGEYKNYTAQKAGEAKDTTLG  171
            A Q  + T  KA E K Y  QKAGEA+    G
Sbjct  52   AQQAKDTTAHKAEETKQYGQQKAGEAQHQAQG  83


 Score = 38.5 bits (88),  Expect = 3e-08, Method: Compositional
matrix adjust.
 Identities = 29/63 (46%), Positives = 36/63 (57%), Gaps = 0/63 (0%)

Query  78
TAEVSRENTDYAYDKGREGGDVAAQKAEEAKEKAKMAKDTTMGKAGEYKDYTAQKAEEAK  137
            TA  + E   Y  +K  E  D  A KA EA+ +A+ AKDTT  KA E K Y  QKA
EA+
Sbjct  19
TAHKAEETKQYGQEKTGEAKDATAHKAGEAQHQAQQAKDTTAHKAEETKQYGQQKAGEAQ  78

Query  138  EKA  140
             +A
Sbjct  79   HQA  81


 Score = 36.6 bits (83),  Expect = 1e-07, Method: Compositional
matrix adjust.
 Identities = 41/104 (39%), Positives = 48/104 (46%), Gaps = 23/104
(22%)

Query  221  AQKAAEAKDTTG-
KDGEYKDYAAQKAAEAKDATMQKTGEYKDYAAQKTAETKDATMEKAK  279
            A  A  AKDTT  K  E K Y  +K  EAKDAT  K GE +  A Q     KD T
KA+
Sbjct  9    ADHAQHAKDTTAHKAEETKQYGQEKTGEAKDATAHKAGEAQHQAQQ----
AKDTTAHKAE  64

Query  280  EYKEYAAQKAAEAKD------------------ATMQKTGEYKD  305
            E K+Y  QKA EA+                   AT +K  + KD
Sbjct  65   ETKQYGQQKAGEAQHQAQGITGAAKEKAEGAAHATQEKATQAKD  108


 Score = 31.6 bits (70),  Expect = 5e-06, Method: Compositional
matrix adjust.
 Identities = 29/73 (40%), Positives = 38/73 (52%), Gaps = 8/73
(11%)

Query  320
EKTKEYKDYTAQKAAETKDATMEKAKEAKDTTVQKTGEYKDYAAEKAKEGKDVTVEKAKE  379
            E+ +E  D+    A   KD T  KA+E K    +KTGE KD  A KA E +
+A++
Sbjct  3    EREREMADH----AQHAKDTTAHKAEETKQYGQEKTGEAKDATAHKAGEAQ----
HQAQQ  54

Query  380  GKDTTVGKMTELK  392
             KDTT  K  E K
Sbjct  55   AKDTTAHKAEETK  67
```

```
 Score = 29.6 bits (65),  Expect = 3e-05, Method: Compositional
matrix adjust.
 Identities = 32/88 (36%), Positives = 37/88 (42%), Gaps = 25/88
(28%)

Query  206
KDYAAQKAAEAKVLAAQKAAEAKDTTGKDGEYKDYAAQKAAEAKDATMQKTGEYKDYAAQ  265
            KD  A KA E K    +K  EAKD T          A KA EA+    Q
Sbjct  16   KDTTAHKAEETKQYGQEKTGEAKDAT----------
AHKAGEAQHQAQQ-----------  54

Query  266  KTAETKDATMEKAKEYKEYAAQKAAEAK  293
                 KD T  KA+E K+Y  QKA EA+
Sbjct  55   ----AKDTTAHKAEETKQYGQQKAGEAQ  78


 Score = 29.3 bits (64),  Expect = 4e-05, Method: Compositional
matrix adjust.
 Identities = 27/71 (38%), Positives = 30/71 (42%), Gaps = 15/71
(21%)

Query  344
AKEAKDTTVQKTGEYKDYAAEKAKEGKDVTVEKAKEGKDTTVGKMTELKDSAADAARKAM  403
            A+ AKDTT  K  E K Y  EK  E KD T  KA E               A   A
++A
Sbjct  12   AQHAKDTTAHKAEETKQYGQEKTGEAKDATAHKAGE---------------
AQHQAQQAK  56

Query  404  DMFLGKKEEVK  414
            D    K EE K
Sbjct  57   DTTAHKAEETK  67


 Score = 19.2 bits (38),  Expect = 0.069, Method: Compositional
matrix adjust.
 Identities = 31/115 (27%), Positives = 52/115 (45%), Gaps = 0/115
(0%)

Query  374
VEKAKEGKDTTVGKMTELKDSAADAARKAMDMFLGKKEEVKGKAGETAEAAKEKYEDTEF  433
             + A+  KDTT  K  E K    +   +A D    K  E + +A +  +    K E
+T+
Sbjct  9
ADHAQHAKDTTAHKAEETKQYGQEKTGEAKDATAHKAGEAQHQAQQAKDTTAHKAEETKQ  68

Query  434  AARKKMEELKLQEEGVKDEAKQRAEADRETAGDRGSAAKGTIFGAMGSVKDAIVG
488
              ++K  E + Q +G+   AK++AE       ++ + AK  +  A     DAI G
Sbjct  69   YGQQKAGEAQHQAQGITGAAKEKAEGAAHATQEKATQAKDGVGHAFQQAGDAIKG
123
```

```
 Score = 13.5 bits (23),  Expect = 5.3, Method: Compositional matrix
adjust.
 Identities = 14/49 (29%), Positives = 23/49 (47%), Gaps = 0/49 (0%)

Query  64   LGQAKEVVVGKAHDTAEVSRENTDYAYDKGREGGDVAAQKAEEAKEKAK  112
             G+AK+    KA +    +++  D    K  E      QKA EA+ +A+
Sbjct  34   TGEAKDATAHKAGEAQHQAQQAKDTTAHKAEETKQYGQQKAGEAQHQAQ  82



Lambda      K        H        a         alpha
   0.300    0.117    0.296    0.792     4.96

Gapped
Lambda      K        H        a         alpha    sigma
   0.267   0.0410    0.140     1.90     42.6     43.6

Effective search space used: 58830




Query= sp|P20075|LEAD8_DAUCA_Embryonic_protein_DC-
8_OS=Daucus_carota_PE=3_SV=1

Length=555

Subject= 300346-68_4_ORF1
>sp|P20075|LEAD8_DAUCA_Embryonic_protein_DC-
8_OS=Daucus_carota_PE=3_SV=1|||3e-20

Length=583


 Score = 86.3 bits (212),  Expect = 5e-22, Method: Compositional
matrix adjust.
 Identities = 120/366 (33%), Positives = 170/366 (46%), Gaps =
62/366 (17%)

Query  100
AAQKAEEAKEKAKMAKDTTMGKAGEYKDYTAQKAEEAKEKAAQKAEETKEKAGEYKNYTA  159
            A +  E++K  A  A D     AG  KD  A KA++AK       E  +EKA +Y
N
Sbjct  44   AHESIEQSKGSAARAGDDARDTAGHVKDAAASKAQDAK----
GGLEAGQEKASQYAN---  96

Query  160
QKAGEAKDTTLGKAGEYKDYAAQKAAEAKDTTAQKAAEAKEKTGEYKDYAAQKAAEAKVL  219
                 A++T +G           KA  AK  T   A +AKEK GE K+ A Q+
K
Sbjct  97   ----AARETVVG-----------
KAESAKGKTEDVAHQAKEKVGETKEGAQQRTGGIKEG  141

Query  220  AAQKAAEAKDTTGKDGEYKDYAAQKAAEAKDATMQKTGE-----------
```

YKDYAAQKTA 268
            A QKA + +   Q A E+KDA +KT E   + D
KT
Sbjct 142 AQQKAGDVQ-------------
QSATESKDAAKEKTSEGVEQSKGYFQSFTDALYGKTQ 187

Query 269 ETKDATMEKAKEYKEYAAQKAAEAKDA---TMQKTGE-
YKDYSAQKAAETKDATMEKTKE 324
              KD+T + A++ K+   A +AKD   T+Q   + KD +   A   KDAT +
+
Sbjct 188
AVKDSTAQTAQQGKDTTYDTATKAKDTTYDTVQSAAQTVKDTTYNTAQRAKDATYDTVQR 247

Query 325
YKDYTAQKAAETKDATMEKAKEAKDTTVQKTGEYKDYAAEKAKEGKDVT----------- 373
             KD T   A   KDAT + A+ AKDTT   KD   + A+ KD T
Sbjct 248
AKDVTYDTAQRAKDATFDAAQRAKDTTYDAAQRAKDSTYDTAQRTKDTTYDTTMQTKDRA 307

Query 374
VEKAKEGKDTTVGKMTELKDSAADAARKAMDMFLGKKEEVKGKAGETAEAAKEKYEDTEF 433
            V+ +++GK++T G + + D+ ++ A   + LGK EE KGKA ETA+ AKE+
+T
Sbjct 308
VDYSQQGKESTKGYLQSVTDTISERAGALKETLLGKTEEAKGKANETAQQAKERSYETSD 367

Query 434 AARKKM 439
            AA++K
Sbjct 368 AAKEKT 373

Score = 53.1 bits (126), Expect = 1e-11, Method: Compositional
matrix adjust.
Identities = 59/171 (35%), Positives = 87/171 (51%), Gaps = 20/171
(12%)

Query 76   HDTAEVSRENTDYAYDKGREGGDVAAQKAEEAKEKAK----
MAKDTTMGKAGEYKDYTAQ 131
            +DTA+ +++ T   YD   + D A   +++ KE K   DT   +AG K+
Sbjct 286 YDTAQRTKDTT---
YDTTMQTKDRAVDYSQQGKESTKGYLQSVTDTISERAGALKETLLG 342

Query 132
KAEEAKEKAAQKAEETKEKAGEYKNYTAQKAGEAKDTTLGKAGEYKDYAAQKAAEAKDTT 191
            K EEAK KA + A++ KE++ E +   +K G KDT +GK EYK+ AA
+AKD T
Sbjct 343
KTEEAKGKANETAQQAKERSYETSDAAKEKTGATKDTVMGKTEEYKNAAADTLNQAKDAT 402

Query 192 AQKAAEAKEKTGEYKDYAAQKAAEAKVLAAQ-----KAAEAKDT---TGKD 234
             + A + K+KT   Y   + A K++   AAEA D+   TGKD
Sbjct 403 YETAVQGKDKT-----YGTIEEARQKIMGKAEEYKGSAAEATDSSKETGKD 448

```
 Score = 52.4 bits (124),  Expect = 3e-11, Method: Compositional
matrix adjust.
 Identities = 90/305 (30%), Positives = 139/305 (46%), Gaps = 25/305
(8%)

Query  186  EAKDTTAQKAAEAKEKTGEYKDYAAQKAAEAK--
VLAAQKAAEAKDTTGKDGEYKDYAAQ  243
            ++K + A+   +A++  G  KD AA KA +AK  + A Q+ A          +Y +
A +
Sbjct  50   QSKGSAARAGDDARDTAGHVKDAAASKAQDAKGGLEAGQEKAS---------
QYANAARE  100

Query  244  KAAEAKDATMQKTGEYKDYAAQKTAETKDATMEKAKEYKEYAAQKAA-------
EAKDAT  296
                  ++   KT +    A +K  ETK+   ++    KE A QKA        E
+KDA
Sbjct  101
TVVGKAESAKGKTEDVAHQAKEKVGETKEGAQQRTGGIKEGAQQKAGDVQQSATESKDAA  160

Query  297
MQKTGEYKDYSAQKAAETKDATMEKTKEYKDYTAQKAAETKDATMEKAKEAKDT---TVQ  353
             +KT E  + S        DA   KT+  KD TAQ A + KD T + A +AKDT
TVQ
Sbjct  161
KEKTSEGVEQSKGYFQSFTDALYGKTQAVKDSTAQTAQQGKDTTYDTATKAKDTTYDTVQ  220

Query  354  KTGE-
YKDYAAEKAKEGKDVTVEKAKEGKDTTVGKMTELKDSAADAARKAMDMFLGKKEE  412
               +  KD     A+  KD T +  +  KD T       KD+  DAA++A D
+
Sbjct  221
SAAQTVKDTTYNTAQRAKDATYDTVQRAKDVTYDTAQRAKDATFDAAQRAKDTTYDAAQR  280

Query  413
VKGKAGETAEAAKEKYEDTEFAARKKMEELKLQEEGVKDEAKQRAEADRETAGDRGSAAK  472
             K    +TA+  K+   DT    + +   +   ++G K+  K   ++  +T  +R
A K
Sbjct  281  AKDSTYDTAQRTKDTTYDTTMQTKDRA--VDYSQQG-
KESTKGYLQSVTDTISERAGALK  337

Query  473  GTIFG  477
             T+ G
Sbjct  338  ETLLG  342


 Score = 47.4 bits (111),  Expect = 8e-10, Method: Compositional
matrix adjust.
 Identities = 123/433 (28%), Positives = 186/433 (43%), Gaps =
61/433 (14%)

Query  86   TDYAYDKGREGGDVAAQKAEEAK----EKAKMAKDTT----MGKAGEYKD---
YTAQKAE  134
            TD  Y K +   D  AQ A++ K    + A  AKDTT       A   KD
TAQ+A+
```

```
Sbjct  179
TDALYGKTQAVKDSTAQTAQQGKDTTYDTATKAKDTTYDTVQSAAQTVKDTTYNTAQRAK  238

Query  135  EAKEKAAQKAEE-
TKEKAGEYKNYTAQKAGEAKDTTLGKAGEYKDYAAQKAAEAKDTTAQ  193
            +A     Q+A++ T + A   K+ T   A  AKDTT   A   KD     A
KDTT
Sbjct  239
DATYDTVQRAKDVTYDTAQRAKDATFDAAQRAKDTTYDAAQRAKDSTYDTAQRTKDTTYD  298

Query  194  KAAEAKEKTGEYKDYAAQKAAEAKV--------LAAQKAAEAKDT-
TGKDGEYKDYAAQK  244
               + K++  +Y    +Q+  E+            +++A   K+T  GK  E K
A +
Sbjct  299  TTMQTKDRAVDY----
SQQGKESTKGYLQSVTDTISERAGALKETLLGKTEEAKGKANET  354

Query  245
AAEAKDATMQKTGEYKDYAAQKTAETKDATMEKAKEYKEYAAQKAAEAKDATMQKTGEYK  304
            A +AK    +++ E  D A +KT  TKD  M K +EYK  AA    +AKDAT +
+ K
Sbjct  355  AQQAK----
ERSYETSDAAKEKTGATKDTVMGKTEEYKNAAADTLNQAKDATYETAVQGK  410

Query  305
DYSAQKAAETKDATMEKTKEYKDYTAQKAAETKDATMEKAKEAKDTTVQKTGEYKDYAAE  364
            D +     E +   M K +EYK      AAE  D++ E  K+  D       +
A +
Sbjct  411  DKTYGTIEEARQKIMGKAEEYKG----
SAAEATDSSKETGKDLTDRAKGTGRDVSQRAQD  466

Query  365
KAKEGKDVTVEKAKEGKDTTVGKMTELKDSAADAARKAMDMFLGKKEEVKGKAGETAEAA  424
             A++ K+   EK +E K    G +  + +  +  A      + GK EE + K  +
+ A
Sbjct  467  SAQQAKETAGEKKEESK----
GYLQTVGEKVSQTAASLKGTWAGKTEEAQEKGQQATDDA  522

Query  425  KEKYEDTEF--------
AARKKMEELKLQEEGVKDEAKQRAEADRETAGDRGSAAKGTIF  476
            +++  D +         AA +KM E   + EG         EA
Sbjct  523
QQRLRDLKLKDEKEGGPAAGQKMREAGKKVEGAAHRGADEVEAK---------------  566

Query  477  GAMGSVKDAIVGK  489
            G   S++D ++GK
Sbjct  567  GVFQSLRDTVLGK  579
```

Score = 36.2 bits (82), Expect = 2e-06, Method: Compositional matrix adjust.
Identities = 44/147 (30%), Positives = 69/147 (47%), Gaps = 28/147 (19%)

```
Query  51   GVIGSILKSVQGTLGQAKEVVVGK-------
AHDTAEVSRENTDYAYDKGREGGDVAAQK  103
            G + S+  ++    G  KE ++GK       A++TA+ ++E
+ +
Sbjct  320  GYLQSVTDTISERAGALKETLLGKTEEAKGKANETAQQAKER--------------
SYET  365

Query  104
AEEAKEKAKMAKDTTMGKAGEYKDYTAQKAEEAKEKAAQKAEETKEKAGEYKNYTAQKAG  163
            ++ AKEK    KDT MGK  EYK+  A    +AK+        T E A + K+ T
Sbjct  366  SDAAKEKTGATKDTVMGKTEEYKNAAADTLNQAKDA-------
TYETAVQGKDKTYGTIE  418

Query  164  EAKDTTLGKAGEYKDYAAQKAAEAKDT  190
            EA+   +GKA EYK  AA+    +K+T
Sbjct  419  EARQKIMGKAEEYKGSAAEATDSSKET  445


 Score = 25.8 bits (55),  Expect = 0.004, Method: Compositional
matrix adjust.
 Identities = 45/157 (29%), Positives = 69/157 (44%), Gaps = 33/157
(21%)

Query  356
GEYKDYAAEKAKEGKDVTVEKAKEGKDTTVGKMTELKDSAADAARKAMDMFLGKKEEVKG  415
            G  KD AA KA++ K               G +   ++ A+  A  A +  +GK E
KG
Sbjct  67   GHVKDAAASKAQDAK---------------
GGLEAGQEKASQYANAARETVVGKAESAKG  111

Query  416
KAGETAEAAKEKYEDTEFAARKKMEELKLQEEGVKDEAKQRAEADRETAGDRGSAAKGTI  475
            K  + A  AKEK  +T+  A+++         G+K+ A+Q+A   +++A +
AAK
Sbjct  112  KTEDVAHQAKEKVGETKEGAQQRT-------
GGIKEGAQQKAGDVQQSATESKDAAKEKT  164

Query  476  F-------GAMGSVKDAIVGKLTMPSDVVKDKQQQEA  505
                    G   S  DA+ GK    +  VKD   Q A
Sbjct  165  SEGVEQSKGYFQSFTDALYGK----TQAVKDSTAQTA  197


 Score = 22.3 bits (46),  Expect = 0.053, Method: Compositional
matrix adjust.
 Identities = 29/100 (29%), Positives = 47/100 (47%), Gaps = 11/100
(11%)

Query  62   GTLGQAKEVVVGKAHD-----------
TAEVSRENTDYAYDKGREGGDVAAQKAEEAKEK  110
            GT+ +A++ ++GKA +           + E  ++ TD A   GR+    A   A+
+AKE
Sbjct  415
GTIEEARQKIMGKAEEYKGSAAEATDSSKETGKDLTDRAKGTGRDVSQRAQDSAQQAKET  474
```

```
Query  111  AKMAKDTTMGKAGEYKDYTAQKAEEAKEKAAQKAEETKEK  150
            A   K+ + G      +  +Q A   K   A K EE +EK
Sbjct  475  AGEKKEESKGYLQTVGEKVSQTAASLKGTWAGKTEEAQEK  514



Lambda      K        H        a         alpha
   0.300    0.117    0.296    0.792     4.96

Gapped
Lambda      K        H        a         alpha    sigma
   0.267   0.0410    0.140     1.90     42.6     43.6

Effective search space used: 58830




Query= sp|P20075|LEAD8_DAUCA_Embryonic_protein_DC-
8_OS=Daucus_carota_PE=3_SV=1

Length=555

Subject= 306456-65_2_ORF1
>sp|P20075|LEAD8_DAUCA_Embryonic_protein_DC-
8_OS=Daucus_carota_PE=3_SV=1|||2e-20

Length=594


 Score = 88.2 bits (217),  Expect = 1e-22, Method: Compositional
matrix adjust.
 Identities = 120/365 (33%), Positives = 173/365 (47%), Gaps =
62/365 (17%)

Query  100
AAQKAEEAKEKAKMAKDTTMGKAGEYKDYTAQKAEEAKEKAAQKAEETKEKAGEYKNYTA  159
            A +  +++K  A  A D     AG  KD  A KA++AK       E  +EKA +Y
N
Sbjct  44   AHESIQQSKSSAARAGDDARDTAGHVKDAAASKAQDAKGGL----
EAGQEKASQYAN---  96

Query  160
QKAGEAKDTTLGKAGEYKDYAAQKAAEAKDTTAQKAAEAKEKTGEYKDYAAQKAAEAKVL  219
                 A+DT +GKA         +AA+ K  T   A +AKEK GE K+ A Q+
K
Sbjct  97   ----AARDTVVGKA---------EAAKGK--
TEDLAHQAKEKVGETKEGAQQRTGGIKEG  141

Query  220  AAQKAAEAKDTTGKDGEYKDYAAQKAAEAKDATMQKTGE-----------
YKDYAAQKTA  268
            A Q+A + +              Q A E+KDA  +KT E           + D
KT
Sbjct  142  AQQRAGDVQ--------------
```

```
QSATESKDAAKEKTSEGVEQSKGYFQSFTDALYGKTQ  187

Query  269  ETKDATMEKAKEYKEYAAQKAAEAKDA---TMQKTGE-
YKDYSAQKAAETKDATMEKTKE  324
             KD+T + A++ K+     A +AKD    T+Q   +  KD +   A   KDAT +
+
Sbjct  188
AVKDSTAQTAQQGKDTTYDTATKAKDTTYDTVQSAAQTVKDTTYNTAQRAKDATYDTVQR  247

Query  325
YKDYTAQKAAETKDATMEKAKEAKDTTVQKTGEYKDYAAEKAKEGKDVT-----------  373
             KD T   A   KDAT + A+ AKDTT       KD   + A+  KD T
Sbjct  248
AKDVTYDTAQRAKDATFDAAQRAKDTTYDAAQRAKDSTYDTAQRTKDTTYDTTMQTKDRA  307

Query  374
VEKAKEGKDTTVGKMTELKDSAADAARKAMDMFLGKKEEVKGKAGETAEAAKEKYEDTEF  433
            V+ +++GK++T G +  + D+ ++ A    +  LGK EE KGKA ETA+ AKE+
+T
Sbjct  308
VDYSQQGKESTKGYLQSVTDTISERAGALKETLLGKTEEAKGKANETAQQAKERSYETSD  367

Query  434  AARKK  438
            AA++K
Sbjct  368  AAKEK  372


 Score = 53.9 bits (128),  Expect = 8e-12, Method: Compositional
matrix adjust.
 Identities = 93/310 (30%), Positives = 141/310 (45%), Gaps = 35/310
(11%)

Query  186
EAKDTTAQKAAEAKEKTGEYKDYAAQKAAEAKVLAAQKAAEAKDTTGKDGEYKDYAAQKA  245
            ++K + A+   +A++  G  KD AA KA +AK     +A + K            A
+Q A
Sbjct  50   QSKSSAARAGDDARDTAGHVKDAAASKAQDAK--GGLEAGQEK------------
ASQYA  95

Query  246  AEAKDATMQKT----GEYKDYAAQ---
KTAETKDATMEKAKEYKEYAAQKAA-------E  291
             A+D  + K     G+ +D A Q   K  ETK+   ++    KE A Q+A
E
Sbjct  96
NAARDTVVGKAEAAKGKTEDLAHQAKEKVGETKEGAQQRTGGIKEGAQQRAGDVQQSATE  155

Query  292
AKDATMQKTGEYKDYSAQKAAETKDATMEKTKEYKDYTAQKAAETKDATMEKAKEAKDT-  350
            +KDA  +KT E  + S        DA   KT+  KD TAQ A + KD T + A
+AKDT
Sbjct  156
SKDAAKEKTSEGVEQSKGYFQSFTDALYGKTQAVKDSTAQTAQQGKDTTYDTATKAKDTT  215

Query  351  --TVQKTGE-
```

```
YKDYAAEKAKEGKDVTVEKAKEGKDTTVGKMTELKDSAADAARKAMDMFL  407
              TVQ   +  KD     A+  KD T +  +  KD T       KD+  DAA++A
D
Sbjct  216
YDTVQSAAQTVKDTTYNTAQRAKDATYDTVQRAKDVTYDTAQRAKDATFDAAQRAKDTTY  275

Query  408
GKKEEVKGKAGETAEAAKEKYEDTEFAARKKMEELKLQEEGVKDEAKQRAEADRETAGDR  467
               +  K    +TA+  K+   DT    + +   +   ++G K+  K   ++  +T
+R
Sbjct  276  DAAQRAKDSTYDTAQRTKDTTYDTTMQTKDRA--VDYSQQG-
KESTKGYLQSVTDTISER  332

Query  468  GSAAKGTIFG  477
              A K T+ G
Sbjct  333  AGALKETLLG  342


 Score = 53.5 bits (127),  Expect = 1e-11, Method: Compositional
matrix adjust.
 Identities = 59/171 (35%), Positives = 87/171 (51%), Gaps = 20/171
(12%)

Query  76   HDTAEVSRENTDYAYDKGREGGDVAAQKAEEAKEKAK----
MAKDTTMGKAGEYKDYTAQ  131
            +DTA+ +++ T   YD   +  D A   +++ KE  K       DT   +AG  K+
Sbjct  286  YDTAQRTKDTT---
YDTTMQTKDRAVDYSQQGKESTKGYLQSVTDTISERAGALKETLLG  342

Query  132
KAEEAKEKAAQKAEETKEKAGEYKNYTAQKAGEAKDTTLGKAGEYKDYAAQKAAEAKDTT  191
            K EEAK KA + A++ KE++ E  +   +K G  KDT +GK  EYK+ AA
+AKD T
Sbjct  343
KTEEAKGKANETAQQAKERSYETSDAAKEKTGATKDTVMGKTEEYKNAAADTLNQAKDAT  402

Query  192  AQKAAEAKEKTGEYKDYAAQKAAEAKVLAAQ-----KAAEAKDT---TGKD  234
             + A + K+KT     Y   + A  K++         AAEA D+   TGKD
Sbjct  403  YETAVQGKDKT-----YGTIEEARQKIMGKAEEYKGSAAEATDSSKQTGKD  448


 Score = 46.2 bits (108),  Expect = 2e-09, Method: Compositional
matrix adjust.
 Identities = 128/435 (29%), Positives = 195/435 (45%), Gaps =
54/435 (12%)

Query  86   TDYAYDKGREGGDVAAQKAEEAK----EKAKMAKDTT----MGKAGEYKD---
YTAQKAE  134
            TD  Y K +   D  AQ A++ K    + A  AKDTT       A   KD
TAQ+A+
Sbjct  179
TDALYGKTQAVKDSTAQTAQQGKDTTYDTATKAKDTTYDTVQSAAQTVKDTTYNTAQRAK  238

Query  135  EAKEKAAQKAEE-
```

```
TKEKAGEYKNYTAQKAGEAKDTTLGKAGEYKDYAAQKAAEAKDTTAQ  193
            +A      Q+A++ T + A   K+ T   A  AKDTT   A   KD      A
KDTT
Sbjct  239
DATYDTVQRAKDVTYDTAQRAKDATFDAAQRAKDTTYDAAQRAKDSTYDTAQRTKDTTYD  298

Query  194  KAAEAKEKTGEYKDYAAQKAAEAKV--------LAAQKAAEAKDT-
TGKDGEYKDYAAQK  244
               + K++  +Y    +Q+  E+            +++A   K+T  GK  E K
A +
Sbjct  299  TTMQTKDRAVDY----
SQQGKESTKGYLQSVTDTISERAGALKETLLGKTEEAKGKANET  354

Query  245
AAEAKDATMQKTGEYKDYAAQKTAETKDATMEKAKEYKEYAAQKAAEAKDATMQKTGEYK  304
            A +AK    +++ E  D A +KT  TKD  M K +EYK  AA    +AKDAT +
+ K
Sbjct  355  AQQAK----
ERSYETSDAAKEKTGATKDTVMGKTEEYKNAAADTLNQAKDATYETAVQGK  410

Query  305
DYSAQKAAETKDATMEKTKEYKDYTAQKAAETKDATMEKAKEAKDTTVQKTGEYKDYAAE  364
            D +     E +   M K +EYK      AAE  D++ +     KD T +  G  +D
+E
Sbjct  411  DKTYGTIEEARQKIMGKAEEYKG----SAAEATDSSKQT---
GKDLTDRAKGTGRD-VSE  462

Query  365
KAKEGKDVTVEKAKEGKDTTVGKMTELKDSAADAARKAMDMFLGKKEEVKGKAGETAEAA  424
            +A++      EKA E K+ + G +  + +  +  A      + GK EE + K  +
+ A
Sbjct  463
RAQDSAQQAKEKAGEKKEESKGYLQTVGEKVSQTAASLKGTWAGKTEEAQEKGQQATDDA  522

Query  425  KEKYEDTEF--------AARKKMEELKLQEEGVKDEAKQRAEAD--
RETAGDRGSAAKGT  474
            +++  D +         AA +KM E   + EG         EA    +T GDR
Sbjct  523
QQRLRDLKLKDEKEGGPAAGQKMREAGKKVEGAAHRGADEVEAKGVFQTVGDR-------  575

Query  475  IFGAMGSVKDAIVGK  489
            +     S++D ++GK
Sbjct  576  VSQTATSLRDTVLGK  590


 Score = 36.2 bits (82),  Expect = 3e-06, Method: Compositional
matrix adjust.
 Identities = 46/150 (31%), Positives = 70/150 (47%), Gaps = 32/150
(21%)

Query  51   GVIGSILKSVQGTLGQAKEVVVGK-------
AHDTAEVSRENTDYAYDKGREGGDVAAQK  103
            G + S+  ++    G  KE ++GK       A++TA+ ++E
+ +
```

```
Sbjct  320  GYLQSVTDTISERAGALKETLLGKTEEAKGKANETAQQAKER--------------
SYET  365

Query  104
AEEAKEKAKMAKDTTMGKAGEYKDYTAQKAEEAKEKAAQKAEETKEKAGEYKNYTAQKAG  163
            ++ AKEK    KDT MGK  EYK+  A    +AK+        T E A + K+ T
Sbjct  366  SDAAKEKTGATKDTVMGKTEEYKNAAADTLNQAKDA-------
TYETAVQGKDKTYGTIE  418

Query  164  EAKDTTLGKAGEYKDYAAQKAAEAKDTTAQ  193
            EA+   +GKA EYK      AAEA D++ Q
Sbjct  419  EARQKIMGKAEEYKG----SAAEATDSSKQ  444


 Score = 28.9 bits (63),  Expect = 4e-04, Method: Compositional
matrix adjust.
 Identities = 47/157 (30%), Positives = 69/157 (44%), Gaps = 33/157
(21%)

Query  356
GEYKDYAAEKAKEGKDVTVEKAKEGKDTTVGKMTELKDSAADAARKAMDMFLGKKEEVKG  415
            G  KD AA KA++ K               G +   ++ A+  A  A D  +GK E
KG
Sbjct  67   GHVKDAAASKAQDAK---------------
GGLEAGQEKASQYANAARDTVVGKAEAAKG  111

Query  416
KAGETAEAAKEKYEDTEFAARKKMEELKLQEEGVKDEAKQRAEADRETAGDRGSAAKGTI  475
            K  + A  AKEK  +T+  A+++         G+K+ A+QRA   +++A +
AAK
Sbjct  112  KTEDLAHQAKEKVGETKEGAQQRT-------
GGIKEGAQQRAGDVQQSATESKDAAKEKT  164

Query  476  F-------GAMGSVKDAIVGKLTMPSDVVKDKQQQEA  505
                    G   S  DA+ GK    +  VKD   Q A
Sbjct  165  SEGVEQSKGYFQSFTDALYGK----TQAVKDSTAQTA  197


 Score = 15.4 bits (28),  Expect = 6.9, Method: Compositional matrix
adjust.
 Identities = 29/100 (29%), Positives = 49/100 (49%), Gaps = 11/100
(11%)

Query  62   GTLGQAKEVVVGKAHD-----------
TAEVSRENTDYAYDKGREGGDVAAQKAEEAKEK  110
            GT+ +A++ ++GKA +           + +  ++ TD A   GR+  + A   A+
+AKEK
Sbjct  415
GTIEEARQKIMGKAEEYKGSAAEATDSSKQTGKDLTDRAKGTGRDVSERAQDSAQQAKEK  474

Query  111  AKMAKDTTMGKAGEYKDYTAQKAEEAKEKAAQKAEETKEK  150
            A   K+ + G      +  +Q A   K   A K EE +EK
Sbjct  475  AGEKKEESKGYLQTVGEKVSQTAASLKGTWAGKTEEAQEK  514
```

```
Lambda      K        H        a         alpha
   0.300    0.117    0.296    0.792     4.96

Gapped
Lambda      K        H        a         alpha    sigma
   0.267   0.0410    0.140     1.90     42.6     43.6

Effective search space used: 58830




Matrix: BLOSUM62
Gap Penalties: Existence: 11, Extension: 1
Neighboring words threshold: 11
Window for multiple hits: 40





Query= sp|P22242|DRPE_CRAPL_Desiccation-related_protein_PCC13-
62_OS=Craterostigma_plantagineum_PE=2_SV=1

Length=313

Subject= 272418-85_1_ORF2
>sp|P22242|DRPE_CRAPL_Desiccation-related_protein_PCC13-
62_OS=Craterostigma_plantagineum_PE=2_SV=1|||7e-96

Length=345


 Score =   260 bits (664),  Expect = 1e-88, Method: Compositional
matrix adjust.
 Identities = 141/266 (53%), Positives = 183/266 (69%), Gaps = 4/266
(2%)

Query  35
IPKSDVSLLEFPLNLELLEAEFFAWAAFGKGIDELEPELAKGGPSPIGVQKANLSPFIRD  94
            IP+ D  L+   LNLE LE+EFF + A G G+D   P LA GGP+PIG Q+ANL
+D
Sbjct  73
IPQGDADLILVALNLEYLESEFFLFGATGTGLDAFAPALAAGGPAPIGAQQANLDTLTKD  132

Query  95   IIAQFAYQEFGHVRAIQSSV-
EGFPRPLLDLSAKSFATVMDSAFGKTLKPPFDPYANDIN  153
            II QF  QE GH+RAI+ ++ + FPR LLDL  + F   M +A G+ L
PFDPYAN +N
Sbjct  133  IIYQFGLQEVGHLRAIKQTIPDAFPRVLLDLRKEVFDATMRAALGQ-
LPSPFDPYANSLN  191
```

```
Query  154
YLLACYVVPYVGLTGYVGANPKLESPVSRKLVAGLLAVEAGQDAIIRALLYERATDKVEP  213
            YLLA Y++PYVGLTGYVGANP+L+S  +R+LVAGLLAVE+GQDA+IR LLYE+  +
+V
Sbjct  192
YLLASYLIPYVGLTGYVGANPQLQSARARRLVAGLLAVESGQDAVIRTLLYEKKDEQV--  249

Query  214
YGITVAEFTNKISELRNKLGDKGVKDLGLIVEPELGAEGKISGNVLAGDKNSLAFPRTPE  273
             G++V + T+K+S+LRNKLG  G+ D GL+V   LGAEG  +GN LA DKNS+ +
RTPE
Sbjct  250
LGMSVGDITDKLSDLRNKLGHSGIVDEGLVVPKCLGAEGSTTGNSLAADKNSVGYARTPE  309

Query  274  RCLGSCTAAAMRPSPAAFIPKAPTGK  299
            +      A+     P  F P    G+
Sbjct  310  QIFSIVYASGDAAKPGGFYPDGGKGR  335



Lambda      K        H        a         alpha
   0.319    0.137    0.407    0.792     4.96

Gapped
Lambda      K        H        a         alpha    sigma
   0.267   0.0410    0.140     1.90     42.6     43.6

Effective search space used: 90345




Matrix: BLOSUM62
Gap Penalties: Existence: 11, Extension: 1
Neighboring words threshold: 11
Window for multiple hits: 40




Query= sp|P27323|HS901_ARATH_Heat_shock_protein_90-
1_OS=Arabidopsis_thaliana_GN=HSP90-1_PE=1_SV=3

Length=700

Subject= 108154-239_4_ORF1
>sp|P27323|HS901_ARATH_Heat_shock_protein_90-
1_OS=Arabidopsis_thaliana_GN=HSP90-1_PE=1_SV=3|||0

Length=373


 Score =   595 bits (1534),  Expect = 0.0, Method: Compositional
```

```
matrix adjust.
 Identities = 291/335 (87%), Positives = 320/335 (96%), Gaps = 1/335
(0%)

Query  255
VSHEWELINKQKPIWLRKPEEITKEEYAAFYKSLTNDWEDHLAVKHFSVEGQLEFKAILF  314
            VSHEW L+NKQKPIW+RKPEEITK+EYAAFYKSLTNDWE
+HLAVKHFSVEGQLEFKA+LF
Sbjct  40
VSHEWTLVNKQKPIWMRKPEEITKDEYAAFYKSLTNDWEEHLAVKHFSVEGQLEFKAVLF  99

Query  315
VPKRAPFDLFDTRKKLNNIKLYVRRVFIMDNCEELIPEYLSFVKGVVDSDDLPLNISRET  374
            VPKRAPFDLFDTRKK+NNIKLYVRRVFIMDNCEE++PEYLSFVKGVVDS
+DLPLNISRET
Sbjct  100
VPKRAPFDLFDTRKKMNNIKLYVRRVFIMDNCEEIMPEYLSFVKGVVDSEDLPLNISRET  159

Query  375
LQQNKILKVIRKNLVKKCIEMFNEIAENKEDYTKFYEAFSKNLKLGIHEDSQNRGKIADL  434
            LQQNKILKVIRKNLVKKC+EMF EIAENKEDY KFYEAF+KN+KLGIHEDSQNR K
+A+L
Sbjct  160
LQQNKILKVIRKNLVKKCVEMFFEIAENKEDYDKFYEAFAKNIKLGIHEDSQNRAKLAEL  219

Query  435
LRYHSTKSGDEMTSFKDYVTRMKEGQKDIFYITGESKKAVENSPFLERLKKRGYEVLYMV  494
            LR+HSTKSGDEMTS KDYVTRMK+GQ +I+YITGESKKAVENSPFLE+LKK
+GYEVL+MV
Sbjct  220
LRFHSTKSGDEMTSLKDYVTRMKDGQTEIYYITGESKKAVENSPFLEKLKKKGYEVLFMV  279

Query  495
DAIDEYAVGQLKEYDGKKLVSATKEGLKLEDETEEEKKKREEKKKSFENLCKTIKEILGD  554
            DAIDEYA+GQLKE+DGKKLVSATKEGLKLED  +E+KKK E K K  E LCK +K
+ILGD
Sbjct  280  DAIDEYAIGQLKEFDGKKLVSATKEGLKLEDSEDEKKKKEELKSKF-
EPLCKVMKDILGD  338

Query  555  KVEKVVVSDRIVDSPCCLVTGEYGWTANMERIMKA  589
            KVEKV+VSDR+VDSPCCLVTGEYGWTANMERIMKA
Sbjct  339  KVEKVIVSDRVVDSPCCLVTGEYGWTANMERIMKA  373



Lambda      K        H        a         alpha
   0.313    0.132    0.364    0.792     4.96

Gapped
Lambda      K        H        a         alpha    sigma
   0.267   0.0410    0.140     1.90     42.6     43.6

Effective search space used: 225774
```

Query= sp|P27323|HS901_ARATH_Heat_shock_protein_90-
1_OS=Arabidopsis_thaliana_GN=HSP90-1_PE=1_SV=3

Length=700

Subject= 21928-533_2_ORF1
>sp|P27323|HS901_ARATH_Heat_shock_protein_90-
1_OS=Arabidopsis_thaliana_GN=HSP90-1_PE=1_SV=3|||7e-137

Length=211

Score = 395 bits (1014), Expect = 6e-138, Method: Compositional matrix adjust.
Identities = 191/211 (91%), Positives = 200/211 (95%), Gaps = 1/211 (0%)

```
Query  6
TFAFQAEINQLLSLIINTFYSNKEIFLRELISNSSDALDKIRFESLTDKSKLDGQPELFI  65
           TFAFQAEINQLLSLIINTFYSNKEIFLRELISN+SDALDKIRFESLTDKSKLD
QPELFI
Sbjct  1
TFAFQAEINQLLSLIINTFYSNKEIFLRELISNASDALDKIRFESLTDKSKLDAQPELFI  60

Query  66
RLVPDKSNKTLSIIDSGIGMTKADLVNNLGTIARSGTKEFMEALQAGADVSMIGQFGVGF  125
           R++PDK+  TL+IIDSGIGMTKADLVNNLGTIARSGTKEFMEAL
AGADVSMIGQFGVGF
Sbjct  61
RIIPDKATNTLTIIDSGIGMTKADLVNNLGTIARSGTKEFMEALAAGADVSMIGQFGVGF  120

Query  126
YSAYLVAEKVVVTTKHNDDEQYVWESQAGGSFTVTRDVDGEPLGRGTKITLFLKDDQLEY  185
           YSAYLVAEKVVVT KHNDDEQY+WESQAGGSFTVTRD  GEPLGRGTKI L LK
+DQL+Y
Sbjct  121  YSAYLVAEKVVVTAKHNDDEQYIWESQAGGSFTVTRD-
HGEPLGRGTKIMLHLKEDQLDY  179

Query  186  LEERRLKDLVKKHSEFISYPIYLWTEKTTEK  216
            LEERRLKDL+KKHSEFISYPIYLW EKTT K
Sbjct  180  LEERRLKDLIKKHSEFISYPIYLWEEKTTTK  210
```

Score = 14.6 bits (26), Expect = 5.4, Method: Compositional matrix adjust.
Identities = 4/10 (40%), Positives = 8/10 (80%), Gaps = 0/10 (0%)

```
Query  430  KIADLLRYHS  439
            ++ DL++ HS
Sbjct  184  RLKDLIKKHS  193
```

```
Lambda      K        H        a         alpha
   0.313    0.132    0.364    0.792     4.96

Gapped
Lambda      K        H        a         alpha    sigma
   0.267   0.0410    0.140     1.90     42.6     43.6

Effective search space used: 225774




Matrix: BLOSUM62
Gap Penalties: Existence: 11, Extension: 1
Neighboring words threshold: 11
Window for multiple hits: 40





Query= sp|P31170|HS25P_ARATH_25.3_kDa_heat_shock_protein,
_chloroplastic_OS=Arabidopsis_thaliana_GN=HSP25.3_PE=2_SV=1

Length=227

Subject= 172805-164_1_ORF2
>sp|P31170|HS25P_ARATH_25.3_kDa_heat_shock_protein,
_chloroplastic_OS=Arabidopsis_thaliana_GN=HSP25.3_PE=2_SV=1|||9e-38

Length=247


 Score =   122 bits (306),  Expect = 6e-38, Method: Compositional
matrix adjust.
 Identities = 67/174 (39%), Positives = 102/174 (59%), Gaps = 3/174
(2%)

Query  54
VVQQGQQKGNQGSSVEKRPQQRLTMDVSPFGLLDPLSPMRTMRQMLDTMDRMFEDTMPVS  113
             V  G+    Q  +V  + +  L   + P GLL P     ++  M+DT+DR+F+ +
+P S
Sbjct  77   AVAVGRSAKEQTPAVSGKNRPTLPRSLFP-
GLLSPFYQPTSLTHMMDTVDRLFDFSIP-S  134

Query  114
GRNRGGSGVSEIRAPWDIKEEEHEIKMRFDMPGLSKEDVKISVEDNVLVIKGEQKKEDSD  173
            G   G    +  R PWD+ E+E   K+R DMPG+SKEDVK+ VED  LVIK E
E  +
Sbjct  135  GSTSGIRNATN-
RMPWDVMEDEKCFKLRVDMPGMSKEDVKLCVEDGDLVIKAEHDAEKEE  193
```

```
Query  174  DSWSGRSVSSYGTRLQLPDNCEKDKIKAELKNGVLFITIPKTKVERKVIDVQIQ
227
              W+ R+  SY  R++LP+N + + IKAE+K+GVL +  PK +  ++  +V I+
Sbjct  194  GDWASRTYGSYNVRIKLPENVDFNGIKAEMKDGVLKVQAPKVEGPKQKHEVPIE
247



Lambda      K        H        a         alpha
   0.313    0.129    0.361    0.792     4.96

Gapped
Lambda      K        H        a         alpha    sigma
   0.267   0.0410    0.140     1.90     42.6     43.6

Effective search space used: 45696




Query= sp|P31170|HS25P_ARATH_25.3_kDa_heat_shock_protein,
_chloroplastic_OS=Arabidopsis_thaliana_GN=HSP25.3_PE=2_SV=1

Length=227

Subject= 189214-147_1_ORF1
>sp|P31170|HS25P_ARATH_25.3_kDa_heat_shock_protein,
_chloroplastic_OS=Arabidopsis_thaliana_GN=HSP25.3_PE=2_SV=1|||6e-40

Length=212


 Score =   128 bits (322),  Expect = 1e-40, Method: Compositional
matrix adjust.
 Identities = 76/183 (42%), Positives = 107/183 (58%), Gaps = 12/183
(7%)

Query  47   QRENSIDVVQQGQQKGNQG--
SSVEKRPQQRLTMDVSPFGLLDPLSPMRTMRQMLDTMDR  104
            Q E+++D     + K +QG  SS   R    +  DV P     PL    ++  M+D
M+
Sbjct  40   QNEDNVDT----KGKASQGTPSSARVRFPVGIFSDVFP-----
PLGRSASLLNMMDIMED  90

Query  105
MFEDTMPVSGRNRGGSGVSEIRAPWDIKEEEHEIKMRFDMPGLSKEDVKISVEDNVLVIK  164
            +F  +  VS      +  S  R PWD+ E+    K+R DMPGLSKE+VK+ VE+
L IK
Sbjct  91   LFSSSQ-
VSANVAPQAFRSSNRTPWDVMEDAKAFKLRLDMPGLSKEEVKVDVEEGNLTIK  149

Query  165
GEQKKEDSDDSWSGRSVSSYGTRLQLPDNCEKDKIKAELKNGVLFITIPKTKVERKVIDV  224
            GE K E+ +D WS RSV SY  +++LPDN + D IKAELKNGVL +T PK +  +K
```

```
+ V
Sbjct  150
GEHKAEEGEDDWSLRSVGSYNIKIKLPDNVKADAIKAELKNGVLLVTAPKMEETKKRLAV  209

Query  225  QIQ  227
            +I+
Sbjct  210  RIE  212



Lambda      K        H        a         alpha
   0.313    0.129    0.361    0.792     4.96

Gapped
Lambda      K        H        a         alpha    sigma
   0.267   0.0410    0.140     1.90     42.6     43.6

Effective search space used: 45696




Matrix: BLOSUM62
Gap Penalties: Existence: 11, Extension: 1
Neighboring words threshold: 11
Window for multiple hits: 40






Query= sp|P36181|
HSP80_SOLLC_Heat_shock_cognate_protein_80_OS=Solanum_lycop
ersicum_GN=HSC80_PE=2_SV=1

Length=699

Subject= 171819-165_4_ORF1
>sp|P36181|HSP80_SOLLC_Heat_shock_cognate_protein_80_OS=Solanum_lyco
persicum_GN=HSC80_PE=2_SV=1|||8e-59

Length=94


 Score =   182 bits (461),  Expect = 4e-59, Method: Compositional
matrix adjust.
 Identities = 90/94 (96%), Positives = 91/94 (97%), Gaps = 0/94 (0%)

Query  17
LSLIINTFYSNKEIFLRELISNSSDALDKIRFESLTDKSKLDGQPELFIHIIPDKANNTL  76
            LSLIINTFYSNKEIFLRELISN+SDALDKIRFESLTDKSKLD QPELFIHIIPDKA
TL
Sbjct  1
LSLIINTFYSNKEIFLRELISNASDALDKIRFESLTDKSKLDAQPELFIHIIPDKAAGTL  60
```

```
Query  77   TIIDSGIGMTKADLVNNLGTIARSGTKEFMEALA  110
            TIIDSGIGMTKADLVNNLGTIARSGTKEFMEALA
Sbjct  61   TIIDSGIGMTKADLVNNLGTIARSGTKEFMEALA  94


 Score = 13.5 bits (23),  Expect = 4.2, Method: Compositional matrix
adjust.
 Identities = 5/16 (31%), Positives = 9/16 (56%), Gaps = 0/16 (0%)

Query  347  ELIPEYLSFVKGIVDS  362
             +IP+  +    I+DS
Sbjct  50   HIIPDKAAGTLTIIDS  65



Lambda      K        H        a         alpha
   0.312    0.132    0.361    0.792     4.96

Gapped
Lambda      K        H        a         alpha    sigma
   0.267   0.0410    0.140     1.90     42.6     43.6

Effective search space used: 47996




Matrix: BLOSUM62
Gap Penalties: Existence: 11, Extension: 1
Neighboring words threshold: 11
Window for multiple hits: 40





Query= sp|P37120|C75A2_SOLME_Flavonoid_3',
5'-hydroxylase_OS=Solanum_melongena_GN=CYP75A2_PE=2_SV=1

Length=513

Subject= 8643-801_5_ORF1
>sp|P37120|C75A2_SOLME_Flavonoid_3',
5'-hydroxylase_OS=Solanum_melongena_GN=CYP75A2_PE=2_SV=1|||5e-110

Length=525


 Score =   314 bits (804),  Expect = 4e-104, Method: Compositional
matrix adjust.
 Identities = 181/501 (36%), Positives = 281/501 (56%), Gaps =
22/501 (4%)
```

```
Query  20   IIIQKLIATG---SWRRRR-----
LPPGPEGWPVIGALPLLGGMPHVALAKMAKKYGPIM  71
            II  + I  G    WRR       +PPGP G P++G L  LG +PH   A+++
GP+M
Sbjct  24
IIAARCILLGIMWVWRREAGAHIIMPPGPRGLPILGYLHKLGALPHQTFARLSDICGPLM  83

Query  72
YLKVGTCGMVVASTPNAAKAFLKTLDINFSNRPPNAGATHMAYNAQDMVFAPYGPRWKLL  131
             +++G   ++VAS+P+ A   L   D  F+ RP    A  + Y  ++++F+  GP
WKL+
Sbjct  84   CVRLGRVPLLVASSPDMASLIL---DKTFAGRP-
VLIAPSIVYGGRNILFSQPGPYWKLM  139

Query  132
RKLSNLHMLGGKALENWANVRANELGHMLKSMFDASHVGERIVVADMLTFAMANMIGQVM  191
            R++    +L  K L ++  VRA+E+  +L S+  A   G  + + D+L   + N I
+
Sbjct  140  RQIFTTDLLTSKRLSHFRPVRAHEMRGLLLSVLAAR--
GSPLCIRDLLHTTINNTISTMA  197

Query  192  LSKRVF-
VEKGKEVNEFKNMVVELMTVAGYFNIGDFIPQIAWMDLQGIEKGMKKLHKKFD  250
            L K ++ V  G  V    + V+E++ + G FN GD+IP +AWMDLQG  K  K++
+
Sbjct  198
LGKPLYQVSAGPNVGTIVSTVMEIVNLIGQFNWGDYIPYLAWMDLQGYGKQSKEIGGRVR  257

Query  251  DLLTKMFEEHEATSNE--RKGKPDFLDFIMANRDNSEGERLSITN--
IKALLLNLFTAGT  306
             +L  + ++    +++       D LD ++A   +++ + L I +  I+A+LL +F
AG+
Sbjct  258
SVLQAVIDKRRRCADDIDSPAACDLLDLLLAASADAKHKELHIGDDSIRAVLLGIFIAGS  317

Query  307
DTSSSVIEWALTEMMKNPTIFKKAQQEMDQIIGKNRRFIESDIPNLPYLRAICKEAFRKH  366
            DT+S  IEWAL E++ NP   ++ Q+E+D+++G+ R   E D+ NL YLRA+  EA
R H
Sbjct  318
DTASITIEWALAELLANPEKLRRVQEELDEVVGRERVVEEGDLANLVYLRAVVNEALRLH  377

Query  367
PSTPLNLPRVSSDACTIDGYYIPKNTRLSVNIWAIGRDPDVWENPLEFIPERFLSEKNAK  426
            P TPL  P    +AC I GY IP +T   VNIWAI RDP +W NPL+F PERFL
Sbjct  378
PPTPLLAPHRCLEACHIGGYRIPADTLAFVNIWAIHRDPSLWANPLDFCPERFLPSLLDV  437

Query  427
IEHRGNDFELIPFGAGRRICAGTRMGIVMVEYILGTLIHSFDWKLPNDVVDINMEETFGL  486
                G  F  +PFG+G R C G ++G++  + +L  L+H+F W  P       + E
FGL
Sbjct  438  TP--GQHFGFLPFGSGPRTCPGWKLGLLNAQNVLAHLLHAFHWTTPTGKPP-
PLNEKFGL  494
```

```
Query  487  ALQKAVPLEAIVTPRLSFDIY  507
             +   +PL  +  PRL   +Y
Sbjct  495  TVAIDIPLSVVPMPRLPMPLY  515



Lambda      K        H        a         alpha
   0.322    0.138    0.417    0.792     4.96

Gapped
Lambda      K        H        a         alpha    sigma
   0.267   0.0410    0.140     1.90     42.6     43.6

Effective search space used: 234220




Matrix: BLOSUM62
Gap Penalties: Existence: 11, Extension: 1
Neighboring words threshold: 11
Window for multiple hits: 40






Query= sp|P37123|
C77A1_SOLME_Cytochrome_P450_77A1_(Fragment)_OS=Solanum_mel
ongena_GN=CYP77A1_PE=2_SV=1

Length=499

Subject= 212095-127_1_ORF1
>sp|P37123|C77A1_SOLME_Cytochrome_P450_77A1_(Fragment)_OS=Solanum_me
longena_GN=CYP77A1_PE=2_SV=1|||1e-135

Length=516


 Score =   376 bits (965),  Expect = 2e-128, Method: Compositional
matrix adjust.
 Identities = 203/475 (43%), Positives = 297/475 (63%), Gaps =
19/475 (4%)

Query  26   NLPPGP-
PGWPIVGNLFQVAGSGKQFFEYIRDLKPKYGSIFTLKMGSRTMIIVASAELAH  84
            NLPP P    P++G+   +       F  I  L+   G IFTL +GS  +I + SA
LAH
Sbjct  57   NLPPSPFLSLPLLGHPHYLPRLRNSSF--
IHSLRNSLGPIFTLHVGSTPIIYITSAALAH  114

Query  85
```

```
EALIQKGQIFASRPRENPTRTIFSCNKFSVNAAVYGPVWRSLRRNMVQNMLSPSRLKEFR  144
            EAL+QK  +FA+RP   P+R +F+ N  ++N+A YGP WR++RRN+V  ML+  ++
F+
Sbjct  115  EALVQKSLLFAARPLL-
PSRVLFTNNFRNINSATYGPYWRAMRRNLVHEMLAAPKILSFK  173

Query  145
EFREIAMDKLIERIRVDAKENNDVVWALKNARFAVFYILVAMCFGVEMDNEEMIERVDQM  204
              R   +D +I RI  +A+ N  +V    N R A+  +L+ MCFG  M  ++++
+  +
Sbjct  174  PVRAHILDNMISRILTEAQHNEGIVSVYSNVRTAMLKLLLFMCFGFHMPEDDLMH-
ISAL  232

Query  205
MKDVLIVLDPRIDDFLPILRLFVGYKQRKRVNEVRKRQIETLVPLIEKRRSVVQNPGSDK  264
            + ++L++    + DF   LR+F   K R++   +R +Q++     I+K + + +
Sbjct  233  IDEILLLAIGNLQDFYSYLRVF---
KSRRKALSIRAKQVQLFSSQIDKHKELHKL----G  285

Query  265  TAASFSYLDTLFDVKVEGRKSGPTNAE-
LVTLCSEFLNGGTDTTATALEWGIGRLMENPT  323
              A  SY++TL  +      S P + + LVTLCSEFL GGTDTT T LEW +  L
+E+P+
Sbjct  286  ELAPGSYVETLLHMDA----
SNPLSIDDLVTLCSEFLVGGTDTTVTTLEWTMACLVEDPS  341

Query  324
IQNQLYQEIKTIVGDKKVDENDIEKMPYLNAVVKELLRKHPPTYFTLTHSVTEPVKLAGY  383
            IQN+LY +I  +VGD+ +DE D+  +PYL AV++E LR HPP +  L H+V+E  K
+ GY
Sbjct  342
IQNKLYDQISDVVGDRLIDEEDLPHLPYLQAVIRETLRLHPPGHSLLPHAVSELCKVGGY  401

Query  384
DIPMDTNVEFFVHGISHDPNVWSDPEKFDPDRFLSGREDADITGVKEVKMMPFGVGRRIC  443
            DIP +  V+F V  IS DP +W +P +F P+RFL+   D DITG +EV M+PFG
GRRIC
Sbjct  402  DIPPNAVVQFHVTSISRDPEIWEEPLEFRPERFLTA--
DVDITGTREVTMIPFGAGRRIC  459

Query  444  PGLGMATVHVNLMLARMVQEFEWFAYPGNNKVDFSEKLEFTVVMKNPLRAKVKLR
498
            PGLG+A+VH  L +AR+VQ F+W  YP   +VD +EK  FTV MK+PLRA VK R
Sbjct  460  PGLGLASVHTELFVARLVQAFQWTNYPSGERVDLTEKPIFTVRMKHPLRALVKER
514



Lambda      K        H        a         alpha
   0.322    0.139    0.412    0.792     4.96

Gapped
Lambda      K        H        a         alpha    sigma
   0.267   0.0410    0.140     1.90     42.6     43.6
```

```
Effective search space used: 224130


Matrix: BLOSUM62
Gap Penalties: Existence: 11, Extension: 1
Neighboring words threshold: 11
Window for multiple hits: 40


Query= sp|P37707|B2_DAUCA_B2_protein_OS=Daucus_carota_PE=2_SV=1

Length=207

Subject= 20165-555_5_ORF2
>sp|P37707|B2_DAUCA_B2_protein_OS=Daucus_carota_PE=2_SV=1|||1e-25

Length=448


 Score = 89.7 bits (221),  Expect = 4e-25, Method: Compositional
matrix adjust.
 Identities = 54/175 (31%), Positives = 95/175 (54%), Gaps = 14/175
(8%)

Query  33   KNKNNNNNSESGNKNGGENKNGVEKRFKTLPPAESLPRNETV---
GGYIFVCNNDTMQEN  89
            K  +     + GN+   + + G     K    A+S+P+ + +    G IF+CN+DT
++
Sbjct  95   KTSDGKETPKKGNRRRSKGRKG-----
KKPSNADSVPKEKKIIDFDGLIFMCNSDTKKDC  149

Query  90
LKRQLFGLPPRYRDSVRAITPGLPLFLYNYSTHQLHGVFEAASFGGTNIDPTAWEDKKNQ  149
             K ++FGLP   ++ V  +  G  LFL++     LHGV++A+S GG N+   A++D
Sbjct  150
FKYRVFGLPEGKKNLVEQVKKGTKLFLFDIDKKVLHGVYKASSEGGINLIEEAFKDSNR-  208

Query  150  GESRFPAQVRVMTRKICEPLEEDSFRPIL--HHYDGPKFRLELNIPEAISLLDIF
202
               +FPAQVR    K C PL+E++F+  +  +++   +F+ ELN  +   L+ +F
Sbjct  209  ---KFPAQVRFRIHKDCMPLDENAFKLAIKENYFRKNQFKCELNAEQVGRLMKLF
260


 Score = 16.2 bits (30),  Expect = 0.94, Method: Compositional
matrix adjust.
 Identities = 6/9 (67%), Positives = 6/9 (67%), Gaps = 0/9 (0%)
```

```
Query  112  LPLFLYNYS  120
            LPLFL   S
Sbjct  2    LPLFLLQIS  10


 Score = 13.9 bits (24),  Expect = 6.3, Method: Compositional matrix
adjust.
 Identities = 4/5 (80%), Positives = 4/5 (80%), Gaps = 0/5 (0%)

Query  97   LPPRY  101
            LPP Y
Sbjct  332  LPPSY  336



Lambda      K        H        a         alpha
   0.314    0.134    0.397    0.792     4.96

Gapped
Lambda      K        H        a         alpha    sigma
   0.267   0.0410    0.140     1.90     42.6     43.6

Effective search space used: 75780




Query= sp|P37707|B2_DAUCA_B2_protein_OS=Daucus_carota_PE=2_SV=1

Length=207

Subject= 277631-81_6_ORF2
>sp|P37707|B2_DAUCA_B2_protein_OS=Daucus_carota_PE=2_SV=1|||1e-27

Length=377


 Score = 95.5 bits (236),  Expect = 1e-27, Method: Compositional
matrix adjust.
 Identities = 50/137 (36%), Positives = 79/137 (58%), Gaps = 7/137
(5%)

Query  68
LPRNETVGGYIFVCNNDTMQENLKRQLFGLPPRYRDSVRAITPGLPLFLYNYSTHQLHGV  127
            LP  +   G IF+CN+ T ++    ++ GLP   RD V  I PG  LFLY++
+L+G+
Sbjct  43
LPPRKGPAGLIFMCNSKTKRDCFHYKVLGLPLAKRDLVEQIVPGTFLFLYDFDARELYGI  102

Query  128  FEAASFGGTNIDPTAWEDKKNQGESRFPAQVRVMTRKICEPLEEDSFRPIL--
HHYDGPK  185
            +EA+S GG N++P A+E     G+  +PAQVR    + C PL ED  R  +  ++Y
+
Sbjct  103  YEASSHGGVNLEPKAFE-----
```

```
GQGNYPAQVRFDIHRECLPLSEDLLRDAIKENYYARNR  157

Query  186  FRLELNIPEAISLLDIF  202
            F++EL   +   L+ +F
Sbjct  158  FQIELTSDQVSRLIQLF  174


 Score = 16.9 bits (32),  Expect = 0.54, Method: Compositional
matrix adjust.
 Identities = 6/8 (75%), Positives = 6/8 (75%), Gaps = 0/8 (0%)

Query  173  SFRPILHH  180
            SFRP  HH
Sbjct  361  SFRPSGHH  368



Lambda      K        H        a         alpha
   0.314    0.134    0.397    0.792     4.96

Gapped
Lambda      K        H        a         alpha    sigma
   0.267   0.0410    0.140     1.90     42.6     43.6

Effective search space used: 75780




Query= sp|P37707|B2_DAUCA_B2_protein_OS=Daucus_carota_PE=2_SV=1

Length=207

Subject= 277928-81_6_ORF2
>sp|P37707|B2_DAUCA_B2_protein_OS=Daucus_carota_PE=2_SV=1|||1e-27

Length=362


 Score = 95.1 bits (235),  Expect = 1e-27, Method: Compositional
matrix adjust.
 Identities = 50/137 (36%), Positives = 79/137 (58%), Gaps = 7/137
(5%)

Query  68
LPRNETVGGYIFVCNNDTMQENLKRQLFGLPPRYRDSVRAITPGLPLFLYNYSTHQLHGV  127
            LP  +   G IF+CN+ T ++    ++ GLP   RD V  I PG  LFLY++
+L+G+
Sbjct  28
LPPRKGPAGLIFMCNSKTKRDCFHYKVLGLPLAKRDLVEQIVPGTFLFLYDFDARELYGI  87

Query  128  FEAASFGGTNIDPTAWEDKKNQGESRFPAQVRVMTRKICEPLEEDSFRPIL--
HHYDGPK  185
            +EA+S GG N++P A+E     G+  +PAQVR    + C PL ED  R  +  ++Y
```

```
+
Sbjct  88   YEASSHGGVNLEPKAFE-----
GQGNYPAQVRFDIHRECLPLSEDLLRDAIKENYYARNR  142

Query  186  FRLELNIPEAISLLDIF  202
            F++EL   +   L+ +F
Sbjct  143  FQIELTSDQVSRLIQLF  159


 Score = 16.9 bits (32),  Expect = 0.53, Method: Compositional
matrix adjust.
 Identities = 6/8 (75%), Positives = 6/8 (75%), Gaps = 0/8 (0%)

Query  173  SFRPILHH  180
            SFRP  HH
Sbjct  346  SFRPSGHH  353



Lambda      K        H        a         alpha
   0.314    0.134    0.397    0.792     4.96

Gapped
Lambda      K        H        a         alpha    sigma
   0.267   0.0410    0.140     1.90     42.6     43.6

Effective search space used: 75780




Query= sp|P37707|B2_DAUCA_B2_protein_OS=Daucus_carota_PE=2_SV=1

Length=207

Subject= 278118-81_6_ORF2
>sp|P37707|B2_DAUCA_B2_protein_OS=Daucus_carota_PE=2_SV=1|||1e-27

Length=364


 Score = 95.1 bits (235),  Expect = 1e-27, Method: Compositional
matrix adjust.
 Identities = 50/137 (36%), Positives = 79/137 (58%), Gaps = 7/137
(5%)

Query  68
LPRNETVGGYIFVCNNDTMQENLKRQLFGLPPRYRDSVRAITPGLPLFLYNYSTHQLHGV  127
            LP  +   G IF+CN+ T ++    ++ GLP   RD V  I PG  LFLY++
+L+G+
Sbjct  30
LPPRKGPAGLIFMCNSKTKRDCFHYKVLGLPLAKRDLVEQIVPGTFLFLYDFDARELYGI  89

Query  128  FEAASFGGTNIDPTAWEDKKNQGESRFPAQVRVMTRKICEPLEEDSFRPIL--
```

```
HHYDGPK  185
            +EA+S GG N++P A+E     G+  +PAQVR    + C PL ED  R  +  ++Y
+
Sbjct  90   YEASSHGGVNLEPKAFE-----
GQGNYPAQVRFDIHRECLPLSEDLLRDAIKENYYARNR  144

Query  186  FRLELNIPEAISLLDIF  202
            F++EL   +   L+ +F
Sbjct  145  FQIELTSDQVSRLIQLF  161


 Score = 16.9 bits (32),  Expect = 0.52, Method: Compositional
matrix adjust.
 Identities = 6/8 (75%), Positives = 6/8 (75%), Gaps = 0/8 (0%)

Query  173  SFRPILHH  180
            SFRP  HH
Sbjct  348  SFRPSGHH  355



Lambda      K        H        a         alpha
   0.314    0.134    0.397    0.792     4.96

Gapped
Lambda      K        H        a         alpha    sigma
   0.267   0.0410    0.140     1.90     42.6     43.6

Effective search space used: 75780




Query= sp|P37707|B2_DAUCA_B2_protein_OS=Daucus_carota_PE=2_SV=1

Length=207

Subject= 278438-81_6_ORF2
>sp|P37707|B2_DAUCA_B2_protein_OS=Daucus_carota_PE=2_SV=1|||1e-27

Length=397


 Score = 95.5 bits (236),  Expect = 2e-27, Method: Compositional
matrix adjust.
 Identities = 50/137 (36%), Positives = 79/137 (58%), Gaps = 7/137
(5%)

Query  68
LPRNETVGGYIFVCNNDTMQENLKRQLFGLPPRYRDSVRAITPGLPLFLYNYSTHQLHGV  127
            LP  +   G IF+CN+ T ++    ++ GLP   RD V  I PG  LFLY++
+L+G+
Sbjct  63
LPPRKGPAGLIFMCNSKTKRDCFHYKVLGLPLAKRDLVEQIVPGTFLFLYDFDARELYGI  122
```

```
Query  128  FEAASFGGTNIDPTAWEDKKNQGESRFPAQVRVMTRKICEPLEEDSFRPIL--
HHYDGPK  185
            +EA+S GG N++P A+E     G+  +PAQVR    + C PL ED  R  +  ++Y
+
Sbjct  123  YEASSHGGVNLEPKAFE-----
GQGNYPAQVRFDIHRECLPLSEDLLRDAIKENYYARNR  177

Query  186  FRLELNIPEAISLLDIF  202
            F++EL   +   L+ +F
Sbjct  178  FQIELTSDQVSRLIQLF  194


 Score = 16.5 bits (31),  Expect = 0.60, Method: Compositional
matrix adjust.
 Identities = 6/8 (75%), Positives = 6/8 (75%), Gaps = 0/8 (0%)

Query  173  SFRPILHH  180
            SFRP  HH
Sbjct  381  SFRPSGHH  388



Lambda      K        H        a         alpha
   0.314    0.134    0.397    0.792     4.96

Gapped
Lambda      K        H        a         alpha    sigma
   0.267   0.0410    0.140     1.90     42.6     43.6

Effective search space used: 75780



Query= sp|P37707|B2_DAUCA_B2_protein_OS=Daucus_carota_PE=2_SV=1

Length=207

Subject= 357656-40_2_ORF1
>sp|P37707|B2_DAUCA_B2_protein_OS=Daucus_carota_PE=2_SV=1|||3e-24

Length=414


 Score = 86.3 bits (212),  Expect = 4e-24, Method: Compositional
matrix adjust.
 Identities = 45/135 (33%), Positives = 76/135 (56%), Gaps = 6/135
(4%)

Query  70
RNETVGGYIFVCNNDTMQENLKRQLFGLPPRYRDSVRAITPGLPLFLYNYSTHQLHGVFE  129
            R   + G IF+CN  T ++  K  +FG P + +D V+ +  G+ LFLY+  + +L
+G++E
```

```
Sbjct  127
REIKIAGMIFMCNAVTKKDCFKYGVFGFPDQKKDIVQQVKRGMKLFLYDIDSKRLYGIYE  186

Query  130  AASFGGTNIDPTAWEDKKNQGESRFPAQVRVMTRKICEPLEEDSFRPILHHY--
DGPKFR  187
            A+S GG ++ P A+     + + +FPAQVR    K C PLE+  F+  +      G
+F
Sbjct  187  ASSRGGMDLVPEAF----
RESDRKFPAQVRFRIHKDCIPLEDSDFKQAIKDKFNRGGRFN  242

Query  188  LELNIPEAISLLDIF  202
             EL+  +   L+ +F
Sbjct  243  CELSSEQVGKLMRLF  257


 Score = 17.3 bits (33),  Expect = 0.38, Method: Compositional
matrix adjust.
 Identities = 10/27 (37%), Positives = 16/27 (59%), Gaps = 5/27
(19%)

Query  169  LEEDSF---RPILHH--YDGPKFRLEL  190
            LE+ S    RP+LH   Y G +++ E+
Sbjct  373  LEQTSLIAKRPLLHDPLYRGAEYQREM  399



Lambda      K        H        a         alpha
   0.314    0.134    0.397    0.792     4.96

Gapped
Lambda      K        H        a         alpha    sigma
   0.267   0.0410    0.140     1.90     42.6     43.6

Effective search space used: 75780




Query= sp|P37707|B2_DAUCA_B2_protein_OS=Daucus_carota_PE=2_SV=1

Length=207

Subject= 357735-40_2_ORF1
>sp|P37707|B2_DAUCA_B2_protein_OS=Daucus_carota_PE=2_SV=1|||3e-24

Length=405


 Score = 86.3 bits (212),  Expect = 4e-24, Method: Compositional
matrix adjust.
 Identities = 45/135 (33%), Positives = 76/135 (56%), Gaps = 6/135
(4%)

Query  70
```

```
RNETVGGYIFVCNNDTMQENLKRQLFGLPPRYRDSVRAITPGLPLFLYNYSTHQLHGVFE  129
            R   + G IF+CN  T ++  K  +FG P + +D V+ +  G+ LFLY+  + +L
+G++E
Sbjct  127
REIKIAGMIFMCNAVTKKDCFKYGVFGFPDQKKDIVQQVKRGMKLFLYDIDSKRLYGIYE  186

Query  130  AASFGGTNIDPTAWEDKKNQGESRFPAQVRVMTRKICEPLEEDSFRPILHHY--
DGPKFR  187
            A+S GG ++ P A+     + + +FPAQVR    K C PLE+  F+  +      G
+F
Sbjct  187  ASSRGGMDLVPEAF----
RESDRKFPAQVRFRIHKDCIPLEDSDFKQAIKDKFNRGGRFN  242

Query  188  LELNIPEAISLLDIF  202
             EL+  +   L+ +F
Sbjct  243  CELSSEQVGKLMRLF  257


 Score = 17.3 bits (33),  Expect = 0.37, Method: Compositional
matrix adjust.
 Identities = 10/27 (37%), Positives = 16/27 (59%), Gaps = 5/27
(19%)

Query  169  LEEDSF---RPILHH--YDGPKFRLEL  190
            LE+ S    RP+LH   Y G +++ E+
Sbjct  364  LEQTSLIAKRPLLHDPLYRGAEYQREM  390



Lambda      K        H        a         alpha
   0.314    0.134    0.397    0.792     4.96

Gapped
Lambda      K        H        a         alpha    sigma
   0.267   0.0410    0.140     1.90     42.6     43.6

Effective search space used: 75780




Query= sp|P37707|B2_DAUCA_B2_protein_OS=Daucus_carota_PE=2_SV=1

Length=207

Subject= 5254-979_5_ORF3
>sp|P37707|B2_DAUCA_B2_protein_OS=Daucus_carota_PE=2_SV=1|||1e-27

Length=359


 Score = 95.1 bits (235),  Expect = 1e-27, Method: Compositional
matrix adjust.
 Identities = 50/137 (36%), Positives = 79/137 (58%), Gaps = 7/137
```

```
(5%)

Query  68
LPRNETVGGYIFVCNNDTMQENLKRQLFGLPPRYRDSVRAITPGLPLFLYNYSTHQLHGV  127
            LP  +   G IF+CN+ T ++    ++ GLP   RD V  I PG  LFLY++
+L+G+
Sbjct  28
LPPRKGPAGLIFMCNSKTKRDCFHYKVLGLPLAKRDLVEQIVPGTFLFLYDFDARELYGI  87

Query  128  FEAASFGGTNIDPTAWEDKKNQGESRFPAQVRVMTRKICEPLEEDSFRPIL--
HHYDGPK  185
            +EA+S GG N++P A+E     G+  +PAQVR    + C PL ED  R  +  ++Y
+
Sbjct  88   YEASSHGGVNLEPKAFE-----
GQGNYPAQVRFDIHRECLPLSEDLLRDAIKENYYARNR  142

Query  186  FRLELNIPEAISLLDIF  202
            F++EL   +   L+ +F
Sbjct  143  FQIELTSDQVSRLIQLF  159


 Score = 16.9 bits (32),  Expect = 0.52, Method: Compositional
matrix adjust.
 Identities = 6/8 (75%), Positives = 6/8 (75%), Gaps = 0/8 (0%)

Query  173  SFRPILHH  180
            SFRP  HH
Sbjct  346  SFRPSGHH  353



Lambda      K        H        a         alpha
   0.314    0.134    0.397    0.792     4.96

Gapped
Lambda      K        H        a         alpha    sigma
   0.267   0.0410    0.140     1.90     42.6     43.6

Effective search space used: 75780




Query= sp|P37707|B2_DAUCA_B2_protein_OS=Daucus_carota_PE=2_SV=1

Length=207

Subject= 5256-979_6_ORF3
>sp|P37707|B2_DAUCA_B2_protein_OS=Daucus_carota_PE=2_SV=1|||1e-27

Length=377


 Score = 95.1 bits (235),  Expect = 1e-27, Method: Compositional
```

```
matrix adjust.
 Identities = 50/137 (36%), Positives = 79/137 (58%), Gaps = 7/137
(5%)

Query  68
LPRNETVGGYIFVCNNDTMQENLKRQLFGLPPRYRDSVRAITPGLPLFLYNYSTHQLHGV  127
            LP  +   G IF+CN+ T ++    ++ GLP   RD V  I PG  LFLY++
+L+G+
Sbjct  43
LPPRKGPAGLIFMCNSKTKRDCFHYKVLGLPLAKRDLVEQIVPGTFLFLYDFDARELYGI  102

Query  128  FEAASFGGTNIDPTAWEDKKNQGESRFPAQVRVMTRKICEPLEEDSFRPIL--
HHYDGPK  185
            +EA+S GG N++P A+E     G+  +PAQVR    + C PL ED  R  +  ++Y
+
Sbjct  103  YEASSHGGVNLEPKAFE-----
GQGNYPAQVRFDIHRECLPLSEDLLRDAIKENYYARNR  157

Query  186  FRLELNIPEAISLLDIF  202
            F++EL   +   L+ +F
Sbjct  158  FQIELTSDQVSRLIQLF  174


 Score = 16.5 bits (31),  Expect = 0.56, Method: Compositional
matrix adjust.
 Identities = 6/8 (75%), Positives = 6/8 (75%), Gaps = 0/8 (0%)

Query  173  SFRPILHH  180
            SFRP  HH
Sbjct  361  SFRPSGHH  368



Lambda      K        H        a         alpha
   0.314    0.134    0.397    0.792     4.96

Gapped
Lambda      K        H        a         alpha    sigma
   0.267   0.0410    0.140     1.90     42.6     43.6

Effective search space used: 75780




Query= sp|P37707|B2_DAUCA_B2_protein_OS=Daucus_carota_PE=2_SV=1

Length=207

Subject= 5259-979_5_ORF3
>sp|P37707|B2_DAUCA_B2_protein_OS=Daucus_carota_PE=2_SV=1|||1e-27

Length=367
```

```
 Score = 95.1 bits (235),  Expect = 1e-27, Method: Compositional
matrix adjust.
 Identities = 50/137 (36%), Positives = 79/137 (58%), Gaps = 7/137
(5%)

Query  68
LPRNETVGGYIFVCNNDTMQENLKRQLFGLPPRYRDSVRAITPGLPLFLYNYSTHQLHGV  127
            LP  +   G IF+CN+ T ++    ++ GLP   RD V  I PG  LFLY++
+L+G+
Sbjct  36
LPPRKGPAGLIFMCNSKTKRDCFHYKVLGLPLAKRDLVEQIVPGTFLFLYDFDARELYGI  95

Query  128  FEAASFGGTNIDPTAWEDKKNQGESRFPAQVRVMTRKICEPLEEDSFRPIL--
HHYDGPK  185
            +EA+S GG N++P A+E     G+  +PAQVR    + C PL ED  R  +  ++Y
+
Sbjct  96   YEASSHGGVNLEPKAFE-----
GQGNYPAQVRFDIHRECLPLSEDLLRDAIKENYYARNR  150

Query  186  FRLELNIPEAISLLDIF  202
            F++EL   +   L+ +F
Sbjct  151  FQIELTSDQVSRLIQLF  167


 Score = 16.9 bits (32),  Expect = 0.53, Method: Compositional
matrix adjust.
 Identities = 6/8 (75%), Positives = 6/8 (75%), Gaps = 0/8 (0%)

Query  173  SFRPILHH  180
            SFRP  HH
Sbjct  354  SFRPSGHH  361



Lambda      K        H        a         alpha
   0.314    0.134    0.397    0.792     4.96

Gapped
Lambda      K        H        a         alpha    sigma
   0.267   0.0410    0.140     1.90     42.6     43.6

Effective search space used: 75780




Query= sp|P37707|B2_DAUCA_B2_protein_OS=Daucus_carota_PE=2_SV=1

Length=207

Subject= 5262-979_5_ORF3
>sp|P37707|B2_DAUCA_B2_protein_OS=Daucus_carota_PE=2_SV=1|||9e-28
```

```
Length=361


 Score = 95.5 bits (236),  Expect = 1e-27, Method: Compositional
matrix adjust.
 Identities = 50/137 (36%), Positives = 79/137 (58%), Gaps = 7/137
(5%)

Query  68
LPRNETVGGYIFVCNNDTMQENLKRQLFGLPPRYRDSVRAITPGLPLFLYNYSTHQLHGV  127
            LP  +   G IF+CN+ T ++    ++ GLP   RD V  I PG  LFLY++
+L+G+
Sbjct  30
LPPRKGPAGLIFMCNSKTKRDCFHYKVLGLPLAKRDLVEQIVPGTFLFLYDFDARELYGI  89

Query  128  FEAASFGGTNIDPTAWEDKKNQGESRFPAQVRVMTRKICEPLEEDSFRPIL--
HHYDGPK  185
            +EA+S GG N++P A+E     G+  +PAQVR    + C PL ED  R  +  ++Y
+
Sbjct  90   YEASSHGGVNLEPKAFE-----
GQGNYPAQVRFDIHRECLPLSEDLLRDAIKENYYARNR  144

Query  186  FRLELNIPEAISLLDIF  202
            F++EL   +   L+ +F
Sbjct  145  FQIELTSDQVSRLIQLF  161


 Score = 16.9 bits (32),  Expect = 0.51, Method: Compositional
matrix adjust.
 Identities = 6/8 (75%), Positives = 6/8 (75%), Gaps = 0/8 (0%)

Query  173  SFRPILHH  180
            SFRP  HH
Sbjct  348  SFRPSGHH  355



Lambda      K        H        a         alpha
   0.314    0.134    0.397    0.792     4.96

Gapped
Lambda      K        H        a         alpha    sigma
   0.267   0.0410    0.140     1.90     42.6     43.6

Effective search space used: 75780




Query= sp|P37707|B2_DAUCA_B2_protein_OS=Daucus_carota_PE=2_SV=1

Length=207

Subject= 5264-979_5_ORF3
```

```
>sp|P37707|B2_DAUCA_B2_protein_OS=Daucus_carota_PE=2_SV=1|||1e-27

Length=374


 Score = 95.5 bits (236),  Expect = 1e-27, Method: Compositional
matrix adjust.
 Identities = 50/137 (36%), Positives = 79/137 (58%), Gaps = 7/137
(5%)

Query  68
LPRNETVGGYIFVCNNDTMQENLKRQLFGLPPRYRDSVRAITPGLPLFLYNYSTHQLHGV  127
            LP  +   G IF+CN+ T ++    ++ GLP   RD V  I PG  LFLY++
+L+G+
Sbjct  43
LPPRKGPAGLIFMCNSKTKRDCFHYKVLGLPLAKRDLVEQIVPGTFLFLYDFDARELYGI  102

Query  128  FEAASFGGTNIDPTAWEDKKNQGESRFPAQVRVMTRKICEPLEEDSFRPIL--
HHYDGPK  185
            +EA+S GG N++P A+E     G+  +PAQVR    + C PL ED  R  +  ++Y
+
Sbjct  103  YEASSHGGVNLEPKAFE-----
GQGNYPAQVRFDIHRECLPLSEDLLRDAIKENYYARNR  157

Query  186  FRLELNIPEAISLLDIF  202
            F++EL   +   L+ +F
Sbjct  158  FQIELTSDQVSRLIQLF  174


 Score = 16.9 bits (32),  Expect = 0.54, Method: Compositional
matrix adjust.
 Identities = 6/8 (75%), Positives = 6/8 (75%), Gaps = 0/8 (0%)

Query  173  SFRPILHH  180
            SFRP  HH
Sbjct  361  SFRPSGHH  368



Lambda      K        H        a         alpha
   0.314    0.134    0.397    0.792     4.96

Gapped
Lambda      K        H        a         alpha    sigma
   0.267   0.0410    0.140     1.90     42.6     43.6

Effective search space used: 75780



Query= sp|P37707|B2_DAUCA_B2_protein_OS=Daucus_carota_PE=2_SV=1

Length=207
```

```
Subject= 5265-979_6_ORF3
>sp|P37707|B2_DAUCA_B2_protein_OS=Daucus_carota_PE=2_SV=1|||1e-27

Length=370


 Score = 95.1 bits (235),  Expect = 1e-27, Method: Compositional
matrix adjust.
 Identities = 50/137 (36%), Positives = 79/137 (58%), Gaps = 7/137
(5%)

Query  68
LPRNETVGGYIFVCNNDTMQENLKRQLFGLPPRYRDSVRAITPGLPLFLYNYSTHQLHGV  127
            LP  +   G IF+CN+ T ++    ++ GLP   RD V  I PG  LFLY++
+L+G+
Sbjct  36
LPPRKGPAGLIFMCNSKTKRDCFHYKVLGLPLAKRDLVEQIVPGTFLFLYDFDARELYGI  95

Query  128  FEAASFGGTNIDPTAWEDKKNQGESRFPAQVRVMTRKICEPLEEDSFRPIL--
HHYDGPK  185
            +EA+S GG N++P A+E     G+  +PAQVR    + C PL ED  R  +  ++Y
+
Sbjct  96   YEASSHGGVNLEPKAFE-----
GQGNYPAQVRFDIHRECLPLSEDLLRDAIKENYYARNR  150

Query  186  FRLELNIPEAISLLDIF  202
            F++EL   +   L+ +F
Sbjct  151  FQIELTSDQVSRLIQLF  167


 Score = 16.5 bits (31),  Expect = 0.54, Method: Compositional
matrix adjust.
 Identities = 6/8 (75%), Positives = 6/8 (75%), Gaps = 0/8 (0%)

Query  173  SFRPILHH  180
            SFRP  HH
Sbjct  354  SFRPSGHH  361



Lambda      K        H        a         alpha
   0.314    0.134    0.397    0.792     4.96

Gapped
Lambda      K        H        a         alpha    sigma
   0.267   0.0410    0.140     1.90     42.6     43.6

Effective search space used: 75780



Query= sp|P37707|B2_DAUCA_B2_protein_OS=Daucus_carota_PE=2_SV=1
```

```
Length=207

Subject= 5269-979_6_ORF3
>sp|P37707|B2_DAUCA_B2_protein_OS=Daucus_carota_PE=2_SV=1|||1e-27

Length=362


 Score = 95.1 bits (235),  Expect = 1e-27, Method: Compositional
matrix adjust.
 Identities = 50/137 (36%), Positives = 79/137 (58%), Gaps = 7/137
(5%)

Query  68
LPRNETVGGYIFVCNNDTMQENLKRQLFGLPPRYRDSVRAITPGLPLFLYNYSTHQLHGV  127
            LP  +   G IF+CN+ T ++    ++ GLP   RD V  I PG  LFLY++
+L+G+
Sbjct  28
LPPRKGPAGLIFMCNSKTKRDCFHYKVLGLPLAKRDLVEQIVPGTFLFLYDFDARELYGI  87

Query  128  FEAASFGGTNIDPTAWEDKKNQGESRFPAQVRVMTRKICEPLEEDSFRPIL--
HHYDGPK  185
            +EA+S GG N++P A+E     G+  +PAQVR    + C PL ED  R  +  ++Y
+
Sbjct  88   YEASSHGGVNLEPKAFE-----
GQGNYPAQVRFDIHRECLPLSEDLLRDAIKENYYARNR  142

Query  186  FRLELNIPEAISLLDIF  202
            F++EL   +   L+ +F
Sbjct  143  FQIELTSDQVSRLIQLF  159


 Score = 16.5 bits (31),  Expect = 0.53, Method: Compositional
matrix adjust.
 Identities = 6/8 (75%), Positives = 6/8 (75%), Gaps = 0/8 (0%)

Query  173  SFRPILHH  180
            SFRP  HH
Sbjct  346  SFRPSGHH  353



Lambda      K        H        a         alpha
   0.314    0.134    0.397    0.792     4.96

Gapped
Lambda      K        H        a         alpha    sigma
   0.267   0.0410    0.140     1.90     42.6     43.6

Effective search space used: 75780
```

Query= sp|P37707|B2_DAUCA_B2_protein_OS=Daucus_carota_PE=2_SV=1

Length=207

Subject= 5270-979_6_ORF4
>sp|P37707|B2_DAUCA_B2_protein_OS=Daucus_carota_PE=2_SV=1|||1e-27

Length=364

```
 Score = 95.5 bits (236),  Expect = 1e-27, Method: Compositional
matrix adjust.
 Identities = 50/137 (36%), Positives = 79/137 (58%), Gaps = 7/137
(5%)

Query  68
LPRNETVGGYIFVCNNDTMQENLKRQLFGLPPRYRDSVRAITPGLPLFLYNYSTHQLHGV  127
            LP  +   G IF+CN+ T ++    ++ GLP   RD V  I PG  LFLY++
+L+G+
Sbjct  30
LPPRKGPAGLIFMCNSKTKRDCFHYKVLGLPLAKRDLVEQIVPGTFLFLYDFDARELYGI  89

Query  128  FEAASFGGTNIDPTAWEDKKNQGESRFPAQVRVMTRKICEPLEEDSFRPIL--
HHYDGPK  185
            +EA+S GG N++P A+E     G+  +PAQVR    + C PL ED  R  +  ++Y
+
Sbjct  90   YEASSHGGVNLEPKAFE-----
GQGNYPAQVRFDIHRECLPLSEDLLRDAIKENYYARNR  144

Query  186  FRLELNIPEAISLLDIF  202
            F++EL   +   L+ +F
Sbjct  145  FQIELTSDQVSRLIQLF  161


 Score = 16.9 bits (32),  Expect = 0.53, Method: Compositional
matrix adjust.
 Identities = 6/8 (75%), Positives = 6/8 (75%), Gaps = 0/8 (0%)

Query  173  SFRPILHH  180
            SFRP  HH
Sbjct  348  SFRPSGHH  355



Lambda      K        H        a         alpha
   0.314    0.134    0.397    0.792     4.96

Gapped
Lambda      K        H        a         alpha    sigma
   0.267   0.0410    0.140     1.90     42.6     43.6

Effective search space used: 75780
```

```
Matrix: BLOSUM62
Gap Penalties: Existence: 11, Extension: 1
Neighboring words threshold: 11
Window for multiple hits: 40
```

```
Query= sp|P42761|GSTFA_ARATH_Glutathione_S-
transferase_F10_OS=Arabidopsis_thaliana_GN=GSTF10_PE=1_SV=3

Length=215

Subject= 11251-715_6_ORF1
>sp|P42761|GSTFA_ARATH_Glutathione_S-
transferase_F10_OS=Arabidopsis_thaliana_GN=GSTF10_PE=1_SV=3|||4e-70

Length=226


 Score =   204 bits (520),  Expect = 4e-70, Method: Compositional
matrix adjust.
 Identities = 102/205 (50%), Positives = 132/205 (64%), Gaps = 0/205
(0%)

Query  8
PLFASSKRAVVTLVEKGVSFETVNVDLMKGEQRQPEYLAIQPFGKIPVLVDGDYKIFESR  67
           P    + R + TL EK V FE + V+LMKGE +QP +LA+QPFG IPVL D D  +
+ESR
Sbjct  21
PRSTCTMRVLATLSEKDVPFELLFVNLMKGEHKQPPFLALQPFGLIPVLQDEDLTLYESR  80

Query  68
AIMRYIAEKYRSQGPDLLGKTIEERGQVEQWLDVEATSYHPPLLALTLNIVFAPLMGFPA  127
           AI RY+AEKY+ QG  L G TI ER  +EQW++VE  +Y P    +   +V  P+
G P
Sbjct  81
AIARYVAEKYQKQGASLYGSTIAERALIEQWIEVEGQNYTPAAQPIFYQLVIGPMRGVPT  140

Query  128
DEKVIKESEEKLAEVLDVYEAQLSKNEYLAGDFVSLADLAHLPFTEYLVGPIGKAHLIKD  187
           D  V++ES  K  +VLD+YE +L K  YLAG   SLAD+ H+P TEYLV      A
Sbjct  141
DNVVVEESLVKFEKVLDIYEERLGKAPYLAGKSFSLADVTHMPLTEYLVNQPKVAAAFHS  200

Query  188  RKHVSAWWDKISSRAAWKEVSAKYS  212
            RK+V AWW++IS+R AWK+V A  S
Sbjct  201  RKNVMAWWERISARPAWKKVVAMSS  225
```

```
Lambda      K        H        a         alpha
   0.318    0.135    0.393    0.792     4.96

Gapped
Lambda      K        H        a         alpha    sigma
   0.267   0.0410    0.140     1.90     42.6     43.6

Effective search space used: 39372



Query= sp|P42761|GSTFA_ARATH_Glutathione_S-
transferase_F10_OS=Arabidopsis_thaliana_GN=GSTF10_PE=1_SV=3

Length=215

Subject= 294468-71_2_ORF1
>sp|P42761|GSTFA_ARATH_Glutathione_S-
transferase_F10_OS=Arabidopsis_thaliana_GN=GSTF10_PE=1_SV=3|||2e-80

Length=240


 Score =   232 bits (591),  Expect = 2e-80, Method: Compositional
matrix adjust.
 Identities = 106/197 (54%), Positives = 142/197 (72%), Gaps = 0/197
(0%)

Query  11
ASSKRAVVTLVEKGVSFETVNVDLMKGEQRQPEYLAIQPFGKIPVLVDGDYKIFESRAIM  70
              + R + T +EK   FE   VDL KG  +QP +LA+QPFG IPVL DGD
+IFESRAI
Sbjct  37
TCTGRVMTTALEKDAPFEIETVDLSKGAHKQPHFLAMQPFGVIPVLEDGDLRIFESRAIA  96

Query  71
RYIAEKYRSQGPDLLGKTIEERGQVEQWLDVEATSYHPPLLALTLNIVFAPLMGFPADEK  130
            RYIA KY  QG  L GKT++++ +VEQWL+VE+ +Y+PP+  +   +VF P    P
E+
Sbjct  97
RYIATKYEEQGTPLYGKTLQQKAKVEQWLEVESQNYNPPISTIVGQLVFRPRYKLPTQEE  156

Query  131
VIKESEEKLAEVLDVYEAQLSKNEYLAGDFVSLADLAHLPFTEYLVGPIGKAHLIKDRKH  190
            V+KE+  KL +VLD+YEA L+KN+YLAGDF SLADL+H+P+T YL+    K  +I
RKH
Sbjct  157
VVKENLGKLEKVLDIYEAHLAKNQYLAGDFFSLADLSHIPYTHYLIHAAKKGDVITSRKH  216

Query  191  VSAWWDKISSRAAWKEV  207
            V+AWW++ISSR +WK+V
Sbjct  217  VNAWWERISSRPSWKKV  233
```

```
 Score = 13.9 bits (24),  Expect = 2.7, Method: Compositional matrix
adjust.
 Identities = 5/12 (42%), Positives = 8/12 (67%), Gaps = 0/12 (0%)

Query  200  SRAAWKEVSAKY  211
            SRA  + ++ KY
Sbjct  92   SRAIARYIATKY  103



Lambda      K        H        a         alpha
   0.318    0.135    0.393    0.792     4.96

Gapped
Lambda      K        H        a         alpha    sigma
   0.267   0.0410    0.140     1.90     42.6     43.6

Effective search space used: 39372




Query= sp|P42761|GSTFA_ARATH_Glutathione_S-
transferase_F10_OS=Arabidopsis_thaliana_GN=GSTF10_PE=1_SV=3

Length=215

Subject= 3863-1099_6_ORF1
>sp|P42761|GSTFA_ARATH_Glutathione_S-
transferase_F10_OS=Arabidopsis_thaliana_GN=GSTF10_PE=1_SV=3|||3e-70

Length=225


 Score =   205 bits (521),  Expect = 3e-70, Method: Compositional
matrix adjust.
 Identities = 100/202 (50%), Positives = 134/202 (66%), Gaps = 1/202
(0%)

Query  8
PLFASSKRAVVTLVEKGVSFETVNVDLMKGEQRQPEYLAIQPFGKIPVLVDGDYKIFESR  67
            P    + R + TL EK V FE + V+LMKGE +QP +LA+QPFG IPVL D D  +
+ESR
Sbjct  21
PRSTCTMRVLATLSEKDVPFELLFVNLMKGEHKQPPFLALQPFGLIPVLQDEDLTLYESR  80

Query  68
AIMRYIAEKYRSQGPDLLGKTIEERGQVEQWLDVEATSYHPPLLALTLNIVFAPLMGFPA  127
            AI RY+AEKY+ +G  L G TI ER  +EQW++VE+ +Y P +  +   ++  P+
G P
Sbjct  81   AIARYVAEKYK-
EGTPLYGSTISERALIEQWIEVESQNYSPTVQPIIYQLIIHPMRGLPT  139
```

```
Query  128
DEKVIKESEEKLAEVLDVYEAQLSKNEYLAGDFVSLADLAHLPFTEYLVGPIGKAHLIKD  187
            D+ V++    K  ++LD+YE QL K  YLAGD  SLADL H+P T+YL+      A
L
Sbjct  140
DDSVVEAGLAKFEKILDIYEKQLGKTPYLAGDSFSLADLTHMPQTQYLISHPKVAGLFHA  199

Query  188  RKHVSAWWDKISSRAAWKEVSA  209
            RK++ AWWDKISSR AWK+V A
Sbjct  200  RKNIMAWWDKISSRPAWKKVLA  221


 Score = 13.9 bits (24),  Expect = 2.5, Method: Compositional matrix
adjust.
 Identities = 6/12 (50%), Positives = 8/12 (67%), Gaps = 0/12 (0%)

Query  200  SRAAWKEVSAKY  211
            SRA  + V+ KY
Sbjct  79   SRAIARYVAEKY  90



Lambda      K        H        a         alpha
   0.318    0.135    0.393    0.792     4.96

Gapped
Lambda      K        H        a         alpha    sigma
   0.267   0.0410    0.140     1.90     42.6     43.6

Effective search space used: 39372




Query= sp|P42761|GSTFA_ARATH_Glutathione_S-
transferase_F10_OS=Arabidopsis_thaliana_GN=GSTF10_PE=1_SV=3

Length=215

Subject= 3873-1098_5_ORF2
>sp|P42761|GSTFA_ARATH_Glutathione_S-
transferase_F10_OS=Arabidopsis_thaliana_GN=GSTF10_PE=1_SV=3|||5e-70

Length=282


 Score =   207 bits (526),  Expect = 4e-70, Method: Compositional
matrix adjust.
 Identities = 99/202 (49%), Positives = 133/202 (66%), Gaps = 1/202
(0%)

Query  8
PLFASSKRAVVTLVEKGVSFETVNVDLMKGEQRQPEYLAIQPFGKIPVLVDGDYKIFESR  67
```

```
            P    ++R +  L EK V FE V V++M  E +QP +LA+QPFG IPVL DGD 
+FESR
Sbjct  78   
PRSTCTRRVLAALSEKNVDFEVVLVNMMAAEHKQPPFLALQPFGVIPVLQDGDLTLFESR  137

Query  68   
AIMRYIAEKYRSQGPDLLGKTIEERGQVEQWLDVEATSYHPPLLALTLNIVFAPLMGFPA  127
            AI RY+AEKY+ +G  L G TI ER  +EQW++VE+ +Y P +  +   ++  P+ 
G P
Sbjct  138  AIARYVAEKYK-
EGTPLYGSTISERALIEQWIEVESQNYSPTVQPIFYQLIIHPMRGLPT  196

Query  128  
DEKVIKESEEKLAEVLDVYEAQLSKNEYLAGDFVSLADLAHLPFTEYLVGPIGKAHLIKD  187
            D+ V++    K  ++LD+YE QL K  YLAGD  SLADL H+P T+YL+     A 
L
Sbjct  197  
DDSVVEAGLAKFEKILDIYEKQLGKTPYLAGDSFSLADLTHMPQTQYLISHPKVAGLFHA  256

Query  188  RKHVSAWWDKISSRAAWKEVSA  209
            RK++ AWWDKISSR AWK+V A
Sbjct  257  RKNIMAWWDKISSRPAWKKVLA  278


 Score = 14.2 bits (25),  Expect = 3.0, Method: Compositional matrix 
adjust.
 Identities = 6/12 (50%), Positives = 8/12 (67%), Gaps = 0/12 (0%)

Query  200  SRAAWKEVSAKY  211
            SRA  + V+ KY
Sbjct  136  SRAIARYVAEKY  147



Lambda      K        H        a         alpha
   0.318    0.135    0.393    0.792     4.96

Gapped
Lambda      K        H        a         alpha    sigma
   0.267   0.0410    0.140     1.90     42.6     43.6

Effective search space used: 39372




Query= sp|P42761|GSTFA_ARATH_Glutathione_S-
transferase_F10_OS=Arabidopsis_thaliana_GN=GSTF10_PE=1_SV=3

Length=215

Subject= 429352-5_4_ORF1
>sp|P42761|GSTFA_ARATH_Glutathione_S-
transferase_F10_OS=Arabidopsis_thaliana_GN=GSTF10_PE=1_SV=3|||1e-82
```

```
Length=229


 Score =   236 bits (602),  Expect = 2e-82, Method: Compositional
matrix adjust.
 Identities = 107/196 (55%), Positives = 146/196 (74%), Gaps = 0/196
(0%)

Query  12
SSKRAVVTLVEKGVSFETVNVDLMKGEQRQPEYLAIQPFGKIPVLVDGDYKIFESRAIMR  71
             + RA+ T  EK    E   +D++ G  +QP YLA+QPFG+IPVL DGD +
+FESRAI R
Sbjct  27
CTGRALATAFEKDAPVELETIDVLNGAHKQPAYLALQPFGQIPVLEDGDLRVFESRAIAR  86

Query  72
YIAEKYRSQGPDLLGKTIEERGQVEQWLDVEATSYHPPLLALTLNIVFAPLMGFPADEKV  131
            YIA KY  QG  L GKT++++ +VEQWL+VE+ +Y+PP+  +   IVF P+ G P
+E+V
Sbjct  87
YIATKYEEQGTPLYGKTLQDKAKVEQWLEVESQNYNPPISTIVAQIVFRPMHGLPVEEEV  146

Query  132
IKESEEKLAEVLDVYEAQLSKNEYLAGDFVSLADLAHLPFTEYLVGPIGKAHLIKDRKHV  191
            +KE+ EKL +VLD+YEA L+K+EYLAGDF SLADL+HLP+T YL+    K  +I
RKHV
Sbjct  147
VKENLEKLEKVLDIYEAHLAKHEYLAGDFFSLADLSHLPYTHYLILAAKKGDVITCRKHV  206

Query  192  SAWWDKISSRAAWKEV  207
            +AWW++ISSR +WK+V
Sbjct  207  NAWWERISSRPSWKKV  222


 Score = 13.9 bits (24),  Expect = 2.9, Method: Compositional matrix
adjust.
 Identities = 5/12 (42%), Positives = 8/12 (67%), Gaps = 0/12 (0%)

Query  200  SRAAWKEVSAKY  211
            SRA  + ++ KY
Sbjct  81   SRAIARYIATKY  92



Lambda      K        H        a         alpha
   0.318    0.135    0.393    0.792     4.96

Gapped
Lambda      K        H        a         alpha    sigma
   0.267   0.0410    0.140     1.90     42.6     43.6

Effective search space used: 39372
```

```
Matrix: BLOSUM62
Gap Penalties: Existence: 11, Extension: 1
Neighboring words threshold: 11
Window for multiple hits: 40
```

```
Query= sp|P42825|
DNAJ2_ARATH_Chaperone_protein_dnaJ_2_OS=Arabidopsis_thalia
na_GN=ATJ2_PE=1_SV=2

Length=419

Subject= 101113-248_5_ORF2
>sp|P42825|DNAJ2_ARATH_Chaperone_protein_dnaJ_2_OS=Arabidopsis_thali
ana_GN=ATJ2_PE=1_SV=2|||0

Length=433


 Score =   579 bits (1492),  Expect = 0.0, Method: Compositional
matrix adjust.
 Identities = 308/424 (73%), Positives = 363/424 (86%), Gaps =
10/424 (2%)

Query  1
MFGRGPSRKSDNTKFYEILGVPKTAAPEDLKKAYKKAAIKNHPDKGGDPEKFKELAQAYE  60
            MFGR P +KS+NT++YEILGVPK A+ ++LKKAY
+KAAIKNHPDKGGDPEKFKELAQAYE
Sbjct  15   MFGRAP-
KKSNNTRYYEILGVPKNASADELKKAYRKAAIKNHPDKGGDPEKFKELAQAYE  73

Query  61
VLSDPEKREIYDQYGEDALKEGMGGGGGHDPFDIFSSFFGSGGHPFGSHSRGRRQRRG-  119
            VLSD EKR+IYDQYGEDA+KEGMGGGG GH+PFDIF SFFG G  PFG  S
+R+
Sbjct  74   VLSDAEKRDIYDQYGEDAIKEGMGGGGEGHNPFDIFESFFG-
GASPFGGGSSRGGRRQRR  132

Query  120  -
EDVVHPLKVSLEDVYLGTTKKLSLSRKALCSKCNGKGSKSGASMKCGGCQGSGMKISIR  178
             EDVVHPLKVSLE++Y GT+KKLSLSR  LCSKC G GSKSG+S KC GCQGSGMK
+SIR
Sbjct  133
GEDVVHPLKVSLEELYNGTSKKLSLSRNVLCSKCKGSGSKSGSSAKCFGCQGSGMKVSIR  192

Query  179
QFGPGMMQQVQHACNDCKGTGETINDRDRCPQCKGEKVVSEKKVLEVNVEKGMQHNQKIT  238
            Q GPGM+QQ+QH C DCKG+GETIN++D+CPQCKGEKVV EKKVLEV+VEKGM
```

```
+HNQKIT
Sbjct  193  
QLGPGMIQQMQHVCPDCKGSGETINEKDKCPQCKGEKVVQEKKVLEVHVEKGMKHNQKIT  252

Query  239  
FSGQADEAPDTVTGDIVFVIQQKEHPKFKRKGEDLFVEHTISLTEALCGFQFVLTHLDKR  298
            F+G+ADEAPDTVTGDIVFV+QQK+H KFKRKG+DLFVEH+++L EALCG+QF+
+THLD R
Sbjct  253  
FAGEADEAPDTVTGDIVFVLQQKDHAKFKRKGDDLFVEHSLTLCEALCGYQFIITHLDGR  312

Query  299  QLLIKSKPGEVVKPDSYKAISDEGMPIYQRPFMKGKLYIHFTVEFPE--
SLSPDQTKAIE  356
            QLLIKS P E+VKP  +KAI+DEGMPIYQRPFMKGKLYIHF+VEFPE  SL+ +Q
K +E
Sbjct  313  
QLLIKSSPSEIVKPGQFKAINDEGMPIYQRPFMKGKLYIHFSVEFPESGSLTLEQCKLLE  372

Query  357  AVLPKPTKAAISDMEIDDCEETTLHDVNIEDEMKRKAQAQR-
EAYDDDEEDHPGGAQRVQ  415
            A+LP      ++DME+D+CEET L DVN+E+EM+RK Q  R EAYD++EE
+VQ
Sbjct  373  AILPPKPANEMTDMELDECEETILQDVNMEEEMRRKQQQSRQEAYDEEEE---
SAGPQVQ  429

Query  416  CAQQ  419
            CAQQ
Sbjct  430  CAQQ  433



Lambda      K        H        a         alpha
   0.315    0.134    0.396    0.792     4.96

Gapped
Lambda      K        H        a         alpha    sigma
   0.267   0.0410    0.140     1.90     42.6     43.6

Effective search space used: 155187




Matrix: BLOSUM62
Gap Penalties: Existence: 11, Extension: 1
Neighboring words threshold: 11
Window for multiple hits: 40




Query= sp|P46416|GSHB_ARATH_Glutathione_synthetase,
_chloroplastic_OS=Arabidopsis_thaliana_GN=GSH2_PE=2_SV=3
```

```
Length=539

Subject= 12699-677_1_ORF2
>sp|P46416|GSHB_ARATH_Glutathione_synthetase,
_chloroplastic_OS=Arabidopsis_thaliana_GN=GSH2_PE=2_SV=3|||0

Length=545


 Score =   553 bits (1426),  Expect = 0.0, Method: Compositional
matrix adjust.
 Identities = 263/468 (56%), Positives = 349/468 (75%), Gaps = 6/468
(1%)

Query  77
EFVQKLVYDALVWSSLHGLVVGDKSYQKSGNVPGVGLMHAPIALLPTAFPEAYWKQACNV  136
            E  +++  +ALVW+SLHG+++GDKS + SG  PG GL+HAPI+LLP+ F E +
+KQA  +
Sbjct  79
ELAEQIAPEALVWASLHGILMGDKSIETSGTTPGTGLVHAPISLLPSPFLEEHFKQAVEL  138

Query  137
TPLFNELIDRVSLDGKFLQDSLSRTKKVDVFTSRLLDIHSKMLERNKKEDIRLGLHRFDY  196
             PLFNEL+DRVS+D KFLQDSLS TK+ D FT+RLL+IHS +L    K+DI
+LGLHR DY
Sbjct  139
APLFNELVDRVSMDHKFLQDSLSMTKQADFFTARLLEIHSLVLHEGIKQDIQLGLHRSDY  198

Query  197
MLDEETNSLLQIEMNTISCSFPGLSRLVSQLHQSLLRSYGDQIGIDSERVPINTSTIQFA  256
            M D  T  LLQ+E+NTIS SF GL   VS LH+ LL   G +  + S+++P N +
FA
Sbjct  199
MADMRTGDLLQVEINTISSSFAGLGSQVSLLHRYLLDLIGKRSNLSSKKIPENEAADGFA  258

Query  257
DALAKAWLEYSNPRAVVMVIVQPEERNMYDQHLLSSILREKHNIVVIRKTLAEVEKEGSV  316
             A+A A+ E+ + RAVV+++VQP ERNMYDQ+ LS+ L EK+ I VIR+TL E
EG +
Sbjct  259
KAMAMAFKEFGDSRAVVLMVVQPGERNMYDQYWLSTKLYEKYGIFVIRRTLTEACNEGVL  318

Query  317
QEDETLIVGGQAVAVVYFRSGYTPNDHPSESEWNARLLIEESSAVKCPSIAYHLTGSKKI  376
              D T  +G Q VA+VY+R+GY P D+PSE EW+AR L+E S+A+KCPSI YHL G
+KKI
Sbjct  319
HPDGTFAIGKQTVALVYYRAGYDPKDYPSEIEWSARTLMERSNAIKCPSITYHLAGTKKI  378

Query  377  QQELAKPGVLERFLDNKEDIAKLRKCFAGLWSLDD---
SEIVKQAIEKPGLFVMKPQREG  433
            QQELAKPGVLER++   + + K+RKCFAGLW  DD   SEI+K+A+  P  FV
+KPQREG
```

```
Sbjct  379
QQELAKPGVLERYVQETDAVEKIRKCFAGLWGFDDNKESEIIKEALRSPDAFVLKPQREG  438

Query  434  GGNNIYGDDVRENLLRLQKEGEEGN--
AAYILMQRIFPKVSNMFLVREGVYHKHQAISEL  491
            GGNN++G+D+R+ L  L KEG   +  AAYILMQRIFP V N + +R G      +
+SEL
Sbjct  439  GGNNLFGEDIRKKLEEL-
KEGRTADSFAAYILMQRIFPPVHNTYFMRGGKLIAQGSVSEL  497

Query  492  GVYGAYLRSKDEVIVNEQSGYLMRTKIASSDEGGVAAGFGVLDSIYLI  539
            G++  Y+R+KD V++NEQ+GYL+RTK + ++EGGVAAGF VLDS+YL+
Sbjct  498  GIFSTYVRNKDNVVLNEQAGYLLRTKASDTNEGGVAAGFAVLDSVYLV  545



Lambda      K        H        a         alpha
   0.317    0.133    0.380    0.792     4.96

Gapped
Lambda      K        H        a         alpha    sigma
   0.267   0.0410    0.140     1.90     42.6     43.6

Effective search space used: 257040




Matrix: BLOSUM62
Gap Penalties: Existence: 11, Extension: 1
Neighboring words threshold: 11
Window for multiple hits: 40





Query= sp|P46518|
LEA14_GOSHI_Late_embryogenesis_abundant_protein_Lea14-
A_OS=Gossypium_hirsutum_GN=LEA14-A_PE=2_SV=1

Length=151

Subject= 319929-58_1_ORF2
>sp|P46518|LEA14_GOSHI_Late_embryogenesis_abundant_protein_Lea14-
A_OS=Gossypium_hirsutum_GN=LEA14-A_PE=2_SV=1|||1e-23

Length=327


 Score = 81.6 bits (200),  Expect = 1e-23, Method: Compositional
matrix adjust.
 Identities = 48/147 (33%), Positives = 76/147 (52%), Gaps = 1/147
(1%)
```

```
Query  4
LLEKAKDFVVDKVANIKKPEASVSDVDLKHVSRECVEYGAKVSVSNPYSHSIPICEISYN  63
               K K F+ D +    KP A VS   L  ++ E  +    + VSNP    IP+
+I Y
Sbjct  37   FFSKVKHFIKDSIG-
FGKPSAEVSGFHLPSITLEKADVVVDLLVSNPNPVPIPLVDIVYL  95

Query  64
FRSAGRGIASGTIPDPGSLKASDTTMLDVPVKVPYNILVSLVKDIGADWDIDYELELGLT  123
              S GR + SGTIPD G++ A  +  + +PV + Y  +     DI     I Y ++
+ L
Sbjct  96
IESDGRKLVSGTIPDAGTIHAHGSETIKIPVTLVYQDIKDTYDDIQPGDVIPYRVKIELI  155

Query  124  IDLPIVGNFTIPLSQKGEIKLPTLSDI  150
            +D+P++G  T+PL + G+I +P   D+
Sbjct  156  VDVPVLGRLTLPLEKTGDIPIPYKPDV  182


 Score = 40.8 bits (94),  Expect = 3e-09, Method: Compositional
matrix adjust.
 Identities = 30/120 (25%), Positives = 49/120 (41%), Gaps = 1/120
(1%)

Query  21
KPEASVSDVDLKHVSRECVEYGAKVSVSNPYSHSIPICEISYNFRSAGRGIASGTIPDPG  80
            KP+  V  ++  H+S E       + V N     + I  + Y F  A   IA+ T+
Sbjct  179
KPDVDVDKIEFDHLSMEETSASLHLKVENKNKFDLGITALDYTFTLADATIANATLSRSA  238

Query  81
SLKASDTTMLDVPVKVPYNILVSLVKDIGADWDIDYELELGLTIDLPIVGNFTIPLSQKG  140
            ++       L++P+      L S V DI       Y +   L +D P  G   +P
S++G
Sbjct  239  NIAQCGEGTLEIPISFRPKDLGSAVWDIVRGRGAGYSMVGKLEVDTPF-
GPMHLPFSKEG  297



Lambda      K        H        a         alpha
   0.316    0.136    0.389    0.792     4.96

Gapped
Lambda      K        H        a         alpha    sigma
   0.267   0.0410    0.140     1.90     42.6     43.6

Effective search space used: 39345



Matrix: BLOSUM62
Gap Penalties: Existence: 11, Extension: 1
```

```
Neighboring words threshold: 11
Window for multiple hits: 40





Query= sp|P46519|
LEA14_SOYBN_Desiccation_protectant_protein_Lea14_homolog_O
S=Glycine_max_PE=2_SV=1

Length=152

Subject= 319597-58_1_ORF2
>sp|P46519|LEA14_SOYBN_Desiccation_protectant_protein_Lea14_homolog_
OS=Glycine_max_PE=2_SV=1|||9e-24

Length=326


 Score = 82.4 bits (202),  Expect = 9e-24, Method: Compositional
matrix adjust.
 Identities = 43/147 (29%), Positives = 82/147 (56%), Gaps = 1/147
(1%)

Query  4
LLDKAKNYVAEKVTNMPKPEASVTDVDFKRVSRDSVEYLAKVSVSNPYSTPIPICEIKYS  63
               K K+++ + +    KP A V+      ++ +  + +  + VSNP   PIP+
+I Y
Sbjct  37   FFSKVKHFIKDSI-
GFGKPSAEVSGFHLPSITLEKADVVVDLLVSNPNPVPIPLVDIVYL  95

Query  64
LKSAGKEIASGTIPDPGSLKASDTTMLDVPVKVPHSILLSLAKDIGADWDIDYQLDLGLV  123
            ++S G+++ SGTIPD G++ A  +  + +PV + +  +     DI     I Y++
+ L+
Sbjct  96
IESDGRKLVSGTIPDAGTIHAHGSETIKIPVTLVYQDIKDTYDDIQPGDVIPYRVKIELI  155

Query  124  IDLPVIGNFTIPLSQKGEIKLPTLSDM  150
            +D+PV+G  T+PL + G+I +P   D+
Sbjct  156  VDVPVLGRLTLPLEKTGDIPIPYKPDV  182


 Score = 34.7 bits (78),  Expect = 3e-07, Method: Compositional
matrix adjust.
 Identities = 30/130 (23%), Positives = 53/130 (41%), Gaps = 4/130
(3%)

Query  14   EKVTNMP---
KPEASVTDVDFKRVSRDSVEYLAKVSVSNPYSTPIPICEIKYSLKSAGKE  70
            EK  ++P   KP+  V  ++F  +S +       + V N     + I  + Y+
A
Sbjct  169
```

```
EKTGDIPIPYKPDVDVDKIEFDHLSMEETSASLHLKVENKNKFDLGITALDYTFTLADAT  228

Query  71
IASGTIPDPGSLKASDTTMLDVPVKVPHSILLSLAKDIGADWDIDYQLDLGLVIDLPVIG  130
            IA+ T+    ++       L++P+      L S   DI       Y +   L +D
P  G
Sbjct  229
IANATLSRSANIAQCGEGTLEIPISFRPKDLGSAVWDIVRGRGAGYSMVGKLEVDTP-FG  287

Query  131  NFTIPLSQKG  140
               +P S++G
Sbjct  288  PMHLPFSKEG  297



Lambda      K        H        a         alpha
   0.314    0.133    0.375    0.792     4.96

Gapped
Lambda      K        H        a         alpha    sigma
   0.267   0.0410    0.140     1.90     42.6     43.6

Effective search space used: 39520




Matrix: BLOSUM62
Gap Penalties: Existence: 11, Extension: 1
Neighboring words threshold: 11
Window for multiple hits: 40





Query= sp|P46633|HS90A_CRIGR_Heat_shock_protein_HSP_90-
alpha_OS=Cricetulus_griseus_GN=HSP90AA1_PE=2_SV=2

Length=733

Subject= 112918-233_2_ORF1
>sp|P46633|HS90A_CRIGR_Heat_shock_protein_HSP_90-
alpha_OS=Cricetulus_griseus_GN=HSP90AA1_PE=2_SV=2|||4e-44

Length=133


 Score =   142 bits (359),  Expect = 3e-44, Method: Compositional
matrix adjust.
 Identities = 70/132 (53%), Positives = 98/132 (74%), Gaps = 3/132
(2%)

Query  342  LFVPRRAPFDLFENRKKKNNIKLYVRRVFIMDNCE-
```

```
ELFPEYLNFIRGVVDSEDLPLNIS  400
            L++P  APF   E   K  NIKLYV+RVFI D+   ELFP YL+F++GVVDS
+DLPLN+S
Sbjct  1
LYIPGMAPFASDEASSKLKNIKLYVKRVFISDDFHGELFPRYLSFVKGVVDSDDLPLNVS  60

Query  401  REILQQSKILKVIRKNLVRKCLELFHELAE--
DKENYKKFYEQFSKNIKLGIHEDSQNRK  458
            RE+LQ+S+I+ ++RK L +K  +LF  +A+  DKE+YK+F+  + K IKLGI +D
N+K
Sbjct  61
REVLQESRIVNLMRKRLTKKSFDLFESIAKRPDKEDYKRFWRNYGKYIKLGITDDKDNQK  120

Query  459  KLSELLRYYTSA  470
            +L+  LRYY+S
Sbjct  121  RLAYFLRYYSST  132


 Score = 16.2 bits (30),  Expect = 1.1, Method: Compositional matrix
adjust.
 Identities = 6/13 (46%), Positives = 10/13 (77%), Gaps = 0/13 (0%)

Query  691  RMIKLGLGIDEDD  703
            + IKLG+  D+D+
Sbjct  106  KYIKLGITDDKDN  118



Lambda      K        H        a         alpha
   0.313    0.132    0.364    0.792     4.96

Gapped
Lambda      K        H        a         alpha    sigma
   0.267   0.0410    0.140     1.90     42.6     43.6

Effective search space used: 74836



Matrix: BLOSUM62
Gap Penalties: Existence: 11, Extension: 1
Neighboring words threshold: 11
Window for multiple hits: 40



Query= sp|P48417|CP74_LINUS_Allene_oxide_synthase,
_chloroplastic_OS=Linum_usitatissimum_GN=CYP74A_PE=1_SV=1

Length=536
```

Subject= 216793-124_3_ORF2
>sp|P48417|CP74_LINUS_Allene_oxide_synthase,
_chloroplastic_OS=Linum_usitatissimum_GN=CYP74A_PE=1_SV=1|||1e-136

Length=504

Score = 397 bits (1020), Expect = 2e-136, Method: Compositional matrix adjust.
Identities = 209/470 (44%), Positives = 293/470 (62%), Gaps = 17/470 (4%)

```
Query  57   ITSQPPPSSDETT---------
LPIRQIPGDYGLPGIGPIQDRLDYFYNQGREEFFKSRL  107
            IT   PPS    T         LP+R IPG YGLP +GP++DRLD+F+ QG +EF+
SR
Sbjct  14
ITMATPPSPSPATAAAPIPLEKLPLRDIPGGYGLPLLGPLKDRLDFFWFQGEKEFWASRR  73

Query  108
QKYKSTVYRANMPPGPFIASNPRVIVLLDAKSFPVLFDMSKVEKKDLFTGTYMPSTELTG  167
            QK+KSTV+R N+PP P   +  +VI+LLD KSFPVLFD  KV KKD+    YMP T
G
Sbjct  74
QKFKSTVFRTNVPPSPPAFAANKVIMLLDQKSFPVLFDTDKVYKKDVLLANYMPHTSYYG  133

Query  168
GYRILSYLDPSEPNHTKLKQLLFNLIKNRRDYVIPEFSSSFTDLCEVVEYDLATKGKA-A  226
            G R   YLDPSE  H KLK  + +L+K R    +PE   + T++    E  L+ K
+
Sbjct  134
GIRPCVYLDPSEERHPKLKSFIMSLLKARAKLWVPEMHKATTEIFASWEAKLSEKPEGFE  193

Query  227
FNDPAEQAAFNFLSRAFFGVKPIDTPLGKDAPSLISKWVLFNLAPILSVGLPKEVEEATL  286
             +    Q A N L R   G  P    L   +P+    W+   L P+ SVGLP
+EE  L
Sbjct  194  ISTETGQVALNVLIRTLLGQSPSKDDL---
SPTTFQVWLGPQLVPVASVGLPHLLEELIL  250

Query  287  HSVRLPPLLVQNDYHRLYEFFTSAAGSVLDEAE-
QSGISRDEACHNILFAVCFNSWGGFK  345
            H++R+P LLV   Y ++  +F      ++DEAE + GI R++A HNI+F   FN+
+GG
Sbjct  251
HNIRIPFLLVAFFYKKIESYFRKHGEKLIDEAEKEHGIDREDALHNIIFFTGFNAFGGLN  310

Query  346
ILFPSLMKWIGRAGLELHTKLAQEIRSAIQSTGGGKVTMAAMEQMPLMKSVVYETLRIEP  405
            IL P ++ ++GR G E+  ++A+E+R A+++  GGK+T+ A+ +MPL++S VYE
LR+EP
Sbjct  311  ILLPWIVSYVGREGEEVKAEMAREVREAVRAE-
GGKITVGAIMKMPLVRSAVYEVLRMEP  369
```

```
Query  406  PVALQYGKAKKDFILESHEAAYQVKEGEMLFGYQPFATKDPKIF-
DRPEEFVADRFVG-E  463
            PV  QYG AK D ++ESHEA Y+VK+GE++ G QP A +DP +F +  E + A
RFVG E
Sbjct  370
PVPYQYGIAKMDMVIESHEARYEVKKGEVIGGCQPVAVRDPVVFGESAERYKAGRFVGEE  429

Query  464  GVKLMEYVMWSNGPETETPSVANKQCAGKDFVVMAARLFVVELFKRYDSF  513
            G +L+++V+WSNGP     +V NKQCA  D V +  + F+  +F +YDSF
Sbjct  430  GERLLKHVLWSNGPADGEATVKNKQCAANDLVPLLGQAFLASIFLKYDSF  479



Lambda      K        H        a         alpha
   0.319    0.136    0.399    0.792     4.96

Gapped
Lambda      K        H        a         alpha    sigma
   0.267   0.0410    0.140     1.90     42.6     43.6

Effective search space used: 234969




Query= sp|P48417|CP74_LINUS_Allene_oxide_synthase,
_chloroplastic_OS=Linum_usitatissimum_GN=CYP74A_PE=1_SV=1

Length=536

Subject= 24435-507_4_ORF2
>sp|P48417|CP74_LINUS_Allene_oxide_synthase,
_chloroplastic_OS=Linum_usitatissimum_GN=CYP74A_PE=1_SV=1|||2e-146

Length=496


 Score =   423 bits (1087),  Expect = 2e-146, Method: Compositional
matrix adjust.
 Identities = 214/455 (47%), Positives = 295/455 (65%), Gaps = 8/455
(2%)

Query  64
SSDETTLPIRQIPGDYGLPGIGPIQDRLDYFYNQGREEFFKSRLQKYKSTVYRANMPPGP  123
            SSDE  LP+R IPG YG+P  G ++DRLD+F+ QG  +F+ SR+QK+ STV+R N+
PGP
Sbjct  21   SSDE--
LPLRAIPGSYGMPFFGALKDRLDFFWFQGETKFWTSRMQKHGSTVFRVNVAPGP  78

Query  124
FIASNPRVIVLLDAKSFPVLFDMSKVEKKDLFTGTYMPSTELTGGYRILSYLDPSEPNHT  183
                  R ++LLD +SFP+LFD+SKVEKKD+    YMPS  L  G R   +LDPSE
HT
Sbjct  79
```

```
PGFRTSRAVMLLDQRSFPILFDVSKVEKKDVLLANYMPSLALFAGNRPCVFLDPSEEKHT  138

Query  184
KLKQLLFNLIKNRRDYVIPEFSSSFTDLCEVVEYDLATKGKAAFNDPAEQAAFNFLSRAF  243
            K+K     LIK      IPE   + T++    + ++  KGKAA N    Q A N L
R F
Sbjct  139  KIKSFFMELIKLNASKWIPEMEKAATEVFSKWDTEI-
KKGKAAVNTQTSQVACNVLIRTF  197

Query  244  FG---
VKPIDTPLGKDAPSLISKWVLFNLAPILSVGLPKEVEEATLHSVRLPPLLVQNDY  300
             G     P +  L    P+L   W+   LAPI SVGLP  +EE T+HS R+P  +V
Y
Sbjct  198
MGRDPAAPGEGSLDTKGPTLFQTWLAPLLAPIASVGLPHLLEELTIHSFRIPAFVVSPTY  257

Query  301  HRLYEFFTSAAGSVLDEAEQS-
GISRDEACHNILFAVCFNSWGGFKILFPSLMKWIGRAG  359
             +L +FF +    ++D AE+  GI R+EA HNI+F + FN++GG  + FP+++
+G AG
Sbjct  258
KKLVKFFGTYGTELIDMAERDYGIERNEALHNIIFFMGFNAFGGLNLFFPTMVGHVGGAG  317

Query  360
LELHTKLAQEIRSAIQSTGGGKVTMAAMEQMPLMKSVVYETLRIEPPVALQYGKAKKDFI  419
            ++LH  L QE+R A++  GGG +T AA+ +M L++S+VYE LR+ PPV  QY KAK
+D I
Sbjct  318
MKLHNTLVQEVRDAVREEGGGALTPAALRKMELVRSIVYECLRMNPPVPYQYAKAKEDII  377

Query  420  LESHEAAYQVKEGEMLFGYQPFATKDPKIFDRPEEFVADRFVG-
EGVKLMEYVMWSNGPE  478
            +ESH+A Y+VK+GE++ G QP A +DP++F   E F+A+RF+G EG KL++YV+W
NGP
Sbjct  378
IESHDARYEVKKGELIGGCQPIAARDPRVFKDAETFIAERFMGEEGKKLIKYVIWGNGPA  437

Query  479  TETPSVANKQCAGKDFVVMAARLFVVELFKRYDSF  513
                SVANKQCA  D V + ++ F+  LF  YDSF
Sbjct  438  DGQTSVANKQCAANDLVPLLSQAFLACLFLHYDSF  472



Lambda      K        H        a         alpha
   0.319    0.136    0.399    0.792     4.96

Gapped
Lambda      K        H        a         alpha    sigma
   0.267   0.0410    0.140     1.90     42.6     43.6

Effective search space used: 234969
```

```
Matrix: BLOSUM62
Gap Penalties: Existence: 11, Extension: 1
Neighboring words threshold: 11
Window for multiple hits: 40




Query= sp|P54365|JANB_DROPS_Sex-regulated_protein_janus-
B_OS=Drosophila_pseudoobscura_pseudoobscura_GN=janB_PE=3_SV=2

Length=140

Subject= 43959-384_1_ORF2
>sp|P54365|JANB_DROPS_Sex-regulated_protein_janus-
B_OS=Drosophila_pseudoobscura_pseudoobscura_GN=janB_PE=3_SV=2|||7e-
07

Length=362


 Score = 33.9 bits (76),  Expect = 7e-07, Method: Compositional
matrix adjust.
 Identities = 25/86 (29%), Positives = 31/86 (36%), Gaps = 14/86
(16%)

Query  11
GQCAKPILRTYTSPAADLLKLPRVDIKEGKLRYLLLSVYIHGETKHARTVVRGWNTDSHD  70
            GQCA P       P    LK P  DI             I G   +  TV+ G  T
+
Sbjct  245  GQCAWPFANADFGPQTPPLKAPNADIG------------
IDGMIINLATVIAGAATNPFS  292

Query  71   DIYYKNVRA--MEKLGLCTKCLGGGK  94
              YY+   A  +E +  CT   G G
Sbjct  293  TGYYQGDAADPLEAVSACTGIFGKGA  318


 Score = 14.2 bits (25),  Expect = 1.9, Method: Compositional matrix
adjust.
 Identities = 5/18 (28%), Positives = 7/18 (39%), Gaps = 0/18 (0%)

Query  51   HGETKHARTVVRGWNTDS  68
            HG  +    +   WN  S
Sbjct  339  HGVNERRYLIPAMWNPSS  356



Lambda      K        H        a         alpha
   0.320    0.136    0.408    0.792     4.96

Gapped
```

```
Lambda      K        H        a         alpha    sigma
   0.267   0.0410    0.140     1.90     42.6     43.6

Effective search space used: 40120




Matrix: BLOSUM62
Gap Penalties: Existence: 11, Extension: 1
Neighboring words threshold: 11
Window for multiple hits: 40




Query= sp|Q01899|HSP7M_PHAVU_Heat_shock_70_kDa_protein,
_mitochondrial_OS=Phaseolus_vulgaris_PE=2_SV=1

Length=675

Subject= 38402-410_4_ORF1
>sp|Q01899|HSP7M_PHAVU_Heat_shock_70_kDa_protein,
_mitochondrial_OS=Phaseolus_vulgaris_PE=2_SV=1|||5e-102

Length=228


 Score =   301 bits (771),  Expect = 9e-102, Method: Compositional
matrix adjust.
 Identities = 142/180 (79%), Positives = 157/180 (87%), Gaps = 0/180
(0%)

Query  31
AYVAQKWSCLARPFSSRPAGNDVIGIDLGTTNSCVSVMEGKNPKVIENSEGARTTPSVVA  90
            A ++++W   AR FSS+  G+D+IGIDLGTTNSCV+VMEGK
+VIENSEGARTTPSVVA
Sbjct  49
ASLSKRWLDSARAFSSKAGGSDIIGIDLGTTNSCVAVMEGKTARVIENSEGARTTPSVVA  108

Query  91
FNQKGELLVGTPAKRQAVTNPTNTVFGTKRLIGRRFDDPQTQKEMKMVPFKIVKAPNGDA  150
            F  KGE LVGTPAKRQAVTNP NTVF  KRLIGR +DD Q QKE +MVP++I+K
NGDA
Sbjct  109
FTSKGERLVGTPAKRQAVTNPVNTVFAAKRLIGRAYDDAQVQKESQMVPYRIIKGANGDA  168

Query  151
WVEANGQQYSPSQIGAFVLTKMKETAEAYLGKSVSKAVITVPAYFNDAQRQATKDAGRIA  210
            WVEA GQQYSPSQIGAFVLTKMKETAEAYLG+
VSKAVITVPAYFNDAQRQATKDAGRIA
Sbjct  169
WVEAGGQQYSPSQIGAFVLTKMKETAEAYLGRPVSKAVITVPAYFNDAQRQATKDAGRIA  228
```

```
Lambda      K        H        a         alpha
   0.312    0.130    0.355    0.792     4.96

Gapped
Lambda      K        H        a         alpha    sigma
   0.267   0.0410    0.140     1.90     42.6     43.6

Effective search space used: 127710




Matrix: BLOSUM62
Gap Penalties: Existence: 11, Extension: 1
Neighboring words threshold: 11
Window for multiple hits: 40





Query= sp|Q03684|BIP4_TOBAC_Luminal-
binding_protein_4_OS=Nicotiana_tabacum_GN=BIP4_PE=2_SV=1

Length=667

Subject= 315574-60_2_ORF1
>sp|Q03684|BIP4_TOBAC_Luminal-
binding_protein_4_OS=Nicotiana_tabacum_GN=BIP4_PE=2_SV=1|||0

Length=430


 Score =   525 bits (1352),  Expect = 0.0, Method: Compositional
matrix adjust.
 Identities = 255/416 (61%), Positives = 328/416 (79%), Gaps = 3/416
(1%)

Query  252
VLSTNGDTHLGGEDFDQRIMEYFIKLIKKKHGKDISKDNRALGKLRREAERAKRALSSQH  311
            V +T GDTHLGGEDFD R++ +F++  K+K+ KDI+ + RAL +LR   ERAKR
LSS
Sbjct  1
VKATAGDTHLGGEDFDNRMVNHFVQEFKRKYKKDITGNARALRRLRTACERAKRTLSSTA  60

Query  312
QVRVEIESLFDGVDFSEPLTRARFEELNNDLFRKTMGPVKKAMDDAGLEKTQIDEIVLVG  371
            Q  +EI+SL++G+DF   +TRARFEELN DLFRK M PV+K + DA ++K+ I +
+VLVG
Sbjct  61
QTTIEIDSLYEGIDFYSTITRARFEELNMDLFRKCMEPVEKCLRDAKMDKSSIHDVVLVG  120
```

Query 372
GSTRIPKVQQLLKDYFDGKEPNKGVNPDEAVAYGAAVQGGILSGEGGDETKDILLLDVAP 431
GSTRIPKVQQLL+D+F+GKE K +NPDEAVAYGAAVQ ILSGEG ++ +D
+LLLDV P
Sbjct 121
GSTRIPKVQQLLQDFFNGKELCKSINPDEAVAYGAAVQAAILSGEGNEKVQDLLLLDVTP 180

Query 432
LTLGIETVGGVMTKLIPRNTVIPTKKSQVFTTYQDQQTTVTIQVFEGERSLTKDCRLLGK 491
L+LG+ET GGVMT LIPRNT IPTKK QVF+TY D Q V IQVFEGER+ T+D
LLGK
Sbjct 181
LSLGLETAGGVMTVLIPRNTTIPTKKEQVFSTYSDNQPGVLIQVFEGERTRTRDNNLLGK 240

Query 492
FDLTGIAPAPRGTPQIEVTFEVDANGILNVKAEDKASGKSEKITITNDKGRLSQEEIERM 551
F+L+GI PAPRG PQI V F++DANGILNV AEDK +G+ KITITNDKGRLS
+EEIE+M
Sbjct 241
FELSGIPPAPRGVPQITVCFDIDANGILNVSAEDKTTGQKNKITITNDKGRLSKEEIEKM 300

Query 552
VKEAEEFAEEDKKVKERIDARNSLETYVYNMRNQINDKDKLADKLESDEKEKIETATKEA 611
V++AE++ ED++VK++++A+NSLE Y YNMRN I D +K+A KLE+ +K+KIE A
+ A
Sbjct 301 VQDAEKYKAEDEEVKKKVEAKNSLENYAYNMRNTIRD-
EKIAGKLEAADKKKIEDAVEAA 359

Query 612 LEWLDDNQSAEKEDYEEKLKEVEAVCNPIITAVYQKSGG--APGGESGASEDDDHD
665
++WLD NQ AE E++E+K+KE+E +CNPII +YQ G PG G + +D D
Sbjct 360 IQWLDHNQLAEAEEFEDKMKELEGLCNPIIARMYQGGAGDVPPGAYGGDAYEDASD
415

Score = 16.2 bits (30), Expect = 3.2, Method: Compositional matrix
adjust.
Identities = 11/42 (26%), Positives = 18/42 (43%), Gaps = 6/42
(14%)

Query 215 IAYGLDKKGGEKNILVFDLGGGTFDVSILTIDNGVFEVLSTN 256
++ GL+ GG +L+ + +I T VF S N
Sbjct 181 LSLGLETAGGVMTVLI------PRNTTIPTKKEQVFSTYSDN 216

Score = 16.2 bits (30), Expect = 3.9, Method: Compositional matrix
adjust.
Identities = 9/23 (39%), Positives = 12/23 (52%), Gaps = 0/23 (0%)

Query 166 EAYLGKKIKDAVVTVPAYFNDAQ 188
EA KKI+DAV + + Q
Sbjct 345 EAADKKKIEDAVEAAIQWLDHNQ 367

```
Lambda      K        H        a         alpha
   0.314    0.134    0.372    0.792     4.96

Gapped
Lambda      K        H        a         alpha    sigma
   0.267   0.0410    0.140     1.90     42.6     43.6

Effective search space used: 249640




Matrix: BLOSUM62
Gap Penalties: Existence: 11, Extension: 1
Neighboring words threshold: 11
Window for multiple hits: 40





Query= sp|Q0IIM3|
HS105_BOVIN_Heat_shock_protein_105_kDa_OS=Bos_taurus_GN=HS
PH1_PE=2_SV=1

Length=859

Subject= 192949-144_3_ORF2
>sp|Q0IIM3|HS105_BOVIN_Heat_shock_protein_105_kDa_OS=Bos_taurus_GN=H
SPH1_PE=2_SV=1|||0

Length=861


 Score =   561 bits (1445),  Expect = 0.0, Method: Compositional
matrix adjust.
 Identities = 314/823 (38%), Positives = 479/823 (58%), Gaps =
51/823 (6%)

Query  1
MSVVGLDVGSQSCYIAVARAGGIETIANEFSDRCTPSVISFGSKNRTIGVAAKSQQITHA  60
            MSVVGLDVG+++C +AVAR  GI+ + N+ S R TP+++SF  K R IG AA +
Sbjct  16
MSVVGLDVGNENCIVAVARQRGIDVVLNDESKRETPAIVSFNDKQRFIGTAASAALTMSP  75

Query  61
NNTVSNFKRFHGRAFNDPFIQKEKENLSYDLVPMKNGGVGIKVMYMDEEHLFSVEQITAM  120
             NT +  KR  GR F+DP +Q++   L + +    +GG  + V Y+ E   F+  Q
+  M
Sbjct  76
KNTFAQIKRLLGRRFSDPEVQQDLHLLPFKVTEGPDGGPLLHVQYLGEIRSFTPSQVLGM  135
```

```
Query  121  
LLTKLKETAENNLKKPVTDCVISVPSFFTDAERRSVLDAAQIVGLNCLRLMNDMTAVALN  180
            + + LK  AE NL+  V+DCVI +P FF++ +RR++LDAA I GL  LRLM++ TA
AL
Sbjct  136  
IFSNLKSIAEKNLQTHVSDCVIGIPVFFSEIQRRAMLDAAAIAGLRPLRLMHETTATALA  195

Query  181  
YGIYKQDLPSLDEKPRIVVFVDMGHSAFQVSACAFNKGKLKVLGTAFDPFLGGKNFDAKL  240
            YGIYK DLP  D  P  VVFVD+GH+A Q+   AF KG+LKVL   FD  LGG+
+FD  +
Sbjct  196  YGIYKTDLP--
DNDPINVVFVDVGHAAMQICVAAFKKGQLKVLAHVFDRSLGGRDFDEVI  253

Query  241  
VEYFCAEFKTKYKLDAKSKIRALLRLYQECEKLKKLMSSNSTDLPLNIECFMNDKDVSGK  300
             ++FCA+FK +Y++D  S  RA  RL   CEK+KK++S+N+ + PLNIEC M+
+KDV G
Sbjct  254  FQHFCAKFKEEYRMDVLSNSRACQRLLSACEKMKKVLSANA-
EAPLNIECLMDEKDVRGF  312

Query  301  
MNRAQFEELCADLLQKIEVPLYLLMEQTQLKVEDVSAVEIVGGTTRIPAVKEKIAKFFGK  360
            M R  FE+L   + ++++      + + ++  + + AVE+VG  +R+PA+   ++
F K
Sbjct  313  
MKRDDFEKLAQPIFERVKAICERGLAEAKIPTDKLYAVEVVGSGSRVPAILRILSGVFRK  372

Query  361  DVSTTLNADEAVARGCALQCAILSPAFKVREFSVTDAVPFPISLVWSHDSEDAE--
GVHE  418
            + S T+NA E +ARGCALQCA+LSP F+VR+F V DA PF I L W   + ++E
G+H
Sbjct  373  
EPSRTMNASECIARGCALQCAMLSPTFRVRDFEVQDAFPFNICLAWKGAAPESEEGGLHG  432

Query  419  -------VFSRNHAAPFSKVLTFLRSGPFELEAFYSD----
PQGVPYPEAKIGRFIVQNV  467
                   VF + +A P +K+LTF RSG F ++A Y D    P G P    KI  F
+
Sbjct  433  EVSCNSIVFPKGNAVPSTKMLTFYRSGTFHIDALYGDLHDLPPGTPQ---
KINTFTIGPF  489

Query  468  SAQKDGEKSRVKVKVRVNTHGIFTISTASMV----EKIPAEENEV--
SSLEADMDCQNQR  521
              K  EK+++KVK+R+N HGI +I +A+M+     +IP  ++E   S++E + +
Q  +
Sbjct  490  KPSKT-EKAKIKVKIRLNLHGIVSIESATMIEEEEVEIPVSKSEGGPSAMEVETN-
QGSK  547

Query  522  PPENPDA-
EKNIQQDNNEAGTQPQVQTDGHQTSQSPPSPELTSEENKIPDADKANEKKVD  580
             E PD  + N+   +N       V+ D  + +    S E+ S +  +P   K+
K +
Sbjct  548  KMEVPDLNDPNVDNADNNPADPSGVEND--
```

```
EPAAEEKSAEMESPKKDVPKKTKSKRKNIG  605

Query  581
QPPEAKKPKIKVVNVELPIEANLVWQLGKDLLNMYIETEGKMIMQDKLEKERNDAKNAVE  640
                              ++   V  L +  L   +E E +M +QD++ +E  D
KNAVE
Sbjct  606  -----------------
VKECAVGALSQGDLQKAVELEYEMALQDRVMEETKDKKNAVE  647

Query  641
EYVYEFRDKLCGPYEKFICEQDHQKFLRLLTETENWLYEEGEDQAKQAYVDKLEELMKIG  700
             YVY+ R+K+   Y  +  E + +     L ETE+WLYE+GED+ K  YV KL EL
K+G
Sbjct  648
AYVYDMRNKMYEKYRDYATEAEREDLASKLQETEDWLYEDGEDETKGVYVAKLAELKKLG  707

Query  701
TPIKVRFQEAEERPKIFEELGQRLQHYAKIAADFRNNDEKYNHIDESEMKKVEKSVNEMM  760
             PI+ RF+E E R    E+L   +  + + A    + D +Y+HID +E +KV
N+
Sbjct  708  DPIEERFKEEEARGPCVEQLLHCINSFREAAL---
SKDPRYDHIDVAEKEKVIAECNKAE  764

Query  761  EWMNNVMSAQAKKSLDQDPVVCAQEIRAKIKELNNNCEPVVTQ  803
            EW+ +  S Q       +P + + +++ K + ++  C+P++T+
Sbjct  765  EWLRDKKSQQDHIPRSGNPALLSADMKKKTESVDRFCKPIMTK  807



Lambda      K        H        a         alpha
   0.314    0.131    0.373    0.792     4.96

Gapped
Lambda      K        H        a         alpha    sigma
   0.267   0.0410    0.140     1.90     42.6     43.6

Effective search space used: 669123




Query= sp|Q0IIM3|
HS105_BOVIN_Heat_shock_protein_105_kDa_OS=Bos_taurus_GN=HS
PH1_PE=2_SV=1

Length=859

Subject= 39064-407_4_ORF2
>sp|Q0IIM3|HS105_BOVIN_Heat_shock_protein_105_kDa_OS=Bos_taurus_GN=H
SPH1_PE=2_SV=1|||0

Length=844
```

Score = 550 bits (1416), Expect = 0.0, Method: Compositional matrix adjust.
Identities = 313/811 (39%), Positives = 464/811 (57%), Gaps = 40/811 (5%)

```
Query  1
MSVVGLDVGSQSCYIAVARAGGIETIANEFSDRCTPSVISFGSKNRTIGVAAKSQQITHA  60
            MSVVGLDVG+ +C +AVAR  GI+ + N+ S R TP++ +F  K R IG AA +
+
Sbjct  4
MSVVGLDVGNDNCIVAVARQRGIDVVLNDESKRETPALAAFSEKQRFIGTAASASLTMNP  63

Query  61
NNTVSNFKRFHGRAFNDPFIQKEKENLSYDLVPMKNGGVGIKVMYMDEEHLFSVEQITAM  120
             NT++  KRF GR F DP +Q++   L + +    +G   I V Y+ +   F+  Q
+  M
Sbjct  64
KNTIAQIKRFIGRHFRDPEVQQDLGFLPFLVTQSPDGSPLIHVQYLGKSRSFTPTQVLGM  123

Query  121
LLTKLKETAENNLKKPVTDCVISVPSFFTDAERRSVLDAAQIVGLNCLRLMNDMTAVALN  180
            +L+ LK  AE NL+  V DCVI +P FF + +RR+VL+AA I GL  LRLM++ TA
AL
Sbjct  124
ILSNLKSIAEKNLQTHVADCVIGIPIFFNEIQRRAVLNAAAIAGLRPLRLMHETTATALA  183

Query  181
YGIYKQDLPSLDEKPRIVVFVDMGHSAFQVSACAFNKGKLKVLGTAFDPFLGGKNFDAKL  240
            YGIY+ DLP  D  P  VVFVD+GH++ QV   AF KG+L+VL  AFD  LGG+
+FD  L
Sbjct  184  YGIYRTDLPEND--
PINVVFVDVGHASMQVCVVAFKKGQLRVLAHAFDRSLGGRDFDEVL  241

Query  241
VEYFCAEFKTKYKLDAKSKIRALLRLYQECEKLKKLMSSNSTDLPLNIECFMNDKDVSGK  300
             E+FC +FK +YK+D +S  RA  RL   CEK+KK++S+N  + PLNIEC M+
+KDV G
Sbjct  242  FEHFCGKFKEEYKMDVQSNPRACQRLRAACEKVKKMLSANP-
EAPLNIECLMDEKDVRGH  300

Query  301
MNRAQFEELCADLLQKIEVPLYLLMEQTQLKVEDVSAVEIVGGTTRIPAVKEKIAKFFGK  360
            M R   E+L   +  ++ +     +++  + V+++ AVE+VG  +R+PA+ + +
F K
Sbjct  301
MKRDDLEKLSQPIFDRVTILCQKALQEANISVDNIYAVEVVGSGSRVPAILKILTTVFKK  360

Query  361  DVSTTLNADEAVARGCALQCAILSPAFKVREFSVTDAVPFPISLVWSHDS-
EDAEGVH--  417
            + S T+NA E +ARGC LQCA+LSP F+VREF V DA PF ISLVW   + E  EG
H
Sbjct  361
EPSRTMNASECIARGCTLQCAMLSPTFRVREFEVQDAFPFSISLVWKGSAPETDEGAHVE  420
```

```
Query  418  -----EVFSRNHAAPFSKVLTFLRSGPFELEAFYSDPQGVP--
YPEAKIGRFIVQNVSAQ  470
                  +F + ++ P +K+LTF RS  F ++  Y+  Q +P   P+ KI  F +
Sbjct  421  AQTSTVIFPKGNSVPSTKMLTFYRSSTFNIDVLYTGMQDLPPGTPQ-
KINTFTIGPFQPS  479

Query  471
KDGEKSRVKVKVRVNTHGIFTISTASMVEKIPAEENEVSSLEADMDCQNQRPPENPDAEK  530
            K  EK+++KVK+R+N HGI +I +A+M+E+   +   V S + D      + P +
E
Sbjct  480  K-
AEKAKIKVKIRLNLHGIVSIESATMIEEEEVDVPVVKSKDTDSVMDTDKEPVDGFGEA  538

Query  531  N---
IQQDNNEAGTQPQVQTDGHQTSQSPPSPELTSEENKIPDADKANEKKVDQPPEAKK  587
                +    N AGT    +  G             + E K  D D      ++ P
+
Sbjct  539  GDAKVDGPENSAGTAAGSENGG-------------ASEQKTTDMD------
LEPPKKDAA  579

Query  588
PKIKVVNVELPIEANLVWQLGKDLLNMYIETEGKMIMQDKLEKERNDAKNAVEEYVYEFR  647
             K K    ++ +  + V  + +  L   IE E  M +QD++ +E  D KNAVE
YVY+ R
Sbjct  580
KKKKSKRKDIDVNESAVGGVLQADLQKAIEEECDMALQDRIMEETKDKKNAVESYVYDMR  639

Query  648
DKLCGPYEKFICEQDHQKFLRLLTETENWLYEEGEDQAKQAYVDKLEELMKIGTPIKVRF  707
            +KL   Y ++  + + +    +L ETE+WLYE+GED +K  YV KL EL K+G PI
+ RF
Sbjct  640
NKLYEKYNQYATDLEREDLAAMLQETEDWLYEDGEDVSKSVYVSKLTELKKLGDPIEERF  699

Query  708
QEAEERPKIFEELGQRLQHYAKIAADFRNNDEKYNHIDESEMKKVEKSVNEMMEWMNNVM  767
            +E E R    E+L   +  + + A   R+ D K++HID +E +KV    N+  EW+
Sbjct  700  KEEESRGPCLEQLMYCINSFREAA---
RSKDSKFDHIDTTEKEKVIAECNKAEEWLREKK  756

Query  768  SAQAKKSLDQDPVVCAQEIRAKIKELNNNCE  798
              Q       +PV+   E++ K + L+  C+
Sbjct  757  QQQDHLPKPANPVLLCAEVKKKTETLDRICK  787



Lambda      K        H        a         alpha
   0.314    0.131    0.373    0.792     4.96

Gapped
Lambda      K        H        a         alpha    sigma
   0.267   0.0410    0.140     1.90     42.6     43.6

Effective search space used: 669123
```

```
Matrix: BLOSUM62
Gap Penalties: Existence: 11, Extension: 1
Neighboring words threshold: 11
Window for multiple hits: 40




Query= sp|Q0JCU7|ZEP_ORYSJ_Zeaxanthin_epoxidase,
_chloroplastic_OS=Oryza_sativa_subsp._japonica_GN=ZEP_PE=2_SV=1

Length=659

Subject= 311894-62_3_ORF2
>sp|Q0JCU7|ZEP_ORYSJ_Zeaxanthin_epoxidase,
_chloroplastic_OS=Oryza_sativa_subsp._japonica_GN=ZEP_PE=2_SV=1|||3e
-32

Length=443


 Score =   116 bits (290),  Expect = 5e-32, Method: Compositional
matrix adjust.
 Identities = 114/376 (30%), Positives = 170/376 (45%), Gaps =
45/376 (12%)

Query  80
VLVAGGGIGGLVLALAARRKGYEVTVFERDMSAVRGEGQYRGPIQIQSNALAALEAIDMS  139
            V++ GGGI GL  ALA  R G +  V E+    +R  G     I + +NA  ALEA
+ ++
Sbjct  43   VVIVGGGIAGLATALALHRLGVKPVVLEQ-AEHLRTAGS---
AIGMWTNAWKALEALGVA  98

Query  140  --
VAEEVMREGCVTGDRINGLVDGISGSWYIKFDTFTPAAERGLPVTRVISRMTLQQILA  197
              + E+ +R   + G  I G  DG   + ++  D       RG      + R  L
+ L
Sbjct  99   DGLREKFVR---LAGAEILG-EDGTKITGFVFSDGSRDVELRG------
VERKALLETLQ  148

Query  198  RAVGDDAILNDSHVVDFID-
DGNKVTAILEDGRKFEGDLLVGADGIWSKVRKVLFGQSEA  256
              + D  +  DS VV     DG       ++G+  +  +L+G DG+ S V K +
+
Sbjct  149  EPIPDGTVFYDSQVVGIKKLDGGYTEVQCKNGQTIQTKVLIGCDGVGSVVGKYM-
NMGKL  207

Query  257  TYSEYTCYTGIADFVPPDIDTVG--
YRVFLGHKQYFVSSDVGAGKMQWYAFHKEPAGGTD  314
```

```
             Y+ YT   G+A  V PD   +   ++  +G         + A ++ W+     P
Sbjct  208  NYAGYTATRGLA--
VYPDGHNLSPTFKQIVGRGVRAAIVPMDANRVYWFVAFNSPDERLS  265

Query  315  PENGKNKRLLEIFNGWCDNVVDLINATDEEAILRRDIYDR-------
PPTFNWGKGRVTL  367
                  K +L    GW   V D IN T  E++ R+ I DR       PP +   KG
VTL
Sbjct  266  DLELVRKEVLNFVRGWPSIVTDTINNTPLESLSRKGISDRWMWPVGGPPLY---
KGGVTL  322

Query  368  LGDSVHAMQPNLGQGGCMAIEDGYQLAVELEKSWQE---SAKSGTPMD-------
IVSSL  417
             GD++H M PNLGQGGC A+ED   LA +L K+      S   G   D       I
+L
Sbjct  323
AGDAMHPMTPNLGQGGCCALEDAVVLARKLSKALSTRDYSPSIGASNDGSQETEKIELAL  382

Query  418  RRYEKE---RILRVSV  430
            + Y +E   R+LRV++
Sbjct  383  KSYTEERWPRMLRVAI  398


 Score = 16.2 bits (30),  Expect = 4.0, Method: Compositional matrix
adjust.
 Identities = 6/17 (35%), Positives = 11/17 (65%), Gaps = 0/17 (0%)

Query  75   TRRPRVLVAGGGIGGLV  91
            T + +VL+   G+G +V
Sbjct  182  TIQTKVLIGCDGVGSVV  198


 Score = 15.0 bits (27),  Expect = 7.3, Method: Compositional matrix
adjust.
 Identities = 12/37 (32%), Positives = 16/37 (43%), Gaps = 4/37
(11%)

Query  487  VLGGNSTKLEGRPLSCRLSDKANDQLRRWFEDDDALE  523
            +LG + TK+ G       SD + D   R  E    LE
Sbjct  113  ILGEDGTKITG----FVFSDGSRDVELRGVERKALLE  145



Lambda      K        H        a         alpha
   0.318    0.136    0.409    0.792     4.96

Gapped
Lambda      K        H        a         alpha    sigma
   0.267   0.0410    0.140     1.90     42.6     43.6

Effective search space used: 254592
```

Matrix: BLOSUM62
Gap Penalties: Existence: 11, Extension: 1
Neighboring words threshold: 11
Window for multiple hits: 40

Query= sp|Q25AG5|ERG3_ORYSI_Elicitor-
responsive_protein_3_OS=Oryza_sativa_subsp._indica_GN=ERG3_PE=2_SV=1

Length=144

Subject= 104606-243_6_ORF2
>sp|Q25AG5|ERG3_ORYSI_Elicitor-
responsive_protein_3_OS=Oryza_sativa_subsp._indica_GN=ERG3_PE=2_SV=1
|||2e-35

Length=256

```
 Score =   113 bits (283),  Expect = 2e-35, Method: Compositional
matrix adjust.
 Identities = 57/124 (46%), Positives = 78/124 (63%), Gaps = 1/124
(1%)

Query  3
QGTLEVLLVGAKGLENTDYLCNMDPYAVLKCRSQEQKSSVASGKGSDPEWNETFMFSVTH  62
            +GTLEV LV    L + D     DPYAV+KC +Q+ KS V    GS+P WNE+F+F
+
Sbjct  132
KGTLEVKLVEGHDLLSADTASKSDPYAVIKCDTQQHKSRVMQNAGSNPVWNESFVFEING  191

Query  63   NATELIIKLMDSDSGTDDDFVGEATISLEAIYTEGSIPPTVYNVV-
KEEEYRGEIKVGLT  121
            NA EL + L D D+ + DD +G ATI L+ ++  G + PT Y VV K  + +G+I
VGLT
Sbjct  192
NANELHVSLFDKDTFSKDDPLGNATIPLQRLFITGQVAPTPYKVVGKAGQPQGDIVVGLT  251

Query  122  FTPE  125
            F P+
Sbjct  252  FNPK  255


 Score = 84.0 bits (206),  Expect = 1e-24, Method: Compositional
matrix adjust.
 Identities = 45/130 (35%), Positives = 73/130 (56%), Gaps = 2/130
(2%)

Query  1
MVQGTLEVLLVGAKGLENTDYLCNMDPYAVLKCRSQEQKSSVASGKGSDPEWNETFMF-S  59
```

```
            M +G LEV L+ A  L++ +     DPYAVL C + + +S V    G +P WNETF
+  +
Sbjct  2
MPEGHLEVELLRAYSLKDVEAFGKSDPYAVLTCGAAKFESKVLQEAGGNPVWNETFLMDT  61

Query  60   VTHNATELIIKLMDSDSGTDDDFVGEATISLEAIYTEGSIPPTVYNV-
VKEEEYRGEIKV  118
             + NA EL+I L D +    D+ +G   + L   Y++  + PT Y V +K  ++
GE+++
Sbjct  62
KSDNAPELLIALFDKEKKGKDEAMGTVRVPLSTAYSQKQVAPTRYKVQLKNGKFHGEVEL  121

Query  119  GLTFTPEDDR  128
             + F P+  R
Sbjct  122  RIKFFPKLSR  131



Lambda      K        H        a         alpha
   0.310    0.131    0.375    0.792     4.96

Gapped
Lambda      K        H        a         alpha    sigma
   0.267   0.0410    0.140     1.90     42.6     43.6

Effective search space used: 29264




Query= sp|Q25AG5|ERG3_ORYSI_Elicitor-
responsive_protein_3_OS=Oryza_sativa_subsp._indica_GN=ERG3_PE=2_SV=1

Length=144

Subject= 60571-325_3_ORF2
>sp|Q25AG5|ERG3_ORYSI_Elicitor-
responsive_protein_3_OS=Oryza_sativa_subsp._indica_GN=ERG3_PE=2_SV=1
|||1e-31

Length=264


 Score =   103 bits (257),  Expect = 1e-31, Method: Compositional
matrix adjust.
 Identities = 50/125 (40%), Positives = 76/125 (61%), Gaps = 2/125
(2%)

Query  3
QGTLEVLLVGAKGLENTDYLCNMDPYAVLKCRSQEQKSSVASGKGSDPEWNETFMFSVTH  62
             G LEV L+ A GL + D L   DP+A++ C  + QKS V +G   DP WN+ F+F
+V +
Sbjct  141  HGALEVHLLEAHGLLDKDLLGKSDPFAIVYCHKESQKSIVINGT-
CDPVWNQKFVFNVNN  199
```

```
Query  63   NATELIIKLMDSDSGTDDDFVGEATISLEAIYTEGSIPPTVYNVVKEE-
EYRGEIKVGLT  121
              TE++IKL D D    DD +G   + L  ++++G +PP  Y V+ E+ + +GE+
+ L
Sbjct  200
EVTEILIKLFDKDDLVADDALGIVVVPLSKVFSDGQLPPMRYKVLGEKGQPQGEVSLALK  259

Query  122  FTPED  126
            FTP D
Sbjct  260  FTPRD  264


 Score = 78.2 bits (191),  Expect = 1e-22, Method: Compositional
matrix adjust.
 Identities = 38/126 (30%), Positives = 68/126 (54%), Gaps = 1/126
(1%)

Query  1
MVQGTLEVLLVGAKGLENTDYLCNMDPYAVLKCRSQEQKSSVASGKGSDPEWNETFMFSV  60
            M +GT+ V L+ A  L++ +     DPYA +   +Q+ +S      G +P WN++F
+  +
Sbjct  13
MPEGTVAVELIKAHALKDVEAFGKSDPYATITIGTQKHRSRTIHDGGGNPLWNQSFLLGI  72

Query  61   THNATELIIKLMDSDSGTDDDFVGEATISLEAIYTEGSIPPTVYNVVKEE-
EYRGEIKVG  119
                 EL I + D +    D+ +G   I L  ++ E  IP + Y V + + +++GE
++VG
Sbjct  73
PEGPHELDIAIYDEERHGVDEIMGTVNIHLPKLFAEKHIPVSKYKVQRPDGKFQGELEVG  132

Query  120  LTFTPE  125
            L F P+
Sbjct  133  LKFFPK  138



Lambda      K        H        a         alpha
   0.310    0.131    0.375    0.792     4.96

Gapped
Lambda      K        H        a         alpha    sigma
   0.267   0.0410    0.140     1.90     42.6     43.6

Effective search space used: 29264




Matrix: BLOSUM62
Gap Penalties: Existence: 11, Extension: 1
Neighboring words threshold: 11
Window for multiple hits: 40
```

Query= sp|Q39196|PIP14_ARATH_Probable_aquaporin_PIP1-
4_OS=Arabidopsis_thaliana_GN=PIP1.4_PE=1_SV=1

Length=287

Subject= 84884-273_3_ORF2
>sp|Q39196|PIP14_ARATH_Probable_aquaporin_PIP1-
4_OS=Arabidopsis_thaliana_GN=PIP1.4_PE=1_SV=1|||2e-173

Length=302

Score = 473 bits (1218), Expect = 2e-173, Method: Compositional
matrix adjust.
Identities = 231/291 (79%), Positives = 254/291 (87%), Gaps = 6/291
(2%)

```
Query  1
MEGKEEDVRVGANKFPERQPIGTSAQSTDKDYKEPPPAPLFEPGELSSWSFYRAGIAEFI  60
            ME K+EDVR+GANKF ERQP+GT+AQ+  +DY EPP   LFEP E SSWSF
+RAGIAEF
Sbjct  12   MESKDEDVRLGANKFNERQPLGTAAQT--
RDYTEPPATRLFEPAEFSSWSFWRAGIAEFF  69

Query  61   ATFLFLYITVLTVMGVKR----
APNMCASVGIQGIAWAFGGMIFALVYCTAGISGGHINP  116
            AT LFLYI++ TVMG K     A N C
VGIQGIAWAFGGMIFALVYCTAGISGGHINP
Sbjct  70
ATLLFLYISIQTVMGYKHGVPSAKNECPGVGIQGIAWAFGGMIFALVYCTAGISGGHINP  129

Query  117
AVTFGLFLARKLSLTRAVFYMIMQCLGAICGAGVVKGFQPTPYQTLGGGANTVAHGYTKG  176
            AVT+GLFLARK+SL R V+Y+IMQCLGAICGAG+VKGFQP  Y   GGGAN V
HGYTKG
Sbjct  130
AVTWGLFLARKVSLPRTVYYIIMQCLGAICGAGIVKGFQPDFYNNNGGGANVVNHGYTKG  189

Query  177
SGLGAEIIGTFVLVYTVFSATDAKRSARDSHVPILAPLPIGFAVFLVHLATIPITGTGIN  236
             GLGAEI+GTFVLVYTVFSATDAKRSARDSHVP
+LAPLPIGFAVFLVHLATIPITGTGIN
Sbjct  190
DGLGAEIVGTFVLVYTVFSATDAKRSARDSHVPLLAPLPIGFAVFLVHLATIPITGTGIN  249

Query  237  PARSLGAAIIYNKDHSWDDHWIFWVGPFIGAALAALYHQIVIRAIPFKSKS  287
            PARSLGAAIIYN+ H+W+DHWIFWVGPFIGA+LA LYH +VIRA+PFKS+
Sbjct  250  PARSLGAAIIYNQKHAWNDHWIFWVGPFIGASLACLYHVVVIRALPFKSRE  300
```

```
Lambda      K        H        a         alpha
   0.324    0.140    0.440    0.792     4.96

Gapped
Lambda      K        H        a         alpha    sigma
   0.267   0.0410    0.140     1.90     42.6     43.6

Effective search space used: 72036



Matrix: BLOSUM62
Gap Penalties: Existence: 11, Extension: 1
Neighboring words threshold: 11
Window for multiple hits: 40




Query= sp|Q42534|
PME2_ARATH_Pectinesterase_2_OS=Arabidopsis_thaliana_GN=PME
2_PE=2_SV=2

Length=587

Subject= 43733-385_4_ORF1
>sp|Q42534|PME2_ARATH_Pectinesterase_2_OS=Arabidopsis_thaliana_GN=PM
E2_PE=2_SV=2|||2e-126

Length=661


 Score =   381 bits (979),  Expect = 2e-127, Method: Compositional
matrix adjust.
 Identities = 231/598 (39%), Positives = 328/598 (55%), Gaps =
46/598 (8%)

Query  25   AAIALLLLASI---VGIAATTTNQNKNQKITTL-----------
SSTSHAILKSVCSSTL  70
            A +A LL A++   V + A+  NQ    +   L           S  SH ++ S+C
T
Sbjct  75
AILAPLLFAAVILCVVLYASAPNQAAQHEGHELFLPPSGLAAAASPASHVLVSSICKHTA  134

Query  71   YPELCFSAVAATGGKEL--------
TSQKEVIEASLNLTTKAVKHNYFAVKKLIAKRKGL  122
            YP +C        G+EL        T+  +V+  ++  T + V      +    +
+GL
Sbjct  135  YPVVC--------GRELSGFRIPLSTTPLDVVSLAVQATARRVNEAQ-
QLATNCSDERGL  185
```

```
Query  123  TPREVTALHDCLETIDETLDELHVAVEDLHQYPKQKS---
LRKHADDLKTLISSAITNQG  179
            +  E    +DC+E +    D++++A   L    ++ S   L+    D+K  +S+++
+ Q
Sbjct  186
SLLEDQCANDCVELLYSVKDQMNLAAARLSGLGRKSSVASLKSALADVKVWLSTSLSYQS  245

Query  180  TCLDGFSYDDADRKVRKALLKGQVHVEHMCSNALAMIKNMTET--DIANF-
ELRDKSSTF  236
             C D F    A   +++ +   Q ++  +   +L+++  +++   DIA +
S+ F
Sbjct  246  VCSDNFQV--
APGSIQQQIQNNQAYLTQVLGVSLSLVDILSQVGNDIAPWLGALPPSAPF  303

Query  237  TNNNNRKLKEVTGDLD---SDGWPKWLSVGDRRLLQG--
STIKADATVADDGSGDFTTVA  291
            T+   R L E   D D    D +P W+S  +R+LLQ   S I A+A VA DGSG
+TT+
Sbjct  304
THVRRRLLSEKPIDADFDIVDEFPHWVSGAERKLLQSTSSAISANAVVAKDGSGQYTTIT  363

Query  292
AAVAAAPEKSNKRFVIHIKAGVYRENVEVTKKKTNIMFLGDGRGKTIITGSRNVVDGS-T  350
            AAV A P+  + R+VI+IK G Y+E   VTK + N+ F+GDG GKTIITGSRNV
G
Sbjct  364
AAVNAIPKSYSGRYVIYIKNGTYKEVFNVTKDQQNVTFVGDGIGKTIITGSRNVASGDYN  423

Query  351
TFHSATVAAVGERFLARDITFQNTAGPSKHQAVALRVGSDFSAFYQCDMFAYQDTLYVHS  410
            T+ ++TV   G  F ARD+T +NTAGPS HQAVALR G+D+  FY+C
YQDTLY  S
Sbjct  424
TYRTSTVGVAGSGFYARDLTIRNTAGPSGHQAVALRAGADYMVFYRCSFEGYQDTLYALS  483

Query  411
NRQFFVKCHITGTVDFIFGNAAAVLQDCDINARRPNSGQKNMVTAQGRSDPNQNTGIVIQ  470
            +RQF+ +C I+GTVDFIFGNA AV Q+C + AR P  GQ+N  TAQGR      +G
Q
Sbjct  484
SRQFYRECQISGTVDFIFGNAIAVFQNCVLLARLPMQGQQNTYTAQGRQLEADISGYAFQ  543

Query  471
NCRIGGTSDLLAVKGTFPTYLGRPWKEYSRTVIMQSDISDVIRPEGWHEWSGSFAL-DTL  529
            NC +   S L     +  TYLGRPWK YSRTV +QS++  VI P GW  W+ S
DT+
Sbjct  544
NCTVKADSTLTKANFSVSTYLGRPWKAYSRTVFLQSELQAVIDPTGWLPWNSSNPFTDTV  603

Query  530
TYREYLNRGGGAGTANRVKWKGYKVITSDTEAQPFTAGQFIGGGGWLASTGFPFSLSL  587
             Y EY NRG G+GT+ RV WKG     S ++A  FT   FI G  WL     P+
SL
```

```
Sbjct  604
YYGEYGNRGAGSGTSKRVSWKGVHSQMSKSDASQFTITNFIAGQSWLDVLQVPYQASL  661



Lambda      K        H        a         alpha
   0.317    0.131    0.382    0.792     4.96

Gapped
Lambda      K        H        a         alpha    sigma
   0.267   0.0410    0.140     1.90     42.6     43.6

Effective search space used: 343200




Query= sp|Q42534|
PME2_ARATH_Pectinesterase_2_OS=Arabidopsis_thaliana_GN=PME
2_PE=2_SV=2

Length=587

Subject= 71754-298_1_ORF1
>sp|Q42534|PME2_ARATH_Pectinesterase_2_OS=Arabidopsis_thaliana_GN=PM
E2_PE=2_SV=2|||1e-126

Length=662


 Score =   381 bits (979),  Expect = 2e-127, Method: Compositional
matrix adjust.
 Identities = 231/598 (39%), Positives = 328/598 (55%), Gaps =
46/598 (8%)

Query  25   AAIALLLLASI---VGIAATTTNQNKNQKITTL-----------
SSTSHAILKSVCSSTL  70
            A +A LL A++   V + A+  NQ    +   L           S  SH ++ S+C
T
Sbjct  76
AILAPLLFAAVILCVVLYASAPNQAAQHEGHELFLPPSGLAAAASPASHVLVSSICKHTA  135

Query  71   YPELCFSAVAATGGKEL--------
TSQKEVIEASLNLTTKAVKHNYFAVKKLIAKRKGL  122
            YP +C       G+EL        T+  +V+  ++  T + V      +    +
+GL
Sbjct  136  YPVVC--------GRELSGFRIPLSTTPLDVVSLAVQATARRVNEAQ-
QLATNCSDERGL  186

Query  123  TPREVTALHDCLETIDETLDELHVAVEDLHQYPKQKS---
LRKHADDLKTLISSAITNQG  179
            +  E    +DC+E +    D++++A   L    ++ S   L+    D+K  +S+++
+ Q
Sbjct  187
```

```
SLLEDQCANDCVELLYSVKDQMNLAAARLSGLGRKSSVASLKSALADVKVWLSTSLSYQS  246

Query  180  TCLDGFSYDDADRKVRKALLKGQVHVEHMCSNALAMIKNMTET--DIANF-
ELRDKSSTF  236
             C D F    A   +++ +   Q ++  +   +L+++  +++   DIA +
S+ F
Sbjct  247  VCSDNFQV--
APGSIQQQIQNNQAYLTQVLGVSLSLVDILSQVGNDIAPWLGALPPSAPF  304

Query  237  TNNNNRKLKEVTGDLD---SDGWPKWLSVGDRRLLQG--
STIKADATVADDGSGDFTTVA  291
            T+   R L E   D D    D +P W+S  +R+LLQ   S I A+A VA DGSG
+TT+
Sbjct  305
THVRRRLLSEKPIDADFDIVDEFPHWVSGAERKLLQSTSSAISANAVVAKDGSGQYTTIT  364

Query  292
AAVAAAPEKSNKRFVIHIKAGVYRENVEVTKKKTNIMFLGDGRGKTIITGSRNVVDGS-T  350
            AAV A P+  + R+VI+IK G Y+E   VTK + N+ F+GDG GKTIITGSRNV
G
Sbjct  365
AAVNAIPKSYSGRYVIYIKNGTYKEVFNVTKDQQNVTFVGDGIGKTIITGSRNVASGDYN  424

Query  351
TFHSATVAAVGERFLARDITFQNTAGPSKHQAVALRVGSDFSAFYQCDMFAYQDTLYVHS  410
            T+ ++TV   G  F ARD+T +NTAGPS HQAVALR G+D+  FY+C
YQDTLY  S
Sbjct  425
TYRTSTVGVAGSGFYARDLTIRNTAGPSGHQAVALRAGADYMVFYRCSFEGYQDTLYALS  484

Query  411
NRQFFVKCHITGTVDFIFGNAAAVLQDCDINARRPNSGQKNMVTAQGRSDPNQNTGIVIQ  470
            +RQF+ +C I+GTVDFIFGNA AV Q+C + AR P  GQ+N  TAQGR      +G
Q
Sbjct  485
SRQFYRECQISGTVDFIFGNAIAVFQNCVLLARLPMQGQQNTYTAQGRQLEADISGYAFQ  544

Query  471
NCRIGGTSDLLAVKGTFPTYLGRPWKEYSRTVIMQSDISDVIRPEGWHEWSGSFAL-DTL  529
            NC +   S L     +  TYLGRPWK YSRTV +QS++  VI P GW  W+ S
DT+
Sbjct  545
NCTVKADSTLTKANFSVSTYLGRPWKAYSRTVFLQSELQAVIDPTGWLPWNSSNPFTDTV  604

Query  530
TYREYLNRGGGAGTANRVKWKGYKVITSDTEAQPFTAGQFIGGGGWLASTGFPFSLSL  587
             Y EY NRG G+GT+ RV WKG     S ++A  FT   FI G  WL     P+
SL
Sbjct  605
YYGEYGNRGAGSGTSKRVSWKGVHSQMSKSDASQFTITNFIAGQSWLDVLQVPYQASL  662



Lambda      K        H        a         alpha
```

```
   0.317    0.131    0.382    0.792     4.96

Gapped
Lambda      K        H        a         alpha    sigma
   0.267   0.0410    0.140     1.90     42.6     43.6

Effective search space used: 343200



Matrix: BLOSUM62
Gap Penalties: Existence: 11, Extension: 1
Neighboring words threshold: 11
Window for multiple hits: 40



Query= sp|Q42798|
C93A1_SOYBN_Cytochrome_P450_93A1_OS=Glycine_max_GN=CYP93A1
_PE=2_SV=1

Length=509

Subject= 292626-72_3_ORF2
>sp|Q42798|C93A1_SOYBN_Cytochrome_P450_93A1_OS=Glycine_max_GN=CYP93A
1_PE=2_SV=1|||1e-112

Length=518


 Score =   322 bits (826),  Expect = 1e-107, Method: Compositional
matrix adjust.
 Identities = 187/507 (37%), Positives = 282/507 (56%), Gaps =
23/507 (5%)

Query  6    LLICLVSTIVFAYILWRKQSKKN-
LPPSPKALPIIGHLHLVSPIPHQDFYKLSTRHGPIM  64
            L+I   S ++    +WR+ +    +PP P+ LPI+G+LH +  +PHQ F +LS
+GP+M
Sbjct  18
LIIAACSILLGVMWVWRRAAAAYIMPPGPRGLPILGYLHKLGALPHQTFARLSNIYGPLM  77

Query  65
QLFLGSVPCVVASTAEAAKEFLKTHEINFSNRPGQNVAVKGLAYDSQDFLFAFAPFGPYW  124
             + LG VP +VAS+ + A   L   +  F+ RP        + Y   + LF+
GPYW
Sbjct  78   CVRLGRVPLLVASSPDMASLIL---DKTFAGRPVH--IAPSIMYGGPNILFSQP--
GPYW  130

Query  125
KFMKKLCMSELLSGRMMDQFLPVRQQETKRFISRVFRKGVAGEAVDFGDELMTLSNNIVS  184
```

```
            K M+++  ++LL+ + +  F PVR  E +  +  V      G  +   D L T 
NN +S
Sbjct  131  KLMRQIFTTDLLTSKRLSHFRPVRAHEMRGLLLSVL--
AARGSPLCIRDLLHTTINNTIS  188

Query  185  RMTLSQKTSE--
NDNQAEEMKKLVSNIAELMGKFNVSDFIWYLKPFDLQGFNRKIKETRD  242
             M L +   +   D     +   V  I  L+G+FN  D+I YL   DLQG+ ++
KE
Sbjct  189
TMALGKPLHQVSADPNVGTIVSTVMEIVNLIGQFNWGDYIPYLAWMDLQGYGKRSKEIGG  248

Query  243
RFDVVVDGIIKQRQEERRKNKETGTAKQFKDMLDVLLDMHEDENAEIKLDKKNIKAFIMD  302
            R   V+  +I +R   RR   +  +A     +  +L    + ++ E+ +   +I+A
++
Sbjct  249  RVRSVLQAVIDKR---
RRCADDIDSAAARDLLDLLLAASEDAKHKELHIGDDSIRAVLLG  305

Query  303
IFVAGTDTSAVSIEWAMAELINNPDVLEKARQEIDAVVGKSRMVEESDIANLPYLQAIVR  362
            +F+AG+DT++++IEWA+AEL+ NP+ L + ++E+D VVG+ R+VEE D+ANL YL
+A+V
Sbjct  306
MFIAGSDTASITIEWALAELLANPEKLRRVQEELDEVVGRERVVEEGDLANLVYLRAVVN  365

Query  363  ETLRLHPGGPLVVRESSKSAV-
VCGYDIPAKTRLFVNVWAIGRDPNHWEKPFEFRPERFI  421
            E LRLHP  PL+       A  V GY IPA T  FVN+WAI RDP+ W  P +F
PERF+
Sbjct  366
EALRLHPPTPLLAPHRCLEACHVGGYRIPADTLAFVNIWAIHRDPSLWANPLDFCPERFL  425

Query  422  RDGQNQLDVR-
GQHYHFIPFGSGRRTCPGASLAWQVVPVNLAIIIQCFQWKLVGGNGKVD  480
                   DV  GQH+ F+PFGSGRRTCPG  L        LA ++  F W    G
Sbjct  426  ----
PSFDVTPGQHFGFLPFGSGRRTCPGWKLGLLNAQNVLAHLLHAFHWTTPTGKPP-P  480

Query  481  MEEKSGITLPRANPIICVPVPRINPFP  507
            + EK G+T+    P+  VP+PR+ P P
Sbjct  481  LNEKFGLTVAIDIPLSVVPMPRL-PMP  506



Lambda      K        H        a         alpha
   0.322    0.138    0.416    0.792     4.96

Gapped
Lambda      K        H        a         alpha    sigma
   0.267   0.0410    0.140     1.90     42.6     43.6

Effective search space used: 228942
```

```
Query= sp|Q42798|
C93A1_SOYBN_Cytochrome_P450_93A1_OS=Glycine_max_GN=CYP93A1
_PE=2_SV=1

Length=509

Subject= 293605-72_3_ORF2
>sp|Q42798|C93A1_SOYBN_Cytochrome_P450_93A1_OS=Glycine_max_GN=CYP93A
1_PE=2_SV=1|||4e-106

Length=482


 Score =   305 bits (780),  Expect = 3e-101, Method: Compositional
matrix adjust.
 Identities = 170/452 (38%), Positives = 259/452 (57%), Gaps =
21/452 (5%)

Query  6    LLICLVSTIVFAYILWRKQSKKN-
LPPSPKALPIIGHLHLVSPIPHQDFYKLSTRHGPIM  64
            L+I   S ++    +WR+ +    +PP P+ LPI+G+LH +  +PHQ F +LS
+GP+M
Sbjct  18
LIIAACSILLGVMWVWRRAAAAYIMPPGPRGLPILGYLHKLGALPHQTFARLSNIYGPLM  77

Query  65
QLFLGSVPCVVASTAEAAKEFLKTHEINFSNRPGQNVAVKGLAYDSQDFLFAFAPFGPYW  124
             + LG VP +VAS+ + A   L   +  F+ RP        + Y   + LF+
GPYW
Sbjct  78   CVRLGRVPLLVASSPDMASLIL---DKTFAGRPVH--IAPSIMYGGPNILFSQP--
GPYW  130

Query  125
KFMKKLCMSELLSGRMMDQFLPVRQQETKRFISRVFRKGVAGEAVDFGDELMTLSNNIVS  184
            K M+++  ++LL+ + +  F PVR  E +  +  V      G  +   D L T
NN +S
Sbjct  131  KLMRQIFTTDLLTSKRLSHFRPVRAHEMRGLLLSVL--
AARGSPLCIRDLLHTTINNTIS  188

Query  185  RMTLSQKTSE--
NDNQAEEMKKLVSNIAELMGKFNVSDFIWYLKPFDLQGFNRKIKETRD  242
             M L +   +   D     +   V  I  L+G+FN  D+I YL   DLQG+ ++
KE
Sbjct  189
TMALGKPLHQVSADPNVGTIVSTVMEIVNLIGQFNWGDYIPYLAWMDLQGYGKRSKEIGG  248

Query  243
RFDVVVDGIIKQRQEERRKNKETGTAKQFKDMLDVLLDMHEDENAEIKLDKKNIKAFIMD  302
            R   V+  +I +R   RR   +  +A     +  +L    + ++ E+ +   +I+A
++
Sbjct  249  RVRSVLQAVIDKR---
```

RRCADDIDSAAARDLLDLLLAASEDAKHKELHIGDDSIRAVLLG 305

Query 303
IFVAGTDTSAVSIEWAMAELINNPDVLEKARQEIDAVVGKSRMVEESDIANLPYLQAIVR 362
+F+AG+DT++++IEWA+AEL+ NP+ L + ++E+D VVG+ R+VEE D+ANL YL
+A+V
Sbjct 306
MFIAGSDTASITIEWALAELLANPEKLRRVQEELDEVVGRERVVEEGDLANLVYLRAVVN 365

Query 363 ETLRLHPGGPLVVRESSKSAV-
VCGYDIPAKTRLFVNVWAIGRDPNHWEKPFEFRPERFI 421
E LRLHP PL+   A V GY IPA T FVN+WAI RDP+ W P +F
PERF+
Sbjct 366
EALRLHPPTPLLAPHRCLEACHVGGYRIPADTLAFVNIWAIHRDPSLWANPLDFCPERFL 425

Query 422 RDGQNQLDVR-GQHYHFIPFGSGRRTCPGASL 452
DV GQH+ F+PFGSGRR+CPG +L
Sbjct 426 ----PSFDVTPGQHFGFLPFGSGRRSCPGWNL 453

Lambda	K	H	a	alpha
0.322	0.138	0.416	0.792	4.96

Gapped

Lambda	K	H	a	alpha	sigma
0.267	0.0410	0.140	1.90	42.6	43.6

Effective search space used: 228942

Query= sp|Q42798|
C93A1_SOYBN_Cytochrome_P450_93A1_OS=Glycine_max_GN=CYP93A1
_PE=2_SV=1

Length=509

Subject= 319623-58_1_ORF2
>sp|Q42798|C93A1_SOYBN_Cytochrome_P450_93A1_OS=Glycine_max_GN=CYP93A
1_PE=2_SV=1|||5e-129

Length=516

Score =   374 bits (960), Expect = 1e-127, Method: Compositional
matrix adjust.
Identities = 205/494 (41%), Positives = 297/494 (60%), Gaps =
24/494 (5%)

Query 19
ILWRKQSKKNLPPSPKALPIIGHLHLVSPIPHQDFYKLSTRHGPIMQLFLGSVPCVVAST 78

```
            IL RK SK  LPP P++LP IGHLHL+   PH     LS  HGP+M L  GSVP
VVAST
Sbjct  35
ILLRKSSKLKLPPGPRSLPFIGHLHLLGSNPHHSLCDLSKTHGPLMYLRFGSVPVVVAST  94

Query  79
AEAAKEFLKTHEINFSNRPGQNVAVKGLAYDSQDFLFAFAPFGPYWKFMKKLCMSELLSG  138
               A+  L+ H+  FS R    VA++    +  + LF+    GPYWK M+++C++
+L
Sbjct  95   PTMARHILQAHDQTFSFRTQPAVAMQ--LNNCVNVLFSQP--
GPYWKLMRQICLTDLFGN  150

Query  139
RMMDQFLPVRQQETKRFISRVFRKGVAGEAVDFGDELMTLSNNIVSRMTLSQKTSE--ND  196
            + ++ F P+  +E    +  +     A E V    +L T ++NI++RM + ++ ++
Sbjct  151  KRLESFRPLITEEVHALLRTIL--
DTAEELVPVRAKLYTATSNIIARMAVGKRLADLATR  208

Query  197  NQAEEMKKLVSNIAE---
LMGKFNVSDFIWYLKPFDLQGFNRKIKETRDRFDVVVDGIIK  253
            +QA+    L++ + E   L+G FNV DFI  L   DLQG   + K    +  +V
+I
Sbjct  209
SQADTSYDLLTLLVEAVHLLGIFNVGDFIPSLAWMDLQGCVSRSKAVGQKLHIVWQDVID  268

Query  254
QRQEERRKNKETGTAKQFKDMLDVLLDMHEDENAEIKLDKKNIKAFIMDIFVAGTDTSAV  313
            +R+E RR++ E   A +  D LDVL+   E    ++++   NI A + D+F
AGTDTS++
Sbjct  269  ERREMRRQSNEI--ASKELDFLDVLMTASE-
RRPDVQITDFNIMAILTDMFAAGTDTSSI  325

Query  314
SIEWAMAELINNPDVLEKARQEIDAVVGKSRMVEESDIANLPYLQAIVRETLRLHPGGPL  373
            S EWA+ EL+ NP  L+K + E++ VVG SR+V+E+DI +LPYL+A+V+ET+RLHP
PL
Sbjct  326
STEWALGELLANPIKLKKVQDELERVVGMSRLVQETDIPHLPYLRAVVKETMRLHPVLPL  385

Query  374  VV-
RESSKSAVVCGYDIPAKTRLFVNVWAIGRDPNHWEKPFEFRPERFIRDGQNQLDVRG  432
            +V  ++S+   + GYDIP  T  +VNVWAIGRDP+ WE+P EF PERF+
DVRG
Sbjct  386  LVPHKASQDCNISGYDIPLGTLAYVNVWAIGRDPSTWERPLEFEPERFL---
DCNTDVRG  442

Query  433
QHYHFIPFGSGRRTCPGASLAWQVVPVNLAIIIQCFQWKLVGGNGKVDMEEKSGITLPRA  492
            QH+  +PFGSGRR CPG  L    V   LA ++ CF W  +    K +M EK GI
+
Sbjct  443  QHFELLPFGSGRRACPGLILGLTNVQYMLASLVHCFDWSAI---
EKPNMSEKFGIVMTLE  499

Query  493  NPIIC---VPVPRI  503
```

```
             NPI+    + VPR+
Sbjct  500  NPIVAKATLRVPRL  513



Lambda      K        H        a         alpha
   0.322    0.138    0.416    0.792     4.96

Gapped
Lambda      K        H        a         alpha    sigma
   0.267   0.0410    0.140     1.90     42.6     43.6

Effective search space used: 228942




Matrix: BLOSUM62
Gap Penalties: Existence: 11, Extension: 1
Neighboring words threshold: 11
Window for multiple hits: 40





Query= sp|Q43078|C97B1_PEA_Cytochrome_P450_97B1,
_chloroplastic_OS=Pisum_sativum_GN=CYP97B1_PE=2_SV=1

Length=552

Subject= 77116-287_1_ORF2
>sp|Q43078|C97B1_PEA_Cytochrome_P450_97B1,
_chloroplastic_OS=Pisum_sativum_GN=CYP97B1_PE=2_SV=1|||0

Length=610


 Score =   763 bits (1969),  Expect = 0.0, Method: Compositional
matrix adjust.
 Identities = 362/507 (71%), Positives = 417/507 (82%), Gaps = 6/507
(1%)

Query  42   SSHSKRFSSIRCQSVNGE----
KRKQSSRNVFDNASNLLTSLLSGANLGSMPIAEGAVTD  97
            SS  +R  ++  +S  G+    K K   R +FDNASNLLT+ L+   L +MP
+AEGAV+D
Sbjct  49
SSTRRRSCTLLTRSQAGQDDIKKGKAEQRTLFDNASNLLTNFLNQGTLANMPVAEGAVSD  108

Query  98
LFDRPLFFSLYDWFLEHGSVYKLAFGPKAFVVVSDPIVARHILRENAFSYDKGVLADILE  157
            LF RPLFF+LYDWF+EHG VYKLAFGPKAF+VVSDPIVARHILRENAF
YDKGVLADILE
```

```
Sbjct  109
LFARPLFFALYDWFIEHGPVYKLAFGPKAFIVVSDPIVARHILRENAFGYDKGVLADILE  168

Query  158  PIMGKGLIPADLETWKQRRRVIAPGFHTSYLEAMVQLFTSCSERTVLKV-
NELLEGEGRD  216
            PIMGKGLIPADL+TWK RRR I PGFHT+YLEAMV +F+ C+ R V KV N LL+
E +
Sbjct  169
PIMGKGLIPADLDTWKVRRRAIVPGFHTAYLEAMVNVFSQCTARAVKKVENMLLDAEAKQ  228

Query  217
GQKSVELDLEAEFSNLALEIIGLGVFNYDFGSVTNESPVIKAVYGTLFEAEHRSTFYIPY  276
             +  VE D+E E+S+LAL+IIGL VFNYDF  +   SPVI+AVYGTL
+EAEHRSTFYIPY
Sbjct  229  AE-
YVEADMEQEYSSLALDIIGLSVFNYDFECINKMSPVIQAVYGTLYEAEHRSTFYIPY  287

Query  277
WKFPLARWIVPRQRKFQDDLKVINTCLDGLIRNAKESRQETDVEKLQQRDYSNLKDASLL  336
            WK P A W+VPRQRKFQ DLKVIN CLDGLI+ A+++RQE D+E LQQRDYS
+KDASLL
Sbjct  288
WKLPFASWVVPRQRKFQKDLKVINDCLDGLIKGARDTRQEEDIEALQQRDYSKVKDASLL  347

Query  337
RFLVDMRGVDVDDRQLRDDLMTMLIAGHETTAAVLTWAVFLLAQNPDKMKKAQAEVDLVL  396
            RFLVDMRG D DD+QLRDDLMTMLIAGHETTAAVLTWA FLLAQNP+K+ KAQ
EVD VL
Sbjct  348
RFLVDMRGEDADDKQLRDDLMTMLIAGHETTAAVLTWATFLLAQNPEKVLKAQEEVDNVL  407

Query  397
GMGKPTFELLKKLEYIRLIVVETLRLYPQPPLLIRRSLKPDVLPGGHKGDKDGYTIPAGT  456
            G   PT++ +K L+YIRLIV E LRLYPQPPLLIRRSL+PD LPGG +GD +GY
IP G
Sbjct  408
GGRIPTWDDIKNLKYIRLIVAEALRLYPQPPLLIRRSLRPDNLPGGFQGDANGYAIPKGV  467

Query  457
DVFISVYNLHRSPYFWDRPNDFEPERFLVQNNNEEVEGWAGFDPSRSPGALYPNEIISDF  516
            D+FISVYNLHRSPYFWD P  FEPERFL + +  ++EGWAGFDP R   +LY NE+
++DF
Sbjct  468
DIFISVYNLHRSPYFWDNPEKFEPERFLQEKSGSDIEGWAGFDPKRGQSSLYANEVMADF  527

Query  517  AFLPFGGGPRKCVGDQFALMESTVALV  543
            AFLPFGGGPRKCVGDQFALMESTVAL
Sbjct  528  AFLPFGGGPRKCVGDQFALMESTVALA  554


Lambda      K        H        a         alpha
   0.321    0.138    0.413    0.792     4.96
```

Gapped
Lambda K H a alpha sigma
0.267 0.0410 0.140 1.90 42.6 43.6

Effective search space used: 296184

Matrix: BLOSUM62
Gap Penalties: Existence: 11, Extension: 1
Neighboring words threshold: 11
Window for multiple hits: 40

Query= sp|Q498J7|
MC6ZA_XENLA_Zygotic_DNA_replication_licensing_factor_mcm6-
A_OS=Xenopus_laevis_GN=zmcm6-a_PE=1_SV=1

Length=823

Subject= 245753-102_2_ORF1
>sp|Q498J7|MC6ZA_XENLA_Zygotic_DNA_replication_licensing_factor_mcm6
-A_OS=Xenopus_laevis_GN=zmcm6-a_PE=1_SV=1|||0

Length=878

Score = 699 bits (1805), Expect = 0.0, Method: Compositional
matrix adjust.
Identities = 383/830 (46%), Positives = 537/830 (65%), Gaps =
46/830 (6%)

```
Query  30   LFQDFLEEFQGS----------DGELK-
YQSDAEELIRPERNTLLVSFVDLEQFNQQLAT  78
            LF DFL  F G           G  K Y+++ E +   E  T+ V F  +  F+
L
Sbjct  58
LFFDFLNSFTGDVATSGVSGEPPGRAKIYETELEVMRSKESTTMFVDFNHVMLFDDALQA  117

Query  79   TIQEEFYRVYPYLCRAVRAFARDHGNI-----
PQNKEFYVAFQDLPTRHKIRELTTPRIG  133
             I EEF R  P+   A + F   H          NK+ ++AF +LPT  K+R+L T
IG
Sbjct  118
AISEEFLRFEPFFSAAAKKFVLQHRPTYFMEEDPNKDVHLAFFNLPTVKKLRDLGTVEIG  177

Query  134
SLLRISAQVVRTHPVHPELVSGTFLCLDCQTLVRDVEQQFKYTQPSICRNPVCANRRRFM  193
             L+ +S  V RT  V PEL+ GTF CLDC  +V++VEQQFKYTQP IC N  C+NR
R+
```

Sbjct 178
KLVSVSGVVTRTSEVRPELLLGTFKCLDCGAVVKNVEQQFKYTQPIICVNATCSNRNRWA 237

Query 194
LDTNKSRFVDFQKVRIQETQAELPRGSIPRSVEVILRAEAVESCQAGDRCDFTGSLIVVP 253
L +S+F D+Q+VR+QE E+P GS+PR++++ILR E VE +AGD+C FTG++
+V+P
Sbjct 238
LLRQESKFADWQRVRMQENSKEIPAGSLPRTLDIILRHEVVEQARAGDKCIFTGTVVVIP 297

Query 254 DISQLSTPGVRAET--SSRVGGRE--
GYEAEGVQGLRALGVRDLSYKLVFLACYVCPTNP 309
D+S L++PG R E S +G R G AEG++GL+ALGVRDLSY+L F+A V
P +
Sbjct 298
DLSALASPGDRTEARRESGMGQRNNAGAGAEGLRGLKALGVRDLSYRLAFVANSVQPQDK 357

Query 310 RF-----
GGKELHEEDMTAESIKNQMSVKEWEKVFEMSQDKNLYHNLCTSLFPTVHGNDE 364
+ GGK+ +ED ES+K + +E E+++ M +Y L S+ P V
G+ +
Sbjct 358 KRSIDFRGGKKDGDED---ESVK--
FTNEEMEQIYRMKSLPQIYERLVNSVSPAVFGHQD 412

Query 365
VKRGILLMLFGGVPKSTMEGTSLRGDINVCVVGDPSTAKSQFLKHVEEFSPRAVYTSGKA 424
+KR ILLMLFGGV K T EG +LRGDINVC+VGDPS AKSQFLK+V F PR
+VYTSGK+
Sbjct 413
IKRAILLMLFGGVHKRTHEGINLRGDINVCIVGDPSCAKSQFLKYVAGFLPRSVYTSGKS 472

Query 425
STAAGLTAAVVKDEESHEFVIEAGALMLADNGVCCIDEFDKMDTKDQVAIHEAMEQQTIS 484
S+AAGLTA+VVK+ E+ EF IEAGALMLADNG+CCIDEFDKMD
KDQVAIHEAMEQQTIS
Sbjct 473
SSAAGLTASVVKEPETGEFCIEAGALMLADNGICCIDEFDKMDIKDQVAIHEAMEQQTIS 532

Query 485
ITKAGVKATLNARTSILAAANPVGGRYDRAKSLKQNVNLSAPIMSRFDLFFILVDECNEV 544
ITKAG++ATLNARTSILAAANP GGRYD++K LK NV L I+SRFDL +++DE
++
Sbjct 533
ITKAGIQATLNARTSILAAANPSGGRYDKSKPLKYNVALPPAILSRFDLVHVMIDEPDDD 592

Query 545
TDYAIARRIVDLHSRIEESIDRVYTVDEVRRYLLFARQFKPKISKESADFIVEQYKRLRQ 604
DY IAR IV +H R EE++ +T+ +++RY+ FAR KPK+S ++ + +VE Y
LR+
Sbjct 593
MDYNIARHIVSVHQRQEEALSPEFTLAQLQRYIAFARTLKPKLSDKAREALVEAYVTLRR 652

Query 605
RDGSGVTKSAWRITVRQLESMIRLSEGMARMHCSDEVQPKHVKEAFRLLNKSIIRVETPD 664

```
            D    ++ A+RITVRQLE++IRLSE +AR+HC  +V+  HV EA RLL  SII V
++ +
Sbjct  653
GDAVPGSQVAYRITVRQLEALIRLSEAIARVHCETQVRRAHVVEAKRLLGTSIISVDSHE  712

Query  665  VNLDQDDEHE-PEDETQEGT-
NGDAEVPNGVNGHVNGINGHSQESNAAAAKPSLRLNFAE  722
            ++LD   E E P+D   + T   +   P     H  G +   +E    A K  +++
+ +
Sbjct  713  IDLDDYQEDEVPDDMLNDVTFTPNGSFPRADESHPAGGDATMEE----
AEKKKMKVTYED  768

Query  723  YKRISNLLVQQLRKMEDEDE-----TSQRRSELMNWYLKEIESE--
IDSEEELINRKQII  775
            +++++  +V +LR+ E+  E        ++++L+ WY++E  ++    S  E++
+ +
Sbjct  769
FQKVTRAIVIRLRQQEESQEDDAGMVGMKQADLVRWYIEEQNTQGLFSSMGEVVEEIRRV  828

Query  776  DKVIHRLVHYDQILI---ELTQTELKGTGDEVVAKEEDPYLVVNPNYILE  822
              +I  L+  + +LI   + +    +  GD+ VA  E   L VNPNY+LE
Sbjct  829  RAIIQHLIKREGVLIVPDDGSGGVEEDGGDQAVAAIERRTLAVNPNYVLE  878



Lambda      K        H        a         alpha
   0.317    0.133    0.379    0.792     4.96

Gapped
Lambda      K        H        a         alpha    sigma
   0.267   0.0410    0.140     1.90     42.6     43.6

Effective search space used: 652916




Matrix: BLOSUM62
Gap Penalties: Existence: 11, Extension: 1
Neighboring words threshold: 11
Window for multiple hits: 40






Query= sp|Q5T9S5|CCD18_HUMAN_Coiled-coil_domain-
containing_protein_18_OS=Homo_sapiens_GN=CCDC18_PE=1_SV=1

Length=1454

Subject= 273590-84_6_ORF1
>sp|Q5T9S5|CCD18_HUMAN_Coiled-coil_domain-
containing_protein_18_OS=Homo_sapiens_GN=CCDC18_PE=1_SV=1|||3e-10
```

Length=1103

Score = 48.1 bits (113), Expect = 4e-09, Method: Compositional matrix adjust.
Identities = 120/466 (26%), Positives = 220/466 (47%), Gaps = 46/466 (10%)

```
Query  735   CKEELVLHLNQLEGNKEKFEKQLKKKSEEVYCLQKELKIKNHSLQETSEQN---
VILQHT  791
               E L LH+  L   + K  +++K+  +    +Q EL+ K     E SE N   +
+LQ
Sbjct  433   AAESLQLHVTDLTAREAKSVERMKEVEDGFQKMQLELQSK----
LEISEANFNSLVLQ--  486

Query  792   LQQQQQMLQQETIRNGELEDTQTKLEKQVSKLEQELQKQRESSAE---
KLRKMEEKCESA  848
             L+++   LQ E++    LE    +  K+ S LE EL+  R+   E   K+R
EEKC
Sbjct  487   LEEKAAQLQ-ESMAAAVLEGEGAR--
KKASDLELELESLRQIQGELEEKVRVSEEKCSQH  543

Query  849
AHEADLKRQKVIELTGTARQVKIEMDQYKEELSKMEKEIMHLKRDGENKAMHLSQLDMIL  908
              + A   R + +EL G   + K + D    +++ +E  ++    D +NKA  L +
+ + L
Sbjct  544   ENSAHTFRTRGVELEGLITEHKTKADDAYGKVASLEAALL----DAQNKAADL-
EMKLKL  598

Query  909   DQTKTELEKKTNA--
VKELEKLQHSTETELTEALQKREVLETELQNAHGELKSTLRQLQE  966
              +  + L   T+A  + ELE+     E ++ E       LET    A    + TL
Sbjct  599   AEDNSLLLGNTSAARIAELERCTKEYEAKVAE-------
LETACAGAKSIEQHTL-----  646

Query  967   LRDVLQKAQLSLEEKYTTIKDLTAELRECKMEIEDKKQEL---
LEMDQALKERNWELKQR  1023
               ++L   +  LEE+ T IK  +  + E ++ +E  + +L   L+ ++ + E
L+++
Sbjct  647   --
EILHSTEKMLEERQTEIKSASQTISELELSVETMESDLKGALKREKLVTEARASLEEK  704

Query  1024
AAQVTHLDMTIREHRGEMEQKIIKLEGTLEKSELELKECNKQIESLNDKLQNAKEQLREK  1083
               ++  L   +   RGE E ++ +    +EK +++L E + Q   L D++ + +
E
Sbjct  705
IVKLEALITQLEGERGEFESRVHQHISLVEKGKVDLHEASVQEGKLKDEISSLQAAKIEL  764

Query  1084  EFIMLQNEQEISQLKKEIERTQQRMKEM----
ESVMKEQEQYIATQYKEAIDLGQELRLT  1139
             E ++ Q E E      +++ TQ+   E+        ++ +Q I T  +E  DL Q
+   T
```

```
Sbjct  765
EDMLSQIEVEKRTTIAQLDSTQKSFVELNEQLVQERQQLQQQITTIMQENGDLAQKFAST  824

Query  1140  REQVQNSHTELAEARHQQVQ-AQREIERLSSELEDMK-QLSKEKDA  1183
             +E++  +    AE   +QVQ    +   L++++ED+  QL K  DA
Sbjct  825   QEKLHGTLA-AAELSAKQVQEGSLKESTLNAQVEDLSLQLRKALDA  869


 Score = 18.5 bits (36),  Expect = 4.0, Method: Compositional matrix
adjust.
 Identities = 21/81 (26%), Positives = 43/81 (53%), Gaps = 5/81 (6%)

Query  143  SIHFELTQSRAKVSMLE--
SAQQQAASVPILEEQIINLEAEVSAQDKVLREAENKLEQSQ  200
            S H +L  S  K+  +E  +AQ++   +  L   +++ E +++   K L  A  +
E  +
Sbjct  70   SDHTQLHHSEHKIGDIELRAAQER---
IEKLHGDLVDAEKKLADSQKKLSAASKQAEDYE  126

Query  201  KMVIEKEQSLQESKEECIKLK  221
            K +  +E+  +E+ +EC +LK
Sbjct  127  KEMKNQEEIAKEALDECSRLK  147


 Score = 18.1 bits (35),  Expect = 5.0, Method: Compositional matrix
adjust.
 Identities = 10/19 (53%), Positives = 11/19 (58%), Gaps = 0/19 (0%)

Query  1377  LKKMQLEQPSTLEESHKNL  1395
              +KMQLE  S LE S  N
Sbjct  462   FQKMQLELQSKLEISEANF  480


 Score = 17.3 bits (33),  Expect = 9.2, Method: Compositional matrix
adjust.
 Identities = 19/73 (26%), Positives = 34/73 (47%), Gaps = 6/73 (8%)

Query  107
APVDQEIKSLREKLNKLRQQNACLVTQNHSLMTKFESIHFELTQSRAKVSMLESAQQQAA  166
            A +++E    REKLN+L    A   ++   L  K ++   EL +    +S  E
Sbjct  196
AKIEEENHLTREKLNQLEGHLADEKSRAAELEGKIQNAEIELKKQEGSLSAAE------G  249

Query  167  SVPILEEQIINLE  179
            +  +++EQ+  LE
Sbjct  250  TAVMVKEQLSALE  262



Lambda      K        H        a         alpha
   0.307    0.123    0.315    0.792     4.96

Gapped
Lambda      K        H        a         alpha    sigma
```

```
   0.267   0.0410    0.140     1.90     42.6     43.6

Effective search space used: 1483330




Query= sp|Q5T9S5|CCD18_HUMAN_Coiled-coil_domain-
containing_protein_18_OS=Homo_sapiens_GN=CCDC18_PE=1_SV=1

Length=1454

Subject= 307683-64_2_ORF2
>sp|Q5T9S5|CCD18_HUMAN_Coiled-coil_domain-
containing_protein_18_OS=Homo_sapiens_GN=CCDC18_PE=1_SV=1|||7e-11

Length=1103


 Score = 50.4 bits (119),  Expect = 7e-10, Method: Compositional
matrix adjust.
 Identities = 121/466 (26%), Positives = 220/466 (47%), Gaps =
46/466 (10%)

Query  735   CKEELVLHLNQLEGNKEKFEKQLKKKSEEVYCLQKELKIKNHSLQETSEQN---
VILQHT  791
               E L LH+  L   + K  +++K+  +    +Q EL+ K     E SE N   +
+LQ
Sbjct  433   AAESLQLHVTDLTAREAKSVERMKEVEDGFQKMQLELQSK----
LEISEANFNSLVLQ--  486

Query  792   LQQQQQMLQQETIRNGELEDTQTKLEKQVSKLEQELQKQRESSAE---
KLRKMEEKCESA  848
             L+++   LQ E++    LE    +  K+ S LE EL+  R+   E   K+R
EEKC
Sbjct  487   LEEKAAQLQ-ESMAAAVLEGEGAR--
KKASDLELELESLRQIQGELEEKVRVSEEKCSQH  543

Query  849
AHEADLKRQKVIELTGTARQVKIEMDQYKEELSKMEKEIMHLKRDGENKAMHLSQLDMIL  908
              + A   R + +EL G   + K + D    +++ +E  ++    D +NKA  L +
+ + L
Sbjct  544   ENSAHTFRTRGVELEGLITEHKTKADDAYGKVASLEAALL----DAQNKAADL-
EMKLKL  598

Query  909   DQTKTELEKKTNA--
VKELEKLQHSTETELTEALQKREVLETELQNAHGELKSTLRQLQE  966
              +  + L   T+A  + ELE+     E ++ E       LET    A    + TL
Sbjct  599   AEDNSLLLGNTSAARIAELERCTKEYEAKVAE-------
LETACAGAKSIEQHTL-----  646

Query  967   LRDVLQKAQLSLEEKYTTIKDLTAELRECKMEIEDKKQEL---
LEMDQALKERNWELKQR  1023
               ++L   +  LEE+ T IK  +  + E ++ +E  + +L   L+ ++ + E
```

L+++
Sbjct 647   --
EILHSTEKMLEERQTEIKSASQTISELELSVETMESDLKGALKREKLVTEARASLEEK 704

Query 1024
AAQVTHLDMTIREHRGEMEQKIIKLEGTLEKSELELKECNKQIESLNDKLQNAKEQLREK 1083
               ++ L   +   RGE E ++ +   +EK +++L E + Q   L D++ +
E
Sbjct 705
IVKLEALITQLEGERGEFESRVHQHISLVEKGKVDLHEASVQEGKLKDEISSLHAAKTEL 764

Query 1084 EFIMLQNEQEISQLKKEIERTQQRMKEM----
ESVMKEQEQYIATQYKEAIDLGQELRLT 1139
             E ++ Q E E   +++ TQ+   E+   ++ +Q IAT +E DL Q
+   T
Sbjct 765
EDMLSQIEVEKRTTIAQLDSTQKSFVELNEQLVQERQQLQQQIATIMQENGDLAQKFAST 824

Query 1140 REQVQNSHTELAEARHQQVQ-AQREIERLSSELEDMK-QLSKEKDA 1183
             +E++ +   AE   +QVQ   +   L++++ED+ QL K DA
Sbjct 825   QEKLHGT-LAAAELSAKQVQEGSLKESNLNTQVEDLNLQLRKALDA 869

Score = 18.5 bits (36), Expect = 3.7, Method: Compositional matrix adjust.
Identities = 21/81 (26%), Positives = 43/81 (53%), Gaps = 5/81 (6%)

Query 143 SIHFELTQSRAKVSMLE--
SAQQQAASVPILEEQIINLEAEVSAQDKVLREAENKLEQSQ 200
            S H +L S K+ +E +AQ++   + L   +++ E +++   K L A +
E +
Sbjct 70   SDHTQLHHSEHKIGDIELRAAQER---
IEKLHGDLVDAEKKLADSQKKLSAASKQAEDYE 126

Query 201 KMVIEKEQSLQESKEECIKLK 221
            K + +E+ +E+ +EC +LK
Sbjct 127 KEMKNQEEIAKEALDECSRLK 147

Score = 18.1 bits (35), Expect = 5.1, Method: Compositional matrix adjust.
Identities = 10/19 (53%), Positives = 11/19 (58%), Gaps = 0/19 (0%)

Query 1377 LKKMQLEQPSTLEESHKNL 1395
              +KMQLE S LE S N
Sbjct 462   FQKMQLELQSKLEISEANF 480

Score = 17.3 bits (33), Expect = 9.1, Method: Compositional matrix adjust.
Identities = 19/73 (26%), Positives = 34/73 (47%), Gaps = 6/73 (8%)

Query 107
APVDQEIKSLREKLNKLRQQNACLVTQNHSLMTKFESIHFELTQSRAKVSMLESAQQQAA 166

```
            A +++E    REKLN+L    A   ++   L  K ++   EL +    +S  E
Sbjct  196
AKIEEENHLTREKLNQLEGHLADEKSRAAELEGKIQNAEIELKKQEGSLSAAE------G  249

Query  167  SVPILEEQIINLE  179
            +  +++EQ+  LE
Sbjct  250  TAVMVKEQLSALE  262



Lambda      K        H        a         alpha
   0.307    0.123    0.315    0.792     4.96

Gapped
Lambda      K        H        a         alpha    sigma
   0.267   0.0410    0.140     1.90     42.6     43.6

Effective search space used: 1483330




Matrix: BLOSUM62
Gap Penalties: Existence: 11, Extension: 1
Neighboring words threshold: 11
Window for multiple hits: 40





Query= sp|Q5XK83|MCM4A_XENLA_DNA_replication_licensing_factor_mcm4-
A_OS=Xenopus_laevis_GN=mcm4-a_PE=1_SV=1

Length=858

Subject= 286223-76_1_ORF1
>sp|Q5XK83|MCM4A_XENLA_DNA_replication_licensing_factor_mcm4-
A_OS=Xenopus_laevis_GN=mcm4-a_PE=1_SV=1|||0

Length=874


 Score =   706 bits (1823),  Expect = 0.0, Method: Compositional
matrix adjust.
 Identities = 378/805 (47%), Positives = 524/805 (65%), Gaps =
50/805 (6%)

Query  84   PLTYGTPSSRVEGTPRSGIRGTPARQRAD----
LGSARKVKQVDLHSDQPAAEELVTSEQ  139
            PL  GTP+S          RGTP+ QR D        ++ +QV   +    +E
+SE
Sbjct  90   PLDLGTPASS------QFARGTPSYQRNDSSVPANGNKRWQQVP-
ATPSSYSEGRPSSEG  142
```

```
Query  140  SLGQK--
LVIWGTDVNVAICKEKFQRFVQRFIDPLAKEEENVGLDLNEPIYMQRLEEINV  197
             L      ++WGT+++V    +  QRF++ + D           DL E  Y+Q LE
++
Sbjct  143  DLDAAPPTLVWGTNISVQDVNQAVQRFLRNYRDNPT--------DL-
EAKYLQLLEQVME  193

Query  198
VGEPFLNIDCDHLRSFDQDLYRQLVCYPQEVIPTFDMAANEIFFERYPDSILEHQIQVRP  257
            + E  LN++  ++  +D+DLY ++V YP EV+  FD+   ++     P  + E
IQVR
Sbjct  194  LEEDSLNVNARNILEYDEDLYVKMVRYPLEVLAIFDIVLMDMAVRLRP--
LWEKHIQVRI  251

Query  258  YNALKTRNMRSLNPEDIDQLITISGMVIRTSQIIPEMQESFFKCQVCAFTTRVE-
IDRGR  316
            +N   T NMR LNP DI++++++ GM+IR S IIPE++ESFF+C VC F+ +
+DRGR
Sbjct  252
FNLKDTVNMRDLNPSDIEKMVSVKGMIIRCSSIIPEIKESFFRCLVCGFSPQPSGVDRGR  311

Query  317  IAEPSVCK--
HCNTTHSMALIHNRSMFSDKQMIKLQESPEDMPAGQTPHTTILYAHNDLV  374
            + EP+ C    C   +SMALIHNR  F+DKQ+++LQE+P+ +P G+TPHT  L  H
+ LV
Sbjct  312
VEEPTRCGRPECAALNSMALIHNRCRFADKQIVRLQETPDSIPDGETPHTVSLLMHDQLV  371

Query  375  DKVQPGDRVNVTGIYRAVPIRVNPRVRNVKSVYKTHIDVIHYRKTDSKRLHG---
IDEDT  431
            D  +PGDRV VTG++RA+ +RV P  R +KS++KT+ID +H +K+D  R+     +
+ D
Sbjct  372
DAAKPGDRVEVTGVFRAMSVRVGPTQRTLKSLFKTYIDCLHLKKSDKSRMQSEDPLERDV  431

Query  432  EQKMFT------------
EERVAVLKELAAKPDIYERLAAALAPSIYEHEDIKKGILLQL  479
            + + +            EE++  LKEL+  PDIYERL  +LAPSI+E +DIKKG
+L QL
Sbjct  432
QNENYASFHEGDVSPLEHEEQIQKLKELSMLPDIYERLTRSLAPSIWELDDIKKGLLCQL  491

Query  480
FGGTRKDFSHTGRGKFRAEVNILLCGDPGTSKSQLLQYVYNLVPRGQYTSGKGSSAVGLT  539
            FGGT K         FR ++N+LL GDPGTSKSQLLQYV+ L PRG YTSG
+GSSAVGLT
Sbjct  492  FGGTGKKLKSGA--
SFRGDMNVLLVGDPGTSKSQLLQYVHKLAPRGIYTSGRGSSAVGLT  549

Query  540
AYVMKDPETRQLVLQTGALVLSDNGICCIDEFDKMNESTRSVLHEVMEQQTLSIAKAGII  599
            AYV KDPET + VL++GALVLSD GICCIDEFDKM+++ RS+LHEVMEQQT+S+AK
+GII
```

```
Sbjct  550  
AYVTKDPETGETVLESGALVLSDRGICCIDEFDKMSDNARSMLHEVMEQQTVSVAKSGII  609

Query  600  
CQLNARTSVLAAANPVESQWNPKKTTIENIQLPHTLLSRFDLIFLMLDPQDETYDRRLAH  659
              LNARTSVLA ANP  S++NP+ + I+NIQLP TLLSRFDLI+L+LD  DE  
DRRLA 
Sbjct  610  
ATLNARTSVLACANPSGSRYNPRMSVIDNIQLPPTLLSRFDLIYLVLDKPDEHTDRRLAR  669

Query  660  
HLVALYYQSEEQLKEEHLDMAVLKDYIAYARTYVNPRLGEEASQALIEAYVDMRKI----  715
            HLVAL+Y+  E   +E LD+  L  YI YAR ++ P+L +EA++ LI  YV MR+    
Sbjct  670  
HLVALHYEDPEIRTQESLDLPTLTAYITYARQHIQPKLSDEAAEDLISGYVAMRRKGNFP  729

Query  716  
GSGRGMVSAYPRQLESLIRLSEAHAKVRFSSKVETIDVEEAKRLHREALKQSATDPRTGI  775
            GS + +++A PRQLESLIR+SEA A++R+S +VE  D  EA RL   AL+QSATD  
TG
Sbjct  730  
GSSKKVITATPRQLESLIRISEALARIRYSEQVERCDAAEAIRLLEVALQQSATDHSTGT  789

Query  776  VDISILTTGMSATARKRKEELAQVLKKLIQSKGKT--
PAFKYQQLFEDLRGQSDAAITKD  833
            +D+ ++TTG+SA+ R R+  L   ++ LI  K +      +  QL E+LR  S  
++
Sbjct  790  
IDMDLITTGVSASERTRRASLVSAVRSLISEKMQAGGSVMRVAQLLEELRKDSSTELSLQ  849

Query  834  MFDEALHALADEDYLTVTGKTVRLL  858
                 AL  LA E  ++  G  ++ L
Sbjct  850  DLRTALGNLAGEGVISFQGDGIKRL  874


 Score = 17.3 bits (33),  Expect = 3.7, Method: Compositional matrix 
adjust.
 Identities = 7/13 (54%), Positives = 10/13 (77%), Gaps = 0/13 (0%)

Query  790  RKRKEELAQVLKK  802
            R+RK ELA V+ +
Sbjct  3    RERKRELAIVMAR  15



Lambda      K        H        a         alpha
   0.317    0.132    0.375    0.792     4.96

Gapped
Lambda      K        H        a         alpha    sigma
   0.267   0.0410    0.140     1.90     42.6     43.6

Effective search space used: 678912
```

Query= sp|Q5XK83|MCM4A_XENLA_DNA_replication_licensing_factor_mcm4-A_OS=Xenopus_laevis_GN=mcm4-a_PE=1_SV=1

Length=858

Subject= 286430-76_1_ORF1
>sp|Q5XK83|MCM4A_XENLA_DNA_replication_licensing_factor_mcm4-A_OS=Xenopus_laevis_GN=mcm4-a_PE=1_SV=1|||0

Length=874

Score = 706 bits (1823), Expect = 0.0, Method: Compositional matrix adjust.
Identities = 378/805 (47%), Positives = 524/805 (65%), Gaps = 50/805 (6%)

```
Query  84   PLTYGTPSSRVEGTPRSGIRGTPARQRAD----
LGSARKVKQVDLHSDQPAAEELVTSEQ  139
            PL  GTP+S          RGTP+ QR D        ++ +QV   +    +E
+SE
Sbjct  90   PLDLGTPASS------QFARGTPSYQRNDSSVPANGNKRWQQVP-
ATPSSYSEGRPSSEG  142

Query  140  SLGQK--
LVIWGTDVNVAICKEKFQRFVQRFIDPLAKEEENVGLDLNEPIYMQRLEEINV  197
             L      ++WGT+++V    +  QRF++ + D           DL E  Y+Q LE
++
Sbjct  143  DLDAAPPTLVWGTNISVQDVNQAVQRFLRNYRDNPT--------DL-
EAKYLQLLEQVME  193

Query  198
VGEPFLNIDCDHLRSFDQDLYRQLVCYPQEVIPTFDMAANEIFFERYPDSILEHQIQVRP  257
            + E  LN++  ++  +D+DLY ++V YP EV+  FD+   ++     P  + E
IQVR
Sbjct  194  LEEDSLNVNARNILEYDEDLYVKMVRYPLEVLAIFDIVLMDMAVRLRP--
LWEKHIQVRI  251

Query  258  YNALKTRNMRSLNPEDIDQLITISGMVIRTSQIIPEMQESFFKCQVCAFTTRVE-
IDRGR  316
            +N   T NMR LNP DI++++++ GM+IR S IIPE++ESFF+C VC F+ +
+DRGR
Sbjct  252
FNLKDTVNMRDLNPSDIEKMVSVKGMIIRCSSIIPEIKESFFRCLVCGFSPQPSGVDRGR  311

Query  317  IAEPSVCK--
HCNTTHSMALIHNRSMFSDKQMIKLQESPEDMPAGQTPHTTILYAHNDLV  374
            + EP+ C    C   +SMALIHNR  F+DKQ+++LQE+P+ +P G+TPHT  L  H
+ LV
Sbjct  312
VEEPTRCGRPECAALNSMALIHNRCRFADKQIVRLQETPDSIPDGETPHTVSLLMHDQLV  371
```

```
Query  375  DKVQPGDRVNVTGIYRAVPIRVNPRVRNVKSVYKTHIDVIHYRKTDSKRLHG---
IDEDT  431
            D  +PGDRV VTG++RA+ +RV P  R +KS++KT+ID +H +K+D  R+     +
+ D
Sbjct  372
DAAKPGDRVEVTGVFRAMSVRVGPTQRTLKSLFKTYIDCLHLKKSDKSRMQSEDPLERDV  431

Query  432  EQKMFT------------
EERVAVLKELAAKPDIYERLAAALAPSIYEHEDIKKGILLQL  479
            + + +             EE++  LKEL+  PDIYERL  +LAPSI+E +DIKKG
+L QL
Sbjct  432
QNENYASFHEGDVSPLEHEEQIQKLKELSMLPDIYERLTRSLAPSIWELDDIKKGLLCQL  491

Query  480
FGGTRKDFSHTGRGKFRAEVNILLCGDPGTSKSQLLQYVYNLVPRGQYTSGKGSSAVGLT  539
            FGGT K         FR ++N+LL GDPGTSKSQLLQYV+ L PRG YTSG
+GSSAVGLT
Sbjct  492  FGGTGKKLKSGA--
SFRGDMNVLLVGDPGTSKSQLLQYVHKLAPRGIYTSGRGSSAVGLT  549

Query  540
AYVMKDPETRQLVLQTGALVLSDNGICCIDEFDKMNESTRSVLHEVMEQQTLSIAKAGII  599
            AYV KDPET + VL++GALVLSD GICCIDEFDKM+++ RS+LHEVMEQQT+S+AK
+GII
Sbjct  550
AYVTKDPETGETVLESGALVLSDRGICCIDEFDKMSDNARSMLHEVMEQQTVSVAKSGII  609

Query  600
CQLNARTSVLAAANPVESQWNPKKTTIENIQLPHTLLSRFDLIFLMLDPQDETYDRRLAH  659
              LNARTSVLA ANP  S++NP+ + I+NIQLP TLLSRFDLI+L+LD  DE
DRRLA
Sbjct  610
ATLNARTSVLACANPSGSRYNPRMSVIDNIQLPPTLLSRFDLIYLVLDKPDEHTDRRLAR  669

Query  660
HLVALYYQSEEQLKEEHLDMAVLKDYIAYARTYVNPRLGEEASQALIEAYVDMRKI----  715
            HLVAL+Y+  E   +E LD+  L  YI YAR ++ P+L +EA++ LI  YV MR+
Sbjct  670
HLVALHYEDPEIRTQESLDLPTLTAYITYARQHIQPKLSDEAAEDLISGYVAMRRKGNFP  729

Query  716
GSGRGMVSAYPRQLESLIRLSEAHAKVRFSSKVETIDVEEAKRLHREALKQSATDPRTGI  775
            GS + +++A PRQLESLIR+SEA A++R+S +VE  D  EA RL   AL+QSATD
TG
Sbjct  730
GSSKKVITATPRQLESLIRISEALARIRYSEQVERCDAAEAIRLLEVALQQSATDHSTGT  789

Query  776  VDISILTTGMSATARKRKEELAQVLKKLIQSKGKT--
PAFKYQQLFEDLRGQSDAAITKD  833
            +D+ ++TTG+SA+ R R+  L   ++ LI  K +      +  QL E+LR  S
++
Sbjct  790
```

```
IDMDLITTGVSASERTRRASLVSAVRSLISEKMQAGGSVMRVAQLLEELRKDSSTELSLQ  849

Query  834  MFDEALHALADEDYLTVTGKTVRLL  858
                AL  LA E  ++  G  ++ L
Sbjct  850  DLRTALGNLAGEGVISFQGDGIKRL  874


 Score = 17.3 bits (33),  Expect = 3.7, Method: Compositional matrix
adjust.
 Identities = 7/13 (54%), Positives = 10/13 (77%), Gaps = 0/13 (0%)

Query  790  RKRKEELAQVLKK  802
            R+RK ELA V+ +
Sbjct  3    RERKRELAIVMAR  15



Lambda      K        H        a         alpha
   0.317    0.132    0.375    0.792     4.96

Gapped
Lambda      K        H        a         alpha    sigma
   0.267   0.0410    0.140     1.90     42.6     43.6

Effective search space used: 678912



Matrix: BLOSUM62
Gap Penalties: Existence: 11, Extension: 1
Neighboring words threshold: 11
Window for multiple hits: 40



Query= sp|Q6F6N3|
DNAK_ACIAD_Chaperone_protein_DnaK_OS=Acinetobacter_sp._(st
rain_ADP1)_GN=dnaK_PE=3_SV=1

Length=647

Subject= 136649-209_1_ORF2
>sp|Q6F6N3|DNAK_ACIAD_Chaperone_protein_DnaK_OS=Acinetobacter_sp._(s
train_ADP1)_GN=dnaK_PE=3_SV=1|||0

Length=726


 Score =   706 bits (1823),  Expect = 0.0, Method: Compositional
matrix adjust.
 Identities = 358/593 (60%), Positives = 462/593 (78%), Gaps = 8/593
```

(1%)

```
Query  4    IIGIDLGTTNSCVAVLEGDKVKVIENAEGTRTTPSIVAYKD-
SEILVGQSAKRQAVTNPK  62
            IIGIDLGTTNSCVAV+EG   +VIENAEG RTTPS+VA+    E LVG
AKRQAVTN
Sbjct  105
IIGIDLGTTNSCVAVMEGKAPRVIENAEGARTTPSVVAFTSKGERLVGIPAKRQAVTNAS  164

Query  63
NTLFAIKRLIGRRYEDQAVQKDIGLVPYKIIKADNGDAWVEVNDKKLAPQQVSAEILKKM  122
            NT+F +KRLIGR+++D   QK++ +VP+KI+KA++GDAWVE   +KL+P QV A
+L+KM
Sbjct  165
NTVFGVKRLIGRKFDDAQTQKEMKMVPFKIVKAESGDAWVEAGGQKLSPSQVGAFVLQKM  224

Query  123
KKTAEDYLGETVTEAVITVPAYFNDAQRQATKDAGRIAGLDVKRIINEPTAAALAFGMDK  182
            K+TAE YLG  V +AVITVPAYFNDAQRQATKDAGRIAGL+V RIINEPTAAAL+
+G+++
Sbjct  225
KETAEGYLGRGVAKAVITVPAYFNDAQRQATKDAGRIAGLEVSRIINEPTAAALSYGLER  284

Query  183
KEGDRKVAVYDLGGGTFDVSIIEIADLDGDQQIEVLSTNGDTFLGGEDFDTALIDYLVEE  242
            KEG   +AVYDLGGGTFDVSI+E+  LDG    EV +TNGDTFLGGEDFD AL++
LV E
Sbjct  285  KEG--VIAVYDLGGGTFDVSILEM--LDG--
VFEVKATNGDTFLGGEDFDNALLNELVRE  338

Query  243
FKKEQSVNLKNDPLALQRLKEAAEKAKIELSSSSSTEINLPYITADATGPKHLVINVTRA  302
            F K++++++  D LALQRL+EAAEKAK+ELS++  T+I LP++TADA+GPKH  I
+TR+
Sbjct  339
FNKQENMDITMDKLALQRLREAAEKAKVELSTAMQTDIQLPFLTADASGPKHFNITITRS  398

Query  303
KLEGLVADLVARTIEPCRIALKDAGLSTSDISDVILVGGQSRMPMVQQKVQEFFGKEPRK  362
            KLEGLV +L+ RT +PC   L+DAG+S +D+ +V+LVGG +RMP V+  V
+FFGKE  K
Sbjct  399
KLEGLVLNLIERTRKPCNDCLEDAGMSPNDLDEVLLVGGMTRMPKVKDFVAKFFGKEASK  458

Query  363
DVNPDEAVAIGAAIQGAVLSGDKTDVLLLDVTPLTLGIETMGGVLTPIIEKNTTIPAKKS  422
             VN DEAVA+GAAIQG VL GD  D+LLLDVTPL++G+ET+GG+ T +IE
+NTTIPAKKS
Sbjct  459
GVNADEAVAMGAAIQGGVLRGDVKDILLLDVTPLSIGLETLGGIFTKLIERNTTIPAKKS  518

Query  423
QVFSTAADNQPAVDISVYQGERKMAQQNKLLGNFQLGDIPPAPRGVPQIEVSFDINADGI  482
            Q+FST  D Q  V +SV+QGERKMA  NKLLG F+L DIP APRG  QIEV+F I
```

```
+A+GI
Sbjct  519
QMFSTTRDRQTVVGVSVFQGERKMAADNKLLGQFELRDIPSAPRGQLQIEVTFSIDANGI  578

Query  483
LKVSAKDKSTGKEQSIQIKANSGLSDAEIEAMIKDAEANAEEDRKFEELAKARNEADALV  542
            ++V+A +KSTGKEQ+I I+++ GL++ EI+ M+KDAE  AEED K   +A A+  A
+ ++
Sbjct  579
VQVTACEKSTGKEQAITIQSSGGLTEDEIQRMVKDAETYAEEDEKRRAVADAKANAELVM  638

Query  543  SSSNKAVKDLGDKVTEDEKTA-ITTAVSELEAATKENDVEDIKAKTEALQNIL
594
            + + K V++  +K    E  A IT AV+ L  +T   + E+I   T  LQ  L
Sbjct  639  AQAEKKVQEDKEKEGTSESLANITKAVAALRKSTFGENAEEITKNTLLLQQAL
691



Lambda      K        H        a         alpha
   0.311    0.130    0.348    0.792     4.96

Gapped
Lambda      K        H        a         alpha    sigma
   0.267   0.0410    0.140     1.90     42.6     43.6

Effective search space used: 417696



Matrix: BLOSUM62
Gap Penalties: Existence: 11, Extension: 1
Neighboring words threshold: 11
Window for multiple hits: 40



Query= sp|Q6VTH5|
RSPH1_CYPCA_Radial_spoke_head_1_homolog_OS=Cyprinus_carpio
_GN=rsph1_PE=1_SV=1

Length=218

Subject= 338575-49_3_ORF1
>sp|Q6VTH5|RSPH1_CYPCA_Radial_spoke_head_1_homolog_OS=Cyprinus_carpi
o_GN=rsph1_PE=1_SV=1|||2e-09

Length=883


 Score = 45.1 bits (105),  Expect = 1e-09, Method: Compositional
```

```
matrix adjust.
 Identities = 51/186 (27%), Positives = 78/186 (42%), Gaps = 46/186
(25%)

Query  57   TYKFKNGARYTGEWYMNLKHGQGVLYYP-
DGSKYEGSWVDDQRQGHGVY--------TYP  107
            +Y FKNG+ Y G  + +L HG+GV     D  +YEG W  +  QGHG+
P
Sbjct  211
SYLFKNGSSYEGTVWDDLAHGKGVYTTAFDICRYEGEWFQNMMQGHGMIEVDLPVDEPIP  270

Query  108  NG------------------DTYDGEWL------
HHQRHGQGTYTHQETGSQYRGTWVVG  143
            +                   D +D EWL        + HG+   + +E     R
W+
Sbjct  271  DSEEAMKAKEEGQILRSDYMDPFDREWLKMDIEEQFEEHGRTMPSWEE-----
RQGWI--  323

Query  144  NMESTGELIQLNH-RYHGNFVNNNPSGPGKYVFDIGCEQHGEYF--
QLEPDKGEAEEDET  200
              E  GEL +  H +Y G + +N   G G Y  + G    G+++  +L PD  E
+ +
Sbjct  324  --EEFGELPEKGHYKYAGQWKHNRFHGCGVYEIN-
GRSLWGKFYFGELLPDAEECNVEMS  380

Query  201  LISTTL  206
             I  +L
Sbjct  381  AIHASL  386


 Score = 14.2 bits (25),  Expect = 7.9, Method: Compositional matrix
adjust.
 Identities = 7/22 (32%), Positives = 8/22 (36%), Gaps = 0/22 (0%)

Query  116  WLHHQRHGQGTYTHQETGSQYR  137
            W H  R G     HQ   +  R
Sbjct  771  WHHRFRLGNMGSEHQSVLTTPR  792



Lambda      K        H        a         alpha
   0.311    0.135    0.426    0.792     4.96

Gapped
Lambda      K        H        a         alpha    sigma
   0.267   0.0410    0.140     1.90     42.6     43.6

Effective search space used: 158286




Matrix: BLOSUM62
Gap Penalties: Existence: 11, Extension: 1
```

Neighboring words threshold: 11
Window for multiple hits: 40

Query= sp|Q7XSQ9|PIP12_ORYSJ_Probable_aquaporin_PIP1-2_OS=Oryza_sativa_subsp._japonica_GN=PIP1-2_PE=2_SV=3

Length=288

Subject= 208825-130_3_ORF2
>sp|Q7XSQ9|PIP12_ORYSJ_Probable_aquaporin_PIP1-2_OS=Oryza_sativa_subsp._japonica_GN=PIP1-2_PE=2_SV=3|||4e-173

Length=297

Score = 473 bits (1216), Expect = 4e-173, Method: Compositional matrix adjust.
Identities = 230/292 (79%), Positives = 254/292 (87%), Gaps = 7/292 (2%)

```
Query  1
MEGKEEDVRLGANKFSERQPIGTAAQGSDDKDYKEPPPAPLFEPGELKSWSFYRAGIAEF  60
            ME K+EDVRLGANKF+ERQP+GTAAQ    +DY EPP   L EP E  SWSF
+RAGIAEF
Sbjct  8    MESKDEDVRLGANKFNERQPLGTAAQ---
TRDYTEPPATRLIEPSEFTSWSFWRAGIAEF  64

Query  61   MATFLFLYITVLTVMGVNNST----
SKCATVGIQGIAWSFGGMIFALVYCTAGISGGHIN  116
             AT LFLYIT+ TVMG  + T    ++C  VGIQGIAW
+FGGMIFALVYCTAGISGGHIN
Sbjct  65
FATLLFLYITIQTVMGYKHGTPSAKNECPGVGIQGIAWAFGGMIFALVYCTAGISGGHIN  124

Query  117
PAVTFGLFLARKLSLTRALFYMVMQCLGAICGAGVVKGFQKGLYETTGGGANVVAPGYTK  176
            PAVT+GLFLARK+SL R ++Y++MQCLGAICGAG+VKGFQ   YE  GGGAN
VAPGYTK
Sbjct  125
PAVTWGLFLARKVSLPRTVYYIIMQCLGAICGAGIVKGFQPSFYEDNGGGANSVAPGYTK  184

Query  177
GDGLGAEIVGTFILVYTVFSATDAKRNARDSHVPILAPLPIGFAVFLVHLATIPITGTGI  236
            GDGLGAEIVGTF+LVYTVFSATDAKR+ARDSHVP
+LAPLPIGFAVFLVHLATIPITGTGI
Sbjct  185
GDGLGAEIVGTFVLVYTVFSATDAKRSARDSHVPLLAPLPIGFAVFLVHLATIPITGTGI  244

Query  237  NPARSLGAAIIYNRGHAWDDHWIFWVGPFIGAALAAIYHQVVIRAIPFKSRS
288
```

```
            NPARSLGAAIIYN+ HAWDDHWIFWVGPFIGA+LA  YH +VIRA+PFKSR
Sbjct  245  NPARSLGAAIIYNKKHAWDDHWIFWVGPFIGASLACAYHVIVIRALPFKSRE
296



Lambda      K        H        a         alpha
   0.324    0.141    0.439    0.792     4.96

Gapped
Lambda      K        H        a         alpha    sigma
   0.267   0.0410    0.140     1.90     42.6     43.6

Effective search space used: 71002




Matrix: BLOSUM62
Gap Penalties: Existence: 11, Extension: 1
Neighboring words threshold: 11
Window for multiple hits: 40





Query= sp|Q8DKR7|
DNAJ_THEEB_Chaperone_protein_DnaJ_OS=Thermosynechococcus_e
longatus_(strain_BP-1)_GN=dnaJ_PE=3_SV=2

Length=373

Subject= 10515-736_3_ORF1
>sp|Q8DKR7|DNAJ_THEEB_Chaperone_protein_DnaJ_OS=Thermosynechococcus_
elongatus_(strain_BP-1)_GN=dnaJ_PE=3_SV=2|||6e-131

Length=495


 Score =   353 bits (906),  Expect = 8e-122, Method: Compositional
matrix adjust.
 Identities = 188/365 (52%), Positives = 246/365 (67%), Gaps =
15/365 (4%)

Query  1    MAR-
DFYEILGVSRSADAEELKRAYRRLARKYHPDVNKEPGAEEKFKEINRAYEVLSDPQ  59
            MA+ D+Y +LGVS++A   ++K +YR+LAR+ HPDVNKEP AE KFKEI+ AYEVL
D +
Sbjct  131
MAKADYYSVLGVSKNASKADIKSSYRKLARQCHPDVNKEPDAEAKFKEISNAYEVLCDDE  190

Query  60   ARANYDRFGEAGVSGVGAAGFSDFGIGDMGGFADIFETFFGGFTTSSRR-------
QQGP  112
```

```
             R  YD++GEAG+ G GA              D+FETFFGG
+  P
Sbjct  191  KRPIYDQYGEAGLKGAGAGASG------
FSNPFDLFETFFGGAGMGGMGGMGGRSARNRP  244

Query  113
TRGEDLRYDLKLEFREAVFGGEKEIRINHLETCKTCQGTGAKPGTRPVTCSTCGGVGQVR  172
             +G D  YDL++EF EAVFG  K+I ++ LETC TC G+GAKPGT P +CS CGG
GQV
Sbjct  245
MQGADEEYDLRIEFLEAVFGTTKDIDVSRLETCNTCDGSGAKPGTSPKSCSRCGGQGQVV  304

Query  173
RSARTPFGSFTQLTTCPTCGGSGVVIEDRCESCGGQGHIQVSKKLKITIPAGVDNGTRLR  232
             SARTP G F Q++TCP+C G G      C  C G G  +  K + + +PAGVD+G
+RLR
Sbjct  305  SSARTPLGEFRQISTCPSCSGVGET-
STPCTMCRGDGRERKKKSISLKVPAGVDSGSRLR  363

Query  233
VSGEGDAGLRGGPPGDLYVYLFVQPDPEFQREGNNILSRIKISYLQAILGCRISVSTVDG  292
            V  EG+AG RGGPPGDLYV++ VQ  P   R+GNNIL+  ++SY+ AILG    V
TVDG
Sbjct  364
VRSEGNAGKRGGPPGDLYVFISVQAHPTLSRDGNNILTTCRVSYIDAILGTSKKVPTVDG  423

Query  293
EAELKIPAGTQPGTVLVLEGRGVPRVGNPVARGDHLITVDVEIPTHITHEERELLEKLAK  352
            + +LKIPAGTQPGT LV+  +GVP +G P  RGD L+ V+VEIP +++ EER+L++
+LA
Sbjct  424
DTDLKIPAGTQPGTTLVMSKKGVPVLGKPSIRGDQLVRVEVEIPKYLSGEERKLIDQLAD  483

Query  353  IRGER  357
            ++  R
Sbjct  484  LKKVR  488


 Score = 15.8 bits (29),  Expect = 2.7, Method: Compositional matrix
adjust.
 Identities = 8/29 (28%), Positives = 13/29 (45%), Gaps = 0/29 (0%)

Query  55   LSDPQARANYDRFGEAGVSGVGAAGFSDF  83
            L+ P  R+N      +G   + +   SDF
Sbjct  76   LASPFPRSNGTHHSSSGKERMVSLSASDF  104



Lambda      K        H        a         alpha
   0.318    0.140    0.418    0.792     4.96

Gapped
Lambda      K        H        a         alpha    sigma
   0.267   0.0410    0.140     1.90     42.6     43.6
```

Effective search space used: 157883

Query= sp|Q8DKR7|
DNAJ_THEEB_Chaperone_protein_DnaJ_OS=Thermosynechococcus_e
longatus_(strain_BP-1)_GN=dnaJ_PE=3_SV=2

Length=373

Subject= 2698-1257_3_ORF2
>sp|Q8DKR7|DNAJ_THEEB_Chaperone_protein_DnaJ_OS=Thermosynechococcus_
elongatus_(strain_BP-1)_GN=dnaJ_PE=3_SV=2|||7e-138

Length=455

Score = 370 bits (949), Expect = 8e-129, Method: Compositional
matrix adjust.
Identities = 195/361 (54%), Positives = 251/361 (70%), Gaps =
17/361 (5%)

```
Query  4
DFYEILGVSRSADAEELKRAYRRLARKYHPDVNKEPGAEEKFKEINRAYEVLSDPQARAN  63
            D+Y ILGVS++A   E+K +YR+LAR+YHPDVNKE GAE+KFKEI+ AYEVLSD +
R+
Sbjct  92
DYYSILGVSKNASKPEIKSSYRKLARQYHPDVNKESGAEQKFKEISNAYEVLSDDEKRSI  151

Query  64   YDRFGEAGVSGVGAAGFSDFGIGDMGGFADIFETFFGGFTTSSRR-----------
QQGP  112
            YD+FGEAG+ G GA G + F         D+FETFFGG
+
Sbjct  152  YDKFGEAGLKGAGAGGAAGFS-----
NPFDLFETFFGGMGGMGGMGGMGGMGGRAARNRA  206

Query  113
TRGEDLRYDLKLEFREAVFGGEKEIRINHLETCKTCQGTGAKPGTRPVTCSTCGGVGQVR  172
             +G+D RYDL L F++AVFG +KEI +  LE+C TC G+GAKPGT PVTC+TCGG
GQV
Sbjct  207
VQGDDERYDLLLNFKDAVFGTDKEIEVTRLESCATCNGSGAKPGTSPVTCTTCGGQGQVA  266

Query  173
RSARTPFGSFTQLTTCPTCGGSGVVIEDRCESCGGQGHIQVSKKLKITIPAGVDNGTRLR  232
             +  TP G F Q++TCP CGG+G      C +C G G ++ +KK+ + +PAGVD+G
+RLR
Sbjct  267  TTTNTPLGQFRQISTCPVCGGTGESFTP-
CNACAGDGRVRRTKKISLKVPAGVDSGSRLR  325

Query  233
VSGEGDAGLRGGPPGDLYVYLFVQPDPEFQREGNNILSRIKISYLQAILGCRISVSTVDG  292
```

```
            V  EG+AG RGGPPGDLYV++ V+ D E +R+GNNIL   KISY+ AILG  + V
TVDG
Sbjct  326
VRSEGNAGKRGGPPGDLYVFINVRQDSELKRDGNNILITCKISYIDAILGTTVKVPTVDG  385

Query  293
EAELKIPAGTQPGTVLVLEGRGVPRVGNPVARGDHLITVDVEIPTHITHEERELLEKLAK  352
              +LKIPAGTQPGT LV+  RGVP +G    RGD L+ V VEIP  ++ EER+L+E
+LA
Sbjct  386
VVDLKIPAGTQPGTTLVMAKRGVPYLGKTNVRGDQLVKVQVEIPKRLSTEERKLVEELAN  445

Query  353  I  353
            +
Sbjct  446  L  446



Lambda      K        H        a         alpha
   0.318    0.140    0.418    0.792     4.96

Gapped
Lambda      K        H        a         alpha    sigma
   0.267   0.0410    0.140     1.90     42.6     43.6

Effective search space used: 157883




Query= sp|Q8DKR7|
DNAJ_THEEB_Chaperone_protein_DnaJ_OS=Thermosynechococcus_e
longatus_(strain_BP-1)_GN=dnaJ_PE=3_SV=2

Length=373

Subject= 78712-284_4_ORF1
>sp|Q8DKR7|DNAJ_THEEB_Chaperone_protein_DnaJ_OS=Thermosynechococcus_
elongatus_(strain_BP-1)_GN=dnaJ_PE=3_SV=2|||2e-88

Length=309


 Score =   260 bits (665),  Expect = 2e-88, Method: Compositional
matrix adjust.
 Identities = 125/232 (54%), Positives = 164/232 (71%), Gaps = 1/232
(0%)

Query  126
FREAVFGGEKEIRINHLETCKTCQGTGAKPGTRPVTCSTCGGVGQVRRSARTPFGSFTQL  185
            + EAV G  K+I ++ LETC TC G+GAKPGT P +CS CGG GQV  SARTP G
F Q+
Sbjct  72
YLEAVLGTTKDIDVSRLETCNTCDGSGAKPGTSPKSCSRCGGQGQVVSSARTPLGEFRQI  131
```

```
Query  186
TTCPTCGGSGVVIEDRCESCGGQGHIQVSKKLKITIPAGVDNGTRLRVSGEGDAGLRGGP  245
            +TCP+C G G      C  C G G  +  K + + +PAGVD+G+RLRV  EG+AG
RGGP
Sbjct  132  STCPSCSGVGET-
STPCTMCRGDGRERKKKSISLKVPAGVDSGSRLRVRSEGNAGKRGGP  190

Query  246
PGDLYVYLFVQPDPEFQREGNNILSRIKISYLQAILGCRISVSTVDGEAELKIPAGTQPG  305
            PGDLYV++ VQ  P   R+GNNIL+  ++SY+ AILG    V TVDG+
+LKIPAGTQPG
Sbjct  191
PGDLYVFISVQAHPTLSRDGNNILTTCRVSYIDAILGTSKKVPTVDGDTDLKIPAGTQPG  250

Query  306  TVLVLEGRGVPRVGNPVARGDHLITVDVEIPTHITHEERELLEKLAKIRGER
357
            T LV+  +GVP +G P  RGD L+ V+VEIP +++ EER+L+++LA ++  R
Sbjct  251  TTLVMSKKGVPVLGKPSIRGDQLVRVEVEIPKYLSGEERKLIDQLADLKKVR
302



Lambda      K        H        a         alpha
   0.318    0.140    0.418    0.792     4.96

Gapped
Lambda      K        H        a         alpha    sigma
   0.267   0.0410    0.140     1.90     42.6     43.6

Effective search space used: 157883




Query= sp|Q8DKR7|
DNAJ_THEEB_Chaperone_protein_DnaJ_OS=Thermosynechococcus_e
longatus_(strain_BP-1)_GN=dnaJ_PE=3_SV=2

Length=373

Subject= 78958-284_4_ORF1
>sp|Q8DKR7|DNAJ_THEEB_Chaperone_protein_DnaJ_OS=Thermosynechococcus_
elongatus_(strain_BP-1)_GN=dnaJ_PE=3_SV=2|||5e-131

Length=494


 Score =   353 bits (907),  Expect = 6e-122, Method: Compositional
matrix adjust.
 Identities = 188/365 (52%), Positives = 246/365 (67%), Gaps =
15/365 (4%)

Query  1    MAR-
```

```
DFYEILGVSRSADAEELKRAYRRLARKYHPDVNKEPGAEEKFKEINRAYEVLSDPQ  59
            MA+ D+Y +LGVS++A   ++K +YR+LAR+ HPDVNKEP AE KFKEI+ AYEVL
D +
Sbjct  130
MAKADYYSVLGVSKNASKADIKSSYRKLARQCHPDVNKEPDAEAKFKEISNAYEVLCDDE  189

Query  60   ARANYDRFGEAGVSGVGAAGFSDFGIGDMGGFADIFETFFGGFTTSSRR-------
QQGP  112
             R  YD++GEAG+ G GA               D+FETFFGG
+  P
Sbjct  190  KRPIYDQYGEAGLKGAGAGASG------
FSNPFDLFETFFGGAGMGGMGGMGGRSARNRP  243

Query  113
TRGEDLRYDLKLEFREAVFGGEKEIRINHLETCKTCQGTGAKPGTRPVTCSTCGGVGQVR  172
             +G D  YDL++EF EAVFG  K+I ++ LETC TC G+GAKPGT P +CS CGG
GQV
Sbjct  244
MQGADEEYDLRIEFLEAVFGTTKDIDVSRLETCNTCDGSGAKPGTSPKSCSRCGGQGQVV  303

Query  173
RSARTPFGSFTQLTTCPTCGGSGVVIEDRCESCGGQGHIQVSKKLKITIPAGVDNGTRLR  232
             SARTP G F Q++TCP+C G G      C  C G G  +  K + + +PAGVD+G
+RLR
Sbjct  304  SSARTPLGEFRQISTCPSCSGVGET-
STPCTMCRGDGRERKKKSISLKVPAGVDSGSRLR  362

Query  233
VSGEGDAGLRGGPPGDLYVYLFVQPDPEFQREGNNILSRIKISYLQAILGCRISVSTVDG  292
            V  EG+AG RGGPPGDLYV++ VQ  P   R+GNNIL+  ++SY+ AILG    V
TVDG
Sbjct  363
VRSEGNAGKRGGPPGDLYVFISVQAHPTLSRDGNNILTTCRVSYIDAILGTSKKVPTVDG  422

Query  293
EAELKIPAGTQPGTVLVLEGRGVPRVGNPVARGDHLITVDVEIPTHITHEERELLEKLAK  352
            + +LKIPAGTQPGT LV+  +GVP +G P  RGD L+ V+VEIP +++ EER+L++
+LA
Sbjct  423
DTDLKIPAGTQPGTTLVMSKKGVPVLGKPSIRGDQLVRVEVEIPKYLSGEERKLIDQLAD  482

Query  353  IRGER  357
            ++  R
Sbjct  483  LKKVR  487


 Score = 15.8 bits (29),  Expect = 2.7, Method: Compositional matrix
adjust.
 Identities = 8/29 (28%), Positives = 13/29 (45%), Gaps = 0/29 (0%)

Query  55   LSDPQARANYDRFGEAGVSGVGAAGFSDF  83
            L+ P  R+N      +G   + +   SDF
Sbjct  75   LASPFPRSNGTHHSSSGKERMVSLSASDF  103
```

```
Lambda      K        H        a         alpha
   0.318    0.140    0.418    0.792     4.96

Gapped
Lambda      K        H        a         alpha    sigma
   0.267   0.0410    0.140     1.90     42.6     43.6

Effective search space used: 157883




Query= sp|Q8DKR7|
DNAJ_THEEB_Chaperone_protein_DnaJ_OS=Thermosynechococcus_e
longatus_(strain_BP-1)_GN=dnaJ_PE=3_SV=2

Length=373

Subject= 99704-250_4_ORF1
>sp|Q8DKR7|DNAJ_THEEB_Chaperone_protein_DnaJ_OS=Thermosynechococcus_
elongatus_(strain_BP-1)_GN=dnaJ_PE=3_SV=2|||1e-137

Length=455


 Score =   370 bits (950),  Expect = 7e-129, Method: Compositional
matrix adjust.
 Identities = 194/361 (54%), Positives = 251/361 (70%), Gaps =
17/361 (5%)

Query  4
DFYEILGVSRSADAEELKRAYRRLARKYHPDVNKEPGAEEKFKEINRAYEVLSDPQARAN  63
            D+Y ILGVS++A   E+K +YR+LAR+YHPDVNKE GAE+KFKEI+ AYEVLSD +
R+
Sbjct  92
DYYSILGVSKNASKPEIKSSYRKLARQYHPDVNKESGAEQKFKEISNAYEVLSDDEKRSI  151

Query  64   YDRFGEAGVSGVGAAGFSDFGIGDMGGFADIFETFFGGFTTSSRR-----------
QQGP  112
            YD+FGEAG+ G GA G + F         D+FETFFGG
+
Sbjct  152  YDKFGEAGLKGAGAGGAAGFS-----
NPFDLFETFFGGMGGMGGMGGMGGMGGRAARNRA  206

Query  113
TRGEDLRYDLKLEFREAVFGGEKEIRINHLETCKTCQGTGAKPGTRPVTCSTCGGVGQVR  172
             +G+D RYDL L F++AVFG +KEI +  LE+C TC G+GAKPGT PVTC+TCGG
GQV
Sbjct  207
VQGDDERYDLLLNFKDAVFGTDKEIEVTRLESCATCNGSGAKPGTSPVTCTTCGGQGQVA  266

Query  173
```

```
RSARTPFGSFTQLTTCPTCGGSGVVIEDRCESCGGQGHIQVSKKLKITIPAGVDNGTRLR  232
             +  TP G F Q++TCP CGG+G      C +C G G ++ +KK+ + +PAGVD+G
+RLR
Sbjct  267  TTTNTPLGQFRQISTCPVCGGTGESFTP-
CNACAGDGRVRRTKKISLKVPAGVDSGSRLR  325

Query  233
VSGEGDAGLRGGPPGDLYVYLFVQPDPEFQREGNNILSRIKISYLQAILGCRISVSTVDG  292
            V  EG+AG RGGPPGDLYV++ V+ D E +R+GNNIL   KI+Y+ AILG  + V
TVDG
Sbjct  326
VRSEGNAGKRGGPPGDLYVFINVRQDSELKRDGNNILITCKITYIDAILGTTVKVPTVDG  385

Query  293
EAELKIPAGTQPGTVLVLEGRGVPRVGNPVARGDHLITVDVEIPTHITHEERELLEKLAK  352
             +LKIPAGTQPGT LV+  RGVP +G    RGD L+ V VEIP  ++ EER+L+E
+LA
Sbjct  386
VVDLKIPAGTQPGTTLVMAKRGVPYLGKTNVRGDQLVKVQVEIPKRLSTEERKLVEELAN  445

Query  353  I  353
            +
Sbjct  446  L  446



Lambda      K        H        a         alpha
   0.318    0.140    0.418    0.792     4.96

Gapped
Lambda      K        H        a         alpha    sigma
   0.267   0.0410    0.140     1.90     42.6     43.6

Effective search space used: 157883



Matrix: BLOSUM62
Gap Penalties: Existence: 11, Extension: 1
Neighboring words threshold: 11
Window for multiple hits: 40



Query= sp|Q8LE52|DHAR3_ARATH_Glutathione_S-transferase_DHAR3,
_chloroplastic_OS=Arabidopsis_thaliana_GN=DHAR3_PE=1_SV=1

Length=258

Subject= 177876-158_1_ORF2
>sp|Q8LE52|DHAR3_ARATH_Glutathione_S-transferase_DHAR3,
```

```
_chloroplastic_OS=Arabidopsis_thaliana_GN=DHAR3_PE=1_SV=1|||2e-106

Length=295


 Score =   302 bits (773),  Expect = 2e-106, Method: Compositional
matrix adjust.
 Identities = 139/219 (63%), Positives = 176/219 (80%), Gaps = 1/219
(0%)

Query  39   FVTMAT-
AASPLEICVKASITTPNKLGDCPFCQKVLLTMEEKNVPYDMKMVDLSNKPEWF  97
             V+MA  +A P+E+ VKA+   P K+GDCPF Q+VLLT+EEK +PY+ K VDL
+NKP+WF
Sbjct  76
IVSMAVVSAEPVEVLVKAATGDPEKIGDCPFSQRVLLTLEEKGIPYNAKYVDLTNKPDWF  135

Query  98
LKISPEGKVPVVKFDEKWVPDSDVITQALEEKYPEPPLATPPEKASVGSKIFSTFVGFLK  157
            L+I+PEGKVPV+K + KWVPDSDVITQ LEEK+PE  L TPPEKAS GSKIF +FV
FLK
Sbjct  136
LEINPEGKVPVIKHEGKWVPDSDVITQILEEKFPETVLQTPPEKASAGSKIFPSFVKFLK  195

Query  158
SKDSGDGTEQVLLDELTTFNDYIKDNGPFINGEKISAADLSLAPKLYHMKIALGHYKNWS  217
            SKDS DG+E  L+ ELT FNDY+KDNGPFING+KIS+ADLSL PKL+H++
+ALGHYK WS
Sbjct  196
SKDSSDGSEDALVAELTAFNDYLKDNGPFINGDKISSADLSLGPKLFHLQVALGHYKKWS  255

Query  218  VPDSLPFVKSYMENVFSRESFTNTRAETEDVIAGWRPKV  256
            +P +L +V  Y++ V +RESF  T+   E V+AGW+  +
Sbjct  256  IPSNLTYVDKYIKAVHARESFVKTKPAEEHVVAGWQKHI  294



Lambda      K        H        a         alpha
   0.316    0.133    0.397    0.792     4.96

Gapped
Lambda      K        H        a         alpha    sigma
   0.267   0.0410    0.140     1.90     42.6     43.6

Effective search space used: 62910




Query= sp|Q8LE52|DHAR3_ARATH_Glutathione_S-transferase_DHAR3,
_chloroplastic_OS=Arabidopsis_thaliana_GN=DHAR3_PE=1_SV=1

Length=258
```

```
Subject= 178555-158_1_ORF2
>sp|Q8LE52|DHAR3_ARATH_Glutathione_S-transferase_DHAR3,
_chloroplastic_OS=Arabidopsis_thaliana_GN=DHAR3_PE=1_SV=1|||6e-107

Length=299


 Score =   303 bits (777),  Expect = 6e-107, Method: Compositional
matrix adjust.
 Identities = 140/224 (63%), Positives = 177/224 (79%), Gaps = 1/224
(0%)

Query  34   GRVGRFVTMAT-
AASPLEICVKASITTPNKLGDCPFCQKVLLTMEEKNVPYDMKMVDLSN  92
            G     V+MA  +A P+E+ VKA+   P K+GDCPF Q+VLLT+EEK +PY+ K
VDL+N
Sbjct  75
GLASSIVSMAVVSAEPVEVLVKAATGDPEKIGDCPFSQRVLLTLEEKGIPYNAKYVDLTN  134

Query  93
KPEWFLKISPEGKVPVVKFDEKWVPDSDVITQALEEKYPEPPLATPPEKASVGSKIFSTF  152
            KP+WFL+I+PEGKVPV+K + KWVPDSDVITQ LEEK+PE  L TPPEKAS
GSKIF +F
Sbjct  135
KPDWFLEINPEGKVPVIKHEGKWVPDSDVITQILEEKFPETVLQTPPEKASAGSKIFPSF  194

Query  153
VGFLKSKDSGDGTEQVLLDELTTFNDYIKDNGPFINGEKISAADLSLAPKLYHMKIALGH  212
            V FLKSKDS DG+E  L+ ELT FNDY+KDNGPFING+KIS+ADLSL PKL+H++
+ALGH
Sbjct  195
VKFLKSKDSSDGSEDALVAELTAFNDYLKDNGPFINGDKISSADLSLGPKLFHLQVALGH  254

Query  213  YKNWSVPDSLPFVKSYMENVFSRESFTNTRAETEDVIAGWRPKV  256
            YK WS+P +L +V  Y++ V +RESF  T+   E V+AGW+  +
Sbjct  255  YKKWSIPSNLTYVDKYIKAVHARESFVKTKPAEEHVVAGWQKHI  298



Lambda      K        H        a         alpha
   0.316    0.133    0.397    0.792     4.96

Gapped
Lambda      K        H        a         alpha    sigma
   0.267   0.0410    0.140     1.90     42.6     43.6

Effective search space used: 62910



Matrix: BLOSUM62
Gap Penalties: Existence: 11, Extension: 1
Neighboring words threshold: 11
```

Window for multiple hits: 40

Query= sp|Q8TL44|
TRPB2_METAC_Tryptophan_synthase_beta_chain_2_OS=Methanosar
cina_acetivorans_(strain_ATCC_35395_/_DSM_2834_/_JCM_12185_/_C2A)_GN
=trpB2_PE=3_SV=1

Length=442

Subject= 158601-182_4_ORF1
>sp|Q8TL44|TRPB2_METAC_Tryptophan_synthase_beta_chain_2_OS=Methanosa
rcina_acetivorans_(strain_ATCC_35395_/_DSM_2834_/_JCM_12185_/_C2A)_G
N=trpB2_PE=3_SV=1|||4e-170

Length=464

Score = 479 bits (1234), Expect = 2e-170, Method: Compositional
matrix adjust.
Identities = 230/371 (62%), Positives = 293/371 (79%), Gaps = 0/371
(0%)

```
Query  15
KIILDENEMPKKWYNVLADLPSPIDPPLDPRTWQPISPDALEPIFPKALIMQEMSSDRYI  74
            KI L +N+MP++WYN++ADLP P   PL P+T QP+ P+ L P+FP+ALI QE S
+ YI
Sbjct  94
KITLPDNDMPQQWYNIIADLPKPPPSPLHPKTLQPLKPEDLMPLFPQALIEQEGSLEAYI  153

Query  75
DIPEEVLDVYRLWRPSPLFRAHQLEKVLKSPAKIYYKYEGVSPAGSHKTNTSIAQAYYNM  134
            +IPEEVLD+Y +WRP+PLFRA +LE  LK+PA+IYYKYEG SPAGSHK NT++ QA
+YN
Sbjct  154
NIPEEVLDIYSMWRPTPLFRARRLEAFLKTPARIYYKYEGGSPAGSHKPNTAVPQAWYNA  213

Query  135
KEGTERLTTETGAGQWGSALSLACNYFDLECKVYMVRSSFYQKPYRKSLITLWGGNVVPS  194
            + G +RLTTETGAGQWGSAL+ AC++F + C+V+ VR+S+ QKPYRK ++  W
V PS
Sbjct  214
QAGVKRLTTETGAGQWGSALAFACSHFGIGCEVWQVRASYDQKPYRKIMMETWAAKVHPS  273

Query  195
PSPDTEFGRKILQEQPDTPGSLGIAISEAVEDAIAHENTKYSLGSVLNHVVLHQTVIGAE  254
            PS  T  GR IL++ P +PGSLGIA+SEAVE A+A +NTKYSLGS+LNHV+LHQT
+IG E
Sbjct  274
PSTLTAAGRSILEKDPSSPGSLGIAVSEAVEAALADDNTKYSLGSILNHVLLHQTIIGEE  333
```

```
Query  255
CKQQLAQVEEYPDVVIGCCGGGSNLGGIGLEFIKDRLEGKHSARVVAVEPSACPSLTKGE  314
            C +QLA VEE PDV+IGC GGGSN  GI   FI+++++GK +  + AVEP
+ACPSLTKG
Sbjct  334
CVKQLAMVEETPDVIIGCTGGGSNFSGIAFPFIREKMKGKMNPIIRAVEPAACPSLTKGV  393

Query  315
YRYDFGDTAEMTPLLKMYTLGHKHVPPAIHAGGLRYHGDSPIISKLCSEGLMEAVSYDQQ  374
            Y YDFGD A +TPLLKM+TLGH  VP  IHAGGLRYHG +P++S L + G MEA++
Q
Sbjct  394
YAYDFGDAAGLTPLLKMHTLGHDFVPDPIHAGGLRYHGMAPLVSHLYNLGFMEAIAIPQI  453

Query  375  EVFDAAVQFAR  385
            E F+AA+ FAR
Sbjct  454  ECFEAALNFAR  464


 Score = 17.3 bits (33),  Expect = 1.1, Method: Compositional matrix
adjust.
 Identities = 7/13 (54%), Positives = 7/13 (54%), Gaps = 0/13 (0%)

Query  201  FGRKILQEQPDTP  213
            FGRKI     D P
Sbjct  91   FGRKITLPDNDMP  103


 Score = 16.2 bits (30),  Expect = 2.4, Method: Compositional matrix
adjust.
 Identities = 7/25 (28%), Positives = 12/25 (48%), Gaps = 0/25 (0%)

Query  392  APESSHAIRCAIDEALAAKQTGEEK  416
            +P  SH    A+ +A    Q G ++
Sbjct  195  SPAGSHKPNTAVPQAWYNAQAGVKR  219



Lambda      K        H        a         alpha
   0.316    0.134    0.403    0.792     4.96

Gapped
Lambda      K        H        a         alpha    sigma
   0.267   0.0410    0.140     1.90     42.6     43.6

Effective search space used: 176279




Query= sp|Q8TL44|
TRPB2_METAC_Tryptophan_synthase_beta_chain_2_OS=Methanosar
cina_acetivorans_(strain_ATCC_35395_/_DSM_2834_/_JCM_12185_/_C2A)_GN
=trpB2_PE=3_SV=1
```

Length=442

Subject= 158734-182_4_ORF1
>sp|Q8TL44|TRPB2_METAC_Tryptophan_synthase_beta_chain_2_OS=Methanosa
rcina_acetivorans_(strain_ATCC_35395_/_DSM_2834_/_JCM_12185_/_C2A)_G
N=trpB2_PE=3_SV=1|||2e-170

Length=461

Score = 480 bits (1236), Expect = 7e-171, Method: Compositional
matrix adjust.
Identities = 230/371 (62%), Positives = 293/371 (79%), Gaps = 0/371
(0%)

```
Query  15
KIILDENEMPKKWYNVLADLPSPIDPPLDPRTWQPISPDALEPIFPKALIMQEMSSDRYI  74
            KI L +N+MP++WYN++ADLP P   PL P+T QP+ P+ L P+FP+ALI QE S
+ YI
Sbjct  91
KITLPDNDMPQQWYNIIADLPKPPPSPLHPKTLQPLKPEDLMPLFPQALIEQEGSLEAYI  150

Query  75
DIPEEVLDVYRLWRPSPLFRAHQLEKVLKSPAKIYYKYEGVSPAGSHKTNTSIAQAYYNM  134
            +IPEEVLD+Y +WRP+PLFRA +LE  LK+PA+IYYKYEG SPAGSHK NT++ QA
+YN
Sbjct  151
NIPEEVLDIYSMWRPTPLFRARRLEAFLKTPARIYYKYEGGSPAGSHKPNTAVPQAWYNA  210

Query  135
KEGTERLTTETGAGQWGSALSLACNYFDLECKVYMVRSSFYQKPYRKSLITLWGGNVVPS  194
            + G +RLTTETGAGQWGSAL+ AC++F + C+V+ VR+S+ QKPYRK ++  W
V PS
Sbjct  211
QAGVKRLTTETGAGQWGSALAFACSHFGIGCEVWQVRASYDQKPYRKIMMETWAAKVHPS  270

Query  195
PSPDTEFGRKILQEQPDTPGSLGIAISEAVEDAIAHENTKYSLGSVLNHVVLHQTVIGAE  254
            PS  T  GR IL++ P +PGSLGIA+SEAVE A+A +NTKYSLGS+LNHV+LHQT
+IG E
Sbjct  271
PSTLTAAGRSILEKDPSSPGSLGIAVSEAVEAALADDNTKYSLGSILNHVLLHQTIIGEE  330

Query  255
CKQQLAQVEEYPDVVIGCCGGGSNLGGIGLEFIKDRLEGKHSARVVAVEPSACPSLTKGE  314
            C +QLA VEE PDV+IGC GGGSN  GI   FI+++++GK +  + AVEP
+ACPSLTKG
Sbjct  331
CVKQLAMVEETPDVIIGCTGGGSNFSGIAFPFIREKMKGKMNPIIRAVEPAACPSLTKGV  390

Query  315
YRYDFGDTAEMTPLLKMYTLGHKHVPPAIHAGGLRYHGDSPIISKLCSEGLMEAVSYDQQ  374
            Y YDFGD A +TPLLKM+TLGH  VP  IHAGGLRYHG +P++S L + G MEA++
```

```
Q
Sbjct  391
YAYDFGDAAGLTPLLKMHTLGHDFVPDPIHAGGLRYHGMAPLVSHLYNLGFMEAIAIPQI  450

Query  375  EVFDAAVQFAR  385
            E F+AA+ FAR
Sbjct  451  ECFEAALNFAR  461


 Score = 17.3 bits (33),  Expect = 1.1, Method: Compositional matrix
adjust.
 Identities = 7/13 (54%), Positives = 7/13 (54%), Gaps = 0/13 (0%)

Query  201  FGRKILQEQPDTP  213
            FGRKI     D P
Sbjct  88   FGRKITLPDNDMP  100


 Score = 16.2 bits (30),  Expect = 2.5, Method: Compositional matrix
adjust.
 Identities = 7/25 (28%), Positives = 12/25 (48%), Gaps = 0/25 (0%)

Query  392  APESSHAIRCAIDEALAAKQTGEEK  416
            +P  SH    A+ +A    Q G ++
Sbjct  192  SPAGSHKPNTAVPQAWYNAQAGVKR  216



Lambda      K        H        a         alpha
   0.316    0.134    0.403    0.792     4.96

Gapped
Lambda      K        H        a         alpha    sigma
   0.267   0.0410    0.140     1.90     42.6     43.6

Effective search space used: 176279



Matrix: BLOSUM62
Gap Penalties: Existence: 11, Extension: 1
Neighboring words threshold: 11
Window for multiple hits: 40



Query= sp|Q8VYZ3|
PME53_ARATH_Probable_pectinesterase_53_OS=Arabidopsis_thal
iana_GN=PME53_PE=2_SV=1

Length=383
```

Subject= 18361-579_1_ORF2
>sp|Q8VYZ3|PME53_ARATH_Probable_pectinesterase_53_OS=Arabidopsis_thaliana_GN=PME53_PE=2_SV=1|||3e-119

Length=388

```
 Score =   332 bits (852),  Expect = 4e-115, Method: Compositional
matrix adjust.
 Identities = 171/342 (50%), Positives = 229/342 (67%), Gaps =
18/342 (5%)

Query  51   QNPEDEFMKWVRFVG---------
SLKHSVFKAAKNKLFPSYTLTVHKKSNKGDFTKIQD  101
            +    +F+ WV+ VG         S++ +      NK   S  LTV     +  FT
+Q
Sbjct  54   EEKRQDFLGWVQSVGERVAASSTLSVEDNTAWLQANKAGGS-VLTVGNSGAQ--
FTSVQA  110

Query  102  AIDSLPLINFVRVVIKVHAGVYKEKVSIPPLKAFITIEGEGAEKTTVEWGDT-
AQTPDSK  160
            A+DS+P  N  RVVI++ AG Y EKV +P  K +I+  G G+  T + +GDT + T
K
Sbjct  111
AVDSVPKHNSQRVVIQIAAGTYHEKVFVPKNKPYISFLGAGSGVTKITYGDTKSDTASGK  170

Query  161
GNPMGTYNSASFAVNSPFFVAKNITFRNTTPVPLPGAVGKQAVALRVSADNAAFFGCRML  220
                 T +SA+  VNS  F+A+ ITF N+ P P PGAVGKQAVA +++ D AA +
C  L
Sbjct  171  -----
TQDSATVGVNSDGFIARGITFENSAPPPPPGAVGKQAVAFKIAGDKAALYACSFL  225

Query  221
GAQDTLYDHLGRHYYKDCYIEGSVDFIFGNALSLYEGCHVHAIADKLGAVTAQGRSSVLE  280
            GAQDTLYD  GRHY+++CYI+GS+DFIFG+  SLY+GC +H ++   G++TAQ
RSS
Sbjct  226
GAQDTLYDDHGRHYFENCYIQGSIDFIFGDGQSLYKGCQLHTLSINPGSLTAQKRSSPSS  285

Query  281
DTGFSFVKCKVTGTGVLYLGRAWGPFSRVVFAYTYMDNIILPRGWYNWGDPSREMTVFYG  340
             TGFSFV C V G+GV+YLGRAWG +SRVVF+YTY+ N+I+P+GW+NWG   R+
TVFYG
Sbjct  286
STGFSFVDCTVGGSGVVYLGRAWGVYSRVVFSYTYLGNVIIPQGWFNWGVSGRDQTVFYG  345

Query  341  QYKCTGAGANYGGRVAWARELTDEEAKPFLSLTFIDGSEWIK  382
            +YKC+G G+N  GRVAWA +LTD +A PF SL FIDG+ WI+
Sbjct  346  EYKCSGPGSNQSGRVAWAHQLTDAQAAPFQSLNFIDGASWIQ  387
```

```
Lambda      K        H        a         alpha
   0.320    0.136    0.422    0.792     4.96

Gapped
Lambda      K        H        a         alpha    sigma
   0.267   0.0410    0.140     1.90     42.6     43.6

Effective search space used: 126374
```

```
Query= sp|Q8VYZ3|
PME53_ARATH_Probable_pectinesterase_53_OS=Arabidopsis_thal
iana_GN=PME53_PE=2_SV=1

Length=383

Subject= 18363-579_1_ORF2
>sp|Q8VYZ3|PME53_ARATH_Probable_pectinesterase_53_OS=Arabidopsis_tha
liana_GN=PME53_PE=2_SV=1|||2e-119

Length=394


 Score =   332 bits (852),  Expect = 6e-115, Method: Compositional
matrix adjust.
 Identities = 171/342 (50%), Positives = 229/342 (67%), Gaps =
18/342 (5%)

Query  51   QNPEDEFMKWVRFVG---------
SLKHSVFKAAKNKLFPSYTLTVHKKSNKGDFTKIQD  101
            +    +F+ WV+ VG         S++ +      NK   S  LTV     +  FT
+Q
Sbjct  60   EEKRQDFLGWVQSVGERVAASSTLSVEDNTAWLQANKAGGS-VLTVGNSGAQ--
FTSVQA  116

Query  102  AIDSLPLINFVRVVIKVHAGVYKEKVSIPPLKAFITIEGEGAEKTTVEWGDT-
AQTPDSK  160
            A+DS+P  N  RVVI++ AG Y EKV +P  K +I+  G G+  T + +GDT + T
K
Sbjct  117
AVDSVPKHNSQRVVIQIAAGTYHEKVFVPKNKPYISFLGAGSGVTKITYGDTKSDTASGK  176

Query  161
GNPMGTYNSASFAVNSPFFVAKNITFRNTTPVPLPGAVGKQAVALRVSADNAAFFGCRML  220
                 T +SA+  VNS  F+A+ ITF N+ P P PGAVGKQAVA +++ D AA +
C  L
Sbjct  177  -----
TQDSATVGVNSDGFIARGITFENSAPPPPPGAVGKQAVAFKIAGDKAALYACSFL  231

Query  221
GAQDTLYDHLGRHYYKDCYIEGSVDFIFGNALSLYEGCHVHAIADKLGAVTAQGRSSVLE  280
            GAQDTLYD  GRHY+++CYI+GS+DFIFG+  SLY+GC +H ++   G++TAQ
```

RSS
Sbjct 232
GAQDTLYDDHGRHYFENCYIQGSIDFIFGDGQSLYKGCQLHTLSINPGSLTAQKRSSPSS 291

Query 281
DTGFSFVKCKVTGTGVLYLGRAWGPFSRVVFAYTYMDNIILPRGWYNWGDPSREMTVFYG 340
TGFSFV C V G+GV+YLGRAWG +SRVVF+YTY+ N+I+P+GW+NWG R+
TVFYG
Sbjct 292
STGFSFVDCTVGGSGVVYLGRAWGVYSRVVFSYTYLGNVIIPQGWFNWGVSGRDQTVFYG 351

Query 341 QYKCTGAGANYGGRVAWARELTDEEAKPFLSLTFIDGSEWIK 382
+YKC+G G+N GRVAWA +LTD +A PF SL FIDG+ WI+
Sbjct 352 EYKCSGPGSNQSGRVAWAHQLTDAQAAPFQSLNFIDGASWIQ 393

Lambda K H a alpha
0.320 0.136 0.422 0.792 4.96

Gapped
Lambda K H a alpha sigma
0.267 0.0410 0.140 1.90 42.6 43.6

Effective search space used: 126374

Query= sp|Q8VYZ3|
PME53_ARATH_Probable_pectinesterase_53_OS=Arabidopsis_thal
iana_GN=PME53_PE=2_SV=1

Length=383

Subject= 350703-43_5_ORF1
>sp|Q8VYZ3|PME53_ARATH_Probable_pectinesterase_53_OS=Arabidopsis_tha
liana_GN=PME53_PE=2_SV=1|||7e-120

Length=349

Score = 332 bits (852), Expect = 1e-115, Method: Compositional
matrix adjust.
Identities = 171/342 (50%), Positives = 229/342 (67%), Gaps =
18/342 (5%)

Query 51 QNPEDEFMKWVRFVG---------
SLKHSVFKAAKNKLFPSYTLTVHKKSNKGDFTKIQD 101
+ +F+ WV+ VG S++ + NK S LTV + FT
+Q
Sbjct 15 EEKRQDFLGWVQSVGERVAASSTLSVEDNTAWLQANKAGGS-VLTVGNSGAQ--
FTSVQA 71

```
Query  102  AIDSLPLINFVRVVIKVHAGVYKEKVSIPPLKAFITIEGEGAEKTTVEWGDT-
AQTPDSK  160
            A+DS+P  N  RVVI++ AG Y EKV +P  K +I+  G G+  T + +GDT + T
K
Sbjct  72
AVDSVPKHNSQRVVIQIAAGTYHEKVFVPKNKPYISFLGAGSGVTKITYGDTKSDTASGK  131

Query  161
GNPMGTYNSASFAVNSPFFVAKNITFRNTTPVPLPGAVGKQAVALRVSADNAAFFGCRML  220
                 T +SA+  VNS  F+A+ ITF N+ P P PGAVGKQAVA +++ D AA +
C  L
Sbjct  132  -----
TQDSATVGVNSDGFIARGITFENSAPPPPPGAVGKQAVAFKIAGDKAALYACSFL  186

Query  221
GAQDTLYDHLGRHYYKDCYIEGSVDFIFGNALSLYEGCHVHAIADKLGAVTAQGRSSVLE  280
            GAQDTLYD  GRHY+++CYI+GS+DFIFG+  SLY+GC +H ++   G++TAQ
RSS
Sbjct  187
GAQDTLYDDHGRHYFENCYIQGSIDFIFGDGQSLYKGCQLHTLSINPGSLTAQKRSSPSS  246

Query  281
DTGFSFVKCKVTGTGVLYLGRAWGPFSRVVFAYTYMDNIILPRGWYNWGDPSREMTVFYG  340
             TGFSFV C V G+GV+YLGRAWG +SRVVF+YTY+ N+I+P+GW+NWG   R+
TVFYG
Sbjct  247
STGFSFVDCTVGGSGVVYLGRAWGVYSRVVFSYTYLGNVIIPQGWFNWGVSGRDQTVFYG  306

Query  341  QYKCTGAGANYGGRVAWARELTDEEAKPFLSLTFIDGSEWIK  382
            +YKC+G G+N  GRVAWA +LTD +A PF SL FIDG+ WI+
Sbjct  307  EYKCSGPGSNQSGRVAWAHQLTDAQAAPFQSLNFIDGASWIQ  348



Lambda      K        H        a         alpha
   0.320    0.136    0.422    0.792     4.96

Gapped
Lambda      K        H        a         alpha    sigma
   0.267   0.0410    0.140     1.90     42.6     43.6

Effective search space used: 126374




Query= sp|Q8VYZ3|
PME53_ARATH_Probable_pectinesterase_53_OS=Arabidopsis_thal
iana_GN=PME53_PE=2_SV=1

Length=383

Subject= 372240-34_6_ORF2
>sp|Q8VYZ3|PME53_ARATH_Probable_pectinesterase_53_OS=Arabidopsis_tha
```

```
liana_GN=PME53_PE=2_SV=1|||8e-93

Length=346


 Score =   263 bits (671),  Expect = 2e-88, Method: Compositional
matrix adjust.
 Identities = 140/295 (47%), Positives = 191/295 (65%), Gaps =
18/295 (6%)

Query  51   QNPEDEFMKWVRFVG---------
SLKHSVFKAAKNKLFPSYTLTVHKKSNKGDFTKIQD  101
            +    +F+ WV+ VG         S++ +      NK   S  LTV     +  FT
+Q
Sbjct  60   EEKRQDFLGWVQSVGERVAASSTLSVEDNTAWLQANKAGGS-VLTVGNSGAQ--
FTSVQA  116

Query  102  AIDSLPLINFVRVVIKVHAGVYKEKVSIPPLKAFITIEGEGAEKTTVEWGDT-
AQTPDSK  160
            A+DS+P  N  RVVI++ AG Y EKV +P  K +I+  G G+  T + +GDT + T
K
Sbjct  117
AVDSVPKHNSQRVVIQIAAGTYHEKVFVPKNKPYISFLGAGSGVTKITYGDTKSDTASGK  176

Query  161
GNPMGTYNSASFAVNSPFFVAKNITFRNTTPVPLPGAVGKQAVALRVSADNAAFFGCRML  220
                 T +SA+  VNS  F+A+ ITF N+ P P PGAVGKQAVA +++ D AA +
C  L
Sbjct  177  -----
TQDSATVGVNSDGFIARGITFENSAPPPPPGAVGKQAVAFKIAGDKAALYACSFL  231

Query  221
GAQDTLYDHLGRHYYKDCYIEGSVDFIFGNALSLYEGCHVHAIADKLGAVTAQGRSSVLE  280
            GAQDTLYD  GRHY+++CYI+GS+DFIFG+  SLY+GC +H ++   G++TAQ
RSS
Sbjct  232
GAQDTLYDDHGRHYFENCYIQGSIDFIFGDGQSLYKGCQLHTLSINPGSLTAQKRSSPSS  291

Query  281  DTGFSFVKCKVTGTGVLYLGRAWGPFSRVVFAYTYMDNIILPRGWYNWGDPSREM
335
             TGFSFV C V G+GV+YLGRAWG +SRVVF+YTY+ N+I+P+GW+NWG   R+
Sbjct  292  STGFSFVDCTVGGSGVVYLGRAWGVYSRVVFSYTYLGNVIIPQGWFNWGVSGRDQ
346



Lambda      K        H        a         alpha
   0.320    0.136    0.422    0.792     4.96

Gapped
Lambda      K        H        a         alpha    sigma
   0.267   0.0410    0.140     1.90     42.6     43.6

Effective search space used: 126374
```

```
Query= sp|Q8VYZ3|
PME53_ARATH_Probable_pectinesterase_53_OS=Arabidopsis_thal
iana_GN=PME53_PE=2_SV=1

Length=383

Subject= 372580-34_6_ORF2
>sp|Q8VYZ3|PME53_ARATH_Probable_pectinesterase_53_OS=Arabidopsis_tha
liana_GN=PME53_PE=2_SV=1|||2e-81

Length=218


 Score =   229 bits (584),  Expect = 2e-77, Method: Compositional
matrix adjust.
 Identities = 113/215 (53%), Positives = 152/215 (71%), Gaps = 6/215
(3%)

Query  122  VYKEKVSIPPLKAFITIEGEGAEKTTVEWGDT-
AQTPDSKGNPMGTYNSASFAVNSPFFV  180
            +  EKV +P  K +I+  G G+  T + +GDT + T   K      T +SA+  VNS
F+
Sbjct  9    IQSEKVFVPKNKPYISFLGAGSGVTKITYGDTKSDTASGK-----
TQDSATVGVNSDGFI  63

Query  181
AKNITFRNTTPVPLPGAVGKQAVALRVSADNAAFFGCRMLGAQDTLYDHLGRHYYKDCYI  240
            A+ ITF N+ P P PGAVGKQAVA +++ D AA + C  LGAQDTLYD  GRHY++
+CYI
Sbjct  64
ARGITFENSAPPPPPGAVGKQAVAFKIAGDKAALYACSFLGAQDTLYDDHGRHYFENCYI  123

Query  241
EGSVDFIFGNALSLYEGCHVHAIADKLGAVTAQGRSSVLEDTGFSFVKCKVTGTGVLYLG  300
            +GS+DFIFG+  SLY+GC +H ++   G++TAQ RSS    TGFSFV C V G+GV
+YLG
Sbjct  124
QGSIDFIFGDGQSLYKGCQLHTLSINPGSLTAQKRSSPSSSTGFSFVDCTVGGSGVVYLG  183

Query  301  RAWGPFSRVVFAYTYMDNIILPRGWYNWGDPSREM  335
            RAWG +SRVVF+YTY+ N+I+P+GW+NWG   R+
Sbjct  184  RAWGVYSRVVFSYTYLGNVIIPQGWFNWGVSGRDQ  218



Lambda      K        H        a         alpha
   0.320    0.136    0.422    0.792     4.96

Gapped
Lambda      K        H        a         alpha    sigma
```

```
   0.267   0.0410    0.140     1.90     42.6     43.6

Effective search space used: 126374




Query= sp|Q8VYZ3|
PME53_ARATH_Probable_pectinesterase_53_OS=Arabidopsis_thal
iana_GN=PME53_PE=2_SV=1

Length=383

Subject= 372674-34_4_ORF1
>sp|Q8VYZ3|PME53_ARATH_Probable_pectinesterase_53_OS=Arabidopsis_tha
liana_GN=PME53_PE=2_SV=1|||5e-93

Length=301


 Score =   263 bits (671),  Expect = 4e-89, Method: Compositional
matrix adjust.
 Identities = 140/295 (47%), Positives = 191/295 (65%), Gaps =
18/295 (6%)

Query  51   QNPEDEFMKWVRFVG---------
SLKHSVFKAAKNKLFPSYTLTVHKKSNKGDFTKIQD  101
            +    +F+ WV+ VG         S++ +      NK   S  LTV     +  FT
+Q
Sbjct  15   EEKRQDFLGWVQSVGERVAASSTLSVEDNTAWLQANKAGGS-VLTVGNSGAQ--
FTSVQA  71

Query  102  AIDSLPLINFVRVVIKVHAGVYKEKVSIPPLKAFITIEGEGAEKTTVEWGDT-
AQTPDSK  160
            A+DS+P  N  RVVI++ AG Y EKV +P  K +I+  G G+  T + +GDT + T
K
Sbjct  72
AVDSVPKHNSQRVVIQIAAGTYHEKVFVPKNKPYISFLGAGSGVTKITYGDTKSDTASGK  131

Query  161
GNPMGTYNSASFAVNSPFFVAKNITFRNTTPVPLPGAVGKQAVALRVSADNAAFFGCRML  220
                 T +SA+  VNS  F+A+ ITF N+ P P PGAVGKQAVA +++ D AA +
C  L
Sbjct  132  -----
TQDSATVGVNSDGFIARGITFENSAPPPPPGAVGKQAVAFKIAGDKAALYACSFL  186

Query  221
GAQDTLYDHLGRHYYKDCYIEGSVDFIFGNALSLYEGCHVHAIADKLGAVTAQGRSSVLE  280
            GAQDTLYD  GRHY+++CYI+GS+DFIFG+  SLY+GC +H ++   G++TAQ
RSS
Sbjct  187
GAQDTLYDDHGRHYFENCYIQGSIDFIFGDGQSLYKGCQLHTLSINPGSLTAQKRSSPSS  246

Query  281  DTGFSFVKCKVTGTGVLYLGRAWGPFSRVVFAYTYMDNIILPRGWYNWGDPSREM
```

```
335
             TGFSFV C V G+GV+YLGRAWG +SRVVF+YTY+ N+I+P+GW+NWG   R+
Sbjct  247  STGFSFVDCTVGGSGVVYLGRAWGVYSRVVFSYTYLGNVIIPQGWFNWGVSGRDQ
301



Lambda      K        H        a         alpha
   0.320    0.136    0.422    0.792     4.96

Gapped
Lambda      K        H        a         alpha    sigma
   0.267   0.0410    0.140     1.90     42.6     43.6

Effective search space used: 126374




Matrix: BLOSUM62
Gap Penalties: Existence: 11, Extension: 1
Neighboring words threshold: 11
Window for multiple hits: 40





Query= sp|Q93Z16|RPN2_ARATH_Dolichyl-diphosphooligosaccharide--
protein_glycosyltransferase_subunit_2_OS=Arabidopsis_thaliana_GN=RPN
2_PE=2_SV=1

Length=691

Subject= 29863-463_1_ORF2
>sp|Q93Z16|RPN2_ARATH_Dolichyl-diphosphooligosaccharide--
protein_glycosyltransferase_subunit_2_OS=Arabidopsis_thaliana_GN=RPN
2_PE=2_SV=1|||4e-180

Length=695


 Score =   506 bits (1304),  Expect = 6e-174, Method: Compositional
matrix adjust.
 Identities = 307/692 (44%), Positives = 422/692 (61%), Gaps =
15/692 (2%)

Query  5    GNVRFLVLILAV-
AICGAASVFQPISDSHRSAALDVFVPVDGSYKSLEEAYEALKTLEIL  63
            GN R L L+LA+     AA+    +S++ + AA  + +  +GS  SLEE Y+ L++
E+L
Sbjct  14
GNGRLLFLVLAMTCFLSAAASPHALSENDKKAAAQLLIDTEGSISSLEEKYQLLRSFEVL  73
```

Query 64
GIDKKSDLSSKTCENVVKVLQSSSSTLKDAFYALNVNGILKCKIGEAGPKDIVSQLQAGV 123
GI ++ + TC+ V K+L SSSS+ K+ FYA + L C E K S L
+ +
Sbjct 74 GIRREELI---
TCDEVGKILASSSSSPKEIFYASKIGEALLCSFSETDLKVTASNLLSYI 130

Query 124
KDAKLLLDFYYSVRGLVLAKEQFPGTHISLGDAEAIFRSIKSLSQSDGRWRYSSNNPESS 183
+ LLD +S+ L + K + + L +A A+F IK+L + DG W+Y++N+
+S
Sbjct 131 SSSDALLDLRFSIGALAVVK-
NWIKDDLVLENAVAVFHKIKALGEEDGTWKYAANDDGTS 189

Query 184
TFAAGLAYETLAGVISLAPSEFDPSLIQSVKTGILKLSDSIQKYDDGTFYFDEKSVDA-- 241
AAG+A ETLA +I++A D + I+ ++ KL D ++KY+DGT YF
EKSVDA
Sbjct 190
PKAAGIALETLAYLITVASPAIDDAKIRYIRGAASKLFDYVEKYEDGTHYFVEKSVDALY 249

Query 242 -
SQGPISTTASVIRGLTSFAASESTGLNLPGDKIVGLAKFFLGVGIPGDAKDFFNQIDAL 300
GP++ T+ V +G+T+ A++ S L + +KIV +A FFL G+ G D F+
+DAL
Sbjct 250
GGGGPLAATSCVAKGVTAIASATSGQLEISAEKIVSVANFFLIAGVQGSHTDLFHVLDAL 309

Query 301
ACLEDNKFSVPLILSLPSTVISLTKKEPLKVKVSTVLGSKAPALSVKLTQALSSKSVDSS 360
L+ N F VPL+LS+ S+V+SLT K+ LKV +TVLGS A +V L +A+ +
S
Sbjct 310 GALDKNSFLVPLVLSVHSSVLSLTAKDTLKVLTTTVLGSPVAA-
TVTLRKAVRATE-KSP 367

Query 361
VINNQELKFDADSATYFLDSFPKNFDIGKYTFVFKIVLDESAHEKVYITEAQTKVPIAAT 420
+NNQEL+ D + A + D K DIG Y FK+ + + Y T
I T
Sbjct 368 FLNNQELESDDNKAIHRFDLVHKKVDIGTYDLTFKV---
KPLEDGKYAANTFTTARILVT 424

Query 421
GAISIENAEIAVLDSDIGSVESQKKLDLTKDGAVSLSANHLQKLRLSFQLTTPLGNAFKP 480
G SI E+ VLDSD GS + K+ D + VSL A HLQKLR S + +P
FKP
Sbjct 425
GVASIAEVEVGVLDSDTGSTDFMKRWDPSTKEVVSLFATHLQKLRFSLDVFSPFQEPFKP 484

Query 481
HQAFFKLKHESQVEHIFLVKTSGKKSELVLDFLGLVEKLYYLSGKYEIQLTIGDASMENS 540
HQ F KLKHES+VEH+FL+K SGKK EL LD LGLVEKL YLSG Y I+L IGDA
+MEN
Sbjct 485

```
HQVFVKLKHESEVEHLFLLKPSGKKLELSLDLLGLVEKLNYLSGVYAIELIIGDAAMENP  544

Query  541
LLSNIGHIELDLPERPEKATRPPLQSTEPYSRYGPKAEISHIFRIPEKLPAKQLSLVFLG  600
             + ++G +ELDLPE PE  T+       P S+YGPK EI+HIFR+ +K     LS
FL
Sbjct  545  AMWSLGTVELDLPEAPEGVTKSSSTRQAP-
SKYGPKPEITHIFRLADKRAPPTLSTSFLI  603

Query  601
VIVLPFIGFLIGLTRLGVNIKSFPSSTGSAISALLFHCGIGAVLLLYVLFWLKLDLFTTL  660
            + ++P +GFLIGL  L  NIKSFPSS    ++AL+FH GI ++L LY LFWLK
+LF TL
Sbjct  604
LSLVPLLGFLIGLKLLNTNIKSFPSSGLPMLAALVFHVGIASILGLYFLFWLKFNLFQTL  663

Query  661  KALSLLGVFLLFVGHRTLSQLA-SASNKLKSA  691
            K L +L   L   G+  LS LA S S K+KSA
Sbjct  664  KLLGVLATLLTVPGYYLLSHLADSPSAKIKSA  695



Lambda      K        H        a         alpha
   0.317    0.134    0.372    0.792     4.96

Gapped
Lambda      K        H        a         alpha    sigma
   0.267   0.0410    0.140     1.90     42.6     43.6

Effective search space used: 427712



Matrix: BLOSUM62
Gap Penalties: Existence: 11, Extension: 1
Neighboring words threshold: 11
Window for multiple hits: 40



Query= sp|Q93ZC5|AOC4_ARATH_Allene_oxide_cyclase_4,
_chloroplastic_OS=Arabidopsis_thaliana_GN=AOC4_PE=2_SV=1

Length=254

Subject= 13447-660_2_ORF1
>sp|Q93ZC5|AOC4_ARATH_Allene_oxide_cyclase_4,
_chloroplastic_OS=Arabidopsis_thaliana_GN=AOC4_PE=2_SV=1|||3e-70

Length=274
```

```
 Score =   207 bits (526),  Expect = 1e-69, Method: Compositional
matrix adjust.
 Identities = 106/181 (59%), Positives = 129/181 (71%), Gaps = 8/181
(4%)

Query  81   IQELNVYEFNEGDRNSPAVLKLGKKPD------
QLCLGDLVPFTNKLYTGDLTKRIGITA  134
            +QE+ VYEFNE DR SPA L    K D         +GDLVPFTNKLY G L  R
+GITA
Sbjct  95
VQEMFVYEFNENDRGSPAYLPFSLKFDFSKGLPTGAIGDLVPFTNKLYDGTLQTRLGITA  154

Query  135
GLCVLIQHVPEKKGDRFEASYSFYFGDYGHISVQGPYLTYEDTFLAITGGSGVFEGAYGQ  194
            G+ +LI++ PEKKG R+EA +SFYFGDYGHISVQG YLT+ED+ LA+TGGSGVF G
YG
Sbjct  155
GMTILIKYYPEKKGHRYEAVFSFYFGDYGHISVQGSYLTFEDSELAVTGGSGVFTGVYGV  214

Query  195  VKLRQLVYPTKLFYTFYLKGVAADLPVELTGKH-
VEPSKEVKPAAEAQATQPGATIANFT  253
            VKL Q+ YP KLFYTF L+G+   LP ELT    V P+  V+P+A A  T+PG+
N+T
Sbjct  215  VKLHQIAYPNKLFYTFQLQGIPP-
LPSELTRTPVVRPTPSVRPSAGAARTRPGSVAPNYT  273

Query  254  N  254
            +
Sbjct  274  D  274



Lambda      K        H        a         alpha
   0.317    0.133    0.383    0.792     4.96

Gapped
Lambda      K        H        a         alpha    sigma
   0.267   0.0410    0.140     1.90     42.6     43.6

Effective search space used: 57021




Query= sp|Q93ZC5|AOC4_ARATH_Allene_oxide_cyclase_4,
_chloroplastic_OS=Arabidopsis_thaliana_GN=AOC4_PE=2_SV=1

Length=254

Subject= 371656-35_4_ORF1
>sp|Q93ZC5|AOC4_ARATH_Allene_oxide_cyclase_4,
_chloroplastic_OS=Arabidopsis_thaliana_GN=AOC4_PE=2_SV=1|||3e-70
```

```
Length=278


 Score =   207 bits (526),  Expect = 1e-69, Method: Compositional
matrix adjust.
 Identities = 106/181 (59%), Positives = 130/181 (72%), Gaps = 8/181
(4%)

Query  81   IQELNVYEFNEGDRNSPAVLKLGKKPD------
QLCLGDLVPFTNKLYTGDLTKRIGITA  134
            +QE+ VYEFNE DR SPA L    K D         +GDLVPFTNKLY G L  R
+GITA
Sbjct  99
VQEMFVYEFNEKDRGSPAYLPFSLKFDFSKGLPTGAIGDLVPFTNKLYDGTLQTRLGITA  158

Query  135
GLCVLIQHVPEKKGDRFEASYSFYFGDYGHISVQGPYLTYEDTFLAITGGSGVFEGAYGQ  194
            G+ +LI++ PEKKG R+EA +SFYFGDYGHISVQG YLT+ED+ LA+TGGSGVF G
YG
Sbjct  159
GMTILIKYYPEKKGHRYEAVFSFYFGDYGHISVQGSYLTFEDSELAVTGGSGVFTGVYGV  218

Query  195  VKLRQLVYPTKLFYTFYLKGVAADLPVELTGKH-
VEPSKEVKPAAEAQATQPGATIANFT  253
            VKL Q++YP KLFYTF L+G+   LP ELT    V P+  V+P+A A  T+PG+
N+T
Sbjct  219  VKLHQIMYPNKLFYTFQLQGIPP-
LPSELTRTPVVRPTPSVRPSAGAARTRPGSVAPNYT  277

Query  254  N  254
            +
Sbjct  278  D  278



Lambda      K        H        a         alpha
   0.317    0.133    0.383    0.792     4.96

Gapped
Lambda      K        H        a         alpha    sigma
   0.267   0.0410    0.140     1.90     42.6     43.6

Effective search space used: 57021




Matrix: BLOSUM62
Gap Penalties: Existence: 11, Extension: 1
Neighboring words threshold: 11
Window for multiple hits: 40
```

Query= sp|Q93ZY3|STT3A_ARATH_Dolichyl-diphosphooligosaccharide--protein_glycosyltransferase_subunit_STT3A_OS=Arabidopsis_thaliana_GN=STT3A_PE=1_SV=1

Length=779

Subject= 267991-88_1_ORF2
>sp|Q93ZY3|STT3A_ARATH_Dolichyl-diphosphooligosaccharide--protein_glycosyltransferase_subunit_STT3A_OS=Arabidopsis_thaliana_GN=STT3A_PE=1_SV=1|||0

Length=755

Score = 747 bits (1929), Expect = 0.0, Method: Compositional matrix adjust.
Identities = 392/739 (53%), Positives = 493/739 (67%), Gaps = 52/739 (7%)

```
Query  22
VLSVLILVLIGVLAFSIRLFSVIKYESVIHEFDPYFNYRVTQFLSKNGIYEFWNWFDDRT  81
            +L +  L+LI VLAF+ RLFSV++YES+IHEFDPYFNYR T +L++ G
YEFWNWFD  T
Sbjct  62
LLRIGALMLIYVLAFAARLFSVLRYESMIHEFDPYFNYRTTLYLTQKGFYEFWNWFDYET  121

Query  82
WYPLGRVIGGTVYPGLTLTAGTIWWGLNSLNIPLSVETVCVFTAPVFSAFASWATYLLTK  141
            WYPLGR+IGGT+YPGL +TA  ++W L  L+  + +  VCV TAP F++  +   Y
K
Sbjct  122
WYPLGRIIGGTLYPGLMVTAAVLYWSLRFLSFAVHIRDVCVLTAPFFASNTTIVAYFFGK  181

Query  142
EVKGSGAGLAAAALLAMVPSYISRSVAGSYDNEAVAIFALIFTFYLYIKTLNTGSLFYAT  201
            E+  +GAG+ AAAL+A+ P YISRSVAGSYDNE VAIFAL+ TFYL++K + TGSL
++
Sbjct  182
EIWDTGAGIVAAALIAICPGYISRSVAGSYDNEGVAIFALLLTFYLFVKAVKTGSLAWSV  241

Query  202
LNALAYFYMVCSWGGYTFIINLIPMHVLLCIVTGRYSPRLYIAYAPLVVLGTLLAALVPV  261
             +A AYFYMV +WGGY FIINLIP++VL  +VTGRYS RLY+AY  + +LG LLA
+
Sbjct  242
ASAFAYFYMVSAWGGYIFIINLIPLYVLALLVTGRYSLRLYVAYNTMYILGMLLAMQIRF  301

Query  262  VGFNAVLTSEHFASFLVFIIIHVVALVYYIKGIL-
SPKMFKVAVTLVVSIGMVVCFIVVA  320
            VGF  V + EH A+  VF++  V  +V ++K  L +PK+F+  + + V    VV
+ +A
Sbjct  302
```

VGFQHVQSGEHMAAMGVFLLSQVFYMVNWVKDQLDNPKLFRAFLRVTVLTATVVGGLALA 361

Query 321
ILVALVASSPTGGWSGRSLSLLDPTYASKYIPIIASVSEHQPPTWPSYFMDINVLAFLVP 380
            + +A   SP   W+GR SLLDPTYA +IPIIASVSEHQP W S+ D +VL
L P
Sbjct 362 LGMATGYISP---
WTGRFYSLLDPTYAKDHIPIIASVSEHQPTAWSSFMFDFHVLLILFP 418

Query 381
AGIIACFSPLSDASSFVVLYIVMSVYFSGVMVRLMLVLAPAACIMSGIALSQAFDVFTGS 440
            AG+ CF L+DA+ F+++Y + S+YF+GVMVRL+LV APA C++S IA+S
+G
Sbjct 419
AGLYFCFKRLTDATIFIIIYGLTSLYFAGVMVRLILVAAPAVCLISAIAVSATLKNLSGL 478

Query 441
IKYQLGASSNSTDDAEDNTSTNNAPKDDVSAGKTDKGEEIVKERSSKKGKKKEREPADKP 500
            ++   S   A   T T+ + K   S KG   + P
Sbjct 479 VR--------SKAQATGPTKTSGSAK-----------------
GSSKGISDQSLPMQ-- 510

Query 501
SVKAKIKKKALVLPLEASIVALLLLIMLGAFYVIHCVWAAAEAYSAPSIVLTSQSRDGLH 560
                  KK A VL   I+ +L L+M   YVIHC W +EAYS+PSIVL ++
+G
Sbjct 511 ------KKGAAVL-----ILGVLYLLMS---
YVIHCTWVTSEAYSSPSIVLAARQSNGDK 556

Query 561 V-
FDDFRESYAWLSHNTDVDDKVASWWDYGYQTTAMANRTVIVDNNTWNNTHIATVGTAM 619
            V FDDFRE+Y WL HNT D KV SWWDYGYQ TAM
NRTVIVDNNTWNNTHIATVG AM
Sbjct 557
VIFDDFREAYYWLRHNTPEDAKVMSWWDYGYQITAMGNRTVIVDNNTWNNTHIATVGRAM 616

Query 620
SSPEKAAWEIFNSLDVKYVLVVFGGLIGYPSDDINKFLWMVRIGGGVFPHIKEADYLRDG 679
            SS E A+EI   LDV YVLVVFGG+ GY SDDINKFLWMVRIGGGVFP IKE
DYL +G
Sbjct 617
SSYEDEAYEIMQELDVSYVLVVFGGVTGYSSDDINKFLWMVRIGGGVFPVIKEPDYLVNG 676

Query 680 QYRIDSEATPTMLNSLMYKLSYYRFVE---TDGK--
GYDRVRRTEIGKKHFKLTHFEEVF 734
             YR+D A P ML LMYKL YYRF E   GK G+DR R EIG K   L H
EE
Sbjct 677
DYRVDKGAAPKMLQCLMYKLCYYRFGELMTEYGKPPGWDRARGVEIGNKDIVLDHLEEAL 736

Query 735 TSHHWMVRLYKLKPPRNRI 753
            T+ +W+VR+YK+K P+NR+
Sbjct 737 TTTNWIVRIYKVKLPKNRV 755

```
Lambda      K        H        a         alpha
   0.323    0.136    0.415    0.792     4.96

Gapped
Lambda      K        H        a         alpha    sigma
   0.267   0.0410    0.140     1.90     42.6     43.6

Effective search space used: 526932
```

```
Matrix: BLOSUM62
Gap Penalties: Existence: 11, Extension: 1
Neighboring words threshold: 11
Window for multiple hits: 40
```

```
Query= sp|Q944K2|OST48_ARATH_Dolichyl-diphosphooligosaccharide--
protein_glycosyltransferase_48_kDa_subunit_OS=Arabidopsis_thaliana_G
N=OST48_PE=2_SV=1

Length=437

Subject= 229904-113_1_ORF2
>sp|Q944K2|OST48_ARATH_Dolichyl-diphosphooligosaccharide--
protein_glycosyltransferase_48_kDa_subunit_OS=Arabidopsis_thaliana_G
N=OST48_PE=2_SV=1|||0

Length=449


 Score =   549 bits (1415),  Expect = 0.0, Method: Compositional
matrix adjust.
 Identities = 272/418 (65%), Positives = 334/418 (80%), Gaps = 0/418
(0%)

Query  20
SFSFSVDNPTDRRVLVLLDDLSLKSSHSIFFNTLKSRGFDLDFKLAEDSKLALQRYGQYL  79
           S   S D     R+LVLLDDL++KSSHS FF +L  RG++LDFKLA D  LAL
RYG+YL
Sbjct  32
SLCLSPDRSLHPRLLVLLDDLAVKSSHSFFFKSLTDRGYELDFKLASDPSLALNRYGEYL  91

Query  80
YDGLIIFAPSTERFGGSLDSKSIADFVDSGRDLILSADTAASDLIRGIATECGVDFDEDS  139
            +DGLI+F P  E FGGS+D+ SI DFVDSGRDLI++AD   SD IR IA ECGV
+FDED
Sbjct  92
```

```
FDGLILFTPFAENFGGSVDAASILDFVDSGRDLIIAADLGMSDTIRDIANECGVEFDEDP  151

Query  140
SAMVIDHTSFSVSDVDGDHTLIAADDLVKSDVILGKTKIEAPVLFRGVAHSLNPTNNLVL  199
             ++VIDH +F++++   +H+LIAAD  V S+ ILG   IEAPVLF+G+AHS+   N
+LV
Sbjct  152
DSVVIDHLNFAITESGIEHSLIAADTFVMSNAILGDKGIEAPVLFQGIAHSVASANSLVT  211

Query  200
KVLSASPSAYSANPSSKLSSPPQLTGSSISLVSVMQARNNARVVISGSVQLFSDRLIRSG  259
            KVLSAS SAYSANP ++L  PP LTGSSI LVSV+QARNNAR++ISGS+ LFS++
S
Sbjct  212
KVLSASSSAYSANPEAQLGDPPALTGSSIGLVSVVQARNNARILISGSLSLFSNKFFTSP  271

Query  260
VQKAGSPNQYEKSGNEQFVTELSKWVFHERGHLKAGNLVHHRVGETDEPAIYRIKDDLEF  319
            V+K GS   +EKSGNEQF  ELSKW FHERGHLKA N+ HH+ GE  EP +YRI D
LE+
Sbjct  272
VKKFGSSTSHEKSGNEQFAMELSKWTFHERGHLKAVNIQHHKAGEVQEPHMYRITDHLEY  331

Query  320
SVEIYEWSGKSWEPYVANDVQVQFYMMSPYVLKTLSTDKKGLFHTSFKVPDVYGVFQFKV  379
            S+ IYEW+GK+W+PY+A+DVQVQFYMMSPYVLKTL  + +GL+HTSF
+VPDVYGVFQFK+
Sbjct  332
SLAIYEWTGKNWQPYIADDVQVQFYMMSPYVLKTLDHNGQGLYHTSFQVPDVYGVFQFKL  391

Query  380
EYEKLGYTTLSLSKQIPVRPYRHNEYERFIPTAYPYYGACFTTMAGFFVFSFVYLYHK  437
            EY +LGYT+LSLSKQIPVRP+RH+EYERFI  AYPYY +CF+ M GFF+F  +
+LYHK
Sbjct  392
EYNRLGYTSLSLSKQIPVRPFRHDEYERFIRAAYPYYSSCFSMMFGFFIFGVLFLYHK  449



Lambda      K        H        a         alpha
   0.319    0.135    0.389    0.792     4.96

Gapped
Lambda      K        H        a         alpha    sigma
   0.267   0.0410    0.140     1.90     42.6     43.6

Effective search space used: 168885



Query= sp|Q944K2|OST48_ARATH_Dolichyl-diphosphooligosaccharide--
protein_glycosyltransferase_48_kDa_subunit_OS=Arabidopsis_thaliana_G
N=OST48_PE=2_SV=1
```

Length=437

Subject= 303509-66_2_ORF2
>sp|Q944K2|OST48_ARATH_Dolichyl-diphosphooligosaccharide--protein_glycosyltransferase_48_kDa_subunit_OS=Arabidopsis_thaliana_GN=OST48_PE=2_SV=1|||0

Length=449

Score = 548 bits (1412), Expect = 0.0, Method: Compositional matrix adjust.
Identities = 271/418 (65%), Positives = 333/418 (80%), Gaps = 0/418 (0%)

```
Query  20
SFSFSVDNPTDRRVLVLLDDLSLKSSHSIFFNTLKSRGFDLDFKLAEDSKLALQRYGQYL  79
            S   S D     R+LVLLDDL++KSSHS FF +L  RG++LDFKLA D  L L
RYG+YL
Sbjct  32
SLCLSPDRSLHPRLLVLLDDLAVKSSHSFFFKSLTDRGYELDFKLASDPSLGLNRYGEYL  91

Query  80
YDGLIIFAPSTERFGGSLDSKSIADFVDSGRDLILSADTAASDLIRGIATECGVDFDEDS  139
            +DGLI+F P  E FGGS+D+ SI DFVDSGRDLI++AD   SD IR IA ECGV
+FDED
Sbjct  92
FDGLILFTPFAENFGGSVDAASILDFVDSGRDLIIAADLGMSDTIRDIANECGVEFDEDP  151

Query  140
SAMVIDHTSFSVSDVDGDHTLIAADDLVKSDVILGKTKIEAPVLFRGVAHSLNPTNNLVL  199
             ++VIDH +F++++   +H+LIAAD  V S+ ILG   IEAPVLF+G+AHS+   N
+LV
Sbjct  152
DSVVIDHLNFAITESGIEHSLIAADTFVMSNAILGDKGIEAPVLFQGIAHSVASANSLVT  211

Query  200
KVLSASPSAYSANPSSKLSSPPQLTGSSISLVSVMQARNNARVVISGSVQLFSDRLIRSG  259
            KVLSAS SAYSANP ++L  PP LTGSSI LVSV+QARNNAR++ISGS+ LFS++
S
Sbjct  212
KVLSASSSAYSANPEAQLGDPPALTGSSIGLVSVVQARNNARILISGSLSLFSNKFFTSP  271

Query  260
VQKAGSPNQYEKSGNEQFVTELSKWVFHERGHLKAGNLVHHRVGETDEPAIYRIKDDLEF  319
            V+K GS   +EKSGNEQF  ELSKW FHERGHLKA N+ HH+ GE  EP +YRI D
LE+
Sbjct  272
VKKFGSSTSHEKSGNEQFAMELSKWTFHERGHLKAVNIQHHKAGEVQEPHMYRITDHLEY  331

Query  320
SVEIYEWSGKSWEPYVANDVQVQFYMMSPYVLKTLSTDKKGLFHTSFKVPDVYGVFQFKV  379
            S+ IYEW+GK+W+PY+A+DVQVQFYMMSPYVLKTL  + +GL+HTSF
```

```
+VPDVYGVFQFK+
Sbjct  332
SLAIYEWTGKNWQPYIADDVQVQFYMMSPYVLKTLEHNGQGLYHTSFQVPDVYGVFQFKL  391

Query  380
EYEKLGYTTLSLSKQIPVRPYRHNEYERFIPTAYPYYGACFTTMAGFFVFSFVYLYHK  437
            EY +LGYT+LSLSKQIPVRP+RH+EYERFI  AYPYY +CF+ M GFF+F  +
+LYHK
Sbjct  392
EYNRLGYTSLSLSKQIPVRPFRHDEYERFIRAAYPYYSSCFSMMFGFFIFGVLFLYHK  449



Lambda      K        H        a         alpha
   0.319    0.135    0.389    0.792     4.96

Gapped
Lambda      K        H        a         alpha    sigma
   0.267   0.0410    0.140     1.90     42.6     43.6

Effective search space used: 168885




Matrix: BLOSUM62
Gap Penalties: Existence: 11, Extension: 1
Neighboring words threshold: 11
Window for multiple hits: 40





Query= sp|Q95M18|
ENPL_BOVIN_Endoplasmin_OS=Bos_taurus_GN=HSP90B1_PE=2_SV=1

Length=804

Subject= 1610-1504_2_ORF1
>sp|Q95M18|ENPL_BOVIN_Endoplasmin_OS=Bos_taurus_GN=HSP90B1_PE=2_SV=1
|||0

Length=599


 Score =   533 bits (1372),  Expect = 0.0, Method: Compositional
matrix adjust.
 Identities = 303/606 (50%), Positives = 426/606 (70%), Gaps =
12/606 (2%)

Query  180  EAQEDGQSTSELIGQFGVGFYSAFLVADKVIVTSKHNNDTQHIWESDS-
NEFSVIADPRG  238
            EA   G   S +IGQFGVGFYSA+LVA+KV+V +KHN+D Q+IWES +   F+V
```

```
DP G
Sbjct  4    EALAAGADVS-
MIGQFGVGFYSAYLVAEKVVVITKHNDDEQYIWESQAGGSFTVTRDP-G  61

Query  239
NTLGRGTTITLVLKEEASDYLELDTIKNLVKKYSQFINFPIYVWSSKTETVEEPAEEEEA  298
              LGRGT ITL LKE+  +Y+E   +K+LVKK+S+FI++PI +W  KT T E
+EE+
Sbjct  62
EVLGRGTKITLYLKEDQLEYVEERRLKDLVKKHSEFISYPISLWVEKTTTKEVSDDEEDE  121

Query  299
AKEDKEESDDEAAVEEEEDEKKPKTKKVEKTVWDWELMNDIKPIWQRPSKEVEEDEYKAF  358
             KEDK+E + +A   EE+ EK+ K KKV++   +W L+N  KPIW R  +E+
+DEY AF
Sbjct  122
EKEDKDEEEGKAEEIEEDKEKEKKKKKVQEVSHEWALVNKQKPIWMRKPEEITKDEYAAF  181

Query  359
YKSFSKESDDPMAYIHFTAEGEVTFKSILFVPTSAPRGLFDEYGSKKSDYIKLYVRRVFI  418
            YKS + + ++ +A  HF+ EG++ FK++LFVP  AP  LFD    KK++
IKLYVRRVFI
Sbjct  182  YKSLTNDWEEHLAVKHFSVEGQLEFKAVLFVPKRAPFDLFDT--
RKKANNIKLYVRRVFI  239

Query  419
TDDFHDMMPKYLNFVKGVVDSDDLPLNVSRETLQQHKLLKVIRKKLVRKTLDMIKKIADE  478
             D+  +++P+YL+FVKGVVDS+DLPLN+SRE LQQ+K+LKVIRK LV+K +++
+IA+
Sbjct  240
MDNCEEIIPEYLSFVKGVVDSEDLPLNISREMLQQNKILKVIRKNLVKKCVELFFEIAEN  299

Query  479  KYN-
DTFWKEFGTNIKLGVIEDHSNRTRLAKLLRFQSSHHPSDMTSLDQYVERMKEKQDK  537
            K + D F++ F  NIKLG+ ED  NR++ A+LLR+ S+    +MTSL  YV RMK+
Q
Sbjct  300
KEDYDKFYEAFSKNIKLGIHEDSQNRSKYAELLRYHSTKSGDEMTSLKDYVTRMKDGQTD  359

Query  538
IYFMAGASRKEAESSPFVERLLKKGYEVIYLTEPVDEYCIQALPEFDGKRFQNVAKEGVK  597
            I+++ G S+K  E+SPF+E+L KKGYEV+Y+ + +DEY +  L EFDGK+  +
KEG+K
Sbjct  360
IFYITGESKKAVENSPFLEKLRKKGYEVLYMVDAIDEYAVGQLKEFDGKKLVSATKEGLK  419

Query  598
FDESEKSKESREAVEKEFEPLLNWMKDKALKDKIEKAVVSQRLTESPCALVASQYGWSGN  657
             D+SE   + +E ++ +FEPL   +KD  L DK+EK VVS R+ +SPC LV
+YGW+ N
Sbjct  420  LDDSEDENKRKEELKSKFEPLCKVVKD-
ILGDKVEKVVVSDRVVDSPCCLVTGEYGWTAN  478

Query  658
```

MERIMKAQAYQTGKDISTNYYASQKKTFEINPRHPLIRDMLRRVKEDEDDKTVSDLAVVL 717
MERIMKAQA   +D S + Y S KKT EINP +P++ ++ +R   D++DK+V DL
++L
Sbjct 479 MERIMKAQAL---
RDSSMSSYMSSKKTMEINPENPIMEELRKRADVDKNDKSVKDLVLLL 535

Query 718
FETATLRSGYLLPDTKAYGDRIERMLRLSLNIDPDAKVEEEPEEEPEETTEDTAEDTEQD 777
FETA L SG+ L D   +G+RI RML+L L+ID DA   E +E+ +   +D   D
E
Sbjct 536 FETALLTSGFSLEDPNTFGNRIHRMLKLGLSIDDDAGTSAEADEDMDAPVDDA--
DAEGS 593

Query 778 EEEEMD 783
+ EE+D
Sbjct 594 KMEEVD 599

Lambda   K   H   a   alpha
0.311   0.130   0.359   0.792   4.96

Gapped
Lambda   K   H   a   alpha   sigma
0.267   0.0410   0.140   1.90   42.6   43.6

Effective search space used: 428400

Matrix: BLOSUM62
Gap Penalties: Existence: 11, Extension: 1
Neighboring words threshold: 11
Window for multiple hits: 40

Query= sp|Q9AR14|PIP15_MAIZE_Aquaporin_PIP1-5_OS=Zea_mays_GN=PIP1-
5_PE=2_SV=1

Length=288

Subject= 82340-277_1_ORF2
>sp|Q9AR14|PIP15_MAIZE_Aquaporin_PIP1-5_OS=Zea_mays_GN=PIP1-
5_PE=2_SV=1|||1e-173

Length=302

Score =   474 bits (1219), Expect = 2e-173, Method: Compositional
matrix adjust.

```
 Identities = 231/292 (79%), Positives = 256/292 (88%), Gaps = 7/292
(2%)

Query  1
MEGKEEDVRLGANRYSERQPIGTAAQGTEEKDYKEPPPAPLFEAEELTSWSFYRAGIAEF  60
            ME K+EDVRLGAN+++ERQP+GTAAQ    +DY EPP   LFE  E +SWSF
+RAGIAEF
Sbjct  12   MESKDEDVRLGANKFNERQPLGTAAQ---
TRDYTEPPATRLFEPAEFSSWSFWRAGIAEF  68

Query  61   VATFLFLYISILTVMG----
VSKSSSKCATVGIQGIAWSFGGMIFALVYCTAGISGGHIN  116
             AT LFLYISI TVMG    V  + ++C  VGIQGIAW
+FGGMIFALVYCTAGISGGHIN
Sbjct  69
FATLLFLYISIQTVMGYKHGVPSAKNECPGVGIQGIAWAFGGMIFALVYCTAGISGGHIN  128

Query  117
PAVTFGLFLARKLSLTRALFYMVMQCLGAICGAGVVKGFQEGLYMGAGGGANAVNPGYTK  176
            PAVT+GLFLARK+SL R L+Y++MQCLGAICGAG+VKGFQ   Y   GGGAN VN
GYTK
Sbjct  129
PAVTWGLFLARKVSLPRTLYYIIMQCLGAICGAGIVKGFQPDFYNNNGGGANVVNHGYTK  188

Query  177
GDGLGAEIVGTFVLVYTVFSATDAKRSARDSHVPILAPLPIGFAVFLVHLATIPITGTGI  236
            GDGLGAEIVGTFVLVYTVFSATDAKRSARDSHVP
+LAPLPIGFAVFLVHLATIPITGTGI
Sbjct  189
GDGLGAEIVGTFVLVYTVFSATDAKRSARDSHVPLLAPLPIGFAVFLVHLATIPITGTGI  248

Query  237  NPARSLGAAIVYNRSHAWNDHWIFWVGPFIGAALAAIYHVVIIRALPFKSRD
288
            NPARSLGAAI+YN+ HAWNDHWIFWVGPFIGA+LA +YHVV+IRALPFKSR+
Sbjct  249  NPARSLGAAIIYNQKHAWNDHWIFWVGPFIGASLACLYHVVVIRALPFKSRE
300



Lambda      K        H        a         alpha
   0.323    0.140    0.432    0.792     4.96

Gapped
Lambda      K        H        a         alpha    sigma
   0.267   0.0410    0.140     1.90     42.6     43.6

Effective search space used: 72312



Matrix: BLOSUM62
Gap Penalties: Existence: 11, Extension: 1
Neighboring words threshold: 11
```

Window for multiple hits: 40

Query= sp|Q9FL28|FLS2_ARATH_LRR_receptor-like_serine/threonine-protein_kinase_FLS2_OS=Arabidopsis_thaliana_GN=FLS2_PE=1_SV=1

Length=1173

Subject= 108318-239_5_ORF2 >sp|Q9FL28|FLS2_ARATH_LRR_receptor-like_serine/threonine-protein_kinase_FLS2_OS=Arabidopsis_thaliana_GN=FLS2_PE=1_SV=1|||3e-55

Length=434

Score = 188 bits (478), Expect = 1e-55, Method: Compositional matrix adjust.
Identities = 131/360 (36%), Positives = 186/360 (52%), Gaps = 39/360 (11%)

```
Query  32   EALKSFKNGISNDPLGVLSDWTIIG-
SLRHCNWTGITCDSTGHVVSVSLLEKQLEGVLSP  90
            +AL  FK  IS+DP   LS W   G S   CNW GITCD+TG V+ V L ++ L+G
+S
Sbjct  70
KALLQFKASISSDPNNALSTWIASGNSSNCCNWNGITCDATGRVIRVKLPQQNLKGAISS  129

Query  91   AIANLTYLQVLDLTSNSFTGKIPAEIGKLTELNQLILYLN-
YFSGSIPSGIWELKNIFYL  149
            ++  L+ LQVL L  N  +G +P+ +GKL  L +L ++ N   SGSIP+   +LK+
+  L
Sbjct  130
SLGALSALQVLILYENKLSGSLPSSLGKLLRLQRLCIFGNPSVSGSIPASFGQLKSLQLL  189

Query  150
DLRNNLLSGDVPEEICKTSSLVLIGFDYNNLTGKIPECLGDLVHLQMFVAAGNHLTGSIP  209
            DL NN LSG +P  I   SSL+                     H++++   GN +
G IP
Sbjct  190  DLSNNALSGSLPTNIGGMSSLM---------------------HIRIY---
GNKIGGQIP  225

Query  210  VSIGTLANLTDLDLSGNQLTGKIPRDFG-
NLLNLQSLVLTENLLEGDIPAEIGNCSSLVQ  268
             S G L+ L + DL  NQL+G+IP  F   L +L  L L  N + G +PA + N +
L
Sbjct  226  ASFGRLSQLFNADLGNNQLSGRIPNGFAYGLSSLAFLFLENNRITG-
LPANLHNLTRLQW  284

Query  269  LELYDNQLTGK-IPAELGNLVQLQALRIYKNKLTSSIPSSLFRLTQ----------
LTHL  317
```

```
            ++L +N LT     A L     +  + +   KL+   PS + RL Q
+
Sbjct  285
VDLSNNPLTNSDAVAGLATAPLVVQIELASCKLSGPFPSWVSRLPQPDFDLISDEVTPSI  344

Query  318
GLSENHLVGPISEEIGFLESLEVLTLHSNNFTGEFPQSITNLRNLTVLTVGFNNISGELP  377
             LS N + GPI   +G L  LE L L  N  TG  P S +NL++L    V  N
+SG++P
Sbjct  345
DLSNNAITGPIPAAVGNLTGLEGLNLAQNQLTGMLPASFSNLQSLRTFNVSNNQLSGQIP  404


 Score =   157 bits (398),  Expect = 5e-45, Method: Compositional
matrix adjust.
 Identities = 114/339 (34%), Positives = 171/339 (50%), Gaps =
19/339 (6%)

Query  466
NLTGTLKPLIGKLQKLRILQVSYNSLTGPIPREIGNLKDLNILYLHSNGFTGRIPREMSN  525
            N  G      G++ ++++ Q    +L G I   +G L  L +L L+ N  +G +P
+
Sbjct  101  NWNGITCDATGRVIRVKLPQ---
QNLKGAISSSLGALSALQVLILYENKLSGSLPSSLGK  157

Query  526  LTLLQGLRMYSN-
DLEGPIPEEMFDMKLLSVLDLSNNKFSGQIPALFSKLESLTYLSLQG  584
            L  LQ L ++ N  + G IP     +K L +LDLSNN  SG +P     + SL ++
+ G
Sbjct  158
LLRLQRLCIFGNPSVSGSIPASFGQLKSLQLLDLSNNALSGSLPTNIGGMSSLMHIRIYG  217

Query  585
NKFNGSIPASLKSLSLLNTFDISDNLLTGTIPGELLASLKNMQLYLNFSNNLLTGTIPKE  644
            NK  G IPAS   LS L   D+ +N L+G IP      L ++  +L   NN +TG
+P
Sbjct  218  NKIGGQIPASFGRLSQLFNADLGNNQLSGRIPNGFAYGLSSLA-FLFLENNRITG-
LPAN  275

Query  645  LGKLEMVQEIDLSNNLFSGS-IPRSLQACKNVFTLDFSQNNLSGHIPDEVFQ----
GMDM  699
            L  L  +Q +DLSNN  + S     L     V  ++ +   LSG  P  V +
D+
Sbjct  276
LHNLTRLQWVDLSNNPLTNSDAVAGLATAPLVVQIELASCKLSGPFPSWVSRLPQPDFDL  335

Query  700  I-----
ISLNLSRNSFSGEIPQSFGNMTHLVSLDLSSNNLTGEIPESLANLSTLKHLKLA  754
            I      S++LS N+ +G IP + GN+T L  L+L+ N LTG +P S +NL +L+
++
Sbjct  336
ISDEVTPSIDLSNNAITGPIPAAVGNLTGLEGLNLAQNQLTGMLPASFSNLQSLRTFNVS  395

Query  755  SNNLKGHVPESGVFKNINASDLM-GNTDLCGSKKPLKPC  792
```

```
            +N L G +P+   F     S    GN  LCG+  PL  C
Sbjct  396  NNQLSGQIPQVAPFTTFAVSAYQPGNAALCGT--PLPAC  432


 Score =   136 bits (342),  Expect = 6e-38, Method: Compositional
matrix adjust.
 Identities = 101/318 (32%), Positives = 163/318 (51%), Gaps =
41/318 (13%)

Query  266
LVQLELYDNQLTGKIPAELGNLVQLQALRIYKNKLTSSIPSSLFRLTQLTHLGLSENHLV  325
            +++++L    L G I + LG L  LQ L +Y+NKL+ S+PSSL +L +L  L +
N  V
Sbjct  113
VIRVKLPQQNLKGAISSSLGALSALQVLILYENKLSGSLPSSLGKLLRLQRLCIFGNPSV  172

Query  326  -
GPISEEIGFLESLEVLTLHSNNFTGEFPQSITNLRNLTVLTVGFNNISGELPADLGLLT  384
             G I    G L+SL++L L +N  +G  P +I  + +L  + +  N I G++PA
G L+
Sbjct  173
SGSIPASFGQLKSLQLLDLSNNALSGSLPTNIGGMSSLMHIRIYGNKIGGQIPASFGRLS  232

Query  385  NLRNLSAHDNLLTGPIPSSISNCTGLKLLDLSHNQMTGEIPRGF--
GRMNLTFISIGRNH  442
             L N                        DL +NQ++G IP GF  G  +L F+ +
N
Sbjct  233  QLFNA------------------------
DLGNNQLSGRIPNGFAYGLSSLAFLFLENNR  268

Query  443  FTGEIPDDIFNCSNLETLSVADNNLTGTLKPLIGKLQKLRILQVSYNS--
LTGPIPREIG  500
             TG +P ++ N + L+ + +++N LT +   + G      ++Q+   S  L+GP P
+
Sbjct  269  ITG-LPANLHNLTRLQWVDLSNNPLTNS-
DAVAGLATAPLVVQIELASCKLSGPFPSWVS  326

Query  501  NL--KDLNILY--------
LHSNGFTGRIPREMSNLTLLQGLRMYSNDLEGPIPEEMFDM  550
             L   D +++         L +N  TG IP  + NLT L+GL +  N L G +P
++
Sbjct  327
RLPQPDFDLISDEVTPSIDLSNNAITGPIPAAVGNLTGLEGLNLAQNQLTGMLPASFSNL  386

Query  551  KLLSVLDLSNNKFSGQIP  568
            + L   ++SNN+ SGQIP
Sbjct  387  QSLRTFNVSNNQLSGQIP  404


 Score =   130 bits (326),  Expect = 5e-36, Method: Compositional
matrix adjust.
 Identities = 93/295 (32%), Positives = 150/295 (51%), Gaps = 16/295
(5%)
```

```
Query  146
IFYLDLRNNLLSGDVPEEICKTSSLVLIGFDYNNLTGKIPECLGDLVHLQMFVAAGN-HL  204
            +  + L    L G +   +   S+L ++    N L+G +P  LG L+ LQ
GN  +
Sbjct  113
VIRVKLPQQNLKGAISSSLGALSALQVLILYENKLSGSLPSSLGKLLRLQRLCIFGNPSV  172

Query  205
TGSIPVSIGTLANLTDLDLSGNQLTGKIPRDFGNLLNLQSLVLTENLLEGDIPAEIGNCS  264
            +GSIP S G L +L  LDLS N L+G +P + G + +L  + +  N + G IPA
G  S
Sbjct  173
SGSIPASFGQLKSLQLLDLSNNALSGSLPTNIGGMSSLMHIRIYGNKIGGQIPASFGRLS  232

Query  265
SLVQLELYDNQLTGKIPAELGNLVQLQALRIYKNKLTSSIPSSLFRLTQLTHLGLSENHL  324
             L   +L +NQL+G+IP      +   A    +N   + +P++L  LT+L  + LS
N L
Sbjct  233
QLFNADLGNNQLSGRIPNGFAYGLSSLAFLFLENNRITGLPANLHNLTRLQWVDLSNNPL  292

Query  325  VGPISEEIGFLESLEVLT---LHSNNFTGEFPQSITNLRN----------
LTVLTVGFNN  371
                S+ +  L +  ++    L S   +G FP  ++ L               + +
N
Sbjct  293  TN--
SDAVAGLATAPLVVQIELASCKLSGPFPSWVSRLPQPDFDLISDEVTPSIDLSNNA  350

Query  372  ISGELPADLGLLTNLRNLSAHDNLLTGPIPSSISNCTGLKLLDLSHNQMTGEIPR
426
            I+G +PA +G LT L  L+   N LTG +P+S SN   L+  ++S+NQ++G+IP+
Sbjct  351  ITGPIPAAVGNLTGLEGLNLAQNQLTGMLPASFSNLQSLRTFNVSNNQLSGQIPQ
405


 Score =   129 bits (323),  Expect = 1e-35, Method: Compositional
matrix adjust.
 Identities = 103/298 (35%), Positives = 146/298 (49%), Gaps =
17/298 (6%)

Query  203  HLTGSIPVSIGTLANLTDLDLSGNQLTGKIPRDFGNLLNLQSLVLTENL-
LEGDIPAEIG  261
            +L G+I  S+G L+ L  L L  N+L+G +P   G LL LQ L +  N  + G
IPA  G
Sbjct  122
NLKGAISSSLGALSALQVLILYENKLSGSLPSSLGKLLRLQRLCIFGNPSVSGSIPASFG  181

Query  262
NCSSLVQLELYDNQLTGKIPAELGNLVQLQALRIYKNKLTSSIPSSLFRLTQLTHLGLSE  321
               SL  L+L +N L+G +P  +G +  L  +RIY NK+   IP+S  RL+QL +
L
Sbjct  182
QLKSLQLLDLSNNALSGSLPTNIGGMSSLMHIRIYGNKIGGQIPASFGRLSQLFNADLGN  241
```

Query 322 NHLVGPISEEIGF-LESLEVLTLHSNNFTGEFPQSITNLRNLTVLTVGFNNIS-
GELPAD 379
N L G I + L SL L L +N TG P ++ NL L + + N ++ +
A
Sbjct 242 NQLSGRIPNGFAYGLSSLAFLFLENNRITG-
LPANLHNLTRLQWVDLSNNPLTNSDAVAG 300

Query 380
LGLLTNLRNLSAHDNLLTGPIPSSISNCTGLKLLDLSHNQMTGEIPRGFGRMNLTFISIG 439
L + + L+GP PS +S DL +++T I
+
Sbjct 301 LATAPLVVQIELASCKLSGPFPSWVSRLPQPDF-
DLISDEVTPSI------------DLS 347

Query 440
RNHFTGEIPDDIFNCSNLETLSVADNNLTGTLKPLIGKLQKLRILQVSYNSLTGPIPR 497
N TG IP + N + LE L++A N LTG L LQ LR VS N L+G
IP+
Sbjct 348
NNAITGPIPAAVGNLTGLEGLNLAQNQLTGMLPASFSNLQSLRTFNVSNNQLSGQIPQ 405

Score = 117 bits (292), Expect = 9e-32, Method: Compositional
matrix adjust.
Identities = 89/289 (31%), Positives = 149/289 (52%), Gaps = 15/289
(5%)

Query 247 LTENLLEGDIPAEIGNCSSLVQLELYDNQLTGKIPAELGNLVQLQALRIYKN-
KLTSSIP 305
L + L+G I + +G S+L L LY+N+L+G +P+ LG L++LQ L I+ N ++
SIP
Sbjct 118
LPQQNLKGAISSSLGALSALQVLILYENKLSGSLPSSLGKLLRLQRLCIFGNPSVSGSIP 177

Query 306
SSLFRLTQLTHLGLSENHLVGPISEEIGFLESLEVLTLHSNNFTGEFPQSITNLRNLTVL 365
+S +L L L LS N L G + IG + SL + ++ N G+ P S L
L
Sbjct 178
ASFGQLKSLQLLDLSNNALSGSLPTNIGGMSSLMHIRIYGNKIGGQIPASFGRLSQLFNA 237

Query 366 TVGFNNISGELPADLGL-
LTNLRNLSAHDNLLTGPIPSSISNCTGLKLLDLSHNQMT-GE 423
+G N +SG +P L++L L +N +TG +P+++ N T L+ +DLS+N
+T +
Sbjct 238 DLGNNQLSGRIPNGFAYGLSSLAFLFLENNRITG-
LPANLHNLTRLQWVDLSNNPLTNSD 296

Query 424 IPRGFGRMNLTF-ISIGRNHFTGEIPD----------
DIFNCSNLETLSVADNNLTGTLK 472
G L I + +G P D+ + ++ +++N
+TG +
Sbjct 297
AVAGLATAPLVVQIELASCKLSGPFPSWVSRLPQPDFDLISDEVTPSIDLSNNAITGPIP 356

```
Query  473  PLIGKLQKLRILQVSYNSLTGPIPREIGNLKDLNILYLHSNGFTGRIPR  521
              +G L  L  L ++ N LTG +P    NL+ L    + +N  +G+IP+
Sbjct  357  AAVGNLTGLEGLNLAQNQLTGMLPASFSNLQSLRTFNVSNNQLSGQIPQ  405


 Score = 50.1 bits (118),  Expect = 2e-10, Method: Compositional
matrix adjust.
 Identities = 24/62 (39%), Positives = 39/62 (63%), Gaps = 0/62 (0%)

Query  101
LDLTSNSFTGKIPAEIGKLTELNQLILYLNYFSGSIPSGIWELKNIFYLDLRNNLLSGDV  160
            +DL++N+ TG IPA +G LT L  L L  N  +G +P+    L+++   ++ NN
LSG +
Sbjct  344
IDLSNNAITGPIPAAVGNLTGLEGLNLAQNQLTGMLPASFSNLQSLRTFNVSNNQLSGQI  403

Query  161  PE  162
            P+
Sbjct  404  PQ  405



Lambda      K        H        a         alpha
   0.317    0.136    0.385    0.792     4.96

Gapped
Lambda      K        H        a         alpha    sigma
   0.267   0.0410    0.140     1.90     42.6     43.6

Effective search space used: 447930




Query= sp|Q9FL28|FLS2_ARATH_LRR_receptor-like_serine/threonine-
protein_kinase_FLS2_OS=Arabidopsis_thaliana_GN=FLS2_PE=1_SV=1

Length=1173

Subject= 17879-586_6_ORF2
>sp|Q9FL28|FLS2_ARATH_LRR_receptor-like_serine/threonine-
protein_kinase_FLS2_OS=Arabidopsis_thaliana_GN=FLS2_PE=1_SV=1|||1e-
50

Length=432


 Score =   161 bits (408),  Expect = 2e-46, Method: Compositional
matrix adjust.
 Identities = 119/336 (35%), Positives = 177/336 (53%), Gaps =
18/336 (5%)

Query  33   ALKSFKNGISNDPLGVLSDWTIIGSLRH--CNWTGITCD-
```

```
STGHVVSVSLLEKQLEGVLS  89
            AL SFK  IS+DP   L+DW    +  H  C+W G+TCD +T  VV + L  +
+G L+
Sbjct  73   ALLSFKASISSDPNKALADWK---
ASHHNCCDWNGVTCDGATSRVVRLKLANQNFKGNLA  129

Query  90   PAIANLTYLQVLDLTSNSFTGKIPAEIGKLTELNQLILYLN-
YFSGSIPSGIWELKNIFY  148
             +++ L+ LQVL L +N F+G IP+  G    L +L L +N   SG IP    +L
++
Sbjct  130
SSLSALSSLQVLILDNNDFSGHIPSSFGNFRRLQRLCLSVNPSISGPIPESFGQLGSLQL  189

Query  149
LDLRNNLLSGDVPEEICKTSSLVLIGFDYNNLTGKIPECLGDLVHLQMFVAAGNHLTGSI  208
            LDLR+N LSG +P    K S+L+ +    N +TG IP   G L  L      GN +
+G I
Sbjct  190
LDLRSNSLSGPLPASFGKMSNLMNLHLFGNKITGPIPPSFGLLSKLYNADLGGNQISGRI  249

Query  209  PVSIGT-LANLTDLDLSGNQLTGKIPRDFGNLLNLQSLVLTEN-
LLEGDIPAEIGNCSSL  266
            P + G  L +L +L L  N++TG +P D  NL  L+ + L+ N L+ GD    I
+
Sbjct  250  PDAFGYGLTSLINLYLENNRITG-
LPADLRNLTRLEWIDLSNNPLMNGDAVKGIATMPQI  308

Query  267  VQLELYDNQLTGKIPAELGNLVQ--------
LQALRIYKNKLTSSIPSSLFRLTQLTHLG  318
             Q+EL   +++G  P  +  L Q          +L +  N +T +IPS++ +LT L
+L
Sbjct  309
SQIELNSCKISGPFPTWVSRLPQPDTISDEVTPSLDLGNNAITGTIPSAVGKLTNLEYLD  368

Query  319  LSENHLVGPISEEIGFLESLEVLTLHSNNFTGEFPQ  354
            L  N L G I      L+SL    +  N  +G+ PQ
Sbjct  369  LQNNKLTGSIPASFAQLQSLRGFNVSYNQLSGKIPQ  404


 Score =   154 bits (389),  Expect = 6e-44, Method: Compositional
matrix adjust.
 Identities = 112/299 (37%), Positives = 156/299 (52%), Gaps =
20/299 (7%)

Query  507  ILYLHSNGFTGRIPREMSNLTLLQGLRMYSN-
DLEGPIPEEMFDMKLLSVLDLSNNKFSG  565
            +L L +N F+G IP    N   LQ L +  N  + GPIPE    +  L +LDL +N
SG
Sbjct  140
VLILDNNDFSGHIPSSFGNFRRLQRLCLSVNPSISGPIPESFGQLGSLQLLDLRSNSLSG  199

Query  566  QIPALFSKLESLTYLSLQGNKFNGSIPASLKSLSLLNTFDISDNLLTGTIP---
GELLAS  622
             +PA F K+ +L  L L GNK  G IP S   LS L   D+  N ++G IP   G
```

```
L S
Sbjct  200  
PLPASFGKMSNLMNLHLFGNKITGPIPPSFGLLSKLYNADLGGNQISGRIPDAFGYGLTS  259

Query  623  LKNMQLYLNFSNNLLTGTIPKELGKLEMVQEIDLSNN-
LFSGSIPRSLQACKNVFTLDFS  681
            L N  LYL   NN +TG +P +L  L  ++ IDLSNN L +G   + +     +
++ +
Sbjct  260  LIN--LYL--ENNRITG-
LPADLRNLTRLEWIDLSNNPLMNGDAVKGIATMPQISQIELN  314

Query  682  QNNLSGHIPDEVFQ-------
GMDMIISLNLSRNSFSGEIPQSFGNMTHLVSLDLSSNNL  734
               +SG  P  V +         ++  SL+L  N+ +G IP + G +T+L  LDL
+N L
Sbjct  315  
SCKISGPFPTWVSRLPQPDTISDEVTPSLDLGNNAITGTIPSAVGKLTNLEYLDLQNNKL  374

Query  735  TGEIPESLANLSTLKHLKLASNNLKGHVPESGVFKNINASDLM-
GNTDLCGSKKPLKPC  792
            TG IP S A L +L+   ++ N L G +P+   F   + S    GN  LCGS  PL
PC
Sbjct  375  TGSIPASFAQLQSLRGFNVSYNQLSGKIPQVKPFTTFDKSSYTPGNPGLCGS--
PLTPC  431


 Score =   129 bits (325),  Expect = 6e-36, Method: Compositional
matrix adjust.
 Identities = 103/309 (33%), Positives = 159/309 (51%), Gaps =
21/309 (7%)

Query  312  
TQLTHLGLSENHLVGPISEEIGFLESLEVLTLHSNNFTGEFPQSITNLRNLTVLTVGFN-  370
            +++  L L+  +  G ++  +  L SL+VL L +N+F+G  P S  N R L  L +
N
Sbjct  112  
SRVVRLKLANQNFKGNLASSLSALSSLQVLILDNNDFSGHIPSSFGNFRRLQRLCLSVNP  171

Query  371  
NISGELPADLGLLTNLRNLSAHDNLLTGPIPSSISNCTGLKLLDLSHNQMTGEIPRGFGR  430
            +ISG +P   G L +L+ L    N L+GP+P+S    + L  L L  N++TG IP
FG
Sbjct  172  
SISGPIPESFGQLGSLQLLDLRSNSLSGPLPASFGKMSNLMNLHLFGNKITGPIPPSFGL  231

Query  431  MNLTF-ISIGRNHFTGEIPDDI-
FNCSNLETLSVADNNLTGTLKPLIGKLQKLRILQVSY  488
            ++  +   +G N  +G IPD   +  ++L  L + +N +TG L   +  L +L  +
+S
Sbjct  232  LSKLYNADLGGNQISGRIPDAFGYGLTSLINLYLENNRITG-
LPADLRNLTRLEWIDLSN  290

Query  489  NSL-
TGPIPREIGNLKDLNILYLHSNGFTGRIPREMSNLTLLQGLRMYSNDLEGPIPEEM  547
```

```
            N L  G   + I  +  ++ + L+S   +G  P  +S L              P
P+ +
Sbjct  291  NPLMNGDAVKGIATMPQISQIELNSCKISGPFPTWVSRL---------------
PQPDTI  335

Query  548
FDMKLLSVLDLSNNKFSGQIPALFSKLESLTYLSLQGNKFNGSIPASLKSLSLLNTFDIS  607
             D ++   LDL NN  +G IP+   KL +L YL LQ NK  GSIPAS   L  L
F++S
Sbjct  336  SD-
EVTPSLDLGNNAITGTIPSAVGKLTNLEYLDLQNNKLTGSIPASFAQLQSLRGFNVS  394

Query  608  DNLLTGTIP  616
             N L+G IP
Sbjct  395  YNQLSGKIP  403


 Score =  119 bits (297),  Expect = 2e-32, Method: Compositional
matrix adjust.
 Identities = 107/351 (30%), Positives = 165/351 (47%), Gaps =
49/351 (14%)

Query  261
GNCSSLVQLELYDNQLTGKIPAELGNLVQLQALRIYKNKLTSSIPSSLFRLTQLTHLGLS  320
            G  S +V+L+L +    G + + L  L  LQ L +  N  +  IPSS     +L
L LS
Sbjct  109
GATSRVVRLKLANQNFKGNLASSLSALSSLQVLILDNNDFSGHIPSSFGNFRRLQRLCLS  168

Query  321  EN-
HLVGPISEEIGFLESLEVLTLHSNNFTGEFPQSITNLRNLTVLTVGFNNISGELPAD  379
             N  + GPI E  G L SL++L L SN+                        +SG
LPA
Sbjct  169  VNPSISGPIPESFGQLGSLQLLDLRSNS------------------------
LSGPLPAS  204

Query  380  LGLLTNLRNLSAHDNLLTGPIPSSISNCTGLKLLDLSHNQMTGEIPR--
GFGRMNLTFIS  437
             G ++NL NL    N +TGPIP S    + L   DL  NQ++G IP   G+G  +L
+
Sbjct  205
FGKMSNLMNLHLFGNKITGPIPPSFGLLSKLYNADLGGNQISGRIPDAFGYGLTSLINLY  264

Query  438  IGRNHFTGEIPDDIFNCSNLETLSVADNNL-
TGTLKPLIGKLQKLRILQVSYNSLTGPIP  496
            +  N  TG +P D+ N + LE + +++N L  G     I  + ++  ++++   +
+GP P
Sbjct  265  LENNRITG-
LPADLRNLTRLEWIDLSNNPLMNGDAVKGIATMPQISQIELNSCKISGPFP  323

Query  497
REIGNLKDLNILYLHSNGFTGRIPREMSNLTLLQGLRMYSNDLEGPIPEEMFDMKLLSVL  556
              +  L                P  +S+  +   L + +N + G IP  +  +
L  L
```

```
Sbjct  324  TWVSRLPQ---------------PDTISD-
EVTPSLDLGNNAITGTIPSAVGKLTNLEYL  367

Query  557  DLSNNKFSGQIPALFSKLESLTYLSLQGNKFNGSIPASLKSLSLLNTFDIS  607
            DL NNK +G IPA F++L+SL   ++  N+ +G IP     +    TFD S
Sbjct  368  DLQNNKLTGSIPASFAQLQSLRGFNVSYNQLSGKIP----QVKPFTTFDKS  414


 Score =   105 bits (262),  Expect = 3e-28, Method: Compositional
matrix adjust.
 Identities = 83/270 (31%), Positives = 135/270 (50%), Gaps = 17/270
(6%)

Query  411  KLLDLSHNQMTGEIPRGFG---
RMNLTFISIGRNHFTGEIPDDIFNCSNLETLSVADNNL  467
            ++L L +N  +G IP  FG   R+    +S+  +  +G IP+      +L+ L +
N+L
Sbjct  139  QVLILDNNDFSGHIPSSFGNFRRLQRLCLSVNPS-
ISGPIPESFGQLGSLQLLDLRSNSL  197

Query  468
TGTLKPLIGKLQKLRILQVSYNSLTGPIPREIGNLKDLNILYLHSNGFTGRIPREMS-NL  526
            +G L    GK+  L  L +  N +TGPIP   G L  L    L  N  +GRIP
L
Sbjct  198
SGPLPASFGKMSNLMNLHLFGNKITGPIPPSFGLLSKLYNADLGGNQISGRIPDAFGYGL  257

Query  527  TLLQGLRMYSNDLEGPIPEEMFDMKLLSVLDLSNNKF-
SGQIPALFSKLESLTYLSLQGN  585
            T L  L + +N + G +P ++ ++  L  +DLSNN   +G      + +  ++ +
L
Sbjct  258  TSLINLYLENNRITG-
LPADLRNLTRLEWIDLSNNPLMNGDAVKGIATMPQISQIELNSC  316

Query  586  KFNGSIPASLKSLSLLNT--------
FDISDNLLTGTIPGELLASLKNMQLYLNFSNNLL  637
            K +G  P  +  L   +T         D+ +N +TGTIP   +  L N++ YL+
NN L
Sbjct  317  KISGPFPTWVSRLPQPDTISDEVTPSLDLGNNAITGTIP-SAVGKLTNLE-
YLDLQNNKL  374

Query  638  TGTIPKELGKLEMVQEIDLSNNLFSGSIPR  667
            TG+IP    +L+ ++  ++S N  SG IP+
Sbjct  375  TGSIPASFAQLQSLRGFNVSYNQLSGKIPQ  404


 Score = 69.7 bits (169),  Expect = 2e-16, Method: Compositional
matrix adjust.
 Identities = 46/115 (40%), Positives = 65/115 (57%), Gaps = 2/115
(2%)

Query  652  QEIDLSNNLFSGSIPRSLQACKNVFTLDFSQN-
NLSGHIPDEVFQGMDMIISLNLSRNSF  710
            Q + L NN FSG IP S    + +  L  S N ++SG IP E F  +  +  L+L
```

NS
Sbjct 139 QVLILDNNDFSGHIPSSFGNFRRLQRLCLSVNPSISGPIP-
ESFGQLGSLQLLDLRSNSL 197

Query 711 SGEIPQSFGNMTHLVSLDLSSNNLTGEIPESLANLSTLKHLKLASNNLKGHVPES
765
            SG +P SFG M++L++L L N +TG IP S   LS L + L N + G +P++
Sbjct 198 SGPLPASFGKMSNLMNLHLFGNKITGPIPPSFGLLSKLYNADLGGNQISGRIPDA
252

Lambda   K   H   a   alpha
   0.317   0.136   0.385   0.792   4.96

Gapped
Lambda   K   H   a   alpha   sigma
   0.267   0.0410   0.140   1.90   42.6   43.6

Effective search space used: 447930

Query= sp|Q9FL28|FLS2_ARATH_LRR_receptor-like_serine/threonine-
protein_kinase_FLS2_OS=Arabidopsis_thaliana_GN=FLS2_PE=1_SV=1

Length=1173

Subject= 214979-125_3_ORF2
>sp|Q9FL28|FLS2_ARATH_LRR_receptor-like_serine/threonine-
protein_kinase_FLS2_OS=Arabidopsis_thaliana_GN=FLS2_PE=1_SV=1|||1e-
50

Length=429

 Score =   160 bits (406), Expect = 3e-46, Method: Compositional
matrix adjust.
 Identities = 119/336 (35%), Positives = 177/336 (53%), Gaps =
18/336 (5%)

Query 33   ALKSFKNGISNDPLGVLSDWTIIGSLRH--CNWTGITCD-
STGHVVSVSLLEKQLEGVLS 89
            AL SFK IS+DP   L+DW   + H C+W G+TCD +T VV + L +
+G L+
Sbjct 70   ALLSFKASISSDPNKALADWK---
ASHHNCCDWNGVTCDGATSRVVRLKLANQNFKGNLA 126

Query 90   PAIANLTYLQVLDLTSNSFTGKIPAEIGKLTELNQLILYLN-
YFSGSIPSGIWELKNIFY 148
             +++ L+ LQVL L +N F+G IP+ G   L +L L +N   SG IP   +L
++
Sbjct 127

```
SSLSALSSLQVLILDNNDFSGHIPSSFGNFRRLQRLCLSVNPSISGPIPESFGQLGSLQL  186

Query  149  
LDLRNNLLSGDVPEEICKTSSLVLIGFDYNNLTGKIPECLGDLVHLQMFVAAGNHLTGSI  208
            LDLR+N LSG +P    K S+L+ +    N +TG IP   G L  L      GN +
+G I
Sbjct  187  
LDLRSNSLSGPLPASFGKMSNLMNLHLFGNKITGPIPPSFGLLSKLYNADLGGNQISGRI  246

Query  209  PVSIGT-LANLTDLDLSGNQLTGKIPRDFGNLLNLQSLVLTEN-
LLEGDIPAEIGNCSSL  266
            P + G  L +L +L L  N++TG +P D  NL  L+ + L+ N L+ GD    I
+
Sbjct  247  PDAFGYGLTSLINLYLENNRITG-
LPADLRNLTRLEWIDLSNNPLMNGDAVKGIATMPQI  305

Query  267  VQLELYDNQLTGKIPAELGNLVQ--------
LQALRIYKNKLTSSIPSSLFRLTQLTHLG  318
             Q+EL   +++G  P  +  L Q          +L +  N +T +IPS++ +LT L
+L
Sbjct  306  
SQIELNSCKISGPFPTWVSRLPQPDTISDEVTPSLDLGNNAITGTIPSAVGKLTNLEYLD  365

Query  319  LSENHLVGPISEEIGFLESLEVLTLHSNNFTGEFPQ  354
            L  N L G I      L+SL    +  N  +G+ PQ
Sbjct  366  LQNNKLTGSIPASFAQLQSLRGFNVSYNQLSGKIPQ  401


 Score =   153 bits (387),  Expect = 1e-43, Method: Compositional
matrix adjust.
 Identities = 112/299 (37%), Positives = 156/299 (52%), Gaps =
20/299 (7%)

Query  507  ILYLHSNGFTGRIPREMSNLTLLQGLRMYSN-
DLEGPIPEEMFDMKLLSVLDLSNNKFSG  565
            +L L +N F+G IP    N   LQ L +  N  + GPIPE    +  L +LDL +N
SG
Sbjct  137  
VLILDNNDFSGHIPSSFGNFRRLQRLCLSVNPSISGPIPESFGQLGSLQLLDLRSNSLSG  196

Query  566  QIPALFSKLESLTYLSLQGNKFNGSIPASLKSLSLLNTFDISDNLLTGTIP---
GELLAS  622
             +PA F K+ +L  L L GNK  G IP S   LS L   D+  N ++G IP   G
L S
Sbjct  197  
PLPASFGKMSNLMNLHLFGNKITGPIPPSFGLLSKLYNADLGGNQISGRIPDAFGYGLTS  256

Query  623  LKNMQLYLNFSNNLLTGTIPKELGKLEMVQEIDLSNN-
LFSGSIPRSLQACKNVFTLDFS  681
            L N  LYL   NN +TG +P +L  L  ++ IDLSNN L +G   + +     +
++ +
Sbjct  257  LIN--LYL--ENNRITG-
LPADLRNLTRLEWIDLSNNPLMNGDAVKGIATMPQISQIELN  311
```

```
Query  682  QNNLSGHIPDEVFQ-------
GMDMIISLNLSRNSFSGEIPQSFGNMTHLVSLDLSSNNL  734
               +SG  P  V +         ++  SL+L  N+ +G IP + G +T+L  LDL
+N L
Sbjct  312
SCKISGPFPTWVSRLPQPDTISDEVTPSLDLGNNAITGTIPSAVGKLTNLEYLDLQNNKL  371

Query  735  TGEIPESLANLSTLKHLKLASNNLKGHVPESGVFKNINASDLM-
GNTDLCGSKKPLKPC  792
            TG IP S A L +L+   ++ N L G +P+   F   + S    GN  LCGS  PL
PC
Sbjct  372  TGSIPASFAQLQSLRGFNVSYNQLSGKIPQVKPFTTFDKSSYTPGNPGLCGS--
PLTPC  428


 Score =   129 bits (324),  Expect = 9e-36, Method: Compositional
matrix adjust.
 Identities = 103/309 (33%), Positives = 159/309 (51%), Gaps =
21/309 (7%)

Query  312
TQLTHLGLSENHLVGPISEEIGFLESLEVLTLHSNNFTGEFPQSITNLRNLTVLTVGFN-  370
            +++  L L+  +  G ++  +  L SL+VL L +N+F+G  P S  N R L  L +
N
Sbjct  109
SRVVRLKLANQNFKGNLASSLSALSSLQVLILDNNDFSGHIPSSFGNFRRLQRLCLSVNP  168

Query  371
NISGELPADLGLLTNLRNLSAHDNLLTGPIPSSISNCTGLKLLDLSHNQMTGEIPRGFGR  430
            +ISG +P   G L +L+ L    N L+GP+P+S    + L  L L  N++TG IP
FG
Sbjct  169
SISGPIPESFGQLGSLQLLDLRSNSLSGPLPASFGKMSNLMNLHLFGNKITGPIPPSFGL  228

Query  431  MNLTF-ISIGRNHFTGEIPDDI-
FNCSNLETLSVADNNLTGTLKPLIGKLQKLRILQVSY  488
            ++  +   +G N  +G IPD   +  ++L  L + +N +TG L   +  L +L  +
+S
Sbjct  229  LSKLYNADLGGNQISGRIPDAFGYGLTSLINLYLENNRITG-
LPADLRNLTRLEWIDLSN  287

Query  489  NSL-
TGPIPREIGNLKDLNILYLHSNGFTGRIPREMSNLTLLQGLRMYSNDLEGPIPEEM  547
            N L  G   + I  +  ++ + L+S   +G  P  +S L               P
P+ +
Sbjct  288  NPLMNGDAVKGIATMPQISQIELNSCKISGPFPTWVSRL---------------
PQPDTI  332

Query  548
FDMKLLSVLDLSNNKFSGQIPALFSKLESLTYLSLQGNKFNGSIPASLKSLSLLNTFDIS  607
             D ++   LDL NN  +G IP+   KL +L YL LQ NK  GSIPAS   L  L
F++S
Sbjct  333  SD-
EVTPSLDLGNNAITGTIPSAVGKLTNLEYLDLQNNKLTGSIPASFAQLQSLRGFNVS  391
```

```
Query  608  DNLLTGTIP  616
             N L+G IP
Sbjct  392  YNQLSGKIP  400


 Score =   118 bits (296),  Expect = 3e-32, Method: Compositional
matrix adjust.
 Identities = 107/351 (30%), Positives = 165/351 (47%), Gaps =
49/351 (14%)

Query  261
GNCSSLVQLELYDNQLTGKIPAELGNLVQLQALRIYKNKLTSSIPSSLFRLTQLTHLGLS  320
            G  S +V+L+L +    G + + L  L  LQ L +  N  +  IPSS     +L
L LS
Sbjct  106
GATSRVVRLKLANQNFKGNLASSLSALSSLQVLILDNNDFSGHIPSSFGNFRRLQRLCLS  165

Query  321  EN-
HLVGPISEEIGFLESLEVLTLHSNNFTGEFPQSITNLRNLTVLTVGFNNISGELPAD  379
             N  + GPI E  G L SL++L L SN+                         +SG
LPA
Sbjct  166  VNPSISGPIPESFGQLGSLQLLDLRSNS------------------------
LSGPLPAS  201

Query  380  LGLLTNLRNLSAHDNLLTGPIPSSISNCTGLKLLDLSHNQMTGEIPR--
GFGRMNLTFIS  437
             G ++NL NL    N +TGPIP S    + L   DL  NQ++G IP   G+G  +L
+
Sbjct  202
FGKMSNLMNLHLFGNKITGPIPPSFGLLSKLYNADLGGNQISGRIPDAFGYGLTSLINLY  261

Query  438  IGRNHFTGEIPDDIFNCSNLETLSVADNNL-
TGTLKPLIGKLQKLRILQVSYNSLTGPIP  496
            +  N  TG +P D+ N + LE + +++N L  G     I  + ++  ++++   +
+GP P
Sbjct  262  LENNRITG-
LPADLRNLTRLEWIDLSNNPLMNGDAVKGIATMPQISQIELNSCKISGPFP  320

Query  497
REIGNLKDLNILYLHSNGFTGRIPREMSNLTLLQGLRMYSNDLEGPIPEEMFDMKLLSVL  556
             +  L                P  +S+  +   L + +N + G IP  +  +
L  L
Sbjct  321  TWVSRLPQ---------------PDTISD-
EVTPSLDLGNNAITGTIPSAVGKLTNLEYL  364

Query  557  DLSNNKFSGQIPALFSKLESLTYLSLQGNKFNGSIPASLKSLSLLNTFDIS  607
            DL NNK +G IPA F++L+SL   ++  N+ +G IP     +    TFD S
Sbjct  365  DLQNNKLTGSIPASFAQLQSLRGFNVSYNQLSGKIP----QVKPFTTFDKS  411


 Score =   105 bits (261),  Expect = 4e-28, Method: Compositional
matrix adjust.
 Identities = 83/270 (31%), Positives = 135/270 (50%), Gaps = 17/270
```

(6%)

```
Query  411  KLLDLSHNQMTGEIPRGFG---
RMNLTFISIGRNHFTGEIPDDIFNCSNLETLSVADNNL  467
            ++L L +N  +G IP  FG   R+    +S+  +  +G IP+      +L+ L +
N+L
Sbjct  136  QVLILDNNDFSGHIPSSFGNFRRLQRLCLSVNPS-
ISGPIPESFGQLGSLQLLDLRSNSL  194

Query  468
TGTLKPLIGKLQKLRILQVSYNSLTGPIPREIGNLKDLNILYLHSNGFTGRIPREMS-NL  526
            +G L    GK+  L  L +  N +TGPIP   G L  L    L  N  +GRIP
L
Sbjct  195
SGPLPASFGKMSNLMNLHLFGNKITGPIPPSFGLLSKLYNADLGGNQISGRIPDAFGYGL  254

Query  527  TLLQGLRMYSNDLEGPIPEEMFDMKLLSVLDLSNNKF-
SGQIPALFSKLESLTYLSLQGN  585
            T L  L + +N + G +P ++ ++  L  +DLSNN   +G      + +  ++ +
L
Sbjct  255  TSLINLYLENNRITG-
LPADLRNLTRLEWIDLSNNPLMNGDAVKGIATMPQISQIELNSC  313

Query  586  KFNGSIPASLKSLSLLNT--------
FDISDNLLTGTIPGELLASLKNMQLYLNFSNNLL  637
            K +G  P  +  L   +T         D+ +N +TGTIP   +  L N++ YL+
NN L
Sbjct  314  KISGPFPTWVSRLPQPDTISDEVTPSLDLGNNAITGTIP-SAVGKLTNLE-
YLDLQNNKL  371

Query  638  TGTIPKELGKLEMVQEIDLSNNLFSGSIPR  667
            TG+IP    +L+ ++  ++S N  SG IP+
Sbjct  372  TGSIPASFAQLQSLRGFNVSYNQLSGKIPQ  401


 Score = 69.3 bits (168),  Expect = 2e-16, Method: Compositional
matrix adjust.
 Identities = 46/115 (40%), Positives = 65/115 (57%), Gaps = 2/115
(2%)

Query  652  QEIDLSNNLFSGSIPRSLQACKNVFTLDFSQN-
NLSGHIPDEVFQGMDMIISLNLSRNSF  710
            Q + L NN FSG IP S    + +  L  S N ++SG IP E F  +  +  L+L
NS
Sbjct  136  QVLILDNNDFSGHIPSSFGNFRRLQRLCLSVNPSISGPIP-
ESFGQLGSLQLLDLRSNSL  194

Query  711  SGEIPQSFGNMTHLVSLDLSSNNLTGEIPESLANLSTLKHLKLASNNLKGHVPES
765
            SG +P SFG M++L++L L  N +TG IP S   LS L +  L  N + G +P++
Sbjct  195  SGPLPASFGKMSNLMNLHLFGNKITGPIPPSFGLLSKLYNADLGGNQISGRIPDA
249
```

```
Lambda      K        H        a         alpha
   0.317    0.136    0.385    0.792     4.96

Gapped
Lambda      K        H        a         alpha    sigma
   0.267   0.0410    0.140     1.90     42.6     43.6

Effective search space used: 447930




Query= sp|Q9FL28|FLS2_ARATH_LRR_receptor-like_serine/threonine-
protein_kinase_FLS2_OS=Arabidopsis_thaliana_GN=FLS2_PE=1_SV=1

Length=1173

Subject= 215063-125_3_ORF2
>sp|Q9FL28|FLS2_ARATH_LRR_receptor-like_serine/threonine-
protein_kinase_FLS2_OS=Arabidopsis_thaliana_GN=FLS2_PE=1_SV=1|||2e-
50

Length=429


 Score =   160 bits (406),  Expect = 3e-46, Method: Compositional
matrix adjust.
 Identities = 119/336 (35%), Positives = 177/336 (53%), Gaps =
18/336 (5%)

Query  33   ALKSFKNGISNDPLGVLSDWTIIGSLRH--CNWTGITCD-
STGHVVSVSLLEKQLEGVLS  89
            AL SFK  IS+DP   L+DW    +  H  C+W G+TCD +T  VV + L  +
+G L+
Sbjct  70   ALLSFKASISSDPNKALADWK---
ASHHNCCDWNGVTCDGATSRVVRLKLANQNFKGNLA  126

Query  90   PAIANLTYLQVLDLTSNSFTGKIPAEIGKLTELNQLILYLN-
YFSGSIPSGIWELKNIFY  148
             +++ L+ LQVL L +N F+G IP+  G    L +L L +N   SG IP    +L
++
Sbjct  127
SSLSALSSLQVLILDNNDFSGHIPSSFGNFRRLQRLCLSVNPSISGPIPESFGQLGSLQL  186

Query  149
LDLRNNLLSGDVPEEICKTSSLVLIGFDYNNLTGKIPECLGDLVHLQMFVAAGNHLTGSI  208
            LDLR+N LSG +P    K S+L+ +    N +TG IP   G L  L      GN +
+G I
Sbjct  187
LDLRSNSLSGPLPASFGKMSNLMNLHLFGNKITGPIPPSFGLLSKLYNADLGGNQISGRI  246

Query  209  PVSIGT-LANLTDLDLSGNQLTGKIPRDFGNLLNLQSLVLTEN-
LLEGDIPAEIGNCSSL  266
```

```
            P + G  L +L +L L  N++TG +P D  NL  L+ + L+ N L+ GD    I
+
Sbjct  247  PDAFGYGLTSLINLYLENNRITG-
LPADLRNLTRLEWIDLSNNPLMNGDAVKGIATMPQI  305

Query  267  VQLELYDNQLTGKIPAELGNLVQ--------
LQALRIYKNKLTSSIPSSLFRLTQLTHLG  318
             Q+EL   +++G  P  +  L Q          +L +  N +T +IPS++ +LT L
+L
Sbjct  306
SQIELNSCKISGPFPTWVSRLPQPDTISDEVTPSLDLGNNAITGTIPSAVGKLTNLEYLD  365

Query  319  LSENHLVGPISEEIGFLESLEVLTLHSNNFTGEFPQ  354
            L  N L G I      L+SL    +  N  +G+ PQ
Sbjct  366  LQNNKLTGSIPASFAQLQSLRGFNVSYNQLSGKIPQ  401


 Score =   149 bits (375),  Expect = 3e-42, Method: Compositional
matrix adjust.
 Identities = 111/299 (37%), Positives = 155/299 (52%), Gaps =
20/299 (7%)

Query  507  ILYLHSNGFTGRIPREMSNLTLLQGLRMYSN-
DLEGPIPEEMFDMKLLSVLDLSNNKFSG  565
            +L L +N F+G IP    N   LQ L +  N  + GPIPE    +  L +LDL +N
SG
Sbjct  137
VLILDNNDFSGHIPSSFGNFRRLQRLCLSVNPSISGPIPESFGQLGSLQLLDLRSNSLSG  196

Query  566  QIPALFSKLESLTYLSLQGNKFNGSIPASLKSLSLLNTFDISDNLLTGTIP---
GELLAS  622
             +PA F K+ +L  L L GNK  G IP S   LS L   D+  N ++G IP   G
L S
Sbjct  197
PLPASFGKMSNLMNLHLFGNKITGPIPPSFGLLSKLYNADLGGNQISGRIPDAFGYGLTS  256

Query  623  LKNMQLYLNFSNNLLTGTIPKELGKLEMVQEIDLSNN-
LFSGSIPRSLQACKNVFTLDFS  681
            L N  LYL   NN +TG +P +L  L  ++ IDLSNN L +G   + +     +
++ +
Sbjct  257  LIN--LYL--ENNRITG-
LPADLRNLTRLEWIDLSNNPLMNGDAVKGIATMPQISQIELN  311

Query  682  QNNLSGHIPDEVFQ-------
GMDMIISLNLSRNSFSGEIPQSFGNMTHLVSLDLSSNNL  734
               +SG  P  V +         ++  SL+L  N+ +G IP + G +T+L  LDL
+N L
Sbjct  312
SCKISGPFPTWVSRLPQPDTISDEVTPSLDLGNNAITGTIPSAVGKLTNLEYLDLQNNKL  371

Query  735  TGEIPESLANLSTLKHLKLASNNLKGHVPESGVFKNINASDLM-
GNTDLCGSKKPLKPC  792
            TG IP S A L +L+   ++ N L G +P+   F   + S    GN  LCGS  PL
C
```

```
Sbjct  372  TGSIPASFAQLQSLRGFNVSYNQLSGKIPQVKPFTTFDKSSYTPGNPGLCGS--
PLTLC  428


 Score =   129 bits (324),  Expect = 8e-36, Method: Compositional
matrix adjust.
 Identities = 103/309 (33%), Positives = 159/309 (51%), Gaps =
21/309 (7%)

Query  312
TQLTHLGLSENHLVGPISEEIGFLESLEVLTLHSNNFTGEFPQSITNLRNLTVLTVGFN-  370
            +++  L L+  +  G ++  +  L SL+VL L +N+F+G  P S  N R L  L +
N
Sbjct  109
SRVVRLKLANQNFKGNLASSLSALSSLQVLILDNNDFSGHIPSSFGNFRRLQRLCLSVNP  168

Query  371
NISGELPADLGLLTNLRNLSAHDNLLTGPIPSSISNCTGLKLLDLSHNQMTGEIPRGFGR  430
            +ISG +P   G L +L+ L    N L+GP+P+S    + L  L L  N++TG IP
FG
Sbjct  169
SISGPIPESFGQLGSLQLLDLRSNSLSGPLPASFGKMSNLMNLHLFGNKITGPIPPSFGL  228

Query  431  MNLTF-ISIGRNHFTGEIPDDI-
FNCSNLETLSVADNNLTGTLKPLIGKLQKLRILQVSY  488
            ++  +   +G N  +G IPD   +  ++L  L + +N +TG L   +  L +L  +
+S
Sbjct  229  LSKLYNADLGGNQISGRIPDAFGYGLTSLINLYLENNRITG-
LPADLRNLTRLEWIDLSN  287

Query  489  NSL-
TGPIPREIGNLKDLNILYLHSNGFTGRIPREMSNLTLLQGLRMYSNDLEGPIPEEM  547
            N L  G   + I  +  ++ + L+S   +G  P  +S L               P
P+ +
Sbjct  288  NPLMNGDAVKGIATMPQISQIELNSCKISGPFPTWVSRL---------------
PQPDTI  332

Query  548
FDMKLLSVLDLSNNKFSGQIPALFSKLESLTYLSLQGNKFNGSIPASLKSLSLLNTFDIS  607
             D ++   LDL NN  +G IP+   KL +L YL LQ NK  GSIPAS   L  L
F++S
Sbjct  333  SD-
EVTPSLDLGNNAITGTIPSAVGKLTNLEYLDLQNNKLTGSIPASFAQLQSLRGFNVS  391

Query  608  DNLLTGTIP  616
             N L+G IP
Sbjct  392  YNQLSGKIP  400


 Score =   118 bits (296),  Expect = 2e-32, Method: Compositional
matrix adjust.
 Identities = 107/351 (30%), Positives = 165/351 (47%), Gaps =
49/351 (14%)
```

Query 261
GNCSSLVQLELYDNQLTGKIPAELGNLVQLQALRIYKNKLTSSIPSSLFRLTQLTHLGLS 320
G S +V+L+L + G + + L L LQ L + N + IPSS +L
L LS
Sbjct 106
GATSRVVRLKLANQNFKGNLASSLSALSSLQVLILDNNDFSGHIPSSFGNFRRLQRLCLS 165

Query 321 EN-
HLVGPISEEIGFLESLEVLTLHSNNFTGEFPQSITNLRNLTVLTVGFNNISGELPAD 379
N + GPI E G L SL++L L SN+ +SG
LPA
Sbjct 166 VNPSISGPIPESFGQLGSLQLLDLRSNS------------------------
LSGPLPAS 201

Query 380 LGLLTNLRNLSAHDNLLTGPIPSSISNCTGLKLLDLSHNQMTGEIPR--
GFGRMNLTFIS 437
G ++NL NL N +TGPIP S + L DL NQ++G IP G+G +L
+
Sbjct 202
FGKMSNLMNLHLFGNKITGPIPPSFGLLSKLYNADLGGNQISGRIPDAFGYGLTSLINLY 261

Query 438 IGRNHFTGEIPDDIFNCSNLETLSVADNNL-
TGTLKPLIGKLQKLRILQVSYNSLTGPIP 496
+ N TG +P D+ N + LE + +++N L G I + ++ ++++ +
+GP P
Sbjct 262 LENNRITG-
LPADLRNLTRLEWIDLSNNPLMNGDAVKGIATMPQISQIELNSCKISGPFP 320

Query 497
REIGNLKDLNILYLHSNGFTGRIPREMSNLTLLQGLRMYSNDLEGPIPEEMFDMKLLSVL 556
+ L P +S+ + L + +N + G IP + +
L L
Sbjct 321 TWVSRLPQ---------------PDTISD-
EVTPSLDLGNNAITGTIPSAVGKLTNLEYL 364

Query 557 DLSNNKFSGQIPALFSKLESLTYLSLQGNKFNGSIPASLKSLSLLNTFDIS 607
DL NNK +G IPA F++L+SL ++ N+ +G IP + TFD S
Sbjct 365 DLQNNKLTGSIPASFAQLQSLRGFNVSYNQLSGKIP----QVKPFTTFDKS 411

Score = 105 bits (261), Expect = 4e-28, Method: Compositional matrix adjust.
Identities = 83/270 (31%), Positives = 135/270 (50%), Gaps = 17/270 (6%)

Query 411 KLLDLSHNQMTGEIPRGFG---
RMNLTFISIGRNHFTGEIPDDIFNCSNLETLSVADNNL 467
++L L +N +G IP FG R+ +S+ + +G IP+ +L+ L +
N+L
Sbjct 136 QVLILDNNDFSGHIPSSFGNFRRLQRLCLSVNPS-
ISGPIPESFGQLGSLQLLDLRSNSL 194

Query 468
TGTLKPLIGKLQKLRILQVSYNSLTGPIPREIGNLKDLNILYLHSNGFTGRIPREMS-NL 526

```
            +G L    GK+  L  L +  N +TGPIP   G L  L    L  N  +GRIP
L
Sbjct  195
SGPLPASFGKMSNLMNLHLFGNKITGPIPPSFGLLSKLYNADLGGNQISGRIPDAFGYGL  254

Query  527  TLLQGLRMYSNDLEGPIPEEMFDMKLLSVLDLSNNKF-
SGQIPALFSKLESLTYLSLQGN  585
            T L  L + +N + G +P ++ ++  L  +DLSNN   +G      + +  ++ +
L
Sbjct  255  TSLINLYLENNRITG-
LPADLRNLTRLEWIDLSNNPLMNGDAVKGIATMPQISQIELNSC  313

Query  586  KFNGSIPASLKSLSLLNT--------
FDISDNLLTGTIPGELLASLKNMQLYLNFSNNLL  637
            K +G  P  +  L   +T         D+ +N +TGTIP   +  L N++ YL+
NN L
Sbjct  314  KISGPFPTWVSRLPQPDTISDEVTPSLDLGNNAITGTIP-SAVGKLTNLE-
YLDLQNNKL  371

Query  638  TGTIPKELGKLEMVQEIDLSNNLFSGSIPR  667
            TG+IP    +L+ ++  ++S N  SG IP+
Sbjct  372  TGSIPASFAQLQSLRGFNVSYNQLSGKIPQ  401


 Score = 69.3 bits (168),  Expect = 2e-16, Method: Compositional
matrix adjust.
 Identities = 46/115 (40%), Positives = 65/115 (57%), Gaps = 2/115
(2%)

Query  652  QEIDLSNNLFSGSIPRSLQACKNVFTLDFSQN-
NLSGHIPDEVFQGMDMIISLNLSRNSF  710
            Q + L NN FSG IP S    + +  L  S N ++SG IP E F  +  +  L+L
NS
Sbjct  136  QVLILDNNDFSGHIPSSFGNFRRLQRLCLSVNPSISGPIP-
ESFGQLGSLQLLDLRSNSL  194

Query  711  SGEIPQSFGNMTHLVSLDLSSNNLTGEIPESLANLSTLKHLKLASNNLKGHVPES
765
            SG +P SFG M++L++L L  N +TG IP S   LS L +  L  N + G +P++
Sbjct  195  SGPLPASFGKMSNLMNLHLFGNKITGPIPPSFGLLSKLYNADLGGNQISGRIPDA
249



Lambda      K        H        a         alpha
   0.317    0.136    0.385    0.792     4.96

Gapped
Lambda      K        H        a         alpha    sigma
   0.267   0.0410    0.140     1.90     42.6     43.6

Effective search space used: 447930
```

Matrix: BLOSUM62
Gap Penalties: Existence: 11, Extension: 1
Neighboring words threshold: 11
Window for multiple hits: 40

Query= sp|Q9FM19|HIR1_ARATH_Hypersensitive-induced_response_protein_1_OS=Arabidopsis_thaliana_GN=HIR1_PE=1_SV=1

Length=286

Subject= 67936-306_5_ORF1
>sp|Q9FM19|HIR1_ARATH_Hypersensitive-induced_response_protein_1_OS=Arabidopsis_thaliana_GN=HIR1_PE=1_SV=1
|||3e-164

Length=329

Score = 451 bits (1161), Expect = 3e-164, Method: Compositional matrix adjust.
Identities = 210/285 (74%), Positives = 250/285 (88%), Gaps = 0/285 (0%)

```
Query  1
MGNLFCCVQVDQSTVAIKETFGKFEDVLEPGCHFLPWCLGSQVAGYLSLRVQQLDVRCET  60
            MG LFCC+QV+Q++V IKE +GK+++ LEPGCH + WC GS VAGYL++R+QQLDV
CET
Sbjct  38
MGQLFCCLQVNQASVGIKERWGKYDEALEPGCHCVNWCFGSNVAGYLTMRIQQLDVHCET  97

Query  61
KTKDNVFVNVVASIQYRALANKANDAYYKLSNTRGQIQAYVFDVIRASVPKLLLDDVFEQ  120
            KT+DNVFV +VASIQYR +   A +A+YKLS  + QIQAYVFDV+R+SVP+L
LDDVFEQ
Sbjct  98
KTRDNVFVTMVASIQYRVVNENAKEAFYKLSRPQEQIQAYVFDVVRSSVPRLNLDDVFEQ  157

Query  121
KNDIAKAVEEELEKAMSAYGYEIVQTLIVDIEPDEHVKRAMNEINAAARMRLAANEKAEA  180
            KNDIAKAVE+ELEKAM  YGYEIVQTLIVDI PD+ VKRAMNEINAAARMRLA
NEKAEA
Sbjct  158
KNDIAKAVEDELEKAMRTYGYEIVQTLIVDIIPDQTVKRAMNEINAAARMRLATNEKAEA  217

Query  181
EKILQIKRAEGEAESKYLSGLGIARQRQAIVDGLRDSVLGFAVNVPGTTAKDVMDMVLVT  240
            EKILQ+KRAE EAESK+LSG GIARQRQAIVDGLR+SVLGF+ +VPGT+
KDVMDMV++T
```

```
Sbjct  218
EKILQVKRAEAEAESKFLSGQGIARQRQAIVDGLRESVLGFSHDVPGTSPKDVMDMVMLT  277

Query  241  QYFDTMKEIGASSKSSAVFIPHGPGAVRDVASQIRDGLLQGSSAN  285
            QYFDT+K+IGA SKSS VF+PHGPG V ++A QIR+G LQG++ +
Sbjct  278  QYFDTLKDIGAHSKSSVVFVPHGPGVVGNIADQIRNGWLQGAAGS  322



Lambda      K        H        a         alpha
   0.318    0.133    0.370    0.792     4.96

Gapped
Lambda      K        H        a         alpha    sigma
   0.267   0.0410    0.140     1.90     42.6     43.6

Effective search space used: 78218




Query= sp|Q9FM19|HIR1_ARATH_Hypersensitive-
induced_response_protein_1_OS=Arabidopsis_thaliana_GN=HIR1_PE=1_SV=1

Length=286

Subject= 68039-306_5_ORF2
>sp|Q9FM19|HIR1_ARATH_Hypersensitive-
induced_response_protein_1_OS=Arabidopsis_thaliana_GN=HIR1_PE=1_SV=1
|||7e-165

Length=297


 Score =   452 bits (1162),  Expect = 7e-165, Method: Compositional
matrix adjust.
 Identities = 210/285 (74%), Positives = 250/285 (88%), Gaps = 0/285
(0%)

Query  1
MGNLFCCVQVDQSTVAIKETFGKFEDVLEPGCHFLPWCLGSQVAGYLSLRVQQLDVRCET  60
            MG LFCC+QV+Q++V IKE +GK+++ LEPGCH + WC GS VAGYL++R+QQLDV
CET
Sbjct  6
MGQLFCCLQVNQASVGIKERWGKYDEALEPGCHCVNWCFGSNVAGYLTMRIQQLDVHCET  65

Query  61
KTKDNVFVNVVASIQYRALANKANDAYYKLSNTRGQIQAYVFDVIRASVPKLLLDDVFEQ  120
            KT+DNVFV +VASIQYR +   A +A+YKLS  + QIQAYVFDV+R+SVP+L
LDDVFEQ
Sbjct  66
KTRDNVFVTMVASIQYRVVNENAKEAFYKLSRPQEQIQAYVFDVVRSSVPRLNLDDVFEQ  125

Query  121
```

```
KNDIAKAVEEELEKAMSAYGYEIVQTLIVDIEPDEHVKRAMNEINAAARMRLAANEKAEA  180
            KNDIAKAVE+ELEKAM  YGYEIVQTLIVDI PD+ VKRAMNEINAAARMRLA
NEKAEA
Sbjct  126
KNDIAKAVEDELEKAMRTYGYEIVQTLIVDIIPDQTVKRAMNEINAAARMRLATNEKAEA  185

Query  181
EKILQIKRAEGEAESKYLSGLGIARQRQAIVDGLRDSVLGFAVNVPGTTAKDVMDMVLVT  240
            EKILQ+KRAE EAESK+LSG GIARQRQAIVDGLR+SVLGF+ +VPGT+
KDVMDMV++T
Sbjct  186
EKILQVKRAEAEAESKFLSGQGIARQRQAIVDGLRESVLGFSHDVPGTSPKDVMDMVMLT  245

Query  241  QYFDTMKEIGASSKSSAVFIPHGPGAVRDVASQIRDGLLQGSSAN  285
            QYFDT+K+IGA SKSS VF+PHGPG V ++A QIR+G LQG++ +
Sbjct  246  QYFDTLKDIGAHSKSSVVFVPHGPGVVGNIADQIRNGWLQGAAGS  290



Lambda      K        H        a         alpha
   0.318    0.133    0.370    0.792     4.96

Gapped
Lambda      K        H        a         alpha    sigma
   0.267   0.0410    0.140     1.90     42.6     43.6

Effective search space used: 78218




Query= sp|Q9FM19|HIR1_ARATH_Hypersensitive-
induced_response_protein_1_OS=Arabidopsis_thaliana_GN=HIR1_PE=1_SV=1

Length=286

Subject= 68096-306_5_ORF2
>sp|Q9FM19|HIR1_ARATH_Hypersensitive-
induced_response_protein_1_OS=Arabidopsis_thaliana_GN=HIR1_PE=1_SV=1
|||8e-165

Length=297


 Score =   452 bits (1162),  Expect = 8e-165, Method: Compositional
matrix adjust.
 Identities = 210/285 (74%), Positives = 250/285 (88%), Gaps = 0/285
(0%)

Query  1
MGNLFCCVQVDQSTVAIKETFGKFEDVLEPGCHFLPWCLGSQVAGYLSLRVQQLDVRCET  60
            MG LFCC+QV+Q++V IKE +GK+++ LEPGCH + WC GS VAGYL++R+QQLDV
CET
Sbjct  6
```

```
MGQLFCCLQVNQASVGIKERWGKYDEALEPGCHCVNWCFGSNVAGYLTMRIQQLDVHCET  65

Query  61
KTKDNVFVNVVASIQYRALANKANDAYYKLSNTRGQIQAYVFDVIRASVPKLLLDDVFEQ  120
            KT+DNVFV +VASIQYR +   A +A+YKLS  + QIQAYVFDV+R+SVP+L
LDDVFEQ
Sbjct  66
KTRDNVFVTMVASIQYRVVNENAKEAFYKLSRPQEQIQAYVFDVVRSSVPRLNLDDVFEQ  125

Query  121
KNDIAKAVEEELEKAMSAYGYEIVQTLIVDIEPDEHVKRAMNEINAAARMRLAANEKAEA  180
            KNDIAKAVE+ELEKAM  YGYEIVQTLIVDI PD+ VKRAMNEINAAARMRLA
NEKAEA
Sbjct  126
KNDIAKAVEDELEKAMRTYGYEIVQTLIVDIIPDQTVKRAMNEINAAARMRLATNEKAEA  185

Query  181
EKILQIKRAEGEAESKYLSGLGIARQRQAIVDGLRDSVLGFAVNVPGTTAKDVMDMVLVT  240
            EKILQ+KRAE EAESK+LSG GIARQRQAIVDGLR+SVLGF+ +VPGT+
KDVMDMV++T
Sbjct  186
EKILQVKRAEAEAESKFLSGQGIARQRQAIVDGLRESVLGFSHDVPGTSPKDVMDMVMLT  245

Query  241  QYFDTMKEIGASSKSSAVFIPHGPGAVRDVASQIRDGLLQGSSAN  285
            QYFDT+K+IGA SKSS VF+PHGPG V ++A QIR+G LQG++ +
Sbjct  246  QYFDTLKDIGAHSKSSVVFVPHGPGVVGNIADQIRNGWLQGAAGS  290



Lambda      K        H        a         alpha
   0.318    0.133    0.370    0.792     4.96

Gapped
Lambda      K        H        a         alpha    sigma
   0.267   0.0410    0.140     1.90     42.6     43.6

Effective search space used: 78218




Query= sp|Q9FM19|HIR1_ARATH_Hypersensitive-
induced_response_protein_1_OS=Arabidopsis_thaliana_GN=HIR1_PE=1_SV=1

Length=286

Subject= 68364-306_5_ORF1
>sp|Q9FM19|HIR1_ARATH_Hypersensitive-
induced_response_protein_1_OS=Arabidopsis_thaliana_GN=HIR1_PE=1_SV=1
|||4e-164

Length=329
```

Score = 451 bits (1161), Expect = 4e-164, Method: Compositional matrix adjust.
Identities = 210/285 (74%), Positives = 250/285 (88%), Gaps = 0/285 (0%)

```
Query  1
MGNLFCCVQVDQSTVAIKETFGKFEDVLEPGCHFLPWCLGSQVAGYLSLRVQQLDVRCET  60
            MG LFCC+QV+Q++V IKE +GK+++ LEPGCH + WC GS VAGYL++R+QQLDV
CET
Sbjct  38
MGQLFCCLQVNQASVGIKERWGKYDEALEPGCHCVNWCFGSNVAGYLTMRIQQLDVHCET  97

Query  61
KTKDNVFVNVVASIQYRALANKANDAYYKLSNTRGQIQAYVFDVIRASVPKLLLDDVFEQ  120
            KT+DNVFV +VASIQYR +   A +A+YKLS  + QIQAYVFDV+R+SVP+L
LDDVFEQ
Sbjct  98
KTRDNVFVTMVASIQYRVVNENAKEAFYKLSRPQEQIQAYVFDVVRSSVPRLNLDDVFEQ  157

Query  121
KNDIAKAVEEELEKAMSAYGYEIVQTLIVDIEPDEHVKRAMNEINAAARMRLAANEKAEA  180
            KNDIAKAVE+ELEKAM  YGYEIVQTLIVDI PD+ VKRAMNEINAAARMRLA
NEKAEA
Sbjct  158
KNDIAKAVEDELEKAMRTYGYEIVQTLIVDIIPDQTVKRAMNEINAAARMRLATNEKAEA  217

Query  181
EKILQIKRAEGEAESKYLSGLGIARQRQAIVDGLRDSVLGFAVNVPGTTAKDVMDMVLVT  240
            EKILQ+KRAE EAESK+LSG GIARQRQAIVDGLR+SVLGF+ +VPGT+
KDVMDMV++T
Sbjct  218
EKILQVKRAEAEAESKFLSGQGIARQRQAIVDGLRESVLGFSHDVPGTSPKDVMDMVMLT  277

Query  241  QYFDTMKEIGASSKSSAVFIPHGPGAVRDVASQIRDGLLQGSSAN  285
            QYFDT+K+IGA SKSS VF+PHGPG V ++A QIR+G LQG++ +
Sbjct  278  QYFDTLKDIGAHSKSSVVFVPHGPGVVGNIADQIRNGWLQGAAGS  322



Lambda      K        H        a         alpha
   0.318    0.133    0.370    0.792     4.96

Gapped
Lambda      K        H        a         alpha    sigma
   0.267   0.0410    0.140     1.90     42.6     43.6

Effective search space used: 78218



Matrix: BLOSUM62
Gap Penalties: Existence: 11, Extension: 1
Neighboring words threshold: 11
```

```
Window for multiple hits: 40





Query= sp|Q9FRL8|DHAR2_ARATH_Glutathione_S-
transferase_DHAR2_OS=Arabidopsis_thaliana_GN=DHAR2_PE=1_SV=1

Length=213

Subject= 50095-359_2_ORF2
>sp|Q9FRL8|DHAR2_ARATH_Glutathione_S-
transferase_DHAR2_OS=Arabidopsis_thaliana_GN=DHAR2_PE=1_SV=1|||3e-23

Length=478


 Score = 84.3 bits (207),  Expect = 3e-23, Method: Compositional
matrix adjust.
 Identities = 60/213 (28%), Positives = 108/213 (51%), Gaps = 26/213
(12%)

Query  20   CPFSQRVLLTLEEKKLPYKTHLINVS---
DKPQWFLDISPEGKVPVVKLDGKWVADSDVI  76
            CP+ +RV L LEEK++PY    IN++    KP W++ + P G +P + + GK +
+S  I
Sbjct  125
CPYCERVWLQLEEKRIPYNVEKINMNCYGTKPDWYIKLIPSGLLPAINITGKVIPESLDI  184

Query  77   VGLLEEKYPE-----PSLKTPPEFASVG------SKIFGAFVTFLKSKDAND-
GSEKALV  124
            + LLE+ +P+     PS  +P + A+V        ++ GA++  L+    N   S
++
Sbjct  185
MMLLEDAFPQHNPLLPSKSSPEKLAAVNPLLKLERRLAGAWLGCLRGGMWNSIDSFNRVL  244

Query  125
DELEALENHLKTHSGPFVAGEKITAVDLSLAPKLYHLEVALGHYKNWSVPESLTSVR---  181
            D+++A    LK   GP+  G++++ VD   AP L  +  ++ +   W  PE   S
R
Sbjct  245  DDVDA---SLKKFGGPYFLGQEVSMVDAVYAPFLERIAASVPY---
WLGPEVRGSGRWPA  298

Query  182  --NYAKALFSRESFENTKAKKEIVVAGWESKVN  212
               +  A+ SR S++  K+    +    E ++
Sbjct  299  LDAWFDAMDSRPSYQAMKSDDFTITHTLEPQIG  331


 Score = 13.9 bits (24),  Expect = 5.3, Method: Compositional matrix
adjust.
 Identities = 5/15 (33%), Positives = 8/15 (53%), Gaps = 0/15 (0%)

Query  54   ISPEGKVPVVKLDGK  68
```

```
            + P G     K++GK
Sbjct  335  VLPAGAAYRAKINGK  349


 Score = 13.1 bits (22),  Expect = 9.7, Method: Compositional matrix
adjust.
 Identities = 7/16 (44%), Positives = 8/16 (50%), Gaps = 1/16 (6%)

Query  42   INVSDKPQWFLDISPE  57
            IN  D   W L + PE
Sbjct  346  INGKDG-SWDLPLKPE  360



Lambda      K        H        a         alpha
   0.314    0.133    0.389    0.792     4.96

Gapped
Lambda      K        H        a         alpha    sigma
   0.267   0.0410    0.140     1.90     42.6     43.6

Effective search space used: 83886




Matrix: BLOSUM62
Gap Penalties: Existence: 11, Extension: 1
Neighboring words threshold: 11
Window for multiple hits: 40





Query= sp|Q9LJX0|
AB19B_ARATH_ABC_transporter_B_family_member_19_OS=Arabidop
sis_thaliana_GN=ABCB19_PE=1_SV=1

Length=1252

Subject= 390256-26_3_ORF2
>sp|Q9LJX0|AB19B_ARATH_ABC_transporter_B_family_member_19_OS=Arabido
psis_thaliana_GN=ABCB19_PE=1_SV=1|||0

Length=419


 Score =   549 bits (1414),  Expect = 0.0, Method: Compositional
matrix adjust.
 Identities = 263/402 (65%), Positives = 322/402 (80%), Gaps = 0/402
(0%)

Query  850
```

```
QQLSLKGFAGDTAKAHAKTSMIAGEGVSNIRTVAAFNAQSKILSLFCHELRVPQKRSLYR  909
             QQL + G AGD  +AHA +SMIAG+   NIRTVAAFNA+ K+LSLF   L+ P K
+S  R
Sbjct  13
QQLLVTGLAGDVNEAHAHSSMIAGDAAGNIRTVAAFNAEGKVLSLFTEALKEPAKKSFLR  72

Query  910
SQTSGFLFGLSQLALYGSEALILWYGAHLVSKGVSTFSKVIKVFVVLVITANSVAETVSL  969
                SGF  GLSQ A+Y S  L+LWYG+ LV  G S F  V KVF+VL++TA
+VAET+SL
Sbjct  73
GNISGFFLGLSQCAMYCSYGLVLWYGSVLVKDGTSKFGSVYKVFLVLMMTAFAVAETLSL  132

Query  970
APEIIRGGEAVGSVFSVLDRQTRIDPDDADADPVETIRGDIEFRHVDFAYPSRPDVMVFR  1029
              P+II+G +A+ SVF ++DRQT IDPDD  A+ V  + GD+E   V+FAYP
+RPDV VF+
Sbjct  133
TPDIIKGSDAIKSVFDIVDRQTSIDPDDQSAEIVSKVHGDVELVDVEFAYPTRPDVTVFK  192

Query  1030
DFNLRIRAGHSQALVGASGSGKSSVIAMIERFYDPLAGKVMIDGKDIRRLNLKSLRLKIG  1089
               NL ++AG S ALVGASGSGKSSVI ++ERFYDP+ G V +DGKDIR LNL
+SLR  IG
Sbjct  193
SLNLSVKAGTSLALVGASGSGKSSVIGLVERFYDPVRGSVTVDGKDIRCLNLRSLRQHIG  252

Query  1090
LVQQEPALFAATIFDNIAYGKDGATESEVIDAARAANAHGFISGLPEGYKTPVGERGVQL  1149
             LVQQEPALFA TIF+NI YG++GATE+EV DAA++ANAHGFIS LP GY+T
VGERGVQL
Sbjct  253
LVQQEPALFATTIFENILYGREGATEAEVEDAAQSANAHGFISALPGGYRTEVGERGVQL  312

Query  1150
SGGQKQRIAIARAVLKNPTVLLLDEATSALDAESECVLQEALERLMRGRTTVVVAHRLST  1209
             SGGQKQR+AIARAVLKNPT+LLLDEATSALDAESE V+Q AL++LM GRTT
+VVAHRL+T
Sbjct  313
SGGQKQRVAIARAVLKNPTILLLDEATSALDAESEKVVQSALDKLMEGRTTIVVAHRLTT  372

Query  1210  IRGVDCIGVIQDGRIVEQGSHSELVSRPEGAYSRLLQLQTHR  1251
             IR  D I V+Q+GRI+EQG+HSEL+S+  G+Y+RL+ +Q  R
Sbjct  373   IRNADIIAVVQEGRILEQGTHSELMSKQNGSYARLVNIQRAR  414


 Score =   386 bits (991),  Expect = 5e-126, Method: Compositional
matrix adjust.
 Identities = 189/395 (48%), Positives = 271/395 (69%), Gaps = 1/395
(0%)

Query  209
LTGITSKSRESYANAGVIAEQAIAQVRTVYSYVGESKALNAYSDAIQYTLKLGYKAGMAK  268
           +TG+     E++A++ +IA  A   +RTV ++  E K L+ +++A++   K  +
```

```
G
Sbjct  17
VTGLAGDVNEAHAHSSMIAGDAAGNIRTVAAFNAEGKVLSLFTEALKEPAKKSFLRGNIS  76

Query  269
GLGLGCTYGIACMSWALVFWYAGVFIRNGQTDGGKAFTAIFSAIVGGMSLGQSFSNLGAF  328
            G  LG +      S+ LV WY  V +++G +  G  +      ++   ++ ++ S
Sbjct  77
GFFLGLSQCAMYCSYGLVLWYGSVLVKDGTSKFGSVYKVFLVLMMTAFAVAETLSLTPDI  136

Query  329
SKGKAAGYKLMEIINQRPTIIQDPLDGKCLDQVHGNIEFKDVTFSYPSRPDVMIFRNFNI  388
             KG  A   + +I++++ +I  D    + + +VHG++E  DV F+YP+RPDV +F+
+ N+
Sbjct  137
IKGSDAIKSVFDIVDRQTSIDPDDQSAEIVSKVHGDVELVDVEFAYPTRPDVTVFKSLNL  196

Query  389
FFPSGKTVAVVGGSGSGKSTVVSLIERFYDPNSGQILLDGVEIKTLQLKFLREQIGLVNQ  448
               +G ++A+VG SGSGKS+V+ L+ERFYDP  G + +DG +I+ L L+ LR+
IGLV Q
Sbjct  197
SVKAGTSLALVGASGSGKSSVIGLVERFYDPVRGSVTVDGKDIRCLNLRSLRQHIGLVQQ  256

Query  449
EPALFATTILENILYGKPDATMVEVEAAASAANAHSFITLLPKGYDTQVGERGVQLSGGQ  508
            EPALFATTI ENILYG+  AT  EVE AA +ANAH FI+ LP GY T
+VGERGVQLSGGQ
Sbjct  257
EPALFATTIFENILYGREGATEAEVEDAAQSANAHGFISALPGGYRTEVGERGVQLSGGQ  316

Query  509
KQRIAIARAMLKDPKILLLDEATSALDASSESIVQEALDRVMVGRTTVVVAHRLCTIRNV  568
            KQR+AIARA+LK+P ILLLDEATSALDA SE +VQ ALD++M GRTT+VVAHRL
TIRN
Sbjct  317
KQRVAIARAVLKNPTILLLDEATSALDAESEKVVQSALDKLMEGRTTIVVAHRLTTIRNA  376

Query  569  DSIAVIQQGQVVETGTHEELIAK-SGAYASLIRFQ  602
            D IAV+Q+G+++E GTH EL++K +G+YA L+  Q
Sbjct  377  DIIAVVQEGRILEQGTHSELMSKQNGSYARLVNIQ  411



Lambda      K        H        a         alpha
   0.321    0.136    0.384    0.792     4.96

Gapped
Lambda      K        H        a         alpha    sigma
   0.267   0.0410    0.140     1.90     42.6     43.6

Effective search space used: 459348
```

Matrix: BLOSUM62
Gap Penalties: Existence: 11, Extension: 1
Neighboring words threshold: 11
Window for multiple hits: 40

Query= sp|Q9LMF1|U85A3_ARATH_UDP-glycosyltransferase_85A3_OS=Arabidopsis_thaliana_GN=UGT85A3_PE=2_SV=2

Length=488

Subject= 230624-113_2_ORF1
>sp|Q9LMF1|U85A3_ARATH_UDP-glycosyltransferase_85A3_OS=Arabidopsis_thaliana_GN=UGT85A3_PE=2_SV=2|||7e-90

Length=482

Score = 275 bits (702), Expect = 7e-90, Method: Compositional matrix adjust.
Identities = 177/493 (36%), Positives = 264/493 (54%), Gaps = 42/493 (9%)

```
Query  1    MGSRFVSNE-QKPHVVCVPYPAQGHINPMMKVAKLLHVKGFHVTFVNTVYNHNRLLRSRG  59
            MG++  S+  ++PHVV +P PA+GH+ PMM  +K+L  +G  +TFVN+ +N ++L+
Sbjct  15   MGAQASSSPMRRPHVVVLPLPARGHVTPMMHFSKMLAAQGVSITFVNSEHN-DKLVTETE  73

Query  60   ANALDGLPSFQFESIPDGLPETGVDATQDIPALSESTTKNCLVPFKKLLQRIVTREDVPP  119
             AL  L   + E++ DGL      A  D+ A  E+        F+ L+  +  +E P
Sbjct  74   REALQRL-GIRLEALRDGL-----SADLDVQAFCETMLSGMPQLFEALIACLFRQEPTP-  126

Query  120  VSCIVSDGSMSFTLDVAEELGVPEIHFWTTSACGFMAYLHFYLFIEKG--LCPVKDASCL  177
             SCI++D  ++F   VA + G+P    WT SA  F     F+  +  G    P +
Sbjct  127  -SCILTDFELTFAPSVASKFGLPLASLWTKSAASFA----FFFMVANGSYTGPSQGEPH-  180

Query  178  TKEYLDTVIDWIPSMNNVKLKDIPSFIRTTNPNDIMLNFVVREACRTKRASAIILNTFDD  237
                       IP    +K +D+ +F R  + ++ M  FV     R  R+  I +NTF +
Sbjct  181  -----------
```

```
IPGAEFLKEEDMNTFARCYDQSNFMFRFVTGGFDRLDRSQWIFINTFQE  229

Query  238  LEHDIIQSM--QSILPPVYPIGPLHLLV-NREIEEDSEIGRMG--
SNLWKEETECLGWLN  292
            LE + ++S+  +  + P+ P+ P  LL  + E E +S   +    S +W EE  CL
WL+
Sbjct  230
LECETLKSLSEKGHILPIGPLLPSSLLSHDNENEANSNYPQTNYVSTIWAEEEGCLTWLD  289

Query  293
TKSRNSVVYVNFGSITIMTTAQLLEFAWGLAATGKEFLWVMRPDSVAGEEAVIPKEFLAE  352
                 SVVYV+FGS+ ++T+ Q+ E A GL A+G  FLWV RP S+ GE     +
FL
Sbjct  290
QFEPKSVVYVSFGSMAMVTSQQIEELALGLEASGYPFLWVARPGSIYGESPNFNENFLER  349

Query  353
TADRRMLTSWCPQEKVLSHPAVGGFLTHCGWNSTLESLSCGVPMVCWPFFAEQQTNCKFS  412
             +R    SW PQ KVLSH AVGGF TH GWNST+E +S GVPM+ WP+FA+Q
+CK
Sbjct  350
VKERAFFVSWAPQLKVLSHKAVGGFFTHGGWNSTVEGISTGVPMLGWPYFADQPMDCKCI  409

Query  413  CDEWEVGIEIGGD-----
VKRGEVEAVVRELMDGEKGKKMREKAVEWRRLAEKATKLPCG  467
            + W++G+ +        V R  +E  V+ELM  +K  K   KA EW  LA+KA+
L  G
Sbjct  410  EEGWKIGLRLRERESEILVPRNVIETKVKELMCQDKFLK---KAYEWSTLAKKAS-
LQGG  465

Query  468  SSVINFETIVNKV  480
            SS  N +  V+ +
Sbjct  466  SSFKNIQGFVDTL  478



Lambda      K        H        a         alpha
   0.320    0.136    0.423    0.792     4.96

Gapped
Lambda      K        H        a         alpha    sigma
   0.267   0.0410    0.140     1.90     42.6     43.6

Effective search space used: 203392




Query= sp|Q9LMF1|U85A3_ARATH_UDP-
glycosyltransferase_85A3_OS=Arabidopsis_thaliana_GN=UGT85A3_PE=2_SV=
2

Length=488
```

Subject= 98919-251_6_ORF1
>sp|Q9LMF1|U85A3_ARATH_UDP-
glycosyltransferase_85A3_OS=Arabidopsis_thaliana_GN=UGT85A3_PE=2_SV=
2|||3e-90

Length=479

Score = 275 bits (704), Expect = 3e-90, Method: Compositional
matrix adjust.
Identities = 174/488 (36%), Positives = 261/488 (53%), Gaps =
35/488 (7%)

```
Query  1    MGSRFVSNE-
QKPHVVCVPYPAQGHINPMMKVAKLLHVKGFHVTFVNTVYNHNRLLRSRG  59
            MG++  S+  ++PHVV +P PA+GH+ PMM  +K+L  +G  +TFVN+ +N ++L+
Sbjct  15   MGAQASSSPMRRPHVVVLPLPARGHVTPMMHFSKMLAAQGVSITFVNSEHN-
DKLVTETE  73

Query  60
ANALDGLPSFQFESIPDGLPETGVDATQDIPALSESTTKNCLVPFKKLLQRIVTREDVPP  119
              AL  L   + E++ DGL      A  D+ A  E+        F+ L+  +  +E
P
Sbjct  74   REALQRL-GIRLEALRDGL-----
SADLDVQAFCETMLSGMPQLFEALIACLFRQEPTP-  126

Query  120
VSCIVSDGSMSFTLDVAEELGVPEIHFWTTSACGFMAYLHFYLFIEKGLCPVKDASCLTK  179
             SCI++D  ++F   VA + G+P    WT SA  F + L            V + S
Sbjct  127  -SCILTDFELTFAPSVASKFGLPLASLWTKSAASFASLLM-----------
VANGSYTGP  174

Query  180
EYLDTVIDWIPSMNNVKLKDIPSFIRTTNPNDIMLNFVVREACRTKRASAIILNTFDDLE  239
               +     IP    +K +D+ +F R  + ++ M  FV     R  R+  I +NTF
+LE
Sbjct  175  SQGEP---
HIPGAEFLKEEDMNTFARCYDQSNFMFRFVTGGFDRLDRSQWIFINTFQELE  231

Query  240  HDIIQSM--
QSILPPVYPIGPLHLLVNREIEEDSEIGRMGSNLWKEETECLGWLNTKSRN  297
             + ++S+  +  + P+ P+ P  LL +    E +      S +W  E +CL WL+
Sbjct  232
CETLKSLSEKGHILPIGPLLPSSLLSHDNENEANSQTNYISTIWAAEEDCLTWLDQFEPK  291

Query  298
SVVYVNFGSITIMTTAQLLEFAWGLAATGKEFLWVMRPDSVAGEEAVIPKEFLAETADRR  357
            SVVYV+FGS+ ++T+ Q+ E A GL A+G  FLWV RP S+ GE     + FL
+R
Sbjct  292
SVVYVSFGSMAMVTSQQIEELALGLEASGYPFLWVARPGSIYGESPNFNENFLERVKERA  351

Query  358
MLTSWCPQEKVLSHPAVGGFLTHCGWNSTLESLSCGVPMVCWPFFAEQQTNCKFSCDEWE  417
```

```
              SW PQ KVLSH AVGGF TH GWNST+E +S GVPM+ WP+FA+Q  +CK
+ W+
Sbjct  352
FFVSWAPQLKVLSHKAVGGFFTHGGWNSTVEGISTGVPMLGWPYFADQPMDCKCIEEGWK  411

Query  418  VGIEIGGD-----
VKRGEVEAVVRELMDGEKGKKMREKAVEWRRLAEKATKLPCGSSVIN  472
            +G+ +        V R  +E  V+ELM  +K  K   KA EW  LA+KA+ L
GSS  N
Sbjct  412  IGLRLRERESEILVPRNVIETKVKELMCQDKFLK---KAYEWSTLAKKAS-
LQGGSSFKN  467

Query  473  FETIVNKV  480
             +  V+ +
Sbjct  468  IQGFVDTL  475



Lambda      K        H        a         alpha
   0.320    0.136    0.423    0.792     4.96

Gapped
Lambda      K        H        a         alpha    sigma
   0.267   0.0410    0.140     1.90     42.6     43.6

Effective search space used: 203392



Matrix: BLOSUM62
Gap Penalties: Existence: 11, Extension: 1
Neighboring words threshold: 11
Window for multiple hits: 40



Query= sp|Q9LVQ0|
PME31_ARATH_Pectinesterase_31_OS=Arabidopsis_thaliana_GN=P
ME31_PE=1_SV=1

Length=317

Subject= 294633-71_1_ORF2
>sp|Q9LVQ0|PME31_ARATH_Pectinesterase_31_OS=Arabidopsis_thaliana_GN=
PME31_PE=1_SV=1|||2e-158

Length=325


 Score =   438 bits (1126),  Expect = 2e-158, Method: Compositional
matrix adjust.
```

```
 Identities = 196/312 (63%), Positives = 246/312 (79%), Gaps = 3/312
(1%)

Query  5
RMVRVSQDGSGDYCSVQDAIDSVPLGNTCRTVIRLSPGIYRQPVYVPKRKNFITFAGISP  64
            ++++V+QDGSGDY +VQDA+D++P  NT R +I ++PG Y+QPVYVPK K  I+
G  P
Sbjct  9
KILKVAQDGSGDYKTVQDAVDAIPANNTQRVLIHVAPGTYQQPVYVPKNKKMISLLGDHP  68

Query  65
EITVLTWNNTASKIEHHQASRVIGTGTFGCGSVIVEGEDFIAENITFENSAPEGSGQAVA  124
            E T+LTW NTA+ I+H Q   VIGTGTF  G+VIVEGE F+A+ ITFEN+AP
+GSGQAVA
Sbjct  69
ETTILTWANTATSIKHPQVVSVIGTGTFASGTVIVEGEGFVAQGITFENAAPQGSGQAVA  128

Query  125
IRVTADRCAFYNCRFLGWQDTLYLHHGKQYLKDCYIEGSVDFIFGNSTALLEHCHIHCKS  184
            IRVTADR AFYNCRFLGWQDT YLHHG+ Y++DCYIEGSVDFIFGN+T
LLEHCHIHCKS
Sbjct  129
IRVTADRSAFYNCRFLGWQDTAYLHHGRIYIRDCYIEGSVDFIFGNATVLLEHCHIHCKS  188

Query  185  QGFITAQSRKSSQESTGYVFLRCVITGNGQSG-
YMYLGRPWGPFGRVVLAYTYMDACIRN  243
             GFITAQ R ++ +STGYVFLRC ITG+G S  Y YLGRPW P+ RV+ AYTYMD
CI+
Sbjct  189
SGFITAQQRTTATDSTGYVFLRCTITGSGSSNPYAYLGRPWAPYARVIYAYTYMDVCIKP  248

Query  244
VGWHNWGNAENERSACFYEYRCFGPGSCSSERVPWSRELMDDEAGHFVHHSFVDPEQDRP  303
             GW+NW N  NE++A +YEYRC+GPGS +S+RV W++++ DD+ G FV  SF+D +
+
Sbjct  249
EGWNNWDNLNNEKTAEYYEYRCYGPGSDTSKRVAWAKQMKDDKVGEFVSLSFIDVQSN--  306

Query  304  WLCLRMGVKTPY  315
            WL      + P+
Sbjct  307  WLSTAKIAEVPF  318



Lambda      K        H        a         alpha
   0.322    0.137    0.450    0.792     4.96

Gapped
Lambda      K        H        a         alpha    sigma
   0.267   0.0410    0.140     1.90     42.6     43.6

Effective search space used: 85833
```

```
Matrix: BLOSUM62
Gap Penalties: Existence: 11, Extension: 1
Neighboring words threshold: 11
Window for multiple hits: 40




Query= sp|Q9M2W2|GSTL2_ARATH_Glutathione_S-transferase_L2,
_chloroplastic_OS=Arabidopsis_thaliana_GN=GSTL2_PE=2_SV=1

Length=292

Subject= 169514-167_2_ORF1
>sp|Q9M2W2|GSTL2_ARATH_Glutathione_S-transferase_L2,
_chloroplastic_OS=Arabidopsis_thaliana_GN=GSTL2_PE=2_SV=1|||4e-33

Length=145


 Score =   108 bits (269),  Expect = 2e-33, Method: Compositional
matrix adjust.
 Identities = 56/141 (40%), Positives = 79/141 (56%), Gaps = 8/141
(6%)

Query  152  IDTNFEGPSLTPDGLEKQVVADELLSYTDSFSKAVRSTLNGTDTNAADVA------
FDYI  205
            ID NFEGP L P   +K+    ELL ++D   K +  +    D +A
D +
Sbjct  1
IDQNFEGPCLFPKEPKKEEATKELLKFSDEVIKQLFGSFRNKDADAVYAEEHIGPILDRL  60

Query  206  EQALSKF-
NEGPFFLGQFSLVDVAYAPFIERFRLILSDVMNVDITSGRPNLALWIQEMNK  264
            E    KF +EGPFFLGQFS VD+ YAPF E+F ++  D++N +I  GRP L  W +
M
Sbjct  61
EAVFGKFESEGPFFLGQFSAVDITYAPFFEKFEILAPDLLNYNIYKGRPKLENWFKAMKT  120

Query  265  IEAYTET-RQDPQELVERYKR  284
            ++AYT     +P+E+ E YK+
Sbjct  121  VDAYTSAINYNPKEVAEGYKK  141



Lambda      K        H        a         alpha
   0.317    0.133    0.381    0.792     4.96

Gapped
Lambda      K        H        a         alpha    sigma
   0.267   0.0410    0.140     1.90     42.6     43.6
```

Effective search space used: 33604

Matrix: BLOSUM62
Gap Penalties: Existence: 11, Extension: 1
Neighboring words threshold: 11
Window for multiple hits: 40

Query= sp|Q9S9U6|1A111_ARATH_1-aminocyclopropane-1-carboxylate_synthase_11_OS=Arabidopsis_thaliana_GN=ACS11_PE=1_SV=1

Length=460

Subject= 254191-97_6_ORF2
>sp|Q9S9U6|1A111_ARATH_1-aminocyclopropane-1-carboxylate_synthase_11_OS=Arabidopsis_thaliana_GN=ACS11_PE=1_SV=1|||1e-87

Length=471

Score =   260 bits (664), Expect = 8e-85, Method: Compositional matrix adjust.
Identities = 145/396 (37%), Positives = 227/396 (57%), Gaps = 20/396 (5%)

```
Query  37
GIVQMGLAENQLSFDLIEKWLEEHPEVLGLKKNDESVFRQLALFQDYHGLPAFKDAMAKF  96
            G   M  AE+ LSF+LI K ++E  EV             + L+ ++ G      A
+AK
Sbjct  72   GYFLMAQAESMLSFELIHKKMKECREVP----------
LTVGLYSNFRGGERLCQAIAKM  121

Query  97
MGKIRENKVKFDTNKMVLTAGSTSANETLMFCLANPGDAFLIPAPYYPGFDRDLKWRTGV  156
            M +     VK D++ + +++G T+  +   F   + GD  LIPAPY+P FD D+
R  V
Sbjct  122  MERTFMG-
VKVDSSNICISSGVTAVLDLFFFATCDSGDGCLIPAPYFPAFDNDMTIRNEV  180

Query  157
EIVPIHCVSSNGYKITEDALEDAYERALKHNLNVKGVLITNPSNPLGTSTTREELDLLLT  216
             +P+    +N Y  T + +E A   A +  + VK +L+TNP NPLGT      L
LL
Sbjct  181
IPLPVQPKDTNTYIPTAEEMEAAVVTAAQRGIKVKVLLVTNPGNPLGTLYPESTLKELLV  240
```

```
Query  217  FTSTKKIHMVSDEIYSGTVF--DSPEFTSVLEVAKD---KNMG----
LDGKIHVVYSLSK  267
            +   + +H++SDEIY+ + F   + EF S+ +VA++   +N+     +   +H  Y
+SK
Sbjct  241
WALNRGLHVLSDEIYANSKFRPSADEFVSMEKVAQNAVAENLVSPEIVKKSLHTAYGMSK  300

Query  268
DLGLPGFRVGLIYSNNEKVVSAATKMSSFGLISSQTQHLLANLLSDERFTTNYLEENKKR  327
            D G+ GFRVG +++ +E++++    M  F  +S+ TQH LA +L DE F  +Y+
ENK+R
Sbjct  301
DFGMNGFRVGCLHTKSEELLTFWQNMGMFAAVSNDTQHALAIMLEDENFVDSYVSENKRR  360

Query  328
LRERKDRLVSGLKEAGISCLKSNAGLFCWVDLRHLLKSNTFEAEHSLWTKIVCEVGLNIS  387
            LR+  + L S L+ A I  + + A +FCW+DLR LL + TFEAE+SLW +I+ E
+ ++
Sbjct  361
LRKSYELLTSHLEAAQIKYMPACAAMFCWLDLRSLLSAPTFEAENSLWKEIMDESKIVLT  420

Query  388  PGSSCHCDEPGWFRVCFANMSDQTMEVAMDRVKGFV  423
            PG SCH  EPG+FR C+A +S + +E A   +  FV
Sbjct  421  PGESCHYAEPGFFRACYALVSPEGLEAACKSLSDFV  456



Lambda      K        H        a         alpha
   0.317    0.133    0.397    0.792     4.96

Gapped
Lambda      K        H        a         alpha    sigma
   0.267   0.0410    0.140     1.90     42.6     43.6

Effective search space used: 187026




Query= sp|Q9S9U6|1A111_ARATH_1-aminocyclopropane-1-
carboxylate_synthase_11_OS=Arabidopsis_thaliana_GN=ACS11_PE=1_SV=1

Length=460

Subject= 254437-97_6_ORF2
>sp|Q9S9U6|1A111_ARATH_1-aminocyclopropane-1-
carboxylate_synthase_11_OS=Arabidopsis_thaliana_GN=ACS11_PE=1_SV=1||
|8e-88

Length=466


 Score =   259 bits (663),  Expect = 9e-85, Method: Compositional
matrix adjust.
```

```
 Identities = 145/396 (37%), Positives = 227/396 (57%), Gaps = 
20/396 (5%)

Query  37   
GIVQMGLAENQLSFDLIEKWLEEHPEVLGLKKNDESVFRQLALFQDYHGLPAFKDAMAKF  96
            G   M  AE+ LSF+LI K ++E  EV             + L+ ++ G      A 
+AK
Sbjct  67   GYFLMAQAESMLSFELIHKKMKECREVP----------
LTVGLYSNFRGGERLCQAIAKM  116

Query  97   
MGKIRENKVKFDTNKMVLTAGSTSANETLMFCLANPGDAFLIPAPYYPGFDRDLKWRTGV  156
            M +     VK D++ + +++G T+  +   F   + GD  LIPAPY+P FD D+  
R  V
Sbjct  117  MERTFMG-
VKVDSSNICISSGVTAVLDLFFFATCDSGDGCLIPAPYFPAFDNDMTIRNEV  175

Query  157  
EIVPIHCVSSNGYKITEDALEDAYERALKHNLNVKGVLITNPSNPLGTSTTREELDLLLT  216
              +P+    +N Y  T + +E A   A +  + VK +L+TNP NPLGT      L  
LL
Sbjct  176  
IPLPVQPKDTNTYIPTAEEMEAAVVTAAQRGIKVKVLLVTNPGNPLGTLYPESTLKELLV  235

Query  217  FTSTKKIHMVSDEIYSGTVF--DSPEFTSVLEVAKD---KNMG----
LDGKIHVVYSLSK  267
            +   + +H++SDEIY+ + F   + EF S+ +VA++   +N+     +   +H  Y 
+SK
Sbjct  236  
WALNRGLHVLSDEIYANSKFRPSADEFVSMEKVAQNAVAENLVSPEIVKKSLHTAYGMSK  295

Query  268  
DLGLPGFRVGLIYSNNEKVVSAATKMSSFGLISSQTQHLLANLLSDERFTTNYLEENKKR  327
            D G+ GFRVG +++ +E++++    M  F  +S+ TQH LA +L DE F  +Y+
ENK+R
Sbjct  296  
DFGMNGFRVGCLHTKSEELLTFWQNMGMFAAVSNDTQHALAIMLEDENFVDSYVSENKRR  355

Query  328  
LRERKDRLVSGLKEAGISCLKSNAGLFCWVDLRHLLKSNTFEAEHSLWTKIVCEVGLNIS  387
            LR+  + L S L+ A I  + + A +FCW+DLR LL + TFEAE+SLW +I+ E
+ ++
Sbjct  356  
LRKSYELLTSHLEAAQIKYMPACAAMFCWLDLRSLLSAPTFEAENSLWKEIMDESKIVLT  415

Query  388  PGSSCHCDEPGWFRVCFANMSDQTMEVAMDRVKGFV  423
            PG SCH  EPG+FR C+A +S + +E A   +  FV
Sbjct  416  PGESCHYAEPGFFRACYALVSPEGLEAACKSLSDFV  451



Lambda      K        H        a         alpha
   0.317    0.133    0.397    0.792     4.96
```

```
Gapped
Lambda      K        H        a         alpha    sigma
   0.267   0.0410    0.140     1.90     42.6     43.6

Effective search space used: 187026




Query= sp|Q9S9U6|1A111_ARATH_1-aminocyclopropane-1-
carboxylate_synthase_11_OS=Arabidopsis_thaliana_GN=ACS11_PE=1_SV=1

Length=460

Subject= 66065-311_4_ORF2
>sp|Q9S9U6|1A111_ARATH_1-aminocyclopropane-1-
carboxylate_synthase_11_OS=Arabidopsis_thaliana_GN=ACS11_PE=1_SV=1||
|6e-88

Length=471


 Score =   261 bits (666),  Expect = 4e-85, Method: Compositional
matrix adjust.
 Identities = 145/396 (37%), Positives = 228/396 (58%), Gaps =
20/396 (5%)

Query  37
GIVQMGLAENQLSFDLIEKWLEEHPEVLGLKKNDESVFRQLALFQDYHGLPAFKDAMAKF  96
            G   M  AE+ LSF+LI K ++E  EV             + L+ ++ G      A
+AK
Sbjct  72   GYFLMAQAESMLSFELIHKKMKECREVP----------
LTVGLYSNFRGGERLCQAIAKM  121

Query  97
MGKIRENKVKFDTNKMVLTAGSTSANETLMFCLANPGDAFLIPAPYYPGFDRDLKWRTGV  156
            M +     VK D++ + +++G T+  +   F   + GD  LIPAPY+P FD D+
R  V
Sbjct  122  MERTFMG-
VKVDSSNICISSGVTAVLDLFFFATCDSGDGCLIPAPYFPAFDNDMTIRNEV  180

Query  157
EIVPIHCVSSNGYKITEDALEDAYERALKHNLNVKGVLITNPSNPLGTSTTREELDLLLT  216
              +P+    +N Y  T + +E A   A +  + VK +L+TNP NPLGT      L
LL
Sbjct  181
IPLPVQPKDTNTYIPTAEEMEAAVVTAAQRGIKVKVLLVTNPGNPLGTLYPESTLKELLV  240

Query  217  FTSTKKIHMVSDEIYSGTVF--DSPEFTSVLEVAKD---KNMG----
LDGKIHVVYSLSK  267
            +   + +H++SDEIY+ + F   + EF S+ +VA++   +N+     +   +H  Y
+SK
Sbjct  241
WALNRGLHVLSDEIYANSKFRPSADEFVSMEKVAQNAVAENLVSPEIVKKSLHTAYGMSK  300
```

```
Query  268
DLGLPGFRVGLIYSNNEKVVSAATKMSSFGLISSQTQHLLANLLSDERFTTNYLEENKKR  327
            D G+ GFRVG +++ +E++++    M  F  +S+ TQH LA +L DE F  +Y+
ENK+R
Sbjct  301
DFGMNGFRVGCLHTKSEELLTFWQNMGMFAAVSNDTQHALAIMLEDENFVDSYVSENKRR  360

Query  328
LRERKDRLVSGLKEAGISCLKSNAGLFCWVDLRHLLKSNTFEAEHSLWTKIVCEVGLNIS  387
            LR+  + L S L+ A I  + + A +FCW+DLR LL + TFEAE+SLW +I+ E
+ ++
Sbjct  361
LRKSYELLTSHLEAAQIKYMPACAAMFCWLDLRSLLSAPTFEAENSLWKEIMDESKIVLT  420

Query  388  PGSSCHCDEPGWFRVCFANMSDQTMEVAMDRVKGFV  423
            PG SCH  EPG+FR C+A +S + +E A + +  FV
Sbjct  421  PGESCHYAEPGFFRACYALVSPEGLEAACNSLSDFV  456



Lambda      K        H        a         alpha
   0.317    0.133    0.397    0.792     4.96

Gapped
Lambda      K        H        a         alpha    sigma
   0.267   0.0410    0.140     1.90     42.6     43.6

Effective search space used: 187026




Query= sp|Q9S9U6|1A111_ARATH_1-aminocyclopropane-1-
carboxylate_synthase_11_OS=Arabidopsis_thaliana_GN=ACS11_PE=1_SV=1

Length=460

Subject= 66095-311_4_ORF2
>sp|Q9S9U6|1A111_ARATH_1-aminocyclopropane-1-
carboxylate_synthase_11_OS=Arabidopsis_thaliana_GN=ACS11_PE=1_SV=1||
|6e-88

Length=466


 Score =   260 bits (665),  Expect = 5e-85, Method: Compositional
matrix adjust.
 Identities = 145/396 (37%), Positives = 228/396 (58%), Gaps =
20/396 (5%)

Query  37
GIVQMGLAENQLSFDLIEKWLEEHPEVLGLKKNDESVFRQLALFQDYHGLPAFKDAMAKF  96
            G   M  AE+ LSF+LI K ++E  EV             + L+ ++ G      A
```

```
+AK
Sbjct  67   GYFLMAQAESMLSFELIHKKMKECREVP----------
LTVGLYSNFRGGERLCQAIAKM  116

Query  97
MGKIRENKVKFDTNKMVLTAGSTSANETLMFCLANPGDAFLIPAPYYPGFDRDLKWRTGV  156
            M +     VK D++ + +++G T+  +   F   + GD  LIPAPY+P FD D+
R  V
Sbjct  117  MERTFMG-
VKVDSSNICISSGVTAVLDLFFFATCDSGDGCLIPAPYFPAFDNDMTIRNEV  175

Query  157
EIVPIHCVSSNGYKITEDALEDAYERALKHNLNVKGVLITNPSNPLGTSTTREELDLLLT  216
              +P+    +N Y  T + +E A   A +  + VK +L+TNP NPLGT      L
LL
Sbjct  176
IPLPVQPKDTNTYIPTAEEMEAAVVTAAQRGIKVKVLLVTNPGNPLGTLYPESTLKELLV  235

Query  217  FTSTKKIHMVSDEIYSGTVF--DSPEFTSVLEVAKD---KNMG----
LDGKIHVVYSLSK  267
            +   + +H++SDEIY+ + F   + EF S+ +VA++   +N+     +   +H  Y
+SK
Sbjct  236
WALNRGLHVLSDEIYANSKFRPSADEFVSMEKVAQNAVAENLVSPEIVKKSLHTAYGMSK  295

Query  268
DLGLPGFRVGLIYSNNEKVVSAATKMSSFGLISSQTQHLLANLLSDERFTTNYLEENKKR  327
            D G+ GFRVG +++ +E++++    M  F  +S+ TQH LA +L DE F  +Y+
ENK+R
Sbjct  296
DFGMNGFRVGCLHTKSEELLTFWQNMGMFAAVSNDTQHALAIMLEDENFVDSYVSENKRR  355

Query  328
LRERKDRLVSGLKEAGISCLKSNAGLFCWVDLRHLLKSNTFEAEHSLWTKIVCEVGLNIS  387
            LR+  + L S L+ A I  + + A +FCW+DLR LL + TFEAE+SLW +I+ E
+ ++
Sbjct  356
LRKSYELLTSHLEAAQIKYMPACAAMFCWLDLRSLLSAPTFEAENSLWKEIMDESKIVLT  415

Query  388  PGSSCHCDEPGWFRVCFANMSDQTMEVAMDRVKGFV  423
            PG SCH  EPG+FR C+A +S + +E A + +  FV
Sbjct  416  PGESCHYAEPGFFRACYALVSPEGLEAACNSLSDFV  451



Lambda      K        H        a         alpha
   0.317    0.133    0.397    0.792     4.96

Gapped
Lambda      K        H        a         alpha    sigma
   0.267   0.0410    0.140     1.90     42.6     43.6

Effective search space used: 187026
```

```
Matrix: BLOSUM62
Gap Penalties: Existence: 11, Extension: 1
Neighboring words threshold: 11
Window for multiple hits: 40




Query= sp|Q9SB48|NCPR1_ARATH_NADPH--
cytochrome_P450_reductase_1_OS=Arabidopsis_thaliana_GN=ATR1_PE=1_SV=
1

Length=692

Subject= 386069-28_3_ORF2
>sp|Q9SB48|NCPR1_ARATH_NADPH--
cytochrome_P450_reductase_1_OS=Arabidopsis_thaliana_GN=ATR1_PE=1_SV=
1|||0

Length=689


 Score =   831 bits (2146),  Expect = 0.0, Method: Compositional
matrix adjust.
 Identities = 419/653 (64%), Positives = 512/653 (78%), Gaps = 8/653
(1%)

Query  46
LWKKTTADRSGELKPLMIPKSLMAKDEDDDLDLGSGKTRVSIFFGTQTGTAEGFAKALSE  105
            +W+ +   +  E++ +  P+ L+ +  + +L+  + K +V+
+FFGTQTGTAEGFAKAL E
Sbjct  39   VWRLSGGQKKKEVEQVA-PRKLVVQVPEPELEDLTQK-
KVTVFFGTQTGTAEGFAKALGE  96

Query  106  EIKARYE-
KAAVKVIDLDDYAADDDQYEEKLKKETLAFFCVATYGDGEPTDNAARFYKWF  164
            E K RY+ +  +KV+DLDDY  DDD+YE+K KKE L  F
+ATYGDGEPTDNAARFYKWF
Sbjct  97
EAKVRYDNRVVLKVVDLDDYGTDDDEYEQKFKKEKLTLFTLATYGDGEPTDNAARFYKWF  156

Query  165  TEENE-
RDIKLQQLAYGVFALGNRQYEHFNKIGIVLDEELCKKGAKRLIEVGLGDDDQSI  223
             E  E R   L  +++GVF LGNRQYEHFN++   +DE L ++GAK+L+
GLGDDDQ I
Sbjct  157
VEGKEARGDWLSGMSFGVFGLGNRQYEHFNEVAKKVDEALIEQGAKQLVPCGLGDDDQCI  216

Query  224  EDDFNAWKESLWSELDKLLKDEDDKS--
VATPYTAVIPEYRVVTHDPRFTTQKSMESNVA  281
```

EDDF AWKE LW+ LD LL D +D + V TPYTA I EYRVV HD +
Sbjct 217
EDDFAAWKEELWAALDPLLIDVNDATPAVTTPYTAAILEYRVVFHDNETKDFEETFDPKM 276

Query 282
NGNTTIDIHHPCRVDVAVQKELHTHESDRSCIHLEFDISRTGITYETGDHVGVYAENHVE 341
NG+ D++HPCR +V V++ELHT ESDRSCIHLEFDIS TG+ Y TGDHVGVY
EN E
Sbjct 277
NGHAVHDVNHPCRANVVVRRELHTPESDRSCIHLEFDISNTGLMYGTGDHVGVYPENTSE 336

Query 342 IVEEAGKLLGHSLDLVFSIHADKEDGSPLE-
SAVPPPFPGPCTLGTGLARYADLLNPPRK 400
VEE K+LG+ LD FS+HAD EDGSP+ S++ PPFPGPC L T ++RYADLL
PPRK
Sbjct 337
NVEEVAKILGYCLDTKFSLHADDEDGSPIGGSSLVPPFPGPCDLRTAISRYADLLTPPRK 396

Query 401
SALVALAAYATEPSEAEKLKHLTSPDGKDEYSQWIVASQRSLLEVMAAFPSAKPPLGVFF 460
+AL ALAA+A++ +E ++LK L SP GKD+Y QWI A+QRSLLEVM+ FPSA
+PPLGVFF
Sbjct 397
AALAALAAHASDDTERDRLKFLASPLGKDDYQQWITANQRSLLEVMSEFPSARPPLGVFF 456

Query 461 AAIAPRLQPRYYSISSSPRLAPSRVHVTSALVYGPTPTGRIHKGVCSTWMKNAVP-
AEKS 519
AAI+PRLQ RYYSISSSPR AP R+HVT +LVYG +PTGR H+GVCSTWMKN++P
+E
Sbjct 457
AAISPRLQARYYSISSSPRFAPDRIHVTCSLVYGVSPTGRFHRGVCSTWMKNSLPVSEVG 516

Query 520
HECSGAPIFIRASNFKLPSNPSTPIVMVGPGTGLAPFRGFLQERMALKEDGEELGSSLLF 579
+CS AP+F+R SNFKLP +PS PIVMVGPGTGLAPFRGF+QER LKE G+ELG
+LLF
Sbjct 517
TKCSWAPVFVRQSNFKLPMDPSIPIVMVGPGTGLAPFRGFMQERAYLKESGKELGPALLF 576

Query 580
FGCRNRQMDFIYEDELNNFVDQGVISELIMAFSREGAQKEYVQHKMMEKAAQVWDLIKEE 639
FGCRNR++D+IYEDELN F+++G IS L +AFSREG KEYVQHKM+E+AA VW
LI
Sbjct 577
FGCRNRKLDYIYEDELNGFLEKGAISGLSVAFSREGPSKEYVQHKMIEQAATVWSLISGG 636

Query 640 GYLYVCGDAKGMARDVHRTLHTIVQEQEGVSSSEAEAIVKKLQTEGRYLRDVW
692
GYLYVCGDAKGMARDVHRTLHTI+QEQ+ V S++AEAIVK+LQTEGRYLRDVW
Sbjct 637 GYLYVCGDAKGMARDVHRTLHTIIQEQDSVDSTKAEAIVKQLQTEGRYLRDVW
689

```
Lambda      K        H        a         alpha
   0.316    0.133    0.391    0.792     4.96

Gapped
Lambda      K        H        a         alpha    sigma
   0.267   0.0410    0.140     1.90     42.6     43.6

Effective search space used: 424450
```

```
Query= sp|Q9SB48|NCPR1_ARATH_NADPH--
cytochrome_P450_reductase_1_OS=Arabidopsis_thaliana_GN=ATR1_PE=1_SV=
1

Length=692

Subject= 49433-362_6_ORF2
>sp|Q9SB48|NCPR1_ARATH_NADPH--
cytochrome_P450_reductase_1_OS=Arabidopsis_thaliana_GN=ATR1_PE=1_SV=
1|||0

Length=689


 Score =   830 bits (2143),  Expect = 0.0, Method: Compositional
matrix adjust.
 Identities = 419/653 (64%), Positives = 512/653 (78%), Gaps = 8/653
(1%)

Query  46
LWKKTTADRSGELKPLMIPKSLMAKDEDDDLDLGSGKTRVSIFFGTQTGTAEGFAKALSE  105
            +W+ +   +  E++ +  P+ L+ +  + +L+  + K +V+
+FFGTQTGTAEGFAKAL E
Sbjct  39   VWRLSGGQKKKEVEQVP-PRKLVVQVPEPELEDLTQK-
KVTVFFGTQTGTAEGFAKALGE  96

Query  106  EIKARYE-
KAAVKVIDLDDYAADDDQYEEKLKKETLAFFCVATYGDGEPTDNAARFYKWF  164
            E K RY+ +  +KV+DLDDY  DDD+YE+K KKE L  F
+ATYGDGEPTDNAARFYKWF
Sbjct  97
EAKVRYDNRVVLKVVDLDDYGTDDDEYEQKFKKEKLTLFTLATYGDGEPTDNAARFYKWF  156

Query  165  TEENE-
RDIKLQQLAYGVFALGNRQYEHFNKIGIVLDEELCKKGAKRLIEVGLGDDDQSI  223
             E  E R   L  +++GVF LGNRQYEHFN++   +DE L ++GAK+L+
GLGDDDQ I
Sbjct  157
VEGKEARGDWLSGMSFGVFGLGNRQYEHFNEVAKKVDEALIEQGAKQLVPCGLGDDDQCI  216

Query  224  EDDFNAWKESLWSELDKLLKDEDDKS--
VATPYTAVIPEYRVVTHDPRFTTQKSMESNVA  281
```

```
            EDDF AWKE LW+ LD LL D +D +  V TPYTA I EYRVV HD      +
Sbjct  217
EDDFAAWKEELWAALDPLLIDVNDATPAVTTPYTAAILEYRVVFHDNETKDFEETFDPKM  276

Query  282
NGNTTIDIHHPCRVDVAVQKELHTHESDRSCIHLEFDISRTGITYETGDHVGVYAENHVE  341
            NG+   D++HPCR +V V++ELHT ESDRSCIHLEFDIS TG+ Y TGDHVGVY
EN  E
Sbjct  277
NGHAVHDVNHPCRANVVVRRELHTPESDRSCIHLEFDISNTGLMYGTGDHVGVYPENTSE  336

Query  342  IVEEAGKLLGHSLDLVFSIHADKEDGSPLE-
SAVPPPFPGPCTLGTGLARYADLLNPPRK  400
             VEE  K+LG+ LD  FS+HAD EDGSP+  S++ PPFPGPC L T ++RYADLL
PPRK
Sbjct  337
NVEEVAKILGYCLDTKFSLHADDEDGSPIGGSSLVPPFPGPCDLRTAISRYADLLTPPRK  396

Query  401
SALVALAAYATEPSEAEKLKHLTSPDGKDEYSQWIVASQRSLLEVMAAFPSAKPPLGVFF  460
            +AL ALAA+A++ +E ++LK L SP GKD+Y QWI A+QRSLLEVM+ FPSA
+PPLGVFF
Sbjct  397
AALAALAAHASDDTERDRLKFLASPLGKDDYQQWITANQRSLLEVMSEFPSARPPLGVFF  456

Query  461  AAIAPRLQPRYYSISSSPRLAPSRVHVTSALVYGPTPTGRIHKGVCSTWMKNAVP-
AEKS  519
            AAI+PRLQ RYYSISSSPR AP R+HVT +LVYG +PTGR H+GVCSTWMKN++P
+E
Sbjct  457
AAISPRLQARYYSISSSPRFAPDRIHVTCSLVYGVSPTGRFHRGVCSTWMKNSLPVSEVG  516

Query  520
HECSGAPIFIRASNFKLPSNPSTPIVMVGPGTGLAPFRGFLQERMALKEDGEELGSSLLF  579
             +CS AP+F+R SNFKLP +PS PIVMVGPGTGLAPFRGF+QER  LKE G+ELG
+LLF
Sbjct  517
TKCSWAPVFVRQSNFKLPMDPSIPIVMVGPGTGLAPFRGFMQERAYLKESGKELGPALLF  576

Query  580
FGCRNRQMDFIYEDELNNFVDQGVISELIMAFSREGAQKEYVQHKMMEKAAQVWDLIKEE  639
            FGCRNR++D+IYEDELN F+++G IS L +AFSREG  KEYVQHKM+E+AA VW
LI
Sbjct  577
FGCRNRKLDYIYEDELNGFLEKGAISGLSVAFSREGPSKEYVQHKMIEQAATVWSLISGG  636

Query  640  GYLYVCGDAKGMARDVHRTLHTIVQEQEGVSSSEAEAIVKKLQTEGRYLRDVW
692
            GYLYVCGDAKGMARDVHRTLHTI+QEQ+ V S++AEAIVK+LQTEGRYLRDVW
Sbjct  637  GYLYVCGDAKGMARDVHRTLHTIIQEQDSVDSTKAEAIVKQLQTEGRYLRDVW
689
```

```
Lambda      K        H        a         alpha
   0.316    0.133    0.391    0.792     4.96

Gapped
Lambda      K        H        a         alpha    sigma
   0.267   0.0410    0.140     1.90     42.6     43.6

Effective search space used: 424450




Matrix: BLOSUM62
Gap Penalties: Existence: 11, Extension: 1
Neighboring words threshold: 11
Window for multiple hits: 40






Query= sp|Q9SE96|GEML1_ARATH_GEM-
like_protein_1_OS=Arabidopsis_thaliana_GN=FIP1_PE=1_SV=1

Length=259

Subject= 378787-31_3_ORF2
>sp|Q9SE96|GEML1_ARATH_GEM-
like_protein_1_OS=Arabidopsis_thaliana_GN=FIP1_PE=1_SV=1|||6e-82

Length=290


 Score =   237 bits (605),  Expect = 3e-81, Method: Compositional
matrix adjust.
 Identities = 123/264 (47%), Positives = 179/264 (68%), Gaps =
15/264 (6%)

Query  2    SGQENHDHGRISSTPAAASEPSKAAAH-----SSDYAPYPK--
LDPTDVTPPPPQPIPTG  54
            SG++ ++ GR  +      +PSK   H     ++ + P P   LD ++ +   P
TG
Sbjct  25   SGEQAYETGRWGT--YTMGKPSKPEVHPGNKQATTWEPLPGNILDQSE-
SKQAPSSSSTG  81

Query  55   AAATTMPAESNPYVSPSPAP----
RNTMDSVKDTLGKWGKMAADATKKAEDLAGNFWQHL  110
             A       +NPY+S SPAP    ++ MD + D LGKW     + +KKAE+ AGN
WQHL
Sbjct  82
YAQPPTAGTANPYISTSPAPGFAGKSPMDVITDYLGKWRLKFEEVSKKAEEAAGNMWQHL  141

Query  111
KTGPSVADAAVSRIAQGTKILAEGGYEKVFKQTFDCLPDEKLLKTYACYLSTSAGPVLGV  170
```

```
            KTGP++AD A  R++QGTK+L EGG++K++KQTF+  PDE+L K++ACYLSTS
+GPV G
Sbjct  142
KTGPNMADTAWGRLSQGTKLLTEGGFDKIYKQTFETTPDEQLRKSFACYLSTSSGPVAGT  201

Query  171  MYLSTHKLAFSSDNPLSYKEG-
EQTLWSYYKVVLPANQLKAVNPSTSRVNTSDKYIQVIS  229
            +Y+ST K+AF SD PLSY+    QT WSYYK+ +P ++L++V PS++  + S
+KYIQV +
Sbjct  202
LYISTKKIAFCSDRPLSYQPSPGQTAWSYYKLEVPLDRLQSVTPSSNPNDASEKYIQVQT  261

Query  230  IDNHEFWFMGFVTYESAVKSLQEA  253
            +D+HEFWFMGFV Y+ A+++L+ A
Sbjct  262  LDDHEFWFMGFVNYDKAMRNLEAA  285



Lambda      K        H        a         alpha
   0.311    0.126    0.376    0.792     4.96

Gapped
Lambda      K        H        a         alpha    sigma
   0.267   0.0410    0.140     1.90     42.6     43.6

Effective search space used: 62010




Matrix: BLOSUM62
Gap Penalties: Existence: 11, Extension: 1
Neighboring words threshold: 11
Window for multiple hits: 40





Query= sp|Q9SJL0|U86A1_ARATH_UDP-
glycosyltransferase_86A1_OS=Arabidopsis_thaliana_GN=UGT86A1_PE=2_SV=
1

Length=490

Subject= 324129-56_3_ORF2
>sp|Q9SJL0|U86A1_ARATH_UDP-
glycosyltransferase_86A1_OS=Arabidopsis_thaliana_GN=UGT86A1_PE=2_SV=
1|||4e-55

Length=399


 Score =   172 bits (435),  Expect = 3e-52, Method: Compositional
```

```
matrix adjust.
 Identities = 117/359 (33%), Positives = 181/359 (50%), Gaps = 
33/359 (9%)

Query  68   SSGQHDIRYTTVSDGFPLDFDRSLNHDQFFEGILHVFSAHV----
DDLIAKLSRRDDPP-  122
            SSG   +R   V DG PL+ +R L   +     + V+   V    +DLI K  
Sbjct  43   
SSGSAKLRLEVVEDGLPLEEERLLPLVERIRISVPVYQNAVKLLLEDLILKSKPSSSSAP  102

Query  123  -----
VTCLIADTFYVWSSMICDKHNLVNVSFWTEPALVLNLYYHMDLLISNGHF---KS  174
                 ++C+I+DTF  W+  + +   L  V FWT  A V ++   + LL+S G 
KS
Sbjct  103  
TPFPPLSCIISDTFLPWTQDLANAVALPRVDFWTSTAAVYSMGTQLSLLVSMGTLPLPKS  162

Query  175  --LDNRKD------
VIDYVPGVKAIEPKDLMSYLQVSDKDVDTNTVVYRILFKAFKDVKR  226
             +D+ K       +ID +PG+      DL       ++  D++    R +  AF 
+
Sbjct  163  CWVDSEKKWKVEAPLIDRIPGLPPFPATDLPVQFVHPEELTDSS---
LRFMMDAFGRARE  219

Query  227  ADFVVCNTVQELEPDSLSALQAKQ-PVYAIGPVFSTDSVVPTSLWAESD-----
CTEWLK  280
            A  +  ++  ELE     ALQA   P++A+GP   + S  P+S    SD     C 
+WL
Sbjct  220  AHTIFVHSAYELEGQVFDALQANGFPIHAVGPFLDSPSE-
PSSYTTLSDNANQECIQWLD  278

Query  281  
GRPTGSVLYVSFGSYAHVGKKEIVEIAHGLLLSGISFIWVLRPDIVGSNVPDFLPAGFVD  340
             +   SV+YV+ GS A +   E+  +A GL  SG+ F+WV+R D + S++ D LP 
GF+
Sbjct  279  
TQLPNSVVYVALGSIASLIPTEMHALALGLEASGLPFLWVIRRDSISSSLSDALPEGFLQ  338

Query  341  QAQDRG--
LVVQWCCQMEVISNPAVGGFFTHCGWNSILESVWCGLPLLCYPLLTDQFTN  397
            +  + G   ++ W  QMEV+ + AVG FF+HCGWNS LE +W G+P++  P   +Q 
+N
Sbjct  339  
RTVENGSARIISWAPQMEVLRHCAVGAFFSHCGWNSTLECMWEGVPMVACPRAAEQRSN  397


 Score = 18.5 bits (36),  Expect = 0.49, Method: Compositional 
matrix adjust.
 Identities = 11/30 (37%), Positives = 13/30 (43%), Gaps = 1/30 (3%)

Query  19   QGHVIPFVHLAIKLASHGFTITFVNTDSIH  48
            + H I FVH A +L    F     N   IH
Sbjct  219  EAHTI-FVHSAYELEGQVFDALQANGFPIH  247
```

```
 Score = 15.4 bits (28),  Expect = 4.5, Method: Compositional matrix
adjust.
 Identities = 5/13 (38%), Positives = 7/13 (54%), Gaps = 0/13 (0%)

Query  15   PYPLQGHVIPFVH  27
            P+P     + FVH
Sbjct  186  PFPATDLPVQFVH  198



Lambda      K        H        a         alpha
   0.322    0.137    0.425    0.792     4.96

Gapped
Lambda      K        H        a         alpha    sigma
   0.267   0.0410    0.140     1.90     42.6     43.6

Effective search space used: 168086



Matrix: BLOSUM62
Gap Penalties: Existence: 11, Extension: 1
Neighboring words threshold: 11
Window for multiple hits: 40



Query= sp|Q9SK82|U85A1_ARATH_UDP-
glycosyltransferase_85A1_OS=Arabidopsis_thaliana_GN=UGT85A1_PE=1_SV=
1

Length=489

Subject= 246374-102_1_ORF1
>sp|Q9SK82|U85A1_ARATH_UDP-
glycosyltransferase_85A1_OS=Arabidopsis_thaliana_GN=UGT85A1_PE=1_SV=
1|||9e-94

Length=505


 Score =   285 bits (730),  Expect = 9e-94, Method: Compositional
matrix adjust.
 Identities = 179/494 (36%), Positives = 273/494 (55%), Gaps =
48/494 (10%)

Query  10   QKPHVVCVPYPAQGHINPMMRVAKLLHARGFYVTFV---
NTVYNHNRFLRSRGSNALDGL  66
            ++PH+V +P++  QGHINP+M++AK L  RG  VTFV   NTV           S A
```

```
+
Sbjct  42   KQPHLVILPFAQQGHINPLMQLAKKLAERGARVTFVVSENTV----------
PSGAVK-T  90

Query  67   PSFRFESIADGL-
PETDMDATQDITALCESTMKNCLAPFRELLQRINAGDNVPPVSCIVS  125
            P+ +F  +AD L PE    ++   +     +M N  A FR+LLQR+   D  P VS
++
Sbjct  91   PNLQFVGVADNLSPERSRGSSFRDSV---DSMINMEAGFRDLLQRLR--
DESPGVSALIY  145

Query  126  DGCMSFTLDVAEELGVPEVLFWTTSGCAFLAYLHFYLFIEKGLCPLK----
DESYLTKEY  181
            D  M++   +A  L +P + F+TT+  +      F   +++G+ P +    +   L
K+
Sbjct  146
DAFMTWAPSIASSLCLPFICFFTTNATSCSLAYQFPSLLQRGILPFRRDGDENGPLPKDI  205

Query  182  LE--
DTVIDFIPTMKNVKLKDIPSFIRTTNPDDVMISFALRETERAKRASAIILNTFDDL  239
            +E  D V D +P    + L        T +       F ++  E    A+AIILNT
++L
Sbjct  206  IEGIDGVPDLLPEEFPLSL--------
TFDATHFQYRFLMKIGETMLDATAIILNTMEEL  257

Query  240
EHDVVHAMQSILPPVYSVGPLHLLANREIEEGSEIGMMSSNLWKEEMECLDWLDTKTQNS  299
            E + V A+++    +Y VGPL LL+ R I        +  N W E+  CL WLD+
+S
Sbjct  258  EPEAVAAVRA-HKSIYPVGPLLLLSGRPISR------
LDVNYWPEDDHCLPWLDSHPPSS  310

Query  300  VIYINFGSITVLSVKQLVEFAWGLAGSGKEFLWVIRPDLV-
AGEEAMVPPDFLMETKDRS  358
            V+Y++FGS+  L +    + A GL  S + FLWV+RPD +    E  +P  FL
TKDR
Sbjct  311
VLYVSFGSVASLPLPHFQQLALGLEASKQPFLWVVRPDSIDVPLELALPDGFLARTKDRG  370

Query  359  MLASWCPQEKVLSHPAIGGFLTHCGWNSILESLSCG-
VPMVCWPFFADQQMNCKFCCDEW  417
            ++ SW PQ  +LSHP+IGGFLTH GWNSI+E+LS G VPM+CWP  A+Q++N +
D W
Sbjct  371
LIISWGPQLLILSHPSIGGFLTHGGWNSIIENLSMGSVPMICWPHVAEQRLNRRLMVDHW  430

Query  418  DVGIEI----
GGDVKREEVEAVVRELMDGEKGKKMREKAVEWQRLAEKATEHKLGSSVMN  473
            ++G+ +     G V +EE+  VV +L++ GE+GK ++  A +   +A+KA  ++
GSS  +
Sbjct  431  NIGLRLQHEEDGSVAKEEIARVVHDLLQGERGKDLKNAASKVGDMAKKAVANE-
GSSQNS  489

Query  474  FETVVSKFLLGQKS  487
```

```
             FE +     L + S
Sbjct  490  FEALYKYVTLPKDS  503



Lambda      K        H        a         alpha
   0.321    0.136    0.422    0.792     4.96

Gapped
Lambda      K        H        a         alpha    sigma
   0.267   0.0410    0.140     1.90     42.6     43.6

Effective search space used: 214305




Query= sp|Q9SK82|U85A1_ARATH_UDP-
glycosyltransferase_85A1_OS=Arabidopsis_thaliana_GN=UGT85A1_PE=1_SV=
1

Length=489

Subject= 298264-69_1_ORF1
>sp|Q9SK82|U85A1_ARATH_UDP-
glycosyltransferase_85A1_OS=Arabidopsis_thaliana_GN=UGT85A1_PE=1_SV=
1|||3e-73

Length=498


 Score =   231 bits (590),  Expect = 2e-73, Method: Compositional
matrix adjust.
 Identities = 150/493 (30%), Positives = 249/493 (51%), Gaps =
40/493 (8%)

Query  2    GSQIIHNS-QKPHVVCVPYPAQGHINPMMRVAKLLHAR-
GFYVTFVNTVYNHNRFLRSRG  59
            G+ + H+  QK H++ VP+P +GH+N M+R+A+ L ++ G  V+F      H+
+
Sbjct  13
GAAMEHDEEQKIHILAVPFPLEGHVNGMLRLAQALASQHGVLVSFAYPARFHSLARKRNK  72

Query  60   SNALDGLPSFRFESIADGLP-
ETDMDATQDITALCESTMKNCLAPFRELLQRI------N  112
             +AL    + R E I DGLP E D   T D+       +   +  FR  L+ +
Sbjct  73   LSALQSHSTLRVEVINDGLPLEEDHPLTPDV-------
LFGSVPIFRHALELLLHNFLHQ  125

Query  113
AGDNVPPVSCIVSDGCMSFTLDVAEELGVPEVLFWTTSGCAFLAYLHFYLFIEKGLCPLK  172
               + PP SCIVSD    +T D+A    +P + FWT++   +         +  G+
PL
Sbjct  126
```

```
TEPSSPPPSCIVSDTFCPWTQDLANAAAIPRIDFWTSTAAVYSMGSQLSSLVSNGILPLP  185

Query  173  DESY--LTKEYLEDT-VIDFIPTMKNVKLKDIP-
SFIRTTNPDDVMISFALRETERAKRA  228
               +  L  ++  D  +ID +P +    + D+P  F+R  +  D  + F L    R
+ A
Sbjct  186
QSCWADLENQWSADAPLIDCVPGLPPFPVTDLPCEFVRPLHVSDPRLQFMLAAFGRTREA  245

Query  229
SAIILNTFDDLEHDVVHAMQSILPPVYSVGPLHLLANREIEEGSEIGMMSSNLWKEEMEC  288
             I++++  +LE  V  A+Q+   P+ +VGPL    +  IE  +     ++
+ EC
Sbjct  246  HTILVHSVYELESQVFDALQADGYPIQAVGPL---LDSHIETST-----
ATQANNAKQEC  297

Query  289  LDWLDTKTQNSVIYINFGSITVLSVKQLVEFAWGLAGSGKEFLWVIRPDLVAGE-
EAMVP  347
            L WLD ++ +SVIY+  GS+  L+  ++   A GL   G  FLWVIR D +    E
+P
Sbjct  298
LKWLDKQSPSSVIYVALGSLAKLNTAEMHSLALGLEACGHPFLWVIRGDAITDTLENALP  357

Query  348  PDFLMETKDRSM--
LASWCPQEKVLSHPAIGGFLTHCGWNSILESLSCGVPMVCWPFFAD  405
              FL  T ++ +  + +W PQ +VLSH +I  F +HCGWNS LE +  GVPMV  P
A+
Sbjct  358
EGFLQRTNEKGLGLIIAWAPQTEVLSHHSISAFFSHCGWNSTLECIWEGVPMVACPRGAE  417

Query  406  QQMNCKFCCDEWDVGIEI----
GGDVKREEVEAVVRELMDGEKGKKMREKAVEWQRLAEK  461
            Q  N ++  + W +G+++     G   +E V+  ++E++    G   +E+A+  +
LA
Sbjct  418  QVSNARWIVENWKIGVQVERQLDGSFTKEAVQRALKEVL----
GSTYKERALHAKTLARS  473

Query  462  ATEHKLGSSVMNF  474
            A ++  G+S  N
Sbjct  474  AVQNG-GTSHSNL  485



Lambda      K        H        a         alpha
   0.321    0.136    0.422    0.792     4.96

Gapped
Lambda      K        H        a         alpha    sigma
   0.267   0.0410    0.140     1.90     42.6     43.6

Effective search space used: 214305
```

```
Query= sp|Q9SK82|U85A1_ARATH_UDP-
glycosyltransferase_85A1_OS=Arabidopsis_thaliana_GN=UGT85A1_PE=1_SV=
1

Length=489

Subject= 298364-69_1_ORF1
>sp|Q9SK82|U85A1_ARATH_UDP-
glycosyltransferase_85A1_OS=Arabidopsis_thaliana_GN=UGT85A1_PE=1_SV=
1|||2e-73

Length=502


 Score =   221 bits (563),  Expect = 2e-69, Method: Compositional
matrix adjust.
 Identities = 151/494 (31%), Positives = 250/494 (51%), Gaps =
38/494 (8%)

Query  2    GSQIIHNS-QKPHVVCVPYPAQGHINPMMRVAKLLHAR-
GFYVTFVNTVYNHNRFLRSRG  59
            G+ + H+  QK H++ VP+P +GH+N M+R+A+ L ++ G  V+F      H+
+
Sbjct  13
GAAMEHDEEQKIHILAVPFPLEGHVNGMLRLAQALASQHGVLVSFAYPARFHSLARKRNK  72

Query  60   SNALDGLPSFRFESIADGLP-
ETDMDATQDITALCESTMKNCLAPFRELL-------QRI  111
              +AL    + R E I DGLP E D   T D+        ++ L    ELL
Sbjct  73   LSALQSHSTLRVEVINDGLPLEEDHPLTPDVLFGSVPIFRHAL----
ELLLHNFLHQTEP  128

Query  112
NAGDNVPPVSCIVSDGCMSFTLDVAEELGVPEVLFWTTSGCAFLAYLHFYLFIEKGLCPL  171
            ++  + PP SCIVSD    +T D+A    +P + FWT++   +         +  G
+ PL
Sbjct  129
SSAPSYPPPSCIVSDTFCPWTQDLANAAAIPRIDFWTSTAAVYSMGSQLSSLVSNGILPL  188

Query  172  KDESY--LTKEYLEDT-VIDFIPTMKNVKLKDIP-
SFIRTTNPDDVMISFALRETERAKR  227
                +  L  ++  D  +ID +P +    + D+P  F+R  +  D  + F L
R +
Sbjct  189
PQSCWADLENQWSADAPLIDCVPGLPPFPVTDLPCEFVRPLHVSDPRLQFMLAAFGRTRE  248

Query  228
ASAIILNTFDDLEHDVVHAMQSILPPVYSVGPLHLLANREIEEGSEIGMMSSNLWKEEME  287
            A  I++++  +LE  V  A+Q+   P+ +VGPL    +  IE  +     ++
+ E
Sbjct  249  AHTILVHSVYELESQVFDALQADGYPIQAVGPL---LDSHIETST-----
ATQANNAKQE  300
```

```
Query  288  CLDWLDTKTQNSVIYINFGSITVLSVKQLVEFAWGLAGSGKEFLWVIRPDLVAGE-
EAMV  346
            CL WLD ++ +SVIY+  GS+  L+  ++   A GL   G  FLWVIR D +
E  +
Sbjct  301
CLKWLDKQSPSSVIYVALGSLAKLNTAEMHSLALGLEACGHPFLWVIRGDAITDTLENAL  360

Query  347  PPDFLMETKDRSM--
LASWCPQEKVLSHPAIGGFLTHCGWNSILESLSCGVPMVCWPFFA  404
            P  FL  T ++ +  + +W PQ +VLSH +I  F +HCGWNS LE +  GVPMV
P  A
Sbjct  361
PEGFLQRTNEKGLGLIIAWAPQTEVLSHHSISAFFSHCGWNSTLECIWEGVPMVACPRGA  420

Query  405  DQQMNCKFCCDEWDVGIEI----
GGDVKREEVEAVVRELMDGEKGKKMREKAVEWQRLAE  460
            +Q  N ++  + W +G+++     G   +E V+  ++E++    G   +E+A+  +
LA
Sbjct  421  EQVSNARWIVENWKIGVQVERQLDGSFTKEAVQRALKEVL----
GSTYKERALHAKTLAR  476

Query  461  KATEHKLGSSVMNF  474
             A ++  G+S  N
Sbjct  477  SAVQNG-GTSHSNL  489



Lambda      K        H        a         alpha
   0.321    0.136    0.422    0.792     4.96

Gapped
Lambda      K        H        a         alpha    sigma
   0.267   0.0410    0.140     1.90     42.6     43.6

Effective search space used: 214305




Query= sp|Q9SK82|U85A1_ARATH_UDP-
glycosyltransferase_85A1_OS=Arabidopsis_thaliana_GN=UGT85A1_PE=1_SV=
1

Length=489

Subject= 301636-68_1_ORF2
>sp|Q9SK82|U85A1_ARATH_UDP-
glycosyltransferase_85A1_OS=Arabidopsis_thaliana_GN=UGT85A1_PE=1_SV=
1|||9e-94

Length=495


 Score =   285 bits (729),  Expect = 9e-94, Method: Compositional
```

```
matrix adjust.
 Identities = 177/501 (35%), Positives = 268/501 (53%), Gaps =
45/501 (9%)

Query  8
NSQKPHVVCVPYPAQGHINPMMRVAKLLHARGFYVTFVNTVYNHNRFLRSRGSN-----A  62
            ++ K HVV VP PAQGH+NP+MR  KLL ++GF VTFVN    H+R   +
Sbjct  14
STSKQHVVAVPLPAQGHLNPLMRFCKLLASQGFLVTFVNIDRVHHRIEEAANRQEEEEAV  73

Query  63   LDGLPSFRFESIADGLPETDMDATQDITALCESTMKNCLAP-
FRELLQRINAGDNVPPVS  121
              G            +P  D+ +   ++        N +AP    L+ ++N
PPV+
Sbjct  74   YAGAAEDNIRKAHIPVPGLDLASDFRLSFQLFYDALNSIAPQLEHLILQLNRQG--
PPVT  131

Query  122  CIVSDGCMSF-
TLDVAEELGVPEVLFWTTSGCAFLAYLHFYLFIEKGLCPLKDESYLTKE  180
            CIVSD  +S  T  VA++L +P +  +  S    L  L +++   + +   K
LT++
Sbjct  132  CIVSDRALSLPTQSVADKLNIPRIALFPASASMLL--
LLYHVVQGEHISTTKVLKALTRK  189

Query  181  YLEDTVI----DFIPTMKNVKLKDIPSFIRTTNPDDVMISFALRETERA-
KRASAIILNT  235
                 V+      +PT+ N    ++PSF    +       F+ +  E + +RASA+
++N+
Sbjct  190  AERREVVCEGLAGLPTIYN---
DELPSFKHVVDESPFFWDFSTKTCEESFRRASAMVINS  246

Query  236  FDDLEHDVVHAMQSILP-
PVYSVGPLHLLANREIEEGSEIGMMSSNLWKEEMECLDWLDT  294
            F+DLE   + A+   L  P+Y VGPL    ++E          S++LWKE+  C+
WLD
Sbjct  247  FEDLEAQALVALSKHLSLPIYGVGPLVEPLHKE---------
SSTSLWKEDDACVLWLDQ  297

Query  295  KTQNSVIYINFGSITVLSVKQLVEFAWGLAGSGKEFLWVIRPDLVAGEEA--
MVPPDFLM  352
            +   SV+YI+FGSIT+LS  Q  E   GL  S + FLWV RPDLV   E+
D L
Sbjct  298
QPPLSVLYISFGSITLLSQPQFEEIVAGLLSSQQRFLWVFRPDLVENVESSSTFVSDVLS  357

Query  353
ETKDRSMLASWCPQEKVLSHPAIGGFLTHCGWNSILESLSCGVPMVCWPFFADQQMNCKF  412
             +  +  +  W PQ  VL+HP++GGFLTHCGWNS +E+++ GVPM+CWP+FADQQ
+N K+
Sbjct  358
RSHGKGYVVDWAPQLPVLAHPSVGGFLTHCGWNSTMEAIAHGVPMLCWPYFADQQLNAKY  417

Query  413  CCDEWDVGIEIGGD---------
VKREEVEAVVRELMDGEKGKKMREKAVEWQRLAEKAT  463
```

```
             +EW VG++            V+R EVE VV+ LM GE+G+ +R+  +   RL
E  +
Sbjct  418  VVEEWKVGLQFESSKEGANKCCLVERGEVERVVKALMQGEEGRGLRKNVM---
RLKEAGS  474

Query  464  E--HKLGSSVMNFETVVSKFL  482
            +     G+S  N E ++  F+
Sbjct  475  QSLQPGGTSHSNMEALIRSFM  495



Lambda      K        H        a         alpha
   0.321    0.136    0.422    0.792     4.96

Gapped
Lambda      K        H        a         alpha    sigma
   0.267   0.0410    0.140     1.90     42.6     43.6

Effective search space used: 214305




Query= sp|Q9SK82|U85A1_ARATH_UDP-
glycosyltransferase_85A1_OS=Arabidopsis_thaliana_GN=UGT85A1_PE=1_SV=
1

Length=489

Subject= 322573-56_4_ORF1
>sp|Q9SK82|U85A1_ARATH_UDP-
glycosyltransferase_85A1_OS=Arabidopsis_thaliana_GN=UGT85A1_PE=1_SV=
1|||2e-72

Length=500


 Score =   229 bits (583),  Expect = 2e-72, Method: Compositional
matrix adjust.
 Identities = 144/471 (31%), Positives = 237/471 (50%), Gaps =
38/471 (8%)

Query  13   HVVCVPYPAQGHINPMMRVAKLLHAR-
GFYVTFVNTVYNHNRFLRSRGSNALDGLPSFRF  71
            H++ VP+P +GH+N M+R+A+ L ++ G  V+F      H+   +    +AL
+ R
Sbjct  27
HILAVPFPLEGHVNGMLRLAQALASQHGVLVSFAYPARFHSLARKRNKLSALQSHSTLRV  86

Query  72   ESIADGLP-ETDMDATQDITALCESTMKNCLAPFRELLQRI------
NAGDNVPPVSCIV  124
            E I DGLP E D   T D+       +   +  FR  L+ +          + PP
SCIV
Sbjct  87   EVINDGLPLEEDHPLTPDV-------
```

```
LFGSVPIFRHALELLLHNFLHQTEPSSPPPSCIV  139

Query  125  SDGCMSFTLDVAEELGVPEVLFWTTSGCAFLAYLHFYLFIEKGLCPLKDESY--
LTKEYL  182
            SD    +T D+A    +P + FWT++   +         +  G+ PL    +  L
++
Sbjct  140
SDTFCPWTQDLANAAAIPRIDFWTSTAAVYSMGSQLSSLVSNGILPLPQSCWADLENQWS  199

Query  183  EDT-VIDFIPTMKNVKLKDIP-
SFIRTTNPDDVMISFALRETERAKRASAIILNTFDDLE  240
             D  +ID +P +    + D+P  F+R  +  D  + F L    R + A  I++++
+LE
Sbjct  200
ADAPLIDCVPGLPPFPVTDLPCEFVRPLHVSDPRLQFMLAAFGRTREAHTILVHSVYELE  259

Query  241
HDVVHAMQSILPPVYSVGPLHLLANREIEEGSEIGMMSSNLWKEEMECLDWLDTKTQNSV  300
             V  A+Q+   P+ +VGPL    +  IE  +     ++     + ECL+WLD +
+SV
Sbjct  260  SQVFDALQADGYPIQAVGPL---LDSHIETST-----
ATQANNAKHECLEWLDKQAPSSV  311

Query  301  IYINFGSITVLSVKQLVEFAWGLAGSGKEFLWVIRPDLVAGE-
EAMVPPDFLMETKDRSM  359
            IY+  GS+  L+  ++   A GL   G  FLWVIR D +    E  +P  FL  T
++ +
Sbjct  312
IYVALGSLAKLNTAEMHSLALGLEACGHPFLWVIRGDAITDTLENALPEGFLQRTNEKGL  371

Query  360  --
LASWCPQEKVLSHPAIGGFLTHCGWNSILESLSCGVPMVCWPFFADQQMNCKFCCDEW  417
             + +W PQ +VLSH +I  F +HCGWNS LE +  GVPMV  P  A+Q  N ++
+ W
Sbjct  372
GLIIAWAPQTEVLSHHSISAFFSHCGWNSTLECIWEGVPMVACPRGAEQVSNARWIVENW  431

Query  418  DVGIEI----GGDVKREEVEAVVRELMDGEKGKKMREKAVEWQRLAEKATE  464
             +G+++     G   +E V+  ++E++    G   +EKA+  + LA  A +
Sbjct  432  KIGVQVERQLDGSFTKEAVQRALKEVL----GSSYKEKALHAKTLARSAVQ  478


 Score = 14.6 bits (26),  Expect = 8.0, Method: Compositional matrix
adjust.
 Identities = 9/47 (19%), Positives = 17/47 (36%), Gaps = 0/47 (0%)

Query  423  IGGDVKREEVEAVVRELMDGEKGKKMREKAVEWQRLAEKATEHKLGS  469
            I GD   + +E  + E       +K     + W    E  + H + +
Sbjct  345  IRGDAITDTLENALPEGFLQRTNEKGLGLIIAWAPQTEVLSHHSISA  391



Lambda      K        H        a         alpha
   0.321    0.136    0.422    0.792     4.96
```

```
Gapped
Lambda      K        H        a         alpha    sigma
   0.267   0.0410    0.140     1.90     42.6     43.6

Effective search space used: 214305




Query= sp|Q9SK82|U85A1_ARATH_UDP-
glycosyltransferase_85A1_OS=Arabidopsis_thaliana_GN=UGT85A1_PE=1_SV=
1

Length=489

Subject= 323716-56_4_ORF1
>sp|Q9SK82|U85A1_ARATH_UDP-
glycosyltransferase_85A1_OS=Arabidopsis_thaliana_GN=UGT85A1_PE=1_SV=
1|||1e-72

Length=504


 Score =   219 bits (557),  Expect = 1e-68, Method: Compositional
matrix adjust.
 Identities = 147/486 (30%), Positives = 245/486 (50%), Gaps =
39/486 (8%)

Query  2    GSQIIHNSQKP---HVVCVPYPAQGHINPMMRVAKLLHAR-
GFYVTFVNTVYNHNRFLRS  57
            G+ + H+ ++    H++ VP+P +GH+N M+R+A+ L ++ G  V+F      H+
+
Sbjct  13
GAAMEHDEEQKIDVHILAVPFPLEGHVNGMLRLAQALASQHGVLVSFAYPARFHSLARKR  72

Query  58   RGSNALDGLPSFRFESIADGLP-
ETDMDATQDITALCESTMKNCLAPFRELL-------Q  109
               +AL    + R E I DGLP E D   T D+        ++ L    ELL
Sbjct  73   NKLSALQSHSTLRVEVINDGLPLEEDHPLTPDVLFGSVPIFRHAL----
ELLLHNFLHQT  128

Query  110
RINAGDNVPPVSCIVSDGCMSFTLDVAEELGVPEVLFWTTSGCAFLAYLHFYLFIEKGLC  169
             ++  + PP SCIVSD    +T D+A    +P + FWT++   +         +
G+
Sbjct  129
EPSSAPSYPPPSCIVSDTFCPWTQDLANAAAIPRIDFWTSTAAVYSMGSQLSSLVSNGIL  188

Query  170  PLKDESY--LTKEYLEDT-VIDFIPTMKNVKLKDIP-
SFIRTTNPDDVMISFALRETERA  225
            PL    +  L  ++  D  +ID +P +    + D+P  F+R  +  D  + F L
R
Sbjct  189
```

```
PLPQSCWADLENQWSADAPLIDCVPGLPPFPVTDLPCEFVRPLHVSDPRLQFMLAAFGRT  248

Query  226
KRASAIILNTFDDLEHDVVHAMQSILPPVYSVGPLHLLANREIEEGSEIGMMSSNLWKEE  285
            + A  I++++  +LE  V  A+Q+   P+ +VGPL    +  IE  +     ++
+
Sbjct  249  REAHTILVHSVYELESQVFDALQADGYPIQAVGPL---LDSHIETST-----
ATQANNAK  300

Query  286
MECLDWLDTKTQNSVIYINFGSITVLSVKQLVEFAWGLAGSGKEFLWVIRPDLVAGE-EA  344
             ECL+WLD +  +SVIY+  GS+  L+  ++   A GL   G  FLWVIR D +
E
Sbjct  301
HECLEWLDKQAPSSVIYVALGSLAKLNTAEMHSLALGLEACGHPFLWVIRGDAITDTLEN  360

Query  345  MVPPDFLMETKDRSM--
LASWCPQEKVLSHPAIGGFLTHCGWNSILESLSCGVPMVCWPF  402
             +P  FL  T ++ +  + +W PQ +VLSH +I  F +HCGWNS LE +  GVPMV
P
Sbjct  361
ALPEGFLQRTNEKGLGLIIAWAPQTEVLSHHSISAFFSHCGWNSTLECIWEGVPMVACPR  420

Query  403  FADQQMNCKFCCDEWDVGIEI----
GGDVKREEVEAVVRELMDGEKGKKMREKAVEWQRL  458
             A+Q  N ++  + W +G+++     G   +E V+  ++E++    G   +EKA+
+ L
Sbjct  421  GAEQVSNARWIVENWKIGVQVERQLDGSFTKEAVQRALKEVL----
GSSYKEKALHAKTL  476

Query  459  AEKATE  464
            A  A +
Sbjct  477  ARSAVQ  482


 Score = 14.6 bits (26),  Expect = 9.5, Method: Compositional matrix
adjust.
 Identities = 9/47 (19%), Positives = 17/47 (36%), Gaps = 0/47 (0%)

Query  423  IGGDVKREEVEAVVRELMDGEKGKKMREKAVEWQRLAEKATEHKLGS  469
            I GD   + +E  + E       +K     + W    E  + H + +
Sbjct  349  IRGDAITDTLENALPEGFLQRTNEKGLGLIIAWAPQTEVLSHHSISA  395



Lambda      K        H        a         alpha
   0.321    0.136    0.422    0.792     4.96

Gapped
Lambda      K        H        a         alpha    sigma
   0.267   0.0410    0.140     1.90     42.6     43.6

Effective search space used: 214305
```

Query= sp|Q9SK82|U85A1_ARATH_UDP-glycosyltransferase_85A1_OS=Arabidopsis_thaliana_GN=UGT85A1_PE=1_SV=1

Length=489

Subject= 357148-41_2_ORF2
>sp|Q9SK82|U85A1_ARATH_UDP-glycosyltransferase_85A1_OS=Arabidopsis_thaliana_GN=UGT85A1_PE=1_SV=1|||3e-94

Length=502

Score = 286 bits (732), Expect = 3e-94, Method: Compositional matrix adjust.
Identities = 182/511 (36%), Positives = 275/511 (54%), Gaps = 50/511 (10%)

```
Query  1
MGSQIIHNSQKPHVVCVPYPAQGHINPMMRVAKLLHARGFYVTFVNTVYNHNRFL----R  56
            M  Q+  +++K HVV VP PAQGH+NP+MR  KLL ++GF VTFVN    H+R
R
Sbjct  13   MADQV--
STRKQHVVAVPLPAQGHLNPLMRFCKLLASQGFLVTFVNIDRVHHRIEEAANR  70

Query  57   SRGSNALD----GLPSFRFESIADGLPETDMDATQDITALCESTMKNCLAP-
FRELLQRI  111
                 A+D    G             +P  D+ +   ++        N +AP    L
+ ++
Sbjct  71
HEEEEAVDAASGGAAEDNIRKAHIPVPGLDLASDFRLSFQLFYDALNSIAPQLEHLILQL  130

Query  112  NAGDNVPPVSCIVSDGCMSF-
TLDVAEELGVPEVLFWTTSGCAFLAYLHFYLFIEKGLCP  170
            N     PPV+CIVSD  +S  T  VA++L +P +  +  S    L  L +++   +
+
Sbjct  131  NRQG--PPVTCIVSDRALSLPTQSVADKLNIPRIALFPASASMLL--
LLYHVVQGEHIST  186

Query  171  LKDESYLTKEYLEDTVI----
DFIPTMKNVKLKDIPSFIRTTNPDDVMISFALRETERA-  225
             K    LT++     V+      +PT+ N    ++PSF    +       F+ +
E +
Sbjct  187  TKVLKALTRKAERREVVCEGLAGLPTIYN---
DELPSFKHVVDESPFFWDFSTKTCEESF  243

Query  226  KRASAIILNTFDDLEHDVVHAMQSILP-
PVYSVGPLHLLANREIEEGSEIGMMSSNLWKE  284
            +RASA+++N+F+DLE   + A+   L  P+Y VGPL    ++E          S+
+LWKE
```

```
Sbjct  244  RRASAMVINSFEDLEAQALVALSKHLSLPIYGVGPLVEPLHKE---------
SSTSLWKE  294

Query  285
EMECLDWLDTKTQNSVIYINFGSITVLSVKQLVEFAWGLAGSGKEFLWVIRPDLVAGEEA  344
            +  C+ WLD +   SV+YI+FGSIT+LS  Q  E   GL  S + FLWV RPDLV
E+
Sbjct  295
DDACVLWLDQQPPLSVLYISFGSITLLSQPQFEEIVAGLLSSQQRFLWVFRPDLVENVES  354

Query  345  --
MVPPDFLMETKDRSMLASWCPQEKVLSHPAIGGFLTHCGWNSILESLSCGVPMVCWPF  402
                  D L  +  +  +  W PQ  VL+HP++GGFLTHCGWNS +E+++ GVPM
+CWP+
Sbjct  355
SSTFVSDVLSRSHGKGYVVDWAPQLPVLAHPSVGGFLTHCGWNSTMEAIAHGVPMLCWPY  414

Query  403  FADQQMNCKFCCDEWDVGIEIGGD---------
VKREEVEAVVRELMDGEKGKKMREKAV  453
            FADQQ+N K+  +EW VG++             V+R EVE VV+ LM GE+G+ +R
+  +
Sbjct  415
FADQQLNAKYVVEEWKVGLQFESSKEGANKCCLVERGEVERVVKALMQGEEGRGLRKNVM  474

Query  454  EWQRLAEKATE--HKLGSSVMNFETVVSKFL  482
               RL E  ++     G+S  N E ++  F+
Sbjct  475  ---RLKEAGSQSLQPGGTSHSNMEALIRSFM  502



Lambda      K        H        a         alpha
   0.321    0.136    0.422    0.792     4.96

Gapped
Lambda      K        H        a         alpha    sigma
   0.267   0.0410    0.140     1.90     42.6     43.6

Effective search space used: 214305




Query= sp|Q9SK82|U85A1_ARATH_UDP-
glycosyltransferase_85A1_OS=Arabidopsis_thaliana_GN=UGT85A1_PE=1_SV=
1

Length=489

Subject= 372264-34_4_ORF2
>sp|Q9SK82|U85A1_ARATH_UDP-
glycosyltransferase_85A1_OS=Arabidopsis_thaliana_GN=UGT85A1_PE=1_SV=
1|||1e-93

Length=488
```

```
 Score =   285 bits (728),  Expect = 1e-93, Method: Compositional
matrix adjust.
 Identities = 171/486 (35%), Positives = 265/486 (55%), Gaps =
40/486 (8%)

Query  10
QKPHVVCVPYPAQGHINPMMRVAKLLHARGFYVTFVNTVYNHNRFLRSRGSNALDGLPSF  69
            ++PH+V +P+  QGHINP+M++A+ L  RG  VTFV    + N      G
P+
Sbjct  28   KRPHIVILPFAQQGHINPLMQLARKLAERGARVTFV---
VSENTVPSGAGKT-----PNL  79

Query  70
RFESIADGLPETDMDATQDITALCESTMKNCLAPFRELLQRINAGDNVPPVSCIVSDGCM  129
            +F  +AD LP      +    ++   +M N  + FR+LLQR+   D  P +S ++
D  M
Sbjct  80   QFVGVADNLPAERSKGSSFRDSV--DSMLNMESGFRDLLQRLR--
DEPPGISGLIYDAFM  135

Query  130  SFTLDVAEELGVPEVLFWTTSGCAFLAYLHFYLFIEKGLCPLK----
DESYLTKEYLE--  183
            S+   VA  L +P + F+TT+  A           ++G+ P +    +   L K+
+E
Sbjct  136
SWAPSVASSLSLPFICFFTTNATACSLVYQLPSLRQRGILPFRRDGDENGPLPKDIIEGI  195

Query  184
DTVIDFIPTMKNVKLKDIPSFIRTTNPDDVMISFALRETERAKRASAIILNTFDDLEHDV  243
            D V D +P    + L        T +       F ++  E    A+AIILNT +
+LE +
Sbjct  196  DGVPDLLPEEFPLSL--------
TFDATHFQYRFLMKIGETMLDATAIILNTMEELEPEA  247

Query  244
VHAMQSILPPVYSVGPLHLLANREIEEGSEIGMMSSNLWKEEMECLDWLDTKTQNSVIYI  303
            V A+++    +Y VGPL LL+ R I        +  N W E+  CL WLD+
+SV+Y+
Sbjct  248  VAAVRA-HKSIYPVGPLLLLSGRPISR------
LDVNYWPEDDHCLPWLDSHPPSSVLYV  300

Query  304  NFGSITVLSVKQLVEFAWGLAGSGKEFLWVIRPDLV-
AGEEAMVPPDFLMETKDRSMLAS  362
            +FGS+  L +    + A GL  S + FLWV+RPD +    +  +P  FL  TKDR
++ S
Sbjct  301
SFGSVASLPLPHFQQLALGLEASKQRFLWVVRPDSIDVPLDLALPEGFLARTKDRGLIIS  360

Query  363  WCPQEKVLSHPAIGGFLTHCGWNSILESLSCG-
VPMVCWPFFADQQMNCKFCCDEWDVGI  421
            W PQ  +LSHP+IGGF TH GWNSI+E+LS G VPM+CWP  A+Q++N +   D W
++G+
Sbjct  361
```

WGPQLLILSHPSIGGFFTHGGWNSIIENLSMGSVPMICWPHVAEQRLNRRLMVDHWNIGL 420

Query 422 EI----
GGDVKREEVEAVVRELMDGEKGKKMREKAVEWQRLAEKATEHKLGSSVMNFETV 477
             +   G V +EE+ VV +L+ GE+GK ++ A +   +A+KA ++ GSS
+FE +
Sbjct 421 RLQYQEDGSVAKEEIARVVHDLLQGERGKDLKNAASKVGDMAKKAVANE-
GSSQNSFEAL 479

Query 478 VSKFLL 483
                +L
Sbjct 480 YKYVML 485

Lambda   K   H   a   alpha
   0.321   0.136   0.422   0.792   4.96

Gapped
Lambda   K   H   a   alpha   sigma
   0.267   0.0410   0.140   1.90   42.6   43.6

Effective search space used: 214305

Query= sp|Q9SK82|U85A1_ARATH_UDP-
glycosyltransferase_85A1_OS=Arabidopsis_thaliana_GN=UGT85A1_PE=1_SV=
1

Length=489

Subject= 372579-34_4_ORF1
>sp|Q9SK82|U85A1_ARATH_UDP-
glycosyltransferase_85A1_OS=Arabidopsis_thaliana_GN=UGT85A1_PE=1_SV=
1|||2e-93

Length=492

 Score =   284 bits (727), Expect = 2e-93, Method: Compositional
matrix adjust.
 Identities = 172/489 (35%), Positives = 266/489 (54%), Gaps =
46/489 (9%)

Query 10   QKPHVVCVPYPAQGHINPMMRVAKLLHARGFYVTFV---
NTVYNHNRFLRSRGSNALDGL 66
            ++PH+V +P+ QGHINP+M++A+ L RG VTFV   NTV   +
Sbjct 32   KRPHIVILPFAQQGHINPLMQLARKLAERGARVTFVVSENTV-----------
PSGAGKT 80

Query 67
PSFRFESIADGLPETDMDATQDITALCESTMKNCLAPFRELLQRINAGDNVPPVSCIVSD 126

```
            P+ +F  +AD LP      +    ++   +M N  + FR+LLQR+   D  P +S
++ D
Sbjct  81   PNLQFVGVADNLPAERSKGSSFRDSV--DSMLNMESGFRDLLQRLR--
DEPPGISGLIYD  136

Query  127  GCMSFTLDVAEELGVPEVLFWTTSGCAFLAYLHFYLFIEKGLCPLK----
DESYLTKEYL  182
              MS+   VA  L +P + F+TT+  A           ++G+ P +    +   L
K+ +
Sbjct  137
AFMSWAPSVASSLSLPFICFFTTNATACSLVYQLPSLRQRGILPFRRDGDENGPLPKDII  196

Query  183  E--
DTVIDFIPTMKNVKLKDIPSFIRTTNPDDVMISFALRETERAKRASAIILNTFDDLE  240
            E  D V D +P    + L        T +       F ++  E    A+AIILNT
++LE
Sbjct  197  EGIDGVPDLLPEEFPLSL--------
TFDATHFQYRFLMKIGETMLDATAIILNTMEELE  248

Query  241
HDVVHAMQSILPPVYSVGPLHLLANREIEEGSEIGMMSSNLWKEEMECLDWLDTKTQNSV  300
             + V A+++    +Y VGPL LL+ R I        +  N W E+  CL WLD+
+SV
Sbjct  249  PEAVAAVRA-HKSIYPVGPLLLLSGRPISR------
LDVNYWPEDDHCLPWLDSHPPSSV  301

Query  301  IYINFGSITVLSVKQLVEFAWGLAGSGKEFLWVIRPDLV-
AGEEAMVPPDFLMETKDRSM  359
            +Y++FGS+  L +    + A GL  S + FLWV+RPD +    +  +P  FL
TKDR +
Sbjct  302
LYVSFGSVASLPLPHFQQLALGLEASKQRFLWVVRPDSIDVPLDLALPEGFLARTKDRGL  361

Query  360  LASWCPQEKVLSHPAIGGFLTHCGWNSILESLSCG-
VPMVCWPFFADQQMNCKFCCDEWD  418
            + SW PQ  +LSHP+IGGF TH GWNSI+E+LS G VPM+CWP  A+Q++N +
D W+
Sbjct  362
IISWGPQLLILSHPSIGGFFTHGGWNSIIENLSMGSVPMICWPHVAEQRLNRRLMVDHWN  421

Query  419  VGIEI----
GGDVKREEVEAVVRELMDGEKGKKMREKAVEWQRLAEKATEHKLGSSVMNF  474
            +G+ +     G V +EE+  VV +L++ GE+GK ++  A +   +A+KA  ++ GSS
+F
Sbjct  422  IGLRLQYQEDGSVAKEEIARVVHDLLQGERGKDLKNAASKVGDMAKKAVANE-
GSSQNSF  480

Query  475  ETVVSKFLL  483
            E +    +L
Sbjct  481  EALYKYVML  489



Lambda      K        H        a         alpha
```

```
   0.321    0.136    0.422    0.792     4.96

Gapped
Lambda      K        H        a         alpha    sigma
   0.267   0.0410    0.140     1.90     42.6     43.6

Effective search space used: 214305




Query= sp|Q9SK82|U85A1_ARATH_UDP-
glycosyltransferase_85A1_OS=Arabidopsis_thaliana_GN=UGT85A1_PE=1_SV=
1

Length=489

Subject= 379558-31_6_ORF1
>sp|Q9SK82|U85A1_ARATH_UDP-
glycosyltransferase_85A1_OS=Arabidopsis_thaliana_GN=UGT85A1_PE=1_SV=
1|||3e-72

Length=504


 Score =   218 bits (556),  Expect = 2e-68, Method: Compositional
matrix adjust.
 Identities = 149/496 (30%), Positives = 250/496 (50%), Gaps =
40/496 (8%)

Query  2    GSQIIHNSQKP---HVVCVPYPAQGHINPMMRVAKLLHAR-
GFYVTFVNTVYNHNRFLRS  57
            G+ + H+ ++    H++ VP+P +GH+N M+R+A+ L ++ G  V+F      H+
+
Sbjct  13
GAAMEHDEEQKIDVHILAVPFPLEGHVNGMLRLAQALASQHGVLVSFAYPARFHSLARKR  72

Query  58   RGSNALDGLPSFRFESIADGLP-
ETDMDATQDITALCESTMKNCLAPFRELL-------Q  109
               +AL    + R E I DGLP E D   T D+        ++ L    ELL
Sbjct  73   NKLSALQSHSTLRVEVINDGLPLEEDHPLTPDVLFGSVPIFRHAL----
ELLLHNFLHQT  128

Query  110
RINAGDNVPPVSCIVSDGCMSFTLDVAEELGVPEVLFWTTSGCAFLAYLHFYLFIEKGLC  169
             ++  + PP SCIVSD    +T D+A    +P + FWT++   +         +
G+
Sbjct  129
EPSSAPSYPPPSCIVSDTFCPWTQDLANAAAIPRIDFWTSTAAVYSMGSQLSSLVSNGIL  188

Query  170  PLKDESY--LTKEYLEDT-VIDFIPTMKNVKLKDIP-
SFIRTTNPDDVMISFALRETERA  225
            PL    +  L  ++  D  +ID +P +    + D+P  F+R  +  D  + F L
R
```

```
Sbjct  189
PLPQSCWADLENQWSADAPLIDCVPGLPPFPVTDLPCEFVRPLHVSDPRLQFMLAAFGRT  248

Query  226
KRASAIILNTFDDLEHDVVHAMQSILPPVYSVGPLHLLANREIEEGSEIGMMSSNLWKEE  285
            + A  I++++  +LE  V  A+Q+   P+ +VGPL    +  IE  +     ++
+
Sbjct  249  REAHTILVHSVYELESQVFDALQADGYPIQAVGPL---LDSHIETST-----
ATQANNAK  300

Query  286
MECLDWLDTKTQNSVIYINFGSITVLSVKQLVEFAWGLAGSGKEFLWVIRPDLVAGE-EA  344
             ECL+WLD +  +SVIY+  GS+  L+  ++   A GL   G  FLWVIR D +
E
Sbjct  301
HECLEWLDKQAPSSVIYVALGSLAKLNTAEMHSLALGLEACGHPFLWVIRGDAITDTLEN  360

Query  345  MVPPDFLMETKDRSM--
LASWCPQEKVLSHPAIGGFLTHCGWNSILESLSCGVPMVCWPF  402
             +P  FL  T ++ +  + +W PQ +VLSH +I  F +HCGWNS LE +  GVPMV
P
Sbjct  361
ALPEGFLQRTNEKGLGLIIAWAPQTEVLSHHSISAFFSHCGWNSTLECIWEGVPMVACPR  420

Query  403  FADQQMNCKFCCDEWDVGIEI----
GGDVKREEVEAVVRELMDGEKGKKMREKAVEWQRL  458
             A+Q  N ++  + W +G+++     G   +E V+  ++E++    G   +E+A+
+ L
Sbjct  421  GAEQVSNARWIVENWKIGVQVERQLDGSFTKEAVQRALKEVL----
GSTYKERALHAKTL  476

Query  459  AEKATEHKLGSSVMNF  474
            A  A ++  G+S  N
Sbjct  477  ARSAVQNG-GTSHSNL  491



Lambda      K        H        a         alpha
   0.321    0.136    0.422    0.792     4.96

Gapped
Lambda      K        H        a         alpha    sigma
   0.267   0.0410    0.140     1.90     42.6     43.6

Effective search space used: 214305




Query= sp|Q9SK82|U85A1_ARATH_UDP-
glycosyltransferase_85A1_OS=Arabidopsis_thaliana_GN=UGT85A1_PE=1_SV=
1

Length=489
```

```
Subject= 380391-31_6_ORF1
>sp|Q9SK82|U85A1_ARATH_UDP-
glycosyltransferase_85A1_OS=Arabidopsis_thaliana_GN=UGT85A1_PE=1_SV=
1|||3e-72

Length=500


 Score =   228 bits (582),  Expect = 3e-72, Method: Compositional
matrix adjust.
 Identities = 146/481 (30%), Positives = 242/481 (50%), Gaps =
39/481 (8%)

Query  13   HVVCVPYPAQGHINPMMRVAKLLHAR-
GFYVTFVNTVYNHNRFLRSRGSNALDGLPSFRF  71
            H++ VP+P +GH+N M+R+A+ L ++ G  V+F      H+   +    +AL
+ R
Sbjct  27
HILAVPFPLEGHVNGMLRLAQALASQHGVLVSFAYPARFHSLARKRNKLSALQSHSTLRV  86

Query  72   ESIADGLP-ETDMDATQDITALCESTMKNCLAPFRELLQRI------
NAGDNVPPVSCIV  124
            E I DGLP E D   T D+       +   +  FR  L+ +          + PP
SCIV
Sbjct  87   EVINDGLPLEEDHPLTPDV-------
LFGSVPIFRHALELLLHNFLHQTEPSSPPPSCIV  139

Query  125  SDGCMSFTLDVAEELGVPEVLFWTTSGCAFLAYLHFYLFIEKGLCPLKDESY--
LTKEYL  182
            SD    +T D+A    +P + FWT++   +         +  G+ PL    +  L
++
Sbjct  140
SDTFCPWTQDLANAAAIPRIDFWTSTAAVYSMGSQLSSLVSNGILPLPQSCWADLENQWS  199

Query  183  EDT-VIDFIPTMKNVKLKDIP-
SFIRTTNPDDVMISFALRETERAKRASAIILNTFDDLE  240
             D  +ID +P +    + D+P  F+R  +  D  + F L    R + A  I++++
+LE
Sbjct  200
ADAPLIDCVPGLPPFPVTDLPCEFVRPLHVSDPRLQFMLAAFGRTREAHTILVHSVYELE  259

Query  241
HDVVHAMQSILPPVYSVGPLHLLANREIEEGSEIGMMSSNLWKEEMECLDWLDTKTQNSV  300
              V  A+Q+   P+ +VGPL    +  IE  +     ++     + ECL+WLD +
+SV
Sbjct  260  SQVFDALQADGYPIQAVGPL---LDSHIETST-----
ATQANNAKHECLEWLDKQAPSSV  311

Query  301  IYINFGSITVLSVKQLVEFAWGLAGSGKEFLWVIRPDLVAGE-
EAMVPPDFLMETKDRSM  359
            IY+  GS+  L+  ++   A GL   G  FLWVIR D +    E  +P  FL  T
++ +
Sbjct  312
```

```
IYVALGSLAKLNTAEMHSLALGLEACGHPFLWVIRGDAITDTLENALPEGFLQRTNEKGL  371

Query  360  --
LASWCPQEKVLSHPAIGGFLTHCGWNSILESLSCGVPMVCWPFFADQQMNCKFCCDEW  417
              + +W PQ +VLSH +I  F +HCGWNS LE +  GVPMV  P  A+Q  N ++
+ W
Sbjct  372
GLIIAWAPQTEVLSHHSISAFFSHCGWNSTLECIWEGVPMVACPRGAEQVSNARWIVENW  431

Query  418  DVGIEI----
GGDVKREEVEAVVRELMDGEKGKKMREKAVEWQRLAEKATEHKLGSSVMN  473
             +G+++     G   +E V+  ++E++    G   +E+A+  + LA  A ++  G
+S  N
Sbjct  432  KIGVQVERQLDGSFTKEAVQRALKEVL----GSTYKERALHAKTLARSAVQNG-
GTSHSN  486

Query  474  F  474

Sbjct  487  L  487



Lambda      K        H        a         alpha
   0.321    0.136    0.422    0.792     4.96

Gapped
Lambda      K        H        a         alpha    sigma
   0.267   0.0410    0.140     1.90     42.6     43.6

Effective search space used: 214305



Matrix: BLOSUM62
Gap Penalties: Existence: 11, Extension: 1
Neighboring words threshold: 11
Window for multiple hits: 40



Query= sp|Q9SKY8|
HSP7H_ARATH_Heat_shock_70_kDa_protein_8_OS=Arabidopsis_tha
liana_GN=HSP70-8_PE=2_SV=1

Length=563

Subject= 14509-636_6_ORF1
>sp|Q9SKY8|HSP7H_ARATH_Heat_shock_70_kDa_protein_8_OS=Arabidopsis_th
aliana_GN=HSP70-8_PE=2_SV=1|||0

Length=631
```

Score = 650 bits (1678), Expect = 0.0, Method: Compositional matrix adjust.
Identities = 320/569 (56%), Positives = 411/569 (72%), Gaps = 18/569 (3%)

```
Query  1    MAE-
AAYTVASDSENTGEEKSSSSPSLPEIALGIDIGTSQCSIAVWNGSQVHILRNTRNQ  59
            MAE + Y VASDSE    E+S +S    ++ALGID+G+S CS+AVW  SQV +L N
R
Sbjct  54   MAEHSGYIVASDSETAAGEESDASV---
QVALGIDLGSSFCSVAVWRNSQVEVLANPRGM  110

Query  60
KLIKSFVTFKDEVPAGGVSNQLAHEQEMLTGAAIFNMKRLVGRVDTDPVVHASKNLPFLV  119
            K + S V FK ++P  GVS  ++ + E+ +G+AI+  K LVGRVDTD VV   KN
PFLV
Sbjct  111  KRMPSHVLFKGDIPCSGVSAAIS-
DAELYSGSAIYLAKSLVGRVDTDQVVQTCKNSPFLV  169

Query  120
QTLDIGVRPFIAALVNNAWRSTTPEEVLAIFLVELRLMAEAQLKRPVRNVVLTVPVSFSR  179
            +TLDIG RPF+AALV+  WRSTTPEEVLAI LVELR MAEA L   VR+ VLT+P
SFSR
Sbjct  170
ETLDIGARPFLAALVDGVWRSTTPEEVLAIVLVELRAMAEAHLGHTVRSAVLTMPASFSR  229

Query  180
FQLTRFERACAMAGLHVLRLMPEPTAIALLYAQQQQMTTHDNMGSGSERLAVIFNMGAGY  239
            FQ TR ERACAMAGLHVLRLMPEPTA+ALLYAQ+QQ     +MGSG E+  +IFN
GAG+
Sbjct  230
FQQTRLERACAMAGLHVLRLMPEPTAVALLYAQEQQKALQGSMGSGIEKNVLIFNAGAGF  289

Query  240  CDVAVTATAGGVSQIKALAGSPIGGEDILQNTIRHIAPPN----
EEASGL--------LR  287
            CDVA+ ATAGGVSQI+A+AG  +GGE ++QN ++++        EE +G
LR
Sbjct  290
CDVAIAATAGGVSQIRAVAGESVGGEAMVQNLVKYVLNDYKSLFEEKTGTSFSRLSAKLR  349

Query  288
VAAQDAIHRLTDQENVQIEVDLGNGNKISKVLDRLEFEEVNQKVFEECERLVVQCLRDAR  347
             AA  A+H L+   +  +E ++G G  ISK + R EFE VN  +F+ CE L +QCL
A+
Sbjct  350  PAAGQAMHALSSSHSTVLECEIG-
GKLISKTITREEFEHVNADIFQRCEALAIQCLHQAQ  408

Query  348
VNGGDIDDLIMVGGCSYIPKVRTIIKNVCKKDEIYKGVNPLEAAVRGAALEGAVTSGIHD  407
            ++   ++D+I+VGGCS++P +R++I  VC   + YKG+NPLEAAV+GAA+EGA+
SG+ D
Sbjct  409
```

```
MSVDSLEDVILVGGCSHVPAMRSLIMKVCGGKQFYKGINPLEAAVQGAAMEGAIASGLTD  468

Query  408
PFGSLDLLTIQATPLAVGVRANGNKFIPVIPRNTMVPARKDLFFTTVQDNQKEALIIIYE  467
              G LDLLTIQA P ++G++  GN+F+PV+ +N  +PAR+D+  TT  DNQ
EALII+YE
Sbjct  469
STGRLDLLTIQALPHSLGLKVRGNEFLPVLQKNGAIPARRDVVVTTSHDNQTEALIIVYE  528

Query  468
GEGETVEENHLLGYFKLVGIPPAPKGVPEINVCMDIDASNALRVFAAVLMPGSSSPVVPV  527
            GE +    N LLG+FKL GIP A KGVP INVCMD+DAS+ LRV   V MPG
PVVP+
Sbjct  529
GEAKQASGNQLLGFFKLTGIPLAQKGVPLINVCMDVDASDVLRVLVGVCMPGVEKPVVPL  588

Query  528  IEVRMPTVDDGHGWCAQALNVKYGATLDL  556
            +EVRMPT+DDGHGWCA A+  KYG+ L+L
Sbjct  589  VEVRMPTIDDGHGWCADAIQSKYGSLLEL  617


 Score = 15.8 bits (29),  Expect = 6.3, Method: Compositional matrix
adjust.
 Identities = 5/7 (71%), Positives = 7/7 (100%), Gaps = 0/7 (0%)

Query  554  LDLITLQ  560
            LDL+T+Q
Sbjct  473  LDLLTIQ  479



Lambda      K        H        a         alpha
   0.319    0.135    0.388    0.792     4.96

Gapped
Lambda      K        H        a         alpha    sigma
   0.267   0.0410    0.140     1.90     42.6     43.6

Effective search space used: 312444




Query= sp|Q9SKY8|
HSP7H_ARATH_Heat_shock_70_kDa_protein_8_OS=Arabidopsis_tha
liana_GN=HSP70-8_PE=2_SV=1

Length=563

Subject= 309127-63_6_ORF1
>sp|Q9SKY8|HSP7H_ARATH_Heat_shock_70_kDa_protein_8_OS=Arabidopsis_th
aliana_GN=HSP70-8_PE=2_SV=1|||0

Length=631
```

```
 Score =   650 bits (1678),  Expect = 0.0, Method: Compositional
matrix adjust.
 Identities = 320/569 (56%), Positives = 411/569 (72%), Gaps =
18/569 (3%)

Query  1    MAE-
AAYTVASDSENTGEEKSSSSPSLPEIALGIDIGTSQCSIAVWNGSQVHILRNTRNQ  59
            MAE + Y VASDSE    E+S +S    ++ALGID+G+S CS+AVW  SQV +L N
R
Sbjct  54   MAEHSGYIVASDSETAAGEESDASV---
QVALGIDLGSSFCSVAVWRNSQVEVLANPRGM  110

Query  60
KLIKSFVTFKDEVPAGGVSNQLAHEQEMLTGAAIFNMKRLVGRVDTDPVVHASKNLPFLV  119
            K + S V FK ++P  GVS  ++ + E+ +G+AI+  K LVGRVDTD VV   KN
PFLV
Sbjct  111  KRMPSHVLFKGDIPCSGVSAAIS-
DAELYSGSAIYLAKSLVGRVDTDQVVQTCKNSPFLV  169

Query  120
QTLDIGVRPFIAALVNNAWRSTTPEEVLAIFLVELRLMAEAQLKRPVRNVVLTVPVSFSR  179
            +TLDIG RPF+AALV+  WRSTTPEEVLAI LVELR MAEA L   VR+ VLT+P
SFSR
Sbjct  170
ETLDIGARPFLAALVDGVWRSTTPEEVLAIVLVELRAMAEAHLGHTVRSAVLTMPASFSR  229

Query  180
FQLTRFERACAMAGLHVLRLMPEPTAIALLYAQQQQMTTHDNMGSGSERLAVIFNMGAGY  239
            FQ TR ERACAMAGLHVLRLMPEPTA+ALLYAQ+QQ     +MGSG E+  +IFN
GAG+
Sbjct  230
FQQTRLERACAMAGLHVLRLMPEPTAVALLYAQEQQKALQGSMGSGIEKNVLIFNAGAGF  289

Query  240  CDVAVTATAGGVSQIKALAGSPIGGEDILQNTIRHIAPPN----
EEASGL---------LR  287
            CDVA+ ATAGGVSQI+A+AG  +GGE ++QN ++++        EE +G
LR
Sbjct  290
CDVAIAATAGGVSQIRAVAGESVGGEAMVQNLVKYVLNDYKSLFEEKTGTSFSRLSAKLR  349

Query  288
VAAQDAIHRLTDQENVQIEVDLGNGNKISKVLDRLEFEEVNQKVFEECERLVVQCLRDAR  347
             AA  A+H L+   +  +E ++G G  ISK + R EFE VN  +F+ CE L +QCL
A+
Sbjct  350  PAAGQAMHALSSSHSTVLECEIG-
GKLISKTITREEFEHVNADIFQRCEALAIQCLHQAQ  408

Query  348
VNGGDIDDLIMVGGCSYIPKVRTIIKNVCKKDEIYKGVNPLEAAVRGAALEGAVTSGIHD  407
            ++   ++D+I+VGGCS++P +R++I  VC   + YKG+NPLEAAV+GAA+EGA+
SG+ D
Sbjct  409
```

MSVDSLEDVILVGGCSHVPAMRSLIMKVCGGKQFYKGINPLEAAVQGAAMEGAIASGLTD 468

Query 408
PFGSLDLLTIQATPLAVGVRANGNKFIPVIPRNTMVPARKDLFFTTVQDNQKEALIIIYE 467
              G LDLLTIQA P ++G++ GN+F+PV+ +N +PAR+D+ TT DNQ
EALII+YE
Sbjct 469
STGRLDLLTIQALPHSLGLKVRGNEFLPVLQKNGAIPARRDVVVTTSHDNQTEALIIVYE 528

Query 468
GEGETVEENHLLGYFKLVGIPPAPKGVPEINVCMDIDASNALRVFAAVLMPGSSSPVVPV 527
            GE +   N LLG+FKL GIP A KGVP INVCMD+DAS+ LRV   V MPG
PVVP+
Sbjct 529
GEAKQASGNQLLGFFKLTGIPLAQKGVPLINVCMDVDASDVLRVLVGVCMPGVEKPVVPL 588

Query 528 IEVRMPTVDDGHGWCAQALNVKYGATLDL 556
            +EVRMPT+DDGHGWCA A+ KYG+ L+L
Sbjct 589 VEVRMPTIDDGHGWCADAIQSKYGSLLEL 617

Score = 15.8 bits (29), Expect = 6.4, Method: Compositional matrix adjust.
Identities = 5/7 (71%), Positives = 7/7 (100%), Gaps = 0/7 (0%)

Query 554 LDLITLQ 560
            LDL+T+Q
Sbjct 473 LDLLTIQ 479

Lambda	K	H	a	alpha
0.319	0.135	0.388	0.792	4.96

Gapped

Lambda	K	H	a	alpha	sigma
0.267	0.0410	0.140	1.90	42.6	43.6

Effective search space used: 312444

Matrix: BLOSUM62
Gap Penalties: Existence: 11, Extension: 1
Neighboring words threshold: 11
Window for multiple hits: 40

Query= sp|Q9SPV5|NEC1_NICPL_Nectarin-1_OS=Nicotiana_plumbaginifolia_GN=NEC1_PE=1_SV=1

```
Length=229

Subject= 2203-1350_3_ORF1
>sp|Q9SPV5|NEC1_NICPL_Nectarin-
1_OS=Nicotiana_plumbaginifolia_GN=NEC1_PE=1_SV=1|||3e-30

Length=254


 Score =   101 bits (252),  Expect = 3e-30, Method: Compositional
matrix adjust.
 Identities = 73/203 (36%), Positives = 95/203 (47%), Gaps = 23/203
(11%)

Query  19
LFLFAISIDRYCFAADEDMLQDVCVADLHSKVKVNGFPCKTNFTAADFSSFAISKPGATN  78
            L +  + +     AAD D L D           ++ F  +  FT  D S
PG T
Sbjct  46   LLVLTLFMSTTVLAADPDPLSDFTAG-------LSSFTLRDIFTNGDVSV----
GPGGTR  94

Query  79   NKFGSKVTTANVEQVPGLNTLGVSLARIDYAPGGINPPHTHPRASEMV-
FVMEGELDVGF  137
                    T NV   P + +  ++  +    P G+N PHTHPRASEM+  V  G L
VGF
Sbjct  95   -------
ATVNVAIFPAMASQSLTYTQFRMKPCGVNLPHTHPRASEMLTLVSGGPLQVGF  147

Query  138
ITTANVLVSKQITKGEVFVFPRGLVHFQKNNGKIPAAVVSAFNSQLPGTQSIPITLFGAS  197
            I TA V     +  G+V VFPRGL+HF+ N GK  A  +SA NSQ PG     +T
GA
Sbjct  148  IDTAGVAHIDILHSGDVTVFPRGLLHFELNVGKRTALYISALNSQNPGV----
LTAAGAL  203

Query  198  PTVPDDVLAQTFQINIEDVQQIK  220
              VP   LA      IE V+ ++
Sbjct  204  LNVPTRALATALNRTIEQVEALE  226



Lambda      K        H        a         alpha
   0.321    0.136    0.393    0.792     4.96

Gapped
Lambda      K        H        a         alpha    sigma
   0.267   0.0410    0.140     1.90     42.6     43.6

Effective search space used: 47586
```

```
Matrix: BLOSUM62
Gap Penalties: Existence: 11, Extension: 1
Neighboring words threshold: 11
Window for multiple hits: 40
```

```
Query= sp|Q9STW6|HSP7F_ARATH_Heat_shock_70_kDa_protein_6,
_chloroplastic_OS=Arabidopsis_thaliana_GN=HSP70-6_PE=1_SV=1

Length=718

Subject= 32621-443_2_ORF1
>sp|Q9STW6|HSP7F_ARATH_Heat_shock_70_kDa_protein_6,
_chloroplastic_OS=Arabidopsis_thaliana_GN=HSP70-6_PE=1_SV=1|||1e-79

Length=198


 Score =   235 bits (600),  Expect = 7e-77, Method: Compositional
matrix adjust.
 Identities = 122/159 (77%), Positives = 133/159 (84%), Gaps = 9/159
(6%)

Query  34   MPRSAFFGTRT---GPFSTPTSAFLRMGTRNGG--
GASRYAVGPVRVVNEKVVGIDLGTT  88
            +PR+AFFG        FS+     +R+   +GG   A R   GPVRVV
+EKVVGIDLGTT
Sbjct  44   LPRNAFFGAHALQKNRFSSQ----
IRLLRSDGGRSAARRSCGGPVRVVSEKVVGIDLGTT  99

Query  89
NSAVAAMEGGKPTIVTNAEGQRTTPSVVAYTKSGDRLVGQIAKRQAVVNPENTFFSVKRF  148
            NSAVAAMEGGKPTIVTNAEGQRTTPSVVAYTK
+GDRLVGQIAKRQAVVNPENTFFSVKRF
Sbjct  100
NSAVAAMEGGKPTIVTNAEGQRTTPSVVAYTKTGDRLVGQIAKRQAVVNPENTFFSVKRF  159

Query  149  IGRKMNEVDEESKQVSYRVVRDENNNVKLECPAINKQFA  187
            IGRKMNEVDEESKQ+SY+VVRD N NVKL+CPAI KQFA
Sbjct  160  IGRKMNEVDEESKQISYKVVRDSNGNVKLDCPAIGKQFA  198


 Score = 14.6 bits (26),  Expect = 4.6, Method: Compositional matrix
adjust.
 Identities = 8/42 (19%), Positives = 20/42 (48%), Gaps = 0/42 (0%)

Query  632  VKEKVEAKLQELKDKIGSGSTQEIKDAMAALNQEVMQIGQSL  673
            VK  +  K+ E+ ++    S + ++D+   +  +   IG+
Sbjct  156  VKRFIGRKMNEVDEESKQISYKVVRDSNGNVKLDCPAIGKQF  197
```

```
Lambda      K        H        a         alpha
   0.312    0.131    0.357    0.792     4.96

Gapped
Lambda      K        H        a         alpha    sigma
   0.267   0.0410    0.140     1.90     42.6     43.6

Effective search space used: 115584



Query= sp|Q9STW6|HSP7F_ARATH_Heat_shock_70_kDa_protein_6,
_chloroplastic_OS=Arabidopsis_thaliana_GN=HSP70-6_PE=1_SV=1

Length=718

Subject= 32859-441_3_ORF1
>sp|Q9STW6|HSP7F_ARATH_Heat_shock_70_kDa_protein_6,
_chloroplastic_OS=Arabidopsis_thaliana_GN=HSP70-6_PE=1_SV=1|||1e-79

Length=197


 Score =   235 bits (600),  Expect = 7e-77, Method: Compositional
matrix adjust.
 Identities = 122/159 (77%), Positives = 133/159 (84%), Gaps = 9/159
(6%)

Query  34   MPRSAFFGTRT---GPFSTPTSAFLRMGTRNGG--
GASRYAVGPVRVVNEKVVGIDLGTT  88
            +PR+AFFG        FS+     +R+   +GG   A R   GPVRVV
+EKVVGIDLGTT
Sbjct  43   LPRNAFFGAHALQKNRFSSQ----
IRLLRSDGGRSAARRSCGGPVRVVSEKVVGIDLGTT  98

Query  89
NSAVAAMEGGKPTIVTNAEGQRTTPSVVAYTKSGDRLVGQIAKRQAVVNPENTFFSVKRF  148
            NSAVAAMEGGKPTIVTNAEGQRTTPSVVAYTK
+GDRLVGQIAKRQAVVNPENTFFSVKRF
Sbjct  99
NSAVAAMEGGKPTIVTNAEGQRTTPSVVAYTKTGDRLVGQIAKRQAVVNPENTFFSVKRF  158

Query  149  IGRKMNEVDEESKQVSYRVVRDENNNVKLECPAINKQFA  187
            IGRKMNEVDEESKQ+SY+VVRD N NVKL+CPAI KQFA
Sbjct  159  IGRKMNEVDEESKQISYKVVRDSNGNVKLDCPAIGKQFA  197


 Score = 14.6 bits (26),  Expect = 4.6, Method: Compositional matrix
adjust.
 Identities = 8/42 (19%), Positives = 20/42 (48%), Gaps = 0/42 (0%)

Query  632  VKEKVEAKLQELKDKIGSGSTQEIKDAMAALNQEVMQIGQSL  673
```

```
            VK  +  K+ E+ ++    S + ++D+   +  +   IG+
Sbjct  155  VKRFIGRKMNEVDEESKQISYKVVRDSNGNVKLDCPAIGKQF  196



Lambda      K        H        a         alpha
   0.312    0.131    0.357    0.792     4.96

Gapped
Lambda      K        H        a         alpha    sigma
   0.267   0.0410    0.140     1.90     42.6     43.6

Effective search space used: 115584




Matrix: BLOSUM62
Gap Penalties: Existence: 11, Extension: 1
Neighboring words threshold: 11
Window for multiple hits: 40





Query= sp|Q9STX5|
ENPL_ARATH_Endoplasmin_homolog_OS=Arabidopsis_thaliana_GN=
SHD_PE=1_SV=1

Length=823

Subject= 149053-199_6_ORF2
>sp|Q9STX5|ENPL_ARATH_Endoplasmin_homolog_OS=Arabidopsis_thaliana_GN
=SHD_PE=1_SV=1|||0

Length=755


 Score =   627 bits (1616),  Expect = 0.0, Method: Compositional
matrix adjust.
 Identities = 355/717 (50%), Positives = 497/717 (69%), Gaps =
40/717 (6%)

Query  76
AEKFEFQAEVSRLMDIIINSLYSNKDIFLRELISNASDALDKIRFLALTDKDVLGEGDTA  135
             E F FQAE+++L+ +IIN+ YSNK+IFLRELISNASDALDKIRF +LTDK  L
Sbjct  59
TETFAFQAEINQLLSLIINTFYSNKEIFLRELISNASDALDKIRFESLTDKSKLDS--QP  116

Query  136
KLEIQIKLDKAKKILSIRDRGIGMTKEDLIKNLGTIAKSGTSAFVEKMQSSGDLNLIGQF  195
            +L I I  DKA   L+I D GIGMTK DL+ NLGTIA+SGT  F+E + +  D++
+IGQF
```

Sbjct 117
ELFIHIIPDKASNSLTIIDSGIGMTKADLVNNLGTIARSGTKEFMEALAAGADVSMIGQF 176

Query 196
GVGFYSAYLVADYIEVISKHNDDSQYVWESKANGKFAVSEDTWNEPLGRGTEIRLHLRDE 255
GVGFYSAYLVA+ + V SKHNDD QY+WES+A G F V+ DT EPLGRGT+I L
+L+++
Sbjct 177 GVGFYSAYLVAEKVIVTSKHNDDEQYIWESQAGGSFTVTRDT-
GEPLGRGTKITLYLKED 235

Query 256
AGEYLEESKLKELVKRYSEFINFPISLWASKEVETEVPVEEDESADEETETTSTEEEKEE 315
EY+EE +LK+LVK++SEFI++PISLW TE E++ DEE E EE
E
Sbjct 236 QLEYVEERRLKDLVKKHSEFISYPISLW------
TEKTTEKEVEDDEEEEEKKEEEGAVE 289

Query 316
DAEEEDGEKKQKTKKVKETVYEWELLNDVKAIWLRSPKEVTEEEYTKFYHSLSKDFTDEK 375
+ +EE ++K KKVKE +EW L+N K IW+R P+++T++EY+ FY SL+ D+
E+
Sbjct 290
EIDEEKEAPEKKKKKVKEVSHEWSLVNKQKPIWMRKPEDITKDEYSAFYKSLTNDW--EE 347

Query 376
PMAWSHFNAEGDVEFKAVLYVPPKAPHDLYESYYNSNKANLKLYVRRVFISDEFDELLPK 435
+A HF+ EG +EFKAVLYVP +AP DL+++ N N+KLYVRRVFI D
+EL+P+
Sbjct 348 HLAVKHFSVEGQLEFKAVLYVPKRAPFDLFDTRKKLN--
NIKLYVRRVFIMDNCEELIPE 405

Query 436
YLSFLKGLVDSDTLPLNVSREMLQQHSSLKTIKKKLIRKALDMIRKLAEEDPDEIHDDEK 495
YL F+KG+VDS+ LPLN+SREMLQQ+ LK I+K L++K L++ ++AE D
Sbjct 406
YLGFVKGVVDSEDLPLNISREMLQQNKILKVIRKNLVKKCLELFSEIAENKED------- 458

Query 496
KDVEKSGENDEKKGQYTKFWNEFGKSVKLGIIEDAANRNRLAKLLRFETTKSDGKLTSLD 555
+ KF+ FGK++KLGI ED++NR +LA+LLR+ +TKS +
+TS
Sbjct 459 ---------------
FDKFYESFGKNIKLGIHEDSSNRAKLAELLRYHSTKSGDEMTSFK 503

Query 556
QYIKRMKKSQKDIFYITGSSKEQLEKSPFLERLIKKGYEVIFFTDPVDEYLMQYLMDYED 615
Y+ RMK Q DI+YITG SK+ +E SPFLERL KKGYEV+F D +DEY + L
+Y+
Sbjct 504
DYVTRMKDGQNDIYYITGESKKAVENSPFLERLKKKGYEVLFLVDAIDEYAVGQLKEYDG 563

Query 616 KKFQNVSKEGLKVGKDSKDKELKE----
AFKELTKWWKGNLASENVDDVKISNRLADTPC 671
KK + +KEGLK+ + ++K+ +E AF+ L K K ++ + V+ V +S+R+

```
D+PC
Sbjct  564  KKLVSATKEGLKLEESEEEKKKQEEKKAAFENLCKVIK-
DILGDRVEKVVVSDRIVDSPC  622

Query  672
VVVTSKFGWSANMERIMQSQTLSDANKQAYMRGKRVLEINPRHPIIKELKDRIASDPEDE  731
             +VT ++GW+ANMERIM++Q L D++  +YM  K+ +EINP + I++EL+ R  +D
D+
Sbjct  623
CLVTGEYGWTANMERIMKAQALRDSSMSSYMSSKKTMEINPDNSIMEELRKRAEADKNDK  682

Query  732
SVKETAQLMYQTALIESGFILTDPKDFAARIYNSVKSGLNISPDAVADEEIEAAEEP  788
            SVK+   L+++TAL+ SGF L DP  F +RI+  +K GL+I  D  A  + +A
P
Sbjct  683
SVKDLILLLHETALLTSGFSLEDPNTFGSRIHRMLKLGLSIDDDTSASADADADMPP  739



Lambda      K        H        a         alpha
   0.311    0.130    0.358    0.792     4.96

Gapped
Lambda      K        H        a         alpha    sigma
   0.267   0.0410    0.140     1.90     42.6     43.6

Effective search space used: 558348




Matrix: BLOSUM62
Gap Penalties: Existence: 11, Extension: 1
Neighboring words threshold: 11
Window for multiple hits: 40





Query= sp|Q9SYB5|OST3B_ARATH_Probable_dolichyl-
diphosphooligosaccharide--
protein_glycosyltransferase_subunit_3B_OS=Arabidopsis_thaliana_GN=OS
T3B_PE=2_SV=1

Length=346

Subject= 49612-361_4_ORF2
>sp|Q9SYB5|OST3B_ARATH_Probable_dolichyl-diphosphooligosaccharide--
protein_glycosyltransferase_subunit_3B_OS=Arabidopsis_thaliana_GN=OS
T3B_PE=2_SV=1|||2e-127

Length=369
```

```
 Score =   367 bits (943),  Expect = 2e-129, Method: Compositional
matrix adjust.
 Identities = 168/324 (52%), Positives = 238/324 (73%), Gaps = 3/324
(1%)

Query  24
SFSDSDSDSDLLNELVSLRSTSESGVIHLDDHGISKFLTSASTPRPYSLLVFFDATQLHS  83
            S   +DS+++ ++ L+ L+S +  GVI  DD  + +F+  A   RPYSL+VFFDA
QL
Sbjct  48   SHGRADSNAERISSLLDLQSRTVDGVIRFDDGAVHRFIQEAEV-
RPYSLIVFFDALQLRD  106

Query  84   KNELRLQELRREFGIVSASFLANNNGS-
EGTKLFFCEIEFSKSQSSFQLFGVNALPHIRL  142
              +L L +LRREFG+++++++ NN  +   +K+FF ++EF +SQ SF LFGVN
+LPHIR
Sbjct  107
NADLHLVDLRREFGMLASAYIRNNQDTPAASKVFFIDLEFKQSQKSFALFGVNSLPHIRH  166

Query  143
VSPSISNLRDESGQMDQSDYSRLAESMAEFVEQRTKLKVGPIQRPPLLSKPQIGIIVALI  202
            ++   S+ +D    M+ SD++R AE MA FVE +TK KVG I+RPP +++ Q+ I
+V  +
Sbjct  167  IAAGSSSFKDVEA-
MEPSDFTRQAEGMASFVESKTKQKVGTIERPPPVTRKQLIILVGGL  225

Query  203
VIATPFIIKRVLKGETILHDTRLWLSGAIFIYFFSVAGTMHNIIRKMPMFLQDRNDPNKL  262
            +  TPFI+K++L G T+ HD ++W S ++F+YFFSV+G MHNIIR MP+F+QDRN
+P KL
Sbjct  226
LAVTPFILKKLLAGNTVFHDAKVWTSFSLFVYFFSVSGGMHNIIRSMPLFMQDRNNPGKL  285

Query  263
VFFYQGSGMQLGAEGFAVGFLYTVVGLLLAFVTNVLVRVKNITAQRLIMLLALFISFWAV  322
            VFFYQ SGMQLGAEGF VGFLYTV GL LA++T+ L RVK+ T QR +M+  + I
W V
Sbjct  286
VFFYQASGMQLGAEGFTVGFLYTVFGLALAYITHFLPRVKSQTVQRTVMMTCMVIGVWVV  345

Query  323  KKVVYLDNWKTGYGIHPYWPSSWR  346
            ++V+YLDNWKTGY +H YWP+ WR
Sbjct  346  RQVIYLDNWKTGYSVHGYWPNRWR  369



Lambda      K        H        a         alpha
   0.324    0.137    0.400    0.792     4.96

Gapped
Lambda      K        H        a         alpha    sigma
   0.267   0.0410    0.140     1.90     42.6     43.6
```

Effective search space used: 107780

Matrix: BLOSUM62
Gap Penalties: Existence: 11, Extension: 1
Neighboring words threshold: 11
Window for multiple hits: 40

Query= sp|Q9XGW1|
AGO10_ARATH_Protein_argonaute_10_OS=Arabidopsis_thaliana_G
N=AGO10_PE=2_SV=1

Length=988

Subject= 429495-5_4_ORF2
>sp|Q9XGW1|AGO10_ARATH_Protein_argonaute_10_OS=Arabidopsis_thaliana_
GN=AGO10_PE=2_SV=1|||3e-161

Length=648

Score = 481 bits (1239), Expect = 4e-161, Method: Compositional
matrix adjust.
Identities = 260/637 (41%), Positives = 389/637 (61%), Gaps =
24/637 (4%)

```
Query  131
RPGFGTLGTKCIVKANHFLADLPTKDLNQYDVTITPEVSSKSVNRAIIAELVRLYKESDL  190
            RPGFG  G    V ANHF      K L  YDV+I PE   K ++RAI+ + V  +
E
Sbjct  25   RPGFGQFGDPTAVFANHFRVRFEAKRLYHYDVSIEPE-
PPKGLHRAIMDQAVAKHGEKLQ  83

Query  191  GRRLPAYDGRKSLYTAGELPFTWKEFSVKIVDEDDGIINGPKRERS-
YKVAIKFVARANM  249
            G  +PAYDG+++LYTA EL     E  V++  +++G    P+R  + +K+ ++  +
+ +M
Sbjct  84   GA-IPAYDGQRALYTARELA-EQVELEVQLTQDNEG----
PRRRNNLFKITLREASKIDM  137

Query  250
HHLGEFLAGKRADCPQEAVQILDIVLRELSVKRFCPVGRSFFSPDIKTPQRLGEGLESWC  309
              L E+L GKR   PQE +Q+LDIVLRE++ K F   GR FF         LG G+
+++
Sbjct  138  GLLHEYLNGKRRILPQENLQVLDIVLREIACKYFLAKGRCFFGSHFGESN-
LGGGIQAYS  196
```

```
Query  310  GFYQSIRPTQMGL-SLNIDMASAAFIEPLPVIEFVAQLLGKDV------LSKP--
LSDSD  360
            GFYQSIRP Q GL SLNID+A+A F+E + + +F+ +   KDV      L +P
D
Sbjct  197
GFYQSIRPAQNGLLSLNIDIAAAPFLESVCLDDFLGKAFRKDVRALNLELGRPDGYGDQA  256

Query  361
RVKIKKGLRGVKVEVTHRANVRRKYRVAGLTTQPTRELMFPVDENCTMKSVIEYFQEMYG  420
            RVK KK L+G++VE  H    +RKYR+  +T +P R L F +D      SV++YF+
Y
Sbjct  257  RVKAKKLLKGIRVETIHNRGAKRKYRIQSITNEPLRNLRFDMDGVSV--
SVVDYFRRTYN  314

Query  421
FTIQHTHLPCLQVGNQKKASYLPMEACKIVEGQRYTKRLNEKQITALLKVTCQRPRDREN  480
            + I+   LP ++ G +++  YLPME CKIV GQ Y K ++E Q  ALL+VTC  P
+R+
Sbjct  315
YEIKFPGLPAVESGTRERKRYLPMEVCKIVAGQPYMKSMSEAQKKALLRVTCVLPGERKK  374

Query  481
DILRTVQHNAYDQDPYAKEFGMNISEKLASVEARILPAPWLKYHENGKEKDCLPQVGQWN  540
               +       +    AKEFG+ I+  L  + AR+LPAP ++YH    +    P
+G WN
Sbjct  375  TTEKIEVDLVKETSKIAKEFGVGINRFLTEIPARVLPAPSIQYHSATVQPP--
PGLGSWN  432

Query  541
MMNKKMINGMTVSRWACVNFSRSVQENVARGFCNELGQMCEVSGMEFNPEPVIPIYSARP  600
            M NK+M++G  ++RW+ +NF  ++ E+  + FC++L + CE  G+  NPE  +
+ R
Sbjct  433
MRNKRMVSGCQINRWSLINFCHNLNESDVKKFCDKLPRECETYGLAMNPEMAVEPLTERR  492

Query  601  DQVEKALKHVYHTSMNKTKGKELELLLAILPD-
NNGSLYGDLKRICETELGLISQCCLTK  659
             Q E A+KH+      K + ++L+LL  I+P  +N S+Y  +KR+CE ELGL+
+QCCL++
Sbjct  493  GQAEGAIKHLNRQCAEKFR-
QDLDLLFCIMPQTDNKSIYAAVKRVCEIELGLVTQCCLSE  551

Query  660
HVFKISKQYLANVSLKINVKMGGRNTVLVDAISCRIPLVSDIPTIIFGADVTHPENGEES  719
            HV K   +Y+AN+ LK+N KMGG N  LVD  + R+PL+S++ +II GADV+HP
G++
Sbjct  552
HVKKNRPEYIANLLLKVNAKMGGYNARLVDEQNRRLPLISEVRSIIIGADVSHPRVGDDY  611

Query  720  SPSIAAVVASQDWPEVTKYAGLVCAQAHRQELIQDLY  756
            SPSI+AV AS DWP  ++Y  +V  Q  R+E+ ++ +
Sbjct  612  SPSISAVAASMDWPYFSQYEAIVQTQRQREEVCEEFF  648
```

```
Lambda      K        H        a         alpha
   0.319    0.134    0.403    0.792     4.96

Gapped
Lambda      K        H        a         alpha    sigma
   0.267   0.0410    0.140     1.90     42.6     43.6

Effective search space used: 574829




Matrix: BLOSUM62
Gap Penalties: Existence: 11, Extension: 1
Neighboring words threshold: 11
Window for multiple hits: 40





Query= sp|Q9ZEJ6|
DNAK1_NOSS1_Chaperone_protein_dnaK1_OS=Nostoc_sp._(strain_
PCC_7120_/_UTEX_2576)_GN=dnaK1_PE=3_SV=2

Length=688

Subject= 825-1762_2_ORF1
>sp|Q9ZEJ6|DNAK1_NOSS1_Chaperone_protein_dnaK1_OS=Nostoc_sp._(strain
_PCC_7120_/_UTEX_2576)_GN=dnaK1_PE=3_SV=2|||0

Length=411


 Score =   580 bits (1494),  Expect = 0.0, Method: Compositional
matrix adjust.
 Identities = 277/410 (68%), Positives = 343/410 (84%), Gaps = 0/410
(0%)

Query  106
LNKEFSAEEISAMVLKKLADDASAYLGSAVTGAVITVPAYFNDSQRQATRDAGRIAGLEV  165
            + K+F+AEEISA VL+KL DDAS +L   V  AVITVPAYFNDSQR AT+DAGRIA
+EV
Sbjct  1
IGKQFAAEEISAQVLRKLVDDASKFLNDKVNKAVITVPAYFNDSQRTATKDAGRIASIEV  60

Query  166
LRILNEPTAASLAYGLDRGDTETILVFDLGGGTFDVSILEVGDGVFEVKATSGDTQLGGN  225
            LRI+NEPTAASLAYG +R   ETILVFDLGGGTFDVS+LEVGDGVFEV +TSGDT
LGG+
Sbjct  61
LRIINEPTAASLAYGFERKSNETILVFDLGGGTFDVSVLEVGDGVFEVLSTSGDTHLGGD  120
```

```
Query  226
DFDKKIVDWLAEQFLETEGVDLRRDRQALQRLMEAAEKAKIELSAVSITDINLPFITATE  285
            DFDK+IVDWLA  F + EG+DL +D+QALQRL E AEKAK+ELS ++ T I
+LPFITAT
Sbjct  121
DFDKRIVDWLAASFKKEEGIDLLKDKQALQRLTETAEKAKMELSTLTQTSISLPFITATA  180

Query  286
DGPKHLETRLTRSQFEGLCVDLLGRVRNPVKRALKDAGLRPDDIEEVVLVGGSTRMPMVK  345
            DGPKH++T +TR++FE LC DLL R + PV+ AL+DA L  +D++EV+LVGGSTR
+P V+
Sbjct  181
DGPKHIDTSITRAKFEELCSDLLDRCKTPVENALRDAKLSFNDVQEVILVGGSTRIPAVQ  240

Query  346
QLVRDLIGIEPSENVNPDEVVAMGAAIQAGILAGEFKDVLLLDVTPLSLGLEAIGGVMKK  405
            QLV+ + G +P+  VNPDEVVA+GAA+QAG+LAGE  D++LLDV+PLSLGLE
+GGVM K
Sbjct  241
QLVKRMTGKDPNVTVNPDEVVALGAAVQAGVLAGEVSDIVLLDVSPLSLGLETLGGVMTK  300

Query  406
LIPRNTTIPVRRSDIFSTSENNQNSVEIHVVQGEREMAGDNKSLGRFKLYGIPPAPRGIP  465
            +IPRNTT+P  +S++FST+ ++Q SVEI+V+QGERE   DNKSLG F+L
GIPPAPRG+P
Sbjct  301
IIPRNTTLPTSKSEVFSTAADSQTSVEINVLQGEREFVRDNKSLGSFRLDGIPPAPRGMP  360

Query  466  QIQVAFDIDANGILQVTALDRTTGREQSITIQGASTLSESEVNRMIQEAQ  515
            QI+V FDIDANGIL VTA D+ +G++Q ITI GASTL + EV RM+ EA+
Sbjct  361  QIEVKFDIDANGILSVTASDKGSGKKQDITITGASTLPKDEVERMVNEAE  410


 Score = 18.1 bits (35),  Expect = 0.77, Method: Compositional
matrix adjust.
 Identities = 7/17 (41%), Positives = 11/17 (65%), Gaps = 0/17 (0%)

Query  529  EKRTRSEALILQGERQL  545
            + +T  E  +LQGER+
Sbjct  321  DSQTSVEINVLQGEREF  337



Lambda      K        H        a         alpha
   0.316    0.135    0.378    0.792     4.96

Gapped
Lambda      K        H        a         alpha    sigma
   0.267   0.0410    0.140     1.90     42.6     43.6

Effective search space used: 245528
```

```
Matrix: BLOSUM62
Gap Penalties: Existence: 11, Extension: 1
Neighboring words threshold: 11
Window for multiple hits: 40




Query= sp|Q9ZT66|E134_MAIZE_Endo-1,3;1,
4-beta-D-glucanase_OS=Zea_mays_PE=1_SV=1

Length=303

Subject= 313076-61_2_ORF2
>sp|Q9ZT66|E134_MAIZE_Endo-1,3;1,
4-beta-D-glucanase_OS=Zea_mays_PE=1_SV=1|||1e-42

Length=245


 Score =   137 bits (344),  Expect = 1e-42, Method: Compositional
matrix adjust.
 Identities = 67/172 (39%), Positives = 103/172 (60%), Gaps = 7/172
(4%)

Query  55
GGLRAYVSGAASSSRAVVLASDVFGYEAPLLRQIVDKVAKAGYFVVVPDFLKGDYLDDKK  114
            G   +Y++   + + AV+L +D+FG++APLLR++ DK A AGYFVVVPDF   D
Sbjct  29
GPFLSYITAHHTPTAAVILVNDIFGFDAPLLRKLADKTASAGYFVVVPDFFNKDPFKPAD  88

Query  115  ------NFTEWLEAHSPVKAAEDAKPLFAALKKEG-
KSVAVGGYCWGGKLSVEVGKTSDV  167
                  NF +W++ H  + + +  + +   L K+G  SV   G+CWG K+ V++ K
++
Sbjct  89
SSNPFANFGDWIKKHGALDSVDGVQQIIEILHKKGFSSVGAVGFCWGAKVVVQIAKGHEL  148

Query  168  KAVCLSHPYSVTADDMKEVKWPIEILGAQNDTTTPPKEVYRFVHVLRERHEV
219
            KA  L+HP  VT +D+++VK PI IL A+ D TTPP    +F  +L  + EV
Sbjct  149  KAAVLAHPSFVTVEDIQDVKTPIAILAAETDNTTPPALAQKFADILESKPEV
200


 Score = 14.6 bits (26),  Expect = 2.5, Method: Compositional matrix
adjust.
 Identities = 6/14 (43%), Positives = 8/14 (57%), Gaps = 0/14 (0%)

Query  111  DDKKNFTEWLEAHS  124
            DD K   +  EAH+
Sbjct  221  DDAKAVADAEEAHN  234
```

```
Lambda      K        H        a         alpha
   0.321    0.134    0.405    0.792     4.96

Gapped
Lambda      K        H        a         alpha    sigma
   0.267   0.0410    0.140     1.90     42.6     43.6

Effective search space used: 61160




Query= sp|Q9ZT66|E134_MAIZE_Endo-1,3;1,
4-beta-D-glucanase_OS=Zea_mays_PE=1_SV=1

Length=303

Subject= 414359-18_3_ORF1
>sp|Q9ZT66|E134_MAIZE_Endo-1,3;1,
4-beta-D-glucanase_OS=Zea_mays_PE=1_SV=1|||2e-43

Length=247


 Score =   139 bits (350),  Expect = 2e-43, Method: Compositional
matrix adjust.
 Identities = 79/175 (45%), Positives = 104/175 (59%), Gaps = 6/175
(3%)

Query  52   DLPGGLRAYVSG---
AASSSRAVVLASDVFGYEAPLLRQIVDKVAKAGYFVVVPDFLKGD  108
            +L G   +YVS    A++   AV+LASDVFG+E PLLR++ DKVA  GY VVVPD+
GD
Sbjct  29
ELMGPFLSYVSHPPIASAPKAAVILASDVFGFEQPLLRKLADKVAALGYVVVVVPDYFNGD  88

Query  109  -YLDDKK-NFTEWLEAHSPVKAAEDAKPLFAALKKEGKS-
VAVGGYCWGGKLSVEVGKTS  165
             Y+ D   + T WL  H P+   E  K L   LK +G S V   G+CWG KL V +
K
Sbjct  89
PYVKDAGVDVTIWLPKHHPIDMVESTKSLVEVLKNKGLSLVGAIGFCWGAKLVVNLAKED  148

Query  166  DVKAVCLSHPYSVTADDMKEVKWPIEILGAQNDTTTPPKEVYRFVHVLRERHEVP
220
             +K   + HP  VT +D+++VK PI IL A+ DT TP   V ++  VL  + EVP
Sbjct  149  HLKVAIMCHPSLVTVEDIRDVKTPIAILAAEMDTITPSALVEQYREVLDSKEEVP
203


 Score = 13.5 bits (23),  Expect = 6.4, Method: Compositional matrix
```

```
adjust.
 Identities = 4/6 (67%), Positives = 5/6 (83%), Gaps = 0/6 (0%)

Query  101  VPDFLK  106
            VP F+K
Sbjct  202  VPSFVK  207



Lambda      K        H        a         alpha
   0.321    0.134    0.405    0.792     4.96

Gapped
Lambda      K        H        a         alpha    sigma
   0.267   0.0410    0.140     1.90     42.6     43.6

Effective search space used: 61160



Matrix: BLOSUM62
Gap Penalties: Existence: 11, Extension: 1
Neighboring words threshold: 11
Window for multiple hits: 40



Query= sp|Q9ZTR1|
SPD1_PEA_Spermidine_synthase_1_OS=Pisum_sativum_GN=SPDSYN1
_PE=2_SV=1

Length=334

Subject= 30962-455_2_ORF1
>sp|Q9ZTR1|SPD1_PEA_Spermidine_synthase_1_OS=Pisum_sativum_GN=SPDSYN
1_PE=2_SV=1|||0

Length=413


 Score =   499 bits (1286),  Expect = 0.0, Method: Compositional
matrix adjust.
 Identities = 227/292 (78%), Positives = 267/292 (91%), Gaps = 1/292
(0%)

Query  39
LSSVIPGWFSEISPMWPGEAHSLKVEKILFQGKSDYQDVMVFQSATYGKVLILDGVIQLT  98
            +SSVIPGWFSE+SPMWPGEAHSL+V++ILF+GKSD+QD++VFQSATYGKVL
+LDGVIQ+T
Sbjct  118
ISSVIPGWFSEVSPMWPGEAHSLEVKEILFEGKSDFQDILVFQSATYGKVLVLDGVIQVT  177
```

```
Query  99
ERDECAYQEMITHLPLCSIPNPKKVLVIGGGDGGVLREVARHSSVEKIDICEIDKMVVDV  158
            ERDECAYQEMITHLPLCSIPNP+KVLVIGGGDGGVLREVARH SVE
+IDICEIDKMV+DV
Sbjct  178
ERDECAYQEMITHLPLCSIPNPRKVLVIGGGDGGVLREVARHKSVEQIDICEIDKMVIDV  237

Query  159
SKEYFPDIAVGFADPRVTLNIGDGVAFLKAAPEGTYDAVIVDSSDPIGPAQELFEKPFFE  218
            +K++FPDIAVGFADPRV L +GDG  FLK   +G YDAVIVDSSDP+GPAQ+LFE
PFFE
Sbjct  238
AKKFFPDIAVGFADPRVKLFVGDGAIFLKEVSDGFYDAVIVDSSDPVGPAQQLFETPFFE  297

Query  219
SVARALRPGGVVCTQAESIWLHMHIIEDIVVNCRQVFKGSVNYAWTTVPTYPSGMIGFML  278
            S+AR LRPGGVVCTQAES+WLHM II+DI   CR+ FKGSVNYAWT+VPTYPSG
IGFML
Sbjct  298
SLARVLRPGGVVCTQAESLWLHMPIIKDIFSACRKTFKGSVNYAWTSVPTYPSGTIGFML  357

Query  279  CSTEGPSVDFKHPVNPIDE-NDSQQAARPLKFYNREIHSAAFCLPSFAKRAI
329
            CSTEGP+V+F+ P+NPI++  ++ +  RPLKFYN E+HS+AFCLP FA++A+
Sbjct  358  CSTEGPAVNFRQPINPIEKIEEADRNTRPLKFYNSEMHSSAFCLPQFARQAL
409



Lambda      K        H        a         alpha
   0.318    0.135    0.408    0.792     4.96

Gapped
Lambda      K        H        a         alpha    sigma
   0.267   0.0410    0.140     1.90     42.6     43.6

Effective search space used: 116432




Matrix: BLOSUM62
Gap Penalties: Existence: 11, Extension: 1
Neighboring words threshold: 11
Window for multiple hits: 40




Query= sp|Q9ZVD0|
SRRT_ARATH_Serrate_RNA_effector_molecule_OS=Arabidopsis_th
aliana_GN=SE_PE=1_SV=2
```

Length=720

Subject= 284827-77_1_ORF2
>sp|Q9ZVD0|SRRT_ARATH_Serrate_RNA_effector_molecule_OS=Arabidopsis_t
haliana_GN=SE_PE=1_SV=2|||0

Length=796

Score = 520 bits (1340), Expect = 2e-177, Method: Compositional
matrix adjust.
Identities = 332/702 (47%), Positives = 424/702 (60%), Gaps =
99/702 (14%)

```
Query  98
RDRRHSPPQRRSPPQKRYRRDDNGYDGRRGSPRGGYGPPDRRFGYDHGGGYDREMGGRPG  157
            R+RR SP +R     KR RR+D+   GRRGSPR G      R     GG  +   G
Sbjct  115
RERRISPLRRSPSSYKRSRREDDYDGGRRGSPRMGMDDRRDRRMGGGGGRSNSYSG----  170

Query  158  YGDERPHGRFMGRYQDWEGGRGGYGDA--
SNSGNPQRDGLMSYKQFIQELEDDILPSEAE  215
              DER +GR  G   ++  GRGG+ D   S    P+R+GLM+YKQFI ELEDDI+P
+EAE
Sbjct  171  --DERSYGRHHGFRPEF--
GRGGFADGPFSYDVGPRREGLMTYKQFITELEDDIIPTEAE  226

Query  216
RRYQEYKSEYITTQKRAFFNTHKEEDWLKNKYHPTNLLSVIERRNDLAQKVAKDFLLDLQ  275
            RRY EY++E+I+TQK+A+F  +K+EDWL++KY P+ L +VI+RRN+  +  AK+F
+L+L+
Sbjct  227
RRYTEYRNEFISTQKKAYFEQNKQEDWLRDKYDPSRLEAVIQRRNEACKTAAKEFILELE  286

Query  276  SGTLDLGPAVTALNKSG--
RTSEPNSEDEAAGVGKRKRHGMGGAKENELLSAAPKAPSFT  333
            SG+LD+GP V   N  G    SE  ++D       R+R+G G AKE E
APKAP+
Sbjct  287  SGSLDIGPNVVGQNPQGAQEVSEEETDD-------RRRNGRGSAKEQEF--
DAPKAPAIF  337

Query  334
SDPKRILTDVEQTQALVRKLDSEKKIEENVLQGSETEKSGREKLHSGSTGPVVIIRGLTS  393
             +P+RI  DVEQ +ALVRKLD EK IE N+L  SE +KS  E+  SG    +V+
+RG
Sbjct  338  CEPRRIEKDVEQARALVRKLDGEKGIERNILSTSEMDKSDGER--
SGGKMNIVVVRGANH  395

Query  394  VKGLEGVELLDTLVTYLWRVHGLDYYGKVETNEA-KGLRHVRAEGKVSD--
AKGDENESK  450
            V+G EGVELLD ++TYLWRVH +DYYG  E  E  K +RHVR +GKV++  A   E
E K
Sbjct  396
```

```
VQGYEGVELLDVVITYLWRVHYVDYYGFKEYKEQPKVMRHVRGDGKVNEDMASSVEWEKK  455

Query  451  
FDSHWQERLKGQDPLEVMAAKEKIDAAATEALDPHVRKIRDEKYGWKYGCGAKGCTKLFH  510
             D  WQ R++GQD LE+M  KE++++A   ALDP +RKI+DEKYGWKYGCGAK
CTKLFH
Sbjct  456  
VDGTWQGRIQGQDLLELMLGKERMESATLLALDPLIRKIKDEKYGWKYGCGAKNCTKLFH  515

Query  511  AAEFVYKHLKLKHTELVTELTTKVREELYFQNYMNDPNAPGGQP--
ATQQ---------S  559
              EFV KHLKLKH EL+ ++  KV EELYF+NYM+D +APG  P  A+Q+
S
Sbjct  516  
GPEFVQKHLKLKHPELIQDVAVKVYEELYFENYMSDADAPGSTPVMASQKDRPRRPPRPS  575

Query  560  GPRDRPIRRK-------PSMENRLRDDRGGRRERDG---------------
RANGNDRND  597
             P D P R         PS       DRGGR  R+
ND++
Sbjct  576  AP-
DEPARISAGLPLPAPSRGGSREADRGGRGARESDKVDKPEKVQEDEQFEQRNNDQSP  634

Query  598  RSEDQQRGD-NDGGNPGEVG------
YDAFGGQGGVHVPPFLSDINPPPMLMPVPGAGPL  650
              + QQ G   D   P E G      +D F G GG+  PPF +D+  PP
+LMPVPGAGPL
Sbjct  635  
SQDYQQSGGPYDSAGPFEGGRGDTQMFDPFSGPGGMRGPPFGADMGMPPVLMPVPGAGPL  694

Query  651  GPFVPAPPEVAMQMFRDPSGPNP----------PFEGSG--------
RGGPA------PFL  687
            GPFVPAPPEVAM+++R+  G  P         PF+  G        R GP+
L
Sbjct  695  
GPFVPAPPEVAMRLWREQGGAGPFHPAGVYDGPFDSEGGNSRGNRKRAGPSGGGRMGAGL  754

Query  688  LSPA--------FRQDPRR-LRSYQDLDAPEEEVTVIDYRSL  720
            +            R D RR LRSY+DLDAPE+EVTVIDYRSL
Sbjct  755  IDTPPLPLPLPNMRPDARRPLRSYRDLDAPEDEVTVIDYRSL  796



Lambda      K        H        a         alpha
   0.312    0.135    0.407    0.792     4.96

Gapped
Lambda      K        H        a         alpha    sigma
   0.267   0.0410    0.140     1.90     42.6     43.6

Effective search space used: 514080
```

```
Matrix: BLOSUM62
Gap Penalties: Existence: 11, Extension: 1
Neighboring words threshold: 11
Window for multiple hits: 40
```
