# Supplementary material for: Proteogenomic Analysis Greatly Expands the Identification of Proteins Related to Reproduction in the Apogamous Fern Dryopteris affinis ssp. affinis
Source: Front Plant Sci. 2017 Mar 22;8:336. doi: 10.3389/fpls.2017.00336 (PMC5360702; doi:10.3389/fpls.2017.00336)

Figure S3

Assigned Peptide-Spectrum-Matches for Single Hit Proteins

Screenshots from Scaffold for proteins with a single peptide sequence (single hit proteins)

Figure S3

Argonaute 10

>sp|Q9XGW1|AGO10_ARATH_Protein_argonaute_10_OS=Arabidopsis_thaliana_GN=AGO10_PE=2_SV=1|||3e-161   429495-5_4_ORF273 kDa   98% (1) 97% (1)   96% (1)

>sp|Q9XGW1|AGO10_ARATH_Protein_argonaute_1...   All Biological Samples

Sequence Coverage	Protein	Accession	Category	Bio Sample	MS/MS Sa...	Prob	%Spec	#Pep	#Uniq...	#S
	>sp|Q9X...	429495-...	filament	jonas Co...		98%	0.001...	1	1	
	>sp|Q9X...	429495-...	filament	jonas Co...		97%	0.002...	1	1	
	>sp|Q9X...	429495-...	heart	jonas Co...		96%	0.002...	1	1	

Valid	W...	Sequence	Prob	Masco...	Masco...	Masco...	NTT	Modifications	Observed	Actual Mass	Charge	Delta
☑	1.0	(R)SIIIGADVSHPR(V)	100%	68.0	41.9	34.7	2		632.86	1,263.70	2	0.00

Protein Sequence | Similar Proteins | Spectrum | Spectrum/Model Error | Fragmentation Table

429495-5_4_ORF2 (98%), 73,487.6 Da
>sp|Q9XGW1|AGO10_ARATH_Protein_argonaute_10_OS=Arabidopsis_thaliana_GN=AGO10_PE=2_SV=1|||3e-161
1 exclusive unique peptides, 1 exclusive unique spectra, 1 total spectra, 12/648 amino acids (2% coverage)

```
DLLLMALQKR  ASGSSAGQEA  FLLRRPGFGQ  FGDPTAVFAN  HFRVRFEAKR  LYHYDVSIEP  EPPKGLHRAI  MDQAVAKHGE
KLQGAIPAYD  GQRALYTARE  LAEQVELEVQ  LTQDNEGPRR  RNNLFKITLR  EASKIDMGLL  HEYLNGKRRI  LPQENLQVLD
IVLREIACKY  FLAKGRCFFG  SHFGESNLGG  GIQAYSGFYQ  SIRPAQNGLL  SLNIDIAAAP  FLESVCLDDF  LGKAFRKDVR
ALNLELGRPD  GYGDQARVKA  KKLLKGIRVE  TIHNRGAKRK  YRIQSITNEP  LRNLRFDMDG  VSVSVVDYFR  RTYNYEIKFP
GLPAVESGTR  ERKRYLPMEV  CKIVAGQPYM  KSMSEAQKKA  LLRVTCVLPG  ERKKTTEKIE  VDLVKETSKI  AKEFGVGINR
FLTEIPARVL  PAPSIQYHSA  TVQPPPGLGS  WNMRNKRMVS  GCQINRWSLI  NFCHNLNESD  VKKFCDKLPR  ECETYGLAMN
PEMAVEPLTE  RRGQAEGAIK  HLNRQCAEKF  RQDLDLLFCI  MPQTDNKSIY  AAVKRVCEIE  LGLVTQCCLS  EHVKKNRPEY
IANLLLKVNA  KMGGYNARLV  DEQNRRLPLI  SEVRSIIIGA  DVSHPRVGDD  YSPSISAVAA  SMDWPYFSQY  EAIVQTQRQR
EEVCEEFF
```

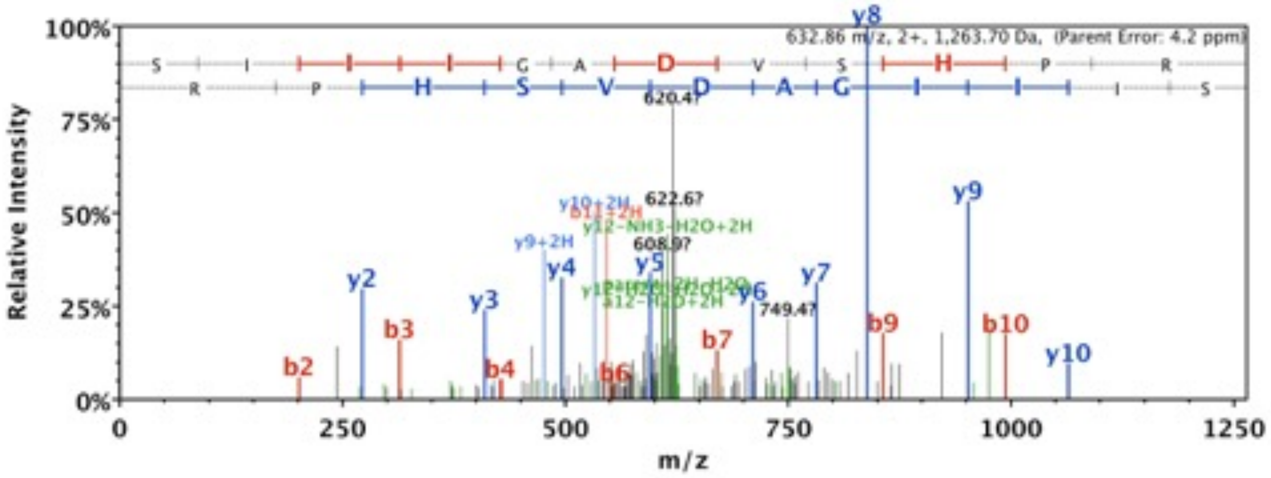

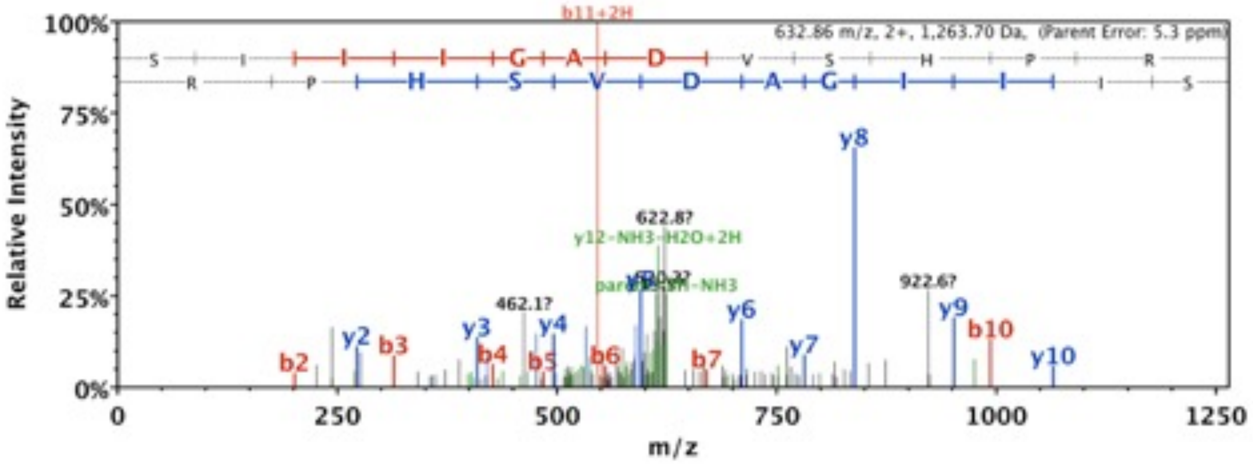

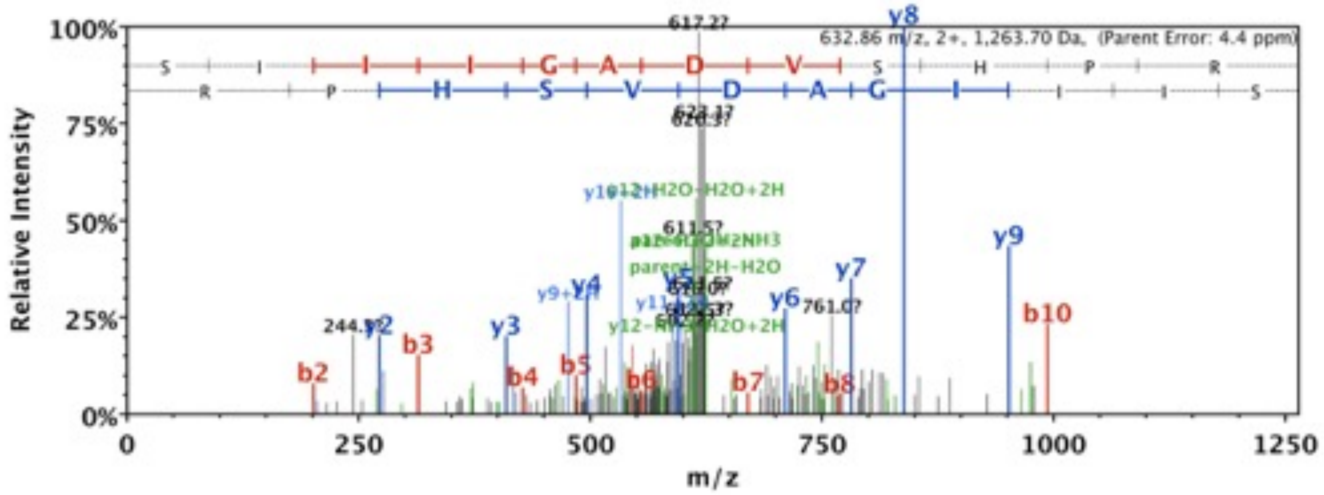

Figure S3

Serrate

>sp|Q9ZVD0|SRRT_ARATH_Serrate_RNA_effector_molecule_OS=Arabidopsis_thaliana_GN=SE_PE=1_SV=2|||0 284827-77_1_ORF2 90 kDa 100% (1) 100% (0)

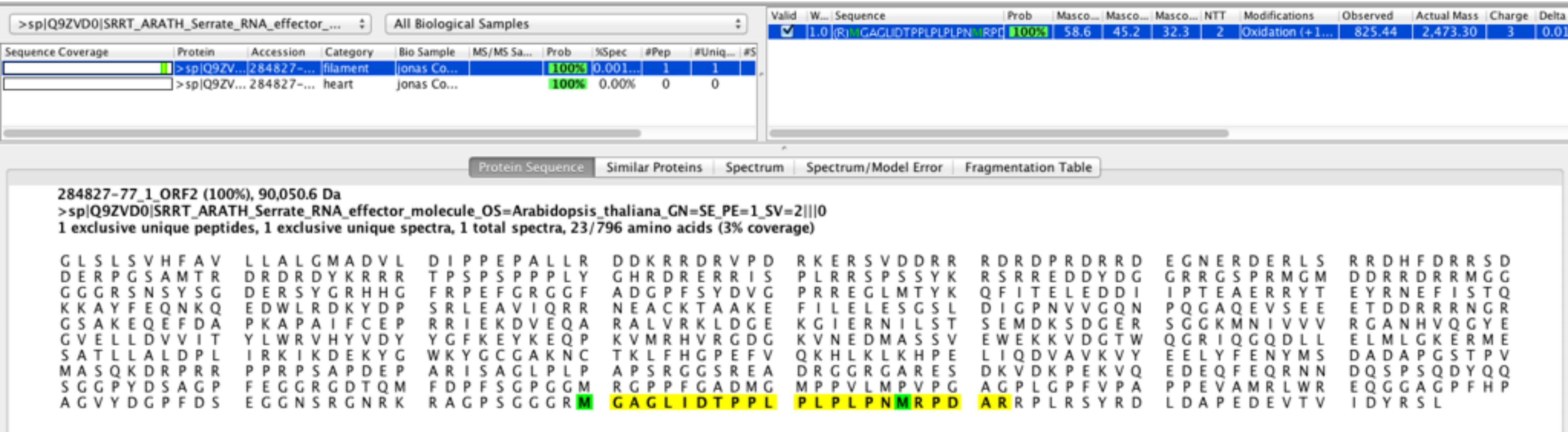

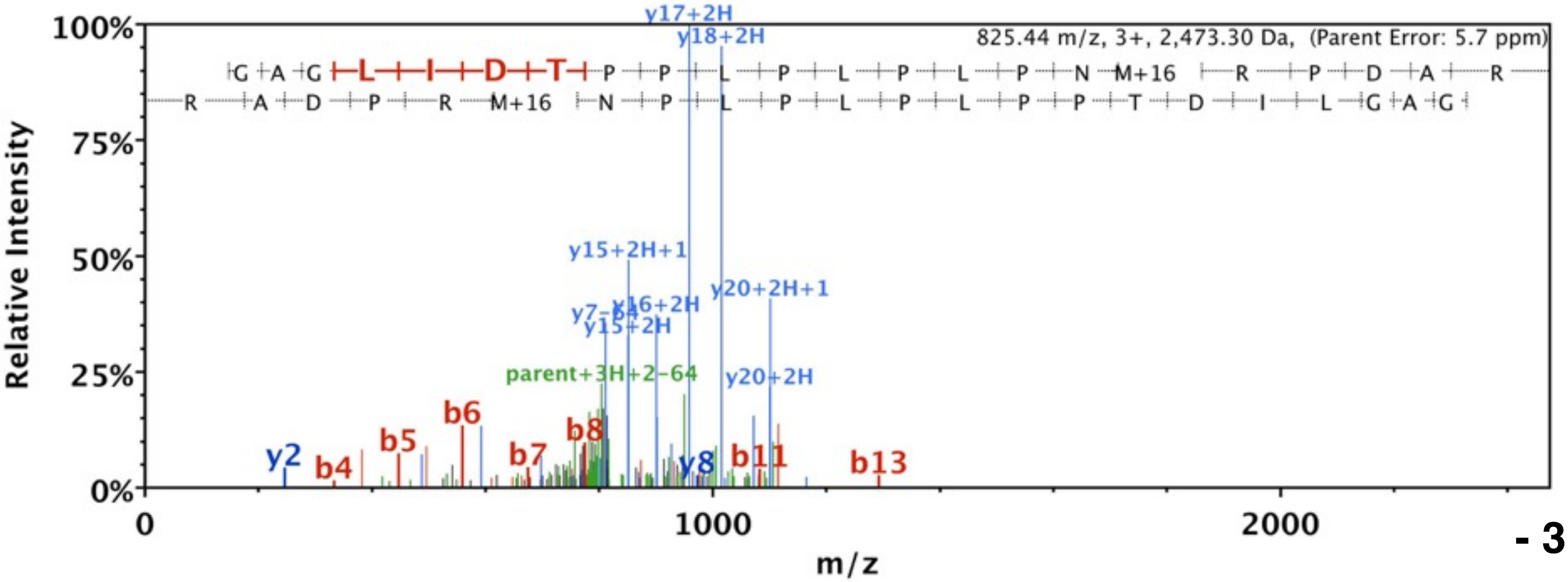

Figure S3

Spermidine Synthase

>sp|Q9ZTR1|SPD1_PEA_Spermidine_synthase_1_OS=Pisum_sativum_GN=SPDSYN1_PE=2_SV=1|||0 30962-455_2_ORF1 (+39)   45 kDa   98% (2) 99% (0)

>sp|Q9ZTR1|SPD1_PEA_Spermidine_synthase_1_O...   All Biological Samples

Sequence Coverage	Protein	Accession	Category	Bio Sample	MS/MS Sa...	Prob	%Spec	#Pep	#Uniq...	#S
	>sp|Q9ZT...	30962-4...	heart	jonas Co...		98%	0.004...	1	1	
	>sp|Q9ZT...	30962-4...	heart	jonas Co...		99%	0.00%	0	0	

Valid	W...	Sequence	Prob	Masco...	Masco...	Masco...	NTT	Modifications	Observed	Actual Mass	Charge	Delta
☑	1.0	(R)KVLVIGGGDGGVLR(E)	100%	47.6	37.4	0.0	2		447.27	1,338.80	3	0.00
☑	1.0	(R)KVLVIGGGDGGVLR(E)	99%	41.3	37.4	0.0	2		447.27	1,338.80	3	0.00

Protein Sequence | Similar Proteins | Spectrum | Spectrum/Model Error | Fragmentation Table

30962-455_2_ORF1 (98%), 44,850.3 Da
>sp|Q9ZTR1|SPD1_PEA_Spermidine_synthase_1_OS=Pisum_sativum_GN=SPDSYN1_PE=2_SV=1|||0
1 exclusive unique peptides, 1 exclusive unique spectra, 2 total spectra, 14/413 amino acids (3% coverage)

```
GTMWKAFTCA KAPLERSAAV FAYKRPPAMA APYSSSPSPR EESSLCTHTE KFVGSSSSSS PSPSPSSVCG VDSSPSKRLC
CDSTLASSGY ISSSELCLRS SSAAHMEGVK PGSGRANISS VIPGWFSEVS PMWPGEAHSL EVKEILFEGK SDFQDILVFQ
SATYGKVLVL DGVIQVTERD ECAYQEMITH LPLCSIPNPR KVLVIGGGDG GVLREVARHK SVEQIDICEI DKMVIDVAKK
FFPDIAVGFA DPRVKLFVGD GAIFLKEVSD GFYDAVIVDS SDPVGPAQQL FETPFFESLA RVLRPGGVVC TQAESLWLHM
PIIKDIFSAC RKTFKGSVNY AWTSVPTYPS GTIGFMLCST EGPAVNFRQP INPIEKIEEA DRNTRPLKFY NSEMHSSAFC
LPQFARQALD YTS
```

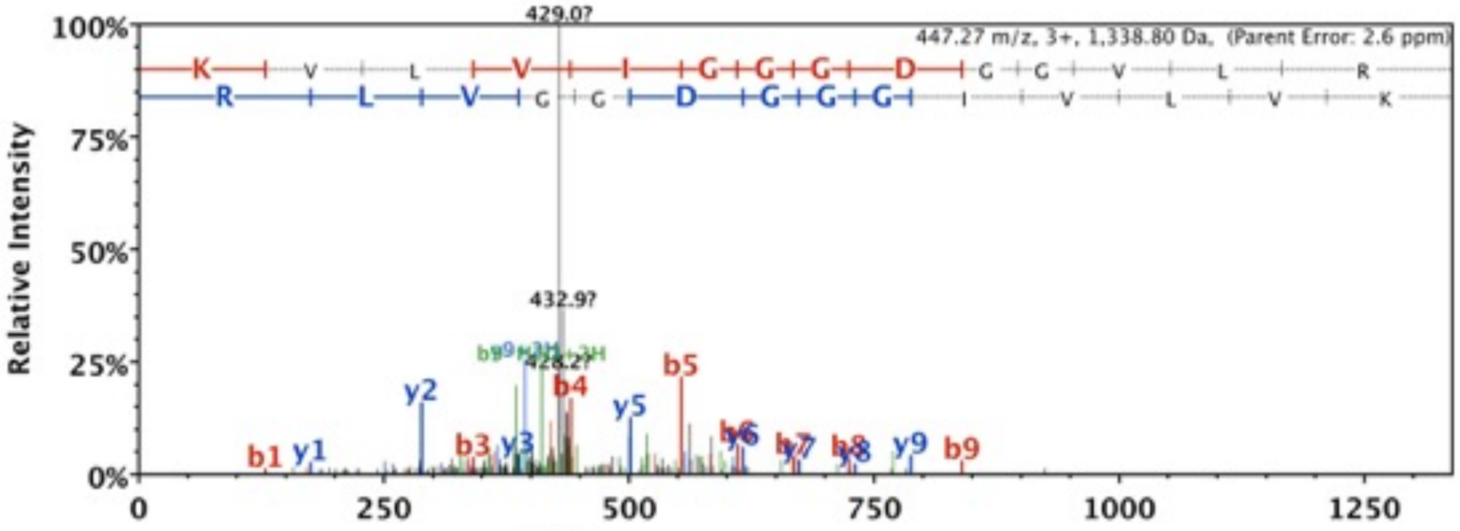

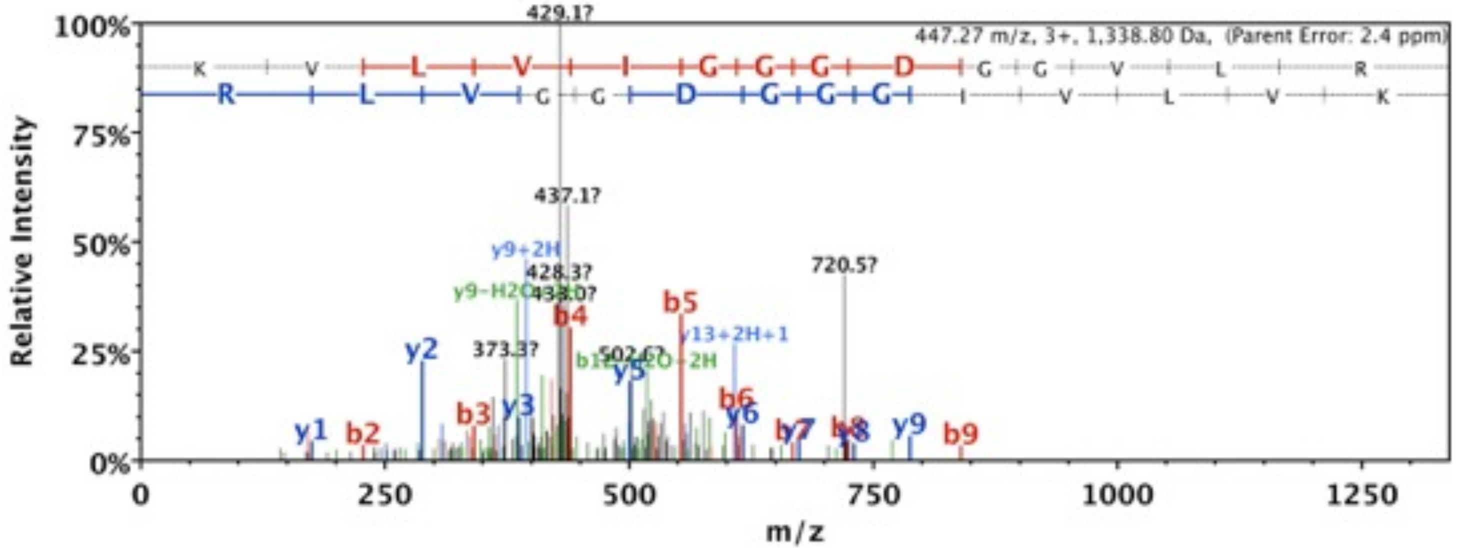

Figure S3

Tryptophan Synthase (beta chain 2)

>sp|Q8TL44|TRPB2_METAC_Tryptophan_synthase_beta_chain_2_OS=Methanosarcina_acetivorans_(strain_ATCC_35395_/_DSM_2834_/_JCM_12185_/_C2A)_GN=trpB2_PE=3_SV=1|||4e-170
158601-182_4_ORF1 (+1) 51 kDa 12% (0) 97% (0) 96% (1)

>sp|Q8TL44|TRPB2_METAC_Tryptophan_synthase_be... All Biological Samples

Sequence Coverage	Protein	Accession	Category	Bio Sample	MS/MS Sa...	Prob	%Spec	#Pep	#Uniq...	#Spec
	>sp|Q8TL...	158601-...	filament	jonas Co...		12%	0.00%	0	0	0
	>sp|Q8TL...	158601-...	heart	jonas Co...		97%	0.00%	0	0	0
	>sp|Q8TL...	158601-...	heart	jonas Co...		96%	0.002...	1	1	1

Valid	W...	Sequence	Prob	Masco...	Masco...	Masco...	NTT	Modifications	Observed	Actual Mass	Charge	Delta Da
✓	1.0	(R)RLEAFLKTPAR(I)	99%	43.6	39.4	17.2	2		434.60	1,300.77	3	0.0044

Protein Sequence | Similar Proteins | Spectrum | Spectrum/Model Error | Fragmentation Table

158601-182_4_ORF1 (96%), 51,157.7 Da
>sp|Q8TL44|TRPB2_METAC_Tryptophan_synthase_beta_chain_2_OS=Methanosarcina_acetivorans_(strain_ATCC_35395_/_DSM_2834_/_JCM_12185_/_C2A)_GN=trpB2_PE=3_SV=1|||4e-170
1 exclusive unique peptides, 1 exclusive unique spectra, 1 total spectra, 11/464 amino acids (2% coverage)

```
RCVCVCVDSR ERERVSALYV RLLEQQSVKR GMALLNLITT THSLPLCHSQ LTSSFFPSIK FLRDSPDIYS TKKPCLSTMA
HKDFDGKRLG FGRKITLPDN DMPQQWYNII ADLPKPPPSP LHPKTLQPLK PEDLMPLFPQ ALIEQEGSLE AYINIPEEVL
DIYSMWRPTP LFRARRLEAF LKTPARIYYK YEGGSPAGSH KPNTAVPQAW YNAQAGVKRL TTETGAGQWG SALAFACSHF
GIGCEVWQVR ASYDQKPYRK IMMETWAAKV HPSPSTLTAA GRSILEKDPS SPGSLGIAVS EAVEAALADD NTKYSLGSIL
NHVLLHQTII GEECVKQLAM VEETPDVIIG CTGGGSNFSG IAFPFIREKM KGKMNPIIRA VEPAACPSLT KGVYAYDFGD
AAGLTPLLKM HTLGHDFVPD PIHAGGLRYH GMAPLVSHLY NLGFMEAIAI PQIECFEAAL NFAR
```

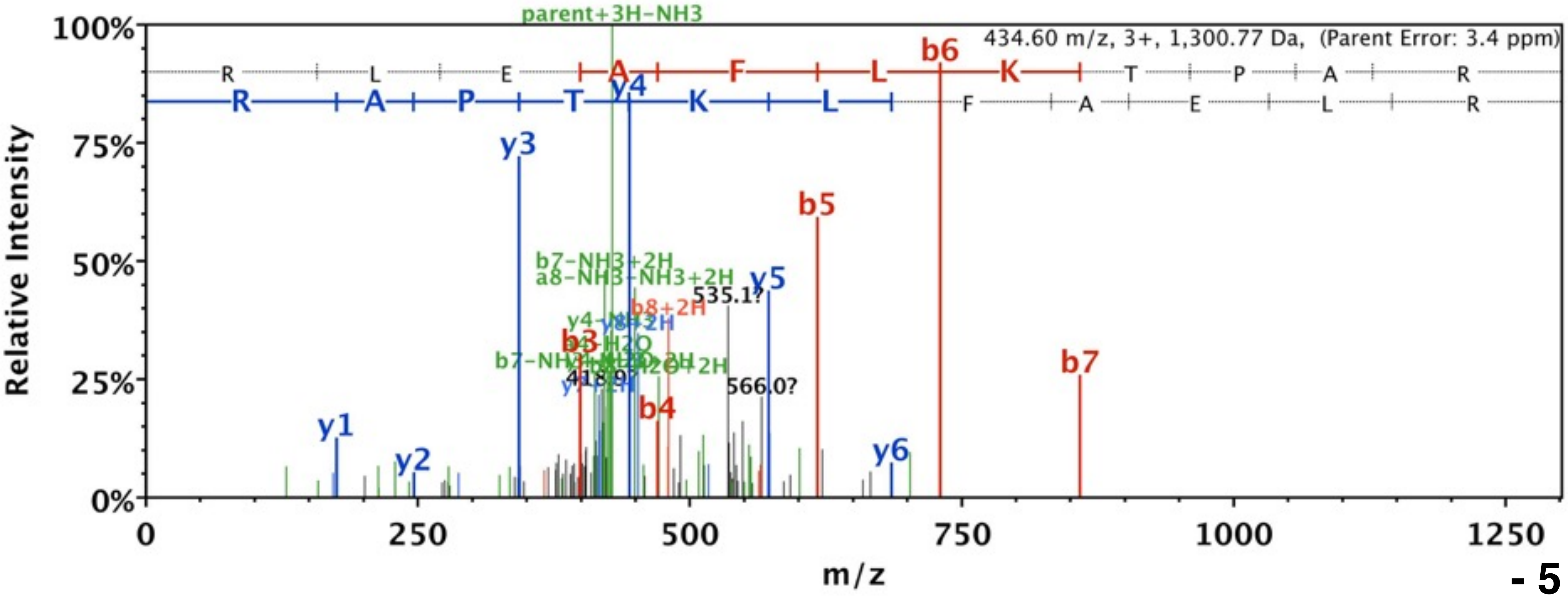

Figure S3

Brefeldin

>sp|F4JSZ5|BIG1_ARATH_Brefeldin_A-inhibited_guanine_nucleotide-exchange_protein_1_OS=Arabidopsis_thaliana_GN=BIG1_PE=2_SV=1|||0 106730-241_3_ORF2 199 kDa

77% (0) 100% (1)

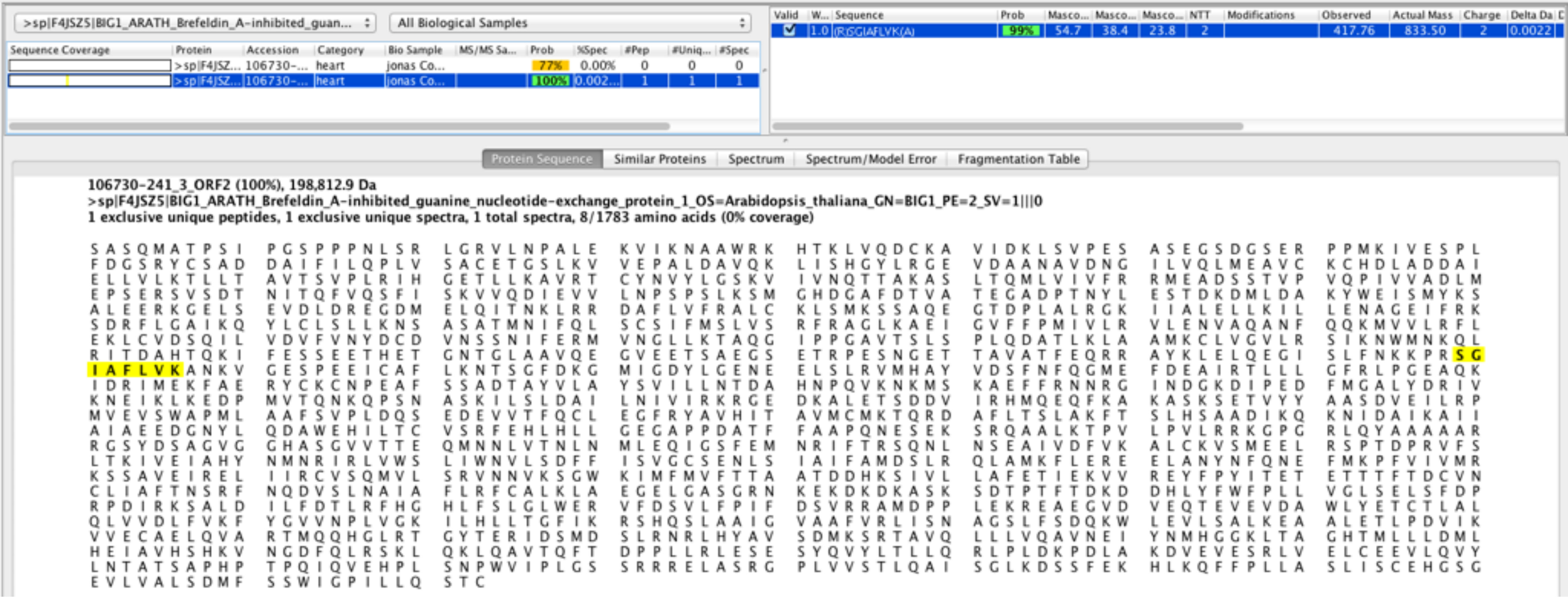

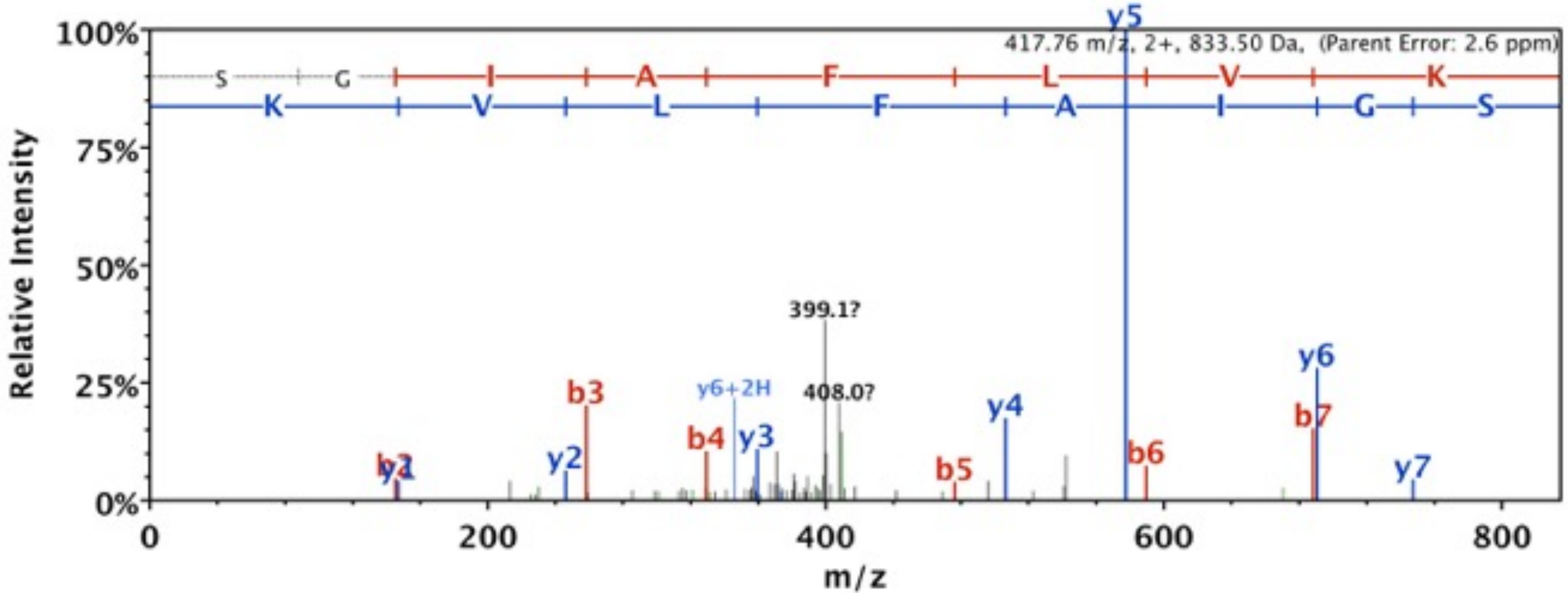

Supplement: Figure S3 — Peptide-spectrum-match assignments for proteins identified with only one single peptide sequence. [file Image3.PDF]
