# Supplementary material for: Proteogenomic Analysis Greatly Expands the Identification of Proteins Related to Reproduction in the Apogamous Fern Dryopteris affinis ssp. affinis
Source: Front Plant Sci. 2017 Mar 22;8:336. doi: 10.3389/fpls.2017.00336 (PMC5360702; doi:10.3389/fpls.2017.00336)
Supplement: Table S1 — Oligonucleotide sequences for truSeq™RNA. [file Table1.DOCX]

**Table S 1 - Adapter Sequences**

**Oligonucleotide sequences for TruSeq™ RNA and DNA Sample Prep Kits**

**TruSeq Universal Adapter**

5’ AATGATACGGCGACCACCGAGATCTACACTCTTTCCCTACACGACGCTCTTCCGATCT

**TruSeq™ Adapters**

**TruSeq Adapter, Index 1**

5’ GATCGGAAGAGCACACGTCTGAACTCCAGTCAC**ATCACG**ATCTCGTATGCCGTCTTCTGCTTG

**TruSeq Adapter, Index 2**

5’ GATCGGAAGAGCACACGTCTGAACTCCAGTCAC**CGATGT**ATCTCGTATGCCGTCTTCTGCTTG

**TruSeq Adapter, Index 3**

5’ GATCGGAAGAGCACACGTCTGAACTCCAGTCAC**TTAGGC**ATCTCGTATGCCGTCTTCTGCTTG

**TruSeq Adapter, Index 4**

5’ GATCGGAAGAGCACACGTCTGAACTCCAGTCAC**TGACCA**ATCTCGTATGCCGTCTTCTGCTTG

**TruSeq Adapter, Index 5**

5’ GATCGGAAGAGCACACGTCTGAACTCCAGTCAC**ACAGTG**ATCTCGTATGCCGTCTTCTGCTTG

**TruSeq Adapter, Index 6**

5’ GATCGGAAGAGCACACGTCTGAACTCCAGTCAC**GCCAAT**ATCTCGTATGCCGTCTTCTGCTTG

**TruSeq Adapter, Index 7**

5’ GATCGGAAGAGCACACGTCTGAACTCCAGTCAC**CAGATC**ATCTCGTATGCCGTCTTCTGCTTG

**TruSeq Adapter, Index 8**

5’ GATCGGAAGAGCACACGTCTGAACTCCAGTCAC**ACTTGA**ATCTCGTATGCCGTCTTCTGCTTG

**TruSeq Adapter, Index 9**

5’ GATCGGAAGAGCACACGTCTGAACTCCAGTCAC**GATCAG**ATCTCGTATGCCGTCTTCTGCTTG

**TruSeq Adapter, Index 10**

5’ GATCGGAAGAGCACACGTCTGAACTCCAGTCAC**TAGCTT**ATCTCGTATGCCGTCTTCTGCTTG

**TruSeq Adapter, Index 11**

5’ GATCGGAAGAGCACACGTCTGAACTCCAGTCAC**GGCTAC**ATCTCGTATGCCGTCTTCTGCTTG

**TruSeq Adapter, Index 12**

5’ GATCGGAAGAGCACACGTCTGAACTCCAGTCAC**CTTGTA**ATCTCGTATGCCGTCTTCTGCTTG

**TruSeq Adapter, Index 13**

5’GATCGGAAGAGCACACGTCTGAACTCCAGTCAC**AGTCAA**CAATCTCGTATGCCGTCTTCTGCTTG

**TruSeq Adapter, Index 14**

5’ GATCGGAAGAGCACACGTCTGAACTCCAGTCAC**AGTTCC**GTATCTCGTATGCCGTCTTCTGCTTG

**TruSeq Adapter, Index 15**

5’ GATCGGAAGAGCACACGTCTGAACTCCAGTCAC**ATGTCA**GAATCTCGTATGCCGTCTTCTGCTTG

**TruSeq Adapter, Index 16**

5’ GATCGGAAGAGCACACGTCTGAACTCCAGTCAC**CCGTCC**CGATCTCGTATGCCGTCTTCTGCTTG

**TruSeq Adapter, Index 18** 4

5’ GATCGGAAGAGCACACGTCTGAACTCCAGTCAC**GTCCGC**ACATCTCGTATGCCGTCTTCTGCTTG

**TruSeq Adapter, Index 19**

5’ GATCGGAAGAGCACACGTCTGAACTCCAGTCAC**GTGAAA**CGATCTCGTATGCCGTCTTCTGCTTG

**TruSeq Adapter, Index 20**

5’ GATCGGAAGAGCACACGTCTGAACTCCAGTCAC**GTGGCC**TTATCTCGTATGCCGTCTTCTGCTTG

**TruSeq Adapter, Index 21**

5’ GATCGGAAGAGCACACGTCTGAACTCCAGTCAC**GTTTCG**GAATCTCGTATGCCGTCTTCTGCTTG

**TruSeq Adapter, Index 22**

5’ GATCGGAAGAGCACACGTCTGAACTCCAGTCAC**CGTACG**TAATCTCGTATGCCGTCTTCTGCTTG

**TruSeq Adapter, Index 23**

5’ GATCGGAAGAGCACACGTCTGAACTCCAGTCAC**GAGTGG**ATATCTCGTATGCCGTCTTCTGCTTG

**TruSeq Adapter, Index 25**

5’ GATCGGAAGAGCACACGTCTGAACTCCAGTCAC**ACTGAT**ATATCTCGTATGCCGTCTTCTGCTTG

**TruSeq Adapter, Index 27**

5’ GATCGGAAGAGCACACGTCTGAACTCCAGTCAC**ATTCCT**TTATCTCGTATGCCGTCTTCTGCTTG
